# Solasonine Induces Apoptosis and Inhibits Proliferation of Bladder Cancer Cells by Suppressing NRP1 Expression

**DOI:** 10.1155/2022/7261486

**Published:** 2022-03-02

**Authors:** Yang Dong, Lin Hao, Zhen-duo Shi, Kun Fang, Hui Yu, Guang-hui Zang, Tao Fan, Cong-hui Han

**Affiliations:** ^1^Department of Urology, Xuzhou Central Hospital, Xuzhou, Jiangsu Province, China; ^2^Xuzhou Clinical School of Xuzhou Medical University, Xuzhou, Jiangsu Province, China; ^3^Medical College of Soochow University, Soochow, Jiangsu Province, China; ^4^Department of Nephrology, The Third Affiliated Hospital of Shandong First Medical University, Jinan, Shandong Province, China; ^5^Department of Urology, Yantai Hospital of Traditional Chinese Medicine, Yantai, Shandong Province, China

## Abstract

Solasonine, a steroidal alkaloid extracted from *Solanum nigrum* L., has been found to exert inhibitory effect on cancers. However, the underlying anticancer mechanisms of solasonine, particularly in urinary bladder cancer (BC), remain unclear. In this study, we identified the potential targets and biological functions associated with solasonine activity using a bioinformatics approach. Ingenuity pathway analysis revealed that neuropilin-1 (NRP1) and other signaling pathways, such as PI3K/AKT and ERK/MAPK pathways, were potentially involved in the therapeutic effects of solasonine. The ability of solasonine in inducing apoptosis and inhibiting proliferation in BC cells was confirmed experimentally, and the inhibition of ERK/MAPK, P38/MAPK, and PI3K/AKT pathways was validated by Western blot. Mechanistically, solasonine suppressed the expression of NRP1 protein, but not that of mRNA. Further results of molecular docking and molecular dynamics simulation analysis indicated that solasonine could directly bind to the b1 domain of NRP1 protein with a reasonable and stable docking conformation. We previously found that targeting NRP1 is a potential antitumor strategy. Combined with these findings, it can be speculated that the binding of solasonine with NRP1 on the cell membrane could prevent the formation of NRP1/VEGFA/VEGFR2 and NRP1/EGFR complexes, resulting in the inhibition of downstream signaling, including ERK/MAPK, P38/MAPK, and PI3K/AKT pathways. Additionally, intracellular solasonine could inhibit the membrane localization of NRP1 and provoke its cytoplasmic retention, facilitating the degradation of NRP1 protein in the cytoplasm. The dual effects induced by the binding of solasonine to NRP1 extracellularly and intracellularly could account for the antiproliferative and proapoptotic effects of solasonine on BC.

## 1. Introduction

Urinary bladder cancer (BC), one of the leading causes of cancer-associated death, is the tenth most common malignancy worldwide. According to the Global Cancer Statistics, approximately 570,000 new cases and 210,000 BC-related deaths were recorded during 2020 [1]; and these values increase annually, with approximately 81,180 new cases and 17,100 deaths expected to occur in the United States in 2022 [2]. Urothelial carcinoma is the most prevalent histological phenotype and accounts for approximately 90% of BC cases. Due to its high recurrence rate and imperceptible symptoms, the long-term outcomes and quality of life of BC patients remain poor. Postoperative recurrence has been reported in approximately 70% of patients, and approximately 20% of these patients may progress to muscle invasion with distant metastatic potential [3–5]. Metastatic BC has a poor prognosis with a 5-year survival rate of only 5% [5, 6]. Moreover, based on tumor stage and grade, long-term follow-up endoscopic examinations and treatment with intravesical Bacillus Calmette–Guérin instillation are required, together with cisplatin-based combination chemotherapy and radiotherapy. Consequently, BC is associated with high financial and psychological burdens.

Emerging immunotherapeutic approaches such as checkpoint inhibitors and tumor-targeted CAR-T cells have shown efficacy in improving the prognosis of patients with advanced BC, but only a fraction of patients can achieve durable clinical responses [6–9]. Moreover, high costs restrict the clinical application of these approaches. Therefore, exploring novel, affordable, and efficacious anticancer drugs with low toxicity is of great clinical importance. Currently, molecular-targeted therapy against cancer has attracted attention worldwide, presenting novel frontiers in BC treatment. Molecular targeting agents such as those targeting vascular endothelial growth factor (VEGF), epidermal growth factor receptor (EGFR), fibroblast growth factor receptor, and mammalian target of rapamycin have been successfully used in several types of cancers, presenting encouraging results in extending patient survival [6, 7, 10]. In addition, certain factors have been identified in the occurrence and progression of BC, including Erb-B2 receptor tyrosine kinase 2, avian myelocytomatosis viral oncogene homolog (MYC), cyclin D1, and hypoxia-inducible factor 1*α* (HIF-1*α*), that are important for clinical applications [6, 10]. Cancer development is a multistage process involving aberrant cell proliferation, apoptosis, invasion, metastasis, and metabolism that are typically linked to the deregulation of tumor-related genes [11]. These regulatory factors are candidate targets associated with improved treatment efficacy for BC.

Natural plants are an important source of anticancer agents. *Solanum nigrum* L. (S. nigrum) is an annual herbaceous plant that has been used as a folklore medicine in China for BC therapy since ancient times [12, 13]. In a previous study, we explored the potential mechanisms underlying *S. nigrum* activity against BC. It was found that it exerted its therapeutic effects via multiple signaling pathways, including the MAPK, PI3K/AKT, and HIF-1 pathways [13]. *S. nigrum* contains two main kinds of effective components, namely, solanine and *Solanum nigrum* polysaccharide [14]. Solasonine is the major contributor to glycoalkaloid solanine, which can be extracted from all parts of the plant and unripe fruits [14, 15]. Since it was first identified in *Solanum* by Oddo and Cesaris in 1911, solasonine has been isolated from over 100 species and has shown beneficial pharmacological effects, such as anticancer and antimicrobial activities [14, 16]. Accumulating studies have suggested that solasonine has multiple antitumor activities, including suppression of proliferation, promotion of apoptosis, and inhibition of metastasis in a variety of tumor cells [17–19]. Solasonine could suppress glioma growth by inhibiting proinflammatory mediators and the activation of the P38/MAPK and JNK/MAPK pathways [20]. The activation of AMPK/FOXO3a axis has also been reported to be involved in the inhibition of cell proliferation mediated by solasonine in acute monocytic leukemia. [17]. In addition, another study showed that solasonine is a potential anticancer agent candidate for hepatocellular carcinoma, which can mediate the reciprocal regulation of miR-375-3p and lncRNA CCAT1, resulting in the downregulation of interferon regulatory factor 5 [18]. However, the effects of solasonine on BC have not been studied, and the underlying anticancer mechanism remains to be elucidated.

Neuropilin-1 (NRP1) is a novel target and immunomodulator for cancer therapy [21–24]. Our previous study demonstrated that NRP1 was overexpressed in BC, while downregulation of NRP1 promoted BC cell apoptosis and inhibited BC cell proliferation, invasion, and migration [24]. In the present study, we identified the potential targets and pathways associated with the activity of solasonine against BC using a bioinformatics approach. We found that solasonine may interact with NRP1 protein. We further explored the potential mechanism by which solasonine influences tumor biology in BC and its effect on NRP1 expression *in vitro*. Our results demonstrated that solasonine significantly inhibited BC cell proliferation and promoted cell apoptosis by inhibiting the MAPK and PI3K/AKT signaling pathways. NRP1 may be a potential effector of solasonine in mediating anti-BC responses.

## 2. Materials and Methods

### 2.1. The Molecular Structure of Solasonine

The molecular structure of solasonine was generated using the JSME platform (https://jsme-editor.github.io/) [25] by importing the SMILES code obtained from the PubChem database (https://pubchem.ncbi.nlm.nih.gov/). The molecular structure was converted to a 3D image using the Yinfo Cloud platform (http://cloud.yinfotek.com/).

### 2.2. Evaluation of Pharmacokinetics Properties for Solasonine

The Traditional Chinese Medicines for Systems Pharmacology (TCMSP) database and analysis platform server (https://tcmsp-e.com/) is a systems-level pharmacology database, which can calculate pharmacokinetics properties for naturally occurring compounds based on ADME (absorption, distribution, metabolism, and excretion) principle. The TCMSP server was used to evaluate the pharmacokinetics characteristics of solasonine on ADME-related properties, such as human oral bioavailability (OB), drug-likeness (DL), Caco-2 permeability (Caco-2), blood-brain barrier (BBB) permeability, and Lipinski's rule of five (MW, AlogP, TPSA, Hdon, Hacc) [26].

### 2.3. Potential Targets Prediction for Solasonine

Potential targets of solasonine were predicted using an integrative application of PharmMapper (http://www.lilab-ecust.cn/pharmmapper/; updated on January 01, 2016) [27, 28], TargetNet (http://targetnet.scbdd.com/, updated on February 25, 2014) [29], and similarity ensemble approach (SEA, http://sea.bkslab.org/, updated on March 26, 2019) servers [30]. These databases adopted multiple prediction algorithms to generate compound target genes. All genes were converted to the HUGO Gene Nomenclature Committee (HGNC, https://www.genenames.org/) symbols to avoid confusion across platforms.

### 2.4. Bioinformatics Analysis by Ingenuity Pathway Analysis (IPA)

The potential target genes of solasonine were analyzed by IPA, which can predict downstream effects and identify new targets or candidate biomarkers and can obtain data analysis and interpretation to understand the experimental results within the context of biological systems [24]. In this study, analyses in five modules were performed using IPA, including canonical pathway analysis, disease and function analysis, upstream analysis, causal regulatory network analysis, and toxicity analysis.

### 2.5. Identification of the Targets of BC

The genes associated with BC were collected from five sources, including the databases of GeneCards (https://www.genecards.org/; version 5.5) [31], Online Mendelian Inheritance in Man (OMIM) (http://www.omim.org/; updated on September 03, 2021) [32], Therapeutic Target Database (TTD, http://db.idrblab.net/ttd/; updated on June 01, 2020) [33], Pharmacogenomics Knowledge Base (PharmGKB, https://www.pharmgkb.org/) [34], and DrugBank (https://www.drugbank.com/; version 5.1.8) [35]. We searched these databases with “bladder cancer,” “bladder carcinoma,” and “bladder tumor” as the keywords.

### 2.6. GO and KEGG Enrichment Analyses

As described in our previous research [24], GO analysis is a commonly used method to annotate genes and gene products for their molecular functions and associated biological pathways and cellular components [36]; KEGG is a useful resource for the systematic analysis of gene functions and related high-level genome functional information [37, 38]. In this study, the DOSE [39] and clusterProfiler [40] packages of the statistical software R (version 4.1.0) were used for mining information related to the biological effects of differential expressed genes and implementing pathway enrichment analysis. Subsequently, the enrichplot and ggplot2 packages were used for high-quality graph generation.

### 2.7. Network Analysis

The gene-gene interaction network was generated using GeneMANIA prediction server (http://www.genemania.org/) [41], which can also reveal the biological functions of the gene set. After KEGG pathway and GO terms analyses, the representative enriched KEGG and GO terms and their related gene symbols were imported into Cytoscape software (version 3.8.2) [42] to construct the gene-pathway/GO term network.

### 2.8. Molecular Docking Analysis

Molecular docking analysis was conducted to evaluate the binding affinity of solasonine towards NRP1 protein. The 3D structure of solasonine was optimized with energy minimization in the MMFF94 force field. The crystal structure of human NRP1 (PDB ID: 1KEX, NRP1-b1 domain) was directly retrieved from the RCSB protein data bank (PDB) database (https://www.rcsb.org/). The protein structures were then prepared by adding hydrogen atoms and merging nonpolar hydrogen atoms using AutoDock Tools (ADT, version 1.5.6) [43] and saved as PDBQT files. The docking pocket of protein was defined using the grid box module. AutoDock Vina (version 1.1.2) [44] was utilized to perform molecular docking, and after evaluating the binding energy (affinity), the conformation with the best affinity was visualized in PyMOL (version 2.5.2) software. The 2D diagram of the protein-ligand was visualized using the Discovery Studio 2019 server.

### 2.9. Molecular Dynamics Simulation and Binding Free Energy Calculation

The protein-ligand docked complex structure of NRP1-solasonine was prepared for molecular dynamics simulation (MDS) using Visual Molecular Dynamics (VMD, version 1.9.4) software [45]. The TIP3P water model was adopted to solvate the complexes followed by adding ions to neutralize. Periodic boundary conditions were employed. Energy minimization was performed with a tolerance of 1000 kJ/mol/nm, and the system was equilibrated by NVT (constant volume) of 1 atm and NPT (constant pressure) of 300K ensemble for 100 picoseconds (ps). Electrostatic interaction calculations and constraint on bond length were performed using the particle mesh Ewald (PME) method and the linear constraint solver (LINCS) algorithm, respectively. MDS was carried out applying the OPLS force field for 200 nanoseconds (ns) by GROMACS (version 2021.3) [46], with trajectories generated every 2 femtoseconds (fs) and collected every 2 ps. Several parameters including root-mean-square deviation (RMSD), root-mean-square fluctuation (RMSF), radius of gyration (Rg), and number of hydrogen bonds were measured to assess the conformational and performance stability of the protein-ligand complex in a dynamic environment. Finally, the binding free energy of the docked complex and the energy contribution of each residue to binding were investigated using the molecular mechanics Poisson–Boltzmann surface area (MM-PBSA) method [47] implemented in the AmberTools21 package.

### 2.10. Cell Culture and Reagents

The human bladder cancer cell lines T24 and 5637 were purchased from the Cell Resource Center of the Shanghai Institutes for Biological Sciences, Chinese Academy of Sciences (Shanghai, China). Solasonine was purchased from Chengdu Must Bio-Technology Company (Chengdu, Sichuan, China). The cell lines were cultured in RPMI-1640 medium with 100 U/mL penicillin, 100 *μ*g/mL streptomycin, and 10% fetal bovine serum at 5% CO_2_ in a 37°C humidified culture environment, which was as previously described [24, 48]. Short-tandem repeat profiling was used to authenticate the cell lines less than 6 months before this project was initiated, and the cells were not in culture for more than 2 months.

### 2.11. Cell Viability Assay

T24 and 5637 cells were seeded in 96-well cell culture plates at an initial density of 0.2 × 10^4^ cells/well in quintuplicate and treated with increasing concentrations of solasonine or vehicle (DMSO) for 48 h. The cell counting kit-8 (CCK8) assay was used to measure cell viability. Lastly, optical density (OD) was measured at 450 nm to detect viable cells.

### 2.12. CCK8 Assay

T24 and 5637 cells were seeded in 96-well cell culture plates at an initial density of 0.2 × 10^4^ cells/well in triplicate. After treating with solasonine or vehicle (DMSO) for 24 h, the medium was discarded and the cells were cultured in a resuspended RPMI-1640 medium at a volume of 100 *μ*L/well. At different time points, the cells were incubated with 10 *μ*L CCK8 reagent (CK04, Dujindo Chemical, Japan) at 37°C for 4 h. Finally, the OD of each well at 450 nm wavelength was measured to evaluate cell proliferation.

### 2.13. Flow Cytometric Apoptosis Test

As performed in our previous study [24], T24 and 5637 cells were digested with 0.25% trypsin, washed with PBS, and centrifuged at 1300 rpm for 3 min. The supernatant was aspirated followed by adding 200 *μ*L of 1× binding buffer to each tube. Next, 5 *μ*L of Annexin-V-APC (eBioscience, 88–8007) and 5 *μ*L of propidium iodide (PI) (Sigma) were added to each tube. The tubes were then incubated for 15 min at room temperature and shielded from light. Then, a BD FACSCalibur flow cytometer (BD Biosciences) was used to assess the cell apoptosis.

### 2.14. Flow Cytometry Cell Cycle Analysis

As described previously [24], cells were digested with 0.25% trypsin, washed with PBS, and centrifuged at 1300 rpm for 3 min. The cell pellet was washed twice with PBS and resuspended in 1 mL of PBS. Next, the tubes were oscillated on a low-speed oscillator, and 3 mL of 70% ice-cold ethanol was added to fix the cells for 2 h at 4°C. After discarding ethanol, the pellet was resuspended in 2 mL PBS and centrifuged at 3000 rpm for 3 min followed by removing the supernatant. Subsequently, 1 mL DNA staining solution (CCS102, MultiSciences, China) and 10 *µ*L permeabilization solution (CCS102, MultiSciences) were treated and the tubes were incubated at room temperature for 30 min in the dark. Finally, cell cycle detection was performed using a NovoCyte 2060R flow cytometer (ACEA Biosciences, USA) and analyzed by FlowJo software (Tree Star, Inc, USA).

### 2.15. Protein Extraction and Western Blot

Total protein was extracted from T24 and 5637 cells, and Western blot were performed as described previously [24, 49]. Briefly, equal protein amounts (20 *μ*g) were loaded into each lane. The primary antibodies for Western blot are listed below: NRP1 (1 : 500; ab25998, Abcam, USA), extracellular signal-regulated protein kinases 1 and 2 (ERK1/2) (1 : 3000; #9107, Cell Signaling Technology Co., Ltd. (CST), China), phosphorylated ERK (p-ERK) (1 : 500; #4376, CST), p38 mitogen-activated protein kinase (P38) (1 : 3000; #8690, CST), phosphorylated P38 (p-P38) (1 : 500; #4631, CST), mitogen-activated protein kinase kinase 1 (MEK1) (1 : 3000; ab32091, Abcam), phosphorylated MEK 1 and 2 (p-MEK1/2) (1 : 1000; #9154, CST), PI 3 kinase (PI3K) (1 : 1000; AF1729, Beyotime, China), phosphorylated PI3K (p-PI3K) (AF3242, Affinity), threonine-protein kinase (AKT) (1 : 1000; AA326, Beyotime), phosphorylated AKT (p-AKT) (1 : 200; ab81823, Abcam), EGFR (1 : 500; GB11084, Servicebio, China), vascular endothelial growth factor A (VEGFA) (1 : 500; GB11034B, Servicebio), glyceraldehyde-3-phosphate dehydrogenase (GAPDH) (1 : 1000; MB001H, BioWorld, USA), and *β*-actin (1 : 10000; TDY051, Beijing TDY Biotech Co., Ltd., China). After washing with TBST, the membranes were further incubated for 2 h with a secondary anti-mouse (1 : 10000; AS1106, Aspen Biotechnology Co., Ltd. (ASPEN), China) or anti-rabbit (1 : 10000; AS1107, ASPEN) antibody, as appropriate. The presentation of target protein bands was enhanced using chemiluminescence (Millipore) and then quantified by densitometry (BioRad image analysis system) and normalized to *β*-actin levels.

### 2.16. Statistical Analysis

Statistical analyses were conducted using SAS 9.43 statistical software (SAS Institute Inc., Cary, NC, USA). One-way ANOVA was carried out to perform significance tests for multiple groups. Significant differences for two groups were evaluated using Student's t-test. A *P* < 0.05 was considered statistically significant.

## 3. Results

### 3.1. Pharmacokinetics Properties and Potential Targets of Solasonine

**Figure 1:**
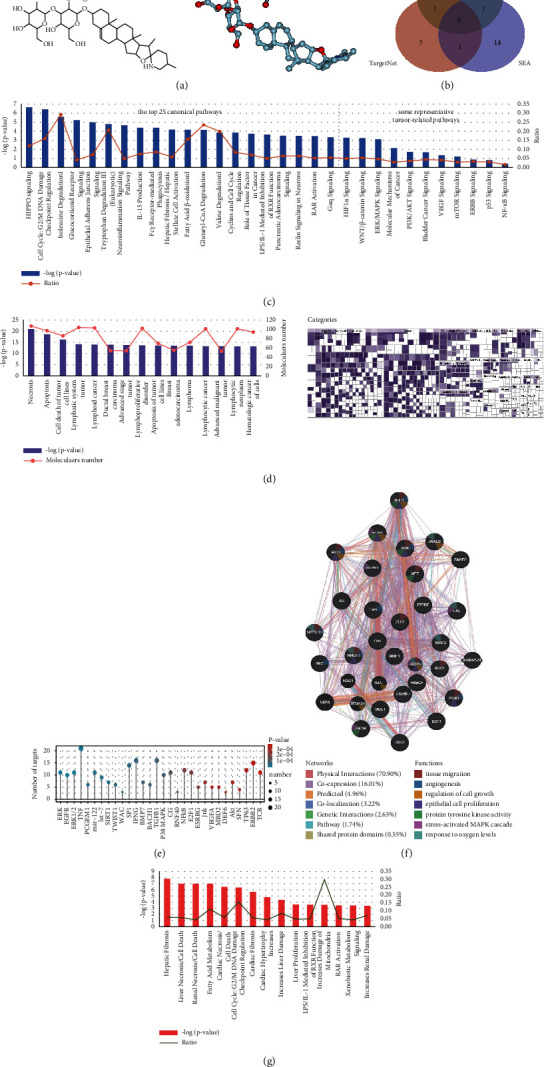
Bioinformatics analysis of potential targets of solasonine using IPA. (a) 2D and 3D molecular structures of solasonine. (b) Venn graph showing overlapping and specific genes predicted from PharmMapper, TargetNet, and SEA databases. (c) Canonical pathway analysis of solasonine by IPA. The top 25 enriched canonical pathways and some representative tumor-related pathways, sorted by −log(*P*-value) in decreasing order, are shown. (d) Disease and functional analysis using IPA for solasonine. The bar graph shows the top 15 annotations, and the heatmap shows the enrichment of targeting genes enriched in disease and function categories. (e) Upstream analysis using IPA for solasonine, showing the upstream regulatory factors for potential target genes of solasonine. The top 30 upstream regulatory factors in decreasing order of *P*-value were shown. (f) Causal regulatory network associated with NRP1. NRP1 was predicted as a master regulator involved in the regulation of 7 upstream regulators, including ERK (group), EGFR, AKT, KDR, MET, SMAD2, SMAD3. (g) Toxicity analysis for solasonine shows the top 15 potential toxicities.

The molecular structure of solasonine is shown in Figure 1(a), and its ADME-related pharmacokinetic properties are presented in Table 1. Notably, the OB of solasonine was 25.94%. Subsequently, the potential targets of solasonine were predicted using three different approaches described in Methods.  Figure 1(b) shows 286 candidate targets identified from PharmMapper (Norm fit > 0.3), five targets from the TargetNet server, and 16 human target proteins from the SEA database. Three genes, AR, G6PD, and CYP17A1, shared by the two databases, and a total of 304 unique genes in the merged union were identified as potential targets of solasonine for further analysis. Information on potential target genes is provided in Table S1.

### 3.2. Bioinformatics Analysis by IPA

The canonical pathway analysis generated by IPA revealed that the potential target genes of solasonine were significantly enriched in 494 canonical pathways, including Hippo signaling (−logP = 6.66), cell cycle (−logP = 6.44), HIF-1 signaling (−logP = 3.28), PI3K/AKT signaling (−logP = 1.72), and ERK/MAPK signaling (−logP = 3.13) (Figure 1(c) and Table S2). The disease and functional analysis showed 500 remarkably enriched annotations of diseases or functions that were most related to cell death and survival, cell cycle, and various cancer processes (Figure 1(d) and Table S3). Upstream regulatory and causal regulatory network analyses were performed to predict the potential upstream regulators of solasonine-targeting genes (Table S4) and the regulators of these predicted upstream regulators (Table S5). The top 30 predicted upstream regulators are listed in Figure 1(e). The ERK cascade was the most significant upstream regulator targeting 11 genes, followed by 9 EGFR targeting genes. Notably, both the upstream regulators ERK and EGFR and the other five upstream regulators AKT, KDR, MET, SMAD2, and SMAD3 were involved in the causal regulatory network associated with NRP1, mainly functioning in tissue migration, angiogenesis, growth, proliferation, hypoxia, MAPK signaling, and protein tyrosine kinase (PTK) activity (Figure 1(f)). In addition, the potential hepatotoxicity, nephrotoxicity, and cardiotoxicity of solasonine were predicted by toxicity analysis using IPA (Figure 1(g) and Table S6).

### 3.3. Target Identification and Analysis for BC

**Figure 2:**
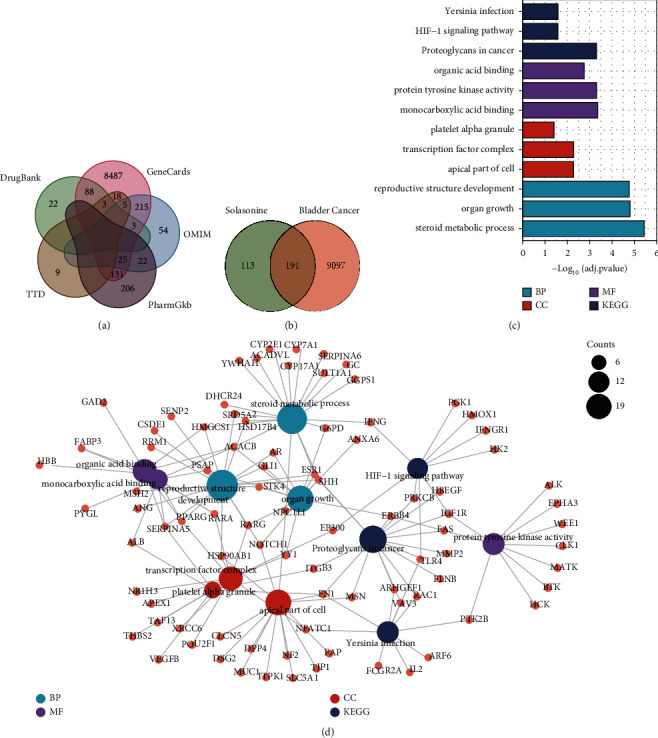
Target identification and functional enrichment analysis of potential target genes of solanine in bladder cancer. (a) Venn graph showing the overlapping and specific genes associated with BC in GeneCards, OMIM, TTD, PharmGKB, and DrugBank databases. (b) Venn graph showing the overlapping and specific genes by integration of the potential targets regulated by solasonine and genes associated with BC. (c) Bar graph showing the top three significantly enriched KEGG pathways and GO terms (BP, CC, and MF), sorted by adjusted *P*-values in increasing order. (d) Gene-pathway/GO terms network showing interactions among gene targets for solasonine and the top significant enriched pathways/GO terms.

By searching the databases of GeneCards, OMIM, TTD, PharmGKB, and DrugBank, we obtained a total of 9288 genes related to the occurrence and development of BC after eliminating the duplicated ones (Figure 2(a)). Integration of the potential targets regulated by solasonine, and the genes associated with BC revealed that 191 genes overlapped (Figure 2(b) and Table S7) and could be candidate therapeutic targets of solasonine in BC.

### 3.4. GO and KEGG Pathway Analyses of Gene Targets for Solasonine in BC

KEGG pathway enrichment analysis showed that these potential therapeutic targets of solasonine for BC were significantly enriched in three pathways (adjusted *P*-value ≤ 0.05), including proteoglycans in cancer, HIF-1 signaling pathway, and Yersinia infection. In addition, the results of GO term analysis indicated that a total of 692, 16, and 48 terms (adjusted *P*-value ≤ 0.05) were remarkably enriched in biological process (BP), cellular component (CC), and molecular function (MF), respectively (Table S8). The top three KEGG pathways, as well as BP, CC, and MF terms, sorted by adjusting *P*-values in increasing order, are presented in a bar graph (Figure 2(c)), showing a significant enrichment of organ growth (BP), transcription factor complex (CC), and protein tyrosine kinase activity (MF). The gene-pathway/GO terms network showed the interactions among the gene targets for solasonine and the top significant enriched pathways/GO terms (Figure 2(d)).

### 3.5. Solasonine Affects BC Cell Viability and Inhibits Cell Proliferation

**Figure 3:**
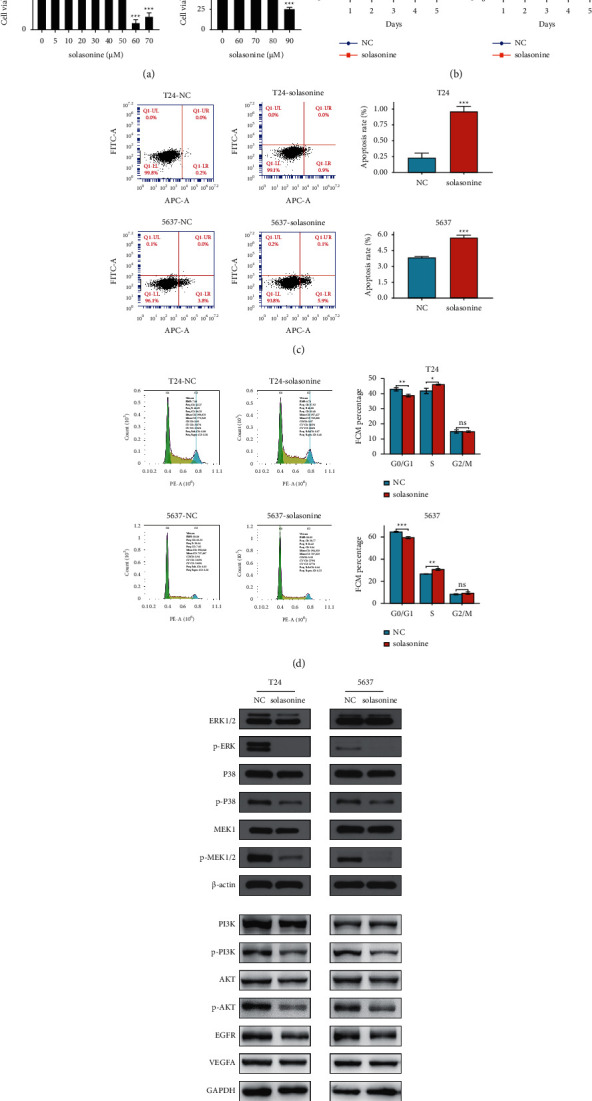
Effects of solasonine on cell proliferation, apoptosis, cell cycle arrest, and the expression of genes in tumor-related pathways in BC cells. (a) T24 and 5637 cells were treated with various concentrations of solasonine for 48 h, and the cell viability was evaluated by CCK8 assay. The doses of 50 and 80 *μ*M solasonine were selected for T24 and 5637 cells in subsequent experiments, respectively. (b) CCK8 assay revealed that solasonine significantly reduced the proliferation of BC cells in a time-dependent manner. (c) Apoptosis assay and quantitation of apoptotic cells showed that solasonine significantly promoted apoptosis in BC cells. (d) Flow cytometric analysis showed that solasonine affected cell cycle progression. (e) Western blot showed changes of molecules in the MAPK and PI3K/AKT signaling pathways, EGFR and VEGFA protein expression in T24 and 5637 BC cells after solasonine treatment. Three independent experiments were conducted for each assay, and data are presented as the mean ± standard error of the mean. ^*∗*^*P* < 0.05, ^*∗∗*^*P* < 0.01, and ^*∗∗∗*^*P* < 0.001 vs. the control group.

The CCK8 assay was performed to determine the effect of solasonine on the viability and growth of BC cells. As illustrated in Figure 3(a), no obvious inhibitory effect on cell viability was observed in T24 cells before treatment with solasonine at 60 *μ*M for 48 h. The 5637 cells showed improved tolerance until 90 *μ*M solasonine was used. Thus, in subsequent experiments, 50 and 80 *μ*M solasonine doses were selected for T24 and 5637 cells, respectively. Proliferation curves showed that, compared with the control group, cell proliferation was markedly inhibited by solasonine in a time-dependent manner (Figure 3(b)). These results demonstrated that solasonine may function as a tumor suppressor in BC cells.

### 3.6. Solasonine Promotes BC Cell Apoptosis and Induces Cell Cycle Arrest

To investigate the possible mechanism of solasonine on suppressing cell proliferation, we evaluated cell apoptosis and cell cycle on BC cells. Compared with the control group, the proportion of apoptotic cells dramatically increased after solasonine treatment (Figure 3(c)). Flow cytometry analysis of the cell cycle showed that solasonine induced a significant decrease in the percentage of cells in the G0/G1 phase and an accumulation of cells arrested in the S phase, but no obvious alteration in the G2/M phase (Figure 3(d)). The results revealed that solasonine inhibited BC cell proliferation by promoting apoptosis and inhibiting the G0/G1 and S phase transitions.

### 3.7. Solasonine Inhibits MAPK and PI3K/AKT Pathways

We performed Western blot to confirm the prediction of canonical pathway analysis by IPA. It was found that the MAPK and PI3K/AKT signaling pathways were involved in the antitumor activity of solasonine. As shown in Figure 3(e), solasonine obviously decreased the phosphorylation of ERK, P38, and MEK1/2 in T24 and 5637 cells, suggesting a close relationship between the inhibition of ERK/MAPK, P38/MAPK, and PI3K/AKT signaling pathways with solasonine treatment. In addition, the expression of EGFR and VEGF, two predicted upstream regulators for solasonine identified by IPA analysis, did not differ significantly after solasonine treatment.

### 3.8. Analysis of Molecular Docking and MDS

**Figure 4:**
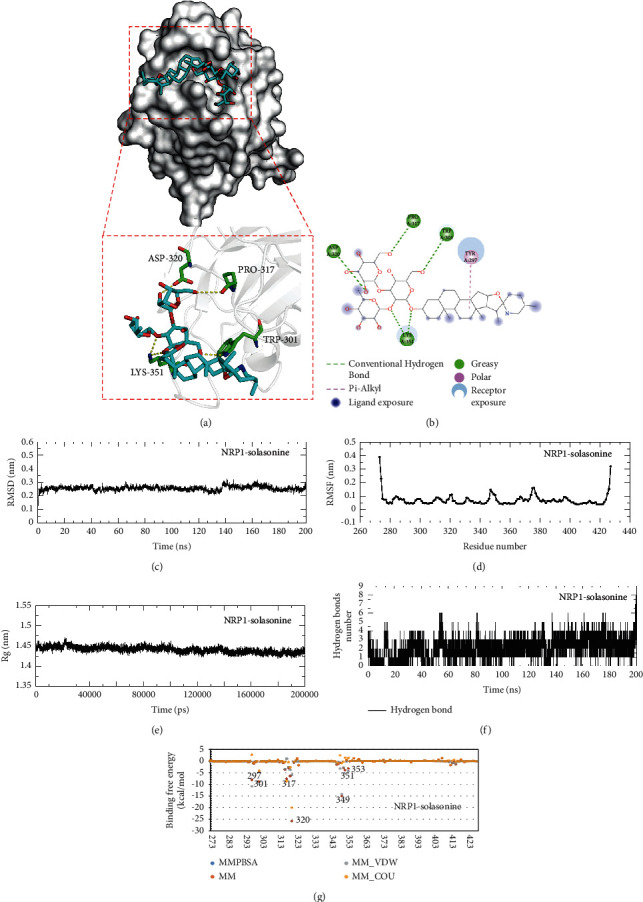
Molecular docking, MDS, and binding free energy calculation for NRP1 docking with solasonine. Molecular docking modes for NRP1 docking with solasonine in (a) 3D and (b) 2D images. Molecular surface representation of NRP1 (white) was shown as the docking pose and interaction of solasonine (blue stick model) with the primary pocket. (c) Plot of root-mean-square deviation (RMSD) values during 200 ns MDS of NRP1 docking with solasonine. (d) Plot of root-mean-square fluctuation (RMSF) values during 200 ns MDS. (e) Plot of radius of gyration (Rg) values during 200 ns MDS. (f) Plot of hydrogen bond number during 200 ns MDS of NRP1 docking with solasonine. (g) Plot of binding free energy simulation of NRP1 docking with solasonine.

Molecular docking analysis suggested that solasonine strongly interacts with the catalytic pocket of NRP1. Binding affinity was measured in kcal/mol, with a smaller affinity indicating stronger binding. An affinity of <−7.00 kcal/mol represents a satisfactory binding strength between the ligand and receptor. It was found solasonine could form hydrogen bonds with several residues and hydrophobic effect with TYR297 bound to NRP1 (affinity = −8.1 kcal/mol), which could stabilize the NRP1 protein and mediate the anchorage of solasonine in the binding site of NRP1 to form a stable complex (Figures 4(a)–4(b)). Further MDS analysis confirmed the docking stability of the NRP1-solasonine complex. The structural variations of NRP1-solasonine complex were examined by RMSD values from 0 to 200 ns, showing a steady increase at the beginning and a stable state throughout the simulation with an average RMSD value of 0.26 nm (Figure 4(c)). The fluctuation of the binding site residues in the NRP1-solasonine docking complex was reflected by RMSF values, which showed a stable docking pose with an average RMSF value of 0.07 nm (Figure 4(d)). Moreover, the Rg value of the complex was in the range of 1.42 and 1.47 nm in MDS (Figure 4(e)), and the hydrogen bonds were varied between 0 and 8 (Figure 4(f)).

### 3.9. Binding Free Energy Calculation

The binding free energy for the NRP1-solasonine complex was calculated to be −97.744 kcal/mol (Figure 4(g) and Table S9). Consistent with the molecular docking results, it is also evident from the binding free energies that TYR297, TRP301, PRO317, ASP320, and LYS351 residues mainly contributed to mediating anchorage with solasonine to form a stable NRP1-solasonine docking. The binding free energy was dominated by van der Waals and Coulombic electrostatic interaction energies. Solasonine binds most tightly with the ASP320 residue of NRP1 with a binding free energy of −25.80 kcal/mol mainly by Coulombic interaction energy, and the next most tightly bound residue was THR349, with a binding free energy of −15.04 kcal/mol mainly by van der Waals interaction energy, both of which were the main binding favorable for stabilizing the NRP1-solasonine complex.

### 3.10. Solasonine Inhibits NRP1 Protein Expression

**Figure 5:**
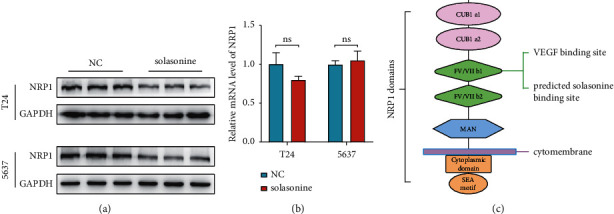
Effect of solasonine on NRP1 expression. (a) Solasonine inhibited NRP1 protein expression level. (b) Solasonine did not affect NRP1 mRNA expression. (c) The structure of NRP1 domains and the extracellular b1 domain was predicted to bind with solasonine.

Solasonine treatment resulted in a significant inhibitory effect on NRP1 protein expression (Figure 5(a)), but no significant difference was detected in NRP1 mRNA expression levels (Figure 5(b)). The NRP1 structure was shown, and the extracellular b1 domain was predicted to bind to solasonine (Figure 5(c)).

### 3.11. Solasonine-NRP1 Pathway Constructed in the Present Study

**Figure 6:**
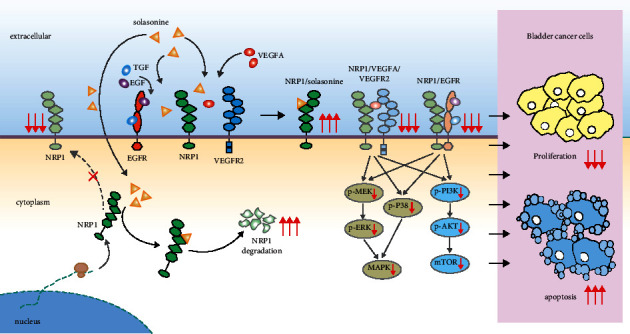
Solasonine-NRP1 pathway constructed in this study. The hypothetical solasonine-NRP1 interactions with subsequent inhibition of downstream pathways.

We further constructed hypothetical solasonine-NRP1 interactions, showing that extracellular solasonine binds to NRP1 at the cell membrane and thereby prevents the binding of VEGFA/VEGFR2 and EGFR to NRP1, resulting in the inhibition of the subsequent activation of downstream pathways, including ERK/MAPK, P38/MAPK, and PI3K/AKT pathways; in addition, intracellular solasonine bound to NRP1 to block the membrane localization of NRP1 and then downregulated NRP1 protein expression by facilitating its degradation (Figure 6). We hypothesized that solasonine might dock NRP1 to induce apoptosis and inhibit proliferation of BC cells.

## 4. Discussion

As single-pass transmembrane, non-tyrosine kinase surface glycoproteins, neuropilins (NRPs), are unique to vertebrates and highly conserved across species [50]. NRPs include two isoforms, NRP1 and NRP2. NRP1 was discovered in 1987 and was originally identified as a receptor in neuronal and endothelial cells and is essential for regulating neural and vascular development [22]. In recent years, high expression of NRP1 was found in other types of cells, such as immune cells, osteoblasts, adipocytes, hepatic stellate cells, and glomerular stromal cells. Increasing evidence on the involvement of NRP1 in cancer and immune processes has triggered research interests worldwide [22, 50–52]. NRP1 is considered a potential therapeutic target in the novel coronavirus disease 2019 [53, 54]. In addition, a growing body of evidence on NRP1-mediated immune modulation suggests that NRP1 is a novel immune checkpoint and immunotherapeutic target that may provide a durable anticancer immunity and help maintain long-term remission in cancer patients, either alone or in combination with current immunotherapeutic strategies [21, 22]. Therefore, the translation of NRP1 into therapeutic interventions is promising.

We previously showed that NRP1 was upregulated in BC, and NRP1 silencing could induce cell apoptosis and suppress proliferation, invasion, metastasis, and angiogenesis in BC cells. We also found that inactivation of the MAPK signaling pathway was involved in mediating the anti-BC effects of NRP1 [24]. In this study, we identified potential genes targeted by solasonine, followed by functional enrichment analysis. According to IPA and KEGG pathway analyses, the majority of these enrichment functions are related to tumorigenesis and progression, including the HIF-1 signaling pathway, ERK/MAPK pathway, P38/MAPK pathway, PI3K/AKT pathway, and PTK activity. The Western blot experiment further demonstrated that the activation of ERK/MAPK, P38/MAPK, and PI3K/AKT pathways could be suppressed after solasonine treatment in BC cells. It has been reported that the NRP1 promoter can directly bind to HIF-1*α* [55], which is a critical tumor driver involved in PI3K/AKT regulation and can facilitate the upregulation of both HIF-1*α* and PI3K/AKT through a positive feedback loop [56]. Overexpression of HIF-1 could induce upregulation of multiple growth factors, such as VEGF, PDGF, and EGF, that could in turn activate the PI3K/AKT pathway via receptor tyrosine kinases (RTKs) and subsequently result in the activation of the mammalian target of rapamycin (mTOR) complex to promote HIF-1 upregulation. It is generally accepted that ERK/MAPK is a pro-proliferation and antiapoptosis factor [57, 58], and P38/MAPK is a proliferation promoter in tumors [59]. This observation is consistent with our experimental findings in BC cells treated with solasonine.

According to the causal regulatory network analysis by IPA, NRP1 was identified as a master upstream regulator involved in solasonine treatment. We then detected the protein and mRNA expression levels of NRP1 in BC cells treated with solasonine. The results showed that treatment with solasonine suppressed NRP1 protein expression but had no effect on NRP1 mRNA expression. Further molecular docking analysis indicated that solasonine could directly target the NRP1 protein with a reasonable docking conformation. The stability and reliability of the tight binding results were validated by the binding free energy of the docked complex calculations through molecular dynamics simulations. These findings suggest that solasonine is not responsible for the transcriptional regulation of NRP1 but may downregulate the stability of NRP1 protein by direct binding to alter the molecular conformation of NRP1. In general, as a membrane protein, NRP1 needs to be inserted into the cell membrane to exert biological functions. By binding to NRP1, solasonine might inhibit the membrane localization of NRP1 and provoke its cytoplasmic retention, leading to its degradation in cells. This mechanism could be among the contributors to NRP1 protein downregulation, which requires further experimental verification.

The NRP1 protein consists of a long N-terminal extracellular domain, followed by a transmembrane region and a short cytosolic tail of 43–44 amino acids [22]. Its extracellular domain contains five subdomains, namely, a1, a2, b1, b2, and c, each of which mediates the interaction of NRP1 with different molecules and cells [50]. The b1 domain is the main functional domain responsible for NRP1 binding to the novel coronavirus disease 2019 receptors [53, 54] and multiple growth factors and receptors, most notably VEGFA and VEGFR2 [60]. The NRP1-b1 domain is also a target for the design of many small-molecule agents. NRP1 binds to VEGFA and VEGFR2 simultaneously to form an NRP1/VEGFA/VEGFR2 complex, eliciting activation of downstream signal transduction pathways, including ERK/MAPK, P38/MAPK, and PI3K/AKT pathways [22, 50, 60, 61]. Upstream regulator analysis by IPA identified both VEGF and EGFR as upstream regulators of solasonine-targeted genes. Current evidence indicates that the extracellular NRP1 domain could also bind to EGFR, which is essential for activating the EGFR signaling cascade upon EGF or TGF-a stimulation [62]. Meanwhile, following NRP1 silencing, the ability of ligand-bound EGFR to cluster on the cytomembrane and AKT pathway activity was severely impaired [62]. In our study, the protein expression levels of both EGFR and VEGF were not remarkably affected by solasonine treatment. These findings imply that solasonine interferes with NRP1 binding, which only affects the transduction of EGFR and VEGF signaling cascades, but not their expression.

Based on these results, we inferred that solasonine binding with NRP1 on the cell membrane could prevent the formation of NRP1/VEGFA/VEGFR2 and NRP1/EGFR complexes, resulting in the inhibition of downstream pathways, including ERK/MAPK, P38/MAPK, and PI3K/AKT pathways; meanwhile, intracellular solasonine could inhibit the membrane localization of NRP1 and provoke its cytoplasmic retention, facilitating the degradation of NRP1 protein in the cytoplasm. The subsequent dual effects induced by the binding of solasonine to NRP1 extracellularly and intracellularly could be potential contributors to the solasonine-dependent antiproliferative and proapoptotic effects on BC cells.

There were some limitations in this study. First, although the target prediction for solasonine against BC was performed in several online databases, it lacked accuracy and specificity compared with mass spectrometry and sequencing experiments. Second, we did not construct the silenced and overexpressed NRP1 groups to observe the anticancer effect of solasonine, which would weaken the evidence that solasonine targeting NRP1 induces apoptosis and inhibits proliferation in BC cells. Finally, immunoprecipitation experiments should be performed to confirm the binding of solasonine to NRP1 and to validate the mechanism of solasonine activity against BC observed in this study. These issues can be addressed in subsequent experiments. Our findings suggest a potential novel mechanism by which solasonine inhibits the growth of BC cells and indicate an important role of NRP1 in the activity of solasonine. Considering the widespread application of *S. nigrum* in various disorders [12], exploring the biological mechanism underlying the activity of solasonine could help reduce the incidence of toxic side effects and advance the development of precision or personalized therapies at a low cost.

## 5. Conclusions

In this study, the mechanisms and candidate targets of solasonine in the treatment of BC were examined using bioinformatics analyses and verified using experimental models. Molecular docking predicted that solasonine could directly bind to NRP1. The inhibition of NRP1 protein expression was observed in BC cells following solasonine treatment. We suggested that solasonine could inhibit the MAPK and PI3K/AKT pathways by preventing the formation of NRP1/VEGFA/VEGFR2 and NRP1/EGFR complexes on the membrane surface, inducing NRP1 protein degradation by restricting its membrane localization. The dual effects induced by solasonine-NRP1 binding extracellularly and intracellularly could be potential contributors to the antitumor effect of solasonine.

## Figures and Tables

**Table 1:** Pharmacological and molecular properties of solasonine.

Name	MW	AlogP	Hdon	Hacc	OB (%)	Caco-2	BBB	DL	FSAF	TPSA	RBN
Solasonine	884.19	0.02	10	17	25.94	−2.32	−3.43	0.06	0.2	258.71	8

Abbreviations: Caco-2, Caco-2 permeability; OB, oral bioavailability; DL, drug-likeness; BBB, blood-brain barrier.

## Data Availability

The data that support the findings of this study are available from the corresponding author upon reasonable request.

## Supplementary Materials

Supplementary Materials Table S1. Potential target genes of solasonine. Table S2. Canonical pathway analysis by IPA for solasonine. Table S3. Disease and functional analysis by IPA for solasonine. Table S4. Upstream analysis by IPA for solasonine. Table S5. Causal regulatory network analysis by IPA for solasonine. Table S6. Toxicity analysis by IPA for solasonine. Table S7. Potential therapeutic targets of solasonine for BC. Table S8. KEGG pathway and GO analyses for genes of solasonine targeting BC. Table S9. Binding free energy of NRP1 docking with solasonine.
