# Supplementary material for: Solasonine Induces Apoptosis and Inhibits Proliferation of Bladder Cancer Cells by Suppressing NRP1 Expression
Source: J Oncol. 2022 Mar 2;2022:7261486. doi: 10.1155/2022/7261486 (PMC8906937; doi:10.1155/2022/7261486)
Supplement: Supplementary Materials — Table S1. Potential target genes of solasonine. Table S2. Canonical pathway analysis by IPA for solasonine. Table S3. Disease and functional analysis by IPA for solasonine. Table S4. Upstream analysis by IPA for solasonine. Table S5. Causal regulatory network analysis by IPA for solasonine. Table S6. Toxicity analysis by IPA for solasonine. Table S7. Potential therapeutic targets of solasonine for BC. Table S8. KEGG pathway and GO analyses for genes of solasonine targeting BC. Table S9. Binding free energy of NRP1 docking with solasonine. [file 7261486.f1.pdf]

Journal of Oncology

Solasonine induces apoptosis and inhibits proliferation of bladder cancer cells by suppressing NRP1 expression

Yang Dong, Lin Hao, Zhen-duo Shi, Kun Fang, Hui Yu, Guang-hui Zang, Tao Fan, Cong-hui Han

Supplementary Materials

Table S1: Potential target genes of solasonine ……………………..………………......... ………………….Page: 2~11

Table S2: Canonical pathway analysis by IPA for solasonine ……………………….......... …………………Page:12~30

Table S3: Disease and functional analysis by IPA for solasonine………………………..... …………………Page:31~92

Table S4: Upstream analysis by IPA for solasonine……………………….......................... ……………….Page: 93~111

Table S5: Causal regulatory network analysis by IPA for solasonine……………………… …...…………Page: 112~146

Table S6: Toxicity analysis by IPA for solasonine…………………….......................... …...…………Page: 147~150

Table S7: Potential therapeutic targets of solasonine for BC…………….......................... …...…………Page: 151~156

Table S8: KEGG pathway and GO analyses for genes of solasonine targeting BC………... …...…………Page:157~207

Table S9: Binding free energy of NRP1 docking with solasonine…………………………. …...…………Page: 208~212

Supplementary Table S1: Potential target genes of solasonine. There were 286 potential targets identified from PharmMapper (Norm fit > 0.3), 5 targets from TargetNet server, and 16 human target proteins from SEA database. After removal of three overlapped genes, a total of 304 unique target genes were obtained in the merged union.

**PharmMapper Database**				
Num	Database	Symbol	Norm Fit	Name
1	PharmMapper	CSDE1	0.830	Cold shock domain-containing protein E1
2	PharmMapper	RGS6	0.816	Regulator of G-protein signaling 6
3	PharmMapper	APPL1	0.797	DCC-interacting protein 13-alpha
4	PharmMapper	POT1	0.783	Protection of telomeres protein 1
5	PharmMapper	F2	0.783	Prothrombin
6	PharmMapper	RARG	0.778	Retinoic acid receptor gamma
7	PharmMapper	NF2	0.718	Merlin
8	PharmMapper	CUL1	0.715	Cullin-1
9	PharmMapper	HK2	0.675	Hexokinase-2
10	PharmMapper	EP300	0.668	Histone acetyltransferase p300
11	PharmMapper	USP19	0.666	Ubiquitin carboxyl-terminal hydrolase 19
12	PharmMapper	SH3YL1	0.663	SH3 domain-containing YSC84-like protein 1
13	PharmMapper	ACBD7	0.660	Acyl-CoA-binding domain-containing protein 7
14	PharmMapper	CAMK4	0.643	Calcium/calmodulin-dependent protein kinase type IV
15	PharmMapper	INADL	0.643	InaD-like protein
16	PharmMapper	RRM1	0.635	Ribonucleoside-diphosphate reductase large subunit
17	PharmMapper	XRCC6	0.632	ATP-dependent DNA helicase 2 subunit 1
18	PharmMapper	HSD17B4	0.631	Peroxisomal multifunctional enzyme type 2
19	PharmMapper	PIP4K2C	0.629	Phosphatidylinositol-5-phosphate 4-kinase type-2 gamma
20	PharmMapper	MMP2	0.625	72 kDa type IV collagenase
21	PharmMapper	IQUB	0.622	IQ and ubiquitin-like domain-containing protein
22	PharmMapper	HIBCH	0.612	3-hydroxyisobutyryl-CoA hydrolase, mitochondrial
23	PharmMapper	ARHGAP5	0.605	Rho GTPase-activating protein 5
24	PharmMapper	ACADVL	0.591	Very long-chain specific acyl-CoA dehydrogenase, mitochondrial
25	PharmMapper	PTPRE	0.585	Receptor-type tyrosine-protein phosphatase epsilon
26	PharmMapper	HBEGF	0.580	Proheparin-binding EGF-like growth factor

27	PharmMapper	HMGCS1	0.575	Hydroxymethylglutaryl-CoA synthase, cytoplasmic
28	PharmMapper	AR	0.573	Androgen receptor
29	PharmMapper	HMOX1	0.571	Heme oxygenase 1
30	PharmMapper	VAV3	0.569	Guanine nucleotide exchange factor VAV3
31	PharmMapper	DMC1	0.568	Meiotic recombination protein DMC1/LIM15 homolog
32	PharmMapper	ZEB2	0.560	Zinc finger E-box-binding homeobox 2
33	PharmMapper	YARS	0.556	Tyrosyl-tRNA synthetase, cytoplasmic
34	PharmMapper	ZBTB43	0.555	Zinc finger and BTB domain-containing protein 43
35	PharmMapper	NME3	0.555	Nucleoside diphosphate kinase 3
36	PharmMapper	GALM	0.553	Aldose 1-epimerase
37	PharmMapper	NR1I3	0.552	Nuclear receptor subfamily 1 group I member 3
38	PharmMapper	BTK	0.552	Tyrosine-protein kinase BTK
39	PharmMapper	MUC1	0.552	Mucin-1
40	PharmMapper	IMPDH1	0.550	Inosine-5-monophosphate dehydrogenase 1
41	PharmMapper	MARK1	0.548	Serine/threonine-protein kinase MARK1
42	PharmMapper	DOK2	0.547	Docking protein 2
43	PharmMapper	SUB1	0.543	Activated RNA polymerase II transcriptional coactivator p15
44	PharmMapper	ACAT2	0.543	Acetyl-CoA acetyltransferase, cytosolic
45	PharmMapper	VEGFB	0.542	Vascular endothelial growth factor B
46	PharmMapper	FABP3	0.541	Fatty acid-binding protein, heart
47	PharmMapper	KLK1	0.538	Kallikrein-1
48	PharmMapper	RND1	0.538	Rho-related GTP-binding protein Rho6
49	PharmMapper	TIMM9	0.536	Mitochondrial import inner membrane translocase subunit Tim9
50	PharmMapper	HBB	0.532	Hemoglobin subunit beta
51	PharmMapper	MAP3K3	0.531	Mitogen-activated protein kinase kinase kinase 3
52	PharmMapper	CDK5R1	0.530	Cyclin-dependent kinase 5 activator 1
53	PharmMapper	ITPKC	0.523	Inositol-trisphosphate 3-kinase C
54	PharmMapper	DFFA	0.520	DNA fragmentation factor subunit alpha
55	PharmMapper	PDK3	0.509	[Pyruvate dehydrogenase [lipoamide]] kinase isozyme 3, mitochondrial
56	PharmMapper	ACADSB	0.508	Short/branched chain specific acyl-CoA dehydrogenase, mitochondrial
57	PharmMapper	RBP5	0.506	Retinol-binding protein 5
58	PharmMapper	ZNF462	0.502	Zinc finger protein 462

59	PharmMapper	NMRK1	0.500	Nicotinamide riboside kinase 1
60	PharmMapper	ESR1	0.500	Estrogen receptor
61	PharmMapper	HSCB	0.496	Co-chaperone protein HscB, mitochondrial
62	PharmMapper	PUS10	0.495	Putative tRNA pseudouridine synthase Pus10
63	PharmMapper	RAC1	0.495	Ras-related C3 botulinum toxin substrate 1
64	PharmMapper	PTPRF	0.494	Receptor-type tyrosine-protein phosphatase F
65	PharmMapper	SH2B2	0.490	SH2B adapter protein 2
66	PharmMapper	HCK	0.490	Tyrosine-protein kinase HCK
67	PharmMapper	CIAO1	0.490	Probable cytosolic iron-sulfur protein assembly protein CIAO1
68	PharmMapper	FCGR2A	0.489	Low affinity immunoglobulin gamma Fc region receptor II-a
69	PharmMapper	EPHA3	0.489	Ephrin type-A receptor 3
70	PharmMapper	PTK2B	0.487	Protein tyrosine kinase 2 beta
71	PharmMapper	TRIM21	0.485	52 kDa Ro protein
72	PharmMapper	NDUFAB1	0.484	Acyl carrier protein, mitochondrial
73	PharmMapper	AMY1B	0.484	Alpha-amylase 1
74	PharmMapper	CRYM	0.484	Mu-crystallin homolog
75	PharmMapper	DTYMK	0.482	Thymidylate kinase
76	PharmMapper	PPP2R1A	0.482	Serine/threonine-protein phosphatase 2A 65 kDa regulatory subunit A alpha isoform
77	PharmMapper	MTAP	0.481	S-methyl-5-thioadenosine phosphorylase
78	PharmMapper	KMT5A	0.481	Histone-lysine N-methyltransferase SETD8
79	PharmMapper	LARS	0.481	Leucyl-tRNA synthetase, cytoplasmic
80	PharmMapper	IGF1R	0.479	Insulin-like growth factor 1 receptor
81	PharmMapper	FLNB	0.479	Filamin-B
82	PharmMapper	HDAC7	0.479	Histone deacetylase 7
83	PharmMapper	ALOX12	0.474	Arachidonate 12-lipoxygenase, 12S-type
84	PharmMapper	ZFP28	0.474	Zinc finger protein 28 homolog
85	PharmMapper	WWP1	0.474	NEDD4-like E3 ubiquitin-protein ligase WWP1
86	PharmMapper	HSP90AB1	0.472	Heat shock protein HSP 90-beta
87	PharmMapper	KIF11	0.472	Kinesin-like protein KIF11
88	PharmMapper	GC	0.471	Vitamin D-binding protein
89	PharmMapper	MATK	0.468	Megakaryocyte-associated tyrosine-protein kinase
90	PharmMapper	DCXR	0.464	L-xylulose reductase

91	PharmMapper	ACPP	0.463	Prostatic acid phosphatase
92	PharmMapper	GTF2F2	0.461	General transcription factor IIF subunit 2
93	PharmMapper	ITSN1	0.459	Intersectin-1
94	PharmMapper	F3	0.458	Tissue factor
95	PharmMapper	ACVR2B	0.458	Activin receptor type-2B
96	PharmMapper	SNX22	0.457	Sorting nexin-22
97	PharmMapper	CLK1	0.456	Dual specificity protein kinase CLK1
98	PharmMapper	SENP2	0.456	Sentrin-specific protease 2
99	PharmMapper	CSF2RB	0.456	Cytokine receptor common subunit beta
100	PharmMapper	MSN	0.455	Moesin
101	PharmMapper	MEMO1	0.454	Protein MEMO1
102	PharmMapper	KYNU	0.453	Kynureninase
103	PharmMapper	SF3B6	0.452	Pre-mRNA branch site protein p14
104	PharmMapper	GSTT2B	0.451	Glutathione S-transferase theta-2
105	PharmMapper	RAB26	0.451	Ras-related protein Rab-26
106	PharmMapper	MAPK6	0.450	Mitogen-activated protein kinase 6
107	PharmMapper	BMP1	0.450	Bone morphogenetic protein 1
108	PharmMapper	PAPSS1	0.449	Bifunctional 3-phosphoadenosine 5-phosphosulfate synthetase 1
109	PharmMapper	PYGL	0.446	Glycogen phosphorylase, liver form
110	PharmMapper	MSH2	0.446	DNA mismatch repair protein Msh2
111	PharmMapper	POU2F1	0.446	POU domain, class 2, transcription factor 1
112	PharmMapper	GTF2I	0.446	General transcription factor II-I
113	PharmMapper	SKP1	0.444	S-phase kinase-associated protein 1
114	PharmMapper	PC	0.444	Pyruvate carboxylase, mitochondrial
115	PharmMapper	ZCWPW1	0.443	Zinc finger CW-type PWWP domain protein 1
116	PharmMapper	DHPS	0.443	Deoxyhypusine synthase
117	PharmMapper	ACSM2A	0.443	Acyl-coenzyme A synthetase ACSM2A, mitochondrial
118	PharmMapper	ASAP1	0.443	Arf-GAP with SH3 domain, ANK repeat and PH domain-containing protein 1
119	PharmMapper	NCOA3	0.443	Nuclear receptor coactivator 3
120	PharmMapper	TTR	0.442	Transthyretin
121	PharmMapper	CSNK1G2	0.442	Casein kinase I isoform gamma-2
122	PharmMapper	PDK2	0.441	[Pyruvate dehydrogenase [lipoamide]] kinase isozyme 2, mitochondrial

123	PharmMapper	ADH1C	0.437	Alcohol dehydrogenase 1C
124	PharmMapper	ITPK1	0.437	Inositol-tetrakisphosphate 1-kinase
125	PharmMapper	BPI	0.436	Bactericidal permeability-increasing protein
126	PharmMapper	HAT1	0.436	Histone acetyltransferase type B catalytic subunit
127	PharmMapper	HPD	0.436	4-hydroxyphenylpyruvate dioxygenase
128	PharmMapper	PAH	0.435	Phenylalanine-4-hydroxylase
129	PharmMapper	B2M	0.434	Beta-2-microglobulin
130	PharmMapper	SULT1A1	0.433	Sulfotransferase 1A1
131	PharmMapper	NT5C3A	0.431	Cytosolic 5-nucleotidase 3
132	PharmMapper	PDF	0.431	Peptide deformylase, mitochondrial
133	PharmMapper	HDX	0.430	Highly divergent homeobox
134	PharmMapper	MYSM1	0.429	Histone H2A deubiquitinase MYSM1
135	PharmMapper	NUP214	0.427	Nuclear pore complex protein Nup214
136	PharmMapper	PANK1	0.426	Pantothenate kinase 1
137	PharmMapper	MAGEA4	0.426	Melanoma-associated antigen 4
138	PharmMapper	NSUN5	0.426	Putative methyltransferase NSUN5
139	PharmMapper	LRBA	0.425	Lipopolysaccharide-responsive and beige-like anchor protein
140	PharmMapper	SART3	0.425	Squamous cell carcinoma antigen recognized by T-cells 3
141	PharmMapper	TXNDC12	0.424	Thioredoxin domain-containing protein 12
142	PharmMapper	FABP7	0.424	Fatty acid-binding protein, brain
143	PharmMapper	BCR	0.423	Breakpoint cluster region protein
144	PharmMapper	CYP2R1	0.422	Vitamin D 25-hydroxylase
145	PharmMapper	PSAP	0.422	Proactivator polypeptide
146	PharmMapper	YWHAQ	0.422	14-3-3 protein theta
147	PharmMapper	SMG5	0.421	Protein SMG5
148	PharmMapper	SMG7	0.419	Protein SMG7
149	PharmMapper	WEE1	0.418	Wee1-like protein kinase
150	PharmMapper	AASDHPPT	0.418	L-aminoadipate-semialdehyde dehydrogenase-phosphopantetheinyl transferase
151	PharmMapper	TLR4	0.418	Toll-like receptor 4
152	PharmMapper	CYP7A1	0.416	Cytochrome P450 7A1
153	PharmMapper	IFNGR1	0.416	Interferon-gamma receptor alpha chain
154	PharmMapper	FAS	0.416	Tumor necrosis factor receptor superfamily member 6

155	PharmMapper	PEX5	0.413	Peroxisomal targeting signal 1 receptor
156	PharmMapper	PA2G4	0.412	Proliferation-associated protein 2G4
157	PharmMapper	EXOSC9	0.411	Exosome complex exonuclease RRP45
158	PharmMapper	RBBP6	0.410	Retinoblastoma-binding protein 6
159	PharmMapper	PHF24	0.410	Protein KIAA1045
160	PharmMapper	ACACB	0.410	Acetyl-CoA carboxylase 2
161	PharmMapper	ATXN3	0.410	Ataxin-3
162	PharmMapper	HNRNPA2B1	0.409	Heterogeneous nuclear ribonucleoproteins A2/B1
163	PharmMapper	ERBB4	0.406	Receptor tyrosine-protein kinase erbB-4
164	PharmMapper	ITGB3	0.405	Integrin beta-3
165	PharmMapper	SMAP1	0.404	Stromal membrane-associated protein 1
166	PharmMapper	ADRBK1	0.402	Beta-adrenergic receptor kinase 1
167	PharmMapper	MYBPC3	0.401	Myosin-binding protein C, cardiac-type
168	PharmMapper	GAD2	0.399	Glutamate decarboxylase 2
169	PharmMapper	PCYT2	0.398	Ethanolamine-phosphate cytidylyltransferase
170	PharmMapper	FAP	0.397	Seprase
171	PharmMapper	SEC23A	0.396	Protein transport protein Sec23A
172	PharmMapper	PARN	0.395	Poly(A)-specific ribonuclease PARN
173	PharmMapper	SMURF2	0.395	E3 ubiquitin-protein ligase SMURF2
174	PharmMapper	ADH7	0.395	Alcohol dehydrogenase class 4 mu/sigma chain
175	PharmMapper	RPA3	0.394	Replication protein A 14 kDa subunit
176	PharmMapper	ANXA6	0.393	Annexin A6
177	PharmMapper	MAGI2	0.392	Membrane-associated guanylate kinase, WW and PDZ domain-containing protein 2
178	PharmMapper	HLA-DRB1	0.391	HLA class II histocompatibility antigen, DRB1-1 beta chain
179	PharmMapper	CD1A	0.390	T-cell surface glycoprotein CD1a
180	PharmMapper	NFATC1	0.390	Nuclear factor of activated T-cells, cytoplasmic 1
181	PharmMapper	ALK	0.390	ALK tyrosine kinase receptor
182	PharmMapper	MDH1	0.389	Malate dehydrogenase, cytoplasmic
183	PharmMapper	CYP2E1	0.389	Cytochrome P450 2E1
184	PharmMapper	B3GAT2	0.388	Galactosylgalactosylxylosylprotein 3-beta-glucuronosyltransferase 2
185	PharmMapper	PPARG	0.387	Peroxisome proliferator-activated receptor gamma
186	PharmMapper	DGKA	0.387	Diacylglycerol kinase alpha

187	PharmMapper	ATP7B	0.386	Copper-transporting ATPase 2
188	PharmMapper	PGK1	0.386	Phosphoglycerate kinase 1
189	PharmMapper	MMP1	0.385	Interstitial collagenase
190	PharmMapper	COL4A3BP	0.384	Collagen type IV alpha-3-binding protein
191	PharmMapper	DAPK2	0.383	Death-associated protein kinase 2
192	PharmMapper	SHFM1	0.383	26S proteasome complex subunit DSS1
193	PharmMapper	S100A6	0.383	Protein S100-A6
194	PharmMapper	CSNK1G1	0.383	Casein kinase I isoform gamma-1
195	PharmMapper	NOTCH1	0.382	Neurogenic locus notch homolog protein 1
196	PharmMapper	SERPINA5	0.382	Plasma serine protease inhibitor
197	PharmMapper	GALNT10	0.382	Polypeptide N-acetylgalactosaminyltransferase 10
198	PharmMapper	NAE1	0.381	NEDD8-activating enzyme E1 regulatory subunit
199	PharmMapper	ZIC3	0.380	Zinc finger protein ZIC 3
200	PharmMapper	MECR	0.380	Trans-2-enoyl-CoA reductase, mitochondrial
201	PharmMapper	IDE	0.379	Insulin-degrading enzyme
202	PharmMapper	ODC1	0.379	Ornithine decarboxylase
203	PharmMapper	RARA	0.378	Retinoic acid receptor alpha
204	PharmMapper	PNMT	0.378	Phenylethanolamine N-methyltransferase
205	PharmMapper	HMBOX1	0.377	Homeobox-containing protein 1
206	PharmMapper	TJP1	0.377	Tight junction protein ZO-1
207	PharmMapper	AOC3	0.375	Membrane primary amine oxidase
208	PharmMapper	CSTF2	0.374	Cleavage stimulation factor 64 kDa subunit
209	PharmMapper	YY1	0.374	Transcriptional repressor protein YY1
210	PharmMapper	CANT1	0.370	Soluble calcium-activated nucleotidase 1
211	PharmMapper	CA8	0.370	Carbonic anhydrase-related protein
212	PharmMapper	IFNG	0.370	Interferon gamma
213	PharmMapper	ANG	0.365	Angiogenin
214	PharmMapper	ACY1	0.365	Aminoacylase-1
215	PharmMapper	SNX1	0.362	Sorting nexin-1
216	PharmMapper	C8A	0.360	Complement component C8 alpha chain
217	PharmMapper	CTCF	0.360	Transcriptional repressor CTCF
218	PharmMapper	TAF13	0.359	Transcription initiation factor TFIID subunit 13

219	PharmMapper	GSG2	0.359	Serine/threonine-protein kinase haspin
220	PharmMapper	GGPS1	0.358	Geranylgeranyl pyrophosphate synthetase
221	PharmMapper	BTRC	0.357	F-box/WD repeat-containing protein 1A
222	PharmMapper	TNFAIP8L2	0.356	Tumor necrosis factor, alpha-induced protein 8-like protein 2
223	PharmMapper	NBEA	0.355	Neurobeachin
224	PharmMapper	MYBPC1	0.355	Myosin-binding protein C, slow-type
225	PharmMapper	STK4	0.355	Serine/threonine-protein kinase 4
226	PharmMapper	ECI2	0.355	Peroxisomal 3,2-trans-enoyl-CoA isomerase
227	PharmMapper	EPS8L2	0.354	Epidermal growth factor receptor kinase substrate 8-like protein 2
228	PharmMapper	DPP4	0.351	Dipeptidyl peptidase 4
229	PharmMapper	ARHGEF1	0.349	Rho guanine nucleotide exchange factor 1
230	PharmMapper	APEX1	0.349	DNA-(apurinic or apyrimidinic site) lyase
231	PharmMapper	B3GAT3	0.348	Galactosylgalactosylxylosylprotein 3-beta-glucuronosyltransferase 3
232	PharmMapper	ARF6	0.347	ADP-ribosylation factor 6
233	PharmMapper	PGC	0.346	Gastricsin
234	PharmMapper	XIAP	0.346	Baculoviral IAP repeat-containing protein 4
235	PharmMapper	UEVLD	0.346	Ubiquitin-conjugating enzyme E2 variant 3
236	PharmMapper	ECHS1	0.346	Enoyl-CoA hydratase, mitochondrial
237	PharmMapper	ALB	0.346	Serum albumin
238	PharmMapper	PRKCB	0.345	Protein kinase C beta type
239	PharmMapper	FHIT	0.345	Bis(5-adenosyl)-triphosphatase
240	PharmMapper	GART	0.342	Trifunctional purine biosynthetic protein adenosine-3
241	PharmMapper	UTRN	0.342	Utrophin
242	PharmMapper	PAFAH1B2	0.342	Platelet-activating factor acetylhydrolase IB subunit beta
243	PharmMapper	STK16	0.338	Serine/threonine-protein kinase 16
244	PharmMapper	DFNB31	0.338	Whirlin
245	PharmMapper	IMPA2	0.337	Inositol monophosphatase 2
246	PharmMapper	C1R	0.335	Complement C1r subcomponent
247	PharmMapper	ITPA	0.334	Inosine triphosphate pyrophosphatase
248	PharmMapper	YWHAH	0.331	14-3-3 protein eta
249	PharmMapper	SFMBT2	0.331	Scm-like with four MBT domains protein 2
250	PharmMapper	OXSR1	0.329	Serine/threonine-protein kinase OSR1

251	PharmMapper	RTN4IP1	0.329	Reticulon-4-interacting protein 1, mitochondrial
252	PharmMapper	HMGB3	0.329	High mobility group protein B3
253	PharmMapper	OAT	0.328	Ornithine aminotransferase, mitochondrial
254	PharmMapper	ERI1	0.328	3-5 exoribonuclease 1
255	PharmMapper	SFN	0.328	14-3-3 protein sigma
256	PharmMapper	STARD13	0.328	StAR-related lipid transfer protein 13
257	PharmMapper	G6PD	0.327	Glucose-6-phosphate 1-dehydrogenase
258	PharmMapper	DSG2	0.327	Desmoglein-2
259	PharmMapper	THBS2	0.327	Thrombospondin-2
260	PharmMapper	DCTD	0.327	Deoxycytidylate deaminase
261	PharmMapper	PDK4	0.325	[Pyruvate dehydrogenase [lipoamide]] kinase isozyme 4, mitochondrial
262	PharmMapper	FN1	0.324	Fibronectin
263	PharmMapper	CHKA	0.324	Choline kinase alpha
264	PharmMapper	SMTN	0.323	Smoothelin
265	PharmMapper	VPS26A	0.322	Vacuolar protein sorting-associated protein 26A
266	PharmMapper	ZNF292	0.320	Zinc finger protein 292
267	PharmMapper	SEMA4D	0.320	Semaphorin-4D
268	PharmMapper	CLCN5	0.319	Chloride channel protein 5
269	PharmMapper	GRHPR	0.318	Glyoxylate reductase/hydroxypyruvate reductase
270	PharmMapper	NMT1	0.318	Glycylpeptide N-tetradecanoyltransferase 1
271	PharmMapper	GRK6	0.318	G protein-coupled receptor kinase 6
272	PharmMapper	WARS	0.318	Tryptophanyl-tRNA synthetase, cytoplasmic
273	PharmMapper	RUVBL1	0.318	RuvB-like 1
274	PharmMapper	SLK	0.317	STE20-like serine/threonine-protein kinase
275	PharmMapper	HSD17B10	0.316	3-hydroxyacyl-CoA dehydrogenase type-2
276	PharmMapper	NDC80	0.315	Kinetochore protein NDC80 homolog
277	PharmMapper	KIAA0430	0.315	Limkain-b1
278	PharmMapper	PARP3	0.315	Poly [ADP-ribose] polymerase 3
279	PharmMapper	RBM39	0.315	RNA-binding protein 39
280	PharmMapper	SYT13	0.313	Synaptotagmin-13
281	PharmMapper	ETF1	0.311	Eukaryotic peptide chain release factor subunit 1
282	PharmMapper	SPEF1	0.307	Sperm flagellar protein 1

283	PharmMapper	HADH	0.306	Hydroxyacyl-coenzyme A dehydrogenase, mitochondrial
284	PharmMapper	NCBP1	0.301	Nuclear cap-binding protein subunit 1
285	PharmMapper	PDHA1	0.301	Pyruvate dehydrogenase E1 component subunit alpha, somatic form, mitochondrial
286	PharmMapper	CD74	0.300	HLA class II histocompatibility antigen gamma chain
**TargetNet Database**				
Num	Databese	Symbol	Probility	Name
1	TargetNet	CYP17A1	1	Steroid 17-alpha-hydroxylase
2	TargetNet	HSP90	1	Heat shock protein HSP 90-alpha
3	TargetNet	AR	0.999	Androgen receptor
4	TargetNet	SLC5A1	0.995	Sodium/glucose cotransporter 1
5	TargetNet	HTR1E	0.933	5-hydroxytryptamine receptor 1E
**SEA Database**				
Num	Databese	Symbol	MaxTC	Name
1	SEA	DHCR24	0.31	Delta(24)-sterol reductase
2	SEA	AMY2A	0.29	Pancreatic alpha-amylase
3	SEA	NPC1	0.31	NPC intracellular cholesterol transporter 1
4	SEA	CYP17A1	0.32	Steroid 17-alpha-hydroxylase/17,20 lyase
5	SEA	IL2	0.69	Interleukin-2
6	SEA	NPC1L1	0.31	NPC1-like intracellular cholesterol transporter 1
7	SEA	EBP	0.41	3-beta-hydroxysteroid-Delta(8),Delta(7)-isomerase
8	SEA	ABCC4	0.32	Multidrug resistance-associated protein 4
9	SEA	G6PD	0.31	Glucose-6-phosphate 1-dehydrogenase
10	SEA	SERPINA6	0.29	Corticosteroid-binding globulin
11	SEA	GLI1	0.29	Zinc finger protein GLI1
12	SEA	POLA1	0.33	DNA polymerase alpha catalytic subunit
13	SEA	SRD5A2	0.32	3-oxo-5-alpha-steroid 4-dehydrogenase 2
14	SEA	NR1H3	0.32	Oxysterols receptor LXR-alpha
15	SEA	SHH	0.29	Sonic hedgehog protein
16	SEA	SHBG	0.29	Sex hormone-binding globulin

Abbreviations: MaxTC, Max Tanimoto Coefficient

Supplementary Table S2: Canonical pathway analysis by IPA for solasonine. The canonical pathway analysis by IPA shows the enrichment of the potential target genes of solasonine in the canonical signaling pathway. Through IPA canonical pathway analysis of the potential target genes of solasonine, 494 remarkably (p-value ≤0.05) enriched canonical pathways were obtained.

Ingenuity Canonical Pathways	-log(p-value)	Ratio	Molecules
HIPPO signaling	6.66	0.12	BTRC,CUL1,NF2,PATJ,PPP2R1A,SFN,SKP1,STK4,YWHAH,YWHAQ
Cell Cycle: G2/M DNA Damage Checkpoint Regulation	6.44	0.16	BTRC,CUL1,EP300,SFN,SKP1,WEE1,YWHAH,YWHAQ
Isoleucine Degradation I	5.60	0.29	ACADSB,ACAT2,ECHS1,HSD17B10,HSD17B4
Glucocorticoid Receptor Signaling	5.24	0.04	AR,B2M,CSF2RB,EP300,ESR1,GTF2F2,HLA-DRB1,HSP90AB1,IFNG,MMP1,MMP2,NCOA3,NDUFAB1,NFATC1,PC,PDK4,PPARG,RAC1,RARA,RARG,TAF13,YWHAH
Epithelial Adherens Junction Signaling	5.01	0.07	ACVR2B,IGF1R,MAGI2,NF2,NOTCH1,PPP2R1A,RAC1,SFN,STK4,YWHAH,YWHAQ
Tryptophan Degradation III (Eukaryotic)	4.80	0.21	ACAT2,HADH,HSD17B10,HSD17B4,KYNU
Neuroinflammation Signaling Pathway	4.66	0.05	ACVR2B,B2M,FAS,GAD2,HLA-DRB1,HMOX1,IDE,IFNG,IFNGR1,KLK1,MAPK6,NFATC1,RAC1,TLR4,XIAP
IL-15 Production	4.39	0.08	ALK,BTK,CLK1,EPHA3,ERBB4,HCK,IGF1R,MATK,PTK2B
Fcγ Receptor-mediated Phagocytosis in Macrophages and Monocytes	4.38	0.09	ARF6,FCGR2A,HCK,HMOX1,PRKCB,PTK2B,RAC1,VAV3
Hepatic Fibrosis / Hepatic Stellate Cell Activation	4.19	0.06	CERT1,CYP2E1,FAS,FN1,IFNG,IFNGR1,IGF1R,MMP1,MMP2,TLR4,VEGFB
Fatty Acid β-oxidation I	4.16	0.16	ECHS1,ECI2,HADH,HSD17B10,HSD17B4
Glutaryl-CoA Degradation	4.15	0.24	ACAT2,HADH,HSD17B10,HSD17B4
Valine Degradation I	3.85	0.20	ACADSB,ECHS1,HIBCH,HSD17B4
Cyclins and Cell Cycle Regulation	3.85	0.08	BTRC,CUL1,HDAC7,PA2G4,PPP2R1A,SKP1,WEE1
Role of Tissue Factor in Cancer	3.73	0.07	F2,F3,HBEGF,HCK,ITGB3,MMP1,PTK2B,RAC1
LPS/IL-1 Mediated Inhibition of RXR Function	3.63	0.05	CYP2E1,CYP2R1,CYP7A1,FABP3,FABP7,GSTT2/GSTT2B,HMGCS1,NR1I3,RARA,SULT1A1,TLR4
Pancreatic Adenocarcinoma Signaling	3.51	0.06	CYP2E1,HBEGF,HDAC7,HMOX1,NOTCH1,PA2G4,RAC1,VEGFB
Reelin Signaling in Neurons	3.48	0.06	ARHGEF1,CDK5R1,HCK,PAFAH1B2,PDK2,PDK3,PDK4,RAC1
RAR Activation	3.47	0.05	ADH1C,ADH7,CSF2RB,EP300,MMP1,PRKCB,RAC1,RARA,RARG,RBP5

Gαq Signaling	3.34	0.05	BTK,CAMK4,GRK2,HMOX1,NFATC1,PRKCB,PTK2B,RAC1,RND1
Sumoylation Pathway	3.32	0.07	AR,EP300,FAS,RAC1,RND1,SENP2,XIAP
Iron homeostasis signaling pathway	3.30	0.06	BMP1,BTRC,CIAO1,CUL1,HBB,HMOX1,HSCB,SKP1
IL-12 Signaling and Production in Macrophages	3.28	0.06	ALB,ALOX12,EP300,IFNG,IFNGR1,PPARG,PRKCB,TLR4
HIF1α Signaling	3.28	0.05	APEX1,CAMK4,EP300,HK2,HMOX1,MMP1,MMP2,PRKCB,RAC1,VEGFB
Sperm Motility	3.25	0.05	ALK,BTK,CAMK4,CLK1,EPHA3,ERBB4,HCK,IGF1R,MATK,PRKCB,PTK2B
WNT/β-catenin Signaling	3.24	0.05	ACVR2B,APPL1,BTRC,CSNK1G1,CSNK1G2,EP300,PPP2R1A,RARA,RARG
ERK/MAPK Signaling	3.13	0.05	ESR1,ITGB3,NFATC1,PPARG,PPP2R1A,PRKCB,PTK2B,RAC1,YWHAH,YWHAQ
Urate Biosynthesis/Inosine 5'-phosphate Degradation	3.08	0.21	ACP3,IMPDH1,NT5C3A
Role of Osteoblasts, Osteoclasts and Chondrocytes in Rheumatoid Arthritis	3.04	0.05	BMP1,CAMK4,CSNK1G1,CSNK1G2,IFNG,ITGB3,MMP1,NFATC1,PTK2B,XIAP
Aryl Hydrocarbon Receptor Signaling	3.02	0.05	EP300,ESR1,FAS,GSTT2/GSTT2B,HSP90AB1,NCOA3,RARA,RARG
Colorectal Cancer Metastasis Signaling	3.00	0.04	APPL1,GRK2,IFNG,IFNGR1,MMP1,MMP2,MSH2,RAC1,RND1,TLR4,VEGFB
Telomere Extension by Telomerase	2.99	0.20	HNRNPA2B1,POT1,XRCC6
Neuregulin Signaling	2.98	0.06	CDK5R1,ERBB4,HBEGF,HSP90AB1,ITGB3,MATK,PRKCB
Production of Nitric Oxide and Reactive Oxygen Species in Macrophages	2.93	0.05	ALB,IFNG,IFNGR1,MAP3K3,PPP2R1A,PRKCB,RAC1,RND1,TLR4
Leukocyte Extravasation Signaling	2.92	0.05	ARHGAP5,BTK,MMP1,MMP2,MSN,PRKCB,PTK2B,RAC1,VAV3
Superpathway of Geranylgeranyldiphosphate Biosynthesis I (via Mevalonate)	2.83	0.18	ACAT2,GGPS1,HMGCS1
LXR/RXR Activation	2.82	0.06	ALB,CYP7A1,ECHS1,GC,HADH,TLR4,TTR
HOTAIR Regulatory Pathway	2.80	0.05	AR,EP300,ESR1,MMP1,MMP2,TLR4,XIAP,YY1
GP6 Signaling Pathway	2.80	0.06	BTK,CAMK4,CERT1,ITGB3,PRKCB,RAC1,VAV3
Mitotic Roles of Polo-Like Kinase	2.77	0.08	HSP90AB1,KIF11,PPP2R1A,SLK,WEE1
Antigen Presentation Pathway	2.76	0.11	B2M,CD74,HLA-DRB1,IFNG
p70S6K Signaling	2.70	0.05	BTK,F2,PPP2R1A,PRKCB,SFN,YWHAH,YWHAQ
Purine Nucleotides Degradation II (Aerobic)	2.68	0.16	ACP3,IMPDH1,NT5C3A
Cell Cycle: G1/S Checkpoint Regulation	2.65	0.07	BTRC,CUL1,HDAC7,PA2G4,SKP1
IL-8 Signaling	2.65	0.04	HBEGF,HMOX1,ITGB3,MMP2,PRKCB,PTK2B,RAC1,RND1,VEGFB
RHOGDI Signaling	2.58	0.04	ARHGAP5,ARHGEF1,EP300,ESR1,ITGB3,MSN,PIP4K2C,RAC1,RND1

PI3K Signaling in B Lymphocytes	2.55	0.05	BTK,CAMK4,NFATC1,PRKCB,RAC1,TLR4,VAV3
MSP-RON Signaling In Cancer Cells Pathway	2.55	0.05	CSF2RB,KLK1,PTK2B,SFN,VEGFB,YWHAH,YWHAQ
Virus Entry via Endocytic Pathways	2.51	0.06	B2M,FLNB,ITGB3,ITSN1,PRKCB,RAC1
NAD Salvage Pathway II	2.49	0.14	ACP3,NMRK1,NT5C3A
Pyrimidine Deoxyribonucleotides De Novo Biosynthesis I	2.49	0.14	DTYMK,NME3,RRM1
iNOS Signaling	2.45	0.09	CAMK4,IFNG,IFNGR1,TLR4
Caveolar-mediated Endocytosis Signaling	2.44	0.07	ALB,B2M,FLNB,ITGB3,ITSN1
Glycoaminoglycan-protein Linkage Region Biosynthesis	2.43	0.29	B3GAT2,B3GAT3
VDR/RXR Activation	2.39	0.06	EP300,IFNG,NCOA3,PRKCB,YY1
Protein Ubiquitination Pathway	2.38	0.04	B2M,BTRC,CUL1,HSCB,HSP90AB1,IFNG,SKP1,SMURF2,USP19,XIAP
Neuroprotective Role of THOP1 in Alzheimer's Disease	2.37	0.05	C1R,DPP4,FAP,IDE,IFNG,KLK1
Xenobiotic Metabolism Signaling	2.37	0.04	CAMK4,EP300,GSTT2/GSTT2B,HMOX1,HSP90AB1,MAP3K3,NR1I3,PPP2R1A,PRKCB,SULT1A1
Phosphatidylethanolamine Biosynthesis II	2.31	0.25	CHKA,PCYT2
Natural Killer Cell Signaling	2.28	0.04	B2M,FCGR2A,IFNG,MAP3K3,NFATC1,PTK2B,RAC1,VAV3
ILK Signaling	2.28	0.04	FLNB,FN1,ITGB3,MUC1,PPP2R1A,RAC1,RND1,VEGFB
Inhibition of ARE-Mediated mRNA Degradation Pathway	2.20	0.04	EXOSC9,MAPK6,PARN,PPP2R1A,SFN,YWHAH,YWHAQ
Superpathway of Cholesterol Biosynthesis	2.19	0.11	ACAT2,GGPS1,HMGCS1
RHOA Signaling	2.17	0.05	ARHGAP5,ARHGEF1,IGF1R,MSN,PIP4K2C,PTK2B
CSDE1 Signaling Pathway	2.17	0.07	CSDE1,FABP7,FN1,RAC1
Serotonin Degradation	2.14	0.07	ADH1C,ADH7,B3GAT3,SULT1A1
Protein Kinase A Signaling	2.13	0.03	APEX1,CAMK4,FLNB,NFATC1,PRKCB,PTK2B,PTPRE,PTPRF,PYGL,SFN,YWHAH,YWHAQ
Molecular Mechanisms of Cancer	2.13	0.03	ARHGEF1,BMP1,EP300,FAS,HAT1,HDAC7,ITGB3,NOTCH1,PA2G4,PRKCB,RAC1,RND1,XIAP
Senescence Pathway	2.12	0.03	ACVR2B,CAMK4,EP300,MAPK6,NFATC1,PDHA1,PDK2,PDK3,PDK4,PPP2R1A
FXR/RXR Activation	2.12	0.05	ALB,CYP7A1,GC,PPARG,RARA,TTR

RANK Signaling in Osteoclasts	2.12	0.06	CAMK4,MAP3K3,NFATC1,PTK2B,XIAP
Retinoic acid Mediated Apoptosis Signaling	2.12	0.07	IFNG,PARP3,RARA,RARG
Noradrenaline and Adrenaline Degradation	2.11	0.10	ADH1C,ADH7,PNMT
MSP-RON Signaling Pathway	2.09	0.07	CSF2RB,IFNG,KLK1,TLR4
Crosstalk between Dendritic Cells and Natural Killer Cells	2.08	0.05	CSF2RB,FAS,HLA-DRB1,IFNG,TLR4
Xenobiotic Metabolism CAR Signaling Pathway	2.03	0.04	EP300,GSTT2/GSTT2B,HSP90AB1,NR1I3,PPP2R1A,PRKCB,SULT1A1
Ketogenesis	2.02	0.18	ACAT2,HMGCS1
Glycogen Degradation II	2.02	0.18	MTAP,PYGL
Purine Nucleotides De Novo Biosynthesis II	2.02	0.18	GART,IMPDH1
Retinoate Biosynthesis I	1.99	0.09	ADH1C,ADH7,RBP5
Role of Macrophages, Fibroblasts and Endothelial Cells in Rheumatoid Arthritis	1.95	0.03	CAMK4,CSNK1G1,CSNK1G2,FN1,MMP1,NFATC1,PRKCB,RAC1,TLR4,VEGFB
Tumor Microenvironment Pathway	1.94	0.04	FAS,FN1,ITGB3,MMP1,MMP2,RAC1,VEGFB
Hepatic Fibrosis Signaling Pathway	1.94	0.03	ACVR2B,CAMK4,CSNK1G1,CSNK1G2,ITGB3,MMP1,PPARG,PRKCB,RAC1,RND1,TLR4,VEGFB
HER-2 Signaling in Breast Cancer	1.92	0.04	ARF6,HBEGF,HCK,ITGB3,MMP2,NUP214,PRKCB,XIAP
Xenobiotic Metabolism General Signaling Pathway	1.92	0.04	EP300,GSTT2/GSTT2B,HMOX1,MAP3K3,NR1I3,PRKCB
Coagulation System	1.92	0.09	F2,F3,SERPINA5
Glycogen Degradation III	1.88	0.15	MTAP,PYGL
Mevalonate Pathway I	1.88	0.15	ACAT2,HMGCS1
Guanosine Nucleotides Degradation III	1.88	0.15	ACP3,NT5C3A
Opioid Signaling Pathway	1.88	0.03	CAMK4,EP300,GRK2,GRK6,HCK,MAPK6,PRKCB,RAC1,RGS6
Acute Phase Response Signaling	1.87	0.04	ALB,C1R,F2,FN1,HMOX1,RBP5,TTR
Acyl Carrier Protein Metabolism	1.86	1.00	AASDHPPT
GNRH Signaling	1.86	0.04	CAMK4,HBEGF,MAP3K3,MMP2,PRKCB,PTK2B,RAC1
Osteoarthritis Pathway	1.85	0.03	FN1,ITGB3,MMP1,NOTCH1,PPARG,RAC1,TLR4,VEGFB
TEC Kinase Signaling	1.84	0.03	BTK,FAS,GTF2I,HCK,ITGB3,PRKCB,PTK2B,RAC1,RND1,TLR4,VAV3
tRNA Charging	1.82	0.08	LARS1,WARS1,YARS1
Inhibition of Matrix Metalloproteases	1.82	0.08	MMP1,MMP2,THBS2

Glioma Invasiveness Signaling	1.79	0.06	ITGB3,MMP2,RAC1,RND1
Sirtuin Signaling Pathway	1.78	0.03	APEX1,G6PD,MAPK6,NDUFAB1,PDHA1,PGK1,PPARG,TIMM9,XRCC6
ERK5 Signaling	1.77	0.06	MAP3K3,SFN,YWHAH,YWHAQ
Axonal Guidance Signaling	1.76	0.03	BMP1,EPHA3,ITGB3,ITSN1,MMP1,MMP2,MYSM1,NFATC1,PRKCB,RAC1,RND1,SEMA4D,VEGFB
Actin Cytoskeleton Signaling	1.75	0.03	ARHGEF1,F2,FN1,ITGB3,MATK,MSN,RAC1,VAV3
Estrogen Biosynthesis	1.74	0.07	CYP2E1,HSD17B10,HSD17B4
Role of WNT/GSK-3β Signaling in the Pathogenesis of Influenza	1.73	0.05	CSNK1G1,CSNK1G2,IFNG,NCOA3
PI3K/AKT Signaling	1.72	0.04	CSF2RB,HSP90AB1,ITGB3,PPP2R1A,SFN,YWHAH,YWHAQ
Extrinsic Prothrombin Activation Pathway	1.71	0.13	F2,F3
Adenosine Nucleotides Degradation II	1.71	0.13	ACP3,NT5C3A
Bladder Cancer Signaling	1.69	0.04	HDAC7,MMP1,MMP2,PA2G4,VEGFB
MSP-RON Signaling In Macrophages Pathway	1.69	0.04	HLA-DRB1,IFNG,IFNGR1,KLK1,TLR4
Macropinocytosis Signaling	1.69	0.05	ARF6,ITGB3,PRKCB,RAC1
Estrogen-Dependent Breast Cancer Signaling	1.67	0.05	ESR1,HSD17B10,HSD17B4,IGF1R
Estrogen Receptor Signaling	1.67	0.03	EP300,ESR1,HSP90AB1,IGF1R,MMP1,MMP2,NCOA3,NDUFAB1,NOTCH1,PRKCB,VEGFB
Granzyme A Signaling	1.66	0.12	APEX1,EP300
HMGB1 Signaling	1.65	0.04	HAT1,IFNG,IFNGR1,RAC1,RND1,TLR4
Th1 Pathway	1.65	0.04	HLA-DRB1,IFNG,IFNGR1,NFATC1,NOTCH1
BEX2 Signaling Pathway	1.62	0.05	KLK1,MMP2,PPP2R1A,VEGFB
1D-myo-inositol Hexakisphosphate Biosynthesis II (Mammalian)	1.61	0.11	ITPK1,ITPKC
Phospholipase C Signaling	1.58	0.03	ARHGEF1,BTK,CAMK4,EP300,FCGR2A,HDAC7,HMOX1,ITGB3,NFATC1,PRKCB,RAC1,RND1
Glioma Signaling	1.58	0.04	CAMK4,HDAC7,IGF1R,PA2G4,PRKCB
NRF2-mediated Oxidative Stress Response	1.58	0.03	CYP2E1,CYP2R1,EP300,GSTT2/GSTT2B,HMOX1,HSP90AB1,PRKCB
Ephrin A Signaling	1.58	0.06	EPHA3,RAC1,VAV3
S-methyl-5'-thioadenosine Degradation II	1.57	0.50	MTAP
Sulfate Activation for Sulfonation	1.57	0.50	PAPSS1

Putrescine Biosynthesis III	1.57	0.50	ODC1
Glutamate Dependent Acid Resistance	1.57	0.50	GAD2
Th1 and Th2 Activation Pathway	1.56	0.04	ACVR2B,HLA-DRB1,IFNG,IFNGR1,NFATC1,NOTCH1
TR/RXR Activation	1.55	0.05	CAMK4,CYP7A1,EP300,NCOA3
Erythropoietin Signaling Pathway	1.55	0.04	CSF2RB,HBB,IFNG,PRKCB,RAC1,XIAP
Androgen Signaling	1.55	0.04	AR,CAMK4,EP300,GTF2F2,PRKCB,TAF13
Atherosclerosis Signaling	1.53	0.04	ALB,ALOX12,F3,IFNG,MMP1
Nicotine Degradation III	1.51	0.06	ADH7,B3GAT3,CYP2E1
Chondroitin Sulfate Biosynthesis	1.47	0.06	B3GAT2,B3GAT3,SULT1A1
Tight Junction Signaling	1.45	0.03	CSTF2,MAGI2,PATJ,PPP2R1A,RAC1,TJP1
Role of PKR in Interferon Induction and Antiviral Response	1.45	0.04	FAS,HSP90AB1,IFNG,IFNGR1,TLR4
Melatonin Degradation I	1.44	0.06	B3GAT3,CYP2E1,SULT1A1
NUR77 Signaling in T Lymphocytes	1.44	0.03	B2M,CAMK4,EP300,HDAC7,HLA-DRB1,MAP3K3,NFATC1,PRKCB
Gα12/13 Signaling	1.44	0.04	ARHGEF1,BTK,F2,PTK2B,VAV3
Non-Small Cell Lung Cancer Signaling	1.41	0.04	FHIT,HDAC7,PA2G4,STK4
Superpathway of D-myo-inositol (1,4,5)-trisphosphate Metabolism	1.41	0.09	IMPA2,ITPKC
Dermatan Sulfate Biosynthesis	1.40	0.05	B3GAT2,B3GAT3,SULT1A1
Nicotine Degradation II	1.40	0.05	ADH7,B3GAT3,CYP2E1
Death Receptor Signaling	1.40	0.04	DFFA,FAS,PARP3,XIAP
Hepatic Cholestasis	1.39	0.03	CYP7A1,ESR1,IFNG,PRKCB,RARA,TLR4
RAC Signaling	1.39	0.04	CDK5R1,ITGB3,PIP4K2C,PTK2B,RAC1
5-aminoimidazole Ribonucleotide Biosynthesis I	1.39	0.33	GART
1,25-dihydroxyvitamin D3 Biosynthesis	1.39	0.33	CYP2R1
D-glucuronate Degradation I	1.39	0.33	DCXR
Hypusine Biosynthesis	1.39	0.33	DHPS
Geranylgeranyldiphosphate Biosynthesis	1.39	0.33	GGPS1
Biotin-carboxyl Carrier Protein Assembly	1.39	0.33	ACACB
1D-myo-inositol Hexakisphosphate Biosynthesis V (from Ins(1,3,4)P3)	1.39	0.33	ITPK1

Tyrosine Biosynthesis IV	1.39	0.33	PAH
Fatty Acid β-oxidation III (Unsaturated, Odd Number)	1.39	0.33	ECI2
Tumoricidal Function of Hepatic Natural Killer Cells	1.37	0.08	DFFA,FAS
Hereditary Breast Cancer Signaling	1.37	0.04	EP300,HDAC7,MSH2,SFN,WEE1
VEGF Signaling	1.35	0.04	PRKCB,PTK2B,SFN,VEGFB
Superpathway of Melatonin Degradation	1.35	0.05	B3GAT3,CYP2E1,SULT1A1
Gluconeogenesis I	1.34	0.08	MDH1,PGK1
Semaphorin Signaling in Neurons	1.33	0.05	RAC1,RND1,SEMA4D
Role of JAK1, JAK2 and TYK2 in Interferon Signaling	1.31	0.08	IFNG,IFNGR1
Clathrin-mediated Endocytosis Signaling	1.31	0.03	ALB,ARF6,F2,ITGB3,RAC1,VEGFB
Adrenomedullin signaling pathway	1.29	0.03	CAMK4,MAPK6,MATK,MMP2,PPARG,PTK2B
Ethanol Degradation II	1.28	0.07	ADH1C,ADH7
PD-1, PD-L1 cancer immunotherapy pathway	1.28	0.04	B2M,HLA-DRB1,IFNG,IFNGR1
Retinoate Biosynthesis II	1.27	0.25	RBP5
Pentose Phosphate Pathway (Oxidative Branch)	1.27	0.25	G6PD
Heme Degradation	1.27	0.25	HMOX1
Catecholamine Biosynthesis	1.27	0.25	PNMT
Phenylethylamine Degradation I	1.27	0.25	AOC3
Glutathione Redox Reactions II	1.27	0.25	TXNDC12
Arginine Degradation I (Arginase Pathway)	1.27	0.25	OAT
Trans, trans-farnesyl Diphosphate Biosynthesis	1.27	0.25	GGPS1
Phenylalanine Degradation I (Aerobic)	1.27	0.25	PAH
Telomerase Signaling	1.26	0.04	HDAC7,HSP90AB1,POT1,PPP2R1A
IGF-1 Signaling	1.26	0.04	IGF1R,SFN,YWHAH,YWHAQ
Induction of Apoptosis by HIV1	1.26	0.05	DFFA,FAS,XIAP
PTEN Signaling	1.26	0.03	IGF1R,ITGB3,MAGI2,RAC1,YWHAH
Paxillin Signaling	1.24	0.04	ARF6,ITGB3,PTK2B,RAC1

eNOS Signaling	1.24	0.03	CAMK4,ESR1,HSP90AB1,PRKCB,VEGFB
Oxidative Ethanol Degradation III	1.23	0.07	CYP2E1,CYP2R1
Cardiac Hypertrophy Signaling (Enhanced)	1.23	0.02	ACVR2B,APEX1,CAMK4,CSF2RB,EP300,HDAC7,IFNG,IGF1R,ITGB3,MAP3K3,NFATC1,PRKCB
Heparan Sulfate Biosynthesis	1.21	0.05	B3GAT2,B3GAT3,SULT1A1
mTOR Signaling	1.21	0.03	HMOX1,PPP2R1A,PRKCB,RAC1,RND1,VEGFB
Prostate Cancer Signaling	1.20	0.04	AR,HDAC7,HSP90AB1,PA2G4
Thrombin Signaling	1.20	0.03	ARHGEF1,CAMK4,F2,PRKCB,RAC1,RND1
Integrin Signaling	1.20	0.03	ARF6,ARHGAP5,ASAP1,ITGB3,RAC1,RND1
Ovarian Cancer Signaling	1.20	0.03	HDAC7,MMP2,MSH2,PA2G4,VEGFB
Regulation of Actin-based Motility by Rho	1.19	0.04	ITGB3,PIP4K2C,RAC1,RND1
B Cell Receptor Signaling	1.19	0.02	BTK,CAMK4,FCGR2A,MAP3K3,NFATC1,PRKCB,PTK2B,RAC1,VAV3
Agrin Interactions at Neuromuscular Junction	1.18	0.04	ERBB4,RAC1,UTRN
Proline Biosynthesis II (from Arginine)	1.18	0.20	OAT
Tetrahydrofolate Salvage from 5,10-methenyltetrahydrofolate	1.18	0.20	GART
NAD Salvage Pathway III	1.18	0.20	NMRK1
Lysine Degradation II	1.18	0.20	AASDHPPT
Myo-inositol Biosynthesis	1.18	0.20	IMPA2
Lysine Degradation V	1.18	0.20	AASDHPPT
Galactose Degradation I (Leloir Pathway)	1.18	0.20	GALM
Lactose Degradation III	1.18	0.20	PSAP
Tyrosine Degradation I	1.18	0.20	HPD
Glutamate Degradation III (via 4-aminobutyrate)	1.18	0.20	GAD2
Thyroid Hormone Metabolism II (via Conjugation and/or Degradation)	1.15	0.06	B3GAT3,SULT1A1
GM-CSF Signaling	1.15	0.04	CSF2RB,HCK,PRKCB
Nitric Oxide Signaling in the Cardiovascular System	1.15	0.04	CAMK4,HSP90AB1,PRKCB,VEGFB
Signaling by Rho Family GTPases	1.14	0.03	ARHGEF1,ITGB3,MSN,PIP4K2C,PTK2B,RAC1,RND1
Airway Inflammation in Asthma	1.13	0.06	IFNG,IFNGR1

Pyrimidine Ribonucleotides Interconversion	1.13	0.06	CANT1,NME3
Phagosome Formation	1.12	0.02	ARF6,FCGR2A,FN1,HCK,HMOX1,HTR1A,ITGB3,MAPK6,PIP4K2C,PRKCB,PTK2B,RAC1,TLR4,VAV3
Ephrin B Signaling	1.12	0.04	ITSN1,RAC1,VAV3
Cholecystokinin/Gastrin-mediated Signaling	1.11	0.03	PRKCB,PTK2B,RAC1,RND1
PAK Signaling	1.11	0.03	EPHA3,ITGB3,PTK2B,RAC1
Fc Epsilon RI Signaling	1.10	0.03	BTK,PRKCB,RAC1,VAV3
Arginine Biosynthesis IV	1.10	0.17	OAT
Trehalose Degradation II (Trehalase)	1.10	0.17	HK2
Arginine Degradation VI (Arginase 2 Pathway)	1.10	0.17	OAT
Acetyl-CoA Biosynthesis I (Pyruvate Dehydrogenase Complex)	1.10	0.17	PDHA1
Tryptophan Degradation to 2-amino-3-carboxymuconate Semialdehyde	1.10	0.17	KYNU
Pyrimidine Ribonucleotides De Novo Biosynthesis	1.08	0.06	CANT1,NME3
MIF-mediated Glucocorticoid Regulation	1.06	0.06	CD74,TLR4
Interferon Signaling	1.06	0.06	IFNG,IFNGR1
Complement System	1.06	0.06	C1R,C8A
Ferroptosis Signaling Pathway	1.05	0.03	ALOX12,ARF6,DPP4,HMOX1
Chemokine Signaling	1.05	0.04	CAMK4,PRKCB,PTK2B
Phosphatidylcholine Biosynthesis I	1.04	0.14	CHKA
Aspartate Degradation II	1.04	0.14	MDH1
IL-3 Signaling	1.03	0.04	CSF2RB,PRKCB,RAC1
Xenobiotic Metabolism AHR Signaling Pathway	1.03	0.04	EP300,GSTT2/GSTT2B,HSP90AB1
Xenobiotic Metabolism PXR Signaling Pathway	1.02	0.03	EP300,GSTT2/GSTT2B,HSP90AB1,PRKCB,SULT1A1
14-3-3-mediated Signaling	1.02	0.03	PRKCB,SFN,YWHAH,YWHAQ
Dopamine-DARPP32 Feedback in cAMP Signaling	1.02	0.03	CAMK4,CSNK1G1,CSNK1G2,PPP2R1A,PRKCB

Role of BRCA1 in DNA Damage Response	1.01	0.04	IFNG,MSH2,POU2F1
Systemic Lupus Erythematosus In T Cell Signaling Pathway	0.99	0.02	B2M,CAMK4,ESR1,FAS,HLA-DRB1,MSN,PPP2R1A,RAC1,RND1
PPARα/RXRα Activation	0.99	0.03	ACVR2B,EP300,HSP90AB1,NCOA3,PRKCB
fMLP Signaling in Neutrophils	0.99	0.03	CAMK4,NFATC1,PRKCB,RAC1
Sucrose Degradation V (Mammalian)	0.98	0.13	GALM
Ketolysis	0.98	0.13	ACAT2
HGF Signaling	0.98	0.03	ITGB3,MAP3K3,PRKCB,RAC1
Apelin Adipocyte Signaling Pathway	0.98	0.04	GSTT2/GSTT2B,MAPK6,RAC1
Intrinsic Prothrombin Activation Pathway	0.97	0.05	F2,KLK1
PEDF Signaling	0.97	0.04	FAS,PPARG,RAC1
Adipogenesis pathway	0.96	0.03	HAT1,HDAC7,PPARG,SENP2
Th2 Pathway	0.96	0.03	ACVR2B,HLA-DRB1,IFNG,NOTCH1
LPS-stimulated MAPK Signaling	0.96	0.04	PRKCB,RAC1,TLR4
BMP signaling pathway	0.96	0.04	BMP1,CAMK4,XIAP
Citrulline Biosynthesis	0.93	0.11	OAT
Glucocorticoid Biosynthesis	0.93	0.11	CYP17A1
GDP-glucose Biosynthesis	0.93	0.11	HK2
MIF Regulation of Innate Immunity	0.92	0.05	CD74,TLR4
Coronavirus Pathogenesis Pathway	0.89	0.03	AR,EP300,HDAC7,IFNG,PA2G4
Actin Nucleation by ARP-WASP Complex	0.89	0.03	ITGB3,RAC1,RND1
Glucose and Glucose-1-phosphate Degradation	0.89	0.10	HK2
NAD Phosphorylation and Dephosphorylation	0.89	0.10	ACP3
Pentose Phosphate Pathway	0.89	0.10	G6PD
Synaptogenesis Signaling Pathway	0.89	0.02	CAMK4,EPHA3,HCK,ITSN1,RAC1,SYT13,THBS2
nNOS Signaling in Neurons	0.88	0.04	CAMK4,PRKCB
PFKFB4 Signaling Pathway	0.88	0.04	HK2,NCOA3
Cardiac Hypertrophy Signaling	0.87	0.02	CAMK4,EP300,IGF1R,MAP3K3,RAC1,RND1
Agranulocyte Adhesion and Diapedesis	0.87	0.03	AOC3,FN1,MMP1,MMP2,MSN
ERBB Signaling	0.87	0.03	ERBB4,HBEGF,PRKCB
Small Cell Lung Cancer Signaling	0.86	0.03	FHIT,HDAC7,PA2G4

Semaphorin Neuronal Repulsive Signaling Pathway	0.86	0.03	ITGB3,RAC1,RND1,SEMA4D
Ephrin Receptor Signaling	0.86	0.03	EPHA3,ITGB3,ITSN1,RAC1,VEGFB
Dilated Cardiomyopathy Signaling Pathway	0.85	0.03	CAMK4,DSG2,MAP3K3,MYBPC3
NAD biosynthesis II (from tryptophan)	0.85	0.09	KYNU
Sertoli Cell-Sertoli Cell Junction Signaling	0.84	0.02	ITGB3,MAGI2,MAP3K3,RAC1,TJP1
Salvage Pathways of Pyrimidine Ribonucleotides	0.84	0.03	GRK6,MAPK6,NME3
TGF-β Signaling	0.84	0.03	ACVR2B,EP300,SMURF2
T Cell Receptor Signaling	0.83	0.02	B2M,CAMK4,HLA-DRB1,IFNG,NFATC1,PTK2B,RAC1,VAV3
Amyloid Processing	0.83	0.04	CDK5R1,MARK1
FAT10 Cancer Signaling Pathway	0.83	0.04	ACVR2B,IFNG
p53 Signaling	0.82	0.03	EP300,FAS,SFN
Cleavage and Polyadenylation of Pre-mRNA	0.82	0.08	CSTF2
UDP-N-acetyl-D-galactosamine Biosynthesis II	0.82	0.08	HK2
Necroptosis Signaling Pathway	0.80	0.03	FAS,PYGL,TIMM9,TLR4
Bile Acid Biosynthesis, Neutral Pathway	0.79	0.08	CYP7A1
Apoptosis Signaling	0.79	0.03	DFFA,FAS,XIAP
Systemic Lupus Erythematosus In B Cell Signaling Pathway	0.78	0.02	BTK,CAMK4,FCGR2A,HCK,IFNG,IFNGR1,NFATC1,PRKCB,RAC1
α-Adrenergic Signaling	0.77	0.03	CAMK4,PRKCB,PYGL
PPAR Signaling	0.77	0.03	EP300,HSP90AB1,PPARG
G-Protein Coupled Receptor Signaling	0.76	0.02	APEX1,CAMK4,GRK2,HTR1A,PRKCB,PTK2B
DNA Double-Strand Break Repair by Non-Homologous End Joining	0.76	0.07	XRCC6
Androgen Biosynthesis	0.76	0.07	CYP17A1
Choline Biosynthesis III	0.76	0.07	HMOX1
Phenylalanine Degradation IV (Mammalian, via Side Chain)	0.76	0.07	HPD
Aldosterone Signaling in Epithelial Cells	0.75	0.03	HSCB,HSP90AB1,PIP4K2C,PRKCB
Role of NFAT in Cardiac Hypertrophy	0.75	0.02	CAMK4,EP300,HDAC7,IGF1R,PRKCB

Superpathway of Citrulline Metabolism	0.73	0.07	OAT
Methylglyoxal Degradation III	0.73	0.07	CYP2E1
Germ Cell-Sertoli Cell Junction Signaling	0.71	0.02	MAP3K3,RAC1,RND1,TJP1
CDK5 Signaling	0.71	0.03	CDK5R1,MAPK6,PPP2R1A
Polyamine Regulation in Colon Cancer	0.70	0.03	ODC1,PPARG
Granzyme B Signaling	0.70	0.06	DFFA
Mismatch Repair in Eukaryotes	0.70	0.06	MSH2
Pregnenolone Biosynthesis	0.70	0.06	CYP2E1
Glioblastoma Multiforme Signaling	0.70	0.02	IGF1R,NF2,RAC1,RND1
Airway Pathology in Chronic Obstructive Pulmonary Disease	0.70	0.03	IFNG,MMP1,MMP2
Superpathway of Inositol Phosphate Compounds	0.70	0.02	ITPK1,ITPKC,PIP4K2C,PPP2R1A,PTPRF
Amyotrophic Lateral Sclerosis Signaling	0.69	0.03	RAC1,VEGFB,XIAP
Sphingosine-1-phosphate Signaling	0.68	0.03	PTK2B,RAC1,RND1
D-myo-inositol (1,4,5)-trisphosphate Degradation	0.68	0.06	IMPA2
FAK Signaling	0.67	0.03	ASAP1,ITGB3,RAC1
PXR/RXR Activation	0.67	0.03	CYP7A1,NR1I3
D-myo-inositol (1,3,4)-trisphosphate Biosynthesis	0.66	0.06	ITPKC
Histidine Degradation VI	0.66	0.06	CYP2E1
Renin-Angiotensin Signaling	0.65	0.03	PRKCB,PTK2B,RAC1
Pyridoxal 5'-phosphate Salvage Pathway	0.65	0.03	GRK6,MAPK6
ERBB4 Signaling	0.64	0.03	ERBB4,PRKCB
IL-17 Signaling	0.64	0.02	HSP90AB1,IFNG,MMP2,VEGFB
The Visual Cycle	0.64	0.05	RBP5
DNA damage-induced 14-3-3 蝰 Signaling	0.64	0.05	SFN
Role of Lipids/Lipid Rafts in the Pathogenesis of Influenza	0.62	0.05	IFNG
Ubiquinol-10 Biosynthesis (Eukaryotic)	0.62	0.05	CYP2E1
Inflammasome pathway	0.62	0.05	TLR4

Synaptic Long Term Potentiation	0.61	0.02	CAMK4,EP300,PRKCB
Melatonin Signaling	0.61	0.03	CAMK4,PRKCB
Growth Hormone Signaling	0.60	0.03	IGF1R,PRKCB
D-myo-inositol-5-phosphate Metabolism	0.59	0.02	ITPK1,PIP4K2C,PPP2R1A,PTPRF
IL-10 Signaling	0.59	0.03	FCGR2A,HMOX1
Netrin Signaling	0.59	0.03	NFATC1,RAC1
Hypoxia Signaling in the Cardiovascular System	0.59	0.03	EP300,HSP90AB1
G Beta Gamma Signaling	0.59	0.02	BTK,HBEGF,PRKCB
IL-7 Signaling Pathway	0.58	0.03	IFNG,NFATC1
CCR3 Signaling in Eosinophils	0.58	0.02	CAMK4,PRKCB,RAC1
Role of NFAT in Regulation of the Immune Response	0.57	0.02	BTK,CAMK4,CSNK1G1,CSNK1G2,FCGR2A,HLA-DRB1,NFATC1
Regulation Of The Epithelial Mesenchymal Transition By Growth Factors Pathway	0.57	0.02	MMP1,MMP2,RAC1,ZEB2
Role of MAPK Signaling in Inhibiting the Pathogenesis of Influenza	0.57	0.03	IFNG,TLR4
Differential Regulation of Cytokine Production in Intestinal Epithelial Cells by IL-17A and IL-17F	0.57	0.04	IFNG
Dopamine Degradation	0.57	0.04	SULT1A1
Glutathione Redox Reactions I	0.57	0.04	GSTT2/GSTT2B
Gap Junction Signaling	0.56	0.02	CSNK1G1,CSNK1G2,PRKCB,TJP1
GDNF Family Ligand-Receptor Interactions	0.55	0.03	DOK2,RAC1
STAT3 Pathway	0.55	0.02	CSF2RB,IGF1R,RAC1
White Adipose Tissue Browning Pathway	0.55	0.02	PPARG,RARA,RARG
Bupropion Degradation	0.55	0.04	CYP2E1
TCA Cycle II (Eukaryotic)	0.55	0.04	MDH1
Glycolysis I	0.55	0.04	PGK1
Maturity Onset Diabetes of Young (MODY) Signaling	0.55	0.03	APPL1,EP300
3-phosphoinositide Biosynthesis	0.54	0.02	ITPK1,PIP4K2C,PPP2R1A,PTPRF

NF-κB Activation by Viruses	0.54	0.03	ITGB3,PRKCB
Renal Cell Carcinoma Signaling	0.54	0.03	EP300,RAC1
Role of Hypercytokinemia/hyperchemokinemia in the Pathogenesis of Influenza	0.52	0.03	IFNG,TLR4
Estrogen-mediated S-phase Entry	0.52	0.04	ESR1
D-myo-inositol (1,4,5)-Trisphosphate Biosynthesis	0.52	0.04	PIP4K2C
Glutathione-mediated Detoxification	0.52	0.04	GSTT2/GSTT2B
Apelin Liver Signaling Pathway	0.52	0.04	FAS
Calcium Signaling	0.50	0.02	CAMK4,EP300,HDAC7,NFATC1
VEGF Family Ligand-Receptor Interactions	0.50	0.02	PRKCB,VEGFB
Role of p14/p19ARF in Tumor Suppression	0.50	0.04	RAC1
Role of Pattern Recognition Receptors in Recognition of Bacteria and Viruses	0.49	0.02	IFNG,PRKCB,TLR4
Factors Promoting Cardiogenesis in Vertebrates	0.49	0.02	ACVR2B,BMP1,PRKCB
Huntington's Disease Signaling	0.49	0.02	CDK5R1,EP300,HDAC7,IGF1R,PRKCB
Autophagy	0.49	0.02	CAMK4,DAPK2,PPP2R1A,TLR4
Prolactin Signaling	0.48	0.02	EP300,PRKCB
Sonic Hedgehog Signaling	0.48	0.03	GRK2
Phagosome Maturation	0.48	0.02	B2M,HLA-DRB1,RAC1
Lipid Antigen Presentation by CD1	0.47	0.02	ARF6,B2M,CD1A,PSAP
TNFR2 Signaling	0.46	0.03	XIAP
Acetone Degradation I (to Methylglyoxal)	0.46	0.03	CYP2E1
Acute Myeloid Leukemia Signaling	0.46	0.02	CSF2RB,RARA
IL-4 Signaling	0.46	0.02	HLA-DRB1,NFATC1
PKC 胄 Signaling in T Lymphocytes	0.45	0.02	HLA-DRB1,MAP3K3,NFATC1,POU2F1,RAC1,VAV3
Role of JAK2 in Hormone-like Cytokine Signaling	0.45	0.03	SH2B2
Melanocyte Development and Pigmentation Signaling	0.43	0.02	EP300,SH2B2

cAMP-mediated signaling	0.43	0.02	APEX1,CAMK4,GRK2,HTR1A
Nucleotide Excision Repair Pathway	0.42	0.03	RPA3
NF-κB Signaling	0.41	0.02	BTRC,EP300,IGF1R,MAP3K3,PRKCB,TLR4
Apelin Cardiomyocyte Signaling Pathway	0.41	0.02	MAPK6,PRKCB
CXCR4 Signaling	0.41	0.02	PRKCB,RAC1,RND1
B Cell Development	0.41	0.03	HLA-DRB1
TWEAK Signaling	0.41	0.03	XIAP
Mitochondrial Dysfunction	0.41	0.02	HSD17B10,NDUFAB1,PDHA1
Neuropathic Pain Signaling In Dorsal Horn Neurons	0.40	0.02	CAMK4,PRKCB
Cell Cycle Regulation by BTG Family Proteins	0.40	0.03	PPP2R1A
Notch Signaling	0.40	0.03	NOTCH1
Type I Diabetes Mellitus Signaling	0.40	0.02	FAS,GAD2,HLA-DRB1,IFNG,IFNGR1
IL-17A Signaling in Fibroblasts	0.39	0.03	MMP1
NER (Nucleotide Excision Repair, Enhanced Pathway)	0.39	0.02	EP300,RPA3
D-myo-inositol (1,4,5,6)-Tetrakisphosphate Biosynthesis	0.38	0.02	ITPK1,PPP2R1A,PTPRF
D-myo-inositol (3,4,5,6)-tetrakisphosphate Biosynthesis	0.38	0.02	ITPK1,PPP2R1A,PTPRF
Cardiac β-adrenergic Signaling	0.38	0.02	APEX1,GRK2,PPP2R1A
Retinol Biosynthesis	0.37	0.03	RBP5
Granulocyte Adhesion and Diapedesis	0.37	0.02	MMP1,MMP2,MSN
Chronic Myeloid Leukemia Signaling	0.37	0.02	HDAC7,PA2G4
Antioxidant Action of Vitamin C	0.37	0.02	CSF2RB,HMOX1
Mechanisms of Viral Exit from Host Cells	0.37	0.02	PRKCB
Allograft Rejection Signaling	0.37	0.02	B2M,FAS,HLA-DRB1,IFNG
April Mediated Signaling	0.36	0.02	NFATC1
Dermatan Sulfate Biosynthesis (Late Stages)	0.36	0.02	SULT1A1
Serotonin Receptor Signaling	0.36	0.02	HTR1A
B Cell Activating Factor Signaling	0.35	0.02	NFATC1

Oncostatin M Signaling	0.35	0.02	MMP1
Role of RIG1-like Receptors in Antiviral Innate Immunity	0.34	0.02	EP300
tRNA Splicing	0.34	0.02	APEX1
Chondroitin Sulfate Biosynthesis (Late Stages)	0.34	0.02	SULT1A1
BER (Base Excision Repair) Pathway	0.34	0.02	APEX1
Calcium-induced T Lymphocyte Apoptosis	0.34	0.02	CAMK4,EP300,HLA-DRB1,PRKCB
Role of OCT4 in Mammalian Embryonic Stem Cell Pluripotency	0.34	0.02	RARA
3-phosphoinositide Degradation	0.34	0.02	ITPK1,PPP2R1A,PTPRF
Endothelin-1 Signaling	0.33	0.02	HMOX1,MAPK6,PRKCB
Synaptic Long Term Depression	0.32	0.02	IGF1R,PPP2R1A,PRKCB
NGF Signaling	0.32	0.02	MAP3K3,RAC1
nNOS Signaling in Skeletal Muscle Cells	0.32	0.02	CAMK4
Regulation of IL-2 Expression in Activated and Anergic T Lymphocytes	0.31	0.02	CAMK4,NFATC1,RAC1,VAV3
Insulin Secretion Signaling Pathway	0.31	0.02	HCK,PC,PDHA1,PRKCB
Primary Immunodeficiency Signaling	0.31	0.02	BTK
Spliceosomal Cycle	0.31	0.02	SF3B6
Systemic Lupus Erythematosus Signaling	0.31	0.01	C8A,CAMK4,FCGR2A,HNRNPA2B1,KLK1,NFATC1
Regulation of the Epithelial-Mesenchymal Transition Pathway	0.31	0.02	MMP2,NOTCH1,ZEB2
MYC Mediated Apoptosis Signaling	0.30	0.02	FAS
TNFR1 Signaling	0.30	0.02	XIAP
Assembly of RNA Polymerase II Complex	0.30	0.02	TAF13
Endocannabinoid Developing Neuron Pathway	0.30	0.02	MAPK6,RAC1
UVC-Induced MAPK Signaling	0.30	0.02	PRKCB
Role of Cytokines in Mediating Communication between Immune Cells	0.29	0.02	IFNG
Stearate Biosynthesis I (Animals)	0.29	0.02	CYP2E1

UVB-Induced MAPK Signaling	0.29	0.02	PRKCB
P2Y Purigenic Receptor Signaling Pathway	0.28	0.02	ITGB3,PRKCB
Lymphotoxin β Receptor Signaling	0.28	0.02	EP300
Transcriptional Regulatory Network in Embryonic Stem Cells	0.28	0.02	ZIC3
FAT10 Signaling Pathway	0.28	0.02	IFNG
CD27 Signaling in Lymphocytes	0.27	0.02	MAP3K3
SPINK1 Pancreatic Cancer Pathway	0.27	0.02	KLK1
Cell Cycle Control of Chromosomal Replication	0.27	0.02	RPA3
Altered T Cell and B Cell Signaling in Rheumatoid Arthritis	0.27	0.01	FAS,HLA-DRB1,IFNG,TLR4
Role of CHK Proteins in Cell Cycle Checkpoint Control	0.26	0.02	PPP2R1A
Breast Cancer Regulation by Stathmin1	0.26	0.01	ARHGEF1,CAMK4,HTR1A,MMP2,PPP2R1A,PRKCB,RAC1,VEGFB
Role of IL-17A in Arthritis	0.26	0.02	MMP1
T Cell Exhaustion Signaling Pathway	0.26	0.01	ACVR2B,HLA-DRB1,IFNG,NFATC1,PPP2R1A
EIF2 Signaling	0.26	0.01	IGF1R,WARS1,XIAP
Heparan Sulfate Biosynthesis (Late Stages)	0.25	0.02	SULT1A1
PCP (Planar Cell Polarity) Pathway	0.25	0.02	RAC1
Apelin Endothelial Signaling Pathway	0.25	0.01	CAMK4,PRKCB
IL-2 Signaling	0.25	0.02	PTK2B
Phospholipases	0.24	0.02	HMOX1
Thrombopoietin Signaling	0.24	0.02	PRKCB
Role of PI3K/AKT Signaling in the Pathogenesis of Influenza	0.24	0.02	IFNG
WNT/Ca+ pathway	0.24	0.02	NFATC1
Endocannabinoid Cancer Inhibition Pathway	0.24	0.01	MMP2,VEGFB
NAD Signaling Pathway	0.23	0.01	NMRK1,PARP3
Corticotropin Releasing Hormone Signaling	0.23	0.01	CAMK4,PRKCB
Glutamate Receptor Signaling	0.23	0.02	CAMK4
Eicosanoid Signaling	0.23	0.02	ALOX12

Remodeling of Epithelial Adherens Junctions	0.22	0.02	ARF6
Role of JAK1 and JAK3 in γc Cytokine Signaling	0.22	0.01	PTK2B
Basal Cell Carcinoma Signaling	0.21	0.01	BMP1
TREM1 Signaling	0.20	0.01	TLR4
Ceramide Signaling	0.00	0.01	PPP2R1A
FcγRIIB Signaling in B Lymphocytes	0.00	0.01	BTK
CCR5 Signaling in Macrophages	0.00	0.01	CAMK4,FAS,PRKCB,PTK2B
Cytotoxic T Lymphocyte-mediated Apoptosis of Target Cells	0.00	0.01	B2M,DFFA,FAS
CTLA4 Signaling in Cytotoxic T Lymphocytes	0.00	0.01	B2M,PPP2R1A
T Helper Cell Differentiation	0.00	0.01	HLA-DRB1,IFNG,IFNGR1
CD28 Signaling in T Helper Cells	0.00	0.01	CAMK4,HLA-DRB1,NFATC1,RAC1
Dendritic Cell Maturation	0.00	0.01	B2M,CD1A,FCGR2A,HLA-DRB1,TLR4
Angiopoietin Signaling	0.00	0.01	DOK2
Cellular Effects of Sildenafil (Viagra)	0.00	0.01	CAMK4
Relaxin Signaling	0.00	0.01	APEX1
ICOS-ICOSL Signaling in T Helper Cells	0.00	0.01	CAMK4,HLA-DRB1,NFATC1,RAC1
Human Embryonic Stem Cell Pluripotency	0.00	0.01	BMP1,ZIC3
ATM Signaling	0.00	0.01	PPP2R1A
Role of NANOG in Mammalian Embryonic Stem Cell Pluripotency	0.00	0.01	BMP1
CREB Signaling in Neurons	0.00	0.01	CAMK4,EP300,HTR1A,IGF1R,PRKCB
Autoimmune Thyroid Disease Signaling	0.00	0.01	FAS,HLA-DRB1
Thyroid Cancer Signaling	0.00	0.01	ALK
Graft-versus-Host Disease Signaling	0.00	0.01	FAS,HLA-DRB1,IFNG
Type II Diabetes Mellitus Signaling	0.00	0.01	PPARG,PRKCB
Communication between Innate and Adaptive Immune Cells	0.00	0.01	B2M,HLA-DRB1,IFNG,TLR4
CDC42 Signaling	0.00	0.01	B2M,HLA-DRB1,ITGB3
AMPK Signaling	0.00	0.01	ACACB,EP300,PPP2R1A

Regulation of eIF4 and p70S6K Signaling	0.00	0.01	ITGB3,PPP2R1A
Role of MAPK Signaling in the Pathogenesis of Influenza	0.00	0.01	IFNG
Antiproliferative Role of TOB in T Cell Signaling	0.00	0.01	CUL1,SKP1
OX40 Signaling Pathway	0.00	0.01	B2M,HLA-DRB1
Mouse Embryonic Stem Cell Pluripotency	0.00	0.01	XIAP
Gαi Signaling	0.00	0.01	HTR1A
Gαs Signaling	0.00	0.01	HCK
Regulation of Cellular Mechanics by Calpain Protease	0.00	0.01	ITGB3
UVA-Induced MAPK Signaling	0.00	0.01	PARP3
Oxidative Phosphorylation	0.00	0.01	NDUFAB1
Unfolded protein response	0.00	0.01	PPARG
Toll-like Receptor Signaling	0.00	0.01	TLR4
SAPK/JNK Signaling	0.00	0.01	MAP3K3,NFATC1,RAC1
FGF Signaling	0.00	0.01	RAC1
GABA Receptor Signaling	0.00	0.01	GAD2
Insulin Receptor Signaling	0.00	0.01	PTPRF
Dopamine Receptor Signaling	0.00	0.01	PPP2R1A
p38 MAPK Signaling	0.00	0.01	FAS
PDGF Signaling	0.00	0.01	PRKCB
GPCR-Mediated Nutrient Sensing in Enteroendocrine Cells	0.00	0.01	PRKCB
Gustation Pathway	0.00	0.01	HTR1A,SLC5A1
Th17 Activation Pathway	0.00	0.01	HSP90AB1,IFNG,NFATC1
Endocannabinoid Neuronal Synapse Pathway	0.00	0.01	MAPK6
BAG2 Signaling Pathway	0.00	0.01	ATXN3
Regulation Of The Epithelial Mesenchymal Transition In Development Pathway	0.00	0.01	NOTCH1
Kinetochore Metaphase Signaling Pathway	0.00	0.01	NDC80

Supplementary Table S3: Disease and functional analysis by IPA for solasonine. The disease and functional analysis by IPA was performed to evaluate the correlation between solasonine and other diseases or functions. Through IPA disease and functional analysis of the potential target genes of solasonine , 500 remarkably diseases or functions annotation involved in 103 categories (p-value ≤0.05, sorted by p-value) were obtained.

No	Diseases or Functions Annotation	Categories	p-value	Molecules number	Molecules list
1	Necrosis	Cell Death and Survival	1.13E,21	107	ACVR2B,ALB,ALK,ALOX12,ANG,APEX1,APPL1,AR,ATP7B,ATXN3,B2M,BPI,BTK,BTRC,CAMK4,CD74,CDK5R1,CHKA,CSF2RB,CTCF,CUL1,CYP2E1,DAPK2,DCTD,DFFA,DPP4,DSG2,DTYMK,EP300,ERBB4,ESR1,F2,F3,FABP3,FABP7,FAP,FAS,FHIT,FLNB,FN1,G6PD,GALNT10,GART,GGPS1,GRK2,GRK6,GTF2F2,HAT1,HBB,HBEGF,HCK,HK2,HMGCS1,HMOX1,HSD17B10,HSP90AB1,IDE,IFNG,IGF1R,IQUB,ITGB3,ITPK1,ITSN1,KIF11,MMP2,MSH2,MSN,MUC1,NAE1,NCOA3,NDC80,NDUFAB1,NF2,NFATC1,NOTCH1,PA2G4,PDHA1,PDK2,PEX5,POT1,PPARG,PPP2R1A,PRKCB,PTK2B,PUS10,RAC1,RARA,RARG,RBBP6,RRM1,S100A6,SEMA4D,SFN,SKP1,SMURF2,SNX1,STK4,TLR4,TNFAIP8L2,VAV3,WEE1,XIAP,XRCC6,YARS1,YWHAH,YWHAQ,YY1
2	Apoptosis	Cell Death and Survival	2.37E,19	97	ACVR2B,ALB,ALK,ALOX12,APEX1,APPL1,AR,ARHGEF1,B2M,BPI,BTK,BTRC,CD74,CDK5R1,CHKA,CSF2RB,CTCF,CUL1,CYP2E1,DAPK2,DFFA,DPP4,DSG2,DTYMK,EP300,ERBB4,ESR1,F2,F3,FAP,FAS,FHIT,FLNB,FN1,G6PD,GALNT10,GRK2,HAT1,HBB,HBEGF,HCK,HK2,HMOX1,HSD17B10,HSP90AB1,IDE,IFNG,IGF1R,ITGB3,ITPK1,ITSN1,KIF11,KMT5A,MAGEA4,MMP2,MSH2,MSN,MUC1,NAE1,NCOA3,NDC80,NF2,NFATC1,NOTCH1,ODC1,PA2G4,PDHA1,PDK4,PEX5,POT1,PPARG,PPP2R1A,PRKCB,PTK2B,PUS10,RAC1,RARA,RARG,RBBP6,RRM1,S100A6,SEMA4D,SFN,SLK,SMURF2,SNX1,STK4,SUB1,TJP1,TLR4,VAV3,WEE1,XIAP,XRCC6,YARS1,YWHAQ,YY1
3	Cell death of tumor cell lines	Cell Death and Survival	5.24E,17	86	ACVR2B,ALB,ALK,ALOX12,ANG,APEX1,APPL1,AR,B2M,BTK,BTRC,CAMK4,CD74,CDK5R1,CHKA,CSF2RB,CTCF,CUL1,CYP2E1,DAPK2,DCTD,DFFA,DPP4,DSG2,DTYMK,EP300,ERBB4,ESR1,FABP7,FAP,FAS,FHIT,FLNB,FN1,G6PD,GALNT10,GRK2,HAT1,HBEGF,HCK,HK2,HMGCS1,HMOX1,HSP90AB1,IDE,IFNG,IGF1R,IQUB,ITGB3,ITPK1,KIF11,MSH2,MSN,MUC1,NCOA3,NDC80,NF2,NOTCH1,PA2G4,PDHA1,PDK2,PEX5,POT1,PPARG,PPP2R1A,PRKCB,PTK2B,PUS10,RAC1,RARA,RARG,RBBP6,RRM1,S100A6,SFN,SMURF2,SNX1,STK4,TLR4,VAV3,WEE1,XIAP,XRCC6,YARS1,YWHAH,YY1
4	Lymphatic system tumor	Cancer,Organismal Injury and Abnormalities	7.41E,15	104	ALB,ALK,ALOX12,ANXA6,AR,ARHGAP5,ARHGEF1,ATXN3,B2M,BCR,BTK,C1R,C8A,CD1A,CD74,CDK5R1,CIAO1,CLK1,CSDE1,CSF2RB,CSNK1G2,CSTF2,CTCF,DGKA,DPP4,ECHS1,ECI2,EP300,EPHA3,ERBB4,ESR1,F2,F3,FAP,FAS,FCGR2A,FHIT,FN1,GAD2,GART,GGPS1,GRHPR,GRK2,GTF2F2,GTF2I,HASPIN,HAT1,HCK,HDAC7,HLADRB1,HMOX1,HNRNPA2B1,HSP90AB1,IFNG,IGF1R,IMPDH1,IQUB,ITGB3,ITPK1,LRBA,MAGI2,MARK1,MMP1,MMP2,MUC1,NAE1,NCBP1,NCOA3,NDC80,NFATC1,NOTCH1,NT5C3A,NUP214,ODC1,PC,PGC,PGK1,POT1,POU2F1,PRKCB,PTK2B,PTPRE,PTPRF,PYGL,RAC1,RARA,RARG,RRM1,SEM1,SH2B2,SLC5A1,SMG5,SNX1,SYT13,TAF13,THBS2,TJP1,TLR4,TXNDC12,VAV3,XIAP,XRCC6,ZEB2,ZNF292
5	Lymphoid cancer	Cancer,Organismal Injury and Abnormalities	1.02E,14	103	ALB,ALK,ALOX12,ANXA6,ARHGAP5,ARHGEF1,ATXN3,B2M,BCR,BTK,C1R,C8A,CD1A,CD74,CDK5R1,CIAO1,CLK1,CSDE1,CSF2RB,CSNK1G2,CSTF2,CTCF,DGKA,DPP4,ECHS1,ECI2,EP300,EPHA3,ERBB4,ESR1,F2,F3,FAP,FAS,FCGR2A,FHIT,FN1,GAD2,GART,GGPS1,GRHPR,GRK2,GTF2F2,GTF2I,HASPIN,HAT1,HCK,HDAC7,HLADRB1,HMOX1,HNRNPA2B1,HSP90AB1,IFNG,IGF1R,IMPDH1,IQUB,ITGB3,ITPK1,LRBA,MAGI2,MARK1,MMP1,MMP2,MUC1,NAE1,NCBP1,NCOA3,NDC80,NFATC1,NOTCH1,NT5C3A,NUP214,ODC1,PC,PGC,PGK1,POT1,POU2F1,PRKCB,PTK2B,PTPRE,PTPRF,PYGL,RAC1,RARA,RARG,RRM1,SEM1,SH2B2,SLC5A1,SMG5,SNX1,SYT13,TAF13,THBS2,TJP1,TLR4,TXNDC12,VAV3,XIAP,XRCC6,ZEB2,ZNF292
6	Ductal breast carcinoma	Cancer,Organismal Injury and Abnormalities,Reproductive System Disease	1.20E,14	54	ACACB,AR,BTK,CAMK4,CSNK1G1,CTCF,DAPK2,DPP4,EP300,EPHA3,ERBB4,ESR1,FAP,FLNB,FN1,GRK2,GRK6,HDAC7,HSP90AB1,IGF1R,ITPKC,KIF11,KMT5A,MAPK6,MARK1,MMP2,MSH2,MSN,NCOA3,NDC80,NF2,NFATC1,NOTCH1,OXSR1,PARP3,PDK3,PDK4,PHF24,POU2F1,PPP2R1A,PRKCB,PTPRE,PTPRF,RAC1,RARA,RBBP6,RBM39,RRM1,SENP2,SLK,SMURF2,TLR4,VAV3,ZEB2
7	Advanced stage tumor	Cancer,Organismal Injury and Abnormalities	1.52E,14	54	ALB,ALK,AOC3,AR,B2M,BCR,BMP1,BTK,C1R,CD74,CSF2RB,CYP17A1,DPP4,DSG2,EP300,EPHA3,ERBB4,ESR1,F2,F3,FAS,FCGR2A,FN1,GAD2,GART,GGPS1,HCK,HLA,DRB1,HMOX1,HSP90AB1,HTR1A,IFNG,IGF1R,IMPDH1,MMP1,MMP2,MSH2,MUC1,NF2,NOTCH1,NR1I3,PARP3,PGC,PPP2R1A,PTK2B,RAC1,RARA,RARG,RRM1,SH2B2,SKP1,TLR4,VAV3,VEGFB

8	Lymphoproliferative disorder	Hematological Disease,Immunological Disease	2.30E,14	102	ALB,ALK,ALOX12,ANXA6,ARHGAP5,ARHGEF1,ATXN3,B2M,BCR,BTK,C1R,C8A,CD1A,CD74,CDK5R1,CIAO1,CLK1,CSDE1,CSF2RB,CSNK1G2,CSTF2,CTCF,DGKA,DPP4,ECHS1,ECI2,EP300,EPHA3,ERBB4,F2,F3,FAP,FAS,FCGR2A,FHIT,FN1,GAD2,GART,GGPS1,GRHPR,GRK2,GTF2F2,GTF2I,HASPIN,HAT1,HCK,HDAC7,HLADRB1,HMOX1,HNRNPA2B1,HSP90AB1,IFNG,IGF1R,IMPDH1,IQUB,ITGB3,ITPK1,LRBA,MAGI2,MARK1,MMP1,MMP2,MUC1,NAE1,NCBP1,NCOA3,NDC80,NFATC1,NOTCH1,NT5C3A,NUP214,ODC1,PC,PGC,PGK1,POT1,POU2F1,PRKCB,PTK2B,PTPRE,PTPRF,PYGL,RAC1,RARA,RARG,RRM1,SEM1,SH2B2,SLC5A1,SMG5,SNX1,SYT13,TAF13,THBS2,TJP1,TLR4,TXNDC12,VAV3,XIAP,XRCC6,ZEB2,ZNF292
9	Apoptosis of tumor cell lines	Cell Death and Survival	2.70E,14	70	ACVR2B,ALB,ALK,ALOX12,AR,B2M,BTK,BTRC,CD74,CDK5R1,CHKA,CSF2RB,CTCF,CYP2E1,DAPK2,DFFA,DPP4,DSG2,DTYMK,EP300,ERBB4,ESR1,FAP,FAS,FHIT,FLNB,FN1,G6PD,GALNT10,GRK2,HAT1,HBEGF,HCK,HK2,HMOX1,HSP90AB1,IDE,IFNG,IGF1R,ITGB3,ITPK1,KIF11,MSH2,MSN,MUC1,NCOA3,NDC80,NOTCH1,PA2G4,PDHA1,PEX5,POT1,PPARG,PRKCB,PTK2B,PUS10,RAC1,RARG,RBBP6,RRM1,S100A6,SFN,SMURF2,SNX1,STK4,TLR4,WEE1,XIAP,XRCC6,YY1
10	Breast adenocarcinoma	Cancer,Organismal Injury and Abnormalities,Reproductive System Disease	2.93E,14	55	ACACB,AR,BTK,CAMK4,CSNK1G1,CTCF,DAPK2,DPP4,EP300,EPHA3,ERBB4,ESR1,FAP,FLNB,FN1,GGPS1,GRK2,GRK6,HDAC7,HSP90AB1,IGF1R,ITPKC,KIF11,KMT5A,MAPK6,MARK1,MMP2,MSH2,MSN,NCOA3,NDC80,NF2,NFATC1,NOTCH1,OXSR1,PARP3,PDK3,PDK4,PHF24,POU2F1,PPP2R1A,PRKCB,PTPRE,PTPRF,RAC1,RARA,RBBP6,RBM39,RRM1,SENP2,SLK,SMURF2,TLR4,VAV3,ZEB2
11	Lymphoma	Cancer,Hematological Disease,Immunological Disease,Organismal Injury and Abnormalities	3.30E,14	72	ALK,ANXA6,ARHGEF1,ATXN3,B2M,BCR,BTK,C1R,CD1A,CLK1,CSDE1,CSF2RB,CSNK1G2,CSTF2,CTCF,DGKA,DPP4,ECI2,EP300,EPHA3,ERBB4,FAP,FAS,FCGR2A,FHIT,FN1,GAD2,GART,GGPS1,GTF2I,HAT1,HCK,HDAC7,HMOX1,HNRNPA2B1,HSP90AB1,IFNG,IMPDH1,IQUB,ITGB3,MMP1,MMP2,MUC1,NCBP1,NFATC1,NOTCH1,NT5C3A,NUP214,ODC1,PC,PGK1,POT1,POU2F1,PRKCB,PTK2B,PTPRE,PTPRF,RAC1,RARA,RARG,RRM1,SEM1,SLC5A1,SNX1,THBS2,TJP1,TLR4,TXNDC12,VAV3,XIAP,XRCC6,ZNF292
12	Lymphocytic cancer	Cancer,Hematological Disease,Immunological Disease,Organismal Injury and Abnormalities	5.17E,14	101	ALB,ALK,ALOX12,ANXA6,ARHGAP5,ARHGEF1,ATXN3,B2M,BCR,BTK,C1R,C8A,CD1A,CD74,CDK5R1,CIAO1,CLK1,CSDE1,CSF2RB,CSNK1G2,CSTF2,CTCF,DGKA,DPP4,ECHS1,ECI2,EP300,EPHA3,ERBB4,F2,F3,FAP,FAS,FCGR2A,FHIT,FN1,GAD2,GART,GGPS1,GRHPR,GRK2,GTF2F2,GTF2I,HASPIN,HAT1,HCK,HDAC7,HLADRB1,HMOX1,HNRNPA2B1,HSP90AB1,IFNG,IMPDH1,IQUB,ITGB3,ITPK1,LRBA,MAGI2,MARK1,MMP1,MMP2,MUC1,NAE1,NCBP1,NCOA3,NDC80,NFATC1,NOTCH1,NT5C3A,NUP214,ODC1,PC,PGC,PGK1,POT1,POU2F1,PRKCB,PTK2B,PTPRE,PTPRF,PYGL,RAC1,RARA,RARG,RRM1,SEM1,SH2B2,SLC5A1,SMG5,SNX1,SYT13,TAF13,THBS2,TJP1,TLR4,TXNDC12,VAV3,XIAP,XRCC6,ZEB2,ZNF292
13	Advanced malignant tumor	Cancer,Organismal Injury and Abnormalities	5.18E,14	53	ALB,ALK,AOC3,AR,B2M,BCR,BTK,C1R,CD74,CSF2RB,CYP17A1,DPP4,DSG2,EP300,EPHA3,ERBB4,ESR1,F2,F3,FAS,FCGR2A,FN1,GAD2,GART,GGPS1,HCK,HLADRB1,HMOX1,HSP90AB1,HTR1A,IFNG,IGF1R,IMPDH1,MMP1,MMP2,MSH2,MUC1,NF2,NOTCH1,NR1I3,PARP3,PGC,PPP2R1A,PTK2B,RAC1,RARA,RARG,RRM1,SH2B2,SKP1,TLR4,VAV3,VEGFB
14	Lymphocytic neoplasm	Cancer,Hematological Disease,Organismal Injury and Abnormalities	5.56E,14	101	ALB,ALK,ALOX12,ANXA6,ARHGAP5,ARHGEF1,ATXN3,B2M,BCR,BTK,C1R,C8A,CD1A,CD74,CDK5R1,CIAO1,CLK1,CSDE1,CSF2RB,CSNK1G2,CSTF2,CTCF,DGKA,DPP4,ECHS1,ECI2,EP300,EPHA3,ERBB4,F2,F3,FAP,FAS,FCGR2A,FHIT,FN1,GAD2,GART,GGPS1,GRHPR,GRK2,GTF2F2,GTF2I,HASPIN,HAT1,HCK,HDAC7,HLADRB1,HMOX1,HNRNPA2B1,HSP90AB1,IFNG,IMPDH1,IQUB,ITGB3,ITPK1,LRBA,MAGI2,MARK1,MMP1,MMP2,MUC1,NAE1,NCBP1,NCOA3,NDC80,NFATC1,NOTCH1,NT5C3A,NUP214,ODC1,PC,PGC,PGK1,POT1,POU2F1,PRKCB,PTK2B,PTPRE,PTPRF,PYGL,RAC1,RARA,RARG,RRM1,SEM1,SH2B2,SLC5A1,SMG5,SNX1,SYT13,TAF13,THBS2,TJP1,TLR4,TXNDC12,VAV3,XIAP,XRCC6,ZEB2,ZNF292
15	Hematologic cancer of cells	Cancer,Hematological Disease,Organismal Injury and Abnormalities	5.71E,14	94	ALB,ALK,ALOX12,ANXA6,ARHGAP5,ARHGEF1,ATXN3,B2M,BCR,BTK,C1R,C8A,CD1A,CD74,CDK5R1,CIAO1,CLK1,CSDE1,CSF2RB,CSNK1G2,CSTF2,CTCF,DGKA,DPP4,ECI2,EP300,EPHA3,ERBB4,F2,F3,FAP,FAS,FCGR2A,FHIT,FN1,GAD2,GART,GGPS1,GRK2,GTF2F2,GTF2I,HASPIN,HAT1,HCK,HDAC7,HMOX1,HNRNPA2B1,HSP90AB1,IFNG,IMPDH1,IQUB,ITGB3,LRBA,MAGI2,MMP1,MMP2,MUC1,NCBP1,NCOA3,NFATC1,NOTCH1,NT5C3A,NUP214,ODC1,PC,PGC,PGK1,POT1,POU2F1,PRKCB,PTK2B,PTPRE,PTPRF,PYGL,RAC1,RARA,RARG,RRM1,SEM1,SH2B2,SLC5A1,SMG5,SNX1,SYT13,TAF13,THBS2,TJP1,TLR4,TXNDC12,VAV3,XIAP,XRCC6,ZEB2,ZNF292
16	Neoplasia of leukocytes	Cancer,Hematological Disease,Immunological	5.76E,14	99	ALB,ALK,ALOX12,ANXA6,ARHGAP5,ARHGEF1,ATXN3,B2M,BCR,BTK,C1R,C8A,CD1A,CD74,CDK5R1,CIAO1,CLK1,CSDE1,CSF2RB,CSNK1G2,CSTF2,CTCF,DGKA,DPP4,ECI2,EP300,EPHA3,ERBB4,F2,F3,FAP,FAS,FCGR2A,FHIT,FN1,GAD2,GART,GGPS1,GRHPR,GRK2,GTF2F2,GTF2I,HASPIN,HAT1,HCK,HDAC7,HLA,DR

		Disease,Organismal Injury and Abnormalities			B1,HMOX1,HNRNPA2B1,HSP90AB1,IFNG,IMPDH1,IQUB,ITGB3,ITPK1,LRBA,MAGI2,MMP1,MMP2,MUC1,NAE1,NCBP1,NCOA3,NDC80,NFATC1,NOTCH1,NT5C3A,NUP214,ODC1,PC,PGC,PGK1,POT1,POU2F1,PRKCB,PTK2B,PTPRE,PTPRF,PYGL,RAC1,RARA,RARG,RRM1,SEM1,SH2B2,SLC5A1,SMG5,SNX1,SYT13,TAF13,THBS2,TJP1,TLR4,TXNDC12,VAV3,XIAP,XRCC6,ZEB2,ZNF292
17	Tumorigenesis of lymphocytes	Cancer,Hematological Disease,Immunological Disease,Organismal Injury and Abnormalities	5.98E,14	96	ALB,ALK,ALOX12,ANXA6,ARHGAP5,ARHGEF1,ATXN3,B2M,BCR,BTK,C1R,C8A,CD1A,CD74,CDK5R1,CIAO1,CLK1,CSDE1,CSF2RB,CSNK1G2,CSTF2,CTCF,DGKA,DPP4,ECI2,EP300,EPHA3,ERBB4,FAP,FAS,FCGR2A,FHIT,FN1,GAD2,GART,GGPS1,GRHPR,GRK2,GTF2F2,GTF2I,HASPIN,HAT1,HCK,HDAC7,HLA,DRB1,HMOX1,HNRNPA2B1,HSP90AB1,IFNG,IMPDH1,IQUB,ITGB3,ITPK1,LRBA,MAGI2,MMP1,MMP2,MUC1,NAE1,NCBP1,NDC80,NFATC1,NOTCH1,NT5C3A,NUP214,ODC1,PC,PGC,PGK1,POT1,POU2F1,PRKCB,PTK2B,PTPRE,PTPRF,PYGL,RAC1,RARA,RARG,RRM1,SEM1,SH2B2,SLC5A1,SMG5,SNX1,SYT13,TAF13,THBS2,TJP1,TLR4,TXNDC12,VAV3,XIAP,XRCC6,ZEB2,ZNF292
18	Viral Infection	Infectious Diseases	1.49E,13	84	ACACB,ACADSB,ACP3,ADH1C,ADH7,ALB,ALOX12,ANXA6,AR,ARF6,B2M,BCR,BMP1,BPI,BTRC,C1R,C8A,CANT1,CD74,CLK1,CSF2RB,CTCF,CYP17A1,CYP2E1,DAPK2,DHPS,DPP4,DSG2,EP300,ESR1,ETF1,F2,F3,FAS,FCGR2A,FN1,G6PD,GGPS1,GRK2,GRK6,GTF2I,HBB,HCK,HIBCH,HLA,DRB1,HMGCS1,IFNG,IFNGR1,IGF1R,IMPA2,IMPDH1,IQUB,ITGB3,KIF11,LARS1,MDH1,MSH2,NBEA,NCOA3,NF2,NFATC1,NMT1,NUP214,OXSR1,PANK1,PPARG,PRKCB,PTK2B,PYGL,RAC1,RARA,RARG,RND1,RRM1,SART3,SERPINA5,SF3B6,SUB1,TLR4,TRIM21,WWP1,XIAP,ZEB2,ZNF292
19	Non,Hodgkin lymphoma	Cancer,Hematological Disease,Immunological Disease,Organismal Injury and Abnormalities	2.81E,13	67	ALK,ANXA6,ARHGEF1,ATXN3,B2M,BCR,BTK,C1R,CD1A,CLK1,CSDE1,CSF2RB,CSTF2,CTCF,DGKA,DPP4,ECI2,EP300,EPHA3,ERBB4,FAS,FCGR2A,FHIT,FN1,GGPS1,GTF2I,HAT1,HCK,HDAC7,HMOX1,HNRNPA2B1,HSP90AB1,IFNG,IMPDH1,IQUB,ITGB3,MMP1,MMP2,MUC1,NCBP1,NFATC1,NOTCH1,NT5C3A,ODC1,PC,PGK1,POT1,POU2F1,PRKCB,PTK2B,PTPRE,PTPRF,RAC1,RARA,RARG,RRM1,SEM1,SLC5A1,SNX1,THBS2,TJP1,TLR4,TXNDC12,VAV3,XIAP,XRCC6,ZNF292
20	Myeloid or lymphoid neoplasm	Cancer,Hematological Disease,Organismal Injury and Abnormalities	3.58E,13	126	ALB,ALK,ALOX12,AMY1C (includes others),ANXA6,APEX1,ARHGAP5,ARHGEF1,ATXN3,B2M,BCR,BMP1,BTK,BTRC,C1R,C8A,CANT1,CD1A,CD74,CDK5R1,CIAO1,CLK1,CSDE1,CSF2RB,CSNK1G2,CSTF2,CTCF,CYP2E1,CYP2R1,DGKA,DPP4,ECHS1,ECI2,EP300,EPHA3,ERBB4,ESR1,ETF1,F2,F3,FAP,FAS,FCGR2A,FHIT,FN1,GAD2,GART,GGPS1,GRHPR,GRK2,GTF2F2,GTF2I,HASPIN,HAT1,HCK,HDAC7,HLA,DRB1,HMGB3,HMOX1,HNRNPA2B1,HSP90AB1,IFNG,IGF1R,IMPDH1,IQUB,ITGB3,ITPK1,KLK1,KMT5A,LRBA,MAGEA4,MAGI2,MARK1,MMP1,MMP2,MSH2,MUC1,NAE1,NCBP1,NCOA3,NDC80,NFATC1,NOTCH1,NT5C3A,NUP214,ODC1,PA2G4,PC,PDHA1,PDK3,PDK4,PGC,PGK1,POT1,POU2F1,PRKCB,PTK2B,PTPRE,PTPRF,PYGL,RAC1,RARA,RARG,RRM1,SEM1,SH2B2,SLC5A1,SMG5,SMG7,SMTN,SNX1,STK4,SYT13,TAF13,THBS2,TJP1,TLR4,TXNDC12,UEVLD,VAV3,VEGFB,WEE1,XIAP,XRCC6,ZEB2,ZNF292
21	Neoplasia of blood cells	Cancer,Hematological Disease,Organismal Injury and Abnormalities	4.88E,13	124	ALB,ALK,ALOX12,AMY1C (includes others),ANXA6,APEX1,ARHGAP5,ARHGEF1,ATXN3,B2M,BCR,BMP1,BTK,BTRC,C1R,C8A,CANT1,CD1A,CD74,CDK5R1,CIAO1,CLK1,CSDE1,CSF2RB,CSNK1G2,CSTF2,CTCF,CYP2E1,CYP2R1,DGKA,DPP4,ECI2,EP300,EPHA3,ERBB4,ESR1,ETF1,F2,F3,FAP,FAS,FCGR2A,FHIT,FN1,GAD2,GART,GGPS1,GRHPR,GRK2,GTF2F2,GTF2I,HASPIN,HAT1,HCK,HDAC7,HLA,DRB1,HMGB3,HMOX1,HNRNPA2B1,HSP90AB1,IFNG,IGF1R,IMPDH1,IQUB,ITGB3,ITPK1,KLK1,KMT5A,LRBA,MAGEA4,MAGI2,MMP1,MMP2,MSH2,MUC1,NAE1,NCBP1,NCOA3,NDC80,NFATC1,NOTCH1,NT5C3A,NUP214,ODC1,PA2G4,PC,PDHA1,PDK3,PDK4,PGC,PGK1,POT1,POU2F1,PRKCB,PTK2B,PTPRE,PTPRF,PYGL,RAC1,RARA,RARG,RRM1,SEM1,SH2B2,SLC5A1,SMG5,SMG7,SMTN,SNX1,STK4,SYT13,TAF13,THBS2,TJP1,TLR4,TXNDC12,UEVLD,VAV3,VEGFB,WEE1,XIAP,XRCC6,ZEB2,ZNF292
22	Cell viability	Cell Death and Survival	9.55E,13	65	ACVR2B,ALB,ALK,ALOX12,APEX1,AR,ARF6,ATP7B,BCR,BTK,BTRC,CAMK4,CD74,CHKA,DGKA,DOK2,DPP4,DTYMK,EP300,ERBB4,ESR1,F2,FAS,FHIT,FN1,GRK2,GRK6,HBB,HBEGF,HCK,HK2,HMGCS1,HMOX1,HSP90AB1,IFNG,IGF1R,ITGB3,KIF11,MSH2,NAE1,NDC80,NME3,NOTCH1,NT5C3A,PDHA1,PPARG,PPP2R1A,PRKCB,PTK2B,PTPRE,RAC1,RBM39,RRM1,RUVBL1,S100A6,SEM1,SEMA4D,SFN,SKP1,THBS2,TLR4,TRIM21,WEE1,XIAP,XRCC6
23	Cell survival	Cell Death and Survival	1.07E,12	66	ACVR2B,ALB,ALK,ALOX12,APEX1,AR,ARF6,ATP7B,BCR,BTK,BTRC,CAMK4,CD74,CHKA,CSF2RB,DGKA,DOK2,DPP4,DTYMK,EP300,ERBB4,ESR1,F2,FAS,FHIT,FN1,GRK2,GRK6,HBB,HBEGF,HCK,HK2,HMGCS1,H

					MOX1,HSP90AB1,IFNG,IGF1R,ITGB3,KIF11,MSH2,NAE1,NDC80,NME3,NOTCH1,NT5C3A,PDHA1,PPARG,PPP2R1A,PRKCB,PTK2B,PTPRE,RAC1,RBM39,RRM1,RUVBL1,S100A6,SEM1,SEMA4D,SFN,SKP1,THBS2,TLR4,TRIM21,WEE1,XIAP,XRCC6
24	Hematologic cancer	Cancer,Hematological Disease,Organismal Injury and Abnormalities	1.11E,12	124	ALB,ALK,ALOX12,AMY1C (includes others),ANXA6,APEX1,ARHGAP5,ARHGEF1,ATXN3,B2M,BCR,BTK,BTRC,C1R,C8A,CANT1,CD1A,CD74,CDK5R1,CIAO1,CLK1,CSDE1,CSF2RB,CSNK1G2,CSTF2,CTCF,CYP2E1,CYP2R1,DGKA,DPP4,ECHS1,ECI2,EP300,EPHA3,ERBB4,ESR1,ETF1,F2,F3,FAP,FAS,FCGR2A,FHIT,FN1,GAD2,GART,GGPS1,GRHPR,GRK2,GTF2F2,GTF2I,HASPIN,HAT1,HCK,HDAC7,HLA,DRB1,HMOX1,HNRNPA2B1,HSP90AB1,IFNG,IGF1R,IMPDH1,IQUB,ITGB3,ITPK1,KLK1,KMT5A,LRBA,MAGEA4,MAGI2,MARK1,MMP1,MMP2,MSH2,MUC1,NAE1,NCBP1,NCOA3,NDC80,NFATC1,NOTCH1,NT5C3A,NUP214,ODC1,PA2G4,PC,PDHA1,PDK3,PDK4,PGC,PGK1,POT1,POU2F1,PRKCB,PTK2B,PTPRE,PTPRF,PYGL,RAC1,RARA,RARG,RRM1,SEM1,SH2B2,SLC5A1,SMG5,SMG7,SMTN,SNX1,STK4,SYT13,TAF13,THBS2,TJP1,TLR4,TXNDC12,UEVLD,VAV3,VEGFB,WEE1,XIAP,XRCC6,ZEB2,ZNF292
25	Phosphorylation of protein	Post,Translational Modification	1.35E,12	34	ALK,ANG,AR,BCR,BTK,CAMK4,CD74,CSNK1G2,DAPK2,ERBB4,F2,FAS,FCGR2A,FN1,HASPIN,HCK,HLA,DRB1,IFNG,IGF1R,ITGB3,MAP3K3,MARK1,MATK,OXSR1,PRKCB,PSAP,PTK2B,RAC1,RARA,SEMA4D,SLK,STK16,STK4,WARS1
26	Cell proliferation of tumor cell lines	Cellular Development,Cellular Growth and Proliferation	1.91E,12	85	AASDHPPT,ACP3,ALK,ANG,ANXA6,APEX1,AR,BTK,CD74,CDK5R1,CHKA,CSF2RB,CSTF2,CTCF,DAPK2,DOK2,DPP4,DSG2,DTYMK,ECHS1,EP300,ERBB4,ESR1,F2,F3,FAS,FHIT,FN1,G6PD,GALNT10,HASPIN,HAT1,HBEGF,HCK,HK2,HMGCS1,HMOX1,HNRNPA2B1,IDE,IFNG,IGF1R,ITGB3,MAPK6,MATK,MEMO1,MMP1,MMP2,MTAP,MUC1,NCOA3,NF2,NFATC1,NOTCH1,NT5C3A,PA2G4,PC,PDK4,PGK1,PPARG,PPP2R1A,PRKCB,PTK2B,PTPRE,PTPRF,RAC1,RARA,RARG,RBBP6,RRM1,S100A6,SEMA4D,SFN,SMURF2,SNX1,STARD13,STK4,TLR4,TRIM21,UTRN,VAV3,XIAP,XRCC6,YWHAQ,YY1,ZEB2
27	Metastasis	Cancer,Organismal Injury and Abnormalities	1.97E,12	46	ALB,ALK,AOC3,AR,B2M,BTK,C1R,CSF2RB,CYP17A1,DSG2,EP300,EPHA3,ERBB4,ESR1,F2,F3,FAS,FCGR2A,FN1,GAD2,GART,GGPS1,HLADRB1,HMOX1,HSP90AB1,HTR1A,IGF1R,IMPDH1,MMP1,MMP2,MSH2,MUC1,NF2,NOTCH1,NR1I3,PARP3,PGC,PPP2R1A,PTK2B,RAC1,RARA,RARG,RRM1,SH2B2,TLR4,VEGFB
28	Head and neck cancer	Cancer,Organismal Injury and Abnormalities	1.99E,12	256	AASDHPPT,ACACB,ACADSB,ACADVL,ACAT2,ACBD7,ACVR2B,ACY1,ADH7,ALB,ALK,ALOX12,AMY1C (includesothers),ANXA6,AOC3,APEX1,APPL1,AR,ARF6,ARHGAP5,ARHGEF1,ASAP1,ATP7B,ATXN3,B2M,B3GAT2,B3GAT3,BCR,BMP1,BPI,BTK,BTRC,C1R,C8A,CA8,CANT1,CD1A,CD74,CHKA,CIAO1,CLCN5,CLK1,CRYM,CSDE1,CSF2RB,CSNK1G1,CSNK1G2,CSTF2,CTCF,CUL1,CYP17A1,CYP2E1,CYP2R1,CYP7A1,DAPK2,DCTD,DCXR,DFFA,DGKA,DHPS,DMC1,DOK2,DPP4,DSG2,DTYMK,EP300,EPHA3,EPS8L2,ERBB4,ERI1,ESR1,ETF1,F2,F3,FAP,FAS,FCGR2A,FHIT,FLNB,FN1,G6PD,GAD2,GALM,GALNT10,GART,GC,GRHPR,GRK2,GRK6,GSTT2/GSTT2B,GTF2F2,GTF2I,HADH,HASPIN,HAT1,HBEGF,HCK,HDAC7,HDX,HIBCH,HK2,HLADRB1,HMBOX1,HMGB3,HMGCS1,HMOX1,HNRNPA2B1,HPD,HSCB,HSD17B4,HSP90AB1,HTR1A,IDE,IFNGR1,IGF1R,IMPA2,IMPDH1,IQUB,ITGB3,ITPK1,ITPKC,ITSN1,KIF11,KLK1,KMT5A,KYNU,LRBA,MAGEA4,MAGI2,MAP3K3,MAPK6,MARF1,MARK1,MATK,MECR,MEMO1,MMP1,MMP2,MSH2,MSN,MTAP,MUC1,MYBPC1,MYBPC3,MYSM1,NAE1,NBEA,NCBP1,NCOA3,NDC80,NDUFAB1,NF2,NFATC1,NME3,NMRK1,NOTCH1,NR1I3,NT5C3A,NUP214,OAT,ODC1,OXSR1,PA2G4,PAFAH1B2,PAH,PANK1,PAPSS1,PARN,PARP3,PATJ,PC,PCYT2,PDF,PDHA1,PDK2,PDK3,PDK4,PEX5,PGK1,PHF24,PIP4K2C,PNMT,POT1,POU2F1,PPARG,PPP2R1A,PRKCB,PSAP,PTK2B,PTPRE,PTPRF,PYGL,RAB26,RAC1,RARA,RARG,RBBP6,RBM39,RGS6,RND1,RPA3,RRM1,RTN4IP1,RUVBL1,SART3,SEC23A,SEM1,SEMA4D,SENP2,SERPINA5,SFMBT2,SFN,SH2B2,SH3YL1,SKP1,SLC5A1,SLK,SMAP1,SMG5,SMG7,SMTN,SMURF2,SNX1,SNX22,SPEF1,STARD13,STK4,SUB1,SULT1A1,SYT13,TAF13,THBS2,TIMM9,TJP1,TLR4,TRIM21,TTR,UEVLD,USP19,UTRN,VAV3,VEGFB,VPS26A,WEE1,WHRN,WWP1,XRCC6,YWHAQ,YY1,ZBTB43,ZCWPW1,ZEB2,ZFP28,ZIC3,ZNF292,ZNF462
29	Dermatitis	Dermatological Diseases and Conditions,Inflammatory Disease,Inflammatory Response,Organismal Injury and Abnormalities	1.99E,12	29	ACADVL,CD1A,CD74,CSF2RB,CYP17A1,DHPS,DPP4,GRK2,HBB,HCK,HLA,DRB1,HTR1A,IDE,IFNG,KLK1,MMP1,MSN,ODC1,PDK4,PPARG,PRKCB,PSAP,RARA,RARG,RRM1,RUVBL1,SFN,TLR4,XRCC6

30	Inflammation of organ	Inflammatory Response,Organismal Injury and Abnormalities	2.00E,12	51	ACADVL,ALB,ALK,ALOX12,AR,CD1A,CD74,CSF2RB,CYP17A1,CYP2E1,DHPS,DPP4,EP300,ESR1,F2,F3,FAS,FCGR2A,FN1,GAD2,GC,GRK2,HBB,HCK,HLADRB1,HTR1A,IDE,IFNG,IFNGR1,IGF1R,IMPDH1,ITGB3,KLK1,MMP1,MSN,MUC1,ODC1,PDK4,PPARG,PRKCB,PSAP,RAC1,RARA,RARG,RRM1,RUVBL1,SFN,SMURF2,TAF13,TLR4,XRCC6
31	Advanced malignant solid tumor	Cancer,Organismal Injury and Abnormalities	2.27E,12	45	ALB,ALK,AOC3,AR,B2M,BCR,BTK,C1R,CSF2RB,CYP17A1,DPP4,DSG2,EP300,EPHA3,ERBB4,ESR1,F2,F3,FAS,FCGR2A,FN1,GAD2,GART,GGPS1,HMOX1,HSP90AB1,IGF1R,IMPDH1,MMP2,MSH2,MUC1,NF2,NOTCH1,NR1I3,PARP3,PGC,PPP2R1A,PTK2B,RAC1,RARA,RARG,RRM1,SKP1,VAV3,VEGFB
32	Cell death of tumor cells	Cancer,Cell Death and Survival,Organismal Injury and Abnormalities,Tumor Morphology	2.98E,12	22	ALB,AR,ATP7B,B2M,BTK,CD74,F2,FAS,FHIT,GRK6,HBEGF,HMOX1,IFNG,IGF1R,MUC1,NCOA3,NOTCH1,PPARG,PTK2B,RAC1,RARA,XIAP
33	Invasive tumor	Cancer,Organismal Injury and Abnormalities	3.55E,12	54	ALB,ALK,AOC3,AR,B2M,BTK,C1R,CSF2RB,CYP17A1,DSG2,EP300,EPHA3,ERBB4,ESR1,F2,F3,FAP,FAS,FCGR2A,FN1,GAD2,GART,GGPS1,HLA,DRB1,HMOX1,HSP90AB1,HTR1A,IGF1R,IMPDH1,MMP1,MMP2,MSH2,MUC1,MYBPC3,NF2,NOTCH1,NR1I3,PARP3,PDK4,PGC,PPP2R1A,PTK2B,PTPRE,PTPRF,RAC1,RARA,RARG,RBM39,RRM1,SH2B2,THBS2,TLR4,VEGFB,YWHAQ
34	Mature lymphocytic neoplasm	Cancer,Hematological Disease,Organismal Injury and Abnormalities	3.94E,12	79	ALK,ALOX12,ANXA6,ARHGEF1,ATXN3,B2M,BCR,BTK,C1R,CD1A,CD74,CLK1,CSDE1,CSF2RB,CSTF2,CTCF,DGKA,DPP4,ECI2,EP300,EPHA3,ERBB4,FAS,FCGR2A,FHIT,FN1,GAD2,GART,GGPS1,GRHPR,GTF2F2,GTF2I,HAT1,HCK,HDAC7,HLA,DRB1,HMOX1,HNRNPA2B1,HSP90AB1,IFNG,IMPDH1,IQUB,ITGB3,ITPK1,MAGI2,MMP1,MMP2,MUC1,NAE1,NCBP1,NDC80,NFATC1,NOTCH1,NT5C3A,ODC1,PC,PGK1,POT1,POU2F1,PRKCB,PTK2B,PTPRE,PTPRF,RAC1,RARA,RARG,RRM1,SEM1,SLC5A1,SNX1,TAF13,THBS2,TJP1,TLR4,TXNDC12,VAV3,XIAP,XRCC6,ZNF292
35	Neck neoplasm	Cancer,Organismal Injury and Abnormalities	4.43E,12	243	AASDHPPT,ACACB,ACADSB,ACADVL,ACAT2,ACBD7,ACVR2B,ACY1,ADH7,ALB,ALK,ALOX12,AMY1C (includesothers),ANXA6,AOC3,APPL1,AR,ARHGAP5,ARHGEF1,ASAP1,ATP7B,ATXN3,B3GAT2,B3GAT3,BCR,BMP1,BPI,BTK,BTRC,C8A,CA8,CANT1,CD1A,CHKA,CIAO1,CLCN5,CLK1,CRYM,CSDE1,CSF2RB,CSNK1G1,CSNK1G2,CSTF2,CTCF,CUL1,CYP17A1,CYP2E1,CYP2R1,CYP7A1,DAPK2,DCTD,DCXR,DFFA,DGKA,DHPS,DMC1,DOK2,DPP4,DSG2,DTYMK,EP300,EPHA3,EPS8L2,ERBB4,ERI1,ESR1,ETF1,F2,F3,FAP,FAS,FLNB,FN1,G6PD,GAD2,GALM,GALNT10,GART,GC,GRHPR,GRK2,GRK6,GSTT2/GSTT2B,GTF2F2,GTF2I,HADH,HASPIN,HAT1,HBEGF,HCK,HDAC7,HDX,HIBCH,HK2,HLADRB1,HMBOX1,HMGB3,HMGCS1,HMOX1,HNRNPA2B1,HPD,HSCB,HSD17B4,HSP90AB1,IDE,IFNGR1,IGF1R,IMPA2,IMPDH1,IQUB,ITGB3,ITPK1,ITPKC,ITSN1,KIF11,KLK1,KMT5A,KYNU,LRBA,MAGI2,MAP3K3,MAPK6,MARF1,MARK1,MATK,MECR,MEMO1,MMP1,MMP2,MSH2,MSN,MTAP,MUC1,MYBPC1,MYBPC3,MYSM1,NAE1,NBEA,NCBP1,NCOA3,NDC80,NDUFAB1,NF2,NFATC1,NME3,NMRK1,NOTCH1,NR1I3,NT5C3A,NUP214,OAT,ODC1,OXSR1,PA2G4,PAFAH1B2,PAH,PANK1,PAPSS1,PARN,PARP3,PATJ,PC,PCYT2,PDF,PDHA1,PDK2,PDK3,PDK4,PEX5,PGK1,PHF24,PIP4K2C,PNMT,POT1,POU2F1,PPARG,PPP2R1A,PRKCB,PSAP,PTK2B,PTPRE,PTPRF,PYGL,RAB26,RAC1,RARA,RARG,RBBP6,RBM39,RGS6,RND1,RPA3,RRM1,RTN4IP1,RUVBL1,SART3,SEC23A,SEM1,SEMA4D,SENP2,SERPINA5,SFMBT2,SH2B2,SH3YL1,SKP1,SLC5A1,SLK,SMAP1,SMG5,SMTN,SMURF2,SNX1,SNX22,SPEF1,STARD13,STK4,SUB1,SULT1A1,SYT13,TAF13,THBS2,TJP1,TLR4,TRIM21,TTR,UEVLD,USP19,UTRN,VAV3,VPS26A,WEE1,WHRN,WWP1,XRCC6,YWHAQ,YY1,ZBTB43,ZCWPW1,ZEB2,ZFP28,ZIC3,ZNF292,ZNF462
36	Non,melanoma solid tumor	Cancer,Organismal Injury and Abnormalities	4.52E,12	285	AASDHPPT,ACACB,ACADSB,ACADVL,ACAT2,ACBD7,ACSM2A,ACVR2B,ACY1,ADH1C,ADH7,ALB,ALK,ALOX12,AMY1C (includes others),ANG,ANXA6,AOC3,APEX1,APPL1,AR,ARF6,ARHGAP5,ARHGEF1,ASAP1,ATP7B,ATXN3,B2M,B3GAT2,B3GAT3,BCR,BMP1,BPI,BTK,BTRC,C1R,C8A,CA8,CAMK4,CANT1,CD1A,CD74,CDK5R1,CERT1,CHKA,CIAO1,CLCN5,CLK1,CRYM,CSDE1,CSF2RB,CSNK1G1,CSNK1G2,CSTF2,CTCF,CUL1,CYP17A1,CYP2E1,CYP2R1,CYP7A1,DAPK2,DCTD,DCXR,DFFA,DGKA,DHPS,DMC1,DOK2,DPP4,DSG2,DTYMK,ECHS1,ECI2,EP300,EPHA3,EPS8L2,ERBB4,ERI1,ESR1,ETF1,EXOSC9,F2,F3,FABP3,FABP7,FAP,FAS,FCGR2A,FHIT,FLNB,FN1,G6PD,GAD2,GALM,GALNT10,GART,GC,GGPS1,GRHPR,GRK2,GRK6,GSTT2/GSTT2B,GTF2F2,GTF2I,HADH,HASPIN,HAT1,HBB,HBEGF,HCK,HDAC7,HDX,HIBCH,HK2,HLA,DRB1,HMBOX1,HMGB3,HMGCS1,HMOX1,HNRNPA2B1,HPD,HSCB,HSD17B10,HSD17B4,HSP90AB1,HTR1A,IDE,IFNG,IFNGR1,IGF1R,IMPA2,IMPDH1

					,IQUB,ITGB3,ITPA,ITPK1,ITPKC,ITSN1,KIF11,KLK1,KMT5A,KYNU,LRBA,MAGEA4,MAGI2,MAP3K3,MAPK6,MARF1,MARK1,MATK,MDH1,MECR,MEMO1,MMP1,MMP2,MSH2,MSN,MTAP,MUC1,MYBPC1,MYBPC3,MYSM1,NAE1,NBEA,NCBP1,NCOA3,NDC80,NDUFAB1,NF2,NFATC1,NME3,NMRK1,NMT1,NOTCH1,NR1I3,NSUN5,NT5C3A,NUP214,OAT,ODC1,OXSR1,PA2G4,PAFAH1B2,PAH,PANK1,PAPSS1,PARN,PARP3,PATJ,PC,PCYT2,PDF,PDHA1,PDK2,PDK3,PDK4,PEX5,PGC,PGK1,PHF24,PIP4K2C,PNMT,POT1,POU2F1,PPARG,PPP2R1A,PRKCB,PSAP,PTK2B,PTPRE,PTPRF,PUS10,PYGL,RAB26,RAC1,RARA,RARG,RBBP6,RBM39,RBP5,RGS6,RND1,RPA3,RRM1,RTN4IP1,RUVBL1,S100A6,SART3,SEC23A,SEM1,SEMA4D,SENP2,SERPINA5,SF3B6,SFMBT2,SFN,SH2B2,SH3YL1,SKP1,SLC5A1,SLK,SMAP1,SMG5,SMG7,SMTN,SMURF2,SNX1,SNX22,SPEF1,STARD13,STK16,STK4,SUB1,SULT1A1,SYT13,TAF13,THBS2,TIMM9,TJP1,TLR4,TNFAIP8L2,TRIM21,TTR,TXNDC12,UEVLD,USP19,UTRN,VAV3,VEGFB,VPS26A,WEE1,WHRN,WWP1,XIAP,XRCC6,YWHAH,YWHAQ,YY1,ZBTB43,ZCWPW1,ZEB2,ZFP28,ZIC3,ZNF292,ZNF462
37	Neoplasia of cells	Cancer,Organismal Injury and Abnormalities	4.70E,12	178	ALB,ALK,ALOX12,AMY1C (includes others),ANG,ANXA6,AOC3,APEX1,AR,ARF6,ARHGAP5,ARHGEF1,ASAP1,ATXN3,B2M,BCR,BMP1,BPI,BTK,BTRC,C1R,C8A,CAMK4,CANT1,CD1A,CD74,CDK5R1,CERT1,CIAO1,CLK1,CSDE1,CSF2RB,CSNK1G1,CSNK1G2,CSTF2,CTCF,CUL1,CYP2E1,CYP2R1,CYP7A1,DGKA,DOK2,DPP4,DSG2,ECI2,EP300,EPHA3,EPS8L2,ERBB4,ESR1,ETF1,F2,F3,FAP,FAS,FCGR2A,FHIT,FN1,GAD2,GALM,GART,GGPS1,GRHPR,GRK2,GTF2F2,GTF2I,HASPIN,HAT1,HBB,HBEGF,HCK,HDAC7,HDX,HLA,DRB1,HMGB3,HMOX1,HNRNPA2B1,HSCB,HSP90AB1,HTR1A,IDE,IFNG,IFNGR1,IGF1R,IMPDH1,IQUB,ITGB3,ITPK1,ITPKC,KLK1,KMT5A,LRBA,MAGEA4,MAGI2,MAPK6,MATK,MDH1,MECR,MMP1,MMP2,MSH2,MSN,MTAP,MUC1,MYBPC1,NAE1,NCBP1,NCOA3,NDC80,NDUFAB1,NF2,NFATC1,NOTCH1,NT5C3A,NUP214,OAT,ODC1,OXSR1,PA2G4,PANK1,PARP3,PATJ,PC,PDHA1,PDK2,PDK3,PDK4,PGC,PGK1,POT1,POU2F1,PPARG,PPP2R1A,PRKCB,PSAP,PTK2B,PTPRE,PTPRF,PYGL,RAC1,RARA,RARG,RBBP6,RGS6,RRM1,SEM1,SEMA4D,SENP2,SERPINA5,SFMBT2,SFN,SH2B2,SLC5A1,SLK,SMG5,SMG7,SMTN,SNX1,STK4,SYT13,TAF13,THBS2,TJP1,TLR4,TTR,TXNDC12,UEVLD,VAV3,VEGFB,WEE1,WWP1,XIAP,XRCC6,ZEB2,ZFP28,ZIC3,ZNF292,ZNF462
38	Development of adenocarcinoma	Cancer,Organismal Injury and Abnormalities	5.89E,12	186	AASDHPPT,ACACB,ACADSB,ACBD7,ACY1,ADH1C,ADH7,ALB,ALK,ALOX12,AOC3,APPL1,AR,ARF6,ARHGEF1,ASAP1,ATP7B,ATXN3,B2M,B3GAT2,BCR,BTK,BTRC,C8A,CA8,CANT1,CD1A,CERT1,CLCN5,CLK1,CSDE1,CSF2RB,CSNK1G1,CSNK1G2,CSTF2,CTCF,CUL1,CYP17A1,CYP7A1,DCTD,DGKA,DOK2,DPP4,DTYMK,ECHS1,EP300,EPHA3,ERBB4,ESR1,ETF1,F2,F3,FAP,FCGR2A,FLNB,FN1,G6PD,GAD2,GART,GGPS1,GRHPR,GRK2,GRK6,GTF2I,HASPIN,HBB,HBEGF,HCK,HDX,HLA,DRB1,HMBOX1,HMGB3,HNRNPA2B1,HPD,HSD17B4,HSP90AB1,HTR1A,IGF1R,IMPDH1,IQUB,ITGB3,ITPA,ITPKC,ITSN1,KIF11,KLK1,KYNU,LRBA,MAGEA4,MAGI2,MAP3K3,MAPK6,MARF1,MARK1,MATK,MDH1,MEMO1,MMP1,MMP2,MSH2,MSN,MUC1,MYBPC1,MYBPC3,MYSM1,NBEA,NCBP1,NCOA3,NDC80,NF2,NFATC1,NMRK1,NMT1,NOTCH1,NR1I3,NT5C3A,NUP214,OAT,ODC1,OXSR1,PAH,PANK1,PARN,PARP3,PATJ,PC,PDK3,PDK4,PGC,PGK1,PIP4K2C,POU2F1,PPARG,PPP2R1A,PRKCB,PTK2B,PTPRE,PTPRF,PYGL,RARA,RARG,RBBP6,RBM39,RBP5,RGS6,RND1,RRM1,RTN4IP1,RUVBL1,SART3,SEMA4D,SENP2,SERPINA5,SF3B6,SFMBT2,SFN,SH3YL1,SKP1,SLC5A1,SLK,SMG5,SMG7,SMTN,SMURF2,STK4,SUB1,SULT1A1,SYT13,THBS2,TJP1,TLR4,TRIM21,TTR,UEVLD,UTRN,VAV3,WHRN,WWP1,XIAP,YY1,ZCWPW1,ZEB2,ZFP28,ZIC3,ZNF292,ZNF462
39	B,cell neoplasm	Cancer,Hematological Disease,Immunological Disease,Organismal Injury and Abnormalities	7.76E,12	83	ALB,ALK,ALOX12,ANXA6,ARHGAP5,ARHGEF1,ATXN3,B2M,BCR,BTK,C8A,CD74,CDK5R1,CIAO1,CLK1,CSDE1,CSF2RB,CSTF2,CTCF,DGKA,DPP4,ECI2,EP300,EPHA3,ERBB4,FAS,FCGR2A,FN1,GAD2,GART,GGPS1,GRHPR,GRK2,GTF2F2,GTF2I,HASPIN,HAT1,HDAC7,HLA,DRB1,HMOX1,HSP90AB1,IFNG,IMPDH1,IQUB,ITGB3,ITPK1,LRBA,MAGI2,MMP2,MUC1,NAE1,NCBP1,NDC80,NFATC1,NOTCH1,NUP214,PC,PGC,PGK1,POT1,PRKCB,PTK2B,PTPRE,PTPRF,PYGL,RAC1,RARA,RRM1,SEM1,SH2B2,SLC5A1,SMG5,SNX1,SYT13,TAF13,THBS2,TLR4,TXNDC12,VAV3,XIAP,XRCC6,ZEB2,ZNF292
40	B cell cancer	Cancer,Hematological Disease,Immunological Disease,Organismal Injury and Abnormalities	8.06E,12	78	ALB,ALK,ANXA6,ARHGAP5,ARHGEF1,ATXN3,B2M,BCR,BTK,C8A,CD74,CDK5R1,CIAO1,CLK1,CSDE1,CSF2RB,CSTF2,CTCF,DGKA,ECI2,EP300,EPHA3,ERBB4,FAS,FCGR2A,FN1,GART,GGPS1,GRHPR,GRK2,GTF2I,HASPIN,HAT1,HDAC7,HLA,DRB1,HMOX1,HSP90AB1,IFNG,IMPDH1,IQUB,ITGB3,ITPK1,LRBA,MMP2,MUC1,NAE1,NCBP1,NDC80,NFATC1,NOTCH1,NUP214,PC,PGC,PGK1,POT1,PRKCB,PTK2B,PTPRE,PTPRF,PYGL,RAC1,RARA,RRM1,SEM1,SH2B2,SLC5A1,SMG5,SNX1,SYT13,TAF13,THBS2,TLR4,TXNDC12,VAV3,XIAP,XRCC6,ZEB2,ZNF292

41	Cancer of secretory structure	Cancer,Organismal Injury and Abnormalities	1.07E,11	256	AASDHPPT,ACACB,ACADSB,ACADVL,ACAT2,ACBD7,ACP3,ACVR2B,ACY1,ADH7,ALB,ALK,ALOX12,AMY1C (includes others),ANXA6,AOC3,APEX1,APPL1,AR,ARHGAP5,ARHGEF1,ASAP1,ATP7B,ATXN3,B2M,B3GAT2,B3GAT3,BCR,BMP1,BPI,BTK,BTRC,C1R,C8A,CA8,CANT1,CD1A,CD74,CDK5R1,CHKA,CIAO1,CLCN5,CLK1,CRYM,CSDE1,CSF2RB,CSNK1G1,CSNK1G2,CSTF2,CTCF,CUL1,CYP17A1,CYP2E1,CYP2R1,CYP7A1,DAPK2,DCTD,DCXR,DFFA,DGKA,DHPS,DMC1,DOK2,DPP4,DSG2,DTYMK,EP300,EPHA3,EPS8L2,ERBB4,ERI1,ESR1,ETF1,F2,F3,FABP7,FAP,FAS,FLNB,FN1,G6PD,GAD2,GALM,GALNT10,GART,GC,GGPS1,GRHPR,GRK2,GRK6,GSTT2/GSTT2B,GTF2F2,GTF2I,HADH,HASPIN,HAT1,HBEGF,HCK,HDAC7,HDX,HIBCH,HK2,HLA,DRB1,HMBOX1,HMGB3,HMGCS1,HMOX1,HNRNPA2B1,HPD,HSCB,HSD17B4,HSP90AB1,IDE,IFNG,IFNGR1,IGF1R,IMPA2,IMPDH1,IQUB,ITGB3,ITPK1,ITPKC,ITSN1,KIF11,KLK1,KMT5A,KYNU,LRBA,MAGI2,MAP3K3,MAPK6,MARF1,MARK1,MATK,MDH1,MECR,MEMO1,MMP1,MMP2,MSH2,MSN,MTAP,MUC1,MYBPC1,MYBPC3,MYSM1,NAE1,NBEA,NCBP1,NCOA3,NDC80,NDUFAB1,NF2,NFATC1,NME3,NMRK1,NOTCH1,NR1I3,NT5C3A,NUP214,OAT,ODC1,OXSR1,PA2G4,PAFAH1B2,PAH,PANK1,PAPSS1,PARN,PARP3,PATJ,PC,PCYT2,PDF,PDHA1,PDK3,PDK4,PEX5,PGK1,PHF24,PIP4K2C,PNMT,POT1,POU2F1,PPARG,PPP2R1A,PRKCB,PSAP,PTK2B,PTPRE,PTPRF,PYGL,RAB26,RAC1,RARA,RARG,RBBP6,RBM39,RGS6,RND1,RPA3,RRM1,RTN4IP1,RUVBL1,S100A6,SART3,SEC23A,SEM1,SEMA4D,SENP2,SERPINA5,SF3B6,SFMBT2,SFN,SH2B2,SH3YL1,SKP1,SLC5A1,SLK,SMAP1,SMG5,SMTN,SMURF2,SNX1,SNX22,SPEF1,STARD13,STK4,SUB1,SULT1A1,SYT13,TAF13,THBS2,TJP1,TLR4,TRIM21,TTR,UEVLD,USP19,UTRN,VAV3,VPS26A,WEE1,WHRN,WWP1,XIAP,XRCC6,YWHAQ,YY1,ZBTB43,ZCWPW1,ZEB2,ZFP28,ZIC3,ZNF292,ZNF462
42	Thyroid cancer	Cancer,Endocrine System Disorders,Organismal Injury and Abnormalities	1.13E,11	240	AASDHPPT,ACACB,ACADSB,ACADVL,ACAT2,ACBD7,ACVR2B,ACY1,ADH7,ALK,ALOX12,AMY1C (includes others),ANXA6,AOC3,APPL1,AR,ARHGAP5,ARHGEF1,ASAP1,ATP7B,ATXN3,B3GAT2,B3GAT3,BCR,BMP1,BPI,BTK,BTRC,C8A,CA8,CANT1,CD1A,CHKA,CIAO1,CLCN5,CLK1,CRYM,CSDE1,CSF2RB,CSNK1G1,CSNK1G2,CSTF2,CTCF,CUL1,CYP17A1,CYP2E1,CYP2R1,CYP7A1,DAPK2,DCTD,DCXR,DFFA,DGKA,DHPS,DMC1,DOK2,DPP4,DSG2,DTYMK,EP300,EPS8L2,ERBB4,ERI1,ESR1,ETF1,F2,F3,FAP,FAS,FLNB,FN1,G6PD,GAD2,GALM,GALNT10,GART,GC,GRHPR,GRK2,GRK6,GSTT2/GSTT2B,GTF2F2,GTF2I,HADH,HASPIN,HAT1,HBEGF,HCK,HDAC7,HDX,HIBCH,HK2,HLA,DRB1,HMBOX1,HMGB3,HMGCS1,HMOX1,HNRNPA2B1,HPD,HSCB,HSD17B4,HSP90AB1,IDE,IFNGR1,IGF1R,IMPA2,IMPDH1,IQUB,ITGB3,ITPK1,ITPKC,ITSN1,KIF11,KLK1,KMT5A,KYNU,LRBA,MAGI2,MAP3K3,MAPK6,MARF1,MARK1,MATK,MECR,MEMO1,MMP1,MMP2,MSH2,MSN,MTAP,MUC1,MYBPC1,MYBPC3,MYSM1,NAE1,NBEA,NCBP1,NCOA3,NDC80,NDUFAB1,NF2,NFATC1,NME3,NMRK1,NOTCH1,NR1I3,NT5C3A,NUP214,OAT,ODC1,OXSR1,PA2G4,PAFAH1B2,PAH,PANK1,PAPSS1,PARN,PARP3,PATJ,PC,PCYT2,PDF,PDHA1,PDK3,PDK4,PEX5,PGK1,PHF24,PIP4K2C,PNMT,POT1,POU2F1,PPARG,PPP2R1A,PRKCB,PSAP,PTK2B,PTPRE,PTPRF,PYGL,RAB26,RAC1,RARA,RARG,RBBP6,RBM39,RGS6,RND1,RPA3,RRM1,RTN4IP1,RUVBL1,SART3,SEC23A,SEM1,SEMA4D,SENP2,SERPINA5,SFMBT2,SH2B2,SH3YL1,SKP1,SLC5A1,SLK,SMAP1,SMG5,SMTN,SMURF2,SNX1,SNX22,SPEF1,STARD13,STK4,SUB1,SULT1A1,SYT13,TAF13,THBS2,TJP1,TLR4,TRIM21,TTR,UEVLD,USP19,UTRN,VAV3,VPS26A,WEE1,WHRN,WWP1,XRCC6,YWHAQ,YY1,ZBTB43,ZCWPW1,ZEB2,ZFP28,ZIC3,ZNF292,ZNF462
43	Breast or ovarian cancer	Cancer,Organismal Injury and Abnormalities,Reproductive System Disease	1.15E,11	136	ACACB,ALB,ALK,ANG,AOC3,AR,ARF6,ARHGEF1,ASAP1,B2M,BCR,BMP1,BTK,CA8,CAMK4,CANT1,CD74,CERT1,CLK1,CSDE1,CSF2RB,CSNK1G1,CSTF2,CTCF,CYP17A1,CYP2E1,DAPK2,DPP4,EP300,EPHA3,ERBB4,ESR1,EXOSC9,F2,FABP7,FAP,FAS,FLNB,FN1,GAD2,GALNT10,GART,GC,GGPS1,GRK2,GRK6,HAT1,HBB,HBEGF,HCK,HDAC7,HLA,DRB1,HMGCS1,HMOX1,HNRNPA2B1,HSP90AB1,HTR1A,IFNGR1,IGF1R,IMPDH1,ITGB3,ITPA,ITPKC,ITSN1,KIF11,KMT5A,MAGEA4,MAGI2,MAP3K3,MAPK6,MARF1,MARK1,MMP1,MMP2,MSH2,MSN,MUC1,MYBPC3,NAE1,NBEA,NCOA3,NDC80,NF2,NFATC1,NOTCH1,NR1I3,NT5C3A,ODC1,OXSR1,PAFAH1B2,PARP3,PC,PDK3,PDK4,PGC,PGK1,PHF24,POU2F1,PPARG,PPP2R1A,PRKCB,PTPRE,PTPRF,PYGL,RAC1,RARA,RARG,RBBP6,RBM39,RRM1,RTN4IP1,S100A6,SENP2,SERPINA5,SFMBT2,SH3YL1,SKP1,SLC5A1,SLK,SMG7,SMTN,SMURF2,SYT13,THBS2,TLR4,TTR,USP19,UTRN,VAV3,WEE1,WWP1,XIAP,YWHAQ,YY1,ZEB2,ZNF462
44	Multiple cancers	Cancer,Organismal Injury and Abnormalities	1.43E,11	139	ACACB,ALB,ALK,ANG,AOC3,AR,ARF6,ARHGEF1,ASAP1,B2M,BCR,BMP1,BTK,CA8,CAMK4,CANT1,CD74,CERT1,CLK1,CSDE1,CSF2RB,CSNK1G1,CSTF2,CTCF,CYP17A1,CYP2E1,DAPK2,DPP4,EP300,EPHA3,ERBB4,ESR1,EXOSC9,F2,FABP7,FAP,FAS,FLNB,FN1,GAD2,GALNT10,GART,GC,GGPS1,GRHPR,GRK2,GRK6,HAT

					1,HBB,HBEGF,HCK,HDAC7,HLA,DRB1,HMGCS1,HMOX1,HNRNPA2B1,HSP90AB1,HTR1A,IFNG,IFNGR1,IGF1R,IMPDH1,ITGB3,ITPA,ITPK1,ITPKC,ITSN1,KIF11,KMT5A,MAGEA4,MAGI2,MAP3K3,MAPK6,MARF1,MARK1,MMP1,MMP2,MSH2,MSN,MUC1,MYBPC3,NAE1,NBEA,NCOA3,NDC80,NF2,NFATC1,NOTCH1,NR1I3,NT5C3A,ODC1,OXSR1,PAFAH1B2,PARP3,PC,PDK3,PDK4,PGC,PGK1,PHF24,POU2F1,PPARG,PPP2R1A,PRKCB,PTPRE,PTPRF,PYGL,RAC1,RARA,RARG,RBBP6,RBM39,RRM1,RTN4IP1,S100A6,SENP2,SERPINA5,SFMBT2,SH3YL1,SKP1,SLC5A1,SLK,SMG7,SMTN,SMURF2,SYT13,THBS2,TLR4,TTR,USP19,UTRN,VAV3,WEE1,WWP1,XIAP,YWHAQ,YY1,ZEB2,ZNF462
45	Tumorigenesis of reproductive tract	Cancer,Organismal Injury and Abnormalities,Reproductive System Disease	1.44E,11	177	AASDHPPT,ACACB,ACADSB,ACBD7,ACY1,ALB,ALK,ANG,AOC3,APPL1,AR,ARF6,ARHGAP5,ARHGEF1,ASAP1,ATP7B,B2M,B3GAT2,BCR,BTK,BTRC,C8A,CA8,CD1A,CD74,CLCN5,CLK1,CSDE1,CSF2RB,CSNK1G1,CSTF2,CTCF,CUL1,CYP7A1,DCTD,DHPS,DMC1,DOK2,DPP4,DTYMK,EP300,EPHA3,ERBB4,ESR1,ETF1,F2,F3,FABP7,FAP,FAS,FCGR2A,FLNB,FN1,G6PD,GAD2,GART,GC,GRHPR,GRK2,GTF2I,HBB,HBEGF,HCK,HDAC7,HDX,HMBOX1,HMGB3,HMOX1,HNRNPA2B1,HPD,HSD17B10,HSD17B4,HSP90AB1,HTR1A,IFNG,IFNGR1,IGF1R,IMPDH1,IQUB,ITGB3,ITPA,ITPKC,ITSN1,KIF11,LRBA,MAGEA4,MAGI2,MAP3K3,MAPK6,MARF1,MARK1,MDH1,MEMO1,MMP1,MMP2,MSH2,MSN,MUC1,MYBPC3,MYSM1,NAE1,NBEA,NCBP1,NCOA3,NF2,NFATC1,NMRK1,NMT1,NOTCH1,NR1I3,NT5C3A,NUP214,OAT,ODC1,PANK1,PARN,PARP3,PATJ,PC,PDK3,PDK4,PGC,PGK1,POU2F1,PPARG,PPP2R1A,PRKCB,PTPRE,PTPRF,PYGL,RAC1,RARA,RARG,RBBP6,RBM39,RGS6,RND1,RRM1,RTN4IP1,RUVBL1,S100A6,SART3,SENP2,SERPINA5,SF3B6,SFMBT2,SH3YL1,SKP1,SLC5A1,SLK,SMG5,SMTN,SMURF2,SNX1,STK4,SUB1,SULT1A1,SYT13,TAF13,THBS2,TJP1,TLR4,TRIM21,TTR,UEVLD,UTRN,VAV3,VEGFB,WEE1,WWP1,XIAP,YY1,ZEB2,ZFP28,ZIC3,ZNF292,ZNF462
46	Female genital neoplasm	Cancer,Organismal Injury and Abnormalities,Reproductive System Disease	1.63E,11	177	AASDHPPT,ACACB,ACADSB,ACBD7,ACY1,ALB,ALK,ANG,AOC3,APPL1,AR,ARF6,ARHGAP5,ARHGEF1,ASAP1,ATP7B,B2M,B3GAT2,BCR,BTK,BTRC,C8A,CA8,CD1A,CD74,CLCN5,CLK1,CSDE1,CSF2RB,CSNK1G1,CSTF2,CTCF,CUL1,CYP7A1,DCTD,DHPS,DMC1,DOK2,DPP4,DTYMK,EP300,EPHA3,ERBB4,ESR1,ETF1,F2,F3,FABP7,FAP,FAS,FCGR2A,FLNB,FN1,G6PD,GAD2,GART,GC,GRHPR,GRK2,GTF2I,HBB,HBEGF,HCK,HDAC7,HDX,HMBOX1,HMGB3,HMOX1,HNRNPA2B1,HPD,HSD17B10,HSD17B4,HSP90AB1,HTR1A,IFNG,IFNGR1,IGF1R,IMPDH1,IQUB,ITGB3,ITPA,ITPKC,ITSN1,KIF11,LRBA,MAGEA4,MAGI2,MAP3K3,MAPK6,MARF1,MARK1,MDH1,MEMO1,MMP1,MMP2,MSH2,MSN,MUC1,MYBPC3,MYSM1,NAE1,NBEA,NCBP1,NCOA3,NF2,NFATC1,NMRK1,NMT1,NOTCH1,NR1I3,NT5C3A,NUP214,OAT,ODC1,PANK1,PARN,PARP3,PATJ,PC,PDK3,PDK4,PGC,PGK1,POU2F1,PPARG,PPP2R1A,PRKCB,PTPRE,PTPRF,PYGL,RAC1,RARA,RARG,RBBP6,RBM39,RGS6,RND1,RRM1,RTN4IP1,RUVBL1,S100A6,SART3,SENP2,SERPINA5,SF3B6,SFMBT2,SH3YL1,SKP1,SLC5A1,SLK,SMG5,SMTN,SMURF2,SNX1,STK4,SUB1,SULT1A1,SYT13,TAF13,THBS2,TJP1,TLR4,TRIM21,TTR,UEVLD,UTRN,VAV3,VEGFB,WEE1,WWP1,XIAP,YY1,ZEB2,ZFP28,ZIC3,ZNF292,ZNF462
47	Recurrent cancer	Cancer,Organismal Injury and Abnormalities	1.79E,11	24	ALB,ALK,AR,BTK,CSF2RB,CYP17A1,ERBB4,ESR1,F3,GART,GGPS1,HCK,HDAC7,HSP90AB1,IGF1R,IMPDH1,NOTCH1,ODC1,PARP3,PTK2B,RARA,RARG,RRM1,VEGFB
48	Progression of malignant tumor	Cancer,Organismal Injury and Abnormalities,Tumor Morphology	1.99E,11	20	ALK,AR,BTK,CSF2RB,CYP17A1,EP300,ERBB4,ESR1,F3,GART,HSP90AB1,IGF1R,NOTCH1,ODC1,PARP3,PTK2B,RARA,RARG,RRM1,VEGFB
49	Non,small cell lung carcinoma	Cancer,Organismal Injury and Abnormalities,Respiratory Disease	2.01E,11	104	ADH1C,ADH7,ALB,ALK,ALOX12,ANXA6,APEX1,AR,B2M,BTK,BTRC,C8A,CAMK4,CD1A,CERT1,CLK1,CSNK1G2,CTCF,CUL1,CYP17A1,CYP2E1,DGKA,DOK2,DPP4,EP300,EPHA3,ERBB4,ESR1,F2,F3,FAP,FAS,FHIT,FN1,GAD2,GART,GGPS1,GRHPR,GRK6,HASPIN,HCK,HDX,HMGB3,HNRNPA2B1,HPD,HSD17B4,HSP90AB1,HTR1A,IFNG,IGF1R,ITPKC,KIF11,KYNU,LRBA,MAGEA4,MAGI2,MAPK6,MATK,MDH1,MMP1,MMP2,MSH2,MSN,MTAP,MUC1,NBEA,NCOA3,NDC80,NMT1,NOTCH1,NT5C3A,OAT,OXSR1,PAH,PANK1,PARP3,PATJ,PDK4,PGC,PIP4K2C,PPARG,PPP2R1A,PRKCB,PTK2B,PTPRE,PTPRF,PYGL,RARA,RARG,RGS6,RRM1,SEMA4D,SENP2,SERPINA5,SFN,SLK,SMG7,STK4,SULT1A1,TLR4,TTR,WWP1,ZCWPW1,ZNF292
50	Head and neck carcinoma	Cancer,Organismal Injury and Abnormalities	2.03E,11	248	AASDHPPT,ACACB,ACADSB,ACADVL,ACAT2,ACBD7,ACVR2B,ACY1,ADH7,ALB,ALK,ALOX12,AMY1C (includes others),ANXA6,AOC3,APPL1,AR,ARF6,ARHGAP5,ARHGEF1,ASAP1,ATP7B,ATXN3,B2M,B3GAT2,B3GAT3,BCR,BMP1,BPI,BTK,BTRC,C8A,CA8,CANT1,CD1A,CD74,CHKA,CIAO1,CLCN5,CLK1,CRYM,CSDE1,CSF2RB,CSNK1G1,CSNK1G2,CSTF2,CTCF,CUL1,CYP17A1,CYP2E1,CYP2R1,CYP7A1,DAPK2,DCTD,DCXR,DFFA,

					DGKA,DHPS,DMC1,DOK2,DPP4,DSG2,DTYMK,EP300,EPHA3,EPS8L2,ERBB4,ERI1,ESR1,ETF1,F2,F3,FAP,FCGR2A,FHIT,FLNB,FN1,G6PD,GAD2,GALM,GALNT10,GART,GC,GRHPR,GRK2,GRK6,GSTT2/GSTT2B,GTF2F2,GTF2I,HADH,HASPIN,HAT1,HBEGF,HCK,HDAC7,HDX,HIBCH,HK2,HLA,DRB1,HMBOX1,HMGB3,HMGCS1,HMOX1,HNRNPA2B1,HPD,HSCB,HSD17B4,HSP90AB1,HTR1A,IDE,IFNGR1,IGF1R,IMPA2,IMPDH1,IQUB,ITGB3,ITPK1,ITPKC,ITSN1,KIF11,KLK1,KMT5A,KYNU,LRBA,MAGI2,MAP3K3,MAPK6,MARF1,MARK1,MATK,MECR,MEMO1,MMP1,MMP2,MSH2,MSN,MTAP,MUC1,MYBPC1,MYBPC3,MYSM1,NAE1,NBEA,NCBP1,NCOA3,NDC80,NDUFAB1,NF2,NFATC1,NME3,NMRK1,NOTCH1,NR1I3,NT5C3A,NUP214,OAT,ODC1,OXSR1,PA2G4,PAFAH1B2,PAH,PANK1,PAPSS1,PARN,PARP3,PATJ,PC,PCYT2,PDF,PDHA1,PDK2,PDK3,PDK4,PEX5,PGK1,PHF24,PIP4K2C,PNMT,POT1,POU2F1,PPARG,PPP2R1A,PRKCB,PSAP,PTK2B,PTPRE,PTPRF,PYGL,RAB26,RAC1,RARA,RARG,RBBP6,RBM39,RGS6,RND1,RPA3,RRM1,RTN4IP1,RUVBL1,SART3,SEC23A,SEM1,SEMA4D,SENP2,SERPINA5,SFMBT2,SH2B2,SH3YL1,SKP1,SLC5A1,SLK,SMAP1,SMG5,SMG7,SMTN,SMURF2,SNX1,SNX22,SPEF1,STARD13,STK4,SUB1,SULT1A1,SYT13,THBS2,TJP1,TLR4,TRIM21,TTR,UEVLD,USP19,UTRN,VAV3,VPS26A,WEE1,WHRN,WWP1,XRCC6,YWHAQ,YY1,ZBTB43,ZCWPW1,ZEB2,ZFP28,ZIC3,ZNF292,ZNF462
51	Communication of cells	Cell,To,Cell Signaling and Interaction	2.88E,11	50	ACVR2B,ALK,APPL1,AR,ARHGEF1,ATXN3,BCR,BTK,BTRC,CAMK4,CSNK1G2,DAPK2,DOK2,ERBB4,ESR1,F2,FAS,FLNB,GRK2,GRK6,HASPIN,HBEGF,HLA,DRB1,HMOX1,HTR1A,IFNG,IFNGR1,IGF1R,ITPK1,ITSN1,MAGI2,MAP3K3,MAPK6,MARK1,NAE1,NFATC1,NOTCH1,NR1I3,OXSR1,PPARG,PSAP,PTK2B,RAC1,RARA,RGS6,S100A6,SFN,STK4,THBS2,VAV3
52	Metastatic solid tumor	Cancer,Organismal Injury and Abnormalities	2.94E,11	39	ALB,ALK,AOC3,AR,B2M,C1R,CSF2RB,CYP17A1,DSG2,EPHA3,ERBB4,ESR1,F2,F3,FAS,FCGR2A,FN1,GAD2,GART,GGPS1,HMOX1,HSP90AB1,IGF1R,IMPDH1,MMP2,MSH2,MUC1,NF2,NOTCH1,NR1I3,PARP3,PGC,PPP2R1A,PTK2B,RAC1,RARA,RARG,RRM1,VEGFB
53	Non,hematological solid tumor	Cancer,Organismal Injury and Abnormalities	3.15E,11	285	AASDHPPT,ACACB,ACADSB,ACADVL,ACAT2,ACBD7,ACSM2A,ACVR2B,ACY1,ADH1C,ADH7,ALB,ALK,ALOX12,AMY1C (includes others),ANG,ANXA6,AOC3,APEX1,APPL1,AR,ARF6,ARHGAP5,ARHGEF1,ASAP1,ATP7B,ATXN3,B2M,B3GAT2,B3GAT3,BCR,BMP1,BPI,BTK,BTRC,C1R,C8A,CA8,CAMK4,CANT1,CD1A,CD74,CDK5R1,CERT1,CHKA,CIAO1,CLCN5,CLK1,CRYM,CSDE1,CSF2RB,CSNK1G1,CSNK1G2,CSTF2,CTCF,CUL1,CYP17A1,CYP2E1,CYP2R1,CYP7A1,DAPK2,DCTD,DCXR,DFFA,DGKA,DHPS,DMC1,DOK2,DPP4,DSG2,DTYMK,ECHS1,ECI2,EP300,EPHA3,EPS8L2,ERBB4,ERI1,ESR1,ETF1,EXOSC9,F2,F3,FABP3,FABP7,FAP,FAS,FCGR2A,FHIT,FLNB,FN1,G6PD,GAD2,GALM,GALNT10,GART,GC,GGPS1,GRHPR,GRK2,GRK6,GSTT2/GSTT2B,GTF2F2,GTF2I,HADH,HASPIN,HAT1,HBB,HBEGF,HCK,HDAC7,HDX,HIBCH,HK2,HLA,DRB1,HMBOX1,HMGB3,HMGCS1,HMOX1,HNRNPA2B1,HPD,HSCB,HSD17B10,HSD17B4,HSP90AB1,HTR1A,IDE,IFNG,IFNGR1,IGF1R,IMPA2,IMPDH1,IQUB,ITGB3,ITPA,ITPK1,ITPKC,ITSN1,KIF11,KLK1,KMT5A,KYNU,LRBA,MAGEA4,MAGI2,MAP3K3,MAPK6,MARF1,MARK1,MATK,MDH1,MECR,MEMO1,MMP1,MMP2,MSH2,MSN,MTAP,MUC1,MYBPC1,MYBPC3,MYSM1,NAE1,NBEA,NCBP1,NCOA3,NDC80,NDUFAB1,NF2,NFATC1,NME3,NMRK1,NMT1,NOTCH1,NR1I3,NSUN5,NT5C3A,NUP214,OAT,ODC1,OXSR1,PA2G4,PAFAH1B2,PAH,PANK1,PAPSS1,PARN,PARP3,PATJ,PC,PCYT2,PDF,PDHA1,PDK2,PDK3,PDK4,PEX5,PGC,PGK1,PHF24,PIP4K2C,PNMT,POT1,POU2F1,PPARG,PPP2R1A,PRKCB,PSAP,PTK2B,PTPRE,PTPRF,PUS10,PYGL,RAB26,RAC1,RARA,RARG,RBBP6,RBM39,RBP5,RGS6,RND1,RPA3,RRM1,RTN4IP1,RUVBL1,S100A6,SART3,SEC23A,SEM1,SEMA4D,SENP2,SERPINA5,SF3B6,SFMBT2,SFN,SH2B2,SH3YL1,SKP1,SLC5A1,SLK,SMAP1,SMG5,SMG7,SMTN,SMURF2,SNX1,SNX22,SPEF1,STARD13,STK16,STK4,SUB1,SULT1A1,SYT13,TAF13,THBS2,TIMM9,TJP1,TLR4,TNFAIP8L2,TRIM21,TTR,TXNDC12,UEVLD,USP19,UTRN,VAV3,VEGFB,VPS26A,WEE1,WHRN,WWP1,XIAP,XRCC6,YWHAH,YWHAQ,YY1,ZBTB43,ZCWPW1,ZEB2,ZFP28,ZIC3,ZNF292,ZNF462
54	Head and neck tumor	Cancer,Organismal Injury and Abnormalities	3.38E,11	264	AASDHPPT,ACACB,ACADSB,ACADVL,ACAT2,ACBD7,ACVR2B,ACY1,ADH7,ALB,ALK,ALOX12,AMY1C (includes others),ANXA6,AOC3,APEX1,APPL1,AR,ARF6,ARHGAP5,ARHGEF1,ASAP1,ATP7B,ATXN3,B2M,B3GAT2,B3GAT3,BCR,BMP1,BPI,BTK,BTRC,C1R,C8A,CA8,CANT1,CD1A,CD74,CHKA,CIAO1,CLCN5,CLK1,CRYM,CSDE1,CSF2RB,CSNK1G1,CSNK1G2,CSTF2,CTCF,CUL1,CYP17A1,CYP2E1,CYP2R1,CYP7A1,DAPK2,DCTD,DCXR,DFFA,DGKA,DHPS,DMC1,DOK2,DPP4,DSG2,DTYMK,EP300,EPHA3,EPS8L2,ERBB4,ERI1,ESR1,ETF1,F2,F3,FAP,FAS,FCGR2A,FHIT,FLNB,FN1,G6PD,GAD2,GALM,GALNT10,GART,GC,GRHPR,GRK2,GRK6,GSTT2/GSTT2B,GTF2F2,GTF2I,HADH,HASPIN,HAT1,HBEGF,HCK,HDAC7,HDX,HIBCH,HK2,HLA,DRB1,HMBOX

					1,HMGB3,HMGCS1,HMOX1,HNRNPA2B1,HPD,HSCB,HSD17B10,HSD17B4,HSP90AB1,HTR1A,IDE,IFNGR1,IGF1R,IMPA2,IMPDH1,IQUB,ITGB3,ITPK1,ITPKC,ITSN1,KIF11,KLK1,KMT5A,KYNU,LRBA,MAGEA4,MAGI2,MAP3K3,MAPK6,MARF1,MARK1,MATK,MECR,MEMO1,MMP1,MMP2,MSH2,MSN,MTAP,MUC1,MYBPC1,MYBPC3,MYSM1,NAE1,NBEA,NCBP1,NCOA3,NDC80,NDUFAB1,NF2,NFATC1,NME3,NMRK1,NMT1,NOTCH1,NR1I3,NSUN5,NT5C3A,NUP214,OAT,ODC1,OXSR1,PA2G4,PAFAH1B2,PAH,PANK1,PAPSS1,PARN,PARP3,PATJ,PC,PCYT2,PDF,PDHA1,PDK2,PDK3,PDK4,PEX5,PGC,PGK1,PHF24,PIP4K2C,PNMT,POT1,POU2F1,PPARG,PPP2R1A,PRKCB,PSAP,PTK2B,PTPRE,PTPRF,PUS10,PYGL,RAB26,RAC1,RARA,RARG,RBBP6,RBM39,RBP5,RGS6,RND1,RPA3,RRM1,RTN4IP1,RUVBL1,SART3,SEC23A,SEM1,SEMA4D,SENP2,SERPINA5,SFMBT2,SFN,SH2B2,SH3YL1,SKP1,SLC5A1,SLK,SMAP1,SMG5,SMG7,SMTN,SMURF2,SNX1,SNX22,SPEF1,STARD13,STK4,SUB1,SULT1A1,SYT13,TAF13,THBS2,TIMM9,TJP1,TLR4,TNFAIP8L2,TRIM21,TTR,UEVLD,USP19,UTRN,VAV3,VEGFB,VPS26A,WEE1,WHRN,WWP1,XIAP,XRCC6,YWHAQ,YY1,ZBTB43,ZCWPW1,ZEB2,ZFP28,ZIC3,ZNF292,ZNF462
55	HIV infection	Infectious Diseases	3.44E,11	43	ACACB,ACADSB,ACP3,ALB,ALOX12,AR,ARF6,B2M,BCR,BMP1,C1R,CANT1,CD74,CSF2RB,DAPK2,DHPS,DPP4,EP300,ESR1,ETF1,FAS,FCGR2A,FN1,GGPS1,GRK2,HIBCH,HMGCS1,IFNG,IMPDH1,IQUB,LARS1,NF2,NMT1,NUP214,PANK1,PPARG,RARA,RARG,RND1,RRM1,SUB1,XIAP,ZNF292
56	Mature B,cell lymphoma	Cancer,Hematological Disease,Immunological Disease,Organismal Injury and Abnormalities	3.89E,11	39	ALK,ANXA6,ARHGEF1,B2M,BCR,BTK,CLK1,CSF2RB,CTCF,ECI2,EP300,EPHA3,ERBB4,FAS,FCGR2A,FN1,GTF2I,HAT1,HMOX1,HSP90AB1,IFNG,IMPDH1,IQUB,ITGB3,MMP2,NOTCH1,PC,PGK1,PRKCB,PTK2B,PTPRE,PTPRF,RAC1,RRM1,SLC5A1,SNX1,TXNDC12,XIAP,XRCC6
57	Nonpituitary endocrine tumor	Cancer,Endocrine System Disorders,Organismal Injury and Abnormalities	4.16E,11	241	AASDHPPT,ACACB,ACADSB,ACADVL,ACAT2,ACBD7,ACVR2B,ACY1,ADH7,ALK,ALOX12,AMY1C (includes others),ANXA6,AOC3,APPL1,AR,ARHGAP5,ARHGEF1,ASAP1,ATP7B,ATXN3,B3GAT2,B3GAT3,BCR,BMP1,BPI,BTK,BTRC,C8A,CA8,CANT1,CD1A,CHKA,CIAO1,CLCN5,CLK1,CRYM,CSDE1,CSF2RB,CSNK1G1,CSNK1G2,CSTF2,CTCF,CUL1,CYP17A1,CYP2E1,CYP2R1,CYP7A1,DAPK2,DCTD,DCXR,DFFA,DGKA,DHPS,DMC1,DOK2,DPP4,DSG2,DTYMK,EP300,EPS8L2,ERBB4,ERI1,ESR1,ETF1,F2,F3,FABP3,FAP,FAS,FLNB,FN1,G6PD,GAD2,GALM,GALNT10,GART,GC,GRHPR,GRK2,GRK6,GSTT2/GSTT2B,GTF2F2,GTF2I,HADH,HASPIN,HAT1,HBEGF,HCK,HDAC7,HDX,HIBCH,HK2,HLA,DRB1,HMBOX1,HMGB3,HMGCS1,HMOX1,HNRNPA2B1,HPD,HSCB,HSD17B4,HSP90AB1,IDE,IFNGR1,IGF1R,IMPA2,IMPDH1,IQUB,ITGB3,ITPK1,ITPKC,ITSN1,KIF11,KLK1,KMT5A,KYNU,LRBA,MAGI2,MAP3K3,MAPK6,MARF1,MARK1,MATK,MECR,MEMO1,MMP1,MMP2,MSH2,MSN,MTAP,MUC1,MYBPC1,MYBPC3,MYSM1,NAE1,NBEA,NCBP1,NCOA3,NDC80,NDUFAB1,NF2,NFATC1,NME3,NMRK1,NOTCH1,NR1I3,NT5C3A,NUP214,OAT,ODC1,OXSR1,PA2G4,PAFAH1B2,PAH,PANK1,PAPSS1,PARN,PARP3,PATJ,PC,PCYT2,PDF,PDHA1,PDK3,PDK4,PEX5,PGK1,PHF24,PIP4K2C,PNMT,POT1,POU2F1,PPARG,PPP2R1A,PRKCB,PSAP,PTK2B,PTPRE,PTPRF,PYGL,RAB26,RAC1,RARA,RARG,RBBP6,RBM39,RGS6,RND1,RPA3,RRM1,RTN4IP1,RUVBL1,SART3,SEC23A,SEM1,SEMA4D,SENP2,SERPINA5,SFMBT2,SH2B2,SH3YL1,SKP1,SLC5A1,SLK,SMAP1,SMG5,SMTN,SMURF2,SNX1,SNX22,SPEF1,STARD13,STK4,SUB1,SULT1A1,SYT13,TAF13,THBS2,TJP1,TLR4,TRIM21,TTR,UEVLD,USP19,UTRN,VAV3,VPS26A,WEE1,WHRN,WWP1,XRCC6,YWHAQ,YY1,ZBTB43,ZCWPW1,ZEB2,ZFP28,ZIC3,ZNF292,ZNF462
58	Cancer of cells	Cancer,Organismal Injury and Abnormalities	4.32E,11	161	ALB,ALK,ALOX12,ANXA6,AOC3,AR,ARF6,ARHGAP5,ARHGEF1,ASAP1,ATXN3,B2M,BCR,BPI,BTK,BTRC,C1R,C8A,CAMK4,CD1A,CD74,CDK5R1,CERT1,CIAO1,CLK1,CSDE1,CSF2RB,CSNK1G1,CSNK1G2,CSTF2,CTCF,CUL1,CYP2E1,CYP7A1,DGKA,DOK2,DPP4,DSG2,ECI2,EP300,EPHA3,EPS8L2,ERBB4,F2,F3,FAP,FAS,FCGR2A,FHIT,FN1,GAD2,GALM,GART,GGPS1,GRHPR,GRK2,GTF2F2,GTF2I,HASPIN,HAT1,HBB,HBEGF,HCK,HDAC7,HDX,HLA,DRB1,HMOX1,HNRNPA2B1,HSCB,HSP90AB1,HTR1A,IDE,IFNG,IFNGR1,IGF1R,IMPDH1,IQUB,ITGB3,ITPK1,ITPKC,KLK1,KMT5A,LRBA,MAGI2,MAPK6,MATK,MDH1,MECR,MMP1,MMP2,MSH2,MSN,MTAP,MUC1,MYBPC1,NAE1,NCBP1,NCOA3,NDC80,NDUFAB1,NF2,NFATC1,NOTCH1,NT5C3A,NUP214,OAT,ODC1,OXSR1,PANK1,PARP3,PATJ,PC,PDK2,PDK4,PGC,PGK1,POT1,POU2F1,PPARG,PPP2R1A,PRKCB,PSAP,PTK2B,PTPRE,PTPRF,PYGL,RAC1,RARA,RARG,RBBP6,RGS6,RRM1,SEM1,SEMA4D,SENP2,SERPINA5,SFMBT2,SH2B2,SLC5A1,SLK,SMG5,SMG7,SMTN,SNX1,STK4,SYT13,TAF13,THBS2,TJP1,TLR4,TTR,TXNDC12,VAV3,WWP1,XIAP,XRCC6,ZEB2,ZFP28,ZIC3,ZNF292,ZNF462

59	Relapsed malignant solid tumor	Cancer,Organismal Injury and Abnormalities	4.53E,11	20	ALB,ALK,AR,BTK,CSF2RB,CYP17A1,ERBB4,ESR1,F3,GART,HDAC7,HSP90AB1,IGF1R,IMPDH1,ODC1,PARP3,PTK2B,RARA,RARG,RRM1
60	Lymphoreticular neoplasm	Cancer,Organismal Injury and Abnormalities	4.65E,11	101	ALK,AMY1C (includes others),ANXA6,APEX1,ARHGEF1,ATXN3,B2M,BCR,BMP1,BTK,BTRC,C1R,CANT1,CD1A,CD74,CLK1,CSDE1,CSF2RB,CSNK1G2,CSTF2,CTCF,CYP2E1,CYP2R1,DGKA,DPP4,ECI2,EP300,EPHA3,ERBB4,ESR1,ETF1,F3,FAP,FAS,FCGR2A,FHIT,FN1,GAD2,GART,GGPS1,GTF2I,HAT1,HCK,HDAC7,HLA,DRB1,HMGB3,HMOX1,HNRNPA2B1,HSP90AB1,IFNG,IGF1R,IMPDH1,IQUB,ITGB3,KLK1,KMT5A,MAGEA4,MMP1,MMP2,MSH2,MUC1,NCBP1,NFATC1,NOTCH1,NT5C3A,NUP214,ODC1,PA2G4,PC,PDHA1,PDK3,PDK4,PGK1,POT1,POU2F1,PRKCB,PTK2B,PTPRE,PTPRF,RAC1,RARA,RARG,RRM1,SEM1,SH2B2,SLC5A1,SMG7,SMTN,SNX1,STK4,THBS2,TJP1,TLR4,TXNDC12,UEVLD,VAV3,VEGFB,WEE1,XIAP,XRCC6,ZNF292
61	Breast cancer	Cancer,Organismal Injury and Abnormalities,Reproductive System Disease	4.76E,11	107	ACACB,ALB,ALK,ANG,AR,ARHGEF1,BCR,BMP1,BTK,CAMK4,CANT1,CERT1,CLK1,CSDE1,CSF2RB,CSNK1G1,CTCF,CYP17A1,CYP2E1,DAPK2,DPP4,EP300,EPHA3,ERBB4,ESR1,EXOSC9,FABP7,FAP,FAS,FLNB,FN1,GALNT10,GGPS1,GRK2,GRK6,HAT1,HBB,HBEGF,HCK,HDAC7,HLA,DRB1,HMGCS1,HMOX1,HSP90AB1,IGF1R,IMPDH1,ITGB3,ITPKC,KIF11,KMT5A,MAGEA4,MAPK6,MARF1,MARK1,MMP1,MMP2,MSH2,MSN,MUC1,MYBPC3,NAE1,NBEA,NCOA3,NDC80,NF2,NFATC1,NOTCH1,ODC1,OXSR1,PAFAH1B2,PARP3,PC,PDK3,PDK4,PGK1,PHF24,POU2F1,PPARG,PPP2R1A,PRKCB,PTPRE,PTPRF,RAC1,RARA,RARG,RBBP6,RBM39,RRM1,S100A6,SENP2,SERPINA5,SLC5A1,SLK,SMG7,SMTN,SMURF2,SYT13,TLR4,TTR,USP19,UTRN,VAV3,WEE1,YWHAQ,YY1,ZEB2,ZNF462
62	Progressive malignant solid tumor	Cancer,Organismal Injury and Abnormalities,Tumor Morphology	5.25E,11	18	ALK,AR,BTK,CSF2RB,CYP17A1,ERBB4,ESR1,F3,GART,HSP90AB1,IGF1R,ODC1,PARP3,PTK2B,RARA,RARG,RRM1,VEGFB
63	B,cell lymphoma	Cancer,Hematological Disease,Immunological Disease,Organismal Injury and Abnormalities	5.78E,11	53	ALK,ANXA6,ARHGEF1,ATXN3,B2M,BCR,BTK,CLK1,CSDE1,CSF2RB,CSTF2,CTCF,DGKA,ECI2,EP300,EPHA3,ERBB4,FAS,FCGR2A,FN1,GTF2I,HAT1,HDAC7,HMOX1,HSP90AB1,IFNG,IMPDH1,IQUB,ITGB3,MMP2,MUC1,NCBP1,NFATC1,NOTCH1,NUP214,PC,PGK1,POT1,PRKCB,PTK2B,PTPRE,PTPRF,RAC1,RRM1,SEM1,SLC5A1,SNX1,THBS2,TXNDC12,VAV3,XIAP,XRCC6,ZNF292
64	Thyroid carcinoma	Cancer,Endocrine System Disorders,Organismal Injury and Abnormalities	6.07E,11	238	AASDHPPT,ACACB,ACADSB,ACADVL,ACAT2,ACBD7,ACVR2B,ACY1,ADH7,ALK,ALOX12,AMY1C (includes others),ANXA6,AOC3,APPL1,AR,ARHGAP5,ARHGEF1,ASAP1,ATP7B,ATXN3,B3GAT2,B3GAT3,BCR,BMP1,BPI,BTK,BTRC,C8A,CA8,CANT1,CD1A,CHKA,CIAO1,CLCN5,CLK1,CRYM,CSDE1,CSF2RB,CSNK1G1,CSNK1G2,CSTF2,CTCF,CUL1,CYP17A1,CYP2E1,CYP2R1,CYP7A1,DAPK2,DCTD,DCXR,DFFA,DGKA,DHPS,DMC1,DOK2,DPP4,DSG2,DTYMK,EP300,EPS8L2,ERBB4,ERI1,ESR1,ETF1,F2,F3,FAP,FLNB,FN1,G6PD,GAD2,GALM,GALNT10,GART,GC,GRHPR,GRK2,GRK6,GSTT2/GSTT2B,GTF2F2,GTF2I,HADH,HASPIN,HAT1,HBEGF,HCK,HDAC7,HDX,HIBCH,HK2,HLA,DRB1,HMBOX1,HMGB3,HMGCS1,HMOX1,HNRNPA2B1,HPD,HSCB,HSD17B4,HSP90AB1,IDE,IFNGR1,IGF1R,IMPA2,IMPDH1,IQUB,ITGB3,ITPK1,ITPKC,ITSN1,KIF11,KLK1,KMT5A,KYNU,LRBA,MAGI2,MAP3K3,MAPK6,MARF1,MARK1,MATK,MECR,MEMO1,MMP1,MMP2,MSH2,MSN,MTAP,MUC1,MYBPC1,MYBPC3,MYSM1,NAE1,NBEA,NCBP1,NCOA3,NDC80,NDUFAB1,NF2,NFATC1,NME3,NMRK1,NOTCH1,NR1I3,NT5C3A,NUP214,OAT,ODC1,OXSR1,PA2G4,PAFAH1B2,PAH,PANK1,PAPSS1,PARN,PARP3,PATJ,PC,PCYT2,PDF,PDHA1,PDK3,PDK4,PEX5,PGK1,PHF24,PIP4K2C,PNMT,POT1,POU2F1,PPARG,PPP2R1A,PRKCB,PSAP,PTK2B,PTPRE,PTPRF,PYGL,RAB26,RAC1,RARA,RARG,RBBP6,RBM39,RGS6,RND1,RPA3,RRM1,RTN4IP1,RUVBL1,SART3,SEC23A,SEM1,SEMA4D,SENP2,SERPINA5,SFMBT2,SH2B2,SH3YL1,SKP1,SLC5A1,SLK,SMAP1,SMG5,SMTN,SMURF2,SNX1,SNX22,SPEF1,STARD13,STK4,SUB1,SULT1A1,SYT13,THBS2,TJP1,TLR4,TRIM21,TTR,UEVLD,USP19,UTRN,VAV3,VPS26A,WEE1,WHRN,WWP1,XRCC6,YWHAQ,YY1,ZBTB43,ZCWPW1,ZEB2,ZFP28,ZIC3,ZNF292,ZNF462
65	Pelvic tumor	Cancer,Organismal Injury and Abnormalities	6.25E,11	213	AASDHPPT,ACACB,ACADSB,ACADVL,ACBD7,ACP3,ACY1,ADH7,ALB,ALK,ANG,ANXA6,AOC3,APEX1,APPL1,AR,ARF6,ARHGAP5,ARHGEF1,ASAP1,ATP7B,B2M,B3GAT2,BCR,BMP1,BTK,BTRC,C1R,C8A,CA8,CANT1,CD1A,CD74,CLCN5,CLK1,CSDE1,CSF2RB,CSNK1G1,CSTF2,CTCF,CUL1,CYP17A1,CYP2E1,CYP7A1,DCTD,DGKA,DHPS,DMC1,DOK2,DPP4,DTYMK,EP300,EPHA3,EPS8L2,ERBB4,ESR1,ETF1,F2,F3,FABP7,FAP,FAS,FCGR2A,FLNB,FN1,G6PD,GAD2,GALM,GALNT10,GART,GC,GGPS1,GRHPR,GRK2,GTF2I,HASPIN,HBB,

					HBEGF,HCK,HDAC7,HDX,HLA,DRB1,HMBOX1,HMGB3,HMOX1,HNRNPA2B1,HPD,HSD17B10,HSD17B4,HSP90AB1,HTR1A,IFNG,IFNGR1,IGF1R,IMPDH1,IQUB,ITGB3,ITPA,ITPKC,ITSN1,KIF11,KMT5A,KYNU,LRBA,MAGEA4,MAGI2,MAP3K3,MAPK6,MARF1,MARK1,MATK,MDH1,MEMO1,MMP1,MMP2,MSH2,MSN,MTAP,MUC1,MYBPC1,MYBPC3,MYSM1,NAE1,NBEA,NCBP1,NCOA3,NF2,NFATC1,NMRK1,NMT1,NOTCH1,NR1I3,NT5C3A,NUP214,OAT,ODC1,OXSR1,PA2G4,PAH,PANK1,PAPSS1,PARN,PARP3,PATJ,PC,PDK3,PDK4,PGC,PGK1,PHF24,POU2F1,PPARG,PPP2R1A,PRKCB,PSAP,PTK2B,PTPRE,PTPRF,PYGL,RAC1,RARA,RARG,RBBP6,RBM39,RGS6,RND1,RRM1,RTN4IP1,RUVBL1,S100A6,SART3,SEMA4D,SENP2,SERPINA5,SF3B6,SFMBT2,SFN,SH3YL1,SKP1,SLC5A1,SLK,SMAP1,SMG5,SMTN,SMURF2,SNX1,STK4,SUB1,SULT1A1,SYT13,TAF13,THBS2,TJP1,TLR4,TRIM21,TTR,UEVLD,USP19,UTRN,VAV3,VEGFB,WEE1,WHRN,WWP1,XIAP,YWHAQ,YY1,ZCWPW1,ZEB2,ZFP28,ZIC3,ZNF292,ZNF462
66	Extracranial solid tumor	Cancer,Organismal Injury and Abnormalities	6.96E,11	286	AASDHPPT,ACACB,ACADSB,ACADVL,ACAT2,ACBD7,ACP3,ACSM2A,ACVR2B,ACY1,ADH1C,ADH7,ALB,ALK,ALOX12,AMY1C (includes others),ANG,ANXA6,AOC3,APEX1,APPL1,AR,ARF6,ARHGAP5,ARHGEF1,ASAP1,ATP7B,ATXN3,B2M,B3GAT2,B3GAT3,BCR,BMP1,BPI,BTK,BTRC,C1R,C8A,CA8,CAMK4,CANT1,CD1A,CD74,CDK5R1,CERT1,CHKA,CIAO1,CLCN5,CLK1,CRYM,CSDE1,CSF2RB,CSNK1G1,CSNK1G2,CSTF2,CTCF,CUL1,CYP17A1,CYP2E1,CYP2R1,CYP7A1,DAPK2,DCTD,DCXR,DFFA,DGKA,DHPS,DMC1,DOK2,DPP4,DSG2,DTYMK,ECHS1,ECI2,EP300,EPHA3,EPS8L2,ERBB4,ERI1,ESR1,ETF1,EXOSC9,F2,F3,FABP3,FABP7,FAP,FAS,FCGR2A,FHIT,FLNB,FN1,G6PD,GAD2,GALM,GALNT10,GART,GC,GGPS1,GRHPR,GRK2,GRK6,GSTT2/GSTT2B,GTF2F2,GTF2I,HADH,HASPIN,HAT1,HBB,HBEGF,HCK,HDAC7,HDX,HIBCH,HK2,HLA,DRB1,HMBOX1,HMGB3,HMGCS1,HMOX1,HNRNPA2B1,HPD,HSCB,HSD17B10,HSD17B4,HSP90AB1,HTR1A,IDE,IFNG,IFNGR1,IGF1R,IMPA2,IMPDH1,IQUB,ITGB3,ITPA,ITPK1,ITPKC,ITSN1,KIF11,KLK1,KMT5A,KYNU,LRBA,MAGEA4,MAGI2,MAP3K3,MAPK6,MARF1,MARK1,MATK,MDH1,MECR,MEMO1,MMP1,MMP2,MSH2,MSN,MTAP,MUC1,MYBPC1,MYBPC3,MYSM1,NAE1,NBEA,NCBP1,NCOA3,NDC80,NDUFAB1,NF2,NFATC1,NME3,NMRK1,NMT1,NOTCH1,NR1I3,NSUN5,NT5C3A,NUP214,OAT,ODC1,OXSR1,PA2G4,PAFAH1B2,PAH,PANK1,PAPSS1,PARN,PARP3,PATJ,PC,PCYT2,PDF,PDHA1,PDK2,PDK3,PDK4,PEX5,PGC,PGK1,PHF24,PIP4K2C,PNMT,POT1,POU2F1,PPARG,PPP2R1A,PRKCB,PSAP,PTK2B,PTPRE,PTPRF,PUS10,PYGL,RAB26,RAC1,RARA,RARG,RBBP6,RBM39,RBP5,RGS6,RND1,RPA3,RRM1,RTN4IP1,RUVBL1,S100A6,SART3,SEC23A,SEM1,SEMA4D,SENP2,SERPINA5,SF3B6,SFMBT2,SFN,SH2B2,SH3YL1,SKP1,SLC5A1,SLK,SMAP1,SMG5,SMG7,SMTN,SMURF2,SNX1,SNX22,SPEF1,STARD13,STK16,STK4,SUB1,SULT1A1,SYT13,TAF13,THBS2,TIMM9,TJP1,TLR4,TNFAIP8L2,TRIM21,TTR,TXNDC12,UEVLD,USP19,UTRN,VAV3,VEGFB,VPS26A,WEE1,WHRN,WWP1,XIAP,XRCC6,YWHAH,YWHAQ,YY1,ZBTB43,ZCWPW1,ZEB2,ZFP28,ZIC3,ZNF292,ZNF462
67	Urinary tract cancer	Cancer,Organismal Injury and Abnormalities,Renal and Urological Disease	8.13E,11	94	ACACB,ALB,ALK,AOC3,AR,ARHGAP5,ASAP1,ATXN3,B2M,BCR,BMP1,BTK,BTRC,CANT1,CSF2RB,CSTF2,CTCF,CUL1,CYP17A1,DGKA,DHPS,DOK2,ECHS1,EP300,EPHA3,EPS8L2,ERBB4,ESR1,FAP,FN1,G6PD,GART,GGPS1,GRHPR,HBB,HIBCH,HLA,DRB1,HMOX1,HNRNPA2B1,HSD17B4,HSP90AB1,IGF1R,IMPDH1,ITGB3,KIF11,KLK1,KMT5A,KYNU,MAGEA4,MAGI2,MARF1,MMP2,MSH2,MTAP,MUC1,MYBPC1,NCOA3,NF2,NOTCH1,NR1I3,NT5C3A,NUP214,ODC1,PA2G4,PAH,PANK1,PDK4,PPARG,PPP2R1A,PRKCB,PSAP,PTK2B,PTPRE,PTPRF,PYGL,RARA,RARG,RBBP6,RBM39,RBP5,RRM1,SLC5A1,SMG7,SMTN,SULT1A1,THBS2,TJP1,TLR4,UTRN,XIAP,ZCWPW1,ZEB2,ZNF292,ZNF462
68	Urinary tract tumor	Cancer,Organismal Injury and Abnormalities,Renal and Urological Disease	8.77E,11	95	ACACB,ALB,ALK,AOC3,AR,ARHGAP5,ASAP1,ATXN3,B2M,BCR,BMP1,BTK,BTRC,CANT1,CSF2RB,CSTF2,CTCF,CUL1,CYP17A1,DGKA,DHPS,DOK2,ECHS1,EP300,EPHA3,EPS8L2,ERBB4,ESR1,FAP,FN1,G6PD,GART,GGPS1,GRHPR,HBB,HIBCH,HLA,DRB1,HMOX1,HNRNPA2B1,HSD17B4,HSP90AB1,IGF1R,IMPDH1,ITGB3,KIF11,KLK1,KMT5A,KYNU,MAGEA4,MAGI2,MARF1,MMP2,MSH2,MTAP,MUC1,MYBPC1,NCOA3,NF2,NOTCH1,NR1I3,NT5C3A,NUP214,ODC1,PA2G4,PAH,PANK1,PDK4,PPARG,PPP2R1A,PRKCB,PSAP,PTK2B,PTPRE,PTPRF,PYGL,RARA,RARG,RBBP6,RBM39,RBP5,RRM1,SLC5A1,SMG7,SMTN,SULT1A1,THBS2,TJP1,TLR4,UTRN,XIAP,YY1,ZCWPW1,ZEB2,ZNF292,ZNF462
69	Formation of solid tumor	Cancer,Organismal Injury and Abnormalities	9.85E,11	259	AASDHPPT,ACACB,ACADSB,ACADVL,ACBD7,ACVR2B,ACY1,ADH1C,ADH7,ALB,ALK,ALOX12,AMY1C (includes others),AOC3,APEX1,APPL1,AR,ARF6,ARHGAP5,ARHGEF1,ASAP1,ATP7B,ATXN3,B2M,B3GAT2,BCR,BMP1,BPI,BTK,BTRC,C1R,C8A,CA8,CAMK4,CANT1,CD1A,CDK5R1,CERT1,CHKA,CLCN5,CLK1,CRYM,CSDE1,CSF2RB,CSNK1G1,CSNK1G2,CSTF2,CTCF,CUL1,CYP17A1,CYP2E1,CYP7A1,DAPK2,DCTD,DCXR,DFFA,D

					GKA,DHPS,DMC1,DOK2,DPP4,DSG2,DTYMK,ECHS1,EP300,EPHA3,EPS8L2,ERBB4,ESR1,ETF1,EXOSC9,F2,F3,FABP3,FABP7,FAP,FAS,FCGR2A,FHIT,FLNB,FN1,G6PD,GAD2,GALNT10,GART,GGPS1,GRHPR,GRK2,GRK6,GTF2I,HADH,HASPIN,HAT1,HBB,HBEGF,HCK,HDAC7,HDX,HIBCH,HK2,HLA,DRB1,HMBOX1,HMGB3,HMGCS1,HMOX1,HNRNPA2B1,HPD,HSD17B10,HSD17B4,HSP90AB1,HTR1A,IDE,IFNG,IFNGR1,IGF1R,IMPDH1,IQUB,ITGB3,ITPA,ITPK1,ITPKC,ITSN1,KIF11,KLK1,KMT5A,KYNU,LRBA,MAGEA4,MAGI2,MAP3K3,MAPK6,MARF1,MARK1,MATK,MDH1,MEMO1,MMP1,MMP2,MSH2,MSN,MTAP,MUC1,MYBPC1,MYBPC3,MYSM1,NAE1,NBEA,NCBP1,NCOA3,NDC80,NF2,NFATC1,NME3,NMRK1,NMT1,NOTCH1,NR1I3,NSUN5,NT5C3A,NUP214,OAT,ODC1,OXSR1,PA2G4,PAFAH1B2,PAH,PANK1,PAPSS1,PARN,PARP3,PATJ,PC,PCYT2,PDHA1,PDK3,PDK4,PEX5,PGC,PGK1,PHF24,PIP4K2C,POT1,POU2F1,PPARG,PPP2R1A,PRKCB,PSAP,PTK2B,PTPRE,PTPRF,PUS10,PYGL,RAC1,RARA,RARG,RBBP6,RBM39,RBP5,RGS6,RND1,RRM1,RTN4IP1,RUVBL1,S100A6,SART3,SEC23A,SEMA4D,SENP2,SERPINA5,SF3B6,SFMBT2,SFN,SH2B2,SH3YL1,SKP1,SLC5A1,SLK,SMAP1,SMG5,SMG7,SMTN,SMURF2,SNX1,SPEF1,STARD13,STK16,STK4,SUB1,SULT1A1,SYT13,TAF13,THBS2,TIMM9,TJP1,TLR4,TNFAIP8L2,TRIM21,TTR,UEVLD,USP19,UTRN,VAV3,VEGFB,VPS26A,WEE1,WHRN,WWP1,XIAP,XRCC6,YWHAH,YWHAQ,YY1,ZBTB43,ZCWPW1,ZEB2,ZFP28,ZIC3,ZNF292,ZNF462
70	Mammary tumor	Cancer,Organismal Injury and Abnormalities,Reproductive System Disease	9.85E,11	108	ACACB,ALB,ALK,ANG,AR,ARHGEF1,ASAP1,BCR,BMP1,BTK,CAMK4,CANT1,CERT1,CLK1,CSDE1,CSF2RB,CSNK1G1,CTCF,CYP17A1,CYP2E1,DAPK2,DPP4,EP300,EPHA3,ERBB4,ESR1,EXOSC9,FABP7,FAP,FAS,FLNB,FN1,GALNT10,GGPS1,GRK2,GRK6,HAT1,HBB,HBEGF,HCK,HDAC7,HLA,DRB1,HMGCS1,HMOX1,HSP90AB1,IGF1R,IMPDH1,ITGB3,ITPKC,KIF11,KMT5A,MAGEA4,MAPK6,MARF1,MARK1,MMP1,MMP2,MSH2,MSN,MUC1,MYBPC3,NAE1,NBEA,NCOA3,NDC80,NF2,NFATC1,NOTCH1,ODC1,OXSR1,PAFAH1B2,PARP3,PC,PDK3,PDK4,PGK1,PHF24,POU2F1,PPARG,PPP2R1A,PRKCB,PTPRE,PTPRF,RAC1,RARA,RARG,RBBP6,RBM39,RRM1,S100A6,SENP2,SERPINA5,SLC5A1,SLK,SMG7,SMTN,SMURF2,SYT13,TLR4,TTR,USP19,UTRN,VAV3,WEE1,YWHAQ,YY1,ZEB2,ZNF462
71	Genital tumor	Cancer,Organismal Injury and Abnormalities,Reproductive System Disease	1.03E,10	205	AASDHPPT,ACACB,ACADSB,ACADVL,ACBD7,ACP3,ACY1,ALB,ALK,ANG,ANXA6,AOC3,APEX1,APPL1,AR,ARF6,ARHGAP5,ARHGEF1,ASAP1,ATP7B,B2M,B3GAT2,BCR,BTK,BTRC,C1R,C8A,CA8,CANT1,CD1A,CD74,CLCN5,CLK1,CSDE1,CSF2RB,CSNK1G1,CSTF2,CTCF,CUL1,CYP17A1,CYP2E1,CYP7A1,DCTD,DHPS,DMC1,DOK2,DPP4,DTYMK,EP300,EPHA3,EPS8L2,ERBB4,ESR1,ETF1,F2,F3,FABP7,FAP,FAS,FCGR2A,FLNB,FN1,G6PD,GAD2,GALM,GALNT10,GART,GC,GGPS1,GRHPR,GRK2,GTF2I,HASPIN,HBB,HBEGF,HCK,HDAC7,HDX,HLA,DRB1,HMBOX1,HMGB3,HMOX1,HNRNPA2B1,HPD,HSD17B10,HSD17B4,HSP90AB1,HTR1A,IFNG,IFNGR1,IGF1R,IMPDH1,IQUB,ITGB3,ITPA,ITPKC,ITSN1,KIF11,KMT5A,LRBA,MAGEA4,MAGI2,MAP3K3,MAPK6,MARF1,MARK1,MATK,MDH1,MEMO1,MMP1,MMP2,MSH2,MSN,MUC1,MYBPC1,MYBPC3,MYSM1,NAE1,NBEA,NCBP1,NCOA3,NF2,NFATC1,NMRK1,NMT1,NOTCH1,NR1I3,NT5C3A,NUP214,OAT,ODC1,OXSR1,PANK1,PAPSS1,PARN,PARP3,PATJ,PC,PDK3,PDK4,PGC,PGK1,PHF24,POU2F1,PPARG,PPP2R1A,PRKCB,PSAP,PTK2B,PTPRE,PTPRF,PYGL,RAC1,RARA,RARG,RBBP6,RBM39,RGS6,RND1,RRM1,RTN4IP1,RUVBL1,S100A6,SART3,SEMA4D,SENP2,SERPINA5,SF3B6,SFMBT2,SFN,SH3YL1,SKP1,SLC5A1,SLK,SMAP1,SMG5,SMTN,SMURF2,SNX1,STK4,SUB1,SULT1A1,SYT13,TAF13,THBS2,TJP1,TLR4,TRIM21,TTR,UEVLD,USP19,UTRN,VAV3,VEGFB,WEE1,WHRN,WWP1,XIAP,YWHAQ,YY1,ZEB2,ZFP28,ZIC3,ZNF292,ZNF462
72	Nonhematologic malignant neoplasm	Cancer,Organismal Injury and Abnormalities	1.16E,10	284	AASDHPPT,ACACB,ACADSB,ACADVL,ACAT2,ACBD7,ACSM2A,ACVR2B,ACY1,ADH1C,ADH7,ALB,ALK,ALOX12,AMY1C (includes others),ANG,ANXA6,AOC3,APEX1,APPL1,AR,ARF6,ARHGAP5,ARHGEF1,ASAP1,ATP7B,ATXN3,B2M,B3GAT2,B3GAT3,BCR,BMP1,BPI,BTK,BTRC,C1R,C8A,CA8,CAMK4,CANT1,CD1A,CD74,CDK5R1,CERT1,CHKA,CIAO1,CLCN5,CLK1,CRYM,CSDE1,CSF2RB,CSNK1G1,CSNK1G2,CSTF2,CTCF,CUL1,CYP17A1,CYP2E1,CYP2R1,CYP7A1,DAPK2,DCTD,DCXR,DFFA,DGKA,DHPS,DMC1,DOK2,DPP4,DSG2,DTYMK,ECHS1,ECI2,EP300,EPHA3,EPS8L2,ERBB4,ERI1,ESR1,ETF1,EXOSC9,F2,F3,FABP3,FABP7,FAP,FAS,FCGR2A,FHIT,FLNB,FN1,G6PD,GAD2,GALM,GALNT10,GART,GC,GGPS1,GRHPR,GRK2,GRK6,GSTT2/GSTT2B,GTF2F2,GTF2I,HADH,HASPIN,HAT1,HBB,HBEGF,HCK,HDAC7,HDX,HIBCH,HK2,HLA,DRB1,HMBOX1,HMGB3,HMGCS1,HMOX1,HNRNPA2B1,HPD,HSCB,HSD17B10,HSD17B4,HSP90AB1,HTR1A,IDE,IFNG,IFNGR1,IGF1R,IMPA2,IMPDH1,IQUB,ITGB3,ITPA,ITPK1,ITPKC,ITSN1,KIF11,KLK1,KMT5A,KYNU,LRBA,MAGEA4,MAGI2,MAP3K3,MAPK6,MARF1,MARK1,MATK,MDH1,MECR,MEMO1,MMP1,MMP2,MSH2,MSN,MTAP,MUC1,MYBPC1,MYBPC3,MYSM1,NAE1,NBEA,NCBP1,NCOA3,NDC80,NDUFAB1,NF2,NFATC1,NME3,NMRK1,NMT1,NOTCH1,NR1I3,NSUN5,NT5C3A,NUP214,OAT,ODC1,OXSR1,PA2G4,PAFAH1B2,PAH,PANK1,PAPSS1,PARN,PARP3,PATJ,

					PC,PCYT2,PDF,PDHA1,PDK2,PDK3,PDK4,PEX5,PGC,PGK1,PHF24,PIP4K2C,PNMT,POT1,POU2F1,PPARG,PPP2R1A,PRKCB,PSAP,PTK2B,PTPRE,PTPRF,PUS10,PYGL,RAB26,RAC1,RARA,RARG,RBBP6,RBM39,RBP5,RGS6,RND1,RPA3,RRM1,RTN4IP1,RUVBL1,SART3,SEC23A,SEM1,SEMA4D,SENP2,SERPINA5,SF3B6,SFMBT2,SFN,SH2B2,SH3YL1,SKP1,SLC5A1,SLK,SMAP1,SMG5,SMG7,SMTN,SMURF2,SNX1,SNX22,SPEF1,STARD13,STK16,STK4,SUB1,SULT1A1,SYT13,TAF13,THBS2,TIMM9,TJP1,TLR4,TNFAIP8L2,TRIM21,TTR,TXNDC12,UEVLD,USP19,UTRN,VAV3,VEGFB,VPS26A,WEE1,WHRN,WWP1,XIAP,XRCC6,YWHAH,YWHAQ,YY1,ZBTB43,ZCWPW1,ZEB2,ZFP28,ZIC3,ZNF292,ZNF462
73	Endocrine cancer	Cancer,Endocrine System Disorders,Organismal Injury and Abnormalities	1.22E,10	244	AASDHPPT,ACACB,ACADSB,ACADVL,ACAT2,ACBD7,ACVR2B,ACY1,ADH7,ALK,ALOX12,AMY1C (includes others),ANXA6,AOC3,APPL1,AR,ARHGAP5,ARHGEF1,ASAP1,ATP7B,ATXN3,B2M,B3GAT2,B3GAT3,BCR,BMP1,BPI,BTK,BTRC,C8A,CA8,CANT1,CD1A,CHKA,CIAO1,CLCN5,CLK1,CRYM,CSDE1,CSF2RB,CSNK1G1,CSNK1G2,CSTF2,CTCF,CUL1,CYP17A1,CYP2E1,CYP2R1,CYP7A1,DAPK2,DCTD,DCXR,DFFA,DGKA,DHPS,DMC1,DOK2,DPP4,DSG2,DTYMK,EP300,EPS8L2,ERBB4,ERI1,ESR1,ETF1,F2,F3,FAP,FAS,FLNB,FN1,G6PD,GAD2,GALM,GALNT10,GART,GC,GRHPR,GRK2,GRK6,GSTT2/GSTT2B,GTF2F2,GTF2I,HADH,HASPIN,HAT1,HBEGF,HCK,HDAC7,HDX,HIBCH,HK2,HLA,DRB1,HMBOX1,HMGB3,HMGCS1,HMOX1,HNRNPA2B1,HPD,HSCB,HSD17B4,HSP90AB1,IDE,IFNGR1,IGF1R,IMPA2,IMPDH1,IQUB,ITGB3,ITPK1,ITPKC,ITSN1,KIF11,KLK1,KMT5A,KYNU,LRBA,MAGEA4,MAGI2,MAP3K3,MAPK6,MARF1,MARK1,MATK,MECR,MEMO1,MMP1,MMP2,MSH2,MSN,MTAP,MUC1,MYBPC1,MYBPC3,MYSM1,NAE1,NBEA,NCBP1,NCOA3,NDC80,NDUFAB1,NF2,NFATC1,NME3,NMRK1,NOTCH1,NR1I3,NT5C3A,NUP214,OAT,ODC1,OXSR1,PA2G4,PAFAH1B2,PAH,PANK1,PAPSS1,PARN,PARP3,PATJ,PC,PCYT2,PDF,PDHA1,PDK3,PDK4,PEX5,PGK1,PHF24,PIP4K2C,PNMT,POT1,POU2F1,PPARG,PPP2R1A,PRKCB,PSAP,PTK2B,PTPRE,PTPRF,PYGL,RAB26,RAC1,RARA,RARG,RBBP6,RBM39,RGS6,RND1,RPA3,RRM1,RTN4IP1,RUVBL1,SART3,SEC23A,SEM1,SEMA4D,SENP2,SERPINA5,SFMBT2,SH2B2,SH3YL1,SKP1,SLC5A1,SLK,SMAP1,SMG5,SMG7,SMTN,SMURF2,SNX1,SNX22,SPEF1,STARD13,STK4,SUB1,SULT1A1,SYT13,TAF13,THBS2,TJP1,TLR4,TRIM21,TTR,TXNDC12,UEVLD,USP19,UTRN,VAV3,VPS26A,WEE1,WHRN,WWP1,XRCC6,YWHAQ,YY1,ZBTB43,ZCWPW1,ZEB2,ZFP28,ZIC3,ZNF292,ZNF462
74	Clear cell neoplasm	Cancer,Organismal Injury and Abnormalities	1.37E,10	53	ACACB,ALB,ALK,AR,ASAP1,ATXN3,BTRC,CANT1,CSF2RB,CTCF,CYP17A1,ECHS1,EP300,EPHA3,ERBB4,FAP,FN1,GAD2,GRHPR,HBB,HLA,DRB1,HSD17B4,HSP90AB1,IGF1R,IMPDH1,ITGB3,KIF11,KLK1,MMP2,MUC1,MYBPC1,NCOA3,NF2,NOTCH1,NR1I3,NUP214,PANK1,PARP3,PDK4,PPP2R1A,PTPRE,PYGL,RARA,RBP5,RRM1,SMG7,SMTN,SULT1A1,TJP1,TLR4,ZCWPW1,ZNF292,ZNF462
75	Gonadal tumor	Cancer,Endocrine System Disorders,Organismal Injury and Abnormalities,Reproductive System Disease	1.38E,10	82	ALB,ALK,ANG,AOC3,AR,ARF6,ARHGEF1,ASAP1,B2M,BCR,CA8,CD74,CLK1,CSF2RB,CSNK1G1,CSTF2,CTCF,DPP4,EP300,EPHA3,ESR1,F2,FAP,FAS,FN1,GAD2,GART,GC,HBEGF,HCK,HNRNPA2B1,HSP90AB1,HTR1A,IFNGR1,IMPDH1,ITPA,ITSN1,MAGEA4,MAGI2,MAP3K3,MMP1,MMP2,MSH2,MUC1,NBEA,NCOA3,NF2,NOTCH1,NR1I3,NT5C3A,ODC1,PARP3,PC,PDK4,PGC,POU2F1,PPARG,PPP2R1A,PRKCB,PTPRF,PYGL,RAC1,RARG,RBM39,RRM1,RTN4IP1,SERPINA5,SFMBT2,SH3YL1,SKP1,SMTN,SYT13,TAF13,THBS2,TLR4,TTR,UTRN,VAV3,VEGFB,WWP1,XIAP,ZEB2
76	Signal transduction	Cell,To,Cell Signaling and Interaction	1.39E,10	47	ACVR2B,ALK,APPL1,AR,ARHGEF1,BCR,BTK,BTRC,CAMK4,CSNK1G2,DAPK2,DOK2,ERBB4,ESR1,F2,FAS,FLNB,GRK2,GRK6,HASPIN,HBEGF,HLA,DRB1,HMOX1,HTR1A,IFNG,IFNGR1,IGF1R,ITPK1,ITSN1,MAP3K3,MAPK6,MARK1,NAE1,NFATC1,NR1I3,OXSR1,PPARG,PSAP,PTK2B,RAC1,RARA,RGS6,S100A6,SFN,STK4,THBS2,VAV3
77	Incidence of tumor	Cancer,Organismal Injury and Abnormalities	1.51E,10	238	AASDHPPT,ACACB,ACADSB,ACADVL,ACBD7,ACY1,ADH1C,ADH7,ALB,ALK,ALOX12,AOC3,APEX1,APPL1,AR,ARF6,ARHGAP5,ARHGEF1,ASAP1,ATP7B,ATXN3,B2M,B3GAT2,BCR,BMP1,BPI,BTK,BTRC,C1R,C8A,CA8,CAMK4,CANT1,CD1A,CDK5R1,CERT1,CHKA,CLCN5,CLK1,CSDE1,CSF2RB,CSNK1G1,CSNK1G2,CSTF2,CTCF,CUL1,CYP17A1,CYP2E1,CYP7A1,DAPK2,DCTD,DCXR,DFFA,DGKA,DHPS,DMC1,DOK2,DPP4,DSG2,DTYMK,ECHS1,EP300,EPHA3,ERBB4,ESR1,ETF1,EXOSC9,F2,F3,FABP3,FABP7,FAP,FAS,FCGR2A,FHIT,FLNB,FN1,G6PD,GAD2,GALNT10,GART,GGPS1,GRHPR,GRK2,GRK6,GTF2I,HASPIN,HBB,HBEGF,HCK,HDAC7,HDX,HIBCH,HLA,DRB1,HMBOX1,HMGB3,HMOX1,HNRNPA2B1,HPD,HSD17B10,HSD17B4,HSP90AB1,HTR1A,IDE,IFNG,IFNGR1,IGF1R,IMPDH1,IQUB,ITGB3,ITPA,ITPKC,ITSN1,KIF11,KLK1,KMT5A,KYNU,LRBA,MAGEA4,MAGI2,MAP3K3,MAPK6,MARF1,MARK1,MATK,MDH1,MEMO1,MMP1,MMP2,MSH2,MSN,M

					TAP,MUC1,MYBPC1,MYBPC3,MYSM1,NAE1,NBEA,NCBP1,NCOA3,NDC80,NF2,NFATC1,NMRK1,NMT1,NOTCH1,NR1I3,NT5C3A,NUP214,OAT,ODC1,OXSR1,PA2G4,PAH,PANK1,PAPSS1,PARN,PARP3,PATJ,PC,PCYT2,PDK3,PDK4,PEX5,PGC,PGK1,PHF24,PIP4K2C,POU2F1,PPARG,PPP2R1A,PRKCB,PTK2B,PTPRE,PTPRF,PUS10,PYGL,RAC1,RARA,RARG,RBBP6,RBM39,RBP5,RGS6,RND1,RRM1,RTN4IP1,RUVBL1,S100A6,SART3,SEC23A,SEMA4D,SENP2,SERPINA5,SF3B6,SFMBT2,SFN,SH2B2,SH3YL1,SKP1,SLC5A1,SLK,SMAP1,SMG5,SMG7,SMTN,SMURF2,SNX1,SPEF1,STARD13,STK16,STK4,SUB1,SULT1A1,SYT13,TAF13,THBS2,TJP1,TLR4,TRIM21,TTR,UEVLD,USP19,UTRN,VAV3,VEGFB,WEE1,WHRN,WWP1,XIAP,XRCC6,YY1,ZCWPW1,ZEB2,ZFP28,ZIC3,ZNF292,ZNF462
78	Metastatic carcinoma	Cancer,Organismal Injury and Abnormalities	1.53E,10	22	ALB,ALK,AR,B2M,CYP17A1,ERBB4,ESR1,F3,FAS,FN1,GART,GGPS1,HSP90AB1,IGF1R,MMP2,MUC1,NR1I3,PARP3,PGC,PTK2B,RAC1,RRM1
79	Cell death of cancer cells	Cancer,Cell Death and Survival,Organismal Injury and Abnormalities,Tumor Morphology	1.55E,10	19	ALB,AR,ATP7B,B2M,BTK,CD74,FAS,FHIT,GRK6,HMOX1,IFNG,IGF1R,MUC1,NCOA3,PPARG,PTK2B,RAC1,RARA,XIAP
80	Mature T,cell or NK,cell neoplasm	Cancer,Hematological Disease,Organismal Injury and Abnormalities	1.65E,10	36	ALK,B2M,C1R,CD1A,CLK1,CTCF,DPP4,EP300,EPHA3,ERBB4,FAS,FHIT,FN1,GTF2I,HCK,HDAC7,HNRNPA2B1,HSP90AB1,IMPDH1,MMP1,MMP2,MUC1,NFATC1,NOTCH1,NT5C3A,ODC1,POT1,POU2F1,PRKCB,PTPRE,RARA,RARG,RRM1,TAF13,TJP1,TLR4
81	Malignant solid tumor	Cancer,Organismal Injury and Abnormalities	1.74E,10	286	AASDHPPT,ACACB,ACADSB,ACADVL,ACAT2,ACBD7,ACP3,ACSM2A,ACVR2B,ACY1,ADH1C,ADH7,ALB,ALK,ALOX12,AMY1C (includes others),ANG,ANXA6,AOC3,APEX1,APPL1,AR,ARF6,ARHGAP5,ARHGEF1,ASAP1,ATP7B,ATXN3,B2M,B3GAT2,B3GAT3,BCR,BMP1,BPI,BTK,BTRC,C1R,C8A,CA8,CAMK4,CANT1,CD1A,CD74,CDK5R1,CERT1,CHKA,CIAO1,CLCN5,CLK1,CRYM,CSDE1,CSF2RB,CSNK1G1,CSNK1G2,CSTF2,CTCF,CUL1,CYP17A1,CYP2E1,CYP2R1,CYP7A1,DAPK2,DCTD,DCXR,DFFA,DGKA,DHPS,DMC1,DOK2,DPP4,DSG2,DTYMK,ECHS1,ECI2,EP300,EPHA3,EPS8L2,ERBB4,ERI1,ESR1,ETF1,EXOSC9,F2,F3,FABP3,FABP7,FAP,FAS,FCGR2A,FHIT,FLNB,FN1,G6PD,GAD2,GALM,GALNT10,GART,GC,GGPS1,GRHPR,GRK2,GRK6,GSTT2/GSTT2B,GTF2F2,GTF2I,HADH,HASPIN,HAT1,HBB,HBEGF,HCK,HDAC7,HDX,HIBCH,HK2,HLA,DRB1,HMBOX1,HMGB3,HMGCS1,HMOX1,HNRNPA2B1,HPD,HSCB,HSD17B10,HSD17B4,HSP90AB1,HTR1A,IDE,IFNG,IFNGR1,IGF1R,IMPA2,IMPDH1,IQUB,ITGB3,ITPA,ITPK1,ITPKC,ITSN1,KIF11,KLK1,KMT5A,KYNU,LRBA,MAGEA4,MAGI2,MAP3K3,MAPK6,MARF1,MARK1,MATK,MDH1,MECR,MEMO1,MMP1,MMP2,MSH2,MSN,MTAP,MUC1,MYBPC1,MYBPC3,MYSM1,NAE1,NBEA,NCBP1,NCOA3,NDC80,NDUFAB1,NF2,NFATC1,NME3,NMRK1,NMT1,NOTCH1,NR1I3,NSUN5,NT5C3A,NUP214,OAT,ODC1,OXSR1,PA2G4,PAFAH1B2,PAH,PANK1,PAPSS1,PARN,PARP3,PATJ,PC,PCYT2,PDF,PDHA1,PDK2,PDK3,PDK4,PEX5,PGC,PGK1,PHF24,PIP4K2C,PNMT,POT1,POU2F1,PPARG,PPP2R1A,PRKCB,PSAP,PTK2B,PTPRE,PTPRF,PUS10,PYGL,RAB26,RAC1,RARA,RARG,RBBP6,RBM39,RBP5,RGS6,RND1,RPA3,RRM1,RTN4IP1,RUVBL1,S100A6,SART3,SEC23A,SEM1,SEMA4D,SENP2,SERPINA5,SF3B6,SFMBT2,SFN,SH2B2,SH3YL1,SKP1,SLC5A1,SLK,SMAP1,SMG5,SMG7,SMTN,SMURF2,SNX1,SNX22,SPEF1,STARD13,STK16,STK4,SUB1,SULT1A1,SYT13,TAF13,THBS2,TIMM9,TJP1,TLR4,TNFAIP8L2,TRIM21,TTR,TXNDC12,UEVLD,USP19,UTRN,VAV3,VEGFB,VPS26A,WEE1,WHRN,WWP1,XIAP,XRCC6,YWHAH,YWHAQ,YY1,ZBTB43,ZCWPW1,ZEB2,ZFP28,ZIC3,ZNF292,ZNF462
82	Cell viability of tumor cell lines	Cell Death and Survival	1.75E,10	53	ACVR2B,ALB,ALK,ALOX12,APEX1,AR,ATP7B,BCR,BTK,BTRC,CHKA,DGKA,DOK2,DPP4,EP300,ERBB4,ESR1,FAS,FHIT,FN1,GRK2,GRK6,HBB,HBEGF,HCK,HK2,HMOX1,HSP90AB1,IFNG,IGF1R,ITGB3,KIF11,MSH2,NAE1,NDC80,NME3,NOTCH1,NT5C3A,PDHA1,PPP2R1A,PTK2B,PTPRE,RAC1,RBM39,RRM1,RUVBL1,S100A6,SEM1,SFN,TRIM21,WEE1,XIAP,XRCC6
83	B,cell non,Hodgkin lymphoma	Cancer,Hematological Disease,Immunological Disease,Organismal Injury and Abnormalities	1.93E,10	51	ALK,ANXA6,ARHGEF1,ATXN3,B2M,BCR,BTK,CLK1,CSDE1,CSF2RB,CSTF2,CTCF,DGKA,ECI2,EP300,EPHA3,ERBB4,FAS,FCGR2A,FN1,GTF2I,HAT1,HDAC7,HMOX1,HSP90AB1,IFNG,IMPDH1,IQUB,ITGB3,MMP2,NCBP1,NFATC1,NOTCH1,PC,PGK1,POT1,PRKCB,PTK2B,PTPRE,PTPRF,RAC1,RRM1,SEM1,SLC5A1,SNX1,THBS2,TXNDC12,VAV3,XIAP,XRCC6,ZNF292
84	Breast or gynecological cancer	Cancer,Organismal Injury and	2.01E,10	194	AASDHPPT,ACACB,ACADSB,ACBD7,ACY1,ALB,ALK,ANG,AOC3,APPL1,AR,ARF6,ARHGEF1,ASAP1,ATP7B,B2M,B3GAT2,BCR,BMP1,BTK,BTRC,C8A,CA8,CAMK4,CANT1,CD1A,CD74,CERT1,CLCN5,CLK1,CSDE1,

		Abnormalities,Reproductive System Disease			CSF2RB,CSNK1G1,CSTF2,CTCF,CUL1,CYP17A1,CYP2E1,CYP7A1,DAPK2,DCTD,DHPS,DMC1,DOK2,DPP4,DTYMK,EP300,EPHA3,ERBB4,ESR1,ETF1,EXOSC9,F2,F3,FABP7,FAP,FAS,FCGR2A,FLNB,FN1,G6PD,GAD2,GALNT10,GART,GC,GGPS1,GRHPR,GRK2,GRK6,GTF2I,HAT1,HBB,HBEGF,HCK,HDAC7,HDX,HLA,DRB1,HMBOX1,HMGB3,HMGCS1,HMOX1,HNRNPA2B1,HPD,HSD17B4,HSP90AB1,HTR1A,IFNGR1,IGF1R,IMPDH1,IQUB,ITGB3,ITPA,ITPKC,ITSN1,KIF11,KMT5A,LRBA,MAGEA4,MAGI2,MAP3K3,MAPK6,MARF1,MARK1,MDH1,MEMO1,MMP1,MMP2,MSH2,MSN,MUC1,MYBPC3,MYSM1,NAE1,NBEA,NCBP1,NCOA3,NDC80,NF2,NFATC1,NMRK1,NMT1,NOTCH1,NR1I3,NT5C3A,NUP214,OAT,ODC1,OXSR1,PAFAH1B2,PANK1,PARN,PARP3,PATJ,PC,PDK3,PDK4,PGC,PGK1,PHF24,POU2F1,PPARG,PPP2R1A,PRKCB,PTPRE,PTPRF,PYGL,RAC1,RARA,RARG,RBBP6,RBM39,RGS6,RND1,RRM1,RTN4IP1,RUVBL1,S100A6,SART3,SENP2,SERPINA5,SF3B6,SFMBT2,SH3YL1,SKP1,SLC5A1,SLK,SMG5,SMG7,SMTN,SMURF2,SNX1,STK4,SUB1,SULT1A1,SYT13,THBS2,TJP1,TLR4,TRIM21,TTR,UEVLD,USP19,UTRN,VAV3,WEE1,WWP1,XIAP,YWHAQ,YY1,ZEB2,ZFP28,ZIC3,ZNF292,ZNF462
85	T,cell non,Hodgkin lymphoma	Cancer,Hematological Disease,Immunological Disease,Organismal Injury and Abnormalities	2.10E,10	34	ALK,B2M,C1R,CD1A,CLK1,CTCF,DPP4,EP300,EPHA3,ERBB4,FAS,FHIT,FN1,GTF2I,HCK,HDAC7,HNRNPA2B1,HSP90AB1,IMPDH1,MMP1,MMP2,MUC1,NFATC1,NOTCH1,NT5C3A,ODC1,POT1,POU2F1,PRKCB,RARA,RARG,RRM1,TJP1,TLR4
86	Ovarian tumor	Cancer,Endocrine System Disorders,Organismal Injury and Abnormalities,Reproductive System Disease	2.11E,10	79	ALB,ALK,ANG,AOC3,AR,ARF6,ARHGEF1,ASAP1,B2M,BCR,CA8,CD74,CLK1,CSF2RB,CSNK1G1,CSTF2,CTCF,DPP4,EP300,EPHA3,ESR1,F2,FAP,FAS,FN1,GAD2,GART,GC,HBEGF,HCK,HNRNPA2B1,HSP90AB1,HTR1A,IFNGR1,IMPDH1,ITPA,ITSN1,MAGEA4,MAGI2,MAP3K3,MMP1,MMP2,MSH2,MUC1,NBEA,NCOA3,NF2,NOTCH1,NR1I3,NT5C3A,ODC1,PARP3,PC,PDK4,PGC,POU2F1,PPARG,PPP2R1A,PRKCB,PTPRF,PYGL,RBM39,RRM1,RTN4IP1,SERPINA5,SFMBT2,SH3YL1,SKP1,SMTN,SYT13,THBS2,TLR4,TTR,UTRN,VAV3,VEGFB,WWP1,XIAP,ZEB2
87	Recurrent malignant solid tumor	Cancer,Organismal Injury and Abnormalities	2.23E,10	20	ALB,ALK,AR,BTK,CSF2RB,CYP17A1,ERBB4,ESR1,F3,GART,HDAC7,HSP90AB1,IGF1R,IMPDH1,ODC1,PARP3,PTK2B,RARA,RARG,RRM1
88	Frequency of tumor	Cancer,Organismal Injury and Abnormalities	2.31E,10	234	AASDHPPT,ACACB,ACADSB,ACADVL,ACBD7,ACY1,ADH1C,ADH7,ALB,ALK,ALOX12,AOC3,APEX1,APPL1,AR,ARF6,ARHGAP5,ARHGEF1,ASAP1,ATP7B,ATXN3,B2M,B3GAT2,BCR,BMP1,BPI,BTK,BTRC,C1R,C8A,CA8,CAMK4,CANT1,CD1A,CDK5R1,CERT1,CHKA,CLCN5,CLK1,CSDE1,CSF2RB,CSNK1G1,CSNK1G2,CSTF2,CTCF,CUL1,CYP17A1,CYP2E1,CYP7A1,DAPK2,DCTD,DCXR,DFFA,DGKA,DHPS,DOK2,DPP4,DSG2,DTYMK,ECHS1,EP300,EPHA3,ERBB4,ESR1,ETF1,EXOSC9,F2,F3,FABP3,FABP7,FAP,FAS,FCGR2A,FHIT,FLNB,FN1,G6PD,GAD2,GALNT10,GART,GGPS1,GRHPR,GRK2,GRK6,GTF2I,HASPIN,HBB,HBEGF,HCK,HDAC7,HDX,HIBCH,HLA,DRB1,HMBOX1,HMGB3,HNRNPA2B1,HPD,HSD17B4,HSP90AB1,HTR1A,IDE,IFNG,IFNGR1,IGF1R,IMPDH1,IQUB,ITGB3,ITPA,ITPKC,ITSN1,KIF11,KLK1,KMT5A,KYNU,LRBA,MAGEA4,MAGI2,MAP3K3,MAPK6,MARF1,MARK1,MATK,MDH1,MEMO1,MMP1,MMP2,MSH2,MSN,MTAP,MUC1,MYBPC1,MYBPC3,MYSM1,NAE1,NBEA,NCBP1,NCOA3,NDC80,NF2,NFATC1,NMRK1,NMT1,NOTCH1,NR1I3,NT5C3A,NUP214,OAT,ODC1,OXSR1,PA2G4,PAH,PANK1,PAPSS1,PARN,PARP3,PATJ,PC,PCYT2,PDK3,PDK4,PEX5,PGC,PGK1,PHF24,PIP4K2C,POU2F1,PPARG,PPP2R1A,PRKCB,PTK2B,PTPRE,PTPRF,PUS10,PYGL,RAC1,RARA,RARG,RBBP6,RBM39,RBP5,RGS6,RND1,RRM1,RTN4IP1,RUVBL1,SART3,SEC23A,SEMA4D,SENP2,SERPINA5,SF3B6,SFMBT2,SFN,SH2B2,SH3YL1,SKP1,SLC5A1,SLK,SMAP1,SMG5,SMG7,SMTN,SMURF2,SNX1,SPEF1,STARD13,STK16,STK4,SUB1,SULT1A1,SYT13,TAF13,THBS2,TJP1,TLR4,TRIM21,TTR,UEVLD,USP19,UTRN,VAV3,VEGFB,WEE1,WHRN,WWP1,XIAP,XRCC6,YY1,ZCWPW1,ZEB2,ZFP28,ZIC3,ZNF292,ZNF462
89	Breast or pancreatic cancer	Cancer,Organismal Injury and Abnormalities	2.34E,10	160	ACACB,ACAT2,ALB,ALK,ANG,AOC3,APPL1,AR,ARF6,ARHGEF1,ASAP1,ATP7B,B2M,BCR,BMP1,BPI,BTK,BTRC,CA8,CAMK4,CANT1,CD74,CDK5R1,CERT1,CHKA,CLK1,CRYM,CSDE1,CSF2RB,CSNK1G1,CSTF2,CTCF,CYP17A1,CYP2E1,DAPK2,DCTD,DFFA,DPP4,EP300,EPHA3,ERBB4,ESR1,EXOSC9,F2,F3,FABP7,FAP,FAS,FLNB,FN1,GAD2,GALNT10,GART,GC,GGPS1,GRHPR,GRK2,GRK6,GTF2I,HAT1,HBB,HBEGF,HCK,HDAC7,HLA,DRB1,HMGCS1,HMOX1,HNRNPA2B1,HPD,HSP90AB1,HTR1A,IFNGR1,IGF1R,IMPDH1,ITGB3,ITPA,ITPKC,ITSN1,KIF11,KMT5A,LRBA,MAGEA4,MAGI2,MAP3K3,MAPK6,MARF1,MARK1,MMP1,MMP2,MSH2,MSN,MUC1,MYBPC1,MYBPC3,NAE1,NBEA,NCOA3,NDC80,NF2,NFATC1,NOTCH1,NR1I3,NT5C3A,NUP214,ODC1,OXSR1,PA2G4,PAFAH1B2,PARP3,PC,PDHA1,PDK3,PDK4,PGC,PGK1,PHF24,POT1,POU2F1,PPARG,PPP2R1A,PRKCB,PTPRE,PTPRF,PYGL,RAC1,RARA,RARG,RBBP6,RBM39,RRM1,RTN4IP1,S100A6,SENP2,SERP

					INA5,SFMBT2,SFN,SH3YL1,SKP1,SLC5A1,SLK,SMG7,SMTN,SMURF2,SYT13,THBS2,TJP1,TLR4,TTR,USP19,UTRN,VAV3,WEE1,WWP1,XIAP,YWHAQ,YY1,ZEB2,ZFP28,ZNF292,ZNF462
90	Advanced stage carcinoma	Cancer,Organismal Injury and Abnormalities	2.60E,10	26	ALB,ALK,AR,B2M,BCR,CYP17A1,DPP4,ERBB4,ESR1,F3,FAS,FN1,GART,GGPS1,HSP90AB1,IGF1R,MMP2,MUC1,NR1I3,PARP3,PGC,PTK2B,RRM1,SKP1,VAV3,VEGFB
91	Mature T,cell neoplasm	Cancer,Hematological Disease,Immunological Disease,Organismal Injury and Abnormalities	2.68E,10	35	ALK,B2M,C1R,CD1A,CLK1,CTCF,DPP4,EP300,EPHA3,ERBB4,FAS,FHIT,FN1,GTF2I,HCK,HDAC7,HNRNPA2B1,HSP90AB1,MMP1,MMP2,MUC1,NFATC1,NOTCH1,NT5C3A,ODC1,POT1,POU2F1,PRKCB,PTPRE,RARA,RARG,RRM1,TAF13,TJP1,TLR4
92	Endocrine gland tumor	Cancer,Endocrine System Disorders,Organismal Injury and Abnormalities	2.70E,10	245	AASDHPPT,ACACB,ACADSB,ACADVL,ACAT2,ACBD7,ACVR2B,ACY1,ADH7,ALK,ALOX12,AMY1C (includes others),ANXA6,AOC3,APPL1,AR,ARHGAP5,ARHGEF1,ASAP1,ATP7B,ATXN3,B2M,B3GAT2,B3GAT3,BCR,BMP1,BPI,BTK,BTRC,C8A,CA8,CANT1,CD1A,CHKA,CIAO1,CLCN5,CLK1,CRYM,CSDE1,CSF2RB,CSNK1G1,CSNK1G2,CSTF2,CTCF,CUL1,CYP17A1,CYP2E1,CYP2R1,CYP7A1,DAPK2,DCTD,DCXR,DFFA,DGKA,DHPS,DMC1,DOK2,DPP4,DSG2,DTYMK,EP300,EPS8L2,ERBB4,ERI1,ESR1,ETF1,F2,F3,FABP3,FAP,FAS,FLNB,FN1,G6PD,GAD2,GALM,GALNT10,GART,GC,GRHPR,GRK2,GRK6,GSTT2/GSTT2B,GTF2F2,GTF2I,HADH,HASPIN,HAT1,HBEGF,HCK,HDAC7,HDX,HIBCH,HK2,HLA,DRB1,HMBOX1,HMGB3,HMGCS1,HMOX1,HNRNPA2B1,HPD,HSCB,HSD17B4,HSP90AB1,IDE,IFNGR1,IGF1R,IMPA2,IMPDH1,IQUB,ITGB3,ITPK1,ITPKC,ITSN1,KIF11,KLK1,KMT5A,KYNU,LRBA,MAGEA4,MAGI2,MAP3K3,MAPK6,MARF1,MARK1,MATK,MECR,MEMO1,MMP1,MMP2,MSH2,MSN,MTAP,MUC1,MYBPC1,MYBPC3,MYSM1,NAE1,NBEA,NCBP1,NCOA3,NDC80,NDUFAB1,NF2,NFATC1,NME3,NMRK1,NOTCH1,NR1I3,NT5C3A,NUP214,OAT,ODC1,OXSR1,PA2G4,PAFAH1B2,PAH,PANK1,PAPSS1,PARN,PARP3,PATJ,PC,PCYT2,PDF,PDHA1,PDK3,PDK4,PEX5,PGK1,PHF24,PIP4K2C,PNMT,POT1,POU2F1,PPARG,PPP2R1A,PRKCB,PSAP,PTK2B,PTPRE,PTPRF,PYGL,RAB26,RAC1,RARA,RARG,RBBP6,RBM39,RGS6,RND1,RPA3,RRM1,RTN4IP1,RUVBL1,SART3,SEC23A,SEM1,SEMA4D,SENP2,SERPINA5,SFMBT2,SH2B2,SH3YL1,SKP1,SLC5A1,SLK,SMAP1,SMG5,SMG7,SMTN,SMURF2,SNX1,SNX22,SPEF1,STARD13,STK4,SUB1,SULT1A1,SYT13,TAF13,THBS2,TJP1,TLR4,TRIM21,TTR,TXNDC12,UEVLD,USP19,UTRN,VAV3,VPS26A,WEE1,WHRN,WWP1,XRCC6,YWHAQ,YY1,ZBTB43,ZCWPW1,ZEB2,ZFP28,ZIC3,ZNF292,ZNF462
93	Tumorigenesis of epithelial neoplasm	Cancer,Organismal Injury and Abnormalities	2.74E,10	233	AASDHPPT,ACACB,ACADSB,ACADVL,ACBD7,ACY1,ADH1C,ADH7,ALB,ALK,ALOX12,AOC3,APEX1,APPL1,AR,ARF6,ARHGAP5,ARHGEF1,ASAP1,ATP7B,ATXN3,B2M,B3GAT2,BCR,BMP1,BPI,BTK,BTRC,C1R,C8A,CA8,CAMK4,CANT1,CD1A,CDK5R1,CERT1,CHKA,CLCN5,CLK1,CSDE1,CSF2RB,CSNK1G1,CSNK1G2,CSTF2,CTCF,CUL1,CYP17A1,CYP2E1,CYP7A1,DAPK2,DCTD,DCXR,DFFA,DGKA,DHPS,DOK2,DPP4,DSG2,DTYMK,ECHS1,EP300,EPHA3,ERBB4,ESR1,ETF1,EXOSC9,F2,F3,FABP3,FABP7,FAP,FAS,FCGR2A,FHIT,FLNB,FN1,G6PD,GAD2,GALNT10,GART,GGPS1,GRHPR,GRK2,GRK6,GTF2I,HASPIN,HBB,HBEGF,HCK,HDAC7,HDX,HIBCH,HLA,DRB1,HMBOX1,HMGB3,HNRNPA2B1,HPD,HSD17B4,HSP90AB1,HTR1A,IDE,IFNGR1,IGF1R,IMPDH1,IQUB,ITGB3,ITPA,ITPKC,ITSN1,KIF11,KLK1,KMT5A,KYNU,LRBA,MAGEA4,MAGI2,MAP3K3,MAPK6,MARF1,MARK1,MATK,MDH1,MEMO1,MMP1,MMP2,MSH2,MSN,MTAP,MUC1,MYBPC1,MYBPC3,MYSM1,NAE1,NBEA,NCBP1,NCOA3,NDC80,NF2,NFATC1,NMRK1,NMT1,NOTCH1,NR1I3,NT5C3A,NUP214,OAT,ODC1,OXSR1,PA2G4,PAH,PANK1,PAPSS1,PARN,PARP3,PATJ,PC,PCYT2,PDK3,PDK4,PEX5,PGC,PGK1,PHF24,PIP4K2C,POU2F1,PPARG,PPP2R1A,PRKCB,PTK2B,PTPRE,PTPRF,PUS10,PYGL,RAC1,RARA,RARG,RBBP6,RBM39,RBP5,RGS6,RND1,RRM1,RTN4IP1,RUVBL1,SART3,SEC23A,SEMA4D,SENP2,SERPINA5,SF3B6,SFMBT2,SFN,SH2B2,SH3YL1,SKP1,SLC5A1,SLK,SMAP1,SMG5,SMG7,SMTN,SMURF2,SNX1,SPEF1,STARD13,STK16,STK4,SUB1,SULT1A1,SYT13,TAF13,THBS2,TJP1,TLR4,TRIM21,TTR,UEVLD,USP19,UTRN,VAV3,VEGFB,WEE1,WHRN,WWP1,XIAP,XRCC6,YY1,ZCWPW1,ZEB2,ZFP28,ZIC3,ZNF292,ZNF462
94	Clear,cell adenocarcinoma	Cancer,Organismal Injury and Abnormalities	3.21E,10	52	ACACB,ALB,ALK,AR,ASAP1,ATXN3,BTRC,CANT1,CSF2RB,CTCF,CYP17A1,ECHS1,EP300,EPHA3,ERBB4,FAP,FN1,GRHPR,HBB,HLA,DRB1,HSD17B4,HSP90AB1,IGF1R,IMPDH1,ITGB3,KIF11,KLK1,MMP2,MUC1,MYBPC1,NCOA3,NF2,NOTCH1,NR1I3,NUP214,PANK1,PARP3,PDK4,PPP2R1A,PTPRE,PYGL,RARA,RBP5,RRM1,SMG7,SMTN,SULT1A1,TJP1,TLR4,ZCWPW1,ZNF292,ZNF462

95	Organismal death	Organismal Survival	3.21E,10	40	ALK,AOC3,AR,CD74,CLK1,DPP4,ECHS1,ESR1,F2,FABP7,FAP,FHIT,FN1,HBEGF,HSP90AB1,IFNG,IFNGR1,IGF1R,ITGB3,MDH1,MMP2,MSN,MUC1,NAE1,NCOA3,NDC80,NOTCH1,ODC1,OXSR1,PDHA1,PGK1,PPARG,RAC1,RARA,RARG,STARD13,STK4,THBS2,VAV3,ZEB2
96	Mature B cell malignant tumor	Cancer,Hematological Disease,Immunological Disease,Organismal Injury and Abnormalities	3.27E,10	58	ALK,ANXA6,ARHGEF1,ATXN3,B2M,BCR,BTK,CLK1,CSDE1,CSF2RB,CSTF2,CTCF,DGKA,ECI2,EP300,EPHA3,ERBB4,FAS,FCGR2A,FN1,GART,GGPS1,GRHPR,GTF2I,HAT1,HLA,DRB1,HMOX1,HSP90AB1,IFNG,IMPDH1,IQUB,ITGB3,ITPK1,MMP2,NAE1,NCBP1,NDC80,NFATC1,NOTCH1,PC,PGK1,POT1,PRKCB,PTK2B,PTPRE,PTPRF,RAC1,RARA,RRM1,SEM1,SLC5A1,SNX1,THBS2,TXNDC12,VAV3,XIAP,XRCC6,ZNF292
97	Allergy	Immunological Disease	3.51E,10	26	ACADVL,AR,C1R,CD1A,CD74,CSF2RB,DPP4,ESR1,FAS,G6PD,GRK2,HBB,HCK,HLA,DRB1,HTR1A,IDE,IFNG,KLK1,MSN,PDK4,PPARG,PRKCB,PSAP,RUVBL1,TLR4,XRCC6
98	Apoptosis of tumor cells	Cancer,Cell Death and Survival,Organismal Injury and Abnormalities,Tumor Morphology	3.75E,10	17	ALB,AR,B2M,BTK,F2,FAS,FHIT,HBEGF,HMOX1,IFNG,IGF1R,MUC1,NCOA3,NOTCH1,RAC1,RARA,XIAP
99	Epithelial neoplasm	Cancer,Organismal Injury and Abnormalities	3.87E,10	281	AASDHPPT,ACACB,ACADSB,ACADVL,ACAT2,ACBD7,ACSM2A,ACVR2B,ACY1,ADH1C,ADH7,ALB,ALK,ALOX12,AMY1C (includes others),ANXA6,AOC3,APEX1,APPL1,AR,ARF6,ARHGAP5,ARHGEF1,ASAP1,ATP7B,ATXN3,B2M,B3GAT2,B3GAT3,BCR,BMP1,BPI,BTK,BTRC,C1R,C8A,CA8,CAMK4,CANT1,CD1A,CD74,CDK5R1,CERT1,CHKA,CIAO1,CLCN5,CLK1,CRYM,CSDE1,CSF2RB,CSNK1G1,CSNK1G2,CSTF2,CTCF,CUL1,CYP17A1,CYP2E1,CYP2R1,CYP7A1,DAPK2,DCTD,DCXR,DFFA,DGKA,DHPS,DMC1,DOK2,DPP4,DSG2,DTYMK,ECHS1,ECI2,EP300,EPHA3,EPS8L2,ERBB4,ERI1,ESR1,ETF1,EXOSC9,F2,F3,FABP3,FABP7,FAP,FAS,FCGR2A,FHIT,FLNB,FN1,G6PD,GAD2,GALM,GALNT10,GART,GC,GGPS1,GRHPR,GRK2,GRK6,GSTT2/GSTT2B,GTF2F2,GTF2I,HADH,HASPIN,HAT1,HBB,HBEGF,HCK,HDAC7,HDX,HIBCH,HK2,HLA,DRB1,HMBOX1,HMGB3,HMGCS1,HMOX1,HNRNPA2B1,HPD,HSCB,HSD17B10,HSD17B4,HSP90AB1,HTR1A,IDE,IFNG,IFNGR1,IGF1R,IMPA2,IMPDH1,IQUB,ITGB3,ITPA,ITPK1,ITPKC,ITSN1,KIF11,KLK1,KMT5A,KYNU,LRBA,MAGEA4,MAGI2,MAP3K3,MAPK6,MARF1,MARK1,MATK,MDH1,MECR,MEMO1,MMP1,MMP2,MSH2,MSN,MTAP,MUC1,MYBPC1,MYBPC3,MYSM1,NAE1,NBEA,NCBP1,NCOA3,NDC80,NDUFAB1,NF2,NFATC1,NME3,NMRK1,NMT1,NOTCH1,NR1I3,NSUN5,NT5C3A,NUP214,OAT,ODC1,OXSR1,PA2G4,PAFAH1B2,PAH,PANK1,PAPSS1,PARN,PARP3,PATJ,PC,PCYT2,PDF,PDHA1,PDK2,PDK3,PDK4,PEX5,PGC,PGK1,PHF24,PIP4K2C,PNMT,POT1,POU2F1,PPARG,PPP2R1A,PRKCB,PSAP,PTK2B,PTPRE,PTPRF,PUS10,PYGL,RAB26,RAC1,RARA,RARG,RBBP6,RBM39,RBP5,RGS6,RND1,RPA3,RRM1,RTN4IP1,RUVBL1,SART3,SEC23A,SEM1,SEMA4D,SENP2,SERPINA5,SF3B6,SFMBT2,SFN,SH2B2,SH3YL1,SKP1,SLC5A1,SLK,SMAP1,SMG5,SMG7,SMTN,SMURF2,SNX1,SNX22,SPEF1,STARD13,STK16,STK4,SUB1,SULT1A1,SYT13,TAF13,THBS2,TJP1,TLR4,TRIM21,TTR,TXNDC12,UEVLD,USP19,UTRN,VAV3,VEGFB,VPS26A,WEE1,WHRN,WWP1,XIAP,XRCC6,YWHAH,YWHAQ,YY1,ZBTB43,ZCWPW1,ZEB2,ZFP28,ZIC3,ZNF292,ZNF462
100	Stage IV malignant tumor	Cancer,Organismal Injury and Abnormalities	4.22E,10	19	ALB,ALK,AR,BTK,CSF2RB,CYP17A1,ERBB4,ESR1,FCGR2A,FN1,GART,HSP90AB1,IGF1R,PARP3,PTK2B,RARA,RARG,RRM1,VEGFB
101	Malignant genitourinary solid tumor	Cancer,Organismal Injury and Abnormalities	4.34E,10	225	AASDHPPT,ACACB,ACADSB,ACADVL,ACBD7,ACP3,ACY1,ALB,ALK,ANG,ANXA6,AOC3,APEX1,APPL1,AR,ARF6,ARHGAP5,ARHGEF1,ASAP1,ATP7B,ATXN3,B2M,B3GAT2,BCR,BMP1,BTK,BTRC,C1R,C8A,CA8,CAMK4,CANT1,CD1A,CD74,CERT1,CLCN5,CLK1,CSDE1,CSF2RB,CSNK1G1,CSTF2,CTCF,CUL1,CYP17A1,CYP2E1,CYP7A1,DAPK2,DCTD,DGKA,DHPS,DMC1,DOK2,DPP4,DTYMK,ECHS1,EP300,EPHA3,EPS8L2,ERBB4,ESR1,ETF1,EXOSC9,F2,F3,FABP7,FAP,FAS,FCGR2A,FLNB,FN1,G6PD,GAD2,GALM,GALNT10,GART,GC,GGPS1,GRHPR,GRK2,GRK6,GTF2I,HASPIN,HAT1,HBB,HBEGF,HCK,HDAC7,HDX,HIBCH,HLA,DRB1,HMBOX1,HMGB3,HMGCS1,HMOX1,HNRNPA2B1,HPD,HSD17B4,HSP90AB1,HTR1A,IFNG,IFNGR1,IGF1R,IMPDH1,IQUB,ITGB3,ITPA,ITPKC,ITSN1,KIF11,KLK1,KMT5A,KYNU,LRBA,MAGEA4,MAGI2,MAP3K3,MAPK6,MARF1,MARK1,MATK,MDH1,MEMO1,MMP1,MMP2,MSH2,MSN,MTAP,MUC1,MYBPC1,MYBPC3,MYSM1,NAE1,NBEA,NCBP1,NCOA3,NDC80,NF2,NFATC1,NMRK1,NMT1,NOTCH1,NR1I3,NT5C3A,NUP214,OAT,ODC1,OXSR1,PA2G4,PAFAH1B2,PAH,PANK1,PAPSS1,PARN,PARP3,PATJ,PC,PDK3,PDK4,PGC,PGK1,PHF24,POU2F1,PPARG,PPP2R1A,PRKCB,PSAP,PTK2B,PTPRE,PTPRF,PYGL,RAC1,RARA,RARG,RBBP6,RBM39,RBP

					5,RGS6,RND1,RRM1,RTN4IP1,RUVBL1,S100A6,SART3,SEMA4D,SENP2,SERPINA5,SF3B6,SFMBT2,SFN,SH3YL1,SKP1,SLC5A1,SLK,SMAP1,SMG5,SMG7,SMTN,SMURF2,SNX1,STK4,SUB1,SULT1A1,SYT13,TAF13,THBS2,TJP1,TLR4,TRIM21,TTR,UEVLD,USP19,UTRN,VAV3,WEE1,WHRN,WWP1,XIAP,YWHAQ,YY1,ZCWPW1,ZEB2,ZFP28,ZIC3,ZNF292,ZNF462
102	Pelvic cancer	Cancer,Organismal Injury and Abnormalities	4.34E,10	209	AASDHPPT,ACACB,ACADSB,ACADVL,ACBD7,ACP3,ACY1,ADH7,ALB,ALK,ANG,ANXA6,AOC3,APEX1,APPL1,AR,ARF6,ARHGAP5,ARHGEF1,ASAP1,ATP7B,B2M,B3GAT2,BCR,BMP1,BTK,BTRC,C1R,C8A,CA8,CANT1,CD1A,CD74,CLCN5,CLK1,CSDE1,CSF2RB,CSNK1G1,CSTF2,CTCF,CUL1,CYP17A1,CYP2E1,CYP7A1,DCTD,DGKA,DHPS,DMC1,DOK2,DPP4,DTYMK,EP300,EPHA3,EPS8L2,ERBB4,ESR1,ETF1,F2,F3,FABP7,FAP,FAS,FCGR2A,FLNB,FN1,G6PD,GAD2,GALM,GALNT10,GART,GC,GGPS1,GRHPR,GRK2,GTF2I,HASPIN,HBB,HBEGF,HCK,HDAC7,HDX,HLA,DRB1,HMBOX1,HMGB3,HMOX1,HNRNPA2B1,HPD,HSD17B4,HSP90AB1,HTR1A,IFNG,IFNGR1,IGF1R,IQUB,ITGB3,ITPA,ITPKC,ITSN1,KIF11,KMT5A,KYNU,LRBA,MAGEA4,MAGI2,MAP3K3,MAPK6,MARF1,MARK1,MATK,MDH1,MEMO1,MMP1,MMP2,MSH2,MSN,MTAP,MUC1,MYBPC1,MYBPC3,MYSM1,NAE1,NBEA,NCBP1,NCOA3,NF2,NFATC1,NMRK1,NMT1,NOTCH1,NR1I3,NT5C3A,NUP214,OAT,ODC1,OXSR1,PA2G4,PAH,PANK1,PAPSS1,PARN,PARP3,PATJ,PC,PDK3,PDK4,PGC,PGK1,PHF24,POU2F1,PPARG,PPP2R1A,PRKCB,PSAP,PTK2B,PTPRE,PTPRF,PYGL,RAC1,RARA,RARG,RBBP6,RBM39,RGS6,RND1,RRM1,RTN4IP1,RUVBL1,SART3,SEMA4D,SENP2,SERPINA5,SF3B6,SFMBT2,SFN,SH3YL1,SKP1,SLC5A1,SLK,SMAP1,SMG5,SMTN,SMURF2,SNX1,STK4,SUB1,SULT1A1,SYT13,TAF13,THBS2,TJP1,TLR4,TRIM21,TTR,UEVLD,USP19,UTRN,VAV3,VEGFB,WHRN,WWP1,XIAP,YWHAQ,YY1,ZCWPW1,ZEB2,ZFP28,ZIC3,ZNF292,ZNF462
103	Genitourinary tumor	Cancer,Organismal Injury and Abnormalities	4.55E,10	227	AASDHPPT,ACACB,ACADSB,ACADVL,ACBD7,ACP3,ACY1,ALB,ALK,ANG,ANXA6,AOC3,APEX1,APPL1,AR,ARF6,ARHGAP5,ARHGEF1,ASAP1,ATP7B,ATXN3,B2M,B3GAT2,BCR,BMP1,BTK,BTRC,C1R,C8A,CA8,CAMK4,CANT1,CD1A,CD74,CERT1,CLCN5,CLK1,CSDE1,CSF2RB,CSNK1G1,CSTF2,CTCF,CUL1,CYP17A1,CYP2E1,CYP7A1,DAPK2,DCTD,DGKA,DHPS,DMC1,DOK2,DPP4,DTYMK,ECHS1,EP300,EPHA3,EPS8L2,ERBB4,ESR1,ETF1,EXOSC9,F2,F3,FABP7,FAP,FAS,FCGR2A,FLNB,FN1,G6PD,GAD2,GALM,GALNT10,GART,GC,GGPS1,GRHPR,GRK2,GRK6,GTF2I,HASPIN,HAT1,HBB,HBEGF,HCK,HDAC7,HDX,HIBCH,HLA,DRB1,HMBOX1,HMGB3,HMGCS1,HMOX1,HNRNPA2B1,HPD,HSD17B10,HSD17B4,HSP90AB1,HTR1A,IFNG,IFNGR1,IGF1R,IMPDH1,IQUB,ITGB3,ITPA,ITPKC,ITSN1,KIF11,KLK1,KMT5A,KYNU,LRBA,MAGEA4,MAGI2,MAP3K3,MAPK6,MARF1,MARK1,MATK,MDH1,MEMO1,MMP1,MMP2,MSH2,MSN,MTAP,MUC1,MYBPC1,MYBPC3,MYSM1,NAE1,NBEA,NCBP1,NCOA3,NDC80,NF2,NFATC1,NMRK1,NMT1,NOTCH1,NR1I3,NT5C3A,NUP214,OAT,ODC1,OXSR1,PA2G4,PAFAH1B2,PAH,PANK1,PAPSS1,PARN,PARP3,PATJ,PC,PDK3,PDK4,PGC,PGK1,PHF24,POU2F1,PPARG,PPP2R1A,PRKCB,PSAP,PTK2B,PTPRE,PTPRF,PYGL,RAC1,RARA,RARG,RBBP6,RBM39,RBP5,RGS6,RND1,RRM1,RTN4IP1,RUVBL1,S100A6,SART3,SEMA4D,SENP2,SERPINA5,SF3B6,SFMBT2,SFN,SH3YL1,SKP1,SLC5A1,SLK,SMAP1,SMG5,SMG7,SMTN,SMURF2,SNX1,STK4,SUB1,SULT1A1,SYT13,TAF13,THBS2,TJP1,TLR4,TRIM21,TTR,UEVLD,USP19,UTRN,VAV3,VEGFB,WEE1,WHRN,WWP1,XIAP,YWHAQ,YY1,ZCWPW1,ZEB2,ZFP28,ZIC3,ZNF292,ZNF462
104	Renal lesion	Organismal Injury and Abnormalities,Renal and Urological Disease	4.87E,10	64	ACACB,ALB,AR,ASAP1,ATXN3,B2M,BTRC,CANT1,CSF2RB,CTCF,CYP17A1,ECHS1,EP300,EPHA3,ERBB4,ESR1,FAP,FN1,G6PD,GGPS1,GRHPR,HBB,HIBCH,HLA,DRB1,HMOX1,HSD17B4,HSP90AB1,IGF1R,IMPDH1,ITGB3,KIF11,KLK1,MMP2,MSH2,MUC1,MYBPC1,NCOA3,NF2,NOTCH1,NR1I3,NUP214,PANK1,PDK4,PPARG,PPP2R1A,PRKCB,PTPRE,PYGL,RARA,RARG,RBP5,RRM1,SMG7,SMTN,SMURF2,SULT1A1,TJP1,TLR4,UTRN,YY1,ZCWPW1,ZEB2,ZNF292,ZNF462
105	Development of malignant tumor	Cancer,Organismal Injury and Abnormalities	5.34E,10	232	AASDHPPT,ACACB,ACADSB,ACADVL,ACBD7,ACY1,ADH1C,ADH7,ALB,ALK,ALOX12,AOC3,APEX1,APPL1,AR,ARF6,ARHGAP5,ARHGEF1,ASAP1,ATP7B,ATXN3,B2M,B3GAT2,BCR,BMP1,BPI,BTK,BTRC,C1R,C8A,CA8,CAMK4,CANT1,CD1A,CDK5R1,CERT1,CHKA,CLCN5,CLK1,CSDE1,CSF2RB,CSNK1G1,CSNK1G2,CSTF2,CTCF,CUL1,CYP17A1,CYP2E1,CYP7A1,DAPK2,DCTD,DCXR,DFFA,DGKA,DHPS,DOK2,DPP4,DSG2,DTYMK,ECHS1,EP300,EPHA3,ERBB4,ESR1,ETF1,EXOSC9,F2,F3,FABP7,FAP,FAS,FCGR2A,FHIT,FLNB,FN1,G6PD,GAD2,GALNT10,GART,GGPS1,GRHPR,GRK2,GRK6,GTF2I,HASPIN,HBB,HBEGF,HCK,HDAC7,HDX,HIBCH,HLA,DRB1,HMBOX1,HMGB3,HNRNPA2B1,HPD,HSD17B4,HSP90AB1,HTR1A,IDE,IFNG,IFNGR1,IGF1R,IMPDH1,IQUB,ITGB3,ITPA,ITPKC,ITSN1,KIF11,KLK1,KMT5A,KYNU,LRBA,MAGEA4,MAGI2,MAP3K3,MAPK6,MARF1,MARK1,MATK,MDH1,MEMO1,MMP1,MMP2,MSH2,MSN,MTAP,MUC1,MYBPC1,MYBPC3,MYSM1,NAE1,NBEA,NCBP1,NCOA3,NDC80,NF2,NFATC1,NMRK1,NMT1,NOTCH1,NR1I3,NT5C3A,NUP214,OA

					T,ODC1,OXSR1,PA2G4,PAH,PANK1,PAPSS1,PARN,PARP3,PATJ,PC,PCYT2,PDK3,PDK4,PEX5,PGC,PGK1,PHF24,PIP4K2C,POU2F1,PPARG,PPP2R1A,PRKCB,PTK2B,PTPRE,PTPRF,PUS10,PYGL,RAC1,RARA,RARG,RBBP6,RBM39,RBP5,RGS6,RND1,RRM1,RTN4IP1,RUVBL1,SART3,SEC23A,SEMA4D,SENP2,SERPINA5,SF3B6,SFMBT2,SFN,SH2B2,SH3YL1,SKP1,SLC5A1,SLK,SMAP1,SMG5,SMG7,SMTN,SMURF2,SNX1,SPEF1,STARD13,STK16,STK4,SUB1,SULT1A1,SYT13,THBS2,TJP1,TLR4,TRIM21,TTR,UEVLD,USP19,UTRN,VAV3,VEGFB,WEE1,WHRN,WWP1,XIAP,XRCC6,YY1,ZCWPW1,ZEB2,ZFP28,ZIC3,ZNF292,ZNF462
106	Endocrine carcinoma	Cancer,Endocrine System Disorders,Organismal Injury and Abnormalities	6.05E,10	242	AASDHPPT,ACACB,ACADSB,ACADVL,ACAT2,ACBD7,ACVR2B,ACY1,ADH7,ALK,ALOX12,AMY1C (includes others),ANXA6,AOC3,APPL1,AR,ARHGAP5,ARHGEF1,ASAP1,ATP7B,ATXN3,B2M,B3GAT2,B3GAT3,BCR,BMP1,BPI,BTK,BTRC,C8A,CA8,CANT1,CD1A,CHKA,CIAO1,CLCN5,CLK1,CRYM,CSDE1,CSF2RB,CSNK1G1,CSNK1G2,CSTF2,CTCF,CUL1,CYP17A1,CYP2E1,CYP2R1,CYP7A1,DAPK2,DCTD,DCXR,DFFA,DGKA,DHPS,DMC1,DOK2,DPP4,DSG2,DTYMK,EP300,EPS8L2,ERBB4,ERI1,ESR1,ETF1,F2,F3,FAP,FLNB,FN1,G6PD,GAD2,GALM,GALNT10,GART,GC,GRHPR,GRK2,GRK6,GSTT2/GSTT2B,GTF2F2,GTF2I,HADH,HASPIN,HAT1,HBEGF,HCK,HDAC7,HDX,HIBCH,HK2,HLA,DRB1,HMBOX1,HMGB3,HMGCS1,HMOX1,HNRNPA2B1,HPD,HSCB,HSD17B4,HSP90AB1,IDE,IFNGR1,IGF1R,IMPA2,IMPDH1,IQUB,ITGB3,ITPK1,ITPKC,ITSN1,KIF11,KLK1,KMT5A,KYNU,LRBA,MAGEA4,MAGI2,MAP3K3,MAPK6,MARF1,MARK1,MATK,MECR,MEMO1,MMP1,MMP2,MSH2,MSN,MTAP,MUC1,MYBPC1,MYBPC3,MYSM1,NAE1,NBEA,NCBP1,NCOA3,NDC80,NDUFAB1,NF2,NFATC1,NME3,NMRK1,NOTCH1,NR1I3,NT5C3A,NUP214,OAT,ODC1,OXSR1,PA2G4,PAFAH1B2,PAH,PANK1,PAPSS1,PARN,PARP3,PATJ,PC,PCYT2,PDF,PDHA1,PDK3,PDK4,PEX5,PGK1,PHF24,PIP4K2C,PNMT,POT1,POU2F1,PPARG,PPP2R1A,PRKCB,PSAP,PTK2B,PTPRE,PTPRF,PYGL,RAB26,RAC1,RARA,RARG,RBBP6,RBM39,RGS6,RND1,RPA3,RRM1,RTN4IP1,RUVBL1,SART3,SEC23A,SEM1,SEMA4D,SENP2,SERPINA5,SFMBT2,SH2B2,SH3YL1,SKP1,SLC5A1,SLK,SMAP1,SMG5,SMG7,SMTN,SMURF2,SNX1,SNX22,SPEF1,STARD13,STK4,SUB1,SULT1A1,SYT13,THBS2,TJP1,TLR4,TRIM21,TTR,TXNDC12,UEVLD,USP19,UTRN,VAV3,VPS26A,WEE1,WHRN,WWP1,XRCC6,YWHAQ,YY1,ZBTB43,ZCWPW1,ZEB2,ZFP28,ZIC3,ZNF292,ZNF462
107	Ovarian cancer	Cancer,Endocrine System Disorders,Organismal Injury and Abnormalities,Reproductive System Disease	6.13E,10	77	ALB,ALK,ANG,AOC3,AR,ARF6,ARHGEF1,ASAP1,B2M,BCR,CA8,CD74,CLK1,CSF2RB,CSNK1G1,CSTF2,CTCF,DPP4,EP300,EPHA3,ESR1,F2,FAP,FAS,FN1,GAD2,GART,GC,HBEGF,HCK,HNRNPA2B1,HSP90AB1,HTR1A,IFNGR1,ITPA,ITSN1,MAGEA4,MAGI2,MAP3K3,MMP1,MMP2,MSH2,MUC1,NBEA,NCOA3,NF2,NOTCH1,NR1I3,NT5C3A,ODC1,PARP3,PC,PDK4,PGC,POU2F1,PPARG,PPP2R1A,PRKCB,PTPRF,PYGL,RBM39,RRM1,RTN4IP1,SERPINA5,SFMBT2,SH3YL1,SKP1,SMTN,SYT13,THBS2,TLR4,TTR,UTRN,VAV3,WWP1,XIAP,ZEB2
108	Development of carcinoma	Cancer,Organismal Injury and Abnormalities	6.54E,10	231	AASDHPPT,ACACB,ACADSB,ACADVL,ACBD7,ACY1,ADH1C,ADH7,ALB,ALK,ALOX12,AOC3,APEX1,APPL1,AR,ARF6,ARHGAP5,ARHGEF1,ASAP1,ATP7B,ATXN3,B2M,B3GAT2,BCR,BMP1,BPI,BTK,BTRC,C1R,C8A,CA8,CAMK4,CANT1,CD1A,CDK5R1,CERT1,CHKA,CLCN5,CLK1,CSDE1,CSF2RB,CSNK1G1,CSNK1G2,CSTF2,CTCF,CUL1,CYP17A1,CYP2E1,CYP7A1,DAPK2,DCTD,DCXR,DFFA,DGKA,DHPS,DOK2,DPP4,DSG2,DTYMK,ECHS1,EP300,EPHA3,ERBB4,ESR1,ETF1,EXOSC9,F2,F3,FABP7,FAP,FAS,FCGR2A,FHIT,FLNB,FN1,G6PD,GAD2,GALNT10,GART,GGPS1,GRHPR,GRK2,GRK6,GTF2I,HASPIN,HBB,HBEGF,HCK,HDAC7,HDX,HIBCH,HLA,DRB1,HMBOX1,HMGB3,HNRNPA2B1,HPD,HSD17B4,HSP90AB1,HTR1A,IDE,IFNGR1,IGF1R,IMPDH1,IQUB,ITGB3,ITPA,ITPKC,ITSN1,KIF11,KLK1,KMT5A,KYNU,LRBA,MAGEA4,MAGI2,MAP3K3,MAPK6,MARF1,MARK1,MATK,MDH1,MEMO1,MMP1,MMP2,MSH2,MSN,MTAP,MUC1,MYBPC1,MYBPC3,MYSM1,NAE1,NBEA,NCBP1,NCOA3,NDC80,NF2,NFATC1,NMRK1,NMT1,NOTCH1,NR1I3,NT5C3A,NUP214,OAT,ODC1,OXSR1,PA2G4,PAH,PANK1,PAPSS1,PARN,PARP3,PATJ,PC,PCYT2,PDK3,PDK4,PEX5,PGC,PGK1,PHF24,PIP4K2C,POU2F1,PPARG,PPP2R1A,PRKCB,PTK2B,PTPRE,PTPRF,PUS10,PYGL,RAC1,RARA,RARG,RBBP6,RBM39,RBP5,RGS6,RND1,RRM1,RTN4IP1,RUVBL1,SART3,SEC23A,SEMA4D,SENP2,SERPINA5,SF3B6,SFMBT2,SFN,SH2B2,SH3YL1,SKP1,SLC5A1,SLK,SMAP1,SMG5,SMG7,SMTN,SMURF2,SNX1,SPEF1,STARD13,STK16,STK4,SUB1,SULT1A1,SYT13,THBS2,TJP1,TLR4,TRIM21,TTR,UEVLD,USP19,UTRN,VAV3,VEGFB,WEE1,WHRN,WWP1,XIAP,XRCC6,YY1,ZCWPW1,ZEB2,ZFP28,ZIC3,ZNF292,ZNF462
109	Renal tumor	Cancer,Organismal Injury and Abnormalities,Renal and Urological Disease	7.57E,10	63	ACACB,ALB,AR,ASAP1,ATXN3,B2M,BTRC,CANT1,CSF2RB,CTCF,CYP17A1,ECHS1,EP300,EPHA3,ERBB4,ESR1,FAP,FN1,G6PD,GGPS1,GRHPR,HBB,HIBCH,HLA,DRB1,HMOX1,HSD17B4,HSP90AB1,IGF1R,IMPDH1,ITGB3,KIF11,KLK1,MMP2,MSH2,MUC1,MYBPC1,NCOA3,NF2,NOTCH1,NR1I3,NUP214,PANK1,PDK4,PPAR

					G,PPP2R1A,PRKCB,PTPRE,PYGL,RARA,RARG,RBP5,RRM1,SMG7,SMTN,SULT1A1,TJP1,TLR4,UTRN,YY1,ZCWPW1,ZEB2,ZNF292,ZNF462
110	Development of genital tumor	Cancer,Organismal Injury and Abnormalities,Reproductive System Disease	7.77E,10	169	AASDHPPT,ACACB,ACADSB,ACBD7,ACY1,ALB,ALK,AOC3,APPL1,AR,ARF6,ARHGAP5,ARHGEF1,ASAP1,ATP7B,B2M,B3GAT2,BCR,BTK,BTRC,C8A,CA8,CD1A,CLCN5,CLK1,CSDE1,CSF2RB,CSNK1G1,CSTF2,CTCF,CUL1,CYP7A1,DCTD,DHPS,DMC1,DOK2,DPP4,DTYMK,EP300,EPHA3,ERBB4,ESR1,ETF1,F2,F3,FABP7,FAP,FAS,FCGR2A,FLNB,FN1,G6PD,GAD2,GART,GRHPR,GRK2,GTF2I,HBB,HBEGF,HCK,HDAC7,HDX,HMBOX1,HMGB3,HMOX1,HNRNPA2B1,HPD,HSD17B10,HSD17B4,HSP90AB1,HTR1A,IFNG,IGF1R,IQUB,ITGB3,ITPA,ITPKC,ITSN1,KIF11,LRBA,MAGEA4,MAGI2,MAP3K3,MAPK6,MARF1,MARK1,MDH1,MEMO1,MMP1,MMP2,MSH2,MSN,MUC1,MYBPC3,MYSM1,NAE1,NBEA,NCBP1,NCOA3,NF2,NFATC1,NMRK1,NMT1,NOTCH1,NR1I3,NT5C3A,NUP214,OAT,ODC1,PANK1,PARN,PARP3,PATJ,PC,PDK3,PDK4,PGC,PGK1,POU2F1,PPARG,PPP2R1A,PTPRE,PTPRF,PYGL,RAC1,RARA,RARG,RBBP6,RBM39,RGS6,RND1,RRM1,RTN4IP1,RUVBL1,S100A6,SART3,SENP2,SERPINA5,SF3B6,SFMBT2,SH3YL1,SKP1,SLC5A1,SLK,SMG5,SMTN,SMURF2,SNX1,STK4,SUB1,SULT1A1,SYT13,TAF13,THBS2,TJP1,TLR4,TRIM21,UEVLD,UTRN,VAV3,WEE1,WWP1,XIAP,YY1,ZEB2,ZFP28,ZIC3,ZNF292,ZNF462
111	Genitourinary adenocarcinoma	Cancer,Organismal Injury and Abnormalities	8.97E,10	196	AASDHPPT,ACACB,ACADSB,ACADVL,ACBD7,ACY1,ALB,ALK,ANXA6,AOC3,APEX1,APPL1,AR,ARF6,ARHGAP5,ARHGEF1,ASAP1,ATP7B,ATXN3,B2M,B3GAT2,BCR,BTK,BTRC,C8A,CA8,CAMK4,CANT1,CD1A,CLCN5,CLK1,CSDE1,CSF2RB,CSNK1G1,CSTF2,CTCF,CUL1,CYP17A1,CYP2E1,CYP7A1,DAPK2,DCTD,DMC1,DOK2,DPP4,DTYMK,ECHS1,EP300,EPHA3,EPS8L2,ERBB4,ESR1,ETF1,F2,F3,FAP,FCGR2A,FLNB,FN1,G6PD,GAD2,GALM,GALNT10,GART,GC,GGPS1,GRHPR,GRK2,GRK6,GTF2I,HASPIN,HBB,HBEGF,HCK,HDAC7,HDX,HLA,DRB1,HMBOX1,HMGB3,HNRNPA2B1,HPD,HSD17B4,HSP90AB1,HTR1A,IFNG,IFNGR1,IGF1R,IMPDH1,IQUB,ITGB3,ITPA,ITPKC,ITSN1,KIF11,KLK1,KMT5A,LRBA,MAGEA4,MAGI2,MAP3K3,MAPK6,MARF1,MARK1,MATK,MDH1,MEMO1,MMP1,MMP2,MSH2,MSN,MUC1,MYBPC1,MYBPC3,MYSM1,NBEA,NCBP1,NCOA3,NDC80,NF2,NFATC1,NMRK1,NMT1,NOTCH1,NR1I3,NT5C3A,NUP214,OAT,ODC1,OXSR1,PANK1,PAPSS1,PARN,PARP3,PATJ,PC,PDK3,PDK4,PGC,PGK1,PHF24,POU2F1,PPARG,PPP2R1A,PRKCB,PTK2B,PTPRE,PTPRF,PYGL,RAC1,RARA,RARG,RBBP6,RBM39,RBP5,RGS6,RND1,RRM1,RTN4IP1,RUVBL1,SART3,SENP2,SERPINA5,SF3B6,SFMBT2,SFN,SH3YL1,SKP1,SLC5A1,SLK,SMAP1,SMG5,SMG7,SMTN,SMURF2,STK4,SUB1,SULT1A1,SYT13,THBS2,TJP1,TLR4,TRIM21,UEVLD,USP19,UTRN,VAV3,WHRN,WWP1,XIAP,YY1,ZCWPW1,ZEB2,ZFP28,ZIC3,ZNF292,ZNF462
112	Infection by RNA virus	Infectious Diseases	9.48E,10	55	ACACB,ACADSB,ACP3,ADH1C,ADH7,ALB,ALOX12,AR,ARF6,B2M,BCR,BMP1,C1R,C8A,CANT1,CD74,CSF2RB,CTCF,DAPK2,DHPS,DPP4,EP300,ESR1,ETF1,F2,FAS,FCGR2A,FN1,GGPS1,GRK2,HBB,HIBCH,HLA,DRB1,HMGCS1,IFNG,IGF1R,IMPDH1,IQUB,ITGB3,LARS1,NBEA,NF2,NMT1,NUP214,PANK1,PPARG,PRKCB,RARA,RARG,RND1,RRM1,SUB1,TLR4,XIAP,ZNF292
113	Renal cancer	Cancer,Organismal Injury and Abnormalities,Renal and Urological Disease	1.00E,09	62	ACACB,ALB,AR,ASAP1,ATXN3,B2M,BTRC,CANT1,CSF2RB,CTCF,CYP17A1,ECHS1,EP300,EPHA3,ERBB4,ESR1,FAP,FN1,G6PD,GGPS1,GRHPR,HBB,HIBCH,HLA,DRB1,HMOX1,HSD17B4,HSP90AB1,IGF1R,IMPDH1,ITGB3,KIF11,KLK1,MMP2,MSH2,MUC1,MYBPC1,NCOA3,NF2,NOTCH1,NR1I3,NUP214,PANK1,PDK4,PPARG,PPP2R1A,PRKCB,PTPRE,PYGL,RARA,RARG,RBP5,RRM1,SMG7,SMTN,SULT1A1,TJP1,TLR4,UTRN,ZCWPW1,ZEB2,ZNF292,ZNF462
114	Systemic autoimmune syndrome	Immunological Disease	1.26E,09	59	ADH1C,ALB,ALOX12,ANG,AR,ASAP1,B2M,BPI,C1R,CD1A,CD74,CDK5R1,CSF2RB,CYP2E1,CYP2R1,DPP4,ECI2,ESR1,EXOSC9,FAS,FCGR2A,FN1,GC,HCK,HDAC7,HLA,DRB1,HMOX1,IDE,IFNG,IGF1R,IMPDH1,ITPA,MMP1,MMP2,MTAP,MUC1,NF2,PGK1,PPARG,PRKCB,PTK2B,PTPRE,PTPRF,RARA,RARG,RGS6,S100A6,SF3B6,SLC5A1,SMG7,TAF13,TIMM9,TJP1,TLR4,TRIM21,VEGFB,WARS1,XIAP,XRCC6
115	Genital tract cancer	Cancer,Organismal Injury and Abnormalities,Reproductive System Disease	1.32E,09	200	AASDHPPT,ACACB,ACADSB,ACADVL,ACBD7,ACP3,ACY1,ALB,ALK,ANG,ANXA6,AOC3,APEX1,APPL1,AR,ARF6,ARHGAP5,ARHGEF1,ASAP1,ATP7B,B2M,B3GAT2,BCR,BTK,BTRC,C1R,C8A,CA8,CANT1,CD1A,CD74,CLCN5,CLK1,CSDE1,CSF2RB,CSNK1G1,CSTF2,CTCF,CUL1,CYP17A1,CYP2E1,CYP7A1,DCTD,DHPS,DMC1,DOK2,DPP4,DTYMK,EP300,EPHA3,EPS8L2,ERBB4,ESR1,ETF1,F2,F3,FABP7,FAP,FAS,FCGR2A,FLNB,FN1,G6PD,GAD2,GALM,GALNT10,GART,GC,GGPS1,GRHPR,GRK2,GTF2I,HASPIN,HBB,HBEGF,HCK,HDAC7,HDX,HLA,DRB1,HMBOX1,HMGB3,HMOX1,HNRNPA2B1,HPD,HSD17B4,HSP90AB1,HTR1A,IFNG,IFNGR1,IGF1R,IQUB,ITGB3,ITPA,ITPKC,ITSN1,KIF11,KMT5A,LRBA,MAGEA4,MAGI2,MAP3K3,MAPK6,MARF1,MARK1,MATK,MDH1,MEMO1,MMP1,MMP2,MSH2,MSN,MUC1,MYBPC1,MYBPC3,MYSM1,NAE1,NBEA,NC

					BP1,NCOA3,NF2,NFATC1,NMRK1,NMT1,NOTCH1,NR1I3,NT5C3A,NUP214,OAT,ODC1,OXSR1,PANK1,PAPSS1,PARN,PARP3,PATJ,PC,PDK3,PDK4,PGC,PGK1,PHF24,POU2F1,PPARG,PPP2R1A,PRKCB,PSAP,PTK2B,PTPRE,PTPRF,PYGL,RAC1,RARA,RARG,RBBP6,RBM39,RGS6,RND1,RRM1,RTN4IP1,RUVBL1,SART3,SEMA4D,SENP2,SERPINA5,SF3B6,SFMBT2,SFN,SH3YL1,SKP1,SLC5A1,SLK,SMAP1,SMG5,SMTN,SMURF2,SNX1,STK4,SUB1,SULT1A1,SYT13,TAF13,THBS2,TJP1,TLR4,TRIM21,TTR,UEVLD,USP19,UTRN,VAV3,WHRN,WWP1,XIAP,YWHAQ,YY1,ZEB2,ZFP28,ZIC3,ZNF292,ZNF462
116	Advanced extracranial solid tumor	Cancer,Organismal Injury and Abnormalities	1.33E,09	35	ALB,ALK,AOC3,AR,B2M,BCR,BTK,C1R,CYP17A1,DPP4,DSG2,ERBB4,ESR1,F3,FAS,FCGR2A,FN1,GAD2,GART,GGPS1,HMOX1,HSP90AB1,IGF1R,MMP2,MUC1,NR1I3,PARP3,PGC,PTK2B,RAC1,RARG,RRM1,SKP1,VAV3,VEGFB
117	Carcinoma	Cancer,Organismal Injury and Abnormalities	1.63E,09	280	AASDHPPT,ACACB,ACADSB,ACADVL,ACAT2,ACBD7,ACSM2A,ACVR2B,ACY1,ADH1C,ADH7,ALB,ALK,ALOX12,AMY1C (includes others),ANXA6,AOC3,APEX1,APPL1,AR,ARF6,ARHGAP5,ARHGEF1,ASAP1,ATP7B,ATXN3,B2M,B3GAT2,B3GAT3,BCR,BMP1,BPI,BTK,BTRC,C1R,C8A,CA8,CAMK4,CANT1,CD1A,CD74,CDK5R1,CERT1,CHKA,CIAO1,CLCN5,CLK1,CRYM,CSDE1,CSF2RB,CSNK1G1,CSNK1G2,CSTF2,CTCF,CUL1,CYP17A1,CYP2E1,CYP2R1,CYP7A1,DAPK2,DCTD,DCXR,DFFA,DGKA,DHPS,DMC1,DOK2,DPP4,DSG2,DTYMK,ECHS1,ECI2,EP300,EPHA3,EPS8L2,ERBB4,ERI1,ESR1,ETF1,EXOSC9,F2,F3,FABP7,FAP,FAS,FCGR2A,FHIT,FLNB,FN1,G6PD,GAD2,GALM,GALNT10,GART,GC,GGPS1,GRHPR,GRK2,GRK6,GSTT2/GSTT2B,GTF2F2,GTF2I,HADH,HASPIN,HAT1,HBB,HBEGF,HCK,HDAC7,HDX,HIBCH,HK2,HLA,DRB1,HMBOX1,HMGB3,HMGCS1,HMOX1,HNRNPA2B1,HPD,HSCB,HSD17B10,HSD17B4,HSP90AB1,HTR1A,IDE,IFNG,IFNGR1,IGF1R,IMPA2,IMPDH1,IQUB,ITGB3,ITPA,ITPK1,ITPKC,ITSN1,KIF11,KLK1,KMT5A,KYNU,LRBA,MAGEA4,MAGI2,MAP3K3,MAPK6,MARF1,MARK1,MATK,MDH1,MECR,MEMO1,MMP1,MMP2,MSH2,MSN,MTAP,MUC1,MYBPC1,MYBPC3,MYSM1,NAE1,NBEA,NCBP1,NCOA3,NDC80,NDUFAB1,NF2,NFATC1,NME3,NMRK1,NMT1,NOTCH1,NR1I3,NSUN5,NT5C3A,NUP214,OAT,ODC1,OXSR1,PA2G4,PAFAH1B2,PAH,PANK1,PAPSS1,PARN,PARP3,PATJ,PC,PCYT2,PDF,PDHA1,PDK2,PDK3,PDK4,PEX5,PGC,PGK1,PHF24,PIP4K2C,PNMT,POT1,POU2F1,PPARG,PPP2R1A,PRKCB,PSAP,PTK2B,PTPRE,PTPRF,PUS10,PYGL,RAB26,RAC1,RARA,RARG,RBBP6,RBM39,RBP5,RGS6,RND1,RPA3,RRM1,RTN4IP1,RUVBL1,SART3,SEC23A,SEM1,SEMA4D,SENP2,SERPINA5,SF3B6,SFMBT2,SFN,SH2B2,SH3YL1,SKP1,SLC5A1,SLK,SMAP1,SMG5,SMG7,SMTN,SMURF2,SNX1,SNX22,SPEF1,STARD13,STK16,STK4,SUB1,SULT1A1,SYT13,TAF13,THBS2,TJP1,TLR4,TRIM21,TTR,TXNDC12,UEVLD,USP19,UTRN,VAV3,VEGFB,VPS26A,WEE1,WHRN,WWP1,XIAP,XRCC6,YWHAH,YWHAQ,YY1,ZBTB43,ZCWPW1,ZEB2,ZFP28,ZIC3,ZNF292,ZNF462
118	Mature B,cell neoplasm	Cancer,Hematological Disease,Immunological Disease,Organismal Injury and Abnormalities	1.69E,09	65	ALK,ALOX12,ANXA6,ARHGEF1,ATXN3,B2M,BCR,BTK,CD74,CLK1,CSDE1,CSF2RB,CSTF2,CTCF,DGKA,DPP4,ECI2,EP300,EPHA3,ERBB4,FAS,FCGR2A,FN1,GAD2,GART,GGPS1,GRHPR,GTF2F2,GTF2I,HAT1,HLA,DRB1,HMOX1,HSP90AB1,IFNG,IMPDH1,IQUB,ITGB3,ITPK1,MAGI2,MMP2,NAE1,NCBP1,NDC80,NFATC1,NOTCH1,PC,PGK1,POT1,PRKCB,PTK2B,PTPRE,PTPRF,RAC1,RARA,RRM1,SEM1,SLC5A1,SNX1,TAF13,THBS2,TXNDC12,VAV3,XIAP,XRCC6,ZNF292
119	Anogenital cancer	Cancer,Organismal Injury and Abnormalities	1.74E,09	214	AASDHPPT,ACACB,ACADSB,ACADVL,ACBD7,ACP3,ACY1,ALB,ALK,ANG,ANXA6,AOC3,APEX1,APPL1,AR,ARF6,ARHGAP5,ARHGEF1,ASAP1,ATP7B,B2M,B3GAT2,BCR,BMP1,BTK,BTRC,C1R,C8A,CA8,CAMK4,CANT1,CD1A,CD74,CERT1,CLCN5,CLK1,CSDE1,CSF2RB,CSNK1G1,CSTF2,CTCF,CUL1,CYP17A1,CYP2E1,CYP7A1,DAPK2,DCTD,DHPS,DMC1,DOK2,DPP4,DTYMK,EP300,EPHA3,EPS8L2,ERBB4,ESR1,ETF1,EXOSC9,F2,F3,FABP7,FAP,FAS,FCGR2A,FLNB,FN1,G6PD,GAD2,GALM,GALNT10,GART,GC,GGPS1,GRHPR,GRK2,GRK6,GTF2I,HASPIN,HAT1,HBB,HBEGF,HCK,HDAC7,HDX,HLA,DRB1,HMBOX1,HMGB3,HMGCS1,HMOX1,HNRNPA2B1,HPD,HSD17B4,HSP90AB1,HTR1A,IFNG,IFNGR1,IGF1R,IMPDH1,IQUB,ITGB3,ITPA,ITPKC,ITSN1,KIF11,KMT5A,LRBA,MAGEA4,MAGI2,MAP3K3,MAPK6,MARF1,MARK1,MATK,MDH1,MEMO1,MMP1,MMP2,MSH2,MSN,MUC1,MYBPC1,MYBPC3,MYSM1,NAE1,NBEA,NCBP1,NCOA3,NDC80,NF2,NFATC1,NMRK1,NMT1,NOTCH1,NR1I3,NT5C3A,NUP214,OAT,ODC1,OXSR1,PAFAH1B2,PANK1,PAPSS1,PARN,PARP3,PATJ,PC,PDK3,PDK4,PGC,PGK1,PHF24,POU2F1,PPARG,PPP2R1A,PRKCB,PSAP,PTK2B,PTPRE,PTPRF,PYGL,RAC1,RARA,RARG,RBBP6,RBM39,RGS6,RND1,RRM1,RTN4IP1,RUVBL1,S100A6,SART3,SEMA4D,SENP2,SERPINA5,SF3B6,SFMBT2,SFN,SH3YL1,SKP1,SLC5A1,SLK,SMAP1,SMG5,SMG7,SMTN,SMURF2,SNX1,STK4,SUB1,SULT1A1,SYT13,TAF13,THBS2,TJP1,TLR4,TRIM21,TTR,UEVLD,USP19,UTRN,VAV3,WEE1,WHRN,WWP1,XIAP,YWHAQ,YY1,ZEB2,ZFP28,ZIC3,ZNF292,ZNF462

120	Cell death of breast cancer cell lines	Cell Death and Survival	1.90E,09	27	AR,B2M,BTK,BTRC,CTCF,EP300,ESR1,FAS,FHIT,FN1,HK2,HMOX1,HSP90AB1,IFNG,IGF1R,IQUB,KIF11,MSH2,MUC1,NCOA3,NOTCH1,PA2G4,PDHA1,PPARG,RAC1,SFN,XIAP
121	Cell proliferation of breast cancer cell lines	Cellular Development,Cellular Growth and Proliferation	2.25E,09	34	AR,CHKA,CTCF,DTYMK,EP300,ERBB4,ESR1,F3,FAS,FN1,HAT1,HBEGF,HK2,IFNG,IGF1R,ITGB3,MAPK6,MATK,MEMO1,MTAP,NCOA3,PA2G4,PPARG,PPP2R1A,PRKCB,PTK2B,PTPRE,RAC1,RARA,SFN,TRIM21,UTRN,YWHAQ,YY1
122	Stage IV solid tumor	Cancer,Organismal Injury and Abnormalities	2.30E,09	17	ALB,ALK,AR,BTK,CSF2RB,CYP17A1,ERBB4,ESR1,FCGR2A,GART,HSP90AB1,IGF1R,PARP3,PTK2B,RARA,RARG,RRM1
123	Female genital tract cancer	Cancer,Organismal Injury and Abnormalities,Reproductive System Disease	2.57E,09	168	AASDHPPT,ACACB,ACADSB,ACBD7,ACY1,ALB,ALK,ANG,AOC3,APPL1,AR,ARF6,ARHGEF1,ASAP1,ATP7B,B2M,B3GAT2,BCR,BTK,BTRC,C8A,CA8,CD1A,CD74,CLCN5,CLK1,CSDE1,CSF2RB,CSNK1G1,CSTF2,CTCF,CUL1,CYP7A1,DCTD,DHPS,DMC1,DOK2,DPP4,DTYMK,EP300,EPHA3,ERBB4,ESR1,ETF1,F2,F3,FABP7,FAP,FAS,FCGR2A,FLNB,FN1,G6PD,GAD2,GART,GC,GRHPR,GRK2,GTF2I,HBB,HBEGF,HCK,HDX,HMBOX1,HMGB3,HMOX1,HNRNPA2B1,HPD,HSD17B4,HSP90AB1,HTR1A,IFNGR1,IGF1R,IQUB,ITGB3,ITPA,ITPKC,ITSN1,KIF11,LRBA,MAGEA4,MAGI2,MAP3K3,MAPK6,MARF1,MARK1,MDH1,MEMO1,MMP1,MMP2,MSH2,MSN,MUC1,MYBPC3,MYSM1,NAE1,NBEA,NCBP1,NCOA3,NF2,NFATC1,NMRK1,NMT1,NOTCH1,NR1I3,NT5C3A,NUP214,OAT,ODC1,PANK1,PARN,PARP3,PATJ,PC,PDK3,PDK4,PGC,PGK1,POU2F1,PPARG,PPP2R1A,PRKCB,PTPRE,PTPRF,PYGL,RAC1,RARA,RARG,RBBP6,RBM39,RGS6,RND1,RRM1,RTN4IP1,RUVBL1,SART3,SENP2,SERPINA5,SF3B6,SFMBT2,SH3YL1,SKP1,SLC5A1,SLK,SMG5,SMTN,SMURF2,SNX1,STK4,SUB1,SULT1A1,SYT13,THBS2,TJP1,TLR4,TRIM21,TTR,UEVLD,UTRN,VAV3,WWP1,XIAP,YY1,ZEB2,ZFP28,ZIC3,ZNF292,ZNF462
124	Cell movement	Cellular Movement	2.88E,09	71	ALB,ALOX12,AOC3,APPL1,AR,ARF6,ARHGEF1,ASAP1,BTK,CD74,CERT1,CUL1,DAPK2,DGKA,DOK2,DPP4,ERBB4,ESR1,F2,F3,FABP7,FAP,FAS,FCGR2A,FHIT,FLNB,FN1,GC,GRK6,GTF2I,HBEGF,HMOX1,HNRNPA2B1,IFNG,IGF1R,ITGB3,MAPK6,MATK,MMP1,MMP2,MSN,MUC1,NCOA3,NF2,NFATC1,NOTCH1,PA2G4,PATJ,PPARG,PRKCB,PTK2B,PTPRF,RAC1,RARG,RUVBL1,SEMA4D,SERPINA5,SFN,SLK,STARD13,STK4,THBS2,TJP1,TLR4,VAV3,VEGFB,WARS1,XIAP,YARS1,YY1,ZEB2
125	Transactivation of RNA	Gene Expression	2.89E,09	27	ACACB,APPL1,AR,ARHGEF1,BCR,BTRC,CIAO1,DPP4,EP300,ERBB4,ESR1,GTF2I,IFNG,ITSN1,NCOA3,NFATC1,NOTCH1,NR1I3,POU2F1,PPARG,RAC1,RARA,RARG,SUB1,XIAP,YWHAH,YY1
126	Invasion of tumor cell lines	Cellular Movement	3.13E,09	46	APEX1,AR,ARF6,ASAP1,CD74,CDK5R1,CTCF,DGKA,DPP4,ERBB4,ESR1,F2,FAP,FAS,FHIT,FN1,HBEGF,HMOX1,HNRNPA2B1,HSP90AB1,IFNG,IGF1R,ITGB3,MAPK6,MATK,MMP1,MMP2,MUC1,NCOA3,NOTCH1,PA2G4,PPARG,PTK2B,RAC1,RARG,RUVBL1,S100A6,SFN,SMURF2,TJP1,VEGFB,VPS26A,XIAP,YWHAQ,YY1,ZEB2
127	Refractory tumor	Cancer,Organismal Injury and Abnormalities	3.68E,09	20	ALB,ALK,AR,BCR,BTK,ERBB4,ESR1,GART,GGPS1,HCK,HDAC7,HMOX1,HSP90AB1,IMPDH1,PARP3,PTK2B,RARA,RARG,RRM1,VEGFB
128	Renal cell carcinoma	Cancer,Organismal Injury and Abnormalities,Renal and Urological Disease	3.75E,09	57	ACACB,ALB,AR,ASAP1,ATXN3,B2M,BTRC,CANT1,CSF2RB,CTCF,CYP17A1,ECHS1,EP300,EPHA3,ERBB4,ESR1,FAP,FN1,G6PD,GGPS1,GRHPR,HBB,HLA,DRB1,HSD17B4,HSP90AB1,IGF1R,IMPDH1,ITGB3,KIF11,KLK1,MMP2,MSH2,MUC1,MYBPC1,NCOA3,NF2,NOTCH1,NR1I3,NUP214,PANK1,PDK4,PPARG,PPP2R1A,PTPRE,PYGL,RARA,RBP5,RRM1,SMG7,SMTN,SULT1A1,TJP1,TLR4,ZCWPW1,ZEB2,ZNF292,ZNF462
129	Progressive metastatic solid tumor	Cancer,Organismal Injury and Abnormalities,Tumor Morphology	4.17E,09	14	ALK,AR,CSF2RB,CYP17A1,ERBB4,ESR1,F3,GART,HSP90AB1,IGF1R,PARP3,PTK2B,RRM1,VEGFB
130	Ductal carcinoma	Cancer,Organismal Injury and Abnormalities	4.67E,09	106	ACACB,ACAT2,ACY1,ADH1C,ALB,ALK,APPL1,AR,ATP7B,BPI,BTK,BTRC,CAMK4,CDK5R1,CHKA,CLK1,CRYM,CSDE1,CSNK1G1,CSTF2,CTCF,DAPK2,DCTD,DFFA,DPP4,DSG2,EP300,EPHA3,EPS8L2,ERBB4,ESR1,F2,F3,FAP,FLNB,FN1,GC,GRHPR,GRK2,GRK6,GTF2I,HCK,HDAC7,HLA,DRB1,HMGCS1,HPD,HSP90AB1,IFNGR1,IGF1R,ITPKC,ITSN1,KIF11,KMT5A,LRBA,MAGI2,MAPK6,MARK1,MMP2,MSH2,MSN,MUC1,MYBPC1,MYBPC3,NAE1,NBEA,NCOA3,NDC80,NF2,NFATC1,NOTCH1,NUP214,OXSR1,PA2G4,PARP3,PC,PDHA1,PDK3,PDK4,PGK1,PHF24,POT1,POU2F1,PPP2R1A,PRKCB,PTPRE,PTPRF,RAC1,RARA,RBBP6,RBM39,RRM1,SENP2,SFN,SH2B2,SLK,SMURF2,TJP1,TLR4,TRIM21,UEVLD,UTRN,VAV3,WEE1,ZEB2,ZFP28,ZNF292
131	Renal clear cell adenocarcinoma	Cancer,Organismal Injury and	5.31E,09	48	ACACB,ALB,AR,ASAP1,ATXN3,BTRC,CANT1,CSF2RB,CTCF,CYP17A1,ECHS1,EP300,EPHA3,ERBB4,FAP,FN1,GRHPR,HBB,HLA,DRB1,HSD17B4,IGF1R,IMPDH1,ITGB3,KIF11,KLK1,MMP2,MUC1,MYBPC1,NCOA3,N

		Abnormalities,Renal and Urological Disease			F2,NOTCH1,NR1I3,NUP214,PANK1,PDK4,PPP2R1A,PTPRE,PYGL,RARA,RBP5,SMG7,SMTN,SULT1A1,TJP1,TLR4,ZCWPW1,ZNF292,ZNF462
132	Binding of tumor cell lines	Cell,To,Cell Signaling and Interaction	5.54E,09	27	AR,ARHGEF1,DPP4,DSG2,ESR1,F2,F3,FAS,FCGR2A,FN1,GRK6,HCK,IFNG,IGF1R,ITGB3,MMP2,MUC1,NOTCH1,PPARG,PTK2B,RAC1,SERPINA5,SNX1,TJP1,TLR4,UTRN,ZEB2
133	Invasion of cells	Cellular Movement	6.07E,09	48	APEX1,AR,ARF6,ASAP1,CD74,CDK5R1,CTCF,DGKA,DPP4,ERBB4,ESR1,F2,F3,FAP,FAS,FHIT,FN1,HBEGF,HMOX1,HNRNPA2B1,HSP90AB1,IFNG,IGF1R,ITGB3,MAPK6,MATK,MMP1,MMP2,MUC1,NCOA3,NOTCH1,PA2G4,PPARG,PTK2B,RAC1,RARG,RUVBL1,S100A6,SFN,SMURF2,THBS2,TJP1,VEGFB,VPS26A,XIAP,YWHAQ,YY1,ZEB2
134	Anemia	Cardiovascular Disease,Hematological Disease,Organismal Injury and Abnormalities	6.60E,09	24	ACADVL,ALK,AR,CSF2RB,DPP4,EP300,ESR1,F3,FAS,FCGR2A,G6PD,GRHPR,HBB,HMOX1,IFNG,IMPDH1,ITPA,KLK1,NT5C3A,PTPRE,RARA,RARG,RRM1,TAF13
135	Genitourinary carcinoma	Cancer,Organismal Injury and Abnormalities	7.05E,09	213	AASDHPPT,ACACB,ACADSB,ACADVL,ACBD7,ACY1,ALB,ALK,ANXA6,AOC3,APEX1,APPL1,AR,ARF6,ARHGAP5,ARHGEF1,ASAP1,ATP7B,ATXN3,B2M,B3GAT2,BCR,BMP1,BTK,BTRC,C8A,CA8,CAMK4,CANT1,CD1A,CLCN5,CLK1,CSDE1,CSF2RB,CSNK1G1,CSTF2,CTCF,CUL1,CYP17A1,CYP2E1,CYP7A1,DAPK2,DCTD,DGKA,DHPS,DMC1,DOK2,DPP4,DTYMK,ECHS1,EP300,EPHA3,EPS8L2,ERBB4,ESR1,ETF1,EXOSC9,F2,F3,FABP7,FAP,FAS,FCGR2A,FLNB,FN1,G6PD,GAD2,GALM,GALNT10,GART,GC,GGPS1,GRHPR,GRK2,GRK6,GTF2I,HASPIN,HAT1,HBB,HBEGF,HCK,HDAC7,HDX,HLA,DRB1,HMBOX1,HMGB3,HMGCS1,HNRNPA2B1,HPD,HSD17B4,HSP90AB1,HTR1A,IFNG,IFNGR1,IGF1R,IMPDH1,IQUB,ITGB3,ITPA,ITPKC,ITSN1,KIF11,KLK1,KMT5A,KYNU,LRBA,MAGEA4,MAGI2,MAP3K3,MAPK6,MARF1,MARK1,MATK,MDH1,MEMO1,MMP1,MMP2,MSH2,MSN,MTAP,MUC1,MYBPC1,MYBPC3,MYSM1,NAE1,NBEA,NCBP1,NCOA3,NDC80,NF2,NFATC1,NMRK1,NMT1,NOTCH1,NR1I3,NT5C3A,NUP214,OAT,ODC1,OXSR1,PA2G4,PAFAH1B2,PAH,PANK1,PAPSS1,PARN,PARP3,PATJ,PC,PDK3,PDK4,PGC,PGK1,PHF24,POU2F1,PPARG,PPP2R1A,PRKCB,PSAP,PTK2B,PTPRE,PTPRF,PYGL,RAC1,RARA,RARG,RBBP6,RBM39,RBP5,RGS6,RND1,RRM1,RTN4IP1,RUVBL1,SART3,SENP2,SERPINA5,SF3B6,SFMBT2,SFN,SH3YL1,SKP1,SLC5A1,SLK,SMAP1,SMG5,SMG7,SMTN,SMURF2,STK4,SUB1,SULT1A1,SYT13,THBS2,TJP1,TLR4,TRIM21,UEVLD,USP19,UTRN,VAV3,WEE1,WHRN,WWP1,XIAP,YWHAQ,YY1,ZCWPW1,ZEB2,ZFP28,ZIC3,ZNF292,ZNF462
136	Migration of cells	Cellular Movement	8.58E,09	65	ALB,ALOX12,AOC3,APPL1,AR,ARF6,ARHGEF1,ASAP1,BTK,CD74,CUL1,DAPK2,DOK2,DPP4,ESR1,F2,F3,FABP7,FAP,FAS,FHIT,FLNB,FN1,GRK6,HBEGF,HMOX1,HNRNPA2B1,IFNG,IGF1R,ITGB3,MAPK6,MATK,MMP1,MMP2,MSN,MUC1,NCOA3,NF2,NFATC1,NOTCH1,PA2G4,PATJ,PPARG,PRKCB,PTK2B,PTPRF,RAC1,RARG,RUVBL1,SEMA4D,SERPINA5,SFN,SLK,STARD13,STK4,THBS2,TJP1,TLR4,VAV3,VEGFB,WARS1,XIAP,YARS1,YY1,ZEB2
137	Breast or ovarian carcinoma	Cancer,Organismal Injury and Abnormalities,Reproductive System Disease	9.68E,09	113	ACACB,ALB,ALK,AR,ARF6,ARHGEF1,ASAP1,B2M,BCR,BTK,CA8,CAMK4,CLK1,CSDE1,CSF2RB,CSNK1G1,CSTF2,CTCF,DAPK2,DPP4,EP300,EPHA3,ERBB4,ESR1,EXOSC9,F2,FAP,FLNB,FN1,GALNT10,GART,GGPS1,GRK2,GRK6,HAT1,HBEGF,HCK,HDAC7,HLA,DRB1,HMGCS1,HNRNPA2B1,HSP90AB1,HTR1A,IFNGR1,IGF1R,ITGB3,ITPA,ITPKC,ITSN1,KIF11,KMT5A,MAGEA4,MAGI2,MAP3K3,MAPK6,MARF1,MARK1,MMP2,MSH2,MSN,MUC1,MYBPC3,NBEA,NCOA3,NDC80,NF2,NFATC1,NOTCH1,NR1I3,NT5C3A,ODC1,OXSR1,PAFAH1B2,PARP3,PC,PDK3,PDK4,PGC,PHF24,POU2F1,PPARG,PPP2R1A,PRKCB,PTPRE,PTPRF,PYGL,RAC1,RARA,RBBP6,RBM39,RRM1,RTN4IP1,SENP2,SERPINA5,SFMBT2,SH3YL1,SKP1,SLC5A1,SLK,SMG7,SMTN,SMURF2,SYT13,THBS2,TLR4,USP19,UTRN,VAV3,WEE1,WWP1,YWHAQ,ZEB2,ZNF462
138	Cytolysis	Cell Death and Survival	1.05E,08	17	ALB,B2M,C8A,DPP4,F3,FAS,FCGR2A,G6PD,HBB,HMOX1,IFNG,IMPDH1,ITPA,MSH2,MUC1,NT5C3A,TLR4
139	Refractory malignant tumor	Cancer,Organismal Injury and Abnormalities	1.12E,08	19	ALB,ALK,AR,BCR,BTK,ERBB4,ESR1,GART,HCK,HDAC7,HMOX1,HSP90AB1,IMPDH1,PARP3,PTK2B,RARA,RARG,RRM1,VEGFB
140	Cell death of pancreatic cancer cell lines	Cell Death and Survival	1.17E,08	13	BTRC,FAS,FHIT,FN1,HBEGF,IFNG,IGF1R,NOTCH1,PDK2,RRM1,SFN,VAV3,XIAP
141	Melanoma	Cancer,Organismal Injury and Abnormalities	1.23E,08	222	ACACB,ACADSB,ACSM2A,ADH7,ALB,ALK,ALOX12,AMY1C (includes others),ANG,AOC3,APEX1,APPL1,AR,ARHGAP5,ARHGEF1,ASAP1,ATP7B,ATXN3,B2M,B3GAT2,BCR,BMP1,BPI,BTK,BTRC,C1R,C8A,CA8,CAMK4,CANT1,CD1A,CD74,CHKA,CIAO1,CLCN5,CLK1,CRYM,CSDE1,CSF2RB,CSNK1G2,CSTF2,CTCF,CUL1,CYP17A1,CYP2E1,CYP2R1,CYP7A1,DAPK2,DCTD,DFFA,DGKA,DHPS,D

					MC1,DOK2,DPP4,DSG2,ECI2,EP300,EPHA3,EPS8L2,ERBB4,ESR1,EXOSC9,F2,F3,FABP3,FAP,FAS,FCGR2A,FHIT,FLNB,FN1,GAD2,GALM,GALNT10,GART,GC,GGPS1,GRHPR,GRK2,GTF2I,HADH,HASPIN,HAT1,HBB,HBEGF,HCK,HDAC7,HDX,HK2,HLA,DRB1,HMGCS1,HMOX1,HNRNPA2B1,HPD,HSCB,HSD17B10,HSD17B4,HSP90AB1,HTR1A,IFNG,IFNGR1,IGF1R,IMPA2,IMPDH1,IQUB,ITGB3,ITPA,ITPK1,ITPKC,ITSN1,KLK1,KMT5A,KYNU,LRBA,MAGEA4,MAGI2,MARF1,MARK1,MATK,MDH1,MECR,MEMO1,MMP1,MMP2,MSH2,MSN,MUC1,MYBPC1,MYBPC3,NBEA,NCBP1,NCOA3,NF2,NFATC1,NOTCH1,NR1I3,NSUN5,NUP214,OAT,ODC1,OXSR1,PA2G4,PAFAH1B2,PAH,PANK1,PAPSS1,PARN,PARP3,PATJ,PC,PCYT2,PDHA1,PDK2,PDK3,PDK4,PEX5,PGC,PGK1,PHF24,POT1,POU2F1,PPARG,PPP2R1A,PRKCB,PSAP,PTK2B,PTPRE,PTPRF,PYGL,RAC1,RARA,RARG,RBBP6,RBP5,RGS6,RND1,RPA3,RRM1,RTN4IP1,RUVBL1,SART3,SEC23A,SEM1,SEMA4D,SENP2,SERPINA5,SFMBT2,SH2B2,SH3YL1,SKP1,SLC5A1,SLK,SMAP1,SMG5,SMG7,SMTN,SMURF2,STARD13,STK4,SULT1A1,SYT13,TAF13,THBS2,TIMM9,TJP1,TLR4,TRIM21,USP19,UTRN,VAV3,VEGFB,WHRN,WWP1,XIAP,XRCC6,YY1,ZCWPW1,ZEB2,ZFP28,ZNF292,ZNF462
142	Metastatic adenocarcinoma	Cancer,Organismal Injury and Abnormalities	1.31E,08	16	ALB,ALK,AR,CYP17A1,ERBB4,ESR1,F3,FAS,GART,HSP90AB1,IGF1R,MUC1,NR1I3,PARP3,PTK2B,RRM1
143	Plasma cell dyscrasia	Cancer,Hematological Disease,Immunological Disease,Organismal Injury and Abnormalities	1.36E,08	35	ANXA6,B2M,BCR,BTK,CLK1,CSF2RB,ECI2,EP300,FAS,FN1,GART,GGPS1,GRHPR,GTF2I,HAT1,HLA,DRB1,HMOX1,HSP90AB1,IFNG,IMPDH1,ITGB3,ITPK1,MMP2,NAE1,NDC80,NFATC1,PRKCB,PTK2B,PTPRE,PTPRF,RAC1,RARA,RRM1,SNX1,XRCC6
144	Lung tumor	Cancer,Organismal Injury and Abnormalities,Respiratory Disease	1.50E,08	135	ACAT2,ADH1C,ADH7,ALB,ALK,ALOX12,ANXA6,APEX1,AR,ARHGAP5,ARHGEF1,B2M,B3GAT2,BMP1,BTK,BTRC,C8A,CA8,CAMK4,CD1A,CERT1,CLCN5,CLK1,CSF2RB,CSNK1G2,CTCF,CUL1,CYP17A1,CYP2E1,DGKA,DOK2,DPP4,DSG2,EP300,EPHA3,ERBB4,ESR1,F2,F3,FAP,FAS,FHIT,FN1,GAD2,GALNT10,GART,GGPS1,GRHPR,GRK6,GTF2I,HASPIN,HCK,HDX,HK2,HMGB3,HMOX1,HNRNPA2B1,HPD,HSD17B4,HSP90AB1,HTR1A,IFNG,IFNGR1,IGF1R,IQUB,ITPKC,KIF11,KYNU,LRBA,MAGEA4,MAGI2,MAPK6,MATK,MDH1,MMP1,MMP2,MSH2,MSN,MTAP,MUC1,MYBPC1,NBEA,NCOA3,NDC80,NF2,NMT1,NOTCH1,NT5C3A,OAT,ODC1,OXSR1,PAH,PANK1,PARP3,PATJ,PDHA1,PDK4,PEX5,PGC,PGK1,PIP4K2C,POT1,PPARG,PPP2R1A,PRKCB,PTK2B,PTPRE,PTPRF,PYGL,RAC1,RARA,RARG,RBBP6,RGS6,RRM1,SEMA4D,SENP2,SERPINA5,SFN,SKP1,SLK,SMG7,SMURF2,STK4,SULT1A1,THBS2,TJP1,TLR4,TTR,TXNDC12,UTRN,VAV3,WWP1,ZCWPW1,ZNF292
145	Chronic inflammatory disorder	Inflammatory Disease	1.54E,08	52	ALB,ALK,ALOX12,ANG,ASAP1,B2M,BTK,CD1A,CD74,CSF2RB,CYP2E1,DPP4,EP300,ERI1,ESR1,F2,F3,FAS,FCGR2A,FN1,G6PD,GC,GGPS1,GRHPR,HBEGF,HCK,HDAC7,HLA,DRB1,HMOX1,IDE,IFNG,IFNGR1,IGF1R,IMPDH1,ITGB3,ITPA,MMP1,MMP2,MTAP,MUC1,PGK1,PPARG,PTK2B,PTPRE,RGS6,RRM1,SF3B6,SMG7,TAF13,TJP1,TLR4,XIAP
146	Adenocarcinoma	Cancer,Organismal Injury and Abnormalities	1.85E,08	258	AASDHPPT,ACACB,ACADSB,ACADVL,ACAT2,ACBD7,ACSM2A,ACVR2B,ACY1,ADH1C,ADH7,ALB,ALK,ALOX12,ANXA6,AOC3,APEX1,APPL1,AR,ARF6,ARHGAP5,ARHGEF1,ASAP1,ATP7B,ATXN3,B2M,B3GAT2,BCR,BMP1,BPI,BTK,BTRC,C1R,C8A,CA8,CAMK4,CANT1,CD1A,CD74,CDK5R1,CERT1,CHKA,CLCN5,CLK1,CRYM,CSDE1,CSF2RB,CSNK1G1,CSNK1G2,CSTF2,CTCF,CUL1,CYP17A1,CYP2E1,CYP7A1,DAPK2,DCTD,DCXR,DFFA,DGKA,DHPS,DMC1,DOK2,DPP4,DSG2,DTYMK,ECHS1,EP300,EPHA3,EPS8L2,ERBB4,ESR1,ETF1,EXOSC9,F2,F3,FAP,FAS,FCGR2A,FHIT,FLNB,FN1,G6PD,GAD2,GALM,GALNT10,GART,GC,GGPS1,GRHPR,GRK2,GRK6,GTF2I,HADH,HASPIN,HBB,HBEGF,HCK,HDAC7,HDX,HIBCH,HK2,HLA,DRB1,HMBOX1,HMGB3,HMGCS1,HMOX1,HNRNPA2B1,HPD,HSCB,HSD17B10,HSD17B4,HSP90AB1,HTR1A,IDE,IFNG,IFNGR1,IGF1R,IMPA2,IMPDH1,IQUB,ITGB3,ITPA,ITPK1,ITPKC,ITSN1,KIF11,KLK1,KMT5A,KYNU,LRBA,MAGEA4,MAGI2,MAP3K3,MAPK6,MARF1,MARK1,MATK,MDH1,MECR,MEMO1,MMP1,MMP2,MSH2,MSN,MTAP,MUC1,MYBPC1,MYBPC3,MYSM1,NAE1,NBEA,NCBP1,NCOA3,NDC80,NF2,NFATC1,NMRK1,NMT1,NOTCH1,NR1I3,NSUN5,NT5C3A,NUP214,OAT,ODC1,OXSR1,PA2G4,PAFAH1B2,PAH,PANK1,PAPSS1,PARN,PARP3,PATJ,PC,PCYT2,PDHA1,PDK2,PDK3,PDK4,PGC,PGK1,PHF24,PIP4K2C,POT1,POU2F1,PPARG,PPP2R1A,PRKCB,PTK2B,PTPRE,PTPRF,PUS10,PYGL,RAB26,RAC1,RARA,RARG,RBBP6,RBM39,RBP5,RGS6,RND1,RRM1,RTN4IP1,RUVBL1,SART3,SEC23A,SEMA4D,SENP2,SERPINA5,SF3B6,SFMBT2,SFN,SH2B2,SH3YL1,SKP1,SLC5A1,SLK,SMAP1,SMG5,SMG7,SMTN,SMURF2,SNX1,SPEF1,STARD13,STK16,STK4,SUB1,SULT1A1,SYT13

					,THBS2,TJP1,TLR4,TRIM21,TTR,UEVLD,USP19,UTRN,VAV3,VEGFB,VPS26A,WEE1,WHRN,WWP1,XIAP,XRCC6,YWHAH,YY1,ZBTB43,ZCWPW1,ZEB2,ZFP28,ZIC3,ZNF292,ZNF462
147	Lung cancer	Cancer,Organismal Injury and Abnormalities,Respiratory Disease	2.01E,08	134	ACAT2,ADH1C,ADH7,ALB,ALK,ALOX12,ANXA6,APEX1,AR,ARHGAP5,ARHGEF1,B2M,B3GAT2,BMP1,BTK,BTRC,C8A,CA8,CAMK4,CD1A,CERT1,CLCN5,CLK1,CSF2RB,CSNK1G2,CTCF,CUL1,CYP17A1,CYP2E1,DGKA,DOK2,DPP4,DSG2,EP300,EPHA3,ERBB4,ESR1,F2,F3,FAP,FAS,FHIT,FN1,GAD2,GALNT10,GART,GGPS1,GRHPR,GRK6,GTF2I,HASPIN,HCK,HDX,HK2,HMGB3,HNRNPA2B1,HPD,HSD17B4,HSP90AB1,HTR1A,IFNG,IFNGR1,IGF1R,IQUB,ITPKC,KIF11,KYNU,LRBA,MAGEA4,MAGI2,MAPK6,MATK,MDH1,MMP1,MMP2,MSH2,MSN,MTAP,MUC1,MYBPC1,NBEA,NCOA3,NDC80,NF2,NMT1,NOTCH1,NT5C3A,OAT,ODC1,OXSR1,PAH,PANK1,PARP3,PATJ,PDHA1,PDK4,PEX5,PGC,PGK1,PIP4K2C,POT1,PPARG,PPP2R1A,PRKCB,PTK2B,PTPRE,PTPRF,PYGL,RAC1,RARA,RARG,RBBP6,RGS6,RRM1,SEMA4D,SENP2,SERPINA5,SFN,SKP1,SLK,SMG7,SMURF2,STK4,SULT1A1,THBS2,TJP1,TLR4,TTR,TXNDC12,UTRN,VAV3,WWP1,ZCWPW1,ZNF292
148	Recurrent carcinoma	Cancer,Organismal Injury and Abnormalities	2.16E,08	14	ALB,ALK,AR,CYP17A1,ERBB4,ESR1,F3,GART,HSP90AB1,IGF1R,IMPDH1,PARP3,PTK2B,RRM1
149	Apoptosis of breast cancer cell lines	Cell Death and Survival	2.34E,08	23	AR,B2M,BTK,BTRC,CTCF,EP300,ESR1,FAS,FHIT,FN1,HK2,HMOX1,IFNG,IGF1R,KIF11,MUC1,NCOA3,PA2G4,PDHA1,PPARG,RAC1,SFN,XIAP
150	Acne vulgaris	Dermatological Diseases and Conditions,Inflammatory Disease,Organismal Injury and Abnormalities	2.36E,08	7	AR,DHPS,ESR1,G6PD,ODC1,RARA,RARG
151	Apoptosis of cancer cells	Cancer,Cell Death and Survival,Organismal Injury and Abnormalities,Tumor Morphology	2.77E,08	14	ALB,AR,B2M,BTK,FAS,FHIT,HMOX1,IFNG,IGF1R,MUC1,NCOA3,RAC1,RARA,XIAP
152	Interaction of DNA	Cell Cycle	2.96E,08	24	ALB,APEX1,AR,BTK,CYP2E1,EP300,ESR1,F2,F3,FN1,HCK,IFNG,MAP3K3,MUC1,NOTCH1,NR1I3,PARP3,POU2F1,PPARG,RAC1,RARA,STK4,TLR4,XRCC6
153	Lung carcinoma	Cancer,Organismal Injury and Abnormalities,Respiratory Disease	3.13E,08	131	ACAT2,ADH1C,ADH7,ALB,ALK,ALOX12,ANXA6,APEX1,AR,ARHGAP5,ARHGEF1,B2M,B3GAT2,BMP1,BTK,BTRC,C8A,CA8,CAMK4,CD1A,CERT1,CLCN5,CLK1,CSF2RB,CSNK1G2,CTCF,CUL1,CYP17A1,CYP2E1,DGKA,DOK2,DPP4,DSG2,EP300,EPHA3,ERBB4,ESR1,F2,F3,FAP,FAS,FHIT,FN1,GAD2,GALNT10,GART,GGPS1,GRHPR,GRK6,GTF2I,HASPIN,HCK,HDX,HK2,HMGB3,HNRNPA2B1,HPD,HSD17B4,HSP90AB1,HTR1A,IFNG,IFNGR1,IGF1R,IQUB,ITPKC,KIF11,KYNU,LRBA,MAGEA4,MAGI2,MAPK6,MATK,MDH1,MMP1,MMP2,MSH2,MSN,MTAP,MUC1,MYBPC1,NBEA,NCOA3,NDC80,NMT1,NOTCH1,NT5C3A,OAT,ODC1,OXSR1,PAH,PANK1,PARP3,PATJ,PDHA1,PDK4,PEX5,PGC,PGK1,PIP4K2C,POT1,PPARG,PPP2R1A,PRKCB,PTK2B,PTPRE,PTPRF,PYGL,RAC1,RARA,RARG,RBBP6,RGS6,RRM1,SEMA4D,SENP2,SERPINA5,SFN,SLK,SMG7,SMURF2,STK4,SULT1A1,THBS2,TJP1,TLR4,TTR,TXNDC12,VAV3,WWP1,ZCWPW1,ZNF292
154	Binding of lipid	Lipid Metabolism,Small Molecule Biochemistry	3.24E,08	9	BPI,ESR1,F2,FAS,HSP90AB1,NF2,PSAP,RARA,TLR4
155	Extrapancreatic malignant tumor	Cancer,Organismal Injury and Abnormalities	3.33E,08	277	AASDHPPT,ACACB,ACADSB,ACADVL,ACAT2,ACBD7,ACP3,ACSM2A,ACVR2B,ACY1,ADH1C,ADH7,ALB,ALK,ALOX12,AMY1C (includes others),ANG,ANXA6,AOC3,APEX1,APPL1,AR,ARF6,ARHGAP5,ARHGEF1,ASAP1,ATP7B,ATXN3,B2M,B3GAT2,BCR,BMP1,BPI,BTK,BTRC,C1R,C8A,CA8,CAMK4,CANT1,CD1A,CD74,CDK5R1,CERT1,CHKA,CIAO1,CLCN5,CLK1,CRYM,CSDE1,CSF2RB,CSNK1G1,CSNK1G2,CSTF2,CTCF,CUL1,CYP17A1,CYP2E1,CYP2R1,CYP7A1,DAPK2,DCTD,DCXR,DFFA,DGKA,DHPS,DMC1,DOK2,DPP4,DSG2,DTYMK,ECHS1,ECI2,EP300,EPHA3,EPS8L2,ERBB4,ESR1,ETF1,EXOSC9,F2,F3,FABP7,FAP,FAS,FCGR2A,FHIT,FLNB,FN1,G6PD,GAD2,GALM,GALNT10,GART,GC,GGPS1,GRHPR,GRK2,GRK6,GTF2F2,GTF2I,HADH,HASPIN,HAT1,HBB,HBEGF,HCK,HDAC7,HDX,HIBCH,HK2,HLA,DRB1,HMBOX1,HMGB3,HMGCS1,HMOX1,HNRNPA2B1,HPD,HSCB,HSD17B10,HSD17B4,HSP90AB1,HTR1A,IDE,IFNG,IFNGR1,IGF1R,IMPA2,IMPDH1,IQUB,ITGB3,ITPA,ITPK1,ITPKC,ITSN1,KIF11,KLK1,KMT5A,KYNU,LRBA,MAGEA4,MAGI2,MAP3K3,MAPK6,MARF1,MARK1,MATK,MDH1,ME

					CR,MEMO1,MMP1,MMP2,MSH2,MSN,MTAP,MUC1,MYBPC1,MYBPC3,MYSM1,NAE1,NBEA,NCBP1,NCOA3,NDC80,NF2,NFATC1,NME3,NMRK1,NMT1,NOTCH1,NR1I3,NSUN5,NT5C3A,NUP214,OAT,ODC1,OXSR1,PA2G4,PAFAH1B2,PAH,PANK1,PAPSS1,PARN,PARP3,PATJ,PC,PCYT2,PDHA1,PDK2,PDK3,PDK4,PEX5,PGC,PGK1,PHF24,PIP4K2C,POT1,POU2F1,PPARG,PPP2R1A,PRKCB,PSAP,PTK2B,PTPRE,PTPRF,PUS10,PYGL,RAB26,RAC1,RARA,RARG,RBBP6,RBM39,RBP5,RGS6,RND1,RRM1,RTN4IP1,RUVBL1,S100A6,SART3,SEC23A,SEM1,SEMA4D,SENP2,SERPINA5,SF3B6,SFMBT2,SFN,SH2B2,SH3YL1,SKP1,SLC5A1,SLK,SMAP1,SMG5,SMG7,SMTN,SMURF2,SNX1,SPEF1,STARD13,STK16,STK4,SUB1,SULT1A1,SYT13,TAF13,THBS2,TIMM9,TJP1,TLR4,TNFAIP8L2,TRIM21,TTR,TXNDC12,UEVLD,USP19,UTRN,VAV3,VEGFB,VPS26A,WEE1,WHRN,WWP1,XIAP,XRCC6,YWHAH,YWHAQ,YY1,ZBTB43,ZCWPW1,ZEB2,ZFP28,ZIC3,ZNF292,ZNF462
156	Apoptosis of prostate cancer cells	Cancer,Cell Death and Survival,Organismal Injury and Abnormalities,Tumor Morphology	3.42E,08	4	AR,NCOA3,RARA,XIAP
157	Glucose metabolism disorder	Metabolic Disease,Organismal Injury and Abnormalities	3.43E,08	44	ACAT2,ALB,APPL1,AR,B2M,BPI,CYP2E1,CYP2R1,DPP4,ECHS1,ESR1,F2,F3,FAS,FCGR2A,FN1,GC,GGPS1,HADH,HBB,HK2,HMBOX1,HTR1A,IDE,IFNG,IGF1R,IMPDH1,ITGB3,KIF11,MAGI2,NOTCH1,PDK4,PPARG,PRKCB,RAC1,SLC5A1,SMURF2,SUB1,TAF13,THBS2,TLR4,UTRN,VEGFB,ZEB2
158	T,cell malignant neoplasm	Cancer,Hematological Disease,Immunological Disease,Organismal Injury and Abnormalities	3.43E,08	38	ALK,B2M,C1R,CD1A,CLK1,CTCF,DPP4,ECHS1,EP300,EPHA3,ERBB4,FAS,FHIT,FN1,GTF2I,HCK,HDAC7,HNRNPA2B1,HSP90AB1,IMPDH1,MARK1,MMP1,MMP2,MUC1,NFATC1,NOTCH1,NT5C3A,ODC1,POT1,POU2F1,PRKCB,PTPRE,RARA,RARG,RRM1,TAF13,TJP1,TLR4
159	Advanced uterine cancer	Cancer,Organismal Injury and Abnormalities,Reproductive System Disease	3.77E,08	10	ALK,AOC3,AR,ESR1,F3,GAD2,GART,HSP90AB1,PARP3,RRM1
160	Cell movement of tumor cell lines	Cellular Movement	4.08E,08	51	ALOX12,APPL1,AR,ARF6,ARHGEF1,CERT1,DPP4,ESR1,F2,F3,FABP7,FHIT,FLNB,FN1,GC,GRK6,HBEGF,HMOX1,HNRNPA2B1,IFNG,IGF1R,ITGB3,MAPK6,MATK,MMP1,MMP2,MSN,MUC1,NCOA3,NF2,NOTCH1,PA2G4,PPARG,PRKCB,PTK2B,RAC1,RARG,RUVBL1,SEMA4D,SERPINA5,SFN,STARD13,THBS2,TLR4,VAV3,VEGFB,WARS1,XIAP,YARS1,YY1,ZEB2
161	Stage I,II cancer	Cancer,Organismal Injury and Abnormalities	4.56E,08	17	ALB,ALK,B2M,BTK,CSF2RB,ERBB4,ESR1,FN1,GART,GGPS1,HSP90AB1,MUC1,PGC,RARA,RARG,RRM1,ZEB2
162	Metabolism of nucleic acid component or derivative	Nucleic Acid Metabolism	4.80E,08	23	ACACB,ACP3,CYP2E1,DCTD,F2,FHIT,G6PD,GRK2,HMOX1,HTR1A,IFNG,IMPDH1,ITPA,KYNU,MTAP,NMRK1,NT5C3A,PAPSS1,PDHA1,PGK1,PRKCB,SNX1,SULT1A1
163	Respiratory system tumor	Cancer,Organismal Injury and Abnormalities,Respiratory Disease	4.95E,08	141	ACAT2,ADH1C,ADH7,ALB,ALK,ALOX12,ANXA6,APEX1,AR,ARHGAP5,ARHGEF1,B2M,B3GAT2,BMP1,BTK,BTRC,C8A,CA8,CAMK4,CD1A,CERT1,CLCN5,CLK1,CSF2RB,CSNK1G2,CTCF,CUL1,CYP17A1,CYP2E1,CYP2R1,DGKA,DOK2,DPP4,DSG2,EP300,EPHA3,ERBB4,ESR1,F2,F3,FAP,FAS,FHIT,FN1,GAD2,GALNT10,GART,GGPS1,GRHPR,GRK6,GTF2I,HASPIN,HCK,HDX,HK2,HMGB3,HMOX1,HNRNPA2B1,HPD,HSD17B4,HSP90AB1,HTR1A,IDE,IFNG,IFNGR1,IGF1R,IQUB,ITPKC,KIF11,KMT5A,KYNU,LRBA,MAGEA4,MAGI2,MAPK6,MATK,MDH1,MMP1,MMP2,MSH2,MSN,MTAP,MUC1,MYBPC1,NBEA,NCOA3,NDC80,NF2,NMT1,NOTCH1,NT5C3A,OAT,ODC1,OXSR1,PAH,PANK1,PARP3,PATJ,PDHA1,PDK2,PDK4,PEX5,PGC,PGK1,PIP4K2C,POT1,PPARG,PPP2R1A,PRKCB,PSAP,PTK2B,PTPRE,PTPRF,PYGL,RAC1,RARA,RARG,RBBP6,RGS6,RRM1,SEMA4D,SENP2,SERPINA5,SFN,SKP1,SLK,SMG7,SMURF2,STK4,SULT1A1,THBS2,TJP1,TLR4,TTR,TXNDC12,UTRN,VAV3,WWP1,ZCWPW1,ZNF292,ZNF462
164	Binding of mononuclear leukocytes	Cell,To,Cell Signaling and Interaction,Hematological System Development and Function	5.26E,08	13	AOC3,ARHGEF1,BPI,F2,FAS,FN1,IFNG,ITGB3,MSN,PPARG,PTK2B,RAC1,TLR4

165	Cellular homeostasis	Cellular Function and Maintenance	5.65E,08	45	ARF6,ATP7B,ATXN3,B2M,BTK,CLCN5,CUL1,CYP2E1,DAPK2,EP300,F2,F3,FAS,FN1,GC,GRK2,HK2,HLA,DRB1,HMOX1,HSD17B10,IFNG,ITPK1,ITSN1,MMP1,MSH2,NDUFAB1,NOTCH1,PAFAH1B2,PAPSS1,PDK2,PDK4,PEX5,PIP4K2C,PPARG,PSAP,PTK2B,PYGL,RAC1,SKP1,THBS2,TRIM21,USP19,VPS26A,XIAP,YWHAQ
166	Pelvic adenocarcinoma	Cancer,Organismal Injury and Abnormalities	5.87E,08	178	AASDHPPT,ACACB,ACADSB,ACADVL,ACBD7,ACY1,ALB,ALK,ANXA6,AOC3,APEX1,APPL1,AR,ARF6,ARHGAP5,ARHGEF1,ASAP1,ATP7B,B2M,B3GAT2,BCR,BTK,C8A,CA8,CANT1,CD1A,CLCN5,CLK1,CSDE1,CSF2RB,CSNK1G1,CSTF2,CTCF,CUL1,CYP17A1,CYP2E1,CYP7A1,DCTD,DMC1,DOK2,DPP4,DTYMK,EP300,EPHA3,EPS8L2,ERBB4,ESR1,ETF1,F2,F3,FAP,FCGR2A,FLNB,FN1,G6PD,GAD2,GALM,GALNT10,GART,GC,GRK2,GTF2I,HASPIN,HBB,HBEGF,HCK,HDAC7,HDX,HLA,DRB1,HMBOX1,HMGB3,HNRNPA2B1,HPD,HSD17B4,HSP90AB1,HTR1A,IFNG,IFNGR1,IGF1R,IQUB,ITGB3,ITPA,ITPKC,ITSN1,KIF11,KMT5A,LRBA,MAGEA4,MAGI2,MAP3K3,MAPK6,MARF1,MARK1,MATK,MDH1,MEMO1,MMP1,MMP2,MSN,MUC1,MYBPC1,MYBPC3,MYSM1,NBEA,NCBP1,NCOA3,NF2,NFATC1,NMRK1,NMT1,NOTCH1,NR1I3,NT5C3A,NUP214,OAT,ODC1,OXSR1,PANK1,PAPSS1,PARN,PARP3,PATJ,PC,PDK3,PDK4,PGC,PGK1,PHF24,POU2F1,PPARG,PPP2R1A,PRKCB,PTK2B,PTPRE,PTPRF,PYGL,RARA,RARG,RBBP6,RBM39,RGS6,RND1,RRM1,RTN4IP1,RUVBL1,SART3,SENP2,SERPINA5,SF3B6,SFMBT2,SFN,SH3YL1,SKP1,SLC5A1,SLK,SMAP1,SMG5,SMTN,SMURF2,STK4,SUB1,SYT13,THBS2,TLR4,TRIM21,UEVLD,USP19,UTRN,VAV3,WHRN,WWP1,XIAP,YY1,ZEB2,ZFP28,ZIC3,ZNF292,ZNF462
167	Extraadrenal retroperitoneal tumor	Cancer,Organismal Injury and Abnormalities	5.96E,08	116	ACACB,ACAT2,ALB,ALK,ALOX12,APPL1,AR,ARHGAP5,ASAP1,ATP7B,ATXN3,B2M,BPI,BTRC,CANT1,CD74,CDK5R1,CHKA,CLK1,CRYM,CSDE1,CSF2RB,CSTF2,CTCF,CYP17A1,DCTD,DFFA,DOK2,DPP4,ECHS1,EP300,EPHA3,ERBB4,ESR1,F2,F3,FAP,FLNB,FN1,G6PD,GART,GC,GGPS1,GRHPR,GTF2I,HBB,HBEGF,HCK,HIBCH,HLA,DRB1,HMGCS1,HMOX1,HPD,HSD17B4,HSP90AB1,IGF1R,IMPDH1,ITGB3,ITSN1,KIF11,KLK1,LRBA,MAGI2,MARK1,MMP2,MSH2,MUC1,MYBPC1,MYBPC3,NBEA,NCOA3,NF2,NFATC1,NOTCH1,NR1I3,NUP214,PA2G4,PANK1,PARP3,PC,PDHA1,PDK4,PGC,PGK1,POT1,POU2F1,PPARG,PPP2R1A,PRKCB,PTPRE,PYGL,RARA,RARG,RBBP6,RBM39,RBP5,RRM1,S100A6,SEC23A,SERPINA5,SFN,SMG7,SMTN,SULT1A1,TJP1,TLR4,UTRN,VAV3,VEGFB,WEE1,YY1,ZCWPW1,ZEB2,ZFP28,ZNF292,ZNF462
168	Synthesis of lipid	Lipid Metabolism,Small Molecule Biochemistry	6.00E,08	32	ACACB,ACAT2,ALB,ALOX12,ANG,CD74,CERT1,CHKA,CSNK1G2,CYP17A1,CYP2E1,CYP2R1,CYP7A1,DGKA,DPP4,ECHS1,F2,FAS,FCGR2A,FN1,G6PD,GGPS1,HMGCS1,HSD17B10,HSD17B4,IFNG,NCOA3,PCYT2,PDK4,PIP4K2C,PPARG,RAC1
169	Stage III cancer	Cancer,Organismal Injury and Abnormalities	6.13E,08	17	ALB,ALK,BTK,CSF2RB,ERBB4,ESR1,FN1,GART,GGPS1,HMOX1,HSP90AB1,IGF1R,MUC1,PARP3,RARA,RARG,RRM1
170	Malignant soft tissue neoplasm	Cancer,Organismal Injury and Abnormalities	6.14E,08	211	ACACB,ACADSB,ACSM2A,ADH7,ALB,ALK,ALOX12,AMY1C (includes others),ANG,AOC3,APEX1,APPL1,AR,ARHGAP5,ARHGEF1,ASAP1,ATP7B,ATXN3,B2M,BCR,BMP1,BPI,BTK,BTRC,C1R,C8A,CA8,CAMK4,CANT1,CD1A,CD74,CHKA,CLCN5,CRYM,CSDE1,CSF2RB,CSNK1G2,CSTF2,CTCF,CYP17A1,CYP2E1,CYP7A1,DAPK2,DCTD,DFFA,DGKA,DHPS,DMC1,DOK2,DPP4,DSG2,ECI2,EP300,EPHA3,EPS8L2,ERBB4,ESR1,F2,F3,FABP3,FABP7,FAP,FAS,FCGR2A,FHIT,FLNB,FN1,GAD2,GALM,GALNT10,GART,GC,GGPS1,GRHPR,GRK2,GTF2I,HASPIN,HAT1,HBB,HBEGF,HCK,HDAC7,HDX,HK2,HLA,DRB1,HMGCS1,HNRNPA2B1,HPD,HSCB,HSD17B10,HSD17B4,HSP90AB1,HTR1A,IFNG,IFNGR1,IGF1R,IMPDH1,IQUB,ITGB3,ITPK1,ITPKC,ITSN1,KMT5A,KYNU,LRBA,MAGEA4,MAGI2,MAPK6,MARF1,MARK1,MATK,MDH1,MECR,MEMO1,MMP1,MMP2,MSH2,MSN,MUC1,MYBPC1,MYBPC3,NAE1,NBEA,NCBP1,NCOA3,NF2,NFATC1,NOTCH1,NR1I3,NSUN5,NUP214,OAT,ODC1,OXSR1,PA2G4,PAFAH1B2,PAH,PANK1,PAPSS1,PARN,PARP3,PATJ,PC,PCYT2,PDK2,PDK3,PDK4,PEX5,PGC,PGK1,PHF24,POT1,POU2F1,PPARG,PPP2R1A,PRKCB,PSAP,PTK2B,PTPRE,PTPRF,PYGL,RAC1,RARA,RARG,RBBP6,RBP5,RGS6,RND1,RPA3,RRM1,RTN4IP1,RUVBL1,SART3,SEC23A,SEM1,SEMA4D,SENP2,SERPINA5,SFMBT2,SH2B2,SH3YL1,SKP1,SLC5A1,SLK,SMAP1,SMG5,SMG7,SMTN,SMURF2,STARD13,STK4,SULT1A1,SYT13,THBS2,TIMM9,TJP1,TLR4,TRIM21,USP19,UTRN,VAV3,WHRN,WWP1,XIAP,XRCC6,ZCWPW1,ZEB2,ZFP28,ZIC3,ZNF292,ZNF462
171	Malignant neoplasm of respiratory system	Cancer,Organismal Injury and Abnormalities,Respiratory Disease	6.23E,08	140	ACAT2,ADH1C,ADH7,ALB,ALK,ALOX12,ANXA6,APEX1,AR,ARHGAP5,ARHGEF1,B2M,B3GAT2,BMP1,BTK,BTRC,C8A,CA8,CAMK4,CD1A,CERT1,CLCN5,CLK1,CSF2RB,CSNK1G2,CTCF,CUL1,CYP17A1,CYP2E1,CYP2R1,DGKA,DOK2,DPP4,DSG2,EP300,EPHA3,ERBB4,ESR1,F2,F3,FAP,FAS,FHIT,FN1,GAD2,GALNT10,GART,GGPS1,GRHPR,GRK6,GTF2I,HASPIN,HCK,HDX,HK2,HMGB3,HNRNPA2B1,HPD,HSD17B4,HSP90AB1,HTR1A,IDE,IFNG,IFNGR1,IGF1R,IQUB,ITPKC,KIF11,KMT5A,KYNU,LRBA,MAGEA4,MAGI2,MAPK6,MATK,

					MDH1,MMP1,MMP2,MSH2,MSN,MTAP,MUC1,MYBPC1,NBEA,NCOA3,NDC80,NF2,NMT1,NOTCH1,NT5C3A,OAT,ODC1,OXSR1,PAH,PANK1,PARP3,PATJ,PDHA1,PDK2,PDK4,PEX5,PGC,PGK1,PIP4K2C,POT1,PPARG,PPP2R1A,PRKCB,PSAP,PTK2B,PTPRE,PTPRF,PYGL,RAC1,RARA,RARG,RBBP6,RGS6,RRM1,SEMA4D,SENP2,SERPINA5,SFN,SKP1,SLK,SMG7,SMURF2,STK4,SULT1A1,THBS2,TJP1,TLR4,TTR,TXNDC12,UTRN,VAV3,WWP1,ZCWPW1,ZNF292,ZNF462
172	Primary immunodeficiency disorder	Immunological Disease	6.30E,08	15	ARHGEF1,BTK,CA8,CD74,FAS,FCGR2A,G6PD,GRHPR,IFNGR1,LRBA,MSN,PPARG,TAF13,TLR4,XIAP
173	Serous ovarian adenocarcinoma	Cancer,Endocrine System Disorders,Organismal Injury and Abnormalities,Reproductive System Disease	6.37E,08	19	ALK,AR,B2M,BCR,CTCF,DPP4,EP300,EPHA3,HSP90AB1,MUC1,NOTCH1,PARP3,PDK4,PPP2R1A,RRM1,SKP1,THBS2,TLR4,VAV3
174	Breast carcinoma	Cancer,Organismal Injury and Abnormalities,Reproductive System Disease	6.47E,08	83	ACACB,ALK,AR,ARHGEF1,BCR,BTK,CAMK4,CLK1,CSDE1,CSNK1G1,CTCF,DAPK2,DPP4,EP300,EPHA3,ERBB4,ESR1,EXOSC9,FAP,FLNB,FN1,GALNT10,GGPS1,GRK2,GRK6,HAT1,HDAC7,HLA,DRB1,HMGCS1,HSP90AB1,IGF1R,ITGB3,ITPKC,KIF11,KMT5A,MAGEA4,MAPK6,MARF1,MARK1,MMP2,MSH2,MSN,MUC1,MYBPC3,NBEA,NCOA3,NDC80,NF2,NFATC1,NOTCH1,OXSR1,PAFAH1B2,PARP3,PC,PDK3,PDK4,PHF24,POU2F1,PPARG,PPP2R1A,PRKCB,PTPRE,PTPRF,RAC1,RARA,RBBP6,RBM39,RRM1,SENP2,SERPINA5,SLC5A1,SLK,SMG7,SMTN,SMURF2,SYT13,TLR4,USP19,VAV3,WEE1,YWHAQ,ZEB2,ZNF462
175	Abdominal adenocarcinoma	Cancer,Organismal Injury and Abnormalities	6.59E,08	253	AASDHPPT,ACACB,ACADSB,ACADVL,ACAT2,ACBD7,ACSM2A,ACVR2B,ACY1,ADH1C,ADH7,ALB,ALK,ALOX12,ANXA6,AOC3,APEX1,APPL1,AR,ARF6,ARHGAP5,ARHGEF1,ASAP1,ATP7B,ATXN3,B2M,B3GAT2,BCR,BMP1,BPI,BTK,BTRC,C1R,C8A,CA8,CAMK4,CANT1,CD1A,CD74,CDK5R1,CHKA,CLCN5,CLK1,CRYM,CSDE1,CSF2RB,CSNK1G1,CSNK1G2,CSTF2,CTCF,CUL1,CYP17A1,CYP2E1,CYP7A1,DAPK2,DCTD,DCXR,DFFA,DGKA,DHPS,DMC1,DOK2,DPP4,DSG2,DTYMK,ECHS1,EP300,EPHA3,EPS8L2,ERBB4,ESR1,ETF1,EXOSC9,F2,F3,FAP,FAS,FCGR2A,FHIT,FLNB,FN1,G6PD,GAD2,GALM,GALNT10,GART,GC,GGPS1,GRHPR,GRK2,GRK6,GTF2I,HADH,HASPIN,HBB,HBEGF,HCK,HDAC7,HDX,HIBCH,HK2,HLA,DRB1,HMBOX1,HMGB3,HMGCS1,HMOX1,HNRNPA2B1,HPD,HSCB,HSD17B10,HSD17B4,HSP90AB1,HTR1A,IDE,IFNG,IFNGR1,IGF1R,IMPA2,IMPDH1,IQUB,ITGB3,ITPA,ITPK1,ITPKC,ITSN1,KIF11,KLK1,KMT5A,KYNU,LRBA,MAGEA4,MAGI2,MAP3K3,MAPK6,MARF1,MARK1,MATK,MDH1,MECR,MEMO1,MMP1,MMP2,MSH2,MSN,MTAP,MUC1,MYBPC1,MYBPC3,MYSM1,NAE1,NBEA,NCBP1,NCOA3,NF2,NFATC1,NMRK1,NMT1,NOTCH1,NR1I3,NSUN5,NT5C3A,NUP214,OAT,ODC1,OXSR1,PA2G4,PAFAH1B2,PANK1,PAPSS1,PARN,PARP3,PATJ,PC,PCYT2,PDHA1,PDK2,PDK3,PDK4,PGC,PGK1,PHF24,POT1,POU2F1,PPARG,PPP2R1A,PRKCB,PTK2B,PTPRE,PTPRF,PUS10,PYGL,RAB26,RAC1,RARA,RARG,RBBP6,RBM39,RBP5,RGS6,RND1,RRM1,RTN4IP1,RUVBL1,SART3,SEC23A,SEMA4D,SENP2,SERPINA5,SF3B6,SFMBT2,SFN,SH2B2,SH3YL1,SKP1,SLC5A1,SLK,SMAP1,SMG5,SMG7,SMTN,SMURF2,SNX1,SPEF1,STARD13,STK16,STK4,SUB1,SULT1A1,SYT13,THBS2,TJP1,TLR4,TRIM21,UEVLD,USP19,UTRN,VAV3,VEGFB,VPS26A,WEE1,WHRN,WWP1,XIAP,XRCC6,YWHAH,YY1,ZBTB43,ZCWPW1,ZEB2,ZFP28,ZIC3,ZNF292,ZNF462
176	Diabetes mellitus	Endocrine System Disorders,Gastrointestinal Disease,Metabolic Disease,Organismal Injury and Abnormalities	6.98E,08	41	ALB,APPL1,AR,B2M,BPI,CYP2E1,CYP2R1,DPP4,ECHS1,ESR1,F3,FAS,FCGR2A,FN1,GC,GGPS1,HADH,HBB,HK2,HMBOX1,HTR1A,IDE,IFNG,IGF1R,IMPDH1,ITGB3,KIF11,MAGI2,NOTCH1,PDK4,PPARG,PRKCB,RAC1,SLC5A1,SMURF2,TAF13,THBS2,TLR4,UTRN,VEGFB,ZEB2
177	Rheumatic Disease	Connective Tissue Disorders,Inflammatory Disease,Organismal Injury and Abnormalities,Skeletal and Muscular Disorders	7.23E,08	54	ALB,ALOX12,ANG,AR,ASAP1,B2M,BPI,C1R,CD1A,CD74,CDK5R1,CSF2RB,CYP2E1,DPP4,ESR1,FAS,FCGR2A,FN1,GGPS1,HBB,HCK,HDAC7,HLA,DRB1,HMOX1,IDE,IFNG,IGF1R,IMPDH1,ITPA,MAP3K3,MMP1,MMP2,MTAP,NF2,NFATC1,PGK1,PPARG,PRKCB,PTK2B,PTPRE,RAC1,RGS6,RRM1,S100A6,SF3B6,SMG7,TAF13,TIMM9,TJP1,TLR4,TRIM21,WARS1,XIAP,XRCC6

178	Bladder cancer	Cancer,Organismal Injury and Abnormalities,Renal and Urological Disease	7.39E,08	59	ACACB,ALB,ALK,AOC3,AR,ARHGAP5,BCR,BMP1,BTK,CSF2RB,CSTF2,CTCF,CUL1,DGKA,DHPS,DOK2,EP300,EPHA3,EPS8L2,ERBB4,FAP,FN1,GART,GRHPR,HNRNPA2B1,HSP90AB1,KMT5A,KYNU,MAGEA4,MAGI2,MARF1,MMP2,MTAP,NCOA3,NF2,NOTCH1,NT5C3A,ODC1,PA2G4,PAH,PDK4,PPARG,PPP2R1A,PRKCB,PSAP,PTK2B,PTPRF,RARA,RARG,RBBP6,RBM39,RRM1,SLC5A1,THBS2,TLR4,UTRN,XIAP,ZCWPW1,ZEB2
179	Metastatic non,small cell lung carcinoma	Cancer,Organismal Injury and Abnormalities,Respiratory Disease	7.73E,08	14	ALB,ALK,B2M,ERBB4,FAS,FN1,GART,GGPS1,HSP90AB1,IGF1R,PARP3,PGC,PTK2B,RRM1
180	Connective or soft tissue tumor	Cancer,Organismal Injury and Abnormalities	7.94E,08	214	ACACB,ACADSB,ACSM2A,ADH7,ALB,ALK,ALOX12,AMY1C (includes others),ANG,AOC3,APEX1,APPL1,AR,ARHGAP5,ARHGEF1,ASAP1,ATP7B,ATXN3,B2M,BCR,BMP1,BPI,BTK,BTRC,C1R,C8A,CA8,CAMK4,CANT1,CD1A,CD74,CHKA,CLCN5,CRYM,CSDE1,CSF2RB,CSNK1G2,CSTF2,CTCF,CYP17A1,CYP2E1,CYP7A1,DAPK2,DCTD,DFFA,DGKA,DHPS,DMC1,DOK2,DPP4,DSG2,ECI2,EP300,EPHA3,EPS8L2,ERBB4,ESR1,F2,F3,FABP3,FABP7,FAP,FAS,FCGR2A,FHIT,FLNB,FN1,GAD2,GALM,GALNT10,GART,GC,GGPS1,GRHPR,GRK2,GTF2I,HASPIN,HAT1,HBB,HBEGF,HCK,HDAC7,HDX,HK2,HLA,DRB1,HMGCS1,HNRNPA2B1,HPD,HSCB,HSD17B10,HSD17B4,HSP90AB1,HTR1A,IFNG,IFNGR1,IGF1R,IMPDH1,IQUB,ITGB3,ITPK1,ITPKC,ITSN1,KMT5A,KYNU,LRBA,MAGEA4,MAGI2,MAPK6,MARF1,MARK1,MATK,MDH1,MECR,MEMO1,MMP1,MMP2,MSH2,MSN,MUC1,MYBPC1,MYBPC3,NAE1,NBEA,NCBP1,NCOA3,NF2,NFATC1,NOTCH1,NR1I3,NSUN5,NUP214,OAT,ODC1,OXSR1,PA2G4,PAFAH1B2,PAH,PANK1,PAPSS1,PARN,PARP3,PATJ,PC,PCYT2,PDK2,PDK3,PDK4,PEX5,PGC,PGK1,PHF24,POT1,POU2F1,PPARG,PPP2R1A,PRKCB,PSAP,PTK2B,PTPRE,PTPRF,PYGL,RAC1,RARA,RARG,RBBP6,RBP5,RGS6,RND1,RPA3,RRM1,RTN4IP1,RUVBL1,S100A6,SART3,SEC23A,SEM1,SEMA4D,SENP2,SERPINA5,SFMBT2,SH2B2,SH3YL1,SKP1,SLC5A1,SLK,SMAP1,SMG5,SMG7,SMTN,SMURF2,STARD13,STK4,SULT1A1,SYT13,TAF13,THBS2,TIMM9,TJP1,TLR4,TRIM21,USP19,UTRN,VAV3,WEE1,WHRN,WWP1,XIAP,XRCC6,ZCWPW1,ZEB2,ZFP28,ZIC3,ZNF292,ZNF462
181	Neuroblastoma	Cancer,Neurological Disease,Organismal Injury and Abnormalities	8.33E,08	14	ALK,AR,CSF2RB,CTCF,EP300,FAP,FCGR2A,HSP90AB1,IDE,IGF1R,PGK1,RARA,RARG,UTRN
182	Advanced adenocarcinoma	Cancer,Organismal Injury and Abnormalities	8.61E,08	19	ALB,ALK,AR,BCR,DPP4,ERBB4,ESR1,F3,FAS,GART,HSP90AB1,IGF1R,MUC1,NR1I3,PARP3,PTK2B,RRM1,SKP1,VAV3
183	Pelvic carcinoma	Cancer,Organismal Injury and Abnormalities	8.65E,08	195	AASDHPPT,ACACB,ACADSB,ACADVL,ACBD7,ACY1,ADH7,ALB,ALK,ANXA6,AOC3,APEX1,APPL1,AR,ARF6,ARHGAP5,ARHGEF1,ASAP1,ATP7B,B2M,B3GAT2,BCR,BMP1,BTK,BTRC,C8A,CA8,CANT1,CD1A,CLCN5,CLK1,CSDE1,CSF2RB,CSNK1G1,CSTF2,CTCF,CUL1,CYP17A1,CYP2E1,CYP7A1,DCTD,DGKA,DHPS,DMC1,DOK2,DPP4,DTYMK,EP300,EPHA3,EPS8L2,ERBB4,ESR1,ETF1,F2,F3,FABP7,FAP,FAS,FCGR2A,FLNB,FN1,G6PD,GAD2,GALM,GALNT10,GART,GC,GRHPR,GRK2,GTF2I,HASPIN,HBB,HBEGF,HCK,HDAC7,HDX,HLA,DRB1,HMBOX1,HMGB3,HNRNPA2B1,HPD,HSD17B4,HSP90AB1,HTR1A,IFNG,IFNGR1,IGF1R,IQUB,ITGB3,ITPA,ITPKC,ITSN1,KIF11,KMT5A,KYNU,LRBA,MAGEA4,MAGI2,MAP3K3,MAPK6,MARF1,MARK1,MATK,MDH1,MEMO1,MMP1,MMP2,MSH2,MSN,MTAP,MUC1,MYBPC1,MYBPC3,MYSM1,NAE1,NBEA,NCBP1,NCOA3,NF2,NFATC1,NMRK1,NMT1,NOTCH1,NR1I3,NT5C3A,NUP214,OAT,ODC1,OXSR1,PA2G4,PAH,PANK1,PAPSS1,PARN,PARP3,PATJ,PC,PDK3,PDK4,PGC,PGK1,PHF24,POU2F1,PPARG,PPP2R1A,PRKCB,PSAP,PTK2B,PTPRE,PTPRF,PYGL,RARA,RARG,RBBP6,RBM39,RGS6,RND1,RRM1,RTN4IP1,RUVBL1,SART3,SENP2,SERPINA5,SF3B6,SFMBT2,SFN,SH3YL1,SKP1,SLC5A1,SLK,SMAP1,SMG5,SMTN,SMURF2,STK4,SUB1,SYT13,THBS2,TJP1,TLR4,TRIM21,UEVLD,USP19,UTRN,VAV3,WHRN,WWP1,XIAP,YY1,ZCWPW1,ZEB2,ZFP28,ZIC3,ZNF292,ZNF462
184	Atopic dermatitis	Dermatological Diseases and Conditions,Immunological Disease,Inflammatory Disease,Inflammatory Response,Organismal Injury and Abnormalities	9.04E,08	19	ACADVL,CD1A,CD74,CSF2RB,GRK2,HBB,HCK,HLA,DRB1,HTR1A,IDE,KLK1,MSN,PDK4,PPARG,PRKCB,PSAP,RUVBL1,TLR4,XRCC6

185	Binding of leukocytes	Cell,To,Cell Signaling and Interaction,Hematological System Development and Function	9.17E,08	17	AOC3,ARHGEF1,BPI,F2,F3,FAS,FCGR2A,FN1,IFNG,ITGB3,MSN,PPARG,PTK2B,RAC1,SEMA4D,TLR4,UTRN
186	Cell death of kidney cell lines	Cell Death and Survival,Organismal Injury and Abnormalities,Renal and Urological Disease	9.36E,08	20	AR,ATXN3,F2,F3,FAS,GRK2,HK2,IFNG,IGF1R,NDUFAB1,NF2,PEX5,PPARG,PRKCB,RAC1,STK4,TNFAIP8L2,XIAP,YWHAQ,YY1
187	Refractory solid tumor	Cancer,Organismal Injury and Abnormalities	9.48E,08	15	ALB,ALK,AR,BTK,ERBB4,GART,GGPS1,HDAC7,HMOX1,HSP90AB1,IMPDH1,PARP3,PTK2B,RRM1,VEGFB
188	Primary neoplasm	Cancer,Organismal Injury and Abnormalities	9.54E,08	29	ALB,ALK,B2M,BTK,CDK5R1,CSF2RB,CSNK1G2,CTCF,DOK2,EP300,ERBB4,ESR1,FN1,GART,HDAC7,HSP90AB1,IGF1R,MUC1,NF2,ODC1,PARP3,PPARG,PPP2R1A,RAC1,RARA,RARG,RRM1,SEMA4D,VEGFB
189	Expression of RNA	Gene Expression	9.55E,08	63	ACVR2B,ANG,APEX1,AR,ATXN3,BTK,BTRC,CAMK4,CD74,CDK5R1,CIAO1,CRYM,CTCF,DHPS,EP300,ERBB4,ESR1,ETF1,F2,F3,FAS,FN1,GTF2F2,GTF2I,HBB,HDAC7,HLA,DRB1,HMBOX1,HNRNPA2B1,IFNG,KMT5A,MAP3K3,MSH2,MSN,MYSM1,NCBP1,NCOA3,NFATC1,NOTCH1,NR1I3,PA2G4,PC,POU2F1,PPARG,PRKCB,RAC1,RARA,RARG,RBBP6,SART3,SEMA4D,SFMBT2,SMURF2,SUB1,TAF13,TLR4,WARS1,XRCC6,YWHAH,YWHAQ,YY1,ZEB2,ZIC3
190	Skin lesion	Dermatological Diseases and Conditions,Organismal Injury and Abnormalities	9.87E,08	209	ACACB,ACADSB,ACSM2A,ADH7,ALB,ALK,ALOX12,AMY1C (includes others),ANG,AOC3,APEX1,APPL1,AR,ARHGAP5,ARHGEF1,ASAP1,ATP7B,ATXN3,B2M,BCR,BMP1,BPI,BTK,BTRC,C1R,C8A,CA8,CAMK4,CANT1,CD1A,CD74,CHKA,CLCN5,CRYM,CSDE1,CSF2RB,CSNK1G2,CSTF2,CTCF,CYP17A1,CYP2E1,CYP7A1,DAPK2,DCTD,DFFA,DGKA,DHPS,DMC1,DOK2,DPP4,DSG2,ECI2,EP300,EPHA3,EPS8L2,ERBB4,ESR1,F2,F3,FABP3,FAP,FAS,FCGR2A,FHIT,FLNB,FN1,G6PD,GAD2,GALM,GALNT10,GART,GC,GGPS1,GRHPR,GRK2,GRK6,GTF2I,HASPIN,HAT1,HBB,HBEGF,HCK,HDAC7,HDX,HK2,HLA,DRB1,HMGCS1,HNRNPA2B1,HPD,HSCB,HSD17B10,HSP90AB1,HTR1A,IFNG,IFNGR1,IGF1R,IMPDH1,IQUB,ITGB3,ITPK1,ITPKC,ITSN1,KYNU,LRBA,MAGEA4,MAGI2,MARF1,MARK1,MATK,MDH1,MECR,MEMO1,MMP1,MMP2,MSH2,MSN,MUC1,MYBPC1,MYBPC3,NBEA,NCBP1,NCOA3,NF2,NFATC1,NOTCH1,NR1I3,NSUN5,NUP214,OAT,ODC1,OXSR1,PA2G4,PAFAH1B2,PAH,PANK1,PAPSS1,PARN,PARP3,PATJ,PC,PCYT2,PDK2,PDK3,PDK4,PEX5,PGC,PGK1,PHF24,POT1,POU2F1,PPARG,PPP2R1A,PRKCB,PSAP,PTK2B,PTPRE,PTPRF,PYGL,RAC1,RARA,RARG,RBBP6,RBP5,RGS6,RND1,RPA3,RRM1,RTN4IP1,RUVBL1,SART3,SEC23A,SEM1,SEMA4D,SENP2,SERPINA5,SFMBT2,SH2B2,SH3YL1,SKP1,SLC5A1,SLK,SMAP1,SMG5,SMG7,SMTN,SMURF2,STARD13,STK4,SULT1A1,SYT13,THBS2,TIMM9,TJP1,TLR4,TRIM21,UEVLD,USP19,UTRN,VAV3,WHRN,WWP1,XIAP,XRCC6,ZBTB43,ZCWPW1,ZEB2,ZFP28,ZNF292,ZNF462
191	Abdominal neoplasm	Cancer,Organismal Injury and Abnormalities	1.00E,07	269	AASDHPPT,ACACB,ACADSB,ACADVL,ACAT2,ACBD7,ACP3,ACSM2A,ACVR2B,ACY1,ADH1C,ADH7,ALB,ALK,ALOX12,ANG,ANXA6,AOC3,APEX1,APPL1,AR,ARF6,ARHGAP5,ARHGEF1,ASAP1,ATP7B,ATXN3,B2M,B3GAT2,BCR,BMP1,BPI,BTK,BTRC,C1R,C8A,CA8,CAMK4,CANT1,CD1A,CD74,CDK5R1,CHKA,CLCN5,CLK1,CRYM,CSDE1,CSF2RB,CSNK1G1,CSNK1G2,CSTF2,CTCF,CUL1,CYP17A1,CYP2E1,CYP7A1,DAPK2,DCTD,DCXR,DFFA,DGKA,DHPS,DMC1,DOK2,DPP4,DSG2,DTYMK,ECHS1,ECI2,EP300,EPHA3,EPS8L2,ERBB4,ERI1,ESR1,ETF1,EXOSC9,F2,F3,FABP3,FABP7,FAP,FAS,FCGR2A,FHIT,FLNB,FN1,G6PD,GAD2,GALM,GALNT10,GART,GC,GGPS1,GRHPR,GRK2,GRK6,GTF2F2,GTF2I,HADH,HASPIN,HBB,HBEGF,HCK,HDAC7,HDX,HIBCH,HK2,HLA,DRB1,HMBOX1,HMGB3,HMGCS1,HMOX1,HNRNPA2B1,HPD,HSCB,HSD17B10,HSD17B4,HSP90AB1,HTR1A,IDE,IFNG,IFNGR1,IGF1R,IMPA2,IMPDH1,IQUB,ITGB3,ITPA,ITPK1,ITPKC,ITSN1,KIF11,KLK1,KMT5A,KYNU,LRBA,MAGEA4,MAGI2,MAP3K3,MAPK6,MARF1,MARK1,MATK,MDH1,MECR,MEMO1,MMP1,MMP2,MSH2,MSN,MTAP,MUC1,MYBPC1,MYBPC3,MYSM1,NAE1,NBEA,NCBP1,NCOA3,NF2,NFATC1,NMRK1,NMT1,NOTCH1,NR1I3,NSUN5,NT5C3A,NUP214,OAT,ODC1,OXSR1,PA2G4,PAFAH1B2,PAH,PANK1,PAPSS1,PARN,PARP3,PATJ,PC,PCYT2,PDHA1,PDK2,PDK3,PDK4,PEX5,PGC,PGK1,PHF24,POT1,POU2F1,PPARG,PPP2R1A,PRKCB,PSAP,PTK2B,PTPRE,PTPRF,PUS10,PYGL,RAB26,RAC1,RARA,RARG,RBBP6,RBM39,RBP5,RGS6,RND1,RRM1,RTN4IP1,RUVBL1,S100A6,SART3,SEC23A,SEMA4D,SENP2,SERPINA5,SF

					3B6,SFMBT2,SFN,SH2B2,SH3YL1,SKP1,SLC5A1,SLK,SMAP1,SMG5,SMG7,SMTN,SMURF2,SNX1,SNX22,SPEF1,STARD13,STK16,STK4,SUB1,SULT1A1,SYT13,TAF13,THBS2,TJP1,TLR4,TNFAIP8L2,TRIM21,TTR,UEVLD,USP19,UTRN,VAV3,VEGFB,VPS26A,WEE1,WHRN,WWP1,XIAP,XRCC6,YWHAH,YWHAQ,YY1,ZBTB43,ZCWPW1,ZEB2,ZFP28,ZIC3,ZNF292,ZNF462
192	Hypersensitive reaction	Immunological Disease	1.01E,07	21	ACADVL,CD1A,CD74,CSF2RB,FCGR2A,GRK2,HBB,HCK,HLA,DRB1,HTR1A,IDE,IFNG,KLK1,MSN,PDK4,PPARG,PRKCB,PSAP,RUVBL1,TLR4,XRCC6
193	Uterine tumor	Cancer,Organismal Injury and Abnormalities,Reproductive System Disease	1.01E,07	148	AASDHPPT,ACACB,ACADSB,ACBD7,ACY1,ALB,ALK,AOC3,APPL1,AR,ARHGAP5,ATP7B,B2M,B3GAT2,BCR,BTK,BTRC,C8A,CA8,CD1A,CLCN5,CSDE1,CSF2RB,CSTF2,CTCF,CUL1,CYP7A1,DCTD,DHPS,DMC1,DOK2,DPP4,DTYMK,EP300,EPHA3,ERBB4,ESR1,ETF1,F3,FABP7,FAP,FAS,FCGR2A,FLNB,FN1,G6PD,GAD2,GART,GRHPR,GRK2,GTF2I,HBB,HDAC7,HDX,HMBOX1,HMGB3,HMOX1,HPD,HSD17B10,HSD17B4,HSP90AB1,HTR1A,IFNG,IGF1R,IQUB,ITGB3,ITPKC,KIF11,LRBA,MAGEA4,MAGI2,MAP3K3,MAPK6,MARF1,MARK1,MDH1,MEMO1,MMP1,MMP2,MSH2,MSN,MUC1,MYBPC3,MYSM1,NAE1,NBEA,NCBP1,NF2,NFATC1,NMRK1,NMT1,NOTCH1,NT5C3A,NUP214,OAT,PANK1,PARN,PARP3,PATJ,PDK3,PDK4,PGK1,PPARG,PPP2R1A,PTPRE,PTPRF,RAC1,RARA,RARG,RBBP6,RBM39,RGS6,RND1,RRM1,RUVBL1,S100A6,SART3,SENP2,SERPINA5,SF3B6,SH3YL1,SLC5A1,SLK,SMG5,SMTN,SMURF2,SNX1,STK4,SUB1,SULT1A1,SYT13,TAF13,THBS2,TJP1,TLR4,TRIM21,UEVLD,UTRN,VAV3,WEE1,WWP1,XIAP,YY1,ZEB2,ZFP28,ZIC3,ZNF292,ZNF462
194	Waldenstr 枚 m macroglobulinemia	Cancer,Hematological Disease,Immunological Disease,Organismal Injury and Abnormalities	1.03E,07	20	ANXA6,BTK,CLK1,CSF2RB,ECI2,EP300,FN1,GTF2I,HAT1,HMOX1,IMPDH1,ITGB3,MMP2,PRKCB,PTK2B,PTPRF,RAC1,RRM1,SNX1,XRCC6
195	Stage I cancer	Cancer,Organismal Injury and Abnormalities	1.12E,07	14	ALK,B2M,CSF2RB,ERBB4,ESR1,FN1,GART,GGPS1,HSP90AB1,MUC1,PGC,RARA,RARG,RRM1
196	Nervous system neoplasm	Cancer,Neurological Disease,Organismal Injury and Abnormalities	1.24E,07	190	ACACB,ACADSB,ACADVL,ACVR2B,ACY1,ADH7,ALB,ALK,ALOX12,AMY1C (includes others),AOC3,APEX1,APPL1,AR,ARHGAP5,ARHGEF1,ASAP1,ATP7B,ATXN3,B3GAT2,BCR,BMP1,BPI,BTK,C1R,C8A,CLCN5,CRYM,CSDE1,CSF2RB,CSNK1G1,CSNK1G2,CSTF2,CTCF,CUL1,CYP2E1,DAPK2,DCXR,DGKA,DOK2,DPP4,DSG2,EP300,EPHA3,EPS8L2,ERBB4,ESR1,F2,FABP7,FAP,FAS,FCGR2A,FLNB,FN1,G6PD,GAD2,GALNT10,GART,GRK2,GRK6,GTF2I,HADH,HASPIN,HAT1,HBEGF,HDX,HIBCH,HK2,HLA,DRB1,HMBOX1,HMGB3,HMGCS1,HMOX1,HNRNPA2B1,HSD17B10,HSD17B4,HSP90AB1,IDE,IFNG,IFNGR1,IGF1R,IMPDH1,ITGB3,ITPK1,ITSN1,KLK1,KMT5A,KYNU,LRBA,MAGEA4,MAGI2,MAP3K3,MARF1,MATK,MMP2,MSH2,MSN,MTAP,MUC1,MYBPC1,MYBPC3,NAE1,NBEA,NCBP1,NCOA3,NF2,NFATC1,NME3,NMT1,NOTCH1,NR1I3,NSUN5,NUP214,ODC1,OXSR1,PA2G4,PAFAH1B2,PAH,PANK1,PAPSS1,PARN,PATJ,PC,PCYT2,PDHA1,PDK4,PGC,PGK1,PHF24,PIP4K2C,POT1,POU2F1,PPARG,PPP2R1A,PRKCB,PSAP,PTK2B,PTPRE,PTPRF,PUS10,PYGL,RAC1,RARA,RARG,RBBP6,RBM39,RBP5,RGS6,RPA3,RRM1,RTN4IP1,RUVBL1,SART3,SEMA4D,SENP2,SERPINA5,SFMBT2,SH2B2,SH3YL1,SLC5A1,SLK,SMG5,SMURF2,SNX1,STARD13,SUB1,TAF13,THBS2,TIMM9,TJP1,TLR4,TNFAIP8L2,USP19,UTRN,VAV3,VEGFB,VPS26A,WEE1,WHRN,XIAP,XRCC6,YWHAH,YWHAQ,ZBTB43,ZCWPW1,ZEB2,ZFP28,ZIC3,ZNF292,ZNF462
197	Cutaneous melanoma	Cancer,Dermatological Diseases and Conditions,Organismal Injury and Abnormalities	1.27E,07	204	ACACB,ACADSB,ACSM2A,ADH7,ALB,ALK,ALOX12,AMY1C (includes others),ANG,AOC3,APEX1,APPL1,AR,ARHGAP5,ARHGEF1,ASAP1,ATP7B,ATXN3,B2M,BCR,BMP1,BPI,BTK,BTRC,C1R,C8A,CA8,CAMK4,CANT1,CD1A,CD74,CHKA,CLCN5,CRYM,CSDE1,CSF2RB,CSNK1G2,CSTF2,CTCF,CYP17A1,CYP2E1,CYP7A1,DAPK2,DCTD,DFFA,DGKA,DHPS,DMC1,DOK2,DPP4,DSG2,ECI2,EP300,EPHA3,EPS8L2,ERBB4,ESR1,F2,F3,FABP3,FAP,FAS,FCGR2A,FHIT,FLNB,FN1,GAD2,GALM,GALNT10,GART,GC,GGPS1,GRHPR,GRK2,GTF2I,HASPIN,HAT1,HBB,HBEGF,HCK,HDAC7,HDX,HK2,HLA,DRB1,HMGCS1,HNRNPA2B1,HPD,HSCB,HSD17B10,HSP90AB1,HTR1A,IFNG,IFNGR1,IGF1R,IMPDH1,IQUB,ITGB3,ITPK1,ITPKC,ITSN1,KYNU,LRBA,MAGEA4,MAGI2,MARF1,MARK1,MATK,MDH1,MECR,MEMO1,MMP1,MMP2,MSH2,MSN,MUC1,MYBPC1,MYBPC3,NBEA,NCBP1,NCOA3,NF2,NFATC1,NOTCH1,NR1I3,NSUN5,NUP214,OAT,ODC1,OXSR1,PA2G4,PAFAH1B2,PAH,PANK1,PAPSS1,PARN,PARP3,PATJ,PC,PCYT2,PDK2,PDK3,PDK4,PEX5,PGC,PGK1,PHF24,POT1,POU2F1,PPARG,PPP2R1A,PRKCB,PSAP,PTK2B,PTPRE,PTPRF,PYGL,RAC1,RARA,RARG,RBBP6,RBP5,RGS6,RND1,RPA3,RRM1,RTN4IP1,RUVBL1,SART3,SEC23A,SEM1,SEMA4D,SENP2,SERPINA5,SFMBT2,SH2B2,SH3YL1,SKP1,SLC5A1,SLK,SMAP1,SMG5,SMG7,SMTN,SMURF2,STARD13,STK4,S

					ULT1A1,SYT13,THBS2,TIMM9,TJP1,TLR4,TRIM21,USP19,UTRN,VAV3,WHRN,WWP1,XIAP,XRCC6,ZCWPW1,ZEB2,ZNF292,ZNF462
198	Production of hydrogen peroxide	Free Radical Scavenging,Small Molecule Biochemistry	1.28E,07	8	AOC3,F2,FAS,FCGR2A,HBB,IFNG,RAC1,TLR4
199	Liver cancer	Cancer,Gastrointestinal Disease,Hepatic System Disease,Organismal Injury and Abnormalities	1.29E,07	137	ACACB,ACADSB,ACADVL,ACY1,ALB,ALK,ANG,APEX1,AR,ARHGAP5,ASAP1,ATXN3,B2M,B3GAT2,BCR,BTK,BTRC,C1R,C8A,CANT1,CD1A,CDK5R1,CLCN5,CLK1,CSDE1,CSF2RB,CSNK1G1,CTCF,CYP7A1,DGKA,ECHS1,ECI2,EP300,EPHA3,EPS8L2,ERBB4,ESR1,EXOSC9,F2,FABP7,FAP,FLNB,FN1,GC,GGPS1,GRHPR,GTF2F2,HBB,HCK,HDAC7,HDX,HIBCH,HK2,HMGB3,HMGCS1,HMOX1,HNRNPA2B1,HPD,HSP90AB1,HTR1A,IDE,IFNGR1,IGF1R,IMPDH1,IQUB,KMT5A,KYNU,MAGI2,MAPK6,MARF1,MDH1,MECR,MMP2,MSH2,MUC1,MYBPC1,MYBPC3,NAE1,NBEA,NCBP1,NCOA3,NF2,NFATC1,NMT1,NOTCH1,NUP214,PAH,PATJ,PC,PCYT2,PDHA1,PDK3,PDK4,PEX5,PGK1,PHF24,POT1,POU2F1,PPARG,PPP2R1A,PRKCB,PSAP,PTPRE,PTPRF,PYGL,RAC1,RARA,RBBP6,RRM1,SART3,SEC23A,SFMBT2,SLK,SMG7,SMURF2,STARD13,SULT1A1,THBS2,TJP1,TNFAIP8L2,TRIM21,UEVLD,USP19,UTRN,VAV3,VEGFB,WEE1,WWP1,XRCC6,YWHAH,YY1,ZBTB43,ZCWPW1,ZEB2,ZIC3,ZNF292,ZNF462
200	Adult solid tumor	Cancer,Organismal Injury and Abnormalities	1.40E,07	15	AR,B2M,CSF2RB,DPP4,EP300,FAS,FHIT,GGPS1,HNRNPA2B1,HTR1A,IMPDH1,NOTCH1,POT1,PRKCB,RRM1
201	Low,grade lymphoma	Cancer,Hematological Disease,Immunological Disease,Organismal Injury and Abnormalities	1.50E,07	35	ANXA6,ATXN3,BTK,CLK1,CSDE1,CSF2RB,CSTF2,DGKA,ECI2,EP300,ERBB4,FN1,GTF2I,HAT1,HMOX1,HSP90AB1,IFNG,IMPDH1,ITGB3,MMP2,NCBP1,NFATC1,NOTCH1,POT1,PRKCB,PTK2B,PTPRF,RAC1,RRM1,SEM1,SNX1,THBS2,VAV3,XRCC6,ZNF292
202	Hepatobiliary system cancer	Cancer,Gastrointestinal Disease,Hepatic System Disease,Organismal Injury and Abnormalities	1.54E,07	143	ACACB,ACADSB,ACADVL,ACY1,ADH1C,ALB,ALK,ANG,APEX1,AR,ARHGAP5,ASAP1,ATXN3,B2M,B3GAT2,BCR,BTK,BTRC,C1R,C8A,CANT1,CD1A,CDK5R1,CLCN5,CLK1,CSDE1,CSF2RB,CSNK1G1,CTCF,CYP7A1,DGKA,DSG2,ECHS1,ECI2,EP300,EPHA3,EPS8L2,ERBB4,ESR1,EXOSC9,F2,FABP7,FAP,FLNB,FN1,GC,GGPS1,GRHPR,GTF2F2,HBB,HCK,HDAC7,HDX,HIBCH,HK2,HMGB3,HMGCS1,HMOX1,HNRNPA2B1,HPD,HSP90AB1,HTR1A,IDE,IFNGR1,IGF1R,IMPDH1,IQUB,KIF11,KMT5A,KYNU,MAGI2,MAPK6,MARF1,MDH1,MECR,MMP2,MSH2,MUC1,MYBPC1,MYBPC3,NAE1,NBEA,NCBP1,NCOA3,NF2,NFATC1,NMT1,NOTCH1,NUP214,PA2G4,PAH,PATJ,PC,PCYT2,PDHA1,PDK3,PDK4,PEX5,PGK1,PHF24,POT1,POU2F1,PPARG,PPP2R1A,PRKCB,PSAP,PTPRE,PTPRF,PYGL,RAC1,RARA,RBBP6,RRM1,SART3,SEC23A,SFMBT2,SH2B2,SLK,SMG7,SMURF2,STARD13,SULT1A1,THBS2,TJP1,TLR4,TNFAIP8L2,TRIM21,UEVLD,USP19,UTRN,VAV3,VEGFB,WEE1,WWP1,XRCC6,YWHAH,YY1,ZBTB43,ZCWPW1,ZEB2,ZIC3,ZNF292,ZNF462
203	Skin cancer	Cancer,Dermatological Diseases and Conditions,Organismal Injury and Abnormalities	1.61E,07	208	ACACB,ACADSB,ACSM2A,ADH7,ALB,ALK,ALOX12,AMY1C (includes others),ANG,AOC3,APEX1,APPL1,AR,ARHGAP5,ARHGEF1,ASAP1,ATP7B,ATXN3,B2M,BCR,BMP1,BPI,BTK,BTRC,C1R,C8A,CA8,CAMK4,CANT1,CD1A,CD74,CHKA,CLCN5,CRYM,CSDE1,CSF2RB,CSNK1G2,CSTF2,CTCF,CYP17A1,CYP2E1,CYP7A1,DAPK2,DCTD,DFFA,DGKA,DHPS,DMC1,DOK2,DPP4,DSG2,ECI2,EP300,EPHA3,EPS8L2,ERBB4,ESR1,F2,F3,FABP3,FAP,FAS,FCGR2A,FHIT,FLNB,FN1,GAD2,GALM,GALNT10,GART,GC,GGPS1,GRHPR,GRK2,GRK6,GTF2I,HASPIN,HAT1,HBB,HBEGF,HCK,HDAC7,HDX,HK2,HLA,DRB1,HMGCS1,HNRNPA2B1,HPD,HSCB,HSD17B10,HSP90AB1,HTR1A,IFNG,IFNGR1,IGF1R,IMPDH1,IQUB,ITGB3,ITPK1,ITPKC,ITSN1,KYNU,LRBA,MAGEA4,MAGI2,MARF1,MARK1,MATK,MDH1,MECR,MEMO1,MMP1,MMP2,MSH2,MSN,MUC1,MYBPC1,MYBPC3,NBEA,NCBP1,NCOA3,NF2,NFATC1,NOTCH1,NR1I3,NSUN5,NUP214,OAT,ODC1,OXSR1,PA2G4,PAFAH1B2,PAH,PANK1,PAPSS1,PARN,PARP3,PATJ,PC,PCYT2,PDK2,PDK3,PDK4,PEX5,PGC,PGK1,PHF24,POT1,POU2F1,PPARG,PPP2R1A,PRKCB,PSAP,PTK2B,PTPRE,PTPRF,PYGL,RAC1,RARA,RARG,RBBP6,RBP5,RGS6,RND1,RPA3,RRM1,RTN4IP1,RUVBL1,SART3,SEC23A,SEM1,SEMA4D,SENP2,SERPINA5,SFMBT2,SH2B2,SH3YL1,SKP1,SLC5A1,SLK,SMAP1,SMG5,SMG7,SMTN,SMURF2,STARD13,STK4,SULT1A1,SYT13,THBS2,TIMM9,TJP1,TLR4,TRIM21,UEVLD,USP19,UTRN,VAV3,WHRN,WWP1,XIAP,XRCC6,ZBTB43,ZCWPW1,ZEB2,ZFP28,ZNF292,ZNF462
204	Metastatic uterine cancer	Cancer,Organismal Injury and	1.83E,07	9	ALK,AOC3,AR,ESR1,F3,GAD2,GART,HSP90AB1,RRM1

		Abnormalities,Reproductive System Disease			
205	Steroid metabolism	Lipid Metabolism,Small Molecule Biochemistry,Vitamin and Mineral Metabolism	1.93E,07	15	ACACB,ACAT2,CYP17A1,CYP2E1,CYP2R1,CYP7A1,G6PD,GC,GGPS1,HMGCS1,HSD17B10,HSD17B4,IFNG,SULT1A1,YWHAH
206	Female genital tract adenocarcinoma	Cancer,Organismal Injury and Abnormalities,Reproductive System Disease	2.06E,07	147	AASDHPPT,ACACB,ACADSB,ACBD7,ACY1,ALB,ALK,AOC3,APPL1,AR,ARF6,ARHGEF1,ASAP1,ATP7B,B2M,B3GAT2,BCR,BTK,C8A,CA8,CD1A,CLCN5,CLK1,CSDE1,CSF2RB,CSNK1G1,CSTF2,CTCF,CUL1,CYP7A1,DCTD,DOK2,DPP4,DTYMK,EP300,EPHA3,ERBB4,ESR1,ETF1,F2,F3,FAP,FCGR2A,FLNB,FN1,G6PD,GAD2,GART,GRK2,GTF2I,HBB,HBEGF,HCK,HDX,HMBOX1,HMGB3,HNRNPA2B1,HPD,HSD17B4,HSP90AB1,HTR1A,IGF1R,IQUB,ITGB3,ITPA,ITPKC,ITSN1,KIF11,LRBA,MAGEA4,MAGI2,MAP3K3,MAPK6,MARF1,MARK1,MDH1,MEMO1,MMP1,MSN,MUC1,MYBPC3,MYSM1,NBEA,NCBP1,NCOA3,NF2,NFATC1,NMRK1,NMT1,NOTCH1,NR1I3,NT5C3A,NUP214,OAT,ODC1,PANK1,PARN,PARP3,PATJ,PC,PDK3,PDK4,PGC,PGK1,POU2F1,PPARG,PPP2R1A,PTPRE,PTPRF,PYGL,RARA,RARG,RBBP6,RBM39,RGS6,RND1,RRM1,RTN4IP1,RUVBL1,SART3,SENP2,SF3B6,SFMBT2,SH3YL1,SKP1,SLC5A1,SLK,SMG5,SMTN,SMURF2,STK4,SUB1,SYT13,THBS2,TLR4,TRIM21,UEVLD,UTRN,VAV3,WWP1,XIAP,YY1,ZEB2,ZFP28,ZIC3,ZNF292,ZNF462
207	Embryonal tumor	Cancer,Organismal Injury and Abnormalities	2.20E,07	36	ALK,AR,CSF2RB,CTCF,EP300,EPHA3,ERBB4,FABP7,FAP,FCGR2A,FN1,GART,GGPS1,HNRNPA2B1,HSP90AB1,IDE,IFNG,IGF1R,ITGB3,MSH2,NAE1,NBEA,NF2,PA2G4,PGK1,PRKCB,RAC1,RARA,RARG,RRM1,TAF13,THBS2,TLR4,UTRN,VAV3,ZEB2
208	Refractory malignant solid tumor	Cancer,Organismal Injury and Abnormalities	2.25E,07	14	ALB,ALK,AR,BTK,ERBB4,GART,HDAC7,HMOX1,HSP90AB1,IMPDH1,PARP3,PTK2B,RRM1,VEGFB
209	Malignant solid organ tumor	Cancer,Organismal Injury and Abnormalities	2.27E,07	209	ACACB,ACADSB,ACSM2A,ADH7,ALB,ALK,ALOX12,AMY1C (includes others),ANG,AOC3,APEX1,APPL1,AR,ARHGAP5,ARHGEF1,ASAP1,ATP7B,ATXN3,B2M,BCR,BMP1,BPI,BTK,BTRC,C1R,C8A,CA8,CAMK4,CANT1,CD1A,CD74,CHKA,CLCN5,CRYM,CSDE1,CSF2RB,CSNK1G2,CSTF2,CTCF,CYP17A1,CYP2E1,CYP7A1,DAPK2,DCTD,DFFA,DGKA,DHPS,DMC1,DOK2,DPP4,DSG2,ECI2,EP300,EPHA3,EPS8L2,ERBB4,ESR1,F2,F3,FABP3,FAP,FAS,FCGR2A,FHIT,FLNB,FN1,GAD2,GALM,GALNT10,GART,GC,GGPS1,GRHPR,GRK2,GRK6,GTF2I,HASPIN,HAT1,HBB,HBEGF,HCK,HDAC7,HDX,HK2,HLA,DRB1,HMGCS1,HNRNPA2B1,HPD,HSCB,HSD17B10,HSP90AB1,HTR1A,IFNG,IFNGR1,IGF1R,IMPDH1,IQUB,ITGB3,ITPK1,ITPKC,ITSN1,KMT5A,KYNU,LRBA,MAGEA4,MAGI2,MARF1,MARK1,MATK,MDH1,MECR,MEMO1,MMP1,MMP2,MSH2,MSN,MUC1,MYBPC1,MYBPC3,NBEA,NCBP1,NCOA3,NF2,NFATC1,NOTCH1,NR1I3,NSUN5,NUP214,OAT,ODC1,OXSR1,PA2G4,PAFAH1B2,PAH,PANK1,PAPSS1,PARN,PARP3,PATJ,PC,PCYT2,PDK2,PDK3,PDK4,PEX5,PGC,PGK1,PHF24,POT1,POU2F1,PPARG,PPP2R1A,PRKCB,PSAP,PTK2B,PTPRE,PTPRF,PYGL,RAC1,RARA,RARG,RBBP6,RBP5,RGS6,RND1,RPA3,RRM1,RTN4IP1,RUVBL1,SART3,SEC23A,SEM1,SEMA4D,SENP2,SERPINA5,SFMBT2,SH2B2,SH3YL1,SKP1,SLC5A1,SLK,SMAP1,SMG5,SMG7,SMTN,SMURF2,STARD13,STK4,SULT1A1,SYT13,THBS2,TIMM9,TJP1,TLR4,TRIM21,UEVLD,USP19,UTRN,VAV3,WHRN,WWP1,XIAP,XRCC6,ZBTB43,ZCWPW1,ZEB2,ZFP28,ZNF292,ZNF462
210	Binding of DNA	Cell Cycle,Gene Expression	2.48E,07	22	ALB,APEX1,AR,BTK,CYP2E1,EP300,ESR1,F2,F3,FN1,HCK,IFNG,MAP3K3,MUC1,NOTCH1,NR1I3,POU2F1,PPARG,RAC1,RARA,STK4,TLR4
211	Peripheral vascular disease	Cardiovascular Disease,Organismal Injury and Abnormalities	2.64E,07	26	ALB,AOC3,AR,B2M,C1R,CA8,CSF2RB,DHPS,ESR1,F2,FABP3,FAS,FCGR2A,HBB,HCK,HIBCH,HLA,DRB1,HTR1A,IGF1R,ITGB3,MARK1,PAH,PPARG,PRKCB,PYGL,SUB1
212	Adhesion of T lymphocytes	Cell,mediated Immune Response,Cell,To,Cell Signaling and Interaction,Cellular Movement,Hematological System Development and Function,Immune Cell Trafficking	2.76E,07	9	AOC3,ARHGEF1,F2,FAS,FN1,IFNG,PTK2B,RAC1,TLR4

213	Transport of molecule	Molecular Transport	2.97E,07	45	ALB,ANG,ANXA6,APPL1,ARF6,ATP7B,B2M,CD74,CERT1,CHKA,CLCN5,CRYM,CUL1,CYP2E1,F2,FABP3,FAS,FCGR2A,FN1,GAD2,GC,HBB,HMOX1,HNRNPA2B1,IFNG,IGF1R,ITGB3,MSN,NCBP1,NUP214,PPARG,PRKCB,PSAP,SKP1,SLC5A1,SMG5,SMG7,SNX1,STK4,TIMM9,TTR,USP19,WARS1,WWP1,YWHAH
214	Transcription	Gene Expression	3.06E,07	59	ACVR2B,ANG,APEX1,AR,ATXN3,BTK,BTRC,CAMK4,CD74,CDK5R1,CIAO1,CRYM,CTCF,EP300,ERBB4,ESR1,F2,F3,FN1,GTF2F2,GTF2I,HBB,HDAC7,HLA,DRB1,HMBOX1,HNRNPA2B1,IFNG,KMT5A,MAP3K3,MSN,MYSM1,NCBP1,NCOA3,NFATC1,NOTCH1,NR1I3,PA2G4,PC,POU2F1,PPARG,PRKCB,RAC1,RARA,RARG,RBBP6,SART3,SEMA4D,SFMBT2,SMURF2,SUB1,TAF13,TLR4,WARS1,XRCC6,YWHAH,YWHAQ,YY1,ZEB2,ZIC3
215	Binding of T lymphocytes	Cell,To,Cell Signaling and Interaction,Hematological System Development and Function	3.10E,07	10	AOC3,ARHGEF1,F2,FAS,FN1,IFNG,MSN,PTK2B,RAC1,TLR4
216	Malignant neoplasm of retroperitoneum	Cancer,Organismal Injury and Abnormalities	3.17E,07	106	ACACB,ACAT2,ALB,ALK,APPL1,AR,ASAP1,ATP7B,ATXN3,B2M,BPI,BTRC,CANT1,CD74,CDK5R1,CHKA,CLK1,CRYM,CSDE1,CSF2RB,CSTF2,CTCF,CYP17A1,DCTD,DFFA,DPP4,ECHS1,EP300,EPHA3,ERBB4,ESR1,F2,F3,FAP,FLNB,FN1,G6PD,GC,GGPS1,GRHPR,GTF2I,HBB,HBEGF,HCK,HIBCH,HLA,DRB1,HMGCS1,HMOX1,HPD,HSD17B4,HSP90AB1,IGF1R,IMPDH1,ITGB3,ITSN1,KIF11,KLK1,LRBA,MAGI2,MARK1,MMP2,MSH2,MUC1,MYBPC1,MYBPC3,NBEA,NCOA3,NF2,NFATC1,NOTCH1,NR1I3,NUP214,PA2G4,PANK1,PARP3,PC,PDHA1,PDK4,PGK1,POT1,POU2F1,PPARG,PPP2R1A,PRKCB,PTPRE,PYGL,RARA,RARG,RBBP6,RBM39,RBP5,RRM1,S100A6,SFN,SMG7,SMTN,SULT1A1,TJP1,TLR4,UTRN,WEE1,ZCWPW1,ZEB2,ZFP28,ZNF292,ZNF462
217	Differentiation of tumor cell lines	Cellular Development	3.33E,07	18	CDK5R1,CHKA,CTCF,DPP4,ERBB4,ESR1,FABP7,FN1,HMOX1,HNRNPA2B1,IFNG,IGF1R,KMT5A,MUC1,NOTCH1,PPARG,RARA,RARG
218	Abdominal cancer	Cancer,Organismal Injury and Abnormalities	3.33E,07	266	AASDHPPT,ACACB,ACADSB,ACADVL,ACAT2,ACBD7,ACP3,ACSM2A,ACVR2B,ACY1,ADH1C,ADH7,ALB,ALK,ALOX12,ANG,ANXA6,AOC3,APEX1,APPL1,AR,ARF6,ARHGAP5,ARHGEF1,ASAP1,ATP7B,ATXN3,B2M,B3GAT2,BCR,BMP1,BPI,BTK,BTRC,C1R,C8A,CA8,CAMK4,CANT1,CD1A,CD74,CDK5R1,CHKA,CLCN5,CLK1,CRYM,CSDE1,CSF2RB,CSNK1G1,CSNK1G2,CSTF2,CTCF,CUL1,CYP17A1,CYP2E1,CYP7A1,DAPK2,DCTD,DCXR,DFFA,DGKA,DHPS,DMC1,DOK2,DPP4,DSG2,DTYMK,ECHS1,ECI2,EP300,EPHA3,EPS8L2,ERBB4,ESR1,ETF1,EXOSC9,F2,F3,FABP7,FAP,FAS,FCGR2A,FHIT,FLNB,FN1,G6PD,GAD2,GALM,GALNT10,GART,GC,GGPS1,GRHPR,GRK2,GRK6,GTF2F2,GTF2I,HADH,HASPIN,HBB,HBEGF,HCK,HDAC7,HDX,HIBCH,HK2,HLA,DRB1,HMBOX1,HMGB3,HMGCS1,HMOX1,HNRNPA2B1,HPD,HSCB,HSD17B10,HSD17B4,HSP90AB1,HTR1A,IDE,IFNG,IFNGR1,IGF1R,IMPA2,IMPDH1,IQUB,ITGB3,ITPA,ITPK1,ITPKC,ITSN1,KIF11,KLK1,KMT5A,KYNU,LRBA,MAGEA4,MAGI2,MAP3K3,MAPK6,MARF1,MARK1,MATK,MDH1,MECR,MEMO1,MMP1,MMP2,MSH2,MSN,MTAP,MUC1,MYBPC1,MYBPC3,MYSM1,NAE1,NBEA,NCBP1,NCOA3,NF2,NFATC1,NMRK1,NMT1,NOTCH1,NR1I3,NSUN5,NT5C3A,NUP214,OAT,ODC1,OXSR1,PA2G4,PAFAH1B2,PAH,PANK1,PAPSS1,PARN,PARP3,PATJ,PC,PCYT2,PDHA1,PDK2,PDK3,PDK4,PEX5,PGC,PGK1,PHF24,POT1,POU2F1,PPARG,PPP2R1A,PRKCB,PSAP,PTK2B,PTPRE,PTPRF,PUS10,PYGL,RAB26,RAC1,RARA,RARG,RBBP6,RBM39,RBP5,RGS6,RND1,RRM1,RTN4IP1,RUVBL1,S100A6,SART3,SEC23A,SEMA4D,SENP2,SERPINA5,SF3B6,SFMBT2,SFN,SH2B2,SH3YL1,SKP1,SLC5A1,SLK,SMAP1,SMG5,SMG7,SMTN,SMURF2,SNX1,SPEF1,STARD13,STK16,STK4,SUB1,SULT1A1,SYT13,TAF13,THBS2,TJP1,TLR4,TNFAIP8L2,TRIM21,TTR,UEVLD,USP19,UTRN,VAV3,VEGFB,VPS26A,WEE1,WHRN,WWP1,XIAP,XRCC6,YWHAH,YWHAQ,YY1,ZBTB43,ZCWPW1,ZEB2,ZFP28,ZIC3,ZNF292,ZNF462
219	Prostatic tumor	Cancer,Organismal Injury and Abnormalities,Reproductive System Disease	3.58E,07	117	ACACB,ACADVL,ACP3,ALB,ALK,ANXA6,APEX1,APPL1,AR,ARHGAP5,ARHGEF1,B2M,BCR,BTK,C1R,CANT1,CD74,CLK1,CSDE1,CSNK1G1,CUL1,CYP17A1,CYP2E1,CYP7A1,DCTD,DMC1,EP300,EPHA3,EPS8L2,ERBB4,ESR1,F2,FABP7,FAP,FAS,FLNB,FN1,GAD2,GALM,GALNT10,GART,GC,GGPS1,HASPIN,HDAC7,HLA,DRB1,HMGB3,HMOX1,HSP90AB1,IFNG,IFNGR1,ITGB3,KMT5A,LRBA,MAGI2,MAPK6,MARF1,MARK1,MATK,MDH1,MMP1,MMP2,MSH2,MUC1,MYBPC1,MYBPC3,NBEA,NCOA3,NF2,NFATC1,NOTCH1,NUP214,ODC1,OXSR1,PAPSS1,PARP3,PC,PDK3,PDK4,PGK1,PHF24,POU2F1,PPARG,PPP2R1A,PRKCB,PSAP,PTK2B,PTPRE,PTPRF,PYGL,RARA,RARG,RRM1,SEMA4D,SENP2,SERPINA5,SF3B6,SFMBT2,SFN,SLK,SMAP1,STK4,TAF13,THBS2,TLR4,TRIM21,TTR,USP19,UTRN,VEGFB,WHRN,XIAP,YWHAQ,YY1,ZEB2,ZNF292,ZNF462

220	Angiogenesis	Cardiovascular System Development and Function,Organismal Development	3.59E,07	30	ANG,BPI,DGKA,DSG2,F2,F3,FLNB,FN1,GTF2I,HBEGF,HK2,HMOX1,IFNG,ITGB3,KLK1,MMP2,NFATC1,NOTCH1,PATJ,PGK1,PPARG,RAC1,STARD13,THBS2,TJP1,TLR4,VEGFB,WARS1,YARS1,YY1
221	Metabolism of terpenoid	Lipid Metabolism,Small Molecule Biochemistry,Vitamin and Mineral Metabolism	3.66E,07	17	ACACB,ACAT2,ADH7,CYP17A1,CYP2E1,CYP2R1,CYP7A1,G6PD,GC,GGPS1,HMGCS1,HSD17B10,HSD17B4,IFNG,SULT1A1,TTR,YWHAH
222	Migration of mononuclear leukocytes	Cellular Movement,Hematological System Development and Function,Immune Cell Trafficking	3.75E,07	15	AOC3,ARHGEF1,CD74,DPP4,F2,FAS,FN1,IFNG,ITGB3,MSN,PPARG,PTK2B,RAC1,SEMA4D,TLR4
223	Cell death of embryonic cell lines	Cell Death and Survival,Embryonic Development	4.02E,07	18	AR,ATXN3,F2,FAS,GRK2,HK2,IFNG,IGF1R,NDUFAB1,NF2,PEX5,PPARG,PRKCB,STK4,TNFAIP8L2,XIAP,YWHAQ,YY1
224	Oxidation of fatty acid	Energy Production,Lipid Metabolism,Small Molecule Biochemistry	4.27E,07	10	ACADVL,ADH1C,ALOX12,ECHS1,HADH,HSD17B10,HSD17B4,MECR,PDK4,PPARG
225	Cell movement of endothelial cell lines	Cardiovascular System Development and Function,Cellular Movement	4.37E,07	11	ESR1,F3,FN1,GTF2I,MMP2,PATJ,PPARG,PTK2B,TLR4,VEGFB,YARS1
226	Progressive adenocarcinoma	Cancer,Organismal Injury and Abnormalities,Tumor Morphology	4.37E,07	11	ALK,AR,CYP17A1,ERBB4,ESR1,GART,HSP90AB1,IGF1R,PARP3,PTK2B,RRM1
227	Progressive neurological disorder	Neurological Disease	4.68E,07	50	ADH1C,ALB,ANG,AR,BCR,BTK,C1R,CD74,CDK5R1,ERBB4,ESR1,F2,F3,FABP3,FAS,FCGR2A,FHIT,GAD2,GART,GC,GGPS1,HDAC7,HLA,DRB1,HMOX1,HNRNPA2B1,HSP90AB1,HTR1A,IDE,IFNG,IGF1R,IMPDH1,ITGB3,MDH1,MMP1,MMP2,NAE1,NOTCH1,NUP214,ODC1,PPARG,PRKCB,PSAP,PTPRE,RRM1,S100A6,SMTN,TAF13,TLR4,TTR,XIAP
228	Liver carcinoma	Cancer,Gastrointestinal Disease,Hepatic System Disease,Organismal Injury and Abnormalities	4.70E,07	133	ACACB,ACADSB,ACADVL,ACY1,ALB,ALK,APEX1,AR,ARHGAP5,ASAP1,ATXN3,B2M,B3GAT2,BCR,BTK,BTRC,C1R,C8A,CANT1,CD1A,CDK5R1,CLCN5,CLK1,CSDE1,CSF2RB,CSNK1G1,CTCF,CYP7A1,DGKA,ECHS1,ECI2,EP300,EPHA3,EPS8L2,ERBB4,ESR1,EXOSC9,F2,FABP7,FAP,FLNB,FN1,GC,GGPS1,GRHPR,GTF2F2,HBB,HCK,HDAC7,HDX,HIBCH,HK2,HMGB3,HMGCS1,HMOX1,HNRNPA2B1,HPD,HSP90AB1,HTR1A,IDE,IFNGR1,IGF1R,IMPDH1,IQUB,KMT5A,KYNU,MAGI2,MAPK6,MARF1,MDH1,MECR,MMP2,MSH2,MUC1,MYBPC1,MYBPC3,NAE1,NBEA,NCBP1,NCOA3,NF2,NFATC1,NMT1,NOTCH1,NUP214,PATJ,PC,PCYT2,PDHA1,PDK3,PDK4,PEX5,PGK1,PHF24,POT1,POU2F1,PPARG,PPP2R1A,PRKCB,PSAP,PTPRE,PTPRF,PYGL,RAC1,RARA,RBBP6,RRM1,SART3,SEC23A,SFMBT2,SLK,SMG7,SMURF2,STARD13,SULT1A1,THBS2,TJP1,TRIM21,UEVLD,USP19,VAV3,VEGFB,WEE1,WWP1,XRCC6,YWHAH,YY1,ZBTB43,ZCWPW1,ZEB2,ZIC3,ZNF292,ZNF462
229	Microtubule dynamics	Cellular Assembly and Organization,Cellular Function and Maintenance	4.81E,07	30	AR,ARF6,ASAP1,ATXN3,BTK,CAMK4,CDK5R1,EPHA3,ERBB4,ESR1,F2,F3,FAS,FHIT,FN1,GRK2,HCK,IFNG,ITGB3,KIF11,MATK,MSN,NF2,PRKCB,PTPRF,RAC1,SEMA4D,SLK,SPEF1,TLR4
230	Progression of carcinoma	Cancer,Organismal Injury and Abnormalities,Tumor Morphology	4.84E,07	12	ALK,AR,CYP17A1,ERBB4,ESR1,F3,GART,HSP90AB1,IGF1R,PARP3,PTK2B,RRM1

231	Binding of lymphatic system cells	Cell,To,Cell Signaling and Interaction	5.22E,07	11	AOC3,ARHGEF1,F2,FAS,FN1,IFNG,ITGB3,MSN,PTK2B,RAC1,TLR4
232	Metabolic bone disease	Connective Tissue Disorders,Metabolic Disease,Organismal Injury and Abnormalities,Skeletal and Muscular Disorders	5.25E,07	10	ALB,BMP1,C1R,CLCN5,CYP2R1,ESR1,GGPS1,HNRNPA2B1,NOTCH1,PEX5
233	Cytostasis	Cellular Growth and Proliferation	5.61E,07	15	ALK,CUL1,ESR1,FABP7,FAS,FHIT,GRK2,IFNG,IGF1R,NF2,NME3,NOTCH1,RAC1,RARA,RARG
234	Hepatobiliary carcinoma	Cancer,Gastrointestinal Disease,Organismal Injury and Abnormalities	5.86E,07	139	ACACB,ACADSB,ACADVL,ACY1,ADH1C,ALB,ALK,APEX1,AR,ARHGAP5,ASAP1,ATXN3,B2M,B3GAT2,BCR,BTK,BTRC,C1R,C8A,CANT1,CD1A,CDK5R1,CLCN5,CLK1,CSDE1,CSF2RB,CSNK1G1,CTCF,CYP7A1,DGKA,DSG2,ECHS1,ECI2,EP300,EPHA3,EPS8L2,ERBB4,ESR1,EXOSC9,F2,FABP7,FAP,FLNB,FN1,GC,GGPS1,GRHPR,GTF2F2,HBB,HCK,HDAC7,HDX,HIBCH,HK2,HMGB3,HMGCS1,HMOX1,HNRNPA2B1,HPD,HSP90AB1,HTR1A,IDE,IFNGR1,IGF1R,IMPDH1,IQUB,KIF11,KMT5A,KYNU,MAGI2,MAPK6,MARF1,MDH1,MECR,MMP2,MSH2,MUC1,MYBPC1,MYBPC3,NAE1,NBEA,NCBP1,NCOA3,NF2,NFATC1,NMT1,NOTCH1,NUP214,PA2G4,PATJ,PC,PCYT2,PDHA1,PDK3,PDK4,PEX5,PGK1,PHF24,POT1,POU2F1,PPARG,PPP2R1A,PRKCB,PSAP,PTPRE,PTPRF,PYGL,RAC1,RARA,RBBP6,RRM1,SART3,SEC23A,SFMBT2,SH2B2,SLK,SMG7,SMURF2,STARD13,SULT1A1,THBS2,TJP1,TLR4,TRIM21,UEVLD,USP19,VAV3,VEGFB,WEE1,WWP1,XRCC6,YWHAH,YY1,ZBTB43,ZCWPW1,ZEB2,ZIC3,ZNF292,ZNF462
235	Prostate cancer	Cancer,Organismal Injury and Abnormalities,Reproductive System Disease	5.89E,07	116	ACACB,ACADVL,ACP3,ALB,ALK,ANXA6,APEX1,APPL1,AR,ARHGAP5,ARHGEF1,B2M,BCR,BTK,C1R,CANT1,CD74,CLK1,CSDE1,CSNK1G1,CUL1,CYP17A1,CYP2E1,CYP7A1,DCTD,DMC1,EP300,EPHA3,EPS8L2,ERBB4,ESR1,F2,FABP7,FAP,FAS,FLNB,FN1,GAD2,GALM,GALNT10,GART,GC,GGPS1,HASPIN,HDAC7,HLA,DRB1,HMGB3,HMOX1,HSP90AB1,IFNG,IFNGR1,ITGB3,KMT5A,LRBA,MAGI2,MAPK6,MARF1,MARK1,MATK,MDH1,MMP1,MMP2,MSH2,MUC1,MYBPC1,MYBPC3,NBEA,NCOA3,NF2,NFATC1,NOTCH1,NUP214,ODC1,OXSR1,PAPSS1,PARP3,PC,PDK3,PDK4,PGK1,PHF24,POU2F1,PPARG,PPP2R1A,PRKCB,PSAP,PTK2B,PTPRE,PTPRF,PYGL,RARA,RARG,RRM1,SEMA4D,SENP2,SERPINA5,SF3B6,SFMBT2,SFN,SLK,SMAP1,STK4,TAF13,THBS2,TLR4,TRIM21,TTR,USP19,UTRN,WHRN,XIAP,YWHAQ,YY1,ZEB2,ZNF292,ZNF462
236	Adhesion of immune cells	Cell,To,Cell Signaling and Interaction,Hematological System Development and Function,Immune Cell Trafficking	5.93E,07	15	AOC3,ARHGEF1,F2,F3,FAS,FCGR2A,FN1,IFNG,MSN,PPARG,PTK2B,RAC1,SEMA4D,TLR4,UTRN
237	Liquid tumor	Cancer,Organismal Injury and Abnormalities	6.04E,07	84	ALB,ALK,ALOX12,AMY1C (includes others),APEX1,ARHGAP5,B2M,BCR,BMP1,BTK,BTRC,C8A,CANT1,CD1A,CD74,CDK5R1,CIAO1,CSF2RB,CTCF,CYP2E1,CYP2R1,DPP4,EP300,EPHA3,ESR1,ETF1,F2,F3,FAP,FAS,FCGR2A,GAD2,GRK2,GTF2F2,HASPIN,HCK,HLA,DRB1,HMGB3,HNRNPA2B1,HSP90AB1,IFNG,IGF1R,IMPDH1,ITGB3,KLK1,KMT5A,LRBA,MAGEA4,MAGI2,MMP2,MSH2,NCOA3,NOTCH1,NT5C3A,NUP214,PA2G4,PDHA1,PDK3,PDK4,PGC,POT1,PRKCB,PTK2B,PTPRE,PYGL,RAC1,RARA,RARG,RRM1,SH2B2,SMG5,SMG7,SMTN,STK4,SYT13,TAF13,TJP1,TLR4,TXNDC12,UEVLD,VEGFB,WEE1,XIAP,ZEB2
238	Chemotaxis	Cellular Movement	6.24E,07	22	CD74,CERT1,DAPK2,DGKA,DPP4,ERBB4,FAS,FN1,HBEGF,IFNG,ITGB3,MMP2,NOTCH1,PPARG,PRKCB,PTK2B,RAC1,SEMA4D,THBS2,TLR4,WARS1,YARS1
239	Organization of cytoskeleton	Cellular Assembly and Organization,Cellular Function and Maintenance	6.50E,07	35	AR,ARF6,ASAP1,ATXN3,BTK,CAMK4,CDK5R1,EPHA3,ERBB4,ESR1,F2,F3,FAS,FCGR2A,FHIT,FN1,GRK2,HCK,IFNG,ITGB3,KIF11,MATK,MSN,NDC80,NF2,PARP3,PRKCB,PTPRF,RAC1,RND1,SEMA4D,SLK,SPEF1,TJP1,TLR4
240	Transcription of RNA	Gene Expression	7.02E,07	53	ACVR2B,ANG,APEX1,AR,ATXN3,BTK,BTRC,CAMK4,CD74,CDK5R1,CIAO1,CRYM,CTCF,EP300,ERBB4,ESR1,F2,FN1,GTF2F2,GTF2I,HBB,HDAC7,HLA,DRB1,HMBOX1,HNRNPA2B1,IFNG,KMT5A,MAP3K3,MYSM1,

					NCBP1,NCOA3,NFATC1,NOTCH1,NR1I3,PA2G4,POU2F1,PPARG,PRKCB,RAC1,RARA,RARG,RBBP6,SEMA4D,SMURF2,SUB1,TAF13,TLR4,XRCC6,YWHAH,YWHAQ,YY1,ZEB2,ZIC3
241	Apoptosis of pancreatic cancer cell lines	Cell Death and Survival	7.10E,07	10	BTRC,FAS,FHIT,FN1,HBEGF,IFNG,IGF1R,NOTCH1,RRM1,SFN
242	Necrosis of epithelial tissue	Cell Death and Survival,Organismal Injury and Abnormalities	7.24E,07	23	AR,ATXN3,BPI,F2,FAS,GRK2,HK2,IFNG,IGF1R,ITSN1,NDUFAB1,NF2,NOTCH1,PEX5,PPARG,PRKCB,RAC1,STK4,TLR4,TNFAIP8L2,XIAP,YWHAQ,YY1
243	Intraabdominal organ tumor	Cancer,Organismal Injury and Abnormalities	7.35E,07	264	AASDHPPT,ACACB,ACADSB,ACADVL,ACAT2,ACBD7,ACSM2A,ACVR2B,ACY1,ADH7,ALB,ALK,ALOX12,ANG,ANXA6,AOC3,APEX1,APPL1,AR,ARF6,ARHGAP5,ARHGEF1,ASAP1,ATP7B,ATXN3,B2M,B3GAT2,BCR,BMP1,BPI,BTK,BTRC,C1R,C8A,CA8,CAMK4,CANT1,CD1A,CD74,CDK5R1,CHKA,CLCN5,CLK1,CRYM,CSDE1,CSF2RB,CSNK1G1,CSNK1G2,CSTF2,CTCF,CUL1,CYP17A1,CYP2E1,CYP7A1,DAPK2,DCTD,DCXR,DFFA,DGKA,DHPS,DMC1,DOK2,DPP4,DSG2,DTYMK,ECHS1,ECI2,EP300,EPHA3,EPS8L2,ERBB4,ERI1,ESR1,ETF1,EXOSC9,F2,F3,FABP7,FAP,FAS,FCGR2A,FHIT,FLNB,FN1,G6PD,GAD2,GALNT10,GART,GC,GGPS1,GRHPR,GRK2,GRK6,GTF2F2,GTF2I,HADH,HASPIN,HBB,HBEGF,HCK,HDAC7,HDX,HIBCH,HK2,HLA,DRB1,HMBOX1,HMGB3,HMGCS1,HMOX1,HNRNPA2B1,HPD,HSCB,HSD17B10,HSD17B4,HSP90AB1,HTR1A,IDE,IFNG,IFNGR1,IGF1R,IMPA2,IMPDH1,IQUB,ITGB3,ITPA,ITPK1,ITPKC,ITSN1,KIF11,KLK1,KMT5A,KYNU,LRBA,MAGEA4,MAGI2,MAP3K3,MAPK6,MARF1,MARK1,MATK,MDH1,MECR,MEMO1,MMP1,MMP2,MSH2,MSN,MTAP,MUC1,MYBPC1,MYBPC3,MYSM1,NAE1,NBEA,NCBP1,NCOA3,NF2,NFATC1,NMRK1,NMT1,NOTCH1,NR1I3,NSUN5,NT5C3A,NUP214,OAT,ODC1,OXSR1,PA2G4,PAFAH1B2,PAH,PANK1,PAPSS1,PARN,PARP3,PATJ,PC,PCYT2,PDHA1,PDK2,PDK3,PDK4,PEX5,PGC,PGK1,PHF24,POT1,POU2F1,PPARG,PPP2R1A,PRKCB,PSAP,PTK2B,PTPRE,PTPRF,PUS10,PYGL,RAB26,RAC1,RARA,RARG,RBBP6,RBM39,RBP5,RGS6,RND1,RRM1,RTN4IP1,RUVBL1,S100A6,SART3,SEC23A,SEMA4D,SENP2,SERPINA5,SF3B6,SFMBT2,SFN,SH2B2,SH3YL1,SKP1,SLC5A1,SLK,SMAP1,SMG5,SMG7,SMTN,SMURF2,SNX1,SNX22,SPEF1,STARD13,STK16,STK4,SUB1,SULT1A1,SYT13,TAF13,THBS2,TJP1,TLR4,TNFAIP8L2,TRIM21,TTR,UEVLD,USP19,UTRN,VAV3,VEGFB,VPS26A,WEE1,WHRN,WWP1,XIAP,XRCC6,YWHAH,YY1,ZBTB43,ZCWPW1,ZEB2,ZFP28,ZIC3,ZNF292,ZNF462
244	Male genital neoplasm	Cancer,Organismal Injury and Abnormalities,Reproductive System Disease	7.43E,07	118	ACACB,ACADVL,ACP3,ALB,ALK,ANXA6,APEX1,APPL1,AR,ARHGAP5,ARHGEF1,B2M,BCR,BTK,C1R,CANT1,CD74,CLK1,CSDE1,CSNK1G1,CUL1,CYP17A1,CYP2E1,CYP7A1,DCTD,DMC1,EP300,EPHA3,EPS8L2,ERBB4,ESR1,F2,FABP7,FAP,FAS,FLNB,FN1,GAD2,GALM,GALNT10,GART,GC,GGPS1,HASPIN,HDAC7,HLA,DRB1,HMGB3,HMOX1,HSP90AB1,IFNG,IFNGR1,ITGB3,KMT5A,LRBA,MAGI2,MAPK6,MARF1,MARK1,MATK,MDH1,MMP1,MMP2,MSH2,MUC1,MYBPC1,MYBPC3,NBEA,NCOA3,NF2,NFATC1,NOTCH1,NUP214,ODC1,OXSR1,PAPSS1,PARP3,PC,PDK3,PDK4,PGK1,PHF24,POU2F1,PPARG,PPP2R1A,PRKCB,PSAP,PTK2B,PTPRE,PTPRF,PYGL,RAC1,RARA,RARG,RRM1,SEMA4D,SENP2,SERPINA5,SF3B6,SFMBT2,SFN,SLK,SMAP1,STK4,TAF13,THBS2,TLR4,TRIM21,TTR,USP19,UTRN,VEGFB,WHRN,XIAP,YWHAQ,YY1,ZEB2,ZNF292,ZNF462
245	Endometrioid carcinoma	Cancer,Organismal Injury and Abnormalities	7.68E,07	135	AASDHPPT,ACACB,ACADSB,ACBD7,ACY1,ALB,ALK,AOC3,APPL1,AR,ARHGEF1,ASAP1,ATP7B,B2M,B3GAT2,BCR,BTK,C8A,CA8,CD1A,CLCN5,CLK1,CSDE1,CSF2RB,CSNK1G1,CSTF2,CTCF,CUL1,CYP7A1,DCTD,DOK2,DPP4,DTYMK,EP300,EPHA3,ERBB4,ESR1,ETF1,FAP,FCGR2A,FLNB,FN1,G6PD,GAD2,GRK2,GTF2I,HBEGF,HDX,HMBOX1,HMGB3,HNRNPA2B1,HPD,HSD17B4,HSP90AB1,HTR1A,IGF1R,IQUB,ITGB3,ITPA,ITPKC,KIF11,LRBA,MAGEA4,MAGI2,MAP3K3,MAPK6,MARF1,MARK1,MDH1,MEMO1,MMP1,MSN,MYBPC3,MYSM1,NBEA,NCBP1,NCOA3,NF2,NFATC1,NMRK1,NMT1,NOTCH1,NR1I3,NT5C3A,NUP214,OAT,ODC1,PANK1,PARN,PARP3,PATJ,PC,PDK3,PGK1,POU2F1,PPARG,PPP2R1A,PTPRE,PTPRF,RARA,RARG,RBBP6,RBM39,RGS6,RND1,RRM1,RTN4IP1,RUVBL1,SART3,SENP2,SF3B6,SFMBT2,SH3YL1,SLC5A1,SLK,SMG5,SMTN,SMURF2,STK4,SUB1,SYT13,THBS2,TLR4,TRIM21,UEVLD,UTRN,VAV3,WWP1,XIAP,YY1,ZEB2,ZFP28,ZIC3,ZNF292,ZNF462
246	Primary tumor	Cancer,Organismal Injury and Abnormalities	7.72E,07	25	ALB,ALK,B2M,BTK,CDK5R1,CSF2RB,CSNK1G2,DOK2,ERBB4,ESR1,FN1,GART,HSP90AB1,IGF1R,MUC1,NF2,PARP3,PPARG,PPP2R1A,RAC1,RARA,RARG,RRM1,SEMA4D,VEGFB
247	Female genital tract serous carcinoma	Cancer,Organismal Injury and	7.83E,07	24	ALK,AOC3,AR,B2M,BCR,CTCF,DPP4,EP300,EPHA3,F3,HBB,HSP90AB1,KIF11,MUC1,NOTCH1,PARP3,PDK4,PPP2R1A,RRM1,SKP1,SMTN,THBS2,TLR4,VAV3

		Abnormalities,Reproductive System Disease			
248	Pelvic serous carcinoma	Cancer,Organismal Injury and Abnormalities	7.83E,07	24	ALK,AOC3,AR,B2M,BCR,CTCF,DPP4,EP300,EPHA3,F3,HBB,HSP90AB1,KIF11,MUC1,NOTCH1,PARP3,PDK4,PPP2R1A,RRM1,SKP1,SMTN,THBS2,TLR4,VAV3
249	Central nervous system solid tumor	Cancer,Neurological Disease,Organismal Injury and Abnormalities	7.96E,07	186	ACACB,ACADSB,ACADVL,ACVR2B,ACY1,ADH7,ALB,ALK,ALOX12,AMY1C (includes others),AOC3,APEX1,APPL1,ARHGAP5,ARHGEF1,ASAP1,ATP7B,ATXN3,B3GAT2,BCR,BMP1,BPI,BTK,C1R,C8A,CLCN5,CRYM,CSDE1,CSF2RB,CSNK1G1,CSNK1G2,CSTF2,CTCF,CUL1,CYP2E1,DAPK2,DCXR,DGKA,DOK2,DPP4,DSG2,EP300,EPHA3,EPS8L2,ERBB4,ESR1,F2,FABP7,FAS,FCGR2A,FLNB,FN1,G6PD,GAD2,GALNT10,GART,GRK2,GRK6,GTF2I,HADH,HASPIN,HAT1,HBEGF,HDX,HIBCH,HK2,HLA,DRB1,HMBOX1,HMGB3,HMGCS1,HMOX1,HNRNPA2B1,HSD17B10,HSD17B4,HSP90AB1,IDE,IFNG,IFNGR1,IGF1R,IMPDH1,ITGB3,ITPK1,ITSN1,KLK1,KMT5A,KYNU,LRBA,MAGEA4,MAGI2,MAP3K3,MARF1,MATK,MMP2,MSH2,MSN,MTAP,MUC1,MYBPC1,MYBPC3,NAE1,NBEA,NCBP1,NCOA3,NF2,NFATC1,NME3,NMT1,NOTCH1,NR1I3,NSUN5,NUP214,ODC1,OXSR1,PA2G4,PAFAH1B2,PAH,PANK1,PAPSS1,PARN,PATJ,PC,PCYT2,PDHA1,PDK4,PGC,PGK1,PHF24,PIP4K2C,POT1,POU2F1,PPARG,PPP2R1A,PRKCB,PSAP,PTK2B,PTPRE,PTPRF,PUS10,PYGL,RAC1,RARA,RARG,RBBP6,RBM39,RBP5,RGS6,RRM1,RTN4IP1,SART3,SEMA4D,SENP2,SERPINA5,SFMBT2,SH2B2,SH3YL1,SLC5A1,SLK,SMG5,SMURF2,SNX1,STARD13,SUB1,TAF13,THBS2,TIMM9,TJP1,TLR4,TNFAIP8L2,USP19,UTRN,VAV3,VEGFB,VPS26A,WEE1,WHRN,XIAP,XRCC6,YWHAH,YWHAQ,ZBTB43,ZCWPW1,ZEB2,ZFP28,ZIC3,ZNF292,ZNF462
250	Abnormality of skin morphology	Dermatological Diseases and Conditions,Organismal Injury and Abnormalities	7.98E,07	22	ALOX12,B2M,CD74,FABP7,FN1,G6PD,HBB,HLA,DRB1,IDE,IFNG,IGF1R,IMPDH1,MMP1,ODC1,PPARG,PRKCB,RAC1,RARA,RARG,RBBP6,RRM1,SART3
251	Lung squamous cell carcinoma	Cancer,Organismal Injury and Abnormalities,Respiratory Disease	8.20E,07	45	ALK,ANXA6,AR,B2M,BTK,C8A,CAMK4,CERT1,CLK1,CUL1,CYP2E1,EP300,EPHA3,ERBB4,FAP,FN1,GART,HASPIN,HDX,HNRNPA2B1,HSP90AB1,IGF1R,ITPKC,MAPK6,MATK,MDH1,NOTCH1,OXSR1,PANK1,PARP3,PATJ,PDK4,PPARG,PPP2R1A,PTK2B,PTPRE,PTPRF,RGS6,RRM1,SENP2,SLK,STK4,TLR4,TTR,WWP1
252	Cell movement of mononuclear leukocytes	Cellular Movement,Hematological System Development and Function,Immune Cell Trafficking	8.31E,07	17	AOC3,ARHGEF1,CD74,DPP4,F2,FAS,FN1,IFNG,ITGB3,MSN,PPARG,PRKCB,PTK2B,RAC1,SEMA4D,TLR4,YARS1
253	Production of superoxide	Free Radical Scavenging	8.42E,07	9	F2,FAS,FCGR2A,G6PD,HBB,IFNG,PRKCB,RAC1,TLR4
254	Malignant neoplasm of male genital organ	Cancer,Organismal Injury and Abnormalities,Reproductive System Disease	8.44E,07	117	ACACB,ACADVL,ACP3,ALB,ALK,ANXA6,APEX1,APPL1,AR,ARHGAP5,ARHGEF1,B2M,BCR,BTK,C1R,CANT1,CD74,CLK1,CSDE1,CSNK1G1,CUL1,CYP17A1,CYP2E1,CYP7A1,DCTD,DMC1,EP300,EPHA3,EPS8L2,ERBB4,ESR1,F2,FABP7,FAP,FAS,FLNB,FN1,GAD2,GALM,GALNT10,GART,GC,GGPS1,HASPIN,HDAC7,HLA,DRB1,HMGB3,HMOX1,HSP90AB1,IFNG,IFNGR1,ITGB3,KMT5A,LRBA,MAGI2,MAPK6,MARF1,MARK1,MATK,MDH1,MMP1,MMP2,MSH2,MUC1,MYBPC1,MYBPC3,NBEA,NCOA3,NF2,NFATC1,NOTCH1,NUP214,ODC1,OXSR1,PAPSS1,PARP3,PC,PDK3,PDK4,PGK1,PHF24,POU2F1,PPARG,PPP2R1A,PRKCB,PSAP,PTK2B,PTPRE,PTPRF,PYGL,RAC1,RARA,RARG,RRM1,SEMA4D,SENP2,SERPINA5,SF3B6,SFMBT2,SFN,SLK,SMAP1,STK4,TAF13,THBS2,TLR4,TRIM21,TTR,USP19,UTRN,WHRN,XIAP,YWHAQ,YY1,ZEB2,ZNF292,ZNF462
255	Progressed metastatic solid tumor	Cancer,Organismal Injury and Abnormalities,Tumor Morphology	8.71E,07	11	ALK,AR,CYP17A1,ERBB4,GART,HSP90AB1,IGF1R,PARP3,PTK2B,RRM1,VEGFB
256	Primary solid tumor	Cancer,Organismal Injury and Abnormalities	8.99E,07	25	ALB,ALK,B2M,BTK,CDK5R1,CSF2RB,CSNK1G2,DOK2,ERBB4,ESR1,FN1,GART,HSP90AB1,IGF1R,MUC1,NF2,PARP3,PPARG,PPP2R1A,RAC1,RARA,RARG,RRM1,SEMA4D,VEGFB
257	Infection of cells	Infectious Diseases	9.27E,07	36	ACACB,ACADSB,ACP3,ARF6,B2M,BCR,BMP1,C1R,CANT1,CD74,DAPK2,DPP4,EP300,ETF1,F2,FAS,FN1,GRK2,HIBCH,HMGCS1,IFNG,IQUB,LARS1,NF2,NMT1,NUP214,PANK1,PRKCB,RAC1,RARA,RND1,SUB1,TLR4,TRIM21,XIAP,ZNF292

258	Abdominal carcinoma	Cancer,Organismal Injury and Abnormalities	9.32E,07	260	AASDHPPT,ACACB,ACADSB,ACADVL,ACAT2,ACBD7,ACSM2A,ACVR2B,ACY1,ADH1C,ADH7,ALB,ALK,ALOX12,ANXA6,AOC3,APEX1,APPL1,AR,ARF6,ARHGAP5,ARHGEF1,ASAP1,ATP7B,ATXN3,B2M,B3GAT2,BCR,BMP1,BPI,BTK,BTRC,C1R,C8A,CA8,CAMK4,CANT1,CD1A,CD74,CDK5R1,CHKA,CLCN5,CLK1,CRYM,CSDE1,CSF2RB,CSNK1G1,CSNK1G2,CSTF2,CTCF,CUL1,CYP17A1,CYP2E1,CYP7A1,DAPK2,DCTD,DCXR,DFFA,DGKA,DHPS,DMC1,DOK2,DPP4,DSG2,DTYMK,ECHS1,ECI2,EP300,EPHA3,EPS8L2,ERBB4,ESR1,ETF1,EXOSC9,F2,F3,FABP7,FAP,FAS,FCGR2A,FHIT,FLNB,FN1,G6PD,GAD2,GALM,GALNT10,GART,GC,GGPS1,GRHPR,GRK2,GRK6,GTF2F2,GTF2I,HADH,HASPIN,HBB,HBEGF,HCK,HDAC7,HDX,HIBCH,HK2,HLA,DRB1,HMBOX1,HMGB3,HMGCS1,HMOX1,HNRNPA2B1,HPD,HSCB,HSD17B10,HSD17B4,HSP90AB1,HTR1A,IDE,IFNG,IFNGR1,IGF1R,IMPA2,IMPDH1,IQUB,ITGB3,ITPA,ITPK1,ITPKC,ITSN1,KIF11,KLK1,KMT5A,KYNU,LRBA,MAGEA4,MAGI2,MAP3K3,MAPK6,MARF1,MARK1,MATK,MDH1,MECR,MEMO1,MMP1,MMP2,MSH2,MSN,MTAP,MUC1,MYBPC1,MYBPC3,MYSM1,NAE1,NBEA,NCBP1,NCOA3,NF2,NFATC1,NMRK1,NMT1,NOTCH1,NR1I3,NSUN5,NT5C3A,NUP214,OAT,ODC1,OXSR1,PA2G4,PAFAH1B2,PAH,PANK1,PAPSS1,PARN,PARP3,PATJ,PC,PCYT2,PDHA1,PDK2,PDK3,PDK4,PEX5,PGC,PGK1,PHF24,POT1,POU2F1,PPARG,PPP2R1A,PRKCB,PSAP,PTK2B,PTPRE,PTPRF,PUS10,PYGL,RAB26,RAC1,RARA,RARG,RBBP6,RBM39,RBP5,RGS6,RND1,RRM1,RTN4IP1,RUVBL1,SART3,SEC23A,SEMA4D,SENP2,SERPINA5,SF3B6,SFMBT2,SFN,SH2B2,SH3YL1,SKP1,SLC5A1,SLK,SMAP1,SMG5,SMG7,SMTN,SMURF2,SNX1,SPEF1,STARD13,STK16,STK4,SUB1,SULT1A1,SYT13,TAF13,THBS2,TJP1,TLR4,TRIM21,UEVLD,USP19,UTRN,VAV3,VEGFB,VPS26A,WEE1,WHRN,WWP1,XIAP,XRCC6,YWHAH,YY1,ZBTB43,ZCWPW1,ZEB2,ZFP28,ZIC3,ZNF292,ZNF462
259	Myelodysplastic syndrome or leukemia	Cancer,Hematological Disease,Organismal Injury and Abnormalities	9.80E,07	82	ALB,ALK,ALOX12,AMY1C (includes others),APEX1,ARHGAP5,B2M,BCR,BTK,BTRC,C8A,CANT1,CD1A,CD74,CDK5R1,CIAO1,CSF2RB,CTCF,CYP2E1,CYP2R1,DPP4,EP300,EPHA3,ESR1,ETF1,F2,F3,FAP,FAS,FCGR2A,GAD2,GRK2,GTF2F2,HASPIN,HCK,HLA,DRB1,HNRNPA2B1,HSP90AB1,IFNG,IGF1R,IMPDH1,ITGB3,KLK1,KMT5A,LRBA,MAGEA4,MAGI2,MMP2,MSH2,NCOA3,NOTCH1,NT5C3A,NUP214,PA2G4,PDHA1,PDK3,PDK4,PGC,POT1,PRKCB,PTK2B,PTPRE,PYGL,RAC1,RARA,RARG,RRM1,SH2B2,SMG5,SMG7,SMTN,STK4,SYT13,TAF13,TJP1,TLR4,TXNDC12,UEVLD,VEGFB,WEE1,XIAP,ZEB2
260	Cell viability of breast cancer cell lines	Cell Death and Survival	9.98E,07	17	APEX1,BTRC,EP300,ERBB4,FN1,IGF1R,ITGB3,KIF11,NME3,NT5C3A,PDHA1,PTK2B,PTPRE,RRM1,RUVBL1,SEM1,SFN
261	Glycolysis of tumor cell lines	Carbohydrate Metabolism,Cellular Function and Maintenance	1.03E,06	8	ESR1,HK2,IGF1R,ITGB3,NCOA3,PDHA1,PDK4,SENP2
262	Cell death of colorectal cancer cell lines	Cell Death and Survival	1.03E,06	21	BTK,DAPK2,DFFA,DSG2,DTYMK,EP300,FAS,HMOX1,IFNG,MUC1,NF2,NOTCH1,PPARG,RAC1,SFN,SMURF2,SNX1,TLR4,WEE1,XIAP,YWHAH
263	High,risk neoplasia	Cancer,Organismal Injury and Abnormalities	1.03E,06	13	ALB,ALK,AR,BTK,CSF2RB,CYP17A1,ERBB4,ESR1,HSP90AB1,IMPDH1,RARA,RARG,RRM1
264	Adult T cell leukemia	Cancer,Hematological Disease,Immunological Disease,Organismal Injury and Abnormalities	1.04E,06	10	B2M,CD1A,FAS,HNRNPA2B1,IMPDH1,NOTCH1,POT1,PRKCB,RARA,RRM1
265	Non,Hodgkin cutaneous lymphoma	Cancer,Dermatological Diseases and Conditions,Hematological Disease,Immunological Disease,Organismal Injury and Abnormalities	1.07E,06	14	CTCF,DPP4,EPHA3,FAS,HSP90AB1,NOTCH1,ODC1,POT1,PRKCB,PTPRE,RARA,RARG,RRM1,TLR4
266	Fibrosis	Organismal Injury and Abnormalities	1.12E,06	26	ALB,AR,B2M,BMP1,FAS,FN1,GRHPR,HBEGF,HMGB3,HMGCS1,IFNG,IFNGR1,IMPDH1,ITGB3,MMP1,MYBPC1,NMT1,NOTCH1,PAH,PARN,PPARG,PTPRE,SERPINA5,SMURF2,STK4,TLR4

267	Cell movement of blood cells	Cellular Movement	1.18E,06	25	ALB,AOC3,ARHGEF1,CD74,DAPK2,DOK2,DPP4,F2,FAS,FCGR2A,FN1,HMOX1,IFNG,ITGB3,MMP1,MSN,PA2G4,PPARG,PRKCB,PTK2B,RAC1,SEMA4D,STK4,TLR4,YARS1
268	Lymphocytic leukemia	Cancer,Hematological Disease,Immunological Disease,Organismal Injury and Abnormalities	1.20E,06	48	ALB,ALK,ALOX12,ARHGAP5,B2M,BTK,C8A,CD1A,CD74,CDK5R1,CIAO1,CTCF,DPP4,EP300,F2,F3,FAS,FCGR2A,GAD2,GRK2,GTF2F2,HASPIN,HCK,HNRNPA2B1,HSP90AB1,IFNG,IMPDH1,ITGB3,LRBA,MAGI2,NCOA3,NOTCH1,PGC,POT1,PRKCB,PTK2B,PTPRE,PYGL,RARA,RRM1,SH2B2,SMG5,SYT13,TAF13,TLR4,TXNDC12,XIAP,ZEB2
269	Cleavage of RNA	RNA Post,Transcriptional Modification	1.24E,06	5	ANG,APEX1,HBB,IFNG,SENP2
270	Ruffling	Cell Morphology	1.26E,06	7	ARF6,FCGR2A,GRK2,HMOX1,IQUB,NF2,RAC1
271	Binding of breast cancer cell lines	Cell,To,Cell Signaling and Interaction	1.26E,06	10	ARHGEF1,ESR1,FN1,GRK6,IGF1R,ITGB3,MMP2,SERPINA5,SNX1,TJP1
272	Adhesion of mononuclear leukocytes	Cell,To,Cell Signaling and Interaction,Hematological System Development and Function,Immune Cell Trafficking	1.26E,06	10	AOC3,ARHGEF1,F2,FAS,FN1,IFNG,PPARG,PTK2B,RAC1,TLR4
273	Migration of tumor cell lines	Cellular Movement	1.36E,06	43	ALOX12,APPL1,AR,ARF6,ARHGEF1,DPP4,ESR1,F2,F3,FABP7,FHIT,FLNB,FN1,GRK6,HBEGF,HMOX1,HNRNPA2B1,IFNG,IGF1R,ITGB3,MAPK6,MATK,MMP1,MMP2,MSN,MUC1,NCOA3,NF2,NOTCH1,PTK2B,RAC1,RARG,RUVBL1,SEMA4D,SERPINA5,SFN,THBS2,TLR4,VAV3,VEGFB,XIAP,YY1,ZEB2
274	Liver lesion	Gastrointestinal Disease,Hepatic System Disease,Organismal Injury and Abnormalities	1.39E,06	152	ACACB,ACADSB,ACADVL,ACY1,ALB,ALK,ANG,APEX1,AR,ARHGAP5,ASAP1,ATP7B,ATXN3,B2M,B3GAT2,BCR,BTK,BTRC,C1R,C8A,CANT1,CD1A,CDK5R1,CLCN5,CLK1,CSDE1,CSF2RB,CSNK1G1,CTCF,CUL1,CYP2E1,CYP7A1,DGKA,ECHS1,ECI2,EP300,EPHA3,EPS8L2,ERBB4,ERI1,ESR1,EXOSC9,F2,FABP7,FAP,FAS,FCGR2A,FLNB,FN1,GALNT10,GC,GGPS1,GRHPR,GTF2F2,GTF2I,HADH,HBB,HCK,HDAC7,HDX,HIBCH,HK2,HMGB3,HMGCS1,HMOX1,HNRNPA2B1,HPD,HSP90AB1,HTR1A,IDE,IFNG,IFNGR1,IGF1R,IMPDH1,IQUB,KMT5A,KYNU,MAGI2,MAPK6,MARF1,MDH1,MECR,MMP2,MSH2,MSN,MUC1,MYBPC1,MYBPC3,NAE1,NBEA,NCBP1,NCOA3,NF2,NFATC1,NMT1,NOTCH1,NT5C3A,NUP214,PAH,PATJ,PC,PCYT2,PDHA1,PDK3,PDK4,PEX5,PGK1,PHF24,POT1,POU2F1,PPARG,PPP2R1A,PRKCB,PSAP,PTPRE,PTPRF,PYGL,RAC1,RARA,RBBP6,RRM1,SART3,SEC23A,SERPINA5,SFMBT2,SLK,SMG7,SMURF2,SNX22,STARD13,SULT1A1,THBS2,TJP1,TLR4,TNFAIP8L2,TRIM21,UEVLD,USP19,UTRN,VAV3,VEGFB,WEE1,WWP1,XRCC6,YWHAH,YY1,ZBTB43,ZCWPW1,ZEB2,ZIC3,ZNF292,ZNF462
275	Acne	Dermatological Diseases and Conditions,Inflammatory Disease,Organismal Injury and Abnormalities	1.51E,06	10	AR,DHPS,ESR1,G6PD,MMP1,MMP2,ODC1,RARA,RARG,TLR4
276	Lymphocyte migration	Cellular Movement,Hematological System Development and Function,Immune Cell Trafficking	1.51E,06	13	AOC3,ARHGEF1,CD74,DPP4,F2,FAS,FN1,IFNG,ITGB3,MSN,PTK2B,RAC1,TLR4
277	Stage IIIA,IV breast cancer	Cancer,Organismal Injury and Abnormalities,Reproductive System Disease	1.53E,06	8	ALB,AR,ERBB4,ESR1,GGPS1,HSP90AB1,PARP3,RRM1
278	Allergy of skin	Dermatological Diseases and Conditions,Immunologic	1.53E,06	8	AR,C1R,DPP4,ESR1,G6PD,IFNG,PRKCB,TLR4

		al Disease,Organismal Injury and Abnormalities			
279	Early stage tumor	Cancer,Organismal Injury and Abnormalities	1.56E,06	17	ALB,ALK,B2M,BTK,CD74,CSF2RB,ERBB4,ESR1,FN1,GART,GGPS1,HSP90AB1,MUC1,PGC,RARA,RARG,RRM1
280	Sickle cell anemia	Cardiovascular Disease,Connective Tissue Disorders,Hematological Disease,Hereditary Disorder,Organismal Injury and Abnormalities	1.65E,06	10	F3,GRHPR,HBB,HMOX1,IFNG,IMPDH1,KLK1,PTPRE,RRM1,TAF13
281	Leukemia	Cancer,Hematological Disease,Immunological Disease,Organismal Injury and Abnormalities	1.73E,06	79	ALB,ALK,ALOX12,AMY1C (includes others),APEX1,ARHGAP5,B2M,BCR,BTK,BTRC,C8A,CANT1,CD1A,CD74,CDK5R1,CIAO1,CSF2RB,CTCF,CYP2E1,CYP2R1,DPP4,EP300,EPHA3,ESR1,ETF1,F2,F3,FAP,FAS,FCGR2A,GAD2,GRK2,GTF2F2,HASPIN,HCK,HLA,DRB1,HNRNPA2B1,HSP90AB1,IFNG,IGF1R,IMPDH1,ITGB3,KLK1,KMT5A,LRBA,MAGEA4,MAGI2,MSH2,NCOA3,NOTCH1,NT5C3A,NUP214,PA2G4,PDHA1,PDK3,PGC,POT1,PRKCB,PTK2B,PTPRE,PYGL,RAC1,RARA,RARG,RRM1,SH2B2,SMG5,SMG7,SMTN,SYT13,TAF13,TJP1,TLR4,TXNDC12,UEVLD,VEGFB,WEE1,XIAP,ZEB2
282	Incorporation of thymidine	DNA Replication, Recombination, and Repair,Nucleic Acid Metabolism,Small Molecule Biochemistry	1.76E,06	7	ANG,AR,FAS,FN1,HBEGF,IGF1R,THBS2
283	Stage IIIB,IV recurrent non,small cell lung cancer	Cancer,Organismal Injury and Abnormalities,Respiratory Disease	1.76E,06	7	ALB,ALK,ERBB4,GART,HSP90AB1,IGF1R,RRM1
284	Hemolysis	Cell Death and Survival,Connective Tissue Disorders,Hematological Disease,Organismal Injury and Abnormalities	1.78E,06	11	ALB,C8A,DPP4,FAS,FCGR2A,G6PD,HBB,HMOX1,IMPDH1,ITPA,NT5C3A
285	Accumulation of lipid	Lipid Metabolism,Molecular Transport,Small Molecule Biochemistry	1.80E,06	10	ARF6,ECHS1,FABP7,FAS,HDAC7,HMOX1,NCOA3,PAFAH1B2,PPARG,PSAP
286	Ovarian adenocarcinoma	Cancer,Endocrine System Disorders,Organismal Injury and Abnormalities,Reproductive System Disease	1.82E,06	60	ALB,ALK,AR,ARF6,ARHGEF1,ASAP1,B2M,BCR,CA8,CLK1,CSF2RB,CSNK1G1,CSTF2,CTCF,DPP4,EP300,EPHA3,F2,GART,HBEGF,HCK,HNRNPA2B1,HSP90AB1,HTR1A,ITPA,ITSN1,MAGEA4,MAGI2,MAP3K3,MUC1,NBEA,NCOA3,NF2,NOTCH1,NR1I3,NT5C3A,ODC1,PARP3,PC,PDK4,PGC,POU2F1,PPARG,PPP2R1A,PTPRF,PYGL,RBM39,RRM1,RTN4IP1,SFMBT2,SH3YL1,SKP1,SMTN,SYT13,THBS2,TLR4,UTRN,VAV3,WWP1,ZEB2
287	Colony formation	Cellular Growth and Proliferation	1.86E,06	27	ALK,AR,BTK,CSF2RB,CTCF,ERBB4,ESR1,FAS,HCK,HNRNPA2B1,IFNG,IGF1R,MSH2,MUC1,NCOA3,NOTCH1,PDHA1,PDK4,PPARG,PPP2R1A,PRKCB,RARG,SEMA4D,SFN,STK4,VEGFB,XIAP
288	Cell cycle progression	Cell Cycle	1.88E,06	35	ALK,AR,BTRC,CAMK4,CDK5R1,EP300,ESR1,F2,FAS,GRK2,HASPIN,HBEGF,HMOX1,IFNG,IGF1R,KIF11,MATK,NAE1,NDC80,NF2,NOTCH1,PPARG,PPP2R1A,PRKCB,RAC1,RARA,RBBP6,RRM1,RUVBL1,SFN,TLR4,TRIM21,WEE1,XIAP,XRCC6

289	Hereditary connective tissue disorder	Connective Tissue Disorders,Hereditary Disorder,Organismal Injury and Abnormalities	1.89E,06	34	ADH1C,ALB,B3GAT3,BMP1,C1R,CANT1,CLCN5,CYP2R1,EP300,ESR1,FLNB,FN1,GC,GGPS1,HBB,HNRNPA2B1,IFNGR1,IMPDH1,KIF11,KYNU,MMP1,MMP2,MTAP,MUC1,MYBPC1,NOTCH1,PEX5,PPARG,PTPRF,PUS10,RARG,RRM1,THBS2,ZNF462
290	Cell movement of leukemia cell lines	Cellular Movement	1.92E,06	13	ARHGEF1,F2,FLNB,FN1,GC,MATK,NOTCH1,PPARG,PRKCB,PTK2B,RAC1,WARS1,YARS1
291	Fatty acid metabolism	Lipid Metabolism,Small Molecule Biochemistry	1.97E,06	22	ACACB,ACADSB,ALB,ALOX12,CD74,CERT1,CHKA,CYP2E1,ECHS1,F2,FABP3,FAS,FCGR2A,FN1,G6PD,IFNG,ITGB3,MECR,MSN,PDK4,PPARG,PSAP
292	Formation of colorectal cancer cell lines	Cellular Development,Cellular Growth and Proliferation	1.98E,06	8	FN1,IFNG,NOTCH1,PDK4,PPP2R1A,RARG,VEGFB,XIAP
293	Cell proliferation of carcinoma cell lines	Cellular Development,Cellular Growth and Proliferation	2.01E,06	29	AASDHPPT,ALK,AR,CDK5R1,CSTF2,DAPK2,ECHS1,EP300,F3,FAS,FHIT,HK2,HMOX1,HNRNPA2B1,IFNG,IGF1R,MMP1,MUC1,NCOA3,NOTCH1,PDK4,PPARG,PRKCB,PTK2B,PTPRF,RAC1,S100A6,STK4,YY1
294	Apoptosis of lymphoma cell lines	Cell Death and Survival	2.04E,06	13	ALB,ALK,BTK,FAS,FN1,HCK,IFNG,IGF1R,MUC1,NOTCH1,PPARG,RAC1,XIAP
295	Metastatic cervical neoplasm	Cancer,Organismal Injury and Abnormalities,Reproductive System Disease	2.07E,06	7	ALK,AOC3,F3,GAD2,GART,HSP90AB1,RRM1
296	Cholangiocarcinoma	Cancer,Gastrointestinal Disease,Hepatic System Disease,Organismal Injury and Abnormalities	2.07E,06	11	ACY1,ADH1C,ALB,ALK,FAP,GC,HPD,HSP90AB1,MMP2,PGK1,RRM1
297	Recurrent pelvic tumor	Cancer,Organismal Injury and Abnormalities	2.22E,06	9	ALK,AR,CYP17A1,ESR1,F3,GART,HSP90AB1,PARP3,RRM1
298	Ovarian carcinoma	Cancer,Endocrine System Disorders,Organismal Injury and Abnormalities,Reproductive System Disease	2.22E,06	62	ALB,ALK,AR,ARF6,ARHGEF1,ASAP1,B2M,BCR,CA8,CLK1,CSF2RB,CSNK1G1,CSTF2,CTCF,DPP4,EP300,EPHA3,ESR1,F2,GART,HBEGF,HCK,HNRNPA2B1,HSP90AB1,HTR1A,IFNGR1,ITPA,ITSN1,MAGEA4,MAGI2,MAP3K3,MUC1,NBEA,NCOA3,NF2,NOTCH1,NR1I3,NT5C3A,ODC1,PARP3,PC,PDK4,PGC,POU2F1,PPARG,PPP2R1A,PTPRF,PYGL,RBM39,RRM1,RTN4IP1,SFMBT2,SH3YL1,SKP1,SMTN,SYT13,THBS2,TLR4,UTRN,VAV3,WWP1,ZEB2
299	Autophosphorylation of protein	Post,Translational Modification	2.23E,06	11	ALK,DAPK2,ERBB4,HCK,IGF1R,MAP3K3,OXSR1,PTK2B,SLK,STK16,STK4
300	Cell death of epithelial cell lines	Cell Death and Survival,Organismal Injury and Abnormalities	2.41E,06	18	AR,ATXN3,F2,FAS,GRK2,HK2,IFNG,IGF1R,NDUFAB1,NF2,PEX5,PPARG,PRKCB,STK4,TNFAIP8L2,XIAP,YWHAQ,YY1
301	Stage IV non,squamous lung carcinoma	Cancer,Organismal Injury and Abnormalities,Respiratory Disease	2.45E,06	9	ALB,ALK,ERBB4,GART,HSP90AB1,IGF1R,PARP3,PTK2B,RRM1
302	Cell death of lymphoma cell lines	Cell Death and Survival	2.45E,06	14	ALB,ALK,BTK,FAS,FN1,HCK,IFNG,IGF1R,MUC1,NOTCH1,PPARG,PRKCB,RAC1,XIAP
303	Stage I,IV non,small cell lung cancer	Cancer,Organismal Injury and Abnormalities,Respiratory Disease	2.47E,06	12	ALB,ALK,B2M,ERBB4,FN1,GART,HSP90AB1,IGF1R,PARP3,PGC,PTK2B,RRM1
304	Nonsquamous non,small cell lung carcinoma	Cancer,Organismal Injury and	2.55E,06	72	ADH1C,ADH7,ALB,ALK,ALOX12,AR,B2M,BTK,CD1A,CERT1,CSNK1G2,CTCF,DGKA,DOK2,EP300,EPHA3,ERBB4,ESR1,F3,FN1,GAD2,GART,GRK6,HASPIN,HCK,HMGB3,HPD,HSD17B4,HSP90AB1,HTR1A,IGF1R,IT

		Abnormalities,Respiratory Disease			PKC,KIF11,KYNU,LRBA,MAGEA4,MAGI2,MAPK6,MATK,MMP1,MSH2,MSN,MUC1,NBEA,NDC80,NMT1,NOTCH1,NT5C3A,OAT,OXSR1,PAH,PARP3,PATJ,PDK4,PIP4K2C,PPP2R1A,PRKCB,PTK2B,PTPRE,PYGL,RARA,RRM1,SEMA4D,SERPINA5,SFN,SLK,SMG7,STK4,TLR4,TTR,ZCWPW1,ZNF292
305	Breast or colorectal cancer	Cancer,Organismal Injury and Abnormalities	2.56E,06	179	ACACB,ACADVL,ADH7,ALB,ALK,ANG,AOC3,APEX1,APPL1,AR,ARHGAP5,ARHGEF1,B2M,BCR,BMP1,BPI,BTK,C1R,CAMK4,CANT1,CDK5R1,CERT1,CLK1,CSDE1,CSF2RB,CSNK1G1,CSNK1G2,CSTF2,CTCF,CUL1,CYP17A1,CYP2E1,CYP7A1,DAPK2,DCXR,DFFA,DGKA,DHPS,DOK2,DPP4,DSG2,ECHS1,EP300,EPHA3,ERBB4,ESR1,EXOSC9,F2,F3,FABP7,FAP,FAS,FCGR2A,FHIT,FLNB,FN1,G6PD,GAD2,GALNT10,GART,GGPS1,GRK2,GRK6,HASPIN,HAT1,HBB,HBEGF,HCK,HDAC7,HIBCH,HLA,DRB1,HMGB3,HMGCS1,HMOX1,HNRNPA2B1,HSP90AB1,HTR1A,IDE,IFNG,IFNGR1,IGF1R,IMPDH1,IQUB,ITGB3,ITPKC,ITSN1,KIF11,KMT5A,LRBA,MAGEA4,MAGI2,MAP3K3,MAPK6,MARF1,MARK1,MATK,MMP1,MMP2,MSH2,MSN,MTAP,MUC1,MYBPC3,NAE1,NBEA,NCOA3,NDC80,NF2,NFATC1,NMT1,NOTCH1,NT5C3A,NUP214,ODC1,OXSR1,PAFAH1B2,PANK1,PAPSS1,PARP3,PATJ,PC,PCYT2,PDK3,PDK4,PEX5,PGK1,PHF24,POU2F1,PPARG,PPP2R1A,PRKCB,PTK2B,PTPRE,PTPRF,PUS10,PYGL,RAC1,RARA,RARG,RBBP6,RBM39,RND1,RRM1,RUVBL1,S100A6,SART3,SEMA4D,SENP2,SERPINA5,SFMBT2,SLC5A1,SLK,SMAP1,SMG5,SMG7,SMTN,SMURF2,SNX1,SPEF1,STARD13,STK4,SYT13,THBS2,TLR4,TTR,UEVLD,USP19,UTRN,VAV3,VEGFB,WEE1,WHRN,XRCC6,YWHAQ,YY1,ZEB2,ZFP28,ZNF292,ZNF462
306	Benign lesion	Organismal Injury and Abnormalities	2.57E,06	38	ALK,AR,BCR,CLCN5,CSF2RB,CYP17A1,EP300,ERBB4,ESR1,F2,FABP3,FAS,FN1,GGPS1,HDAC7,HSD17B10,HSP90AB1,IGF1R,IMPDH1,MDH1,MMP1,MMP2,MSH2,MSN,MUC1,NOTCH1,ODC1,PDK4,PPARG,PRKCB,RARA,RRM1,RUVBL1,S100A6,TAF13,UTRN,WEE1,ZEB2
307	Metastatic malignant neoplasm of prostate	Cancer,Organismal Injury and Abnormalities,Reproductive System Disease	2.58E,06	11	AR,CYP17A1,ESR1,GGPS1,HSP90AB1,MMP2,MSH2,PARP3,PPP2R1A,RARG,RRM1
308	Colony formation of cells	Cellular Development,Cellular Growth and Proliferation	2.62E,06	25	ALK,AR,CTCF,ERBB4,ESR1,FAS,HCK,HNRNPA2B1,IFNG,IGF1R,MSH2,MUC1,NCOA3,NOTCH1,PDHA1,PDK4,PPARG,PPP2R1A,PRKCB,RARG,SEMA4D,SFN,STK4,VEGFB,XIAP
309	Stage IV advanced non,small cell lung carcinoma	Cancer,Organismal Injury and Abnormalities,Respiratory Disease	2.70E,06	9	ALB,ALK,ERBB4,GART,HSP90AB1,IGF1R,PARP3,PTK2B,RRM1
310	HER2 negative progesterone receptor negative breast cancer	Cancer,Organismal Injury and Abnormalities,Reproductive System Disease	2.72E,06	14	AR,CERT1,ERBB4,ESR1,FAS,HCK,HSP90AB1,MAGEA4,MMP2,MUC1,PARP3,PPARG,RRM1,WEE1
311	Cell viability of lung cancer cell lines	Cell Death and Survival	2.73E,06	13	ALK,FHIT,FN1,HBEGF,HCK,HK2,HMOX1,IGF1R,KIF11,MSH2,NOTCH1,PTK2B,RRM1
312	Repair of DNA	DNA Replication, Recombination, and Repair	2.79E,06	20	APEX1,ATXN3,BTRC,EP300,G6PD,HAT1,HMOX1,IFNG,IGF1R,MSH2,MYSM1,NAE1,PARP3,RAC1,RARA,RPA3,RRM1,USP19,WEE1,XRCC6
313	Remodeling of actin cytoskeleton	Cellular Assembly and Organization	2.80E,06	6	ARHGEF1,F2,MSN,PTK2B,RAC1,TLR4
314	Stage IIIB,IV nonsquamous non,small cell lung carcinoma	Cancer,Organismal Injury and Abnormalities,Respiratory Disease	2.85E,06	8	ALB,ALK,ERBB4,GART,HSP90AB1,IGF1R,PARP3,RRM1
315	Astrocytoma	Cancer,Neurological Disease,Organismal Injury and Abnormalities	2.86E,06	146	ACADVL,ACVR2B,ADH7,ALB,ALK,ALOX12,AMY1C (includes others),APEX1,APPL1,ARHGAP5,ARHGEF1,ASAP1,ATP7B,ATXN3,B3GAT2,BMP1,BPI,C1R,C8A,CLCN5,CSDE1,CSF2RB,CSNK1G2,CSTF2,CTCF,CUL1,CYP2E1,DGKA,DOK2,DPP4,DSG2,EP300,EPHA3,EPS8L2,ERBB4,ESR1,F2,FABP7,FAS,FLNB,FN1,G6PD,GAD2,GALNT10,GART,GRK2,GRK6,GTF2I,HADH,HASPIN,HDX,HIBC

					H,HLA,DRB1,HMBOX1,HMGCS1,HMOX1,HNRNPA2B1,HSD17B10,HSP90AB1,IDE,IFNG,IFNGR1,IGF1R,ITGB3,ITSN1,KMT5A,KYNU,LRBA,MAGI2,MAP3K3,MARF1,MATK,MSH2,MSN,MTAP,MUC1,MYBPC3,NBEA,NCBP1,NCOA3,NF2,NFATC1,NMT1,NOTCH1,NSUN5,NUP214,ODC1,OXSR1,PA2G4,PAFAH1B2,PAH,PANK1,PAPSS1,PARN,PATJ,PC,PCYT2,PDHA1,PGK1,POU2F1,PPARG,PPP2R1A,PRKCB,PSAP,PTK2B,PUS10,PYGL,RAC1,RARA,RARG,RBBP6,RBP5,RRM1,RTN4IP1,SART3,SEMA4D,SENP2,SERPINA5,SFMBT2,SH2B2,SH3YL1,SLC5A1,SMURF2,SNX1,STARD13,SUB1,TAF13,THBS2,TJP1,TLR4,TNFAIP8L2,USP19,UTRN,VAV3,VPS26A,WEE1,WHRN,YWHAH,YWHAQ,ZBTB43,ZCWPW1,ZEB2,ZFP28,ZIC3,ZNF292,ZNF462
316	Cell movement of leukocytes	Cellular Movement,Hematological System Development and Function,Immune Cell Trafficking	2.99E,06	20	ALB,AOC3,ARHGEF1,CD74,DAPK2,DPP4,F2,FAS,FN1,IFNG,ITGB3,MSN,PPARG,PRKCB,PTK2B,RAC1,SEMA4D,STK4,TLR4,YARS1
317	Anoikis	Cell Death and Survival	2.99E,06	10	DAPK2,FAS,FN1,IGF1R,ITGB3,NCOA3,NOTCH1,PDK4,SNX1,XIAP
318	Neoplasia of lymphoid organ	Cancer,Immunological Disease,Organismal Injury and Abnormalities	3.02E,06	14	BTK,EP300,ERBB4,ESR1,GART,GTF2I,HSP90AB1,IGF1R,MMP2,NOTCH1,NT5C3A,RRM1,TXNDC12,XIAP
319	Leukocyte migration	Cellular Movement,Immune Cell Trafficking	3.12E,06	24	ALB,AOC3,ARHGEF1,CD74,DAPK2,DOK2,DPP4,F2,FAS,FN1,HMOX1,IFNG,ITGB3,MMP1,MSN,PA2G4,PPARG,PRKCB,PTK2B,RAC1,SEMA4D,STK4,TLR4,YARS1
320	Psoriasis	Dermatological Diseases and Conditions,Organismal Injury and Abnormalities	3.33E,06	28	ACP3,ATXN3,CDK5R1,CYP2E1,DPP4,ETF1,FABP7,FCGR2A,FN1,HBB,HMOX1,IFNG,IFNGR1,KLK1,KYNU,MAPK6,MMP1,MMP2,ODC1,PPARG,PRKCB,PSAP,RARA,RARG,SFN,SUB1,TLR4,YWHAQ
321	Glioma	Cancer,Neurological Disease,Organismal Injury and Abnormalities	3.33E,06	181	ACACB,ACADSB,ACADVL,ACVR2B,ADH7,ALB,ALK,ALOX12,AMY1C (includes others),AOC3,APEX1,APPL1,ARHGAP5,ARHGEF1,ASAP1,ATP7B,ATXN3,B3GAT2,BCR,BMP1,BPI,C1R,C8A,CLCN5,CRYM,CSDE1,CSF2RB,CSNK1G1,CSNK1G2,CSTF2,CTCF,CUL1,CYP2E1,DAPK2,DCXR,DGKA,DOK2,DPP4,DSG2,EP300,EPHA3,EPS8L2,ERBB4,ESR1,F2,FABP7,FAS,FCGR2A,FLNB,FN1,G6PD,GAD2,GALNT10,GART,GRK2,GRK6,GTF2I,HADH,HASPIN,HAT1,HBEGF,HDX,HIBCH,HK2,HLA,DRB1,HMBOX1,HMGB3,HMGCS1,HMOX1,HNRNPA2B1,HSD17B10,HSD17B4,HSP90AB1,IDE,IFNG,IFNGR1,IGF1R,ITGB3,ITPK1,ITSN1,KMT5A,KYNU,LRBA,MAGEA4,MAGI2,MAP3K3,MARF1,MATK,MMP2,MSH2,MSN,MTAP,MUC1,MYBPC1,MYBPC3,NBEA,NCBP1,NCOA3,NF2,NFATC1,NME3,NMT1,NOTCH1,NR1I3,NSUN5,NUP214,ODC1,OXSR1,PA2G4,PAFAH1B2,PAH,PANK1,PAPSS1,PARN,PATJ,PC,PCYT2,PDHA1,PDK4,PGC,PGK1,PHF24,PIP4K2C,POT1,POU2F1,PPARG,PPP2R1A,PRKCB,PSAP,PTK2B,PTPRE,PTPRF,PUS10,PYGL,RAC1,RARA,RARG,RBBP6,RBM39,RBP5,RGS6,RRM1,RTN4IP1,SART3,SEMA4D,SENP2,SERPINA5,SFMBT2,SH2B2,SH3YL1,SLC5A1,SLK,SMG5,SMURF2,SNX1,STARD13,SUB1,TAF13,THBS2,TIMM9,TJP1,TLR4,TNFAIP8L2,USP19,UTRN,VAV3,VEGFB,VPS26A,WEE1,WHRN,XIAP,XRCC6,YWHAH,YWHAQ,ZBTB43,ZCWPW1,ZEB2,ZFP28,ZIC3,ZNF292,ZNF462
322	Familial encephalopathy	Hereditary Disorder,Neurological Disease,Organismal Injury and Abnormalities	3.34E,06	58	ADH1C,ALB,ANG,ARF6,ARHGEF1,ATP7B,ATXN3,CA8,CDK5R1,CERT1,CRYM,CTCF,CYP7A1,ECHS1,EP300,ERBB4,ESR1,EXOSC9,F3,FAS,FCGR2A,G6PD,HADH,HMBOX1,HMOX1,HNRNPA2B1,HSD17B10,HTR1A,IFNG,IFNGR1,ITPA,MECR,MMP1,MSH2,NBEA,NDUFAB1,NF2,NR1I3,NUP214,PCYT2,PDHA1,PGK1,PPARG,PPP2R1A,PRKCB,PSAP,PTK2B,PTPRE,RAC1,RTN4IP1,SMTN,SUB1,TAF13,XIAP,XRCC6,ZEB2,ZIC3,ZNF292
323	Proliferation of prostate cancer cell lines	Cellular Development,Cellular Growth and Proliferation	3.39E,06	21	ACP3,AR,CD74,EP300,F2,HBEGF,HMOX1,IFNG,IGF1R,MMP2,NCOA3,NOTCH1,PA2G4,PDK4,PPARG,PPP2R1A,RAC1,RARG,STK4,VAV3,YWHAQ
324	Adhesion of peripheral blood leukocytes	Cell,To,Cell Signaling and Interaction,Hematological System Development	3.43E,06	6	F2,F3,IFNG,PPARG,PTK2B,RAC1

		and Function,Immune Cell Trafficking			
325	Progressive recurrent neoplasm	Cancer,Organismal Injury and Abnormalities,Tumor Morphology	3.52E,06	10	ALK,ERBB4,ESR1,F3,GART,HSP90AB1,IGF1R,PARP3,PTK2B,RRM1
326	Squamous,cell carcinoma	Cancer,Organismal Injury and Abnormalities	3.58E,06	118	ALB,ALK,ANXA6,AOC3,AR,ARF6,ARHGAP5,ARHGEF1,ASAP1,ATXN3,B2M,BCR,BPI,BTK,BTRC,C8A,CAMK4,CD74,CERT1,CLK1,CSF2RB,CSNK1G1,CTCF,CUL1,CYP2E1,CYP7A1,DGKA,DOK2,DPP4,DSG2,EP300,EPHA3,EPS8L2,ERBB4,F3,FAP,FAS,FCGR2A,FHIT,FN1,GALM,GART,GRHPR,HASPIN,HBB,HBEGF,HDX,HLA,DRB1,HNRNPA2B1,HSCB,HSP90AB1,HTR1A,IDE,IFNGR1,IGF1R,IMPDH1,IQUB,ITPKC,KLK1,KMT5A,MAGI2,MAPK6,MATK,MDH1,MECR,MSH2,MSN,MTAP,MUC1,MYBPC1,NCOA3,NDUFAB1,NF2,NFATC1,NOTCH1,NT5C3A,NUP214,OAT,OXSR1,PANK1,PARP3,PATJ,PDK2,PDK4,PGC,PGK1,POT1,POU2F1,PPARG,PPP2R1A,PSAP,PTK2B,PTPRE,PTPRF,RAC1,RARG,RBBP6,RGS6,RRM1,SEMA4D,SENP2,SERPINA5,SFMBT2,SH2B2,SLK,SMG7,SMTN,STK4,TJP1,TLR4,TTR,VAV3,WWP1,ZEB2,ZFP28,ZIC3,ZNF292,ZNF462
327	Metastasis of primitive neuroectodermal tumor	Cancer,Neurological Disease,Organismal Injury and Abnormalities	3.79E,06	7	ALK,CSF2RB,ERBB4,FCGR2A,HSP90AB1,RARA,RARG
328	Primary carcinoma	Cancer,Organismal Injury and Abnormalities	4.04E,06	13	CDK5R1,DOK2,ERBB4,ESR1,HSP90AB1,IGF1R,MUC1,NF2,PARP3,PPARG,RAC1,RRM1,VEGFB
329	Hemoglobinopathy	Connective Tissue Disorders,Hematological Disease,Organismal Injury and Abnormalities	4.22E,06	11	F3,GGPS1,GRHPR,HBB,HMOX1,IFNG,IMPDH1,KLK1,PTPRE,RRM1,TAF13
330	Vasculogenesis	Cardiovascular System Development and Function,Organismal Development	4.25E,06	24	ANG,BPI,DGKA,DSG2,F2,F3,FN1,HBEGF,HMOX1,IFNG,ITGB3,KLK1,MMP2,NFATC1,NOTCH1,PATJ,PPARG,RAC1,STARD13,THBS2,TLR4,VEGFB,YARS1,YY1
331	Stage III epithelial cancer	Cancer,Organismal Injury and Abnormalities	4.46E,06	10	ALB,ALK,ERBB4,ESR1,GART,HSP90AB1,IGF1R,MUC1,PARP3,RRM1
332	Pancreatobiliary tumor	Cancer,Gastrointestinal Disease,Organismal Injury and Abnormalities	4.47E,06	99	ACACB,ACAT2,ACY1,ADH1C,ALB,ALK,ALOX12,APPL1,AR,ARHGAP5,ATP7B,BCR,BPI,BTRC,CD74,CDK5R1,CHKA,CLK1,CRYM,CSDE1,CSF2RB,CSTF2,CTCF,DCTD,DFFA,DOK2,DPP4,DSG2,EP300,EPHA3,EPS8L2,ERBB4,ESR1,F2,F3,FAP,FLNB,FN1,GART,GC,GRHPR,GTF2I,HBEGF,HCK,HLA,DRB1,HMGCS1,HPD,HSP90AB1,IFNGR1,IGF1R,ITSN1,KIF11,LRBA,MAGI2,MARK1,MMP2,MSH2,MUC1,MYBPC1,MYBPC3,NAE1,NBEA,NF2,NFATC1,NOTCH1,NUP214,PA2G4,PARP3,PC,PDHA1,PDK4,PGC,PGK1,POT1,POU2F1,PPP2R1A,PRKCB,RARA,RBBP6,RBM39,RRM1,S100A6,SEC23A,SERPINA5,SFN,SH2B2,SMURF2,TJP1,TLR4,TRIM21,UEVLD,UTRN,VAV3,VEGFB,WEE1,YY1,ZEB2,ZFP28,ZNF292
333	Stage IV metastatic non,small cell lung carcinoma	Cancer,Organismal Injury and Abnormalities,Respiratory Disease	4.50E,06	8	ALK,ERBB4,GART,HSP90AB1,IGF1R,PARP3,PTK2B,RRM1
334	Non,insulin,dependent diabetes mellitus	Endocrine System Disorders,Gastrointestinal Disease,Metabolic Disease,Organismal Injury and Abnormalities	4.51E,06	21	ALB,AR,BPI,CYP2E1,DPP4,ECHS1,ESR1,GGPS1,HK2,HMBOX1,HTR1A,IDE,IGF1R,IMPDH1,KIF11,PPARG,RAC1,SLC5A1,TAF13,THBS2,VEGFB
335	Invasion of breast cancer cell lines	Cellular Movement	4.62E,06	20	ARF6,ASAP1,DGKA,ESR1,F2,FAS,FN1,HSP90AB1,ITGB3,MATK,MMP1,MMP2,MUC1,PTK2B,RAC1,RUVBL1,SMURF2,TJP1,VPS26A,ZEB2
336	Diabetic complication	Endocrine System Disorders,Gastrointestin	4.62E,06	20	AR,B2M,DPP4,ESR1,F3,FAS,FN1,HBB,IGF1R,IMPDH1,ITGB3,MAGI2,NOTCH1,PDK4,PPARG,PRKCB,SMURF2,UTRN,VEGFB,ZEB2

		al Disease,Metabolic Disease,Organismal Injury and Abnormalities			
337	Entry into interphase	Cell Cycle	4.75E,06	9	EP300,F2,FN1,HBEGF,HMOX1,IGF1R,NCOA3,TRIM21,WEE1
338	Connective tissue tumor	Cancer,Organismal Injury and Abnormalities	4.81E,06	52	ACACB,ALB,ALK,ANG,AR,CSF2RB,CYP7A1,DHPS,ERBB4,ESR1,F2,FABP7,FAP,FN1,GART,GGPS1,HMGCS1,HSD17B4,HSP90AB1,IFNGR1,IGF1R,IMPDH1,KMT5A,MAPK6,MMP1,MMP2,MSH2,NAE1,NBEA,NCBP1,NF2,NOTCH1,PARP3,PDK4,PEX5,PGK1,PHF24,PPARG,PPP2R1A,PRKCB,RARA,RARG,RRM1,SEC23A,SEMA4D,SLK,TJP1,TLR4,UTRN,WWP1,XIAP,ZIC3
339	Adult T,cell non,Hodgkin lymphoma	Cancer,Hematological Disease,Immunological Disease,Organismal Injury and Abnormalities	4.83E,06	11	B2M,DPP4,EP300,FAS,FHIT,HNRNPA2B1,IMPDH1,NOTCH1,POT1,PRKCB,RRM1
340	Liver tumor	Cancer,Gastrointestinal Disease,Hepatic System Disease,Organismal Injury and Abnormalities	4.88E,06	147	ACACB,ACADSB,ACADVL,ACY1,ALB,ALK,ANG,APEX1,AR,ARHGAP5,ASAP1,ATP7B,ATXN3,B2M,B3GAT2,BCR,BTK,BTRC,C1R,C8A,CANT1,CD1A,CDK5R1,CLCN5,CLK1,CSDE1,CSF2RB,CSNK1G1,CTCF,CUL1,CYP7A1,DGKA,ECHS1,ECI2,EP300,EPHA3,EPS8L2,ERBB4,ERI1,ESR1,EXOSC9,F2,FABP7,FAP,FLNB,FN1,GALNT10,GC,GGPS1,GRHPR,GTF2F2,GTF2I,HADH,HBB,HCK,HDAC7,HDX,HIBCH,HK2,HMGB3,HMGCS1,HMOX1,HNRNPA2B1,HPD,HSP90AB1,HTR1A,IDE,IFNG,IFNGR1,IGF1R,IMPDH1,IQUB,KMT5A,KYNU,MAGI2,MAPK6,MARF1,MDH1,MECR,MMP2,MSH2,MSN,MUC1,MYBPC1,MYBPC3,NAE1,NBEA,NCBP1,NCOA3,NF2,NFATC1,NMT1,NOTCH1,NT5C3A,NUP214,PAH,PATJ,PC,PCYT2,PDHA1,PDK3,PDK4,PEX5,PGK1,PHF24,POT1,POU2F1,PPARG,PPP2R1A,PRKCB,PSAP,PTPRE,PTPRF,PYGL,RAC1,RARA,RBBP6,RRM1,SART3,SEC23A,SFMBT2,SLK,SMG7,SMURF2,SNX22,STARD13,SULT1A1,THBS2,TJP1,TNFAIP8L2,TRIM21,UEVLD,USP19,UTRN,VAV3,VEGFB,WEE1,WWP1,XRCC6,YWHAH,YY1,ZBTB43,ZCWPW1,ZEB2,ZIC3,ZNF292,ZNF462
341	Uterine cancer	Cancer,Organismal Injury and Abnormalities,Reproductive System Disease	5.07E,06	139	AASDHPPT,ACACB,ACADSB,ACBD7,ACY1,ALB,ALK,AOC3,APPL1,AR,ATP7B,B2M,B3GAT2,BCR,BTK,BTRC,C8A,CA8,CD1A,CLCN5,CSDE1,CSF2RB,CSTF2,CTCF,CUL1,CYP7A1,DCTD,DHPS,DMC1,DOK2,DPP4,DTYMK,EP300,EPHA3,ERBB4,ESR1,ETF1,F3,FABP7,FAP,FAS,FCGR2A,FLNB,FN1,G6PD,GAD2,GART,GRHPR,GRK2,GTF2I,HBB,HDX,HMBOX1,HMGB3,HMOX1,HPD,HSD17B4,HSP90AB1,HTR1A,IGF1R,IQUB,ITGB3,ITPKC,KIF11,LRBA,MAGEA4,MAGI2,MAP3K3,MAPK6,MARF1,MARK1,MDH1,MEMO1,MMP1,MMP2,MSH2,MSN,MUC1,MYBPC3,MYSM1,NAE1,NBEA,NCBP1,NF2,NFATC1,NMRK1,NMT1,NOTCH1,NT5C3A,NUP214,OAT,PANK1,PARN,PARP3,PATJ,PDK3,PGK1,PPP2R1A,PTPRE,PTPRF,RAC1,RARA,RARG,RBBP6,RBM39,RGS6,RND1,RRM1,RUVBL1,SART3,SENP2,SERPINA5,SF3B6,SH3YL1,SLC5A1,SLK,SMG5,SMTN,SMURF2,SNX1,STK4,SUB1,SULT1A1,SYT13,THBS2,TJP1,TLR4,TRIM21,UEVLD,UTRN,VAV3,WWP1,XIAP,YY1,ZEB2,ZFP28,ZIC3,ZNF292,ZNF462
342	Inflammation of joint	Connective Tissue Disorders,Inflammatory Disease,Inflammatory Response,Organismal Injury and Abnormalities,Skeletal and Muscular Disorders	5.08E,06	39	ALB,ALOX12,ANG,AR,ASAP1,B2M,CD1A,CD74,CSF2RB,DPP4,FAS,FCGR2A,FN1,GGPS1,HBB,HCK,HDAC7,HLA,DRB1,HMOX1,IDE,IFNG,IMPDH1,MAP3K3,MMP1,MMP2,MTAP,NFATC1,PGK1,PPARG,PRKCB,PTK2B,PTPRE,RGS6,SF3B6,SMG7,TAF13,TJP1,TLR4,XIAP
343	Apoptosis of carcinoma cell lines	Cell Death and Survival	5.44E,06	20	ALK,AR,DAPK2,EP300,FAS,FHIT,FN1,G6PD,HK2,HMOX1,IFNG,IGF1R,MSH2,NCOA3,NOTCH1,PPARG,RAC1,RRM1,TLR4,XIAP
344	Cutaneous T,cell lymphoma	Cancer,Dermatological Diseases and Conditions,Hematological Disease,Immunological Disease,Organismal Injury and Abnormalities	5.50E,06	12	CTCF,DPP4,EPHA3,FAS,NOTCH1,ODC1,POT1,PRKCB,RARA,RARG,RRM1,TLR4

345	Stage IIIB cancer	Cancer,Organismal Injury and Abnormalities	5.61E,06	10	ALB,ALK,ERBB4,ESR1,FN1,GART,GGPS1,HSP90AB1,IGF1R,RRM1
346	Bladder carcinoma	Cancer,Organismal Injury and Abnormalities,Renal and Urological Disease	5.84E,06	51	ACACB,ALB,ALK,AOC3,AR,ARHGAP5,BCR,BMP1,BTK,CSTF2,CTCF,CUL1,DGKA,DHPS,DOK2,EP300,EPHA3,EPS8L2,ERBB4,GRHPR,HNRNPA2B1,HSP90AB1,KMT5A,KYNU,MAGEA4,MAGI2,MARF1,MMP2,MTAP,NCOA3,NF2,NOTCH1,NT5C3A,PA2G4,PAH,PDK4,PPARG,PPP2R1A,PRKCB,PTK2B,PTPRF,RARA,RARG,RBBP6,RBM39,RRM1,SLC5A1,TLR4,XIAP,ZCWPW1,ZEB2
347	Undifferentiated malignant neoplasm	Cancer,Organismal Injury and Abnormalities	6.01E,06	26	ALK,AMY1C (includes others),AR,DSG2,EPHA3,ERBB4,FN1,GALNT10,GART,GRK2,HASPIN,HSP90AB1,IGF1R,IMPDH1,MMP1,MSH2,PARP3,PDK4,PGK1,PTK2B,RRM1,SLK,STK4,TLR4,YY1,ZEB2
348	High grade astrocytoma	Cancer,Neurological Disease,Organismal Injury and Abnormalities	6.02E,06	144	ACADVL,ACVR2B,ADH7,ALB,ALK,ALOX12,AMY1C (includes others),APEX1,APPL1,ARHGAP5,ARHGEF1,ASAP1,ATP7B,ATXN3,B3GAT2,BMP1,BPI,C1R,C8A,CLCN5,CSDE1,CSF2RB,CSNK1G2,CSTF2,CTCF,CUL1,CYP2E1,DGKA,DOK2,DPP4,DSG2,EP300,EPHA3,EPS8L2,ERBB4,ESR1,F2,FABP7,FAS,FLNB,FN1,G6PD,GAD2,GALNT10,GART,GRK2,GRK6,GTF2I,HADH,HASPIN,HDX,HIBCH,HLA,DRB1,HMBOX1,HMGCS1,HMOX1,HNRNPA2B1,HSD17B10,HSP90AB1,IDE,IFNG,IFNGR1,IGF1R,ITGB3,ITSN1,KMT5A,KYNU,LRBA,MAGI2,MAP3K3,MARF1,MATK,MSH2,MSN,MTAP,MUC1,MYBPC3,NBEA,NCBP1,NCOA3,NF2,NFATC1,NMT1,NOTCH1,NSUN5,NUP214,ODC1,OXSR1,PA2G4,PAFAH1B2,PAH,PANK1,PAPSS1,PARN,PATJ,PC,PCYT2,PDHA1,PGK1,POU2F1,PPARG,PPP2R1A,PRKCB,PSAP,PTK2B,PUS10,PYGL,RAC1,RARA,RARG,RBBP6,RBP5,RRM1,RTN4IP1,SART3,SEMA4D,SENP2,SERPINA5,SFMBT2,SH2B2,SH3YL1,SLC5A1,SMURF2,SNX1,STARD13,SUB1,TAF13,THBS2,TJP1,TLR4,TNFAIP8L2,USP19,UTRN,VAV3,VPS26A,WEE1,WHRN,ZBTB43,ZCWPW1,ZEB2,ZFP28,ZIC3,ZNF292,ZNF462
349	Export of molecule	Molecular Transport	6.06E,06	14	ALB,ATP7B,CRYM,FAS,HMOX1,HNRNPA2B1,MSN,NCBP1,NUP214,PPARG,PRKCB,SMG5,SMG7,TTR
350	Non,traumatic arthropathy	Connective Tissue Disorders,Organismal Injury and Abnormalities,Skeletal and Muscular Disorders	6.12E,06	37	ALB,ALOX12,ANG,AR,ASAP1,B2M,CD1A,CD74,CSF2RB,DPP4,FAS,FCGR2A,FN1,GGPS1,HBB,HCK,HDAC7,HLA,DRB1,HMOX1,IDE,IFNG,IMPDH1,MMP1,MMP2,MTAP,NFATC1,PGK1,PPARG,PTK2B,PTPRE,RGS6,SF3B6,SMG7,TAF13,TJP1,TLR4,XIAP
351	Unresectable malignant solid tumor	Cancer,Organismal Injury and Abnormalities	6.18E,06	12	ALK,CSF2RB,ERBB4,ESR1,FN1,GART,HSP90AB1,IGF1R,PARP3,PTK2B,RRM1,VEGFB
352	Peritoneal tumor	Cancer,Organismal Injury and Abnormalities	6.20E,06	9	ALB,ALK,ESR1,GART,HSP90AB1,IFNGR1,PARP3,RRM1,XIAP
353	Outgrowth of cells	Cellular Growth and Proliferation	6.20E,06	9	AR,ESR1,FN1,IFNG,ITGB3,MMP2,NOTCH1,PSAP,PTK2B
354	Metabolism of nucleotide	Nucleic Acid Metabolism,Small Molecule Biochemistry	6.49E,06	17	DCTD,F2,FHIT,G6PD,GRK2,HMOX1,HTR1A,IFNG,IMPDH1,ITPA,KYNU,NMRK1,PAPSS1,PDHA1,PRKCB,SNX1,SULT1A1
355	Immunodeficiency	Immunological Disease	6.49E,06	19	ARHGEF1,B2M,BTK,C8A,CA8,CD74,DAPK2,FAS,FCGR2A,G6PD,GRHPR,IFNGR1,LRBA,MSN,PPARG,STK4,TAF13,TLR4,XIAP
356	Cell death of carcinoma cell lines	Cell Death and Survival	6.50E,06	22	ALK,AR,DAPK2,EP300,FABP7,FAS,FHIT,FN1,G6PD,HK2,HMOX1,IFNG,IGF1R,ITGB3,MSH2,NCOA3,NOTCH1,PPARG,RAC1,RRM1,TLR4,XIAP
357	Stage IIIA,IV invasive breast cancer	Cancer,Organismal Injury and Abnormalities,Reproductive System Disease	6.57E,06	7	ALB,AR,ERBB4,ESR1,HSP90AB1,PARP3,RRM1
358	Brain lesion	Neurological Disease,Organismal Injury and Abnormalities	6.59E,06	177	ACACB,ACADSB,ACADVL,ACVR2B,ACY1,ADH7,ALB,ALK,ALOX12,AMY1C (includes others),AOC3,APEX1,APPL1,ARHGAP5,ARHGEF1,ASAP1,ATP7B,ATXN3,B3GAT2,BCR,BMP1,BPI,C1R,C8A,CLCN5,CRYM,CSDE1,CSF2RB,CSNK1G1,CSNK1G2,CSTF2,CTCF,CUL1,CYP2E1,DAPK2,DCXR,DGKA,DOK2,DPP4,DSG2,EP300,EPHA3,EPS8L2,ERBB4,ESR1,F2,FAS,FCGR2A,FLNB,FN1,G6PD,GAD2,GALNT10,GART,GRK2,GRK6,GTF2I,HADH,HASPIN,HAT1,HDX,HIBCH,HLA,DRB1,HMBOX1,HMGB3,HMGCS1,HMOX1,HNRNPA2B1,HSD17B10,HSD17B4,HSP90AB1,HTR1A,IDE,IFNGR1,IGF1R,IMPDH1,ITGB3,ITPK1,ITSN1,KMT5A

					,KYNU,LRBA,MAGEA4,MAGI2,MAP3K3,MARF1,MATK,MMP2,MSH2,MSN,MTAP,MUC1,MYBPC1,MYBPC3,NBEA,NCBP1,NCOA3,NF2,NFATC1,NME3,NMT1,NOTCH1,NR1I3,NSUN5,NUP214,ODC1,OXSR1,PAFAH1B2,PAH,PANK1,PAPSS1,PARN,PATJ,PC,PCYT2,PDHA1,PDK4,PGC,PGK1,PHF24,PIP4K2C,POT1,POU2F1,PPARG,PPP2R1A,PRKCB,PSAP,PTK2B,PTPRE,PTPRF,PUS10,PYGL,RAC1,RARA,RARG,RBBP6,RBM39,RBP5,RGS6,RRM1,RTN4IP1,SART3,SEMA4D,SENP2,SERPINA5,SFMBT2,SH2B2,SH3YL1,SLC5A1,SLK,SMG5,SMURF2,SNX1,STARD13,SUB1,THBS2,TIMM9,TJP1,TLR4,TNFAIP8L2,USP19,UTRN,VAV3,VEGFB,VPS26A,WEE1,WHRN,XIAP,XRCC6,YWHAQ,ZBTB43,ZCWPW1,ZEB2,ZFP28,ZIC3,ZNF292,ZNF462
359	Interphase	Cell Cycle	6.71E,06	30	AASDHPPT,AR,BTRC,CHKA,CUL1,DPP4,EP300,F2,FAS,FHIT,FN1,HBEGF,HMOX1,IFNG,IGF1R,KIF11,MSH2,NAE1,NCOA3,NOTCH1,PPARG,PPP2R1A,RBBP6,RPA3,SART3,SFN,SKP1,TRIM21,WEE1,YWHAQ
360	Digestive system cancer	Cancer,Gastrointestinal Disease,Organismal Injury and Abnormalities	7.14E,06	251	ACACB,ACADSB,ACADVL,ACAT2,ACSM2A,ACVR2B,ACY1,ADH1C,ADH7,ALB,ALK,ALOX12,ANG,ANXA6,AOC3,APEX1,APPL1,AR,ARF6,ARHGAP5,ARHGEF1,ASAP1,ATP7B,ATXN3,B2M,B3GAT2,BCR,BMP1,BPI,BTK,BTRC,C1R,C8A,CAMK4,CANT1,CD1A,CD74,CDK5R1,CHKA,CLCN5,CLK1,CRYM,CSDE1,CSF2RB,CSNK1G1,CSNK1G2,CSTF2,CTCF,CUL1,CYP17A1,CYP2E1,CYP2R1,CYP7A1,DAPK2,DCTD,DCXR,DFFA,DGKA,DHPS,DMC1,DOK2,DPP4,DSG2,ECHS1,ECI2,EP300,EPHA3,EPS8L2,ERBB4,ESR1,ETF1,EXOSC9,F2,F3,FABP7,FAP,FAS,FCGR2A,FHIT,FLNB,FN1,G6PD,GAD2,GALM,GALNT10,GART,GC,GGPS1,GRHPR,GRK2,GRK6,GTF2F2,GTF2I,HADH,HASPIN,HBB,HBEGF,HCK,HDAC7,HDX,HIBCH,HK2,HLA,DRB1,HMGB3,HMGCS1,HMOX1,HNRNPA2B1,HPD,HSCB,HSD17B10,HSD17B4,HSP90AB1,HTR1A,IDE,IFNG,IFNGR1,IGF1R,IMPA2,IMPDH1,IQUB,ITGB3,ITPK1,ITPKC,ITSN1,KIF11,KLK1,KMT5A,KYNU,LRBA,MAGEA4,MAGI2,MAP3K3,MAPK6,MARF1,MARK1,MATK,MDH1,MECR,MMP2,MSH2,MSN,MTAP,MUC1,MYBPC1,MYBPC3,NAE1,NBEA,NCBP1,NCOA3,NF2,NFATC1,NMRK1,NMT1,NOTCH1,NSUN5,NT5C3A,NUP214,OAT,ODC1,OXSR1,PA2G4,PAFAH1B2,PAH,PANK1,PAPSS1,PARN,PARP3,PATJ,PC,PCYT2,PDHA1,PDK2,PDK3,PDK4,PEX5,PGC,PGK1,PHF24,POT1,POU2F1,PPARG,PPP2R1A,PRKCB,PSAP,PTK2B,PTPRE,PTPRF,PUS10,PYGL,RAB26,RAC1,RARA,RARG,RBBP6,RBM39,RGS6,RND1,RRM1,RTN4IP1,RUVBL1,S100A6,SART3,SEC23A,SEMA4D,SERPINA5,SF3B6,SFMBT2,SFN,SH2B2,SH3YL1,SKP1,SLC5A1,SLK,SMAP1,SMG5,SMG7,SMTN,SMURF2,SNX1,SPEF1,STARD13,STK16,STK4,SULT1A1,SYT13,TAF13,THBS2,TJP1,TLR4,TNFAIP8L2,TRIM21,TTR,UEVLD,USP19,UTRN,VAV3,VEGFB,VPS26A,WEE1,WHRN,WWP1,XRCC6,YWHAH,YY1,ZBTB43,ZCWPW1,ZEB2,ZFP28,ZIC3,ZNF292,ZNF462
361	Large intestine carcinoma	Cancer,Gastrointestinal Disease,Organismal Injury and Abnormalities	7.26E,06	218	ACACB,ACADSB,ACADVL,ACSM2A,ACVR2B,ACY1,ADH7,ALB,ALK,ALOX12,ANXA6,AOC3,APEX1,APPL1,AR,ARF6,ARHGAP5,ARHGEF1,ASAP1,ATP7B,ATXN3,B2M,B3GAT2,BCR,BMP1,BPI,BTK,BTRC,C1R,C8A,CAMK4,CANT1,CD1A,CD74,CDK5R1,CHKA,CLCN5,CSDE1,CSF2RB,CSNK1G2,CSTF2,CTCF,CUL1,CYP17A1,CYP2E1,CYP7A1,DAPK2,DCXR,DFFA,DGKA,DHPS,DMC1,DOK2,DPP4,DSG2,ECHS1,EP300,EPHA3,EPS8L2,ERBB4,ESR1,ETF1,EXOSC9,F2,F3,FAP,FAS,FHIT,FLNB,FN1,G6PD,GAD2,GALNT10,GART,GRHPR,GRK2,GRK6,GTF2I,HADH,HASPIN,HCK,HDAC7,HDX,HIBCH,HK2,HLA,DRB1,HMGB3,HMOX1,HNRNPA2B1,HPD,HSCB,HSD17B10,HSD17B4,HSP90AB1,HTR1A,IDE,IFNGR1,IGF1R,IMPA2,IMPDH1,IQUB,ITGB3,ITPK1,ITPKC,ITSN1,KIF11,KMT5A,KYNU,LRBA,MAGEA4,MAGI2,MAP3K3,MAPK6,MARF1,MARK1,MATK,MECR,MMP2,MSH2,MSN,MTAP,MUC1,MYBPC1,MYBPC3,NAE1,NBEA,NCBP1,NCOA3,NF2,NFATC1,NMRK1,NMT1,NOTCH1,NSUN5,NT5C3A,NUP214,OAT,ODC1,OXSR1,PAFAH1B2,PANK1,PARN,PARP3,PATJ,PC,PCYT2,PDHA1,PDK2,PDK3,PDK4,PEX5,PGC,PGK1,PHF24,POT1,POU2F1,PPARG,PPP2R1A,PRKCB,PTK2B,PTPRF,PUS10,PYGL,RAB26,RAC1,RARA,RARG,RBBP6,RBM39,RGS6,RND1,RRM1,RTN4IP1,RUVBL1,SART3,SEC23A,SEMA4D,SERPINA5,SF3B6,SFMBT2,SH2B2,SH3YL1,SKP1,SLC5A1,SLK,SMAP1,SMG5,SMG7,SMTN,SMURF2,SNX1,SPEF1,STARD13,STK16,SYT13,TAF13,THBS2,TJP1,TLR4,TRIM21,UEVLD,USP19,UTRN,VAV3,VEGFB,VPS26A,WHRN,WWP1,XRCC6,YWHAH,YY1,ZBTB43,ZCWPW1,ZEB2,ZFP28,ZIC3,ZNF292,ZNF462
362	Metabolism of reactive oxygen species	Free Radical Scavenging	7.35E,06	18	ALB,AOC3,CYP2E1,F2,FAS,FCGR2A,G6PD,HBB,HK2,IFNG,ITGB3,ITSN1,MUC1,PDK4,PRKCB,RAC1,S100A6,TLR4
363	Metastatic progressive tumor	Cancer,Organismal Injury and Abnormalities,Tumor Morphology	7.53E,06	10	ALK,AR,ERBB4,ESR1,GART,HSP90AB1,IGF1R,PTK2B,RRM1,VEGFB

364	Invasive mammary tumor	Cancer,Organismal Injury and Abnormalities,Reproductive System Disease	7.67E,06	19	ALB,ALK,AR,CSF2RB,ERBB4,ESR1,FAP,FAS,FN1,HSP90AB1,MMP2,MYBPC3,PARP3,PDK4,PTPRE,PTPRF,RBM39,RRM1,YWHAQ
365	Cell movement of granulocytes	Cellular Movement,Hematological System Development and Function,Immune Cell Trafficking	7.77E,06	12	ALB,DAPK2,DPP4,FAS,FN1,IFNG,ITGB3,PPARG,PRKCB,RAC1,TLR4,YARS1
366	Organization of cytoplasm	Cellular Assembly and Organization,Cellular Function and Maintenance	8.02E,06	36	AR,ARF6,ASAP1,ATXN3,BTK,CAMK4,CDK5R1,EPHA3,ERBB4,ESR1,F2,F3,FAS,FCGR2A,FHIT,FN1,GRK2,HCK,HSD17B10,IFNG,ITGB3,KIF11,MATK,MSN,NDC80,NF2,PARP3,PRKCB,PTPRF,RAC1,RND1,SEMA4D,SLK,SPEF1,TJP1,TLR4
367	Follicular lymphoma	Cancer,Hematological Disease,Immunological Disease,Organismal Injury and Abnormalities	8.10E,06	10	B2M,BTK,EP300,FAS,FCGR2A,HSP90AB1,IFNG,NOTCH1,RRM1,XIAP
368	Colony formation of tumor cell lines	Cellular Development,Cellular Growth and Proliferation	8.30E,06	21	ALK,AR,CTCF,ERBB4,ESR1,HCK,HNRNPA2B1,IFNG,IGF1R,MUC1,NCOA3,NOTCH1,PDHA1,PDK4,PPP2R1A,PRKCB,RARG,SFN,STK4,VEGFB,XIAP
369	Central nervous system cancer	Cancer,Neurological Disease,Organismal Injury and Abnormalities	8.38E,06	179	ACACB,ACADSB,ACADVL,ACVR2B,ACY1,ADH7,ALB,ALK,ALOX12,AMY1C (includes others),AOC3,APEX1,APPL1,ARHGAP5,ARHGEF1,ASAP1,ATP7B,ATXN3,B3GAT2,BCR,BMP1,BPI,BTK,C1R,C8A,CLCN5,CRYM,CSDE1,CSF2RB,CSNK1G1,CSNK1G2,CSTF2,CTCF,CUL1,CYP2E1,DAPK2,DCXR,DGKA,DOK2,DPP4,DSG2,EP300,EPHA3,EPS8L2,ERBB4,ESR1,F2,FABP7,FAS,FCGR2A,FLNB,FN1,G6PD,GAD2,GALNT10,GART,GRK2,GRK6,GTF2I,HADH,HASPIN,HAT1,HDX,HIBCH,HLA,DRB1,HMBOX1,HMGCS1,HMOX1,HNRNPA2B1,HSD17B10,HSD17B4,HSP90AB1,IDE,IFNG,IFNGR1,IGF1R,ITGB3,ITPK1,ITSN1,KMT5A,KYNU,LRBA,MAGEA4,MAGI2,MAP3K3,MARF1,MATK,MMP2,MSH2,MSN,MTAP,MUC1,MYBPC1,MYBPC3,NAE1,NBEA,NCBP1,NCOA3,NF2,NFATC1,NME3,NMT1,NOTCH1,NR1I3,NSUN5,NUP214,ODC1,OXSR1,PA2G4,PAFAH1B2,PAH,PANK1,PAPSS1,PARN,PATJ,PC,PCYT2,PDHA1,PDK4,PGC,PGK1,PHF24,PIP4K2C,POT1,POU2F1,PPARG,PPP2R1A,PRKCB,PSAP,PTK2B,PTPRE,PTPRF,PUS10,PYGL,RAC1,RARA,RARG,RBBP6,RBM39,RBP5,RGS6,RRM1,RTN4IP1,SART3,SEMA4D,SENP2,SERPINA5,SFMBT2,SH2B2,SH3YL1,SLC5A1,SLK,SMG5,SMURF2,SNX1,STARD13,SUB1,TAF13,THBS2,TIMM9,TJP1,TLR4,TNFAIP8L2,USP19,UTRN,VAV3,VEGFB,VPS26A,WEE1,WHRN,XRCC6,YWHAQ,ZBTB43,ZCWPW1,ZEB2,ZFP28,ZIC3,ZNF292,ZNF462
370	Growth of malignant tumor	Cancer,Organismal Injury and Abnormalities	8.44E,06	13	AR,BTK,ESR1,FHIT,IFNG,IGF1R,MUC1,NOTCH1,ODC1,PRKCB,PTK2B,RUVBL1,XIAP
371	Recurrent nonsquamous non,small cell lung carcinoma	Cancer,Organismal Injury and Abnormalities,Respiratory Disease	8.50E,06	7	ALB,ALK,ERBB4,GART,HSP90AB1,IGF1R,RRM1
372	Lung adenocarcinoma	Cancer,Organismal Injury and Abnormalities,Respiratory Disease	8.54E,06	70	ADH1C,ADH7,ALK,ALOX12,AR,B2M,BTK,CD1A,CERT1,CSNK1G2,CTCF,DGKA,DOK2,EP300,EPHA3,ERBB4,ESR1,F3,FN1,GAD2,GART,GRK6,HASPIN,HCK,HMGB3,HPD,HSD17B4,HSP90AB1,HTR1A,IGF1R,ITPKC,KIF11,KYNU,LRBA,MAGEA4,MAGI2,MAPK6,MATK,MMP1,MSH2,MSN,MUC1,NBEA,NDC80,NMT1,NOTCH1,NT5C3A,OAT,OXSR1,PAH,PATJ,PDK4,PIP4K2C,PPP2R1A,PRKCB,PTK2B,PTPRE,PYGL,RARA,RRM1,SEMA4D,SERPINA5,SFN,SLK,SMG7,STK4,TLR4,TTR,ZCWPW1,ZNF292
373	Stage IV advanced solid tumor	Cancer,Organismal Injury and Abnormalities	8.65E,06	11	ALB,ALK,BTK,ERBB4,ESR1,GART,HSP90AB1,IGF1R,PARP3,PTK2B,RRM1
374	Recurrent advanced cancer	Cancer,Organismal Injury and Abnormalities	8.71E,06	9	ALB,ALK,AR,ERBB4,ESR1,GART,HSP90AB1,PTK2B,RRM1

375	Uterine corpus cancer	Cancer,Organismal Injury and Abnormalities,Reproductive System Disease	9.21E,06	11	AR,CTCF,EP300,EPHA3,ERBB4,HSP90AB1,NOTCH1,PPP2R1A,RAC1,RRM1,TLR4
376	Inflammation of absolute anatomical region	Inflammatory Response	9.25E,06	30	ALB,ALK,ALOX12,AR,CSF2RB,CYP17A1,CYP2E1,DHPS,EP300,ESR1,F2,F3,FAS,FCGR2A,FN1,GAD2,GC,IDE,IFNG,IFNGR1,IGF1R,IMPDH1,ITGB3,MUC1,PPARG,RAC1,RARA,SMURF2,TAF13,TLR4
377	Locally advanced non,small cell lung carcinoma	Cancer,Organismal Injury and Abnormalities,Respiratory Disease	9.35E,06	8	ALK,ERBB4,GART,HSP90AB1,IGF1R,PARP3,PTK2B,RRM1
378	Brain tumor	Cancer,Neurological Disease,Organismal Injury and Abnormalities	9.37E,06	176	ACACB,ACADSB,ACADVL,ACVR2B,ACY1,ADH7,ALB,ALK,ALOX12,AMY1C (includes others),AOC3,APEX1,APPL1,ARHGAP5,ARHGEF1,ASAP1,ATP7B,ATXN3,B3GAT2,BCR,BMP1,BPI,C1R,C8A,CLCN5,CRYM,CSDE1,CSF2RB,CSNK1G1,CSNK1G2,CSTF2,CTCF,CUL1,CYP2E1,DAPK2,DCXR,DGKA,DOK2,DPP4,DSG2,EP300,EPHA3,EPS8L2,ERBB4,ESR1,F2,FAS,FCGR2A,FLNB,FN1,G6PD,GAD2,GALNT10,GART,GRK2,GRK6,GTF2I,HADH,HASPIN,HAT1,HDX,HIBCH,HLA,DRB1,HMBOX1,HMGB3,HMGCS1,HMOX1,HNRNPA2B1,HSD17B10,HSD17B4,HSP90AB1,IDE,IFNGR1,IGF1R,IMPDH1,ITGB3,ITPK1,ITSN1,KMT5A,KYNU,LRBA,MAGEA4,MAGI2,MAP3K3,MARF1,MATK,MMP2,MSH2,MSN,MTAP,MUC1,MYBPC1,MYBPC3,NBEA,NCBP1,NCOA3,NF2,NFATC1,NME3,NMT1,NOTCH1,NR1I3,NSUN5,NUP214,ODC1,OXSR1,PAFAH1B2,PAH,PANK1,PAPSS1,PARN,PATJ,PC,PCYT2,PDHA1,PDK4,PGC,PGK1,PHF24,PIP4K2C,POT1,POU2F1,PPARG,PPP2R1A,PRKCB,PSAP,PTK2B,PTPRE,PTPRF,PUS10,PYGL,RAC1,RARA,RARG,RBBP6,RBM39,RBP5,RGS6,RRM1,RTN4IP1,SART3,SEMA4D,SENP2,SERPINA5,SFMBT2,SH2B2,SH3YL1,SLC5A1,SLK,SMG5,SMURF2,SNX1,STARD13,SUB1,THBS2,TIMM9,TJP1,TLR4,TNFAIP8L2,USP19,UTRN,VAV3,VEGFB,VPS26A,WEE1,WHRN,XIAP,XRCC6,YWHAQ,ZBTB43,ZCWPW1,ZEB2,ZFP28,ZIC3,ZNF292,ZNF462
379	Primitive neuroectodermal tumor	Cancer,Neurological Disease,Organismal Injury and Abnormalities	9.79E,06	15	ALK,AR,CSF2RB,CTCF,EP300,ERBB4,FAP,FCGR2A,HSP90AB1,IDE,IGF1R,PGK1,RARA,RARG,UTRN
380	Unresectable advanced cancer	Cancer,Organismal Injury and Abnormalities	9.79E,06	11	ALK,ERBB4,ESR1,FN1,GART,HSP90AB1,IGF1R,PARP3,PTK2B,RRM1,VEGFB
381	Synthesis of reactive oxygen species	Free Radical Scavenging	1.01E,05	17	ALB,AOC3,CYP2E1,F2,FAS,FCGR2A,G6PD,HBB,HK2,IFNG,ITGB3,ITSN1,MUC1,PRKCB,RAC1,S100A6,TLR4
382	Grade 3,4 tumor	Cancer,Organismal Injury and Abnormalities	1.02E,05	154	ACACB,ACADVL,ACVR2B,ADH7,ALB,ALK,ALOX12,AMY1C (includes others),APEX1,APPL1,ARHGAP5,ARHGEF1,ASAP1,ATP7B,ATXN3,B3GAT2,BMP1,BPI,C1R,C8A,CLCN5,CSDE1,CSF2RB,CSNK1G2,CSTF2,CTCF,CUL1,CYP2E1,DAPK2,DCXR,DGKA,DMC1,DOK2,DPP4,DSG2,EP300,EPHA3,EPS8L2,ERBB4,ESR1,F2,FABP7,FAS,FCGR2A,FLNB,FN1,G6PD,GAD2,GALNT10,GART,GRK2,GRK6,GTF2I,HADH,HASPIN,HDX,HIBCH,HLA,DRB1,HMBOX1,HMGCS1,HMOX1,HSD17B10,HSD17B4,HSP90AB1,IDE,IFNG,IFNGR1,IGF1R,ITGB3,ITSN1,KMT5A,KYNU,LRBA,MAGI2,MAP3K3,MARF1,MATK,MMP2,MSH2,MSN,MTAP,MUC1,MYBPC3,NBEA,NCBP1,NCOA3,NF2,NFATC1,NME3,NMT1,NOTCH1,NSUN5,NUP214,ODC1,OXSR1,PAFAH1B2,PAH,PANK1,PAPSS1,PARN,PATJ,PC,PCYT2,PDHA1,PGC,PGK1,PHF24,POU2F1,PPARG,PPP2R1A,PSAP,PTK2B,PUS10,PYGL,RARA,RARG,RBBP6,RBM39,RBP5,RGS6,RRM1,RTN4IP1,SART3,SEMA4D,SENP2,SERPINA5,SFMBT2,SH2B2,SH3YL1,SLC5A1,SLK,SMG5,SMURF2,SNX1,STARD13,SUB1,THBS2,TJP1,TLR4,TNFAIP8L2,USP19,UTRN,VAV3,VPS26A,WEE1,WHRN,XRCC6,ZBTB43,ZCWPW1,ZEB2,ZFP28,ZIC3,ZNF292,ZNF462
383	Cell death of neuroblastoma cell lines	Cell Death and Survival	1.08E,05	12	ANG,APPL1,AR,CAMK4,DPP4,FAS,IDE,IFNG,IGF1R,PRKCB,RARG,S100A6
384	Aggregation of cells	Cell,To,Cell Signaling and Interaction	1.08E,05	12	ALB,ALOX12,BTK,CANT1,DSG2,F2,FCGR2A,HNRNPA2B1,IGF1R,ITGB3,SEMA4D,TLR4
385	Early stage solid tumor	Cancer,Organismal Injury and Abnormalities	1.11E,05	15	ALB,ALK,B2M,CSF2RB,ERBB4,ESR1,FN1,GART,GGPS1,HSP90AB1,MUC1,PGC,RARA,RARG,RRM1

386	Inflammation of body cavity	Inflammatory Response	1.12E,05	29	ALB,ALK,ALOX12,AR,CSF2RB,CYP2E1,DHPS,EP300,ESR1,F2,F3,FAS,FCGR2A,FN1,GAD2,GC,IDE,IFNG,IFNGR1,IGF1R,IMPDH1,ITGB3,MUC1,PPARG,RAC1,RARA,SMURF2,TAF13,TLR4
387	Stage I non,small cell lung carcinoma	Cancer,Organismal Injury and Abnormalities,Respiratory Disease	1.14E,05	8	ALK,B2M,ERBB4,FN1,GART,HSP90AB1,PGC,RRM1
388	Metastatic recurrent cancer	Cancer,Organismal Injury and Abnormalities	1.14E,05	8	ALB,AR,CYP17A1,ERBB4,ESR1,GGPS1,HSP90AB1,RRM1
389	Benign pelvic disease	Organismal Injury and Abnormalities,Reproductive System Disease	1.16E,05	28	APEX1,AR,ATP7B,CLCN5,ESR1,FAS,FN1,HSD17B10,HSD17B4,HSP90AB1,IGF1R,ITGB3,MDH1,MMP1,MMP2,MUC1,PDK4,PPARG,PRKCB,RAC1,RRM1,S100A6,TAF13,THBS2,UTRN,WEE1,XRCC6,YWHAH
390	Metabolism of DNA	DNA Replication, Recombination, and Repair	1.19E,05	16	APEX1,CUL1,DFFA,EP300,FAS,IFNG,IGF1R,KMT5A,MSH2,NAE1,PGK1,RBBP6,RPA3,SUB1,WEE1,XRCC6
391	Benign Tumors	Cancer,Organismal Injury and Abnormalities	1.22E,05	35	ALK,AR,BCR,CLCN5,CSF2RB,EP300,ERBB4,ESR1,FABP3,FAS,FN1,GGPS1,HDAC7,HSD17B10,HSP90AB1,IGF1R,IMPDH1,MDH1,MMP2,MSH2,MSN,MUC1,NOTCH1,ODC1,PDK4,PPARG,PRKCB,RARA,RRM1,RUVBL1,S100A6,TAF13,UTRN,WEE1,ZEB2
392	Large intestine adenocarcinoma	Cancer,Gastrointestinal Disease,Organismal Injury and Abnormalities	1.23E,05	216	ACACB,ACADSB,ACADVL,ACSM2A,ACVR2B,ACY1,ADH7,ALB,ALK,ALOX12,ANXA6,AOC3,APEX1,APPL1,AR,ARF6,ARHGAP5,ARHGEF1,ASAP1,ATP7B,ATXN3,B2M,B3GAT2,BCR,BMP1,BPI,BTK,BTRC,C1R,C8A,CAMK4,CANT1,CD1A,CD74,CDK5R1,CHKA,CLCN5,CSDE1,CSF2RB,CSNK1G2,CSTF2,CTCF,CUL1,CYP17A1,CYP2E1,CYP7A1,DAPK2,DCXR,DFFA,DGKA,DHPS,DMC1,DOK2,DPP4,DSG2,ECHS1,EP300,EPHA3,EPS8L2,ERBB4,ESR1,ETF1,EXOSC9,F2,F3,FAP,FAS,FHIT,FLNB,FN1,G6PD,GAD2,GALNT10,GART,GRHPR,GRK2,GRK6,GTF2I,HADH,HASPIN,HCK,HDAC7,HDX,HIBCH,HK2,HLA,DRB1,HMGB3,HMOX1,HNRNPA2B1,HPD,HSCB,HSD17B10,HSD17B4,HSP90AB1,HTR1A,IDE,IFNGR1,IGF1R,IMPA2,IMPDH1,IQUB,ITGB3,ITPK1,ITPKC,ITSN1,KIF11,KMT5A,KYNU,LRBA,MAGEA4,MAGI2,MAP3K3,MAPK6,MARF1,MARK1,MATK,MECR,MMP2,MSH2,MSN,MTAP,MUC1,MYBPC1,MYBPC3,NAE1,NBEA,NCBP1,NCOA3,NF2,NFATC1,NMRK1,NMT1,NOTCH1,NSUN5,NT5C3A,NUP214,OAT,ODC1,OXSR1,PAFAH1B2,PANK1,PARN,PARP3,PATJ,PC,PCYT2,PDHA1,PDK2,PDK3,PDK4,PGC,PGK1,PHF24,POT1,POU2F1,PPARG,PPP2R1A,PRKCB,PTK2B,PTPRF,PUS10,PYGL,RAB26,RAC1,RARA,RARG,RBBP6,RBM39,RGS6,RND1,RRM1,RTN4IP1,RUVBL1,SART3,SEC23A,SEMA4D,SERPINA5,SF3B6,SFMBT2,SH2B2,SH3YL1,SKP1,SLC5A1,SLK,SMAP1,SMG5,SMG7,SMTN,SMURF2,SNX1,SPEF1,STARD13,STK16,SYT13,THBS2,TJP1,TLR4,TRIM21,UEVLD,USP19,UTRN,VAV3,VEGFB,VPS26A,WHRN,WWP1,XRCC6,YWHAH,YY1,ZBTB43,ZCWPW1,ZEB2,ZFP28,ZIC3,ZNF292,ZNF462
393	Peripheral T,cell lymphoma	Cancer,Hematological Disease,Immunological Disease,Organismal Injury and Abnormalities	1.23E,05	16	ALK,C1R,CLK1,EPHA3,ERBB4,FN1,GTF2I,HDAC7,HSP90AB1,MMP1,MMP2,NFATC1,NT5C3A,POU2F1,RRM1,TJP1
394	Development of tumor cell lines	Cellular Development,Cellular Growth and Proliferation	1.24E,05	22	ALK,AR,CTCF,ERBB4,ESR1,FN1,HCK,HNRNPA2B1,IFNG,IGF1R,MUC1,NCOA3,NOTCH1,PDHA1,PDK4,PPP2R1A,PRKCB,RARG,SFN,STK4,VEGFB,XIAP
395	Activation of cells	Cell,To,Cell Signaling and Interaction	1.24E,05	23	ALB,BPI,BTK,CD1A,DGKA,DPP4,F2,F3,FAS,FCGR2A,FN1,GC,HBEGF,IFNG,ITGB3,MMP1,MMP2,PPARG,PRKCB,PTK2B,SEMA4D,TLR4,VAV3
396	Peritoneal cavity cancer	Cancer,Organismal Injury and Abnormalities	1.25E,05	8	ALB,ALK,ESR1,GART,HSP90AB1,IFNGR1,PARP3,RRM1
397	Development of cytoplasm	Cellular Assembly and Organization	1.27E,05	18	ANG,AR,ARF6,CD74,DGKA,F2,FCGR2A,FHIT,FN1,HCK,ITGB3,NF2,NFATC1,NOTCH1,PTK2B,RAC1,STK4,XIAP
398	Digestive organ tumor	Cancer,Gastrointestinal Disease,Organismal Injury and Abnormalities	1.29E,05	253	ACACB,ACADSB,ACADVL,ACAT2,ACSM2A,ACVR2B,ACY1,ADH1C,ADH7,ALB,ALK,ALOX12,ANG,ANXA6,AOC3,APEX1,APPL1,AR,ARF6,ARHGAP5,ARHGEF1,ASAP1,ATP7B,ATXN3,B2M,B3GAT2,BCR,BMP1,BPI,BTK,BTRC,C1R,C8A,CAMK4,CANT1,CD1A,CD74,CDK5R1,CHKA,CLCN5,CLK1,CRYM,CSDE1,CSF2RB,CSNK1G1,CSNK1G2,CSTF2,CTCF,CUL1,CYP17A1,CYP2E1,CYP2R1,CYP7A1,DAPK2,DCTD,DCXR,DFFA,DGK

					A,DHPS,DMC1,DOK2,DPP4,DSG2,ECHS1,ECI2,EP300,EPHA3,EPS8L2,ERBB4,ERI1,ESR1,ETF1,EXOSC9,F2,F3,FABP7,FAP,FAS,FCGR2A,FHIT,FLNB,FN1,G6PD,GAD2,GALM,GALNT10,GART,GC,GGPS1,GRHPR,GRK2,GRK6,GTF2F2,GTF2I,HADH,HASPIN,HBB,HBEGF,HCK,HDAC7,HDX,HIBCH,HK2,HLA,DRB1,HMGB3,HMGCS1,HMOX1,HNRNPA2B1,HPD,HSCB,HSD17B10,HSD17B4,HSP90AB1,HTR1A,IDE,IFNG,IFNGR1,IGF1R,IMPA2,IMPDH1,IQUB,ITGB3,ITPK1,ITPKC,ITSN1,KIF11,KLK1,KMT5A,KYNU,LRBA,MAGEA4,MAGI2,MAP3K3,MAPK6,MARF1,MARK1,MATK,MDH1,MECR,MMP2,MSH2,MSN,MTAP,MUC1,MYBPC1,MYBPC3,NAE1,NBEA,NCBP1,NCOA3,NF2,NFATC1,NMRK1,NMT1,NOTCH1,NSUN5,NT5C3A,NUP214,OAT,ODC1,OXSR1,PA2G4,PAFAH1B2,PAH,PANK1,PAPSS1,PARN,PARP3,PATJ,PC,PCYT2,PDHA1,PDK2,PDK3,PDK4,PEX5,PGC,PGK1,PHF24,POT1,POU2F1,PPARG,PPP2R1A,PRKCB,PSAP,PTK2B,PTPRE,PTPRF,PUS10,PYGL,RAB26,RAC1,RARA,RARG,RBBP6,RBM39,RGS6,RND1,RRM1,RTN4IP1,RUVBL1,S100A6,SART3,SEC23A,SEMA4D,SERPINA5,SF3B6,SFMBT2,SFN,SH2B2,SH3YL1,SKP1,SLC5A1,SLK,SMAP1,SMG5,SMG7,SMTN,SMURF2,SNX1,SNX22,SPEF1,STARD13,STK16,STK4,SULT1A1,SYT13,TAF13,THBS2,TJP1,TLR4,TNFAIP8L2,TRIM21,TTR,UEVLD,USP19,UTRN,VAV3,VEGFB,VPS26A,WEE1,WHRN,WWP1,XRCC6,YWHAH,YY1,ZBTB43,ZCWPW1,ZEB2,ZFP28,ZIC3,ZNF292,ZNF462
399	Cell cycle progression of tumor cell lines	Cell Cycle	1.30E,05	17	AR,CAMK4,EP300,ESR1,HBEGF,HMOX1,IGF1R,NOTCH1,PPARG,RAC1,RARA,RRM1,SFN,TLR4,WEE1,XIAP,XRCC6
400	HER2 negative hormone receptor negative breast cancer	Cancer,Organismal Injury and Abnormalities,Reproductive System Disease	1.31E,05	13	AR,CERT1,ERBB4,ESR1,FAS,HCK,HSP90AB1,MMP2,MUC1,PARP3,PPARG,RRM1,WEE1
401	Glioma cancer	Cancer,Neurological Disease,Organismal Injury and Abnormalities	1.32E,05	176	ACACB,ACADSB,ACADVL,ACVR2B,ADH7,ALB,ALK,ALOX12,AMY1C (includes others),AOC3,APEX1,APPL1,ARHGAP5,ARHGEF1,ASAP1,ATP7B,ATXN3,B3GAT2,BCR,BMP1,BPI,C1R,C8A,CLCN5,CRYM,CSDE1,CSF2RB,CSNK1G1,CSNK1G2,CSTF2,CTCF,CUL1,CYP2E1,DAPK2,DCXR,DGKA,DOK2,DPP4,DSG2,EP300,EPHA3,EPS8L2,ERBB4,ESR1,F2,FABP7,FAS,FCGR2A,FLNB,FN1,G6PD,GAD2,GALNT10,GART,GRK2,GRK6,GTF2I,HADH,HASPIN,HAT1,HDX,HIBCH,HLA,DRB1,HMBOX1,HMGCS1,HMOX1,HNRNPA2B1,HSD17B10,HSD17B4,HSP90AB1,IDE,IFNG,IFNGR1,IGF1R,ITGB3,ITPK1,ITSN1,KMT5A,KYNU,LRBA,MAGEA4,MAGI2,MAP3K3,MARF1,MATK,MMP2,MSH2,MSN,MTAP,MUC1,MYBPC1,MYBPC3,NBEA,NCBP1,NCOA3,NF2,NFATC1,NME3,NMT1,NOTCH1,NR1I3,NSUN5,NUP214,ODC1,OXSR1,PA2G4,PAFAH1B2,PAH,PANK1,PAPSS1,PARN,PATJ,PC,PCYT2,PDHA1,PDK4,PGC,PGK1,PHF24,PIP4K2C,POT1,POU2F1,PPARG,PPP2R1A,PRKCB,PSAP,PTK2B,PTPRE,PTPRF,PUS10,PYGL,RAC1,RARA,RARG,RBBP6,RBM39,RBP5,RGS6,RRM1,RTN4IP1,SART3,SEMA4D,SENP2,SERPINA5,SFMBT2,SH2B2,SH3YL1,SLC5A1,SLK,SMG5,SMURF2,SNX1,STARD13,SUB1,TAF13,THBS2,TIMM9,TJP1,TLR4,TNFAIP8L2,USP19,UTRN,VAV3,VEGFB,VPS26A,WEE1,WHRN,XRCC6,YWHAQ,ZBTB43,ZCWPW1,ZEB2,ZFP28,ZIC3,ZNF292,ZNF462
402	Interphase of tumor cell lines	Cell Cycle	1.34E,05	23	AASDHPPT,AR,BTRC,CHKA,DPP4,EP300,F2,FAS,FHIT,HMOX1,IFNG,IGF1R,KIF11,MSH2,NAE1,NCOA3,NOTCH1,PPARG,SART3,SFN,TRIM21,WEE1,YWHAQ
403	Brain glioma	Cancer,Neurological Disease,Organismal Injury and Abnormalities	1.37E,05	174	ACACB,ACADSB,ACADVL,ACVR2B,ADH7,ALB,ALK,ALOX12,AMY1C (includes others),AOC3,APEX1,APPL1,ARHGAP5,ARHGEF1,ASAP1,ATP7B,ATXN3,B3GAT2,BCR,BMP1,BPI,C1R,C8A,CLCN5,CRYM,CSDE1,CSF2RB,CSNK1G1,CSNK1G2,CSTF2,CTCF,CUL1,CYP2E1,DAPK2,DCXR,DGKA,DOK2,DPP4,DSG2,EP300,EPHA3,EPS8L2,ERBB4,ESR1,F2,FAS,FCGR2A,FLNB,FN1,G6PD,GAD2,GALNT10,GART,GRK2,GRK6,GTF2I,HADH,HASPIN,HAT1,HDX,HIBCH,HLA,DRB1,HMBOX1,HMGB3,HMGCS1,HMOX1,HNRNPA2B1,HSD17B10,HSD17B4,HSP90AB1,IDE,IFNGR1,IGF1R,ITGB3,ITPK1,ITSN1,KMT5A,KYNU,LRBA,MAGEA4,MAGI2,MAP3K3,MARF1,MATK,MMP2,MSH2,MSN,MTAP,MUC1,MYBPC1,MYBPC3,NBEA,NCBP1,NCOA3,NF2,NFATC1,NME3,NMT1,NOTCH1,NR1I3,NSUN5,NUP214,ODC1,OXSR1,PAFAH1B2,PAH,PANK1,PAPSS1,PARN,PATJ,PC,PCYT2,PDHA1,PDK4,PGC,PGK1,PHF24,PIP4K2C,POT1,POU2F1,PPARG,PPP2R1A,PRKCB,PSAP,PTK2B,PTPRE,PTPRF,PUS10,PYGL,RAC1,RARA,RARG,RBBP6,RBM39,RBP5,RGS6,RRM1,RTN4IP1,SART3,SEMA4D,SENP2,SERPINA5,SFMBT2,SH2B2,SH3YL1,SLC5A1,SLK,SMG5,SMURF2,SNX1,STARD13,SUB1,THBS2,TIMM9,TJP1,TLR4,TNFAIP8L2,USP19,UTRN,VAV3,VEGFB,VPS26A,WEE1,WHRN,XIAP,XRCC6,YWHAQ,ZBTB43,ZCWPW1,ZEB2,ZFP28,ZIC3,ZNF292,ZNF462

404	Stage IIIA cancer	Cancer,Organismal Injury and Abnormalities	1.37E,05	8	ALB,ALK,ERBB4,ESR1,GART,GGPS1,HSP90AB1,RRM1
405	Colony formation of colorectal cancer cell lines	Cellular Development,Cellular Growth and Proliferation	1.38E,05	7	IFNG,NOTCH1,PDK4,PPP2R1A,RARG,VEGFB,XIAP
406	Bile duct adenocarcinoma	Cancer,Gastrointestinal Disease,Hepatic System Disease,Organismal Injury and Abnormalities	1.38E,05	16	ACY1,ADH1C,ALB,ALK,EPS8L2,FAP,GC,HPD,HSP90AB1,MMP2,NOTCH1,PGK1,POU2F1,RRM1,SH2B2,TLR4
407	Gastrointestinal carcinoma	Cancer,Gastrointestinal Disease,Organismal Injury and Abnormalities	1.38E,05	230	ACACB,ACADSB,ACADVL,ACSM2A,ACVR2B,ACY1,ADH7,ALB,ALK,ALOX12,ANXA6,AOC3,APEX1,APPL1,AR,ARF6,ARHGAP5,ARHGEF1,ASAP1,ATP7B,ATXN3,B2M,B3GAT2,BCR,BMP1,BPI,BTK,BTRC,C1R,C8A,CAMK4,CANT1,CD1A,CD74,CDK5R1,CHKA,CLCN5,CSDE1,CSF2RB,CSNK1G1,CSNK1G2,CSTF2,CTCF,CUL1,CYP17A1,CYP2E1,CYP2R1,CYP7A1,DAPK2,DCXR,DFFA,DGKA,DHPS,DMC1,DOK2,DPP4,DSG2,ECHS1,EP300,EPHA3,EPS8L2,ERBB4,ESR1,ETF1,EXOSC9,F2,F3,FABP7,FAP,FAS,FHIT,FLNB,FN1,G6PD,GAD2,GALM,GALNT10,GART,GRHPR,GRK2,GRK6,GTF2I,HADH,HASPIN,HBB,HCK,HDAC7,HDX,HIBCH,HK2,HLA,DRB1,HMGB3,HMOX1,HNRNPA2B1,HPD,HSCB,HSD17B10,HSD17B4,HSP90AB1,HTR1A,IDE,IFNGR1,IGF1R,IMPA2,IMPDH1,IQUB,ITGB3,ITPK1,ITPKC,ITSN1,KIF11,KLK1,KMT5A,KYNU,LRBA,MAGEA4,MAGI2,MAP3K3,MAPK6,MARF1,MARK1,MATK,MECR,MMP2,MSH2,MSN,MTAP,MUC1,MYBPC1,MYBPC3,NAE1,NBEA,NCBP1,NCOA3,NF2,NFATC1,NMRK1,NMT1,NOTCH1,NSUN5,NT5C3A,NUP214,OAT,ODC1,OXSR1,PA2G4,PAFAH1B2,PAH,PANK1,PAPSS1,PARN,PARP3,PATJ,PC,PCYT2,PDHA1,PDK2,PDK3,PDK4,PEX5,PGC,PGK1,PHF24,POT1,POU2F1,PPARG,PPP2R1A,PRKCB,PTK2B,PTPRE,PTPRF,PUS10,PYGL,RAB26,RAC1,RARA,RARG,RBBP6,RBM39,RGS6,RND1,RRM1,RTN4IP1,RUVBL1,SART3,SEC23A,SEMA4D,SERPINA5,SF3B6,SFMBT2,SH2B2,SH3YL1,SKP1,SLC5A1,SLK,SMAP1,SMG5,SMG7,SMTN,SMURF2,SNX1,SPEF1,STARD13,STK16,SULT1A1,SYT13,TAF13,THBS2,TJP1,TLR4,TRIM21,UEVLD,USP19,UTRN,VAV3,VEGFB,VPS26A,WEE1,WHRN,WWP1,XRCC6,YWHAH,YY1,ZBTB43,ZCWPW1,ZEB2,ZFP28,ZIC3,ZNF292,ZNF462
408	Adult T,cell leukemia/lymphoma	Cancer,Hematological Disease,Immunological Disease,Organismal Injury and Abnormalities	1.41E,05	9	B2M,DPP4,EP300,FAS,FHIT,HNRNPA2B1,NOTCH1,POT1,PRKCB
409	Cell viability of carcinoma cell lines	Cell Death and Survival	1.41E,05	15	ALK,APEX1,FHIT,FN1,HBEGF,HCK,HK2,HMOX1,IGF1R,KIF11,MSH2,NOTCH1,PTK2B,RRM1,SFN
410	Stage IV secondary tumor	Cancer,Organismal Injury and Abnormalities	1.49E,05	11	ALB,ALK,AR,ERBB4,ESR1,GART,HSP90AB1,IGF1R,PARP3,PTK2B,RRM1
411	Apoptosis of colorectal cancer cell lines	Cell Death and Survival	1.50E,05	17	BTK,DAPK2,DFFA,DSG2,DTYMK,EP300,FAS,IFNG,MUC1,NOTCH1,PPARG,RAC1,SFN,SMURF2,SNX1,TLR4,XIAP
412	Stage III,IV breast cancer	Cancer,Organismal Injury and Abnormalities,Reproductive System Disease	1.50E,05	10	ALB,AR,ERBB4,ESR1,FN1,GGPS1,HMOX1,HSP90AB1,PARP3,RRM1
413	Metastatic gastrointestinal carcinoma	Cancer,Gastrointestinal Disease,Organismal Injury and Abnormalities	1.50E,05	10	ALB,ALK,ERBB4,FAS,GART,HSP90AB1,MMP2,PARP3,RAC1,RRM1
414	Muscle tumor	Cancer,Organismal Injury and Abnormalities,Skeletal and Muscular Disorders	1.51E,05	31	ACACB,ALK,AR,CLCN5,CSF2RB,CYP7A1,ERBB4,ESR1,FAP,FAS,FN1,HSD17B10,HSP90AB1,IGF1R,KMT5A,MDH1,MMP2,MSH2,NOTCH1,PDK4,PPARG,PRKCB,RRM1,S100A6,SEC23A,SLK,TAF13,TJP1,TLR4,UTRN,WEE1
415	Stage I epithelial cancer	Cancer,Organismal Injury and Abnormalities	1.52E,05	9	ALK,B2M,ERBB4,FN1,GART,HSP90AB1,MUC1,PGC,RRM1

416	Replication of virus	Infectious Diseases	1.53E,05	24	B2M,CANT1,CLK1,CYP17A1,CYP2E1,DAPK2,ESR1,G6PD,GRK2,GRK6,HCK,IFNG,KIF11,MDH1,MSH2,NCOA3,NUP214,OXSR1,PTK2B,SERPINA5,SF3B6,TLR4,TRIM21,ZEB2
417	Ruffling of plasma membrane	Cell Morphology	1.55E,05	4	ARF6,HMOX1,IQUB,NF2
418	Stage IIIB locally advanced non,small cell lung cancer	Cancer,Organismal Injury and Abnormalities,Respiratory Disease	1.61E,05	6	ALK,ERBB4,GART,HSP90AB1,IGF1R,RRM1
419	Advanced nonsquamous non,small cell lung carcinoma	Cancer,Organismal Injury and Abnormalities,Respiratory Disease	1.64E,05	9	ALB,ALK,ERBB4,GART,HSP90AB1,IGF1R,PARP3,PTK2B,RRM1
420	Transformation of tumor cell lines	Cancer	1.65E,05	8	ARF6,ERBB4,HNRNPA2B1,IGF1R,MATK,RAC1,SFN,STARD13
421	Serous neoplasm	Cancer,Organismal Injury and Abnormalities	1.66E,05	40	ALB,ALK,AOC3,AR,ARF6,B2M,BCR,CA8,CSF2RB,CTCF,DPP4,EP300,EPHA3,F2,F3,GART,HBB,HCK,HDAC7,HSP90AB1,HTR1A,ITSN1,KIF11,MAGEA4,MAP3K3,MUC1,NF2,NOTCH1,NT5C3A,PARP3,PDK4,PGC,PPP2R1A,PYGL,RRM1,SKP1,SMTN,THBS2,TLR4,VAV3
422	Familial cardiovascular disease	Cardiovascular Disease,Hereditary Disorder,Organismal Injury and Abnormalities	1.68E,05	24	ACADVL,ACVR2B,B3GAT3,CA8,DPP4,DSG2,ECI2,F2,FAS,HTR1A,IMPDH1,ITGB3,ITPKC,KYNU,MYBPC3,NOTCH1,PPARG,RRM1,STK4,TIMM9,TJP1,TTR,WWP1,ZIC3
423	Adhesion of tumor cell lines	Cell,To,Cell Signaling and Interaction	1.71E,05	18	ARHGEF1,DSG2,F2,FN1,GRK6,IFNG,IGF1R,ITGB3,MMP2,MUC1,NOTCH1,PPARG,PTK2B,RAC1,SNX1,TJP1,UTRN,ZEB2
424	Proliferation of blood cells	Cellular Development,Cellular Growth and Proliferation	1.75E,05	19	ACP3,ARF6,BCR,CD74,CSF2RB,DGKA,DPP4,FAS,FN1,HLA,DRB1,HMOX1,HNRNPA2B1,IFNG,IGF1R,ITGB3,MSH2,MSN,SEMA4D,VAV3
425	X,linked hereditary disease	Hereditary Disorder,Organismal Injury and Abnormalities	1.77E,05	24	AR,BMP1,BTK,CD74,CLCN5,CYP17A1,DAPK2,ESR1,FCGR2A,G6PD,HLA,DRB1,HMGB3,HSD17B10,MSN,PDHA1,PDK3,PGK1,TAF13,TLR4,UTRN,WHRN,XIAP,ZIC3,ZNF462
426	Neuromuscular disease	Neurological Disease,Skeletal and Muscular Disorders	1.90E,05	37	ADH1C,ALB,ARHGEF1,BCR,BTK,CD74,CDK5R1,CRYM,ESR1,F3,FAS,FCGR2A,FHIT,GGPS1,HADH,HDAC7,HLA,DRB1,HMOX1,HTR1A,IFNG,IMPDH1,ITGB3,MDH1,NBEA,NDUFAB1,PGK1,PPARG,PRKCB,PSAP,PTK2B,PTPRE,RRM1,SMTN,SUB1,TLR4,XIAP,XRCC6
427	Metastatic squamous cell carcinoma	Cancer,Organismal Injury and Abnormalities	1.91E,05	9	ALK,ERBB4,F3,GART,HSP90AB1,IGF1R,PARP3,PTK2B,RRM1
428	Rheumatoid arthritis	Connective Tissue Disorders,Immunological Disease,Inflammatory Disease,Inflammatory Response,Organismal Injury and Abnormalities,Skeletal and Muscular Disorders	1.95E,05	32	ALB,ALOX12,ANG,ASAP1,B2M,CD1A,CD74,CSF2RB,DPP4,FCGR2A,FN1,HCK,HDAC7,HLA,DRB1,HMOX1,IDE,IFNG,IMPDH1,MMP1,MMP2,MTAP,PGK1,PPARG,PTK2B,PTPRE,RGS6,SF3B6,SMG7,TAF13,TJP1,TLR4,XIAP
429	Quantity of cells	Tissue Morphology	1.95E,05	22	ALK,AOC3,AR,ARF6,ATXN3,ESR1,FAS,FN1,GAD2,HBB,HMOX1,IFNG,IFNGR1,IGF1R,NOTCH1,PPARG,RAC1,SFN,THBS2,VAV3,VEGFB,XIAP
430	Cell spreading of blood cells	Cell Morphology,Cellular Movement	1.96E,05	5	ALB,FCGR2A,FN1,PTK2B,RAC1
431	Locally advanced malignant tumor	Cancer,Organismal Injury and Abnormalities	1.98E,05	11	ALK,AR,ERBB4,ESR1,GART,GGPS1,HSP90AB1,IGF1R,PARP3,PTK2B,RRM1

432	Cytostasis of tumor cell lines	Cellular Growth and Proliferation	1.98E,05	11	ALK,ESR1,FABP7,FHIT,GRK2,IFNG,IGF1R,NF2,NME3,NOTCH1,RARG
433	Proliferation of lung cancer cell lines	Cellular Development,Cellular Growth and Proliferation	2.04E,05	19	AASDHPPT,ALK,CSTF2,DOK2,F3,FHIT,HK2,HMOX1,HNRNPA2B1,IFNG,IGF1R,MMP1,MUC1,NCOA3,NOTCH1,PDK4,PPARG,PRKCB,RAC1
434	Movement Disorders	Neurological Disease	2.08E,05	38	ADH1C,ALB,AR,ARHGEF1,ATP7B,ATXN3,BCR,CA8,CD74,CDK5R1,CRYM,DHPS,ESR1,F3,FAS,FCGR2A,HADH,HMOX1,HTR1A,IFNG,IFNGR1,MDH1,MECR,MYBPC1,NBEA,NDUFAB1,PGK1,PPARG,PRKCB,PSAP,PTK2B,PTPRE,RTN4IP1,SMTN,SUB1,TLR4,XIAP,XRCC6
435	Aggregation of blood platelets	Cell,To,Cell Signaling and Interaction,Hematological System Development and Function,Inflammatory Response	2.15E,05	8	ALB,ALOX12,BTK,CANT1,F2,FCGR2A,ITGB3,TLR4
436	Binding of monocytes	Cell,To,Cell Signaling and Interaction,Hematological System Development and Function,Inflammatory Response	2.15E,05	6	BPI,F2,FN1,ITGB3,PPARG,RAC1
437	Adhesion of PBMCs	Cell,To,Cell Signaling and Interaction,Hematological System Development and Function,Immune Cell Trafficking,Inflammatory Response	2.22E,05	4	F2,F3,IFNG,PPARG
438	Apoptosis of leukemia cell lines	Cell Death and Survival	2.25E,05	17	ALK,B2M,CHKA,CSF2RB,DFFA,DPP4,FAS,FN1,HCK,HMOX1,MSN,MUC1,NOTCH1,PRKCB,RAC1,STK4,XIAP
439	Cell death of leukemia cell lines	Cell Death and Survival	2.29E,05	19	ALK,B2M,CHKA,CSF2RB,DFFA,DPP4,FAS,FN1,HCK,HMOX1,IFNG,MSN,MUC1,NOTCH1,PRKCB,RAC1,STK4,WEE1,XIAP
440	Stage IIIC cancer	Cancer,Organismal Injury and Abnormalities	2.34E,05	8	ALB,ESR1,FN1,GART,GGPS1,HSP90AB1,MUC1,RRM1
441	Recurrent non,small cell lung cancer	Cancer,Organismal Injury and Abnormalities,Respiratory Disease	2.34E,05	8	ALB,ALK,ERBB4,GART,HSP90AB1,IGF1R,PTK2B,RRM1
442	Invasive breast cancer	Cancer,Organismal Injury and Abnormalities,Reproductive System Disease	2.34E,05	18	ALB,AR,CSF2RB,ERBB4,ESR1,FAP,FAS,FN1,HSP90AB1,MMP2,MYBPC3,PARP3,PDK4,PTPRE,PTPRF,RBM39,RRM1,YWHAQ
443	Vascular tumor	Cancer,Cardiovascular Disease,Organismal Injury and Abnormalities	2.35E,05	15	ALK,AR,BCR,CSF2RB,ESR1,HSP90AB1,IGF1R,MSH2,MSN,NOTCH1,PPARG,RARA,RARG,TJP1,ZEB2
444	Glycolysis of cells	Carbohydrate Metabolism	2.35E,05	10	ESR1,HK2,IGF1R,ITGB3,NCOA3,NUP214,PDHA1,PDK4,PGK1,SENP2

445	Cell movement of neutrophils	Cellular Movement,Hematological System Development and Function,Immune Cell Trafficking,Inflammatory Response	2.35E,05	10	ALB,DAPK2,DPP4,FAS,FN1,IFNG,ITGB3,PRKCB,RAC1,TLR4
446	Stage IV epithelial cancer	Cancer,Organismal Injury and Abnormalities	2.35E,05	10	ALB,ALK,ERBB4,ESR1,GART,HSP90AB1,IGF1R,PARP3,PTK2B,RRM1
447	Progressive metastatic carcinoma	Cancer,Organismal Injury and Abnormalities,Tumor Morphology	2.37E,05	9	ALK,ERBB4,ESR1,F3,GART,HSP90AB1,IGF1R,PTK2B,RRM1
448	Quantity of tumor cell lines	Tissue Morphology	2.38E,05	14	ALK,AR,ARF6,ESR1,FAS,FN1,HMOX1,IFNG,IGF1R,NOTCH1,RAC1,SFN,VAV3,VEGFB
449	Acute leukemia	Cancer,Hematological Disease,Immunological Disease,Organismal Injury and Abnormalities	2.44E,05	66	ALB,ALK,AMY1C (includes others),APEX1,ARHGAP5,BCR,BTK,BTRC,C8A,CANT1,CD1A,CD74,CDK5R1,CIAO1,CSF2RB,CTCF,CYP2E1,CYP2R1,EP300,EPHA3,ESR1,ETF1,F2,F3,FAP,FAS,GRK2,HASPIN,HCK,HLA,DRB1,HSP90AB1,IFNG,IGF1R,IMPDH1,ITGB3,KLK1,KMT5A,LRBA,MAGEA4,MSH2,NCOA3,NOTCH1,NT5C3A,NUP214,PA2G4,PDHA1,PDK3,PGC,PTK2B,PYGL,RAC1,RARA,RARG,RRM1,SH2B2,SMG5,SMG7,SMTN,SYT13,TJP1,TLR4,UEVLD,VEGFB,WEE1,XIAP,ZEB2
450	Proliferation of cancer cells	Cancer,Cellular Development,Cellular Growth and Proliferation,Organismal Injury and Abnormalities,Tumor Morphology	2.46E,05	12	AR,BTK,ESR1,FHIT,IFNG,IGF1R,MUC1,NOTCH1,ODC1,PTK2B,RUVBL1,XIAP
451	TP53 mutation negative malignant solid tumor	Cancer,Organismal Injury and Abnormalities	2.48E,05	3	AR,BTK,CYP17A1
452	Arrest in G0/G1 phase transition of carcinoma cell lines	Cell Cycle	2.48E,05	3	AASDHPPT,FHIT,IFNG
453	Benign facial lentiginoses	Dermatological Diseases and Conditions,Organismal Injury and Abnormalities	2.48E,05	3	ODC1,RARA,RARG
454	Mottled facial hypopigmentation	Dermatological Diseases and Conditions,Organismal Injury and Abnormalities	2.48E,05	3	ODC1,RARA,RARG
455	Mottled facial hyperpigmentation	Dermatological Diseases and Conditions,Organismal Injury and Abnormalities	2.48E,05	3	ODC1,RARA,RARG
456	Efflux of L,triiodothyronine	Amino Acid Metabolism,Molecular Transport,Small Molecule Biochemistry	2.48E,05	3	ALB,CRYM,TTR

457	Ovarian serous tumor	Cancer,Endocrine System Disorders,Organismal Injury and Abnormalities,Reproductive System Disease	2.48E,05	34	ALB,ALK,AR,ARF6,B2M,BCR,CA8,CSF2RB,CTCF,DPP4,EP300,EPHA3,F2,GART,HCK,HSP90AB1,HTR1A,ITSN1,MAGEA4,MAP3K3,MUC1,NF2,NOTCH1,NT5C3A,PARP3,PDK4,PGC,PPP2R1A,PYGL,RRM1,SKP1,THBS2,TLR4,VAV3
458	Hematopoiesis of mononuclear leukocytes	Cellular Development,Cellular Growth and Proliferation,Hematological System Development and Function,Hematopoiesis,Lymphoid Tissue Structure and Development,Tissue Development	2.50E,05	13	BTK,CD74,CSF2RB,FAS,HLA,DRB1,IFNG,ITGB3,MSN,NOTCH1,PPARG,RAC1,RARA,TLR4
459	Biliary tract adenocarcinoma	Cancer,Gastrointestinal Disease,Hepatic System Disease,Organismal Injury and Abnormalities	2.57E,05	17	ACY1,ADH1C,ALB,ALK,BCR,EPS8L2,FAP,GC,HPD,HSP90AB1,MMP2,NOTCH1,PGK1,POU2F1,RRM1,SH2B2,TLR4
460	Graft,vs,host disease	Immunological Disease	2.65E,05	7	BTK,CSF2RB,DPP4,FCGR2A,FN1,IMPDH1,RRM1
461	Cell death of stomach cancer cell lines	Cell Death and Survival	2.77E,05	8	ALB,ALOX12,CD74,FAS,HK2,IGF1R,PRKCB,TLR4
462	Stage III non,small cell lung cancer	Cancer,Organismal Injury and Abnormalities,Respiratory Disease	2.77E,05	8	ALB,ALK,ERBB4,GART,HSP90AB1,IGF1R,PARP3,RRM1
463	Secretion of molecule	Molecular Transport	2.80E,05	14	ANG,ARF6,CD74,CYP2E1,F2,FCGR2A,FN1,GAD2,IFNG,IGF1R,PPARG,PRKCB,STK4,USP19
464	Anaplastic carcinoma	Cancer,Organismal Injury and Abnormalities	2.84E,05	24	ALK,AMY1C (includes others),AR,DSG2,EPHA3,ERBB4,FN1,GALNT10,GART,GRK2,HASPIN,HSP90AB1,IGF1R,MMP1,MSH2,PARP3,PGK1,PTK2B,RRM1,SLK,STK4,TLR4,YY1,ZEB2
465	Transitional,cell carcinoma	Cancer,Organismal Injury and Abnormalities	2.91E,05	18	AR,CTCF,EP300,EPHA3,ERBB4,FN1,GART,HSP90AB1,MMP2,NCOA3,NF2,NOTCH1,PARP3,PPP2R1A,PRKCB,RARA,RRM1,TLR4
466	Aggregation of blood cells	Cell,To,Cell Signaling and Interaction,Hematological System Development and Function	2.93E,05	9	ALB,ALOX12,BTK,CANT1,F2,FCGR2A,ITGB3,SEMA4D,TLR4
467	Sarcoma	Cancer,Organismal Injury and Abnormalities	2.93E,05	47	ACACB,ALK,ANG,AR,CSF2RB,CYP7A1,DHPS,ERBB4,ESR1,F2,FABP7,FAP,FN1,GGPS1,HMGCS1,HSD17B4,HSP90AB1,IGF1R,KMT5A,MAPK6,MMP1,MMP2,MSH2,NAE1,NBEA,NCBP1,NF2,NOTCH1,PARP3,PDK4,PEX5,PGK1,PHF24,PPARG,PPP2R1A,PRKCB,RARA,RARG,RRM1,SEC23A,SEMA4D,SLK,TJP1,TLR4,UTRN,WWP1,ZIC3
468	Permeability of vascular system	Cardiovascular System Development and Function,Tissue Morphology	2.94E,05	7	ARF6,F2,IFNG,IQUB,MMP2,PTK2B,RAC1
469	Blastoma	Cancer,Organismal Injury and Abnormalities	2.98E,05	23	CSF2RB,EP300,EPHA3,ERBB4,FABP7,FN1,GART,HNRNPA2B1,HSP90AB1,IDE,IFNG,ITGB3,MSH2,NBEA,PA2G4,PRKCB,RAC1,RRM1,TAF13,THBS2,TLR4,VAV3,ZEB2

470	Thrombus	Cardiovascular Disease,Hematological Disease,Organismal Injury and Abnormalities	3.00E,05	10	ALOX12,AR,ESR1,F2,F3,ITGB3,MMP2,RRM1,SERPINA5,VEGFB
471	Interphase of lung cancer cell lines	Cell Cycle	3.00E,05	8	AASDHPPT,BTRC,EP300,FHIT,HMOX1,IFNG,NOTCH1,SART3
472	Concentration of lipid	Lipid Metabolism,Molecular Transport,Small Molecule Biochemistry	3.05E,05	15	ACACB,ALB,ARF6,BCR,CERT1,CHKA,CYP7A1,EP300,F2,FAS,HMOX1,IFNG,PPARG,PSAP,RAC1
473	Amyloidosis	Metabolic Disease,Organismal Injury and Abnormalities	3.05E,05	29	ALB,AR,B2M,C1R,CD74,CDK5R1,ESR1,F3,FCGR2A,GAD2,GC,GGPS1,HMOX1,HNRNPA2B1,HTR1A,IDE,MMP1,MMP2,NAE1,NOTCH1,PPARG,PRKCB,PSAP,PTPRE,SMTN,TAF13,TLR4,TTR,XIAP
474	Cell death of immune cells	Cell Death and Survival	3.14E,05	16	BTK,CAMK4,FAS,FN1,HBB,IFNG,NFATC1,NOTCH1,PPARG,PRKCB,RAC1,SEMA4D,TLR4,VAV3,XIAP,YY1
475	Endometrium tumor	Cancer,Organismal Injury and Abnormalities,Reproductive System Disease	3.17E,05	129	AASDHPPT,ACACB,ACADSB,ACBD7,ACY1,ALB,ALK,AOC3,APPL1,AR,ATP7B,B2M,B3GAT2,BCR,BTK,C8A,CA8,CD1A,CLCN5,CSDE1,CSF2RB,CSTF2,CTCF,CUL1,CYP7A1,DCTD,DHPS,DOK2,DPP4,DTYMK,EP300,EPHA3,ERBB4,ESR1,ETF1,FABP7,FAP,FCGR2A,FLNB,FN1,G6PD,GAD2,GRK2,GTF2I,HBB,HDAC7,HDX,HMBOX1,HMGB3,HPD,HSD17B4,HSP90AB1,HTR1A,IGF1R,IQUB,ITGB3,ITPKC,KIF11,LRBA,MAGEA4,MAGI2,MAP3K3,MAPK6,MARF1,MARK1,MDH1,MEMO1,MMP1,MMP2,MSH2,MSN,MYBPC3,MYSM1,NAE1,NBEA,NCBP1,NF2,NFATC1,NMRK1,NMT1,NOTCH1,NT5C3A,NUP214,OAT,PANK1,PARN,PARP3,PATJ,PDK3,PGK1,PPP2R1A,PTPRE,PTPRF,RARA,RARG,RBBP6,RBM39,RGS6,RND1,RUVBL1,SART3,SENP2,SF3B6,SH3YL1,SLC5A1,SLK,SMG5,SMTN,SMURF2,SNX1,STK4,SUB1,SULT1A1,SYT13,THBS2,TJP1,TLR4,TRIM21,UEVLD,UTRN,VAV3,WWP1,XIAP,YY1,ZEB2,ZFP28,ZIC3,ZNF292,ZNF462
476	Recurrent advanced non,small cell lung cancer	Cancer,Organismal Injury and Abnormalities,Respiratory Disease	3.23E,05	6	ALK,ERBB4,GART,HSP90AB1,PTK2B,RRM1
477	Evans stage IVS neuroblastoma	Cancer,Neurological Disease,Organismal Injury and Abnormalities	3.23E,05	6	ALK,CSF2RB,FCGR2A,HSP90AB1,RARA,RARG
478	Serous adenocarcinoma	Cancer,Organismal Injury and Abnormalities	3.27E,05	39	ALB,ALK,AOC3,AR,ARF6,B2M,BCR,CA8,CSF2RB,CTCF,DPP4,EP300,EPHA3,F2,F3,GART,HBB,HCK,HSP90AB1,HTR1A,ITSN1,KIF11,MAGEA4,MAP3K3,MUC1,NF2,NOTCH1,NT5C3A,PARP3,PDK4,PGC,PPP2R1A,PYGL,RRM1,SKP1,SMTN,THBS2,TLR4,VAV3
479	Formation of cytoskeleton	Cellular Assembly and Organization	3.28E,05	15	AR,ARF6,F2,FCGR2A,FHIT,FN1,HCK,ITGB3,NF2,NFATC1,NOTCH1,PTK2B,RAC1,STK4,XIAP
480	Advanced,stage chronic kidney disease	Organismal Injury and Abnormalities,Renal and Urological Disease	3.36E,05	9	AR,F2,FCGR2A,IMPDH1,PPARG,PRKCB,SLC5A1,SMURF2,TTR
481	Grade 3,4 glioma	Cancer,Neurological Disease,Organismal Injury and Abnormalities	3.36E,05	151	ACACB,ACADVL,ACVR2B,ADH7,ALB,ALK,ALOX12,AMY1C (includes others),APEX1,APPL1,ARHGAP5,ARHGEF1,ASAP1,ATP7B,ATXN3,B3GAT2,BMP1,BPI,C1R,C8A,CLCN5,CSDE1,CSF2RB,CSNK1G2,CSTF2,CTCF,CUL1,CYP2E1,DAPK2,DCXR,DGKA,DOK2,DPP4,DSG2,EP300,EPHA3,EPS8L2,ERBB4,ESR1,F2,FABP7,FAS,FCGR2A,FLNB,FN1,G6PD,GAD2,GALNT10,GART,GRK2,GRK6,GTF2I,HADH,HASPIN,HDX,HIBCH,HLA,DRB1,HMBOX1,HMGCS1,HMOX1,HSD17B10,HSD17B4,HSP90AB1,IDE,IFNGR1,IGF1R,ITGB3,ITSN1,KMT5A,KYNU,LRBA,MAGI2,MAP3K3,MARF1,MATK,MSH2,MSN,MTAP,MUC1,MYBPC3,NBEA,NCBP1,NCOA3,NF2,NFATC1,NME3,NMT1,NOTCH1,NSUN5,NUP214,ODC1,OXSR1,PAFAH1B2,PAH,PANK1,PAPSS1,PARN,PATJ,PC,PCYT2,PDHA1,PGC,PGK1,PHF24,POU2F1,PPARG,PPP2R1A,PSAP,PTK2B,PUS10,PYGL,RARA,RARG,RBBP6,RBM39,RBP5,RGS6,RRM1,RTN4IP1,SART3,SEMA4D,SENP2,SERPINA5,SFMBT2,SH2B2,SH3YL1,SLC5A1,SLK,SMG5,SMURF2,SNX1,STARD13,SUB1,THBS2,TJP1,TLR4,TNFAIP8L2,USP19,UTRN,VAV3,VPS26A,WEE1,WHRN,XRCC6,ZBTB43,ZCWPW1,ZEB2,ZFP28,ZIC3,ZNF292,ZNF462

482	Granuloma	Inflammatory Disease,Organismal Injury and Abnormalities	3.39E,05	11	AMY1C (includes others),G6PD,GRHPR,HBEGF,HLA,DRB1,IFNG,IFNGR1,IMPDH1,KLK1,PTPRE,TAF13
483	Gastrointestinal adenocarcinoma	Cancer,Gastrointestinal Disease,Organismal Injury and Abnormalities	3.57E,05	220	ACACB,ACADSB,ACADVL,ACSM2A,ACVR2B,ACY1,ADH7,ALB,ALK,ALOX12,ANXA6,AOC3,APEX1,APPL1,AR,ARF6,ARHGAP5,ARHGEF1,ASAP1,ATP7B,ATXN3,B2M,B3GAT2,BCR,BMP1,BPI,BTK,BTRC,C1R,C8A,CAMK4,CANT1,CD1A,CD74,CDK5R1,CHKA,CLCN5,CSDE1,CSF2RB,CSNK1G1,CSNK1G2,CSTF2,CTCF,CUL1,CYP17A1,CYP2E1,CYP7A1,DAPK2,DCXR,DFFA,DGKA,DHPS,DMC1,DOK2,DPP4,DSG2,ECHS1,EP300,EPHA3,EPS8L2,ERBB4,ESR1,ETF1,EXOSC9,F2,F3,FAP,FAS,FHIT,FLNB,FN1,G6PD,GAD2,GALNT10,GART,GRHPR,GRK2,GRK6,GTF2I,HADH,HASPIN,HCK,HDAC7,HDX,HIBCH,HK2,HLA,DRB1,HMGB3,HMOX1,HNRNPA2B1,HPD,HSCB,HSD17B10,HSD17B4,HSP90AB1,HTR1A,IDE,IFNGR1,IGF1R,IMPA2,IMPDH1,IQUB,ITGB3,ITPK1,ITPKC,ITSN1,KIF11,KMT5A,KYNU,LRBA,MAGEA4,MAGI2,MAP3K3,MAPK6,MARF1,MARK1,MATK,MECR,MMP2,MSH2,MSN,MTAP,MUC1,MYBPC1,MYBPC3,NAE1,NBEA,NCBP1,NCOA3,NF2,NFATC1,NMRK1,NMT1,NOTCH1,NSUN5,NT5C3A,NUP214,OAT,ODC1,OXSR1,PAFAH1B2,PAH,PANK1,PARN,PARP3,PATJ,PC,PCYT2,PDHA1,PDK2,PDK3,PDK4,PGC,PGK1,PHF24,POT1,POU2F1,PPARG,PPP2R1A,PRKCB,PTK2B,PTPRE,PTPRF,PUS10,PYGL,RAB26,RAC1,RARA,RARG,RBBP6,RBM39,RGS6,RND1,RRM1,RTN4IP1,RUVBL1,SART3,SEC23A,SEMA4D,SERPINA5,SF3B6,SFMBT2,SH2B2,SH3YL1,SKP1,SLC5A1,SLK,SMAP1,SMG5,SMG7,SMTN,SMURF2,SNX1,SPEF1,STARD13,STK16,SULT1A1,SYT13,THBS2,TJP1,TLR4,TRIM21,UEVLD,USP19,UTRN,VAV3,VEGFB,VPS26A,WHRN,WWP1,XRCC6,YWHAH,YY1,ZBTB43,ZCWPW1,ZEB2,ZFP28,ZIC3,ZNF292,ZNF462
484	Unresectable advanced non,small cell lung carcinoma	Cancer,Organismal Injury and Abnormalities,Respiratory Disease	3.59E,05	7	ALK,ERBB4,GART,HSP90AB1,IGF1R,PARP3,PTK2B
485	Advanced squamous cell cancer	Cancer,Organismal Injury and Abnormalities	3.59E,05	9	ALK,ERBB4,F3,GART,HSP90AB1,IGF1R,PARP3,PTK2B,RRM1
486	Stage IV metastatic solid tumor	Cancer,Organismal Injury and Abnormalities	3.59E,05	9	ALK,ERBB4,ESR1,GART,HSP90AB1,IGF1R,PARP3,PTK2B,RRM1
487	Malignant neoplasm of large intestine	Cancer,Gastrointestinal Disease,Organismal Injury and Abnormalities	3.59E,05	223	ACACB,ACADSB,ACADVL,ACSM2A,ACVR2B,ACY1,ADH7,ALB,ALK,ALOX12,ANXA6,AOC3,APEX1,APPL1,AR,ARF6,ARHGAP5,ARHGEF1,ASAP1,ATP7B,ATXN3,B2M,B3GAT2,BCR,BMP1,BPI,BTK,BTRC,C1R,C8A,CAMK4,CANT1,CD1A,CD74,CDK5R1,CHKA,CLCN5,CSDE1,CSF2RB,CSNK1G2,CSTF2,CTCF,CUL1,CYP17A1,CYP2E1,CYP7A1,DAPK2,DCXR,DFFA,DGKA,DHPS,DMC1,DOK2,DPP4,DSG2,ECHS1,EP300,EPHA3,EPS8L2,ERBB4,ESR1,ETF1,EXOSC9,F2,F3,FAP,FAS,FCGR2A,FHIT,FLNB,FN1,G6PD,GAD2,GALNT10,GART,GRHPR,GRK2,GRK6,GTF2I,HADH,HASPIN,HCK,HDAC7,HDX,HIBCH,HK2,HLA,DRB1,HMGB3,HMOX1,HNRNPA2B1,HPD,HSCB,HSD17B10,HSD17B4,HSP90AB1,HTR1A,IDE,IFNG,IFNGR1,IGF1R,IMPA2,IMPDH1,IQUB,ITGB3,ITPK1,ITPKC,ITSN1,KIF11,KMT5A,KYNU,LRBA,MAGEA4,MAGI2,MAP3K3,MAPK6,MARF1,MARK1,MATK,MECR,MMP2,MSH2,MSN,MTAP,MUC1,MYBPC1,MYBPC3,NAE1,NBEA,NCBP1,NCOA3,NF2,NFATC1,NMRK1,NMT1,NOTCH1,NSUN5,NT5C3A,NUP214,OAT,ODC1,OXSR1,PAFAH1B2,PANK1,PAPSS1,PARN,PARP3,PATJ,PC,PCYT2,PDHA1,PDK2,PDK3,PDK4,PEX5,PGC,PGK1,PHF24,POT1,POU2F1,PPARG,PPP2R1A,PRKCB,PTK2B,PTPRF,PUS10,PYGL,RAB26,RAC1,RARA,RARG,RBBP6,RBM39,RGS6,RND1,RRM1,RTN4IP1,RUVBL1,SART3,SEC23A,SEMA4D,SERPINA5,SF3B6,SFMBT2,SH2B2,SH3YL1,SKP1,SLC5A1,SLK,SMAP1,SMG5,SMG7,SMTN,SMURF2,SNX1,SPEF1,STARD13,STK16,STK4,SYT13,TAF13,THBS2,TJP1,TLR4,TRIM21,TTR,UEVLD,USP19,UTRN,VAV3,VEGFB,VPS26A,WHRN,WWP1,XRCC6,YWHAH,YY1,ZBTB43,ZCWPW1,ZEB2,ZFP28,ZIC3,ZNF292,ZNF462
488	BRCA1 mutation positive cancer	Cancer,Organismal Injury and Abnormalities	3.67E,05	6	ESR1,GART,HSP90AB1,PARP3,RARG,RRM1
489	Angioedema	Dermatological Diseases and Conditions,Immunologic	3.67E,05	6	AR,C1R,DPP4,ESR1,G6PD,TLR4

		al Disease,Organismal Injury and Abnormalities			
490	BRCA1 mutation positive solid tumor	Cancer,Organismal Injury and Abnormalities	3.67E,05	6	ESR1,GART,HSP90AB1,PARP3,RARG,RRM1
491	Cell transformation	Cancer	3.68E,05	13	ARF6,CHKA,EP300,ERBB4,HNRNPA2B1,IGF1R,MATK,NCOA3,NOTCH1,ODC1,RAC1,SFN,STARD13
492	Smooth muscle tumor	Cancer,Organismal Injury and Abnormalities,Skeletal and Muscular Disorders	3.70E,05	18	AR,CLCN5,ERBB4,ESR1,FAS,FN1,HSD17B10,HSP90AB1,IGF1R,MDH1,MMP2,PDK4,PPARG,RRM1,S100A6,TAF13,UTRN,WEE1
493	Synthesis of steroid	Lipid Metabolism,Small Molecule Biochemistry,Vitamin and Mineral Metabolism	3.76E,05	12	ACACB,ACAT2,ALOX12,CYP17A1,CYP2R1,CYP7A1,G6PD,GGPS1,HMGCS1,HSD17B10,HSD17B4,IFNG
494	Glioblastoma	Cancer,Neurological Disease,Organismal Injury and Abnormalities	3.77E,05	22	CSF2RB,EP300,EPHA3,ERBB4,FABP7,FN1,GART,HNRNPA2B1,HSP90AB1,IDE,IFNG,ITGB3,MSH2,NBEA,PA2G4,PRKCB,RAC1,RRM1,TAF13,TLR4,VAV3,ZEB2
495	Advanced breast cancer	Cancer,Organismal Injury and Abnormalities,Reproductive System Disease	3.79E,05	10	ALK,AR,CYP17A1,ERBB4,ESR1,GGPS1,HSP90AB1,MUC1,PARP3,RRM1
496	Alzheimer disease	Metabolic Disease,Neurological Disease,Organismal Injury and Abnormalities,Psychological Disorders	3.81E,05	28	ALB,AR,C1R,CD74,CDK5R1,ESR1,F3,FCGR2A,GAD2,GC,GGPS1,HMOX1,HNRNPA2B1,HTR1A,IDE,MMP1,MMP2,NAE1,NOTCH1,PPARG,PRKCB,PSAP,PTPRE,SMTN,TAF13,TLR4,TTR,XIAP
497	EGFR or ROS1 mutation positive non,small cell lung carcinoma	Cancer,Organismal Injury and Abnormalities,Respiratory Disease	3.82E,05	8	ALK,ERBB4,GART,HSP90AB1,IGF1R,MUC1,PTK2B,SFN
498	Metastatic squamous cell lung cancer	Cancer,Organismal Injury and Abnormalities,Respiratory Disease	3.82E,05	8	ALK,ERBB4,GART,HSP90AB1,IGF1R,PARP3,PTK2B,RRM1
499	Gastrointestinal tract cancer	Cancer,Gastrointestinal Disease,Organismal Injury and Abnormalities	3.87E,05	235	ACACB,ACADSB,ACADVL,ACSM2A,ACVR2B,ACY1,ADH7,ALB,ALK,ALOX12,ANXA6,AOC3,APEX1,APPL1,AR,ARF6,ARHGAP5,ARHGEF1,ASAP1,ATP7B,ATXN3,B2M,B3GAT2,BCR,BMP1,BPI,BTK,BTRC,C1R,C8A,CAMK4,CANT1,CD1A,CD74,CDK5R1,CHKA,CLCN5,CSDE1,CSF2RB,CSNK1G1,CSNK1G2,CSTF2,CTCF,CUL1,CYP17A1,CYP2E1,CYP2R1,CYP7A1,DAPK2,DCXR,DFFA,DGKA,DHPS,DMC1,DOK2,DPP4,DSG2,ECHS1,EP300,EPHA3,EPS8L2,ERBB4,ESR1,ETF1,EXOSC9,F2,F3,FABP7,FAP,FAS,FCGR2A,FHIT,FLNB,FN1,G6PD,GAD2,GALM,GALNT10,GART,GRHPR,GRK2,GRK6,GTF2I,HADH,HASPIN,HBB,HCK,HDAC7,HDX,HIBCH,HK2,HLA,DRB1,HMGB3,HMOX1,HNRNPA2B1,HPD,HSCB,HSD17B10,HSD17B4,HSP90AB1,HTR1A,IDE,IFNG,IFNGR1,IGF1R,IMPA2,IMPDH1,IQUB,ITGB3,ITPK1,ITPKC,ITSN1,KIF11,KLK1,KMT5A,KYNU,LRBA,MAGEA4,MAGI2,MAP3K3,MAPK6,MARF1,MARK1,MATK,MECR,MMP2,MSH2,MSN,MTAP,MUC1,MYBPC1,MYBPC3,NAE1,NBEA,NCBP1,NCOA3,NF2,NFATC1,NMRK1,NMT1,NOTCH1,NSUN5,NT5C3A,NUP214,OAT,ODC1,OXSR1,PA2G4,PAFAH1B2,PAH,PANK1,PAPSS1,PARN,PARP3,PATJ,PC,PCYT2,PDHA1,PDK2,PDK3,PDK4,PEX5,PGC,PGK1,PHF24,POT1,POU2F1,PPARG,PPP2R1A,PRKCB,PTK2B,PTPRE,PTPRF,PUS10,PYGL,RAB26,RAC1,RARA,RARG,RBBP6,RBM39,RGS6,RND1,RRM1,RTN4IP1,RUVBL1,SART3,SEC23A,SEMA4D,SERPINA5,SF3B6,SFMBT2,SFN,SH2B2,SH3YL1,SKP1,SLC5A1,SLK,SMAP1,SMG5,SMG7,SMTN,SMURF2,SNX1,SPEF1,STARD13,STK16,STK4,SULT1A1,SYT13,TAF13,THBS2,TJP1,TLR4,TRIM21,TTR,UEVLD,USP19,UTRN,VAV

					3,VEGFB,VPS26A,WEE1,WHRN,WWP1,XRCC6,YWHAH,YY1,ZBTB43,ZCWPW1,ZEB2,ZFP28,ZIC3,ZNF292, ZNF462
500	Mixed cell carcinoma	Cancer,Organismal Injury and Abnormalities	3.99E,05	39	ALK,AR,ARHGEF1,ASAP1,CLK1,CSNK1G1,CSTF2,DPP4,EPHA3,ERBB4,F3,GART,HBEGF,HNRNPA2B1,HSP 90AB1,ITPA,MAGI2,NBEA,NCOA3,NF2,NR1I3,ODC1,PARP3,PC,PDK4,POU2F1,PPARG,PPP2R1A,PTPRF,RB M39,RRM1,RTN4IP1,SFMBT2,SH3YL1,SMTN,SYT13,UTRN,WWP1,ZEB2

Supplementary Table S4: Upstream analysis by IPA for solasonine. The upstream analysis by IPA was performed to predict the upstream regulatory factors for potential target genes of solasonine. The prediction of upstream regulators were sorted by increasing of p-value (p-value ≤0.05).

Upstream Regulator	Molecule Type	p-value	Target Molecules in Dataset of Solasonine Related Genes
ERK	group	2.85E-07	CDK5R1,ESR1,FAS,HBEGF,IFNG,ITGB3,MMP1,MMP2,MUC1,ODC1,PDK4
EGFR	kinase	3.98E-06	AR,ESR1,F3,HK2,HNRNPA2B1,IFNG,MMP1,PTPRE,XIAP,ZEB2
ERK1/2	group	4.67E-06	B2M,CYP17A1,CYP7A1,ESR1,FN1,HBEGF,HK2,IGF1R,MMP1,MMP2,PTPRE
TNF	cytokine	8.89E-06	CD74,CYP17A1,DPP4,F3,FAS,FN1,HBEGF,HMOX1,IFNGR1,ITGB3,KYNU,MMP1,MMP2,MSH2,MUC1,NFATC1,NOTCH1,PPARG,TJP1,TLR4,XIAP
PCGEM1	other	2.58E-05	CANT1,G6PD,HK2,PDHA1,PDK4,PGK1
mir-122	microRNA	3.13E-05	CTCF,CYP7A1,G6PD,GALNT10,IGF1R,MAP3K3,MARK1,SEMA4D,VAV3,WARS1,YARS1
let-7	microRNA	3.51E-05	AR,FN1,HK2,IGF1R,ITGB3,MAPK6,PA2G4,STARD13,TLR4
SIRT1	transcription regulator	3.61E-05	EP300,FN1,HMOX1,MMP2,NMRK1,PDK4,RAC1
TWIST1	transcription regulator	4.98E-05	AR,FAS,FN1,MMP1,MMP2,ZEB2
WAC	other	5.41E-05	G6PD,PDK4,PPARG
SP1	transcription regulator	5.43E-05	AR,CYP17A1,ESR1,FN1,HBB,HBEGF,HK2,HMOX1,IFNGR1,IGF1R,ITGB3,MMP2,RARA,ZEB2
IFNG	cytokine	6.23E-05	CAMK4,DPP4,FAS,GART,GRK2,IFNG,ITGB3,MMP1,MMP2,MUC1,PPARG,SERPINA5,SMTN,TJP1,TLR4,ZEB2
BMP7	growth factor	7.68E-05	BMP1,ESR1,FN1,IGF1R,MMP2,MSN,ZEB2
BACH1	transcription regulator	7.97E-05	HMOX1,MMP1,NDUFAB1,PDK2,PDK3,PDK4
TGFB1	growth factor	8.05E-05	ALB,BMP1,F3,FAP,FAS,FN1,HBEGF,HMOX1,IFNG,ITGB3,MMP1,MMP2,NDC80,SMTN,SMURF2,ZEB2
P38 MAPK	group	8.18E-05	DGKA,F3,FAS,FN1,HMOX1,ITGB3,MMP1,MMP2,MSH2,RARA
CG	complex	1.30E-04	ACP3,AR,C1R,FAS,HK2,IFNGR1,ITGB3,MMP1,MMP2,SMURF2,XIAP
RNF40	enzyme	1.48E-04	G6PD,PDK4,PPARG
NFkB (complex)	complex	1.95E-04	CTCF,F3,FAS,HMOX1,IFNG,KYNU,MMP1,MMP2,MSH2,NOTCH1,SENP2,XIAP
E2F1	transcription regulator	2.00E-04	AR,EXOSC9,FAS,MSH2,NCOA3,NDC80,NOTCH1,PA2G4,RPA3,RRM1,VEGFB
ESRRG	ligand-dependent nuclear receptor	2.11E-04	HK2,KLK1,MDH1,PDHA1,PDK4
JNK	group	2.68E-04	BTRC,IFNG,MMP1,MMP2,MSH2,RARA,XIAP
VEGFA	growth factor	3.02E-04	CD1A,F3,ITGB3,MMP2,NOTCH1
MBD2	transcription regulator	3.02E-04	DPP4,ESR1,G6PD,MMP2,XIAP
DEF6	other	3.11E-04	FN1,MMP2,ZEB2

Akt	group	3.29E-04	F3,IGF1R,MMP2,MUC1,NOTCH1,PDK4,TJP1
SFN	other	3.32E-04	DSG2,F3,PDK4,TJP1
TP63	transcription regulator	3.36E-04	F3,FAS,FN1,G6PD,HBEGF,HK2,NOTCH1,SFN,SMURF2,THBS2,WEE1,ZEB2
ERBB2	kinase	3.77E-04	AR,BMP1,ERBB4,F3,FN1,GART,IFNG,MMP1,MUC1,NDC80,PGK1,PPARG,PTPRF,RRM1,SMTN
TCR	complex	3.90E-04	ALOX12,ARHGEF1,HADH,HSD17B10,IFNG,MARF1,MDH1,ODC1,PA2G4,PGK1,TLR4
IKBKE	kinase	4.18E-04	PDHA1,PDK3,PDK4,PGK1,PYGL
GPI	enzyme	4.23E-04	FN1,RAC1,ZEB2
PAX3-FOXO1	fusion gene/product	4.83E-04	ALK,CLCN5,HMOX1,IGF1R,IMPA2,PPP2R1A,XRCC6
EGR1	transcription regulator	5.13E-04	CDK5R1,F3,FN1,HBEGF,PPARG
TAL1	transcription regulator	5.35E-04	CD1A,DSG2,HBB,HMGB3,LRBA,MUC1,NOTCH1,PTK2B,XRCC6,YARS1
RFX5	transcription regulator	5.59E-04	B2M,CD74,HLA-DRB1
miR-615-3p (miRNAs w/seed CCGAGCC)	mature microRNA	5.91E-04	AR,PPARG
mir-17	microRNA	6.12E-04	AR,ESR1,FAS,NCOA3
mir-322	microRNA	7.19E-04	FN1,IGF1R,SMURF2
OLR1	transmembrane receptor	7.19E-04	MMP1,RARA,RARG
JUN	transcription regulator	7.83E-04	ALOX12,FN1,HK2,HMOX1,IFNG,MMP1,MMP2,VAV3,ZEB2
mir-15	microRNA	8.01E-04	AR,BTRC,IFNG,WEE1
PRKAA1	kinase	8.21E-04	EPHA3,FN1,IGF1R,MAGEA4,TJP1
WT1	transcription regulator	9.63E-04	BTK,ESR1,HBEGF,IGF1R,ODC1,TRIM21,WARS1
CCND1	transcription regulator	1.01E-03	AR,ERI1,ITGB3,KIF11,MAGI2,NCOA3,PAH,UTRN,ZFP28,ZNF462
IL1B	cytokine	1.09E-03	ACP3,BTRC,CYP7A1,DPP4,F3,FAS,IFNG,MMP1,MUC1,PPARG,TLR4
TP53	transcription regulator	1.10E-03	AR,DGKA,ESR1,FAS,FN1,GART,HK2,HMGCS1,HMOX1,IGF1R,MMP2,MSH2,NDC80,NOTCH1,PANK1,PRKCB,RBBP6,SFN,SMURF2,TJP1,YWHAH
SREBF2	transcription regulator	1.12E-03	FABP7,GTF2I,PPARG
RELA	transcription regulator	1.16E-03	B2M,CERT1,CTCF,FAS,FN1,IFNG,MMP1,PPARG,STK4,XIAP
MOAP1	other	1.17E-03	G6PD,HMOX1
mir-192	microRNA	1.17E-03	IGF1R,ZEB2
mir-503	microRNA	1.17E-03	IGF1R,SMURF2
DYSF	other	1.17E-03	FN1,ITGB3
CDK19	kinase	1.25E-03	DCXR,FAS,HBEGF,HMGCS1,HMOX1,NOTCH1,SFN
IL15	cytokine	1.25E-03	G6PD,GALM,HK2,HMOX1,IFNG,IGF1R,PGK1

RXRA	ligand-dependent nuclear receptor	1.35E-03	CYP7A1,HMOX1,PPARG,RARA,RARG
ITGAV	transmembrane receptor	1.36E-03	ITGB3,MMP1,MMP2
ITGB3	transmembrane receptor	1.36E-03	ITGB3,MMP1,MMP2
WNT5A	cytokine	1.38E-03	FN1,HMOX1,ITGB3,MMP1,SEMA4D,TLR4
PRMT1	enzyme	1.45E-03	ALOX12,FN1,PDK4,ZEB2
HRAS	enzyme	1.57E-03	DTYMK,FAS,HMOX1,PRKCB,RRM1
MTOR	kinase	1.65E-03	ACACB,AR,CHKA,HK2,IFNG,MUC1
IGF1	growth factor	1.70E-03	F3,FN1,IGF1R,SFN,XIAP
PKM	kinase	1.70E-03	HK2,MMP2,PC,PDHA1,PDK4
HNF4A	transcription regulator	1.74E-03	CYP7A1,MSN,NR1I3,PPARG,RARA,RARG
c-Src	group	1.93E-03	CYP7A1,HMOX1
KIF26A	other	1.93E-03	RAC1,VAV3
PRKCB	kinase	1.93E-03	FN1,MMP2
SENP2	peptidase	1.93E-03	HK2,PGK1
ICAM1	transmembrane receptor	1.95E-03	IFNG,NOTCH1,ZEB2
CSF3	cytokine	1.95E-03	PPARG,PRKCB,RARA
MMP2	peptidase	1.95E-03	CSF2RB,PPP2R1A,TJP1
PCGF2	transcription regulator	1.95E-03	FN1,NOTCH1,ZEB2
SMAD2	transcription regulator	1.97E-03	FN1,MMP2,RAC1,XIAP
GATA3	transcription regulator	1.97E-03	ECHS1,ESR1,IFNG,TJP1
EPO	cytokine	2.29E-03	MMP2,PRKCB,TJP1
COL18A1	other	2.41E-03	F3,FN1,ITGB3,MMP1,MMP2
PGR	ligand-dependent nuclear receptor	2.64E-03	ESR1,F3,FN1,HBEGF,MATK,MUC1,PNMT,SERPINA5
LGALS1	other	2.66E-03	FN1,IFNGR1,MMP1
CXCL8	cytokine	2.86E-03	AR,CD74,MMP2,RAC1
FGF2	growth factor	2.86E-03	F3,HK2,MMP1,PPARG
NCF1	enzyme	2.87E-03	F3,FN1
mir-548	microRNA	2.87E-03	AR,MMP2
AXL	kinase	2.87E-03	MMP1,MMP2
PTBP3	other	2.87E-03	FN1,ZEB2
COMMD1	transporter	2.87E-03	ATP7B,IFNG

NFIC	transcription regulator	2.87E-03	CYP17A1,FABP7
PI3K (complex)	complex	2.92E-03	CDK5R1,HBEGF,IGF1R,NOTCH1,TLR4
Lh	complex	3.00E-03	ACP3,AR,HK2,MAPK6,MMP2,PGK1,PPP2R1A,PTPRE,PTPRF
IRF8	transcription regulator	3.07E-03	B2M,CD74,TLR4
CDK4	kinase	3.11E-03	ERI1,KIF11,MAGI2,PAH,UTRN,ZFP28,ZNF462
CDH1	other	3.12E-03	ERBB4,FN1,MMP1,XIAP
SPP1	cytokine	3.31E-03	FN1,IFNG,MECR,MMP2,PPARG
RNA polymerase II	complex	3.38E-03	B2M,CYP7A1,ESR1,HBB,MMP2,NCOA3,SFN
EBI3	cytokine	3.52E-03	B2M,FAS,NFATC1
JUNB	transcription regulator	3.69E-03	FN1,HK2,HMOX1,MMP1
Mmp	group	3.99E-03	FAS,TJP1
KLF17	transcription regulator	3.99E-03	FN1,TJP1
MIA	other	3.99E-03	FN1,ITGB3
LOXL2	enzyme	3.99E-03	ESR1,FN1
miR-491-5p (and other miRNAs w/seed GUGGGGA)	mature microRNA	3.99E-03	AR,MMP2
BANCR	other	3.99E-03	MMP1,MMP2
OCLN	other	3.99E-03	MMP1,MMP2
mir-24	microRNA	3.99E-03	IFNG,PSAP
NR1D1	ligand-dependent nuclear receptor	3.99E-03	MDH1,TLR4
mir-19	microRNA	4.01E-03	ESR1,F3,PSAP
CCN2	growth factor	4.01E-03	ESR1,FN1,MMP2
RUNX1	transcription regulator	4.32E-03	ALB,ALOX12,EP300,ITGB3
CDK9	kinase	4.53E-03	G6PD,PDK4,PPARG
mir-181	microRNA	4.53E-03	AR,IFNG,NOTCH1
ANXA2	other	5.10E-03	ANG,FAS,MMP1
CSF2	cytokine	5.18E-03	CD1A,CSF2RB,MMP1,RARA,TLR4,XIAP
CD4	transmembrane receptor	5.27E-03	F3,IFNG
DDR2	kinase	5.27E-03	MMP1,MMP2
ERP29	transporter	5.27E-03	TJP1,ZEB2
ERBB4	kinase	5.27E-03	ERBB4,PGK1

XBP1	transcription regulator	5.40E-03	ESR1,NCOA3,S100A6,XRCC6
SMARCA4	transcription regulator	5.53E-03	CD74,CLK1,F3,FAS,FCGR2A,FLNB,FN1,HBB,IFNG,MMP1,MMP2,MUC1,S100A6,YY1
TRIM37	enzyme	5.71E-03	CUL1,PEX5,SFN
F7	peptidase	5.71E-03	AR,F3,HBEGF
UPF1	enzyme	5.71E-03	HBB,SMG5,SMG7
Hsp90	group	5.79E-03	AR,ESR1,GRK6,IFNG
TRPS1	transcription regulator	6.35E-03	FN1,KIF11,MMP2
HIF1A	transcription regulator	6.58E-03	CHKA,ERBB4,FN1,HK2,IFNG,ITGB3,MMP2,MUC1,NAE1
BMI1	transcription regulator	6.64E-03	CUL1,FAS,HK2,SFN
FZD8	G-protein coupled receptor	6.71E-03	FN1,TJP1
E2F5	transcription regulator	6.71E-03	PA2G4,VEGFB
TPM3	other	6.71E-03	MMP1,MMP2
mir-132	microRNA	6.71E-03	EP300,IFNG
mir-634	microRNA	6.71E-03	AR,XIAP
ETV1	transcription regulator	6.71E-03	ITGB3,MMP1
MCAM	other	6.71E-03	FN1,MMP2
VTN	other	6.71E-03	F3,SFN
NPC1	transporter	8.31E-03	HDAC7,TLR4
DCLK1	kinase	8.31E-03	NOTCH1,ZEB2
RASSF1	other	8.31E-03	ESR1,MMP2
miR-7a-5p (and other miRNAs w/seed GGAAGAC)	mature microRNA	8.31E-03	IGF1R,KMT5A
SRSF1	other	8.31E-03	FN1,RAC1
AKT2	kinase	8.31E-03	ESR1,RAC1
GFI1B	transcription regulator	8.31E-03	CSDE1,ITSN1
CSF1	cytokine	8.55E-03	FAS,FN1,NFATC1
ESR2	ligand-dependent nuclear receptor	8.60E-03	FN1,IGF1R,MMP1,RARA,VAV3
Gsk3	group	9.37E-03	ESR1,IGF1R,XIAP
DLX6-AS1	other	1.01E-02	NOTCH1,WEE1
IDH2	enzyme	1.01E-02	FN1,ZEB2
NRBP2	kinase	1.01E-02	EP300,NOTCH1

IGFBP5	other	1.01E-02	F3,SFN
CD209	other	1.01E-02	MMP2,TJP1
LINC-ROR	other	1.01E-02	FAS,NOTCH1
FABP2	transporter	1.01E-02	ACACB,PPARG
DDX3X	enzyme	1.01E-02	AR,RAC1
POSTN	other	1.01E-02	MMP2,VEGFB
ECM1	transporter	1.01E-02	MUC1,ZEB2
HEIH	other	1.02E-02	FAS,MMP2,VEGFB
CCN1	other	1.02E-02	FN1,ITGB3,SENP2
NEUROG1	transcription regulator	1.02E-02	FABP3,FAP,FN1,ITGB3
EGLN	group	1.12E-02	HK2,PDK3,PYGL,SH2B2,ZNF292
BCOR	transcription regulator	1.20E-02	CD74,TLR4
PLK1	kinase	1.20E-02	ESR1,SFN
NR6A1	ligand-dependent nuclear receptor	1.20E-02	BMP1,TLR4
IL3	cytokine	1.21E-02	CD1A,FCGR2A,MMP1
miR-145-5p (and other miRNAs w/seed UCCAGUU)	mature microRNA	1.21E-02	DFFA,MMP1,MUC1
BRAF	kinase	1.21E-02	BTRC,MMP1,XIAP
IL27	cytokine	1.21E-02	B2M,FAS,IFNG,NFATC1
STAT3	transcription regulator	1.26E-02	ESR1,FN1,HK2,HMOX1,MMP2,MUC1,NOTCH1,PGK1
POU5F1	transcription regulator	1.29E-02	DFFA,FAS,IGF1R,MMP2,WEE1,ZEB2
WWTR1	transcription regulator	1.31E-02	DTYMK,FN1,RRM1
IGF1R	transmembrane receptor	1.31E-02	ESR1,IGF1R,RAC1
TCF12	transcription regulator	1.31E-02	FN1,LRBA,XRCC6
MYCN	transcription regulator	1.31E-02	APEX1,HK2,PA2G4
CDKN1A	kinase	1.35E-02	FN1,MMP1,PSAP,RRM1
IL18	cytokine	1.35E-02	FAS,IFNG,MMP1,TLR4
SAA	group	1.40E-02	F3,MMP1
MDGA2	other	1.40E-02	FAS,MSH2
MED16	transcription regulator	1.40E-02	MMP1,MMP2
APOA1	transporter	1.40E-02	CHKA,TLR4

MS4A1	other	1.40E-02	HK2,XIAP
IL7	cytokine	1.40E-02	FAS,IFNG
Dgk	group	1.41E-02	CYP17A1
Pdgf (complex)	complex	1.41E-02	MMP1
ATP synthase	complex	1.41E-02	IFNG
DIS3L2	enzyme	1.41E-02	RAC1
BLVRA	enzyme	1.41E-02	CERT1
FBXL17	other	1.41E-02	HMOX1
PROM1	other	1.41E-02	DSG2
S100A3	transporter	1.41E-02	RARA
RBMY1A1 (includes others)	other	1.41E-02	AR
VPS36	other	1.41E-02	ESR1
USP12	peptidase	1.41E-02	AR
LAYN	other	1.41E-02	IFNG
PABIR1	other	1.41E-02	WEE1
LECT2	other	1.41E-02	PPARG
SMG6	enzyme	1.41E-02	HBB
S100A14	other	1.41E-02	MMP2
KIR	group	1.41E-02	IFNG
XDH	enzyme	1.41E-02	F3
SELL	transmembrane receptor	1.41E-02	IFNG
CD6	transmembrane receptor	1.41E-02	IFNG
HLTF	transcription regulator	1.41E-02	HBB
CGB3 (includes others)	other	1.41E-02	MMP2
IFNGR1	transmembrane receptor	1.41E-02	FAS
GNB5	enzyme	1.41E-02	XIAP
miR-155-3p (miRNAs w/seed UCCUACA)	mature microRNA	1.41E-02	AR
miR-223-5p (miRNAs w/seed GUGUAUU)	mature microRNA	1.41E-02	AR
mir-448	microRNA	1.41E-02	AR

mir-567	microRNA	1.41E-02	AR
miR-509-3p (miRNAs w/seed GAUUGGU)	mature microRNA	1.41E-02	AR
miR-92a-2-5p (miRNAs w/seed GGUGGGG)	mature microRNA	1.41E-02	AR
miR-488-5p (miRNAs w/seed CCAGAUA)	mature microRNA	1.41E-02	AR
mir-488	microRNA	1.41E-02	AR
miR-29a-5p (miRNAs w/seed CUGAUUU)	mature microRNA	1.41E-02	AR
miR-93-3p (miRNAs w/seed CUGCUGA)	mature microRNA	1.41E-02	AR
mir-544	microRNA	1.41E-02	AR
miR-299a-3p (and other miRNAs w/seed AUGUGGG)	mature microRNA	1.41E-02	AR
miR-28-3p (and other miRNAs w/seed ACUAGAU)	mature microRNA	1.41E-02	AR
mir-365	microRNA	1.41E-02	AR
mir-541	microRNA	1.41E-02	AR
miR-1273h-5p (and other miRNAs w/seed UGGGAGG)	mature microRNA	1.41E-02	AR
miR-129-1-3p (and other miRNAs w/seed AGCCCUU)	mature microRNA	1.41E-02	AR
miR-767 (and other miRNAs w/seed GCACCAU)	mature microRNA	1.41E-02	AR
mir-631	microRNA	1.41E-02	AR

miR-744-3p (miRNAs w/seed UGUUGCC)	mature microRNA	1.41E-02	AR
miR-876-3p (miRNAs w/seed GGUGGUU)	mature microRNA	1.41E-02	AR
miR-501-3p (and other miRNAs w/seed AUGCACC)	mature microRNA	1.41E-02	AR
mir-646	microRNA	1.41E-02	AR
miR-367-5p (miRNAs w/seed CUGUUGC)	mature microRNA	1.41E-02	AR
miR-149-3p (and other miRNAs w/seed GGGAGGG)	mature microRNA	1.41E-02	AR
mir-552	microRNA	1.41E-02	AR
mir-298	microRNA	1.41E-02	AR
mir-493	microRNA	1.41E-02	IGF1R
miR-541-3p (and other miRNAs w/seed GGUGGGC)	mature microRNA	1.41E-02	AR
mir-586	microRNA	1.41E-02	AR
mir-644	microRNA	1.41E-02	AR
miR-486-3p (and other miRNAs w/seed GGGGCAG)	mature microRNA	1.41E-02	AR
PPP2R5E	phosphatase	1.41E-02	FN1
DNAJB2	other	1.41E-02	ATXN3
GBP1	enzyme	1.41E-02	MMP1
COL5A1	other	1.41E-02	FN1
BCAR1	enzyme	1.41E-02	ESR1
HTRA2	peptidase	1.41E-02	XIAP
KIR2DS2 (includes others)	transmembrane receptor	1.41E-02	IFNG
SLC2A1	transporter	1.41E-02	MMP2

RYK	kinase	1.41E-02	FN1
PSMC3IP	transcription regulator	1.41E-02	CYP17A1
ASH2L	transcription regulator	1.41E-02	ESR1
ALDH1A3	enzyme	1.41E-02	RRM1
PLPP3	phosphatase	1.41E-02	FN1
SNCG	other	1.41E-02	IGF1R
MXD4	transcription regulator	1.41E-02	ODC1
B4GALT5	enzyme	1.41E-02	NOTCH1
KIR2DL1/KIR2DL3	other	1.41E-02	IFNG
ICAM3	transmembrane receptor	1.41E-02	PPARG
CAPNS1	peptidase	1.41E-02	MMP2
KIF4A	other	1.41E-02	AR
ARL6IP5	other	1.41E-02	ITGB3
PTPN9	phosphatase	1.41E-02	MMP2
PES1	other	1.41E-02	HMOX1
GPX4	enzyme	1.41E-02	MMP1
CD226	other	1.41E-02	IFNG
MRC1	transmembrane receptor	1.41E-02	IFNG
KLRB1	transmembrane receptor	1.41E-02	IFNG
RANBP2	enzyme	1.41E-02	FN1
NBN	other	1.41E-02	XIAP
mir-297	microRNA	1.41E-02	AR
CTD_2245E153	other	1.41E-02	PC
SOCS2	other	1.41E-02	IFNG
protein phosphatase	complex	1.41E-02	ESR1
ATF3	transcription regulator	1.42E-02	DCTD,IFNG,MSH2,RRM1
PI3K (family)	group	1.43E-02	ESR1,HMOX1,MMP2,TJP1,XIAP
AR	ligand-dependent nuclear receptor	1.46E-02	AR,IGF1R,MMP2,MUC1,PGC,SMTN,TLR4,WEE1
BRCA1	transcription regulator	1.49E-02	ESR1,FHIT,HBB,XRCC6
ETV5	transcription regulator	1.52E-02	FN1,MMP2,TJP1
OGA	enzyme	1.53E-02	FLNB,FN1,G6PD,GTF2F2,HMGCS1,MAP3K3,PDHA1,S100A6
MEG3	other	1.62E-02	FN1,ZEB2

SULF2	enzyme	1.62E-02	MMP2,VEGFB
GALNT6	enzyme	1.62E-02	DPP4,FN1
IFT88	other	1.62E-02	ACAT2,MMP2
LCK	kinase	1.62E-02	IFNG,PPARG
PTP4A3	phosphatase	1.62E-02	FN1,ITGB3
SMAD7	transcription regulator	1.62E-02	IFNG,XIAP
miR-296-5p (miRNAs w/seed GGGCCCC)	mature microRNA	1.62E-02	KLK1,MMP1
MXI1	transcription regulator	1.62E-02	APEX1,LARS1
ABL1	kinase	1.62E-02	FAS,MMP1
TXNIP	other	1.62E-02	G6PD,PGK1
CFLAR	other	1.62E-02	HMOX1,XIAP
MAX	transcription regulator	1.64E-02	APEX1,BCR,CSDE1
MET	kinase	1.64E-02	FN1,HMOX1,MMP2
FOXA2	transcription regulator	1.75E-02	ALB,RARA,RARG
CCN5	growth factor	1.75E-02	ESR1,FN1,MUC1
IDH1	enzyme	1.85E-02	FN1,ZEB2
mir-218	microRNA	1.85E-02	VAV3,ZEB2
SPARC	other	1.85E-02	FN1,NOTCH1
EIF4E	translation regulator	1.85E-02	ESR1,XIAP
IFN Beta	group	1.88E-02	CD74,FN1,IFNG
ERG	transcription regulator	1.88E-02	AR,BCR,FLNB,IGF1R,POU2F1,UTRN
KDM1A	enzyme	1.94E-02	CRYM,CYP7A1,HBB,IGF1R,RUVBL1,SMURF2
mir-155	microRNA	2.00E-02	HK2,IFNG,WEE1
IKZF1	transcription regulator	2.07E-02	B2M,FN1,TLR4,TNFAIP8L2,UEVLD
DPH5	enzyme	2.09E-02	EP300,IFNG
PAX5	transcription regulator	2.09E-02	FN1,MMP2
C3	peptidase	2.09E-02	FN1,IFNG
CLU	other	2.09E-02	ATP7B,MMP2
SOX9	transcription regulator	2.09E-02	AR,PPARG
PRDM1	transcription regulator	2.09E-02	ESR1,IFNG
KLRC4-KLRK1/KLRK1	transmembrane receptor	2.09E-02	IFNG,PPARG

Ap1	complex	2.14E-02	F3,MMP1,MMP2
mir-21	microRNA	2.14E-02	FAS,FN1,MSH2
FAS	transmembrane receptor	2.14E-02	FAS,MMP1,RND1
PPARG	ligand-dependent nuclear receptor	2.17E-02	FN1,MUC1,PDK4,PPARG
CD44	other	2.17E-02	EP300,FAS,FN1,XIAP
IL4	cytokine	2.22E-02	CD1A,IFNG,MMP1,MUC1,PPARG
SMAD4	transcription regulator	2.22E-02	CYP17A1,FAP,IFNG,RAC1,XIAP
STAT5a/b	group	2.27E-02	IFNG,RARA,XIAP
MAP2K1	kinase	2.27E-02	F3,MMP1,MMP2
HMOX1	enzyme	2.27E-02	HMOX1,ITGB3,THBS2
NCOA3	transcription regulator	2.27E-02	HMOX1,NCOA3,PPARG
IL22	cytokine	2.35E-02	IFNG,MUC1
FGFR1	kinase	2.35E-02	HK2,SFN
CTBP1	enzyme	2.35E-02	MUC1,RAC1
DDIT3	transcription regulator	2.35E-02	FN1,IFNG
PAK1	kinase	2.35E-02	F3,FN1
IL10	cytokine	2.36E-02	CD1A,IFNG,MUC1,TLR4
SREBF1	transcription regulator	2.37E-02	ALOX12,FABP3,FABP7,HMOX1,OAT
RBM5	other	2.41E-02	ITPA,NCOA3,XIAP
mir-8	microRNA	2.56E-02	MMP1,RAC1,ZEB2
PKNOX2	transcription regulator	2.62E-02	ESR1,FAS
NPPB	other	2.62E-02	ACAT2,HMGCS1
TWIST2	transcription regulator	2.62E-02	FN1,ZEB2
HNF1B	transcription regulator	2.62E-02	ALB,SLC5A1
NPC2	transporter	2.62E-02	PPARG,TLR4
MBD1	transcription regulator	2.62E-02	DPP4,ESR1
KLF11	transcription regulator	2.62E-02	FABP3,PDK4
MUC1	other	2.71E-02	CD1A,MUC1,NOTCH1
E2F4	transcription regulator	2.78E-02	EXOSC9,HMGB3,MSH2,NDC80,RPA3,RRM1
Alpha 1 antitrypsin	group	2.81E-02	MMP2
Ras homolog	group	2.81E-02	TJP1
Ikb	group	2.81E-02	MMP1

CK1	group	2.81E-02	XIAP
Rbp	group	2.81E-02	PPARG
BTN3A1	other	2.81E-02	IFNG
SLC48A1	transporter	2.81E-02	IGF1R
SNX27	other	2.81E-02	PPARG
VSIR	other	2.81E-02	IFNG
Integrin alpha V beta 3	complex	2.81E-02	MMP1
BTBD7	other	2.81E-02	MMP2
CNEP1R1	other	2.81E-02	IFNG
DENND1A	other	2.81E-02	CYP17A1
ESRP2	other	2.81E-02	ZEB2
ZNF385B	other	2.81E-02	FAS
FOXG1	transcription regulator	2.81E-02	NCOA3
Vla-4	complex	2.81E-02	F3
FAS-AS1	other	2.81E-02	FAS
Ferritin	complex	2.81E-02	HBB
VTRNA2-1	other	2.81E-02	IFNG
CABIN1	other	2.81E-02	MMP2
MXD1	transcription regulator	2.81E-02	ODC1
CD244	transmembrane receptor	2.81E-02	IFNG
IBSP	other	2.81E-02	ITGB3
PMEPA1	other	2.81E-02	AR
NUP98	transporter	2.81E-02	YWHAH
TAC4	other	2.81E-02	MMP2
SP100	transcription regulator	2.81E-02	MMP1
ARID5B	transcription regulator	2.81E-02	IFNG
LAMA3	other	2.81E-02	MMP1
LY96	transmembrane receptor	2.81E-02	TLR4
PIAS2	transcription regulator	2.81E-02	MUC1
mir-708	microRNA	2.81E-02	AR

miR-16-1-3p (miRNAs w/seed CAGUAUU)	mature microRNA	2.81E-02	WEE1
miR-675-3p (miRNAs w/seed UGUAUGC)	mature microRNA	2.81E-02	TJP1
miR-424-3p (miRNAs w/seed AAAACGU)	mature microRNA	2.81E-02	WEE1
mir-368	microRNA	2.81E-02	AR
miR-292-3p (and other miRNAs w/seed AGUGCCG)	mature microRNA	2.81E-02	AR
miR-128-3p (and other miRNAs w/seed CACAGUG)	mature microRNA	2.81E-02	WEE1
mir-346	microRNA	2.81E-02	BTK
miR-4728-3p (miRNAs w/seed AUGCUGA)	mature microRNA	2.81E-02	ESR1
MIR4728	microRNA	2.81E-02	ESR1
miR-758-5p (and other miRNAs w/seed GGUUGAC)	mature microRNA	2.81E-02	AR
EGFL7	other	2.81E-02	NOTCH1
KAT6A	enzyme	2.81E-02	ESR1
MTBP	other	2.81E-02	ODC1
PDE4A	enzyme	2.81E-02	FN1
FCGR1A	transmembrane receptor	2.81E-02	F3
CDC14A	phosphatase	2.81E-02	WEE1
NCSTN	peptidase	2.81E-02	NOTCH1
VPS35	transporter	2.81E-02	PPARG
USP14	peptidase	2.81E-02	AR
PDCD6IP	other	2.81E-02	ESR1
BCAN	other	2.81E-02	ITGB3

DRD5	G-protein coupled receptor	2.81E-02	IFNG
BRD8	transcription regulator	2.81E-02	PPARG
SOX10	transcription regulator	2.81E-02	NOTCH1
PTGS1	enzyme	2.81E-02	MMP2
TRADD	other	2.81E-02	AR
SLAMF6	transmembrane receptor	2.81E-02	IFNG
CD276	other	2.81E-02	IFNG
BMP5	growth factor	2.81E-02	CYP17A1
PPP1CA	phosphatase	2.81E-02	AR
PARP2	enzyme	2.81E-02	FAS
ENTPD1	enzyme	2.81E-02	IFNG
HP	peptidase	2.81E-02	HMOX1
NDFIP2	other	2.81E-02	IFNG
PTK2B	kinase	2.81E-02	IFNG
ANGPTL4	other	2.81E-02	MMP1
BRD2	kinase	2.81E-02	HMOX1
DGKA	kinase	2.81E-02	WEE1
CCR5	G-protein coupled receptor	2.81E-02	F3
RAMAC	other	2.81E-02	RUVBL1
LGALS9	other	2.81E-02	IFNG
NOS1AP	other	2.81E-02	RAC1
IL12RB2	transmembrane receptor	2.81E-02	IFNG
EIF4A1	translation regulator	2.81E-02	MUC1
NCOA4	transcription regulator	2.81E-02	AR
HNRNPAB	enzyme	2.81E-02	HBB
THRB	ligand-dependent nuclear receptor	2.87E-02	IGF1R,MMP1,PPARG
STAT1	transcription regulator	2.88E-02	C1R,FAS,MUC1,TRIM21,WARS1
CLDN7	other	2.88E-02	ASAP1,F3,MMP2,MUC1,SF3B6
RICTOR	other	2.90E-02	HK2,RAC1
mir-27	microRNA	2.90E-02	ESR1,PSAP
mir-146	microRNA	2.90E-02	IFNG,TLR4
JARID2	transcription regulator	2.90E-02	PPARG,ZEB2

SFPQ	other	2.90E-02	AR,CYP17A1
NFKBIA	transcription regulator	2.90E-02	MSH2,XIAP
TCF7	transcription regulator	2.90E-02	PPARG,RARA
NR3C1	ligand-dependent nuclear receptor	2.93E-02	BTK,CUL1,DAPK2,FN1,IGF1R,NFATC1,NMT1,NR1I3,PRKCB,PTK2B,YWHAH
TLR4	transmembrane receptor	3.01E-02	IFNG,MMP1,RAC1,TLR4
ESRRA	transcription regulator	3.06E-02	HK2,HMOX1,PNMT,PPARG,RARA
F2	peptidase	3.20E-02	FN1,MMP1
SDCBP	enzyme	3.20E-02	MMP2,RAC1
C5	cytokine	3.20E-02	FN1,MMP1
PML-RARA	fusion gene/product	3.20E-02	F3,HMOX1
EML4-ALK	fusion gene/product	3.24E-02	CERT1,MMP1,MMP2,PPARG
TREM1	transmembrane receptor	3.32E-02	F3,FABP3,HBEGF,MMP1,PPARG,SFMBT2
Secretase gamma	complex	3.50E-02	NOTCH1,PTPRF
Tlr	group	3.50E-02	IFNG,NOTCH1
JUND	transcription regulator	3.50E-02	F3,HMOX1
SOX11	transcription regulator	3.64E-02	CUL1,EP300,ERBB4,FAS,HMGB3
SAFB	other	3.71E-02	B2M,CD74,HMOX1
FSH	complex	3.79E-02	ACP3,HK2,MAPK6,MMP2,PGK1,PPP2R1A,PTPRE,PTPRF
POU2F1	transcription regulator	3.82E-02	APEX1,ESR1
TFEB	transcription regulator	3.82E-02	PSAP,VPS26A
IRF4	transcription regulator	3.88E-02	B2M,IFNG,STK4,SUB1
MAP2K1/2	group	3.90E-02	CYP7A1,HK2,RRM1
FOS	transcription regulator	4.01E-02	FN1,HK2,MMP1,VAV3
IRF1	transcription regulator	4.08E-02	B2M,IFNG,ODC1
KLF5	transcription regulator	4.08E-02	NOTCH1,PPARG,RARA
SRC (family)	group	4.14E-02	FN1,ITGB3
HLX	transcription regulator	4.14E-02	IFNG,KYNU
CCL2	cytokine	4.14E-02	MMP1,MMP2
RAF1	kinase	4.14E-02	FAS,MMP1
MIF	cytokine	4.14E-02	MMP1,MMP2
FFAR3	G-protein coupled receptor	4.18E-02	PPARG
HSP	group	4.18E-02	IFNG

PI3K 纬	group	4.18E-02	IFNG
MIR100HG	other	4.18E-02	FN1
CREB3L3	transcription regulator	4.18E-02	CYP7A1
CCDC134	other	4.18E-02	IFNG
NDUFAF3	other	4.18E-02	ESR1
MMS19	transcription regulator	4.18E-02	CIAO1
FFAR2	G-protein coupled receptor	4.18E-02	PPARG
NAA30	enzyme	4.18E-02	FAS
ASCL1	transcription regulator	4.18E-02	CDK5R1
KLHL42	other	4.18E-02	FN1
CIAO2B	other	4.18E-02	CIAO1
FBXL16	other	4.18E-02	NCOA3
MAGEA3/MAGEA6	other	4.18E-02	IFNG
gelatinase	group	4.18E-02	TJP1
MEN1	transcription regulator	4.18E-02	AR
POLG	enzyme	4.18E-02	AR
HEY2	transcription regulator	4.18E-02	SMTN
AP2M1	other	4.18E-02	CD74
STAT4	transcription regulator	4.18E-02	IFNG
SH2D1A	other	4.18E-02	IFNG
COL3A1	other	4.18E-02	FN1
TAF4	transcription regulator	4.18E-02	AR
TIMP2	other	4.18E-02	MMP2
FBLN2	other	4.18E-02	MMP2
ARHGAP31	other	4.18E-02	ZEB2
miR-222-5p (miRNAs w/seed UCAGUAG)	mature microRNA	4.18E-02	AR
mir-147	microRNA	4.18E-02	AR
mir-95	microRNA	4.18E-02	AR
miR-132-3p (and other miRNAs w/seed AACAGUC)	mature microRNA	4.18E-02	EP300

miR-346 (and other miRNAs w/seed GUCUGCC)	mature microRNA	4.18E-02	BTK
miR-1-3p (and other miRNAs w/seed GGAAUGU)	mature microRNA	4.18E-02	ESR1
HGS	other	4.18E-02	ESR1
PTGDR	G-protein coupled receptor	4.18E-02	IFNG
EPHB4	kinase	4.18E-02	AR
CEACAM3	other	4.18E-02	IFNG
DDR1	kinase	4.18E-02	MMP1
KCNMA1	ion channel	4.18E-02	MMP2
PRKCQ	kinase	4.18E-02	IFNG
CD86	transmembrane receptor	4.18E-02	IFNG
GFER	enzyme	4.18E-02	NR1I3
MAP3K5	kinase	4.18E-02	MSH2
HTRA1	peptidase	4.18E-02	MMP1
RBP4	other	4.18E-02	RARA
PAX2	transcription regulator	4.18E-02	ITGB3
ITGB6	other	4.18E-02	MMP2
USP19	peptidase	4.18E-02	ATXN3
AJUBA	transcription regulator	4.18E-02	WEE1
C8orf44-SGK3/SGK3	kinase	4.18E-02	ESR1
SENP1	peptidase	4.18E-02	MMP2
CD48	other	4.18E-02	IFNG
TGFBR3	kinase	4.18E-02	FN1
RECQL	enzyme	4.18E-02	HBB
CCNA1	other	4.18E-02	MMP2
XCL1	cytokine	4.18E-02	IFNG
CD247	transmembrane receptor	4.18E-02	IFNG
CRLF1	other	4.18E-02	IFNG
EXOC7	transporter	4.18E-02	ZEB2
G3BP2	enzyme	4.18E-02	SART3

TFE3	transcription regulator	4.18E-02	VPS26A
SMAD6	transcription regulator	4.18E-02	IFNG
GADD45GIP1	other	4.18E-02	HMOX1
ACTG1-MITF	fusion gene/product	4.18E-02	APEX1
EP300	transcription regulator	4.29E-02	AR,CHKA,FN1,MMP1
CST5	other	4.30E-02	ACAT2,ANXA6,ARF6,HNRNPA2B1,KIF11,MSN,NCBP1,NF2
MAPK9	kinase	4.47E-02	PPARG,RAC1,SEM1
PIM1	kinase	4.48E-02	IFNG,IGF1R
RAC1	enzyme	4.48E-02	MMP1,RAC1
Interferon alpha	group	4.64E-02	BCR,CSF2RB,IFNG,NF2,NT5C3A,SLC5A1,TLR4
PPARA	ligand-dependent nuclear receptor	4.67E-02	CYP7A1,EP300,PDK4
SP3	transcription regulator	4.73E-02	CYP17A1,ESR1,IGF1R,MMP2
SMAD3	transcription regulator	4.73E-02	FN1,IFNG,RAC1,XIAP
CAV1	transmembrane receptor	4.82E-02	HK2,TJP1

Supplementary Table S5: The causal regulatory network analysis by IPA for solasonine. The causal regulatory network analysis by IPA was performed to explore the causal network these predicted upstream regulators may be involved in and their correspondent master regulators. Master regulators were predicted and sorted by increasing of p-value (p-value ≤0.05).

Master regulator	Molecule type	p-value	Network bias-corrected p-value	Participating regulators	Target molecules in dataset of solasonine related genes	Number of target molecules-connected regulators	Number of target molecules
DDX5	enzyme	8.91E-14	1.00E-04	DDX5,ERK1/2,let-7,MMP2,MMP9,NFkB (complex)	AR,B2M,CSF2RB,CTCF,CYP17A1,CYP7A1,ESR1,FAS,FN1,HBEGF,HK2,HMOX1,IFNG,IGF1R,ITGB3,KYNU,MAPK6,MDH1,MMP1,MMP2,MSH2,NDUFAB1,NOTCH1,PA2G4,PPP2R1A,PTPRE,SENP2,STARD13,TJP1,TLR4,XIAP	6	31
let-7	microRNA	1.79E-13	2.00E-04	ERK1/2,let-7,MMP2,MMP9,NFkB (complex)	AR,B2M,CSF2RB,CTCF,CYP17A1,CYP7A1,ESR1,FAS,FN1,HBEGF,HK2,HMOX1,IFNG,IGF1R,ITGB3,KYNU,MAPK6,MMP1,MMP2,MSH2,NOTCH1,PA2G4,PPP2R1A,PTPRE,SENP2,STARD13,TJP1,TLR4,XIAP	5	29
TIMP2	other	2.33E-12	5.00E-04	Akt,ERK1/2,GSK3B,MMP2,PI3K (family),RELA,TIMP2	B2M,CERT1,CSF2RB,CTCF,CYP17A1,CYP7A1,ESR1,F3,FAS,FN1,HBEGF,HK2,HMOX1,IFNG,IGF1R,MMP1,MMP2,MUC1,NOTCH1,PDK4,PPARG,PPP2R1A,PTPRE,RAC1,STK4,XIAP	7	26
miR-491-5p (and other miRNAs w/seed GUGGGGA)	mature microRNA	3.36E-12	4.00E-04	ERK1/2,Hsp90,miR-491-5p (and other miRNAs w/seed GUGGGGA),MMP2,MMP9,NFkB (complex)	AR,B2M,CSF2RB,CTCF,CYP17A1,CYP7A1,ESR1,FAS,FN1,GRK6,HBEGF,HK2,HMOX1,IFNG,IGF1R,KYNU,MMP1,MMP2,MSH2,NOTCH1,PPP2R1A,PTPRE,SENP2,TJP1,XIAP	6	25
RASGRP1	other	5.94E-12	6.00E-04	ERK,ERK1/2,Jnk,KRAS,RASGRP1	B2M,BTRC,CDK5R1,CYP17A1,CYP7A1,ESR1,FAS,FN1,HK2,IFNG,IGF1R,ITGB3,MMP1,MMP2,MSH2,MUC1,ODC1,PC,PDK4,PTPRE,RARA,XIAP,YY1	5	23
RAF1	kinase	8.66E-12	1.40E-03	ADCY,CHUK,ERK,ERK1/2,IKBKB,Jnk,MAPK1,RAF1,RB1,RELA,SRC	AR,B2M,BTRC,CDK5R1,CERT1,CTCF,CYP17A1,CYP7A1,DGKA,ESR1,FAS,FN1,HBB,HK2,IFNG,IGF1R,ITGB3,MMP1,MMP2,MSH2,MUC1,NFATC1,NOTCH1,ODC1,PDK4,PPARG,PTPRE,RARA,STK4,TRIM21,XIAP,ZEB2	11	32
PDPN	other	9.63E-12	1.20E-03	ERK,ERK1/2,MMP2,MMP9,P38 MAPK,PDPN,RHOA,RHOC,Tgf beta	B2M,CDK5R1,CSF2RB,CYP17A1,CYP7A1,DGKA,ESR1,F3,HK2,HMOX1,IFNG,IGF1R,ITGB3,MMP2,MSH2,MUC1,ODC1,PDHA1,PDK4,PPP2R1A,PTPRE,RARA,TJP1,XIAP	7	24
ARHGAP35	transcription regulator	1.03E-11	1.20E-03	ARHGAP35,ERK,ERK1/2,MMP2,MMP9,P38 MAPK,RHOA,RHOC,Tgf beta	B2M,CDK5R1,CSF2RB,CYP17A1,CYP7A1,DGKA,ESR1,F3,HK2,HMOX1,IFNG,IGF1R,ITGB3,MMP2,MSH2,MUC1,ODC1,PDHA1,PDK4,PPP2R1A,PTPRE,RARA,TJP1,XIAP	7	24

TNFSF11	cytokine	1.22E-11	8.00E-04	ERK1/2,NFkB (complex),STAT1,TNFSF11	B2M,C1R,CTCF,CYP17A1,CYP7A1,ESR1,FAS,FN1,HBEGF,HK2,HMOX1,IFNG,IGF1R,KYNU,MMP1,MMP2,MSH2,MUC1,NFATC1,NOTCH1,PTPRE,SENP2,TRIM21,WARS1,XIAP	4	25
RPTOR	other	1.48E-11	1.00E-03	Akt,EIF4E,ERK1/2,MTOR,RAC1,RHOA,RPTOR,SREBF1,SREBF2,TFEB	AR,B2M,CHKA,CYP17A1,CYP7A1,ESR1,F3,FABP3,FABP7,FN1,GTF2I,HBEGF,HK2,IFNG,IGF1R,MMP1,NOTCH1,PDHA1,PDK4,PPARG,PSAP,PTPRE,TJP1,VPS26A,XIAP	9	25
IKBKE	kinase	3.00E-11	5.00E-03	Akt,ERK1/2,GSK3B,IKBKE,IRF3,MAP2K1/2,NFkB (complex),P38 MAPK,RELA	B2M,CERT1,CYP17A1,CYP7A1,DGKA,ESR1,FAS,HBEGF,HK2,IFNG,ITGB3,KYNU,MMP1,MMP2,MUC1,NOTCH1,PDHA1,PDK3,PDK4,PGK1,PPARG,PTPRE,PYGL,RAC1,RARA,RRM1,SENP2,STK4,TJP1,XIAP	9	30
ARRB1	transcription regulator	3.18E-11	1.90E-03	ADCY,AKT1,ARRB1,EGFR,ERK,ERK1/2,IGF1R,Jnk,MAPK1,NR3C1,Ras homolog,RELA	B2M,BTRC,CDK5R1,CERT1,CTCF,CYP17A1,CYP7A1,ESR1,F3,FN1,HBB,HK2,HMOX1,HNRNPA2B1,IGF1R,ITGB3,MMP1,MMP2,MSH2,MUC1,NFATC1,NR1I3,ODC1,PDK4,PPARG,PTPRE,RAC1,RARA,STK4,TJP1,TRIM21,XIAP,ZEB2	11	33
LCK	kinase	3.48E-11	3.30E-03	DGKA,ERK,ERK1/2,Jnk,LCK,Mapk,NFkB (complex),PTPN6	B2M,BTRC,CDK5R1,CTCF,CYP17A1,CYP7A1,ESR1,FN1,HK2,HMOX1,IFNG,IGF1R,ITGB3,KYNU,MMP1,MMP2,MUC1,NOTCH1,NR1I3,ODC1,PDK4,PPARG,PTPRE,RARA,SENP2,WEE1	8	26
RHOC	enzyme	3.53E-11	2.00E-04	ERK,MMP2,MMP9,P38 MAPK,RHOA,RHOC	CDK5R1,CSF2RB,DGKA,ESR1,F3,FN1,HBEGF,HMOX1,IFNG,ITGB3,MMP1,MMP2,MSH2,MUC1,ODC1,PDHA1,PDK4,PPP2R1A,RARA,TJP1	5	20
LYN	kinase	4.81E-11	3.20E-03	EGFR,ERK1/2,LYN,MAPK8,NFkB (complex),RAC1,STAT1	AR,B2M,C1R,CTCF,CYP17A1,CYP7A1,ESR1,F3,FAS,FN1,HBEGF,HK2,HMOX1,HNRNPA2B1,IGF1R,KYNU,MMP1,MMP2,MSH2,MUC1,NOTCH1,PTPRE,RARA,SENP2,TRIM21,WARS1,ZEB2	6	27
IL31	other	5.03E-11	5.30E-03	Akt,ERK1/2,Hsp27,IL31,JAK1,JAK2,Jnk,Mapk,MAPK8,MAPK9,P38 MAPK,PI3K (complex),STAT1,STAT3	B2M,BTRC,C1R,CD1A,CYP17A1,CYP7A1,DGKA,ESR1,FAS,HBEGF,HK2,HMOX1,IFNG,ITGB3,MMP1,MMP2,MSH2,MUC1,NOTCH1,NR1I3,PDK4,PGK1,PPARG,PTPRE,RAC1,RARA,SEM1,TJP1,TRIM21,WARS1,XIAP	13	31
IL15	cytokine	5.11E-11	4.00E-03	Akt,ERK1/2,IL15,JAK1,Jnk,P38 MAPK,STAT1,STAT5a/b,TERT	AR,B2M,BTRC,C1R,CYP17A1,CYP7A1,DGKA,ESR1,FAS,G6PD,GALM,HBEGF,HK2,HMOX1,IFNG,IGF1R,ITGB3,MMP1,MMP2,MSH2,MUC1,NOTCH1,PDK4,PGK1,PTPRE,RARA,TJP1,TRIM21,WARS1,XIAP	9	30
PF4	cytokine	8.45E-11	2.70E-03	ERK1/2,Jnk,P38 MAPK,PF4,PI3K (complex)	B2M,BTRC,CD1A,CYP17A1,CYP7A1,DGKA,ESR1,F3,FAS,HBEGF,HK2,HMOX1,IFNG,IGF1R,ITGB3,MMP1,MMP2,MSH2,NOTCH1,PTPRE,RARA,XIAP	5	22
RICTOR	other	8.65E-11	1.50E-03	Akt,AKT1,AKT2,FOXO1,MTOR,RAC1,RHOA,RICTOR,SREBF1,SREBF2,STAT1,TYK2	AR,C1R,CAMK4,CHKA,ESR1,F3,FABP3,FABP7,FAS,GTF2I,HK2,HMOX1,IFNG,IGF1R,KIF11,MMP1,MMP2,MUC1,NOTCH1,PDHA1,PDK4,PPARG,RAC1,TJP1,TRIM21,WARS1	12	26

CHRNA7	transmembrane receptor	9.58E-11	1.63E-02	Akt,CEBPB,CHRNA7,EGFR,ERK,FOXO1,GSK3B,Hsp27,IRF3,KAT5,MMP2,MYC,NFkB (complex),NR3C1,P38 MAPK,PGR,PPARGC1A,RELA,SREBF2,STAT3,TP63,YAP1,YBX1	ALB,APEX1,BCR,CAMK4,CD1A,CDK5R1,CERT1,CSF2RB,CYP7A1,DGKA,DTYMK,F3,FABP7,FAP,FAS,FN1,GTF2I,HBB,HK2,HMOX1,HNRNPA2B1,IFNG,IGF1R,ITGB3,KIF11,KYNU,MATK,MMP1,MSH2,MUC1,NFATC1,NOTCH1,NR1I3,ODC1,PDK4,PGK1,PNMT,PPP2R1A,PTPRE,RAC1,RARA,RRM1,SENP2,SERPINA5,SFN,STK4,THBS2,TJP1,WEE1	23	49
SUPT20H	other	1.01E-10	1.85E-02	Akt,CEBPB,EGFR,Hsp27,Jnk,KAT5,MAP3K7,MAPK9,MMP2,MTOR,NFkB (complex),NR3C1,P38 MAPK,PGR,PPARGC1A,RELA,RHOA,SP1,SREBF1,STAT1,SUPT20H,TP63,YAP1	ALB,APEX1,BTRC,C1R,CD1A,CERT1,CHKA,CSF2RB,CTCF,CYP7A1,DGKA,DTYMK,F3,FABP3,FABP7,FAS,FN1,HBEGF,HMOX1,HNRNPA2B1,IFNG,ITGB3,KYNU,MATK,MMP1,MMP2,MSH2,MUC1,NFATC1,NR1I3,PDHA1,PDK4,PNMT,PPARG,PPP2R1A,PTPRE,RAC1,RARA,RRM1,SEM1,SENP2,SERPINA5,SFN,STK4,THBS2,TRIM21,WARS1,WEE1	21	48
GNL1	other	1.36E-10	2.90E-03	Akt,CCND1,CDKN1A,ERK1/2,GNL1,Hsp70,Jnk,MAPK9,NFE2L2,NFkB (complex),RB1,RELA	AR,B2M,BTRC,CERT1,CTCF,CYP17A1,CYP7A1,ESR1,F3,FN1,G6PD,HBEGF,HK2,ITGB3,KYNU,MMP1,MSH2,MUC1,NCOA3,NOTCH1,PDK4,PPARG,PSAP,PTPRE,RAC1,RRM1,SEM1,SENP2,STK4,TJP1,XIAP,ZEB2	10	32
ARRB2	other	1.54E-10	5.50E-03	ADCY,Akt,ARRB2,EGFR,ERK,IGF1R,Jnk,NOS2,P38 MAPK,RELA	AR,BTRC,CDK5R1,CERT1,CTCF,DGKA,FN1,HBEGF,HK2,HNRNPA2B1,IGF1R,ITGB3,MMP1,MMP2,MSH2,MUC1,NFATC1,NOTCH1,ODC1,PPARG,PTPRE,RAC1,RARA,STK4,TJP1,TLR4,ZEB2	9	27
PIP5K1C	kinase	1.61E-10	7.40E-03	Akt,EGFR,ERK,ERK1/2,FOXO1,HIF1A,IRF3,Jnk,MAP2K1/2,Mapk,MAPK1,MTOR,P38 MAPK,PGR,PIP5K1C,RELA,RHOA,SREBF2,STAT1,YBX1	AR,B2M,BTRC,C1R,CAMK4,CDK5R1,CERT1,CHKA,CYP17A1,CYP7A1,DGKA,ERBB4,ESR1,F3,FABP7,GTF2I,HBB,HK2,HNRNPA2B1,IFNG,IGF1R,KIF11,MATK,MMP1,MMP2,MUC1,NAE1,NOTCH1,NR1I3,ODC1,PDHA1,PDK4,PNMT,PTPRE,RARA,RRM1,SERPINA5,STK4,TJP1,TRIM21,WARS1,XIAP,ZEB2	19	43
CCN1	other	1.66E-10	8.90E-03	Akt,CCN1,ERK1/2,JUN,LRP6,MMP2,NFkB (complex)	B2M,CSF2RB,CTCF,CYP17A1,CYP7A1,ESR1,F3,FAS,FN1,HBEGF,HK2,HMOX1,IFNG,ITGB3,KYNU,MMP1,MMP2,MSH2,MUC1,NOTCH1,PDK4,PPP2R1A,PTPRE,SENP2,TJP1,XIAP	7	26
ACKR3	G-protein coupled receptor	1.82E-10	2.10E-03	ACKR3,EGFR,ERK,ERK1/2	AR,B2M,CDK5R1,CYP17A1,CYP7A1,ESR1,F3,FAS,FN1,HK2,HNRNPA2B1,IGF1R,ITGB3,MMP1,MMP2,MUC1,ODC1,PDK4,PTPRE,XIAP,ZEB2	3	21
WNT5A	cytokine	1.93E-10	8.40E-03	Akt,AR,CEBPB,EGFR,ERK1/2,ESR1,FOXO1,HSF1,Hsp27,IRF3,Jnk,KAT5,MMP2,MTOR,MYC,NR3C1,P38 MAPK,PGR,RELA,RHOA,SIRT1,SREBF2,TFEB,TP63,WNT5A,YBX1	ALB,APEX1,AR,B2M,BCR,BTRC,CAMK4,CD1A,CERT1,CHKA,CSF2RB,CYP17A1,DGKA,EP300,F3,FABP7,FAP,FAS,FN1,GTF2I,HBB,HMOX1,HNRNPA2B1,HSP90AB1,IGF1R,ITGB3,KIF11,MATK,MMP1,MSH2,MUC1,NFATC1,NMRK1,NOTCH1,NR1I3,ODC1,PDHA1,PDK4,PNMT,PPP2R1A,PSAP,PTPRE,RARA,SEMA4D,SERPINA5,SMTN,STK4,THBS2,TJP1,TLR4,TTR,VPS26A,XIAP	26	53

KRAS	enzyme	2.28E-10	7.60E-03	Akt,ATM,ERK,ERK1/2,KRAS,NFkB (complex)	B2M,CDK5R1,CTCF,CYP17A1,CYP7A1,ESR1,F3,FN1,HK2,HMOX1,IFNG,IGF1R,ITGB3,KYNU,MMP1,MMP2,MSH2,MUC1,NOTCH1,ODC1,PC,PTPRE,SENP2,TJP1,XIAP,YY1	6	26
LILRB4	other	2.32E-10	2.98E-02	Akt,AR,CEBPB,CHUK,CTNNB1,EGFR,FOXO1,GLI1,GSK3B,Hsp27,IKBKB,IRF3,Jnk,KAT5,LILRB4,MMP2,MTOR,NFkB (complex),NFKBIA,NR3C1,P38 MAPK,PGR,PPARGC1A,RELA,SP1,SREBF2,STAT1,STAT3,TP63,TP73,YAP1,YBX1	ALB,ANG,ANXA6,APEX1,BTRC,C1R,CAMK4,CD1A,CERT1,CHKA,CSF2RB,CYP7A1,DTYMK,FABP7,FAS,FN1,G6PD,GTF2I,HBB,HBEGF,HMOX1,HNRNPA2B1,IFNG,IGF1R,IMPA2,ITGB3,KIF11,KLK1,KYNU,MATK,MMP1,MMP2,MSH2,MUC1,NFATC1,NOTCH1,NR1I3,PAH,PDK4,PGK1,PNMT,PPP2R1A,PTPRE,PTPRF,RAC1,RARA,RRM1,S100A6,SENP2,SERPINA5,SFN,SMTN,STK4,THBS2,TJP1,TLR4,TRIM21,WARS1,WEE1	30	59
CD6	transmembrane receptor	2.53E-10	2.50E-03	CD6,ERK1/2,Jnk,P38 MAPK	B2M,BTRC,CYP17A1,CYP7A1,DGKA,ESR1,F3,FAS,HBEGF,HK2,HMOX1,IFNG,IGF1R,ITGB3,MMP1,MMP2,MSH2,PTPRE,RARA,XIAP	4	20
MED28	other	2.53E-10	2.50E-03	ERK1/2,Jnk,MED28,P38 MAPK	B2M,BTRC,CYP17A1,CYP7A1,DGKA,ESR1,F3,FAS,HBEGF,HK2,HMOX1,IFNG,IGF1R,ITGB3,MMP1,MMP2,MSH2,PTPRE,RARA,XIAP	3	20
RNF40	enzyme	2.97E-10	1.30E-03	Akt,ERK,RNF40	CDK5R1,ESR1,F3,FAS,G6PD,HBEGF,IFNG,IGF1R,ITGB3,MMP1,MMP2,MUC1,NOTCH1,ODC1,PDK4,PPARG,TJP1	3	17
Pro-inflammatory Cytokine	group	3.27E-10	7.90E-03	ERK1/2,MTOR,NFkB (complex),P38 MAPK,Pro-inflammatory Cytokine,SMPD1,SMPD2	AR,B2M,CHKA,CTCF,CYP17A1,CYP7A1,DGKA,ESR1,F3,FAS,HBEGF,HK2,IFNG,IGF1R,ITGB3,KYNU,MMP1,MMP2,MUC1,NOTCH1,PTPRE,RARA,SENP2,XIAP	4	24
S100A9	other	3.39E-10	6.00E-03	ERK1/2,Jnk,P38 MAPK,S100A9,TERT	AR,B2M,BTRC,CYP17A1,CYP7A1,DGKA,ESR1,F3,FAS,HBEGF,HK2,HMOX1,IFNG,IGF1R,ITGB3,MMP1,MMP2,MSH2,PTPRE,RARA,XIAP	5	21
CCN6	growth factor	3.40E-10	2.16E-02	Akt,AKT1,AKT2,CCN6,CEBPB,ERBB2,ERK,FOXO1,Hsp27,IRF3,Jnk,KAT5,MAP3K7,MAPK1,MAPK9,MMP2,MTOR,NFkB (complex),NR3C1,P38 MAPK,PGR,PPARGC1A,RELA,RHOA,SREBF1,STAT1,TP63,YAP1	ALB,APEX1,BMP1,BTRC,C1R,CAMK4,CD1A,CDK5R1,CERT1,CHKA,CSF2RB,CYP7A1,DGKA,DTYMK,ERBB4,ESR1,F3,FABP3,FABP7,FAS,FN1,GART,HBB,HMOX1,ITGB3,KIF11,KYNU,MATK,MMP1,MMP2,MSH2,NFATC1,NR1I3,ODC1,PDHA1,PDK4,PGK1,PNMT,PPP2R1A,PTPRF,RAC1,RARA,RRM1,SEM1,SENP2,SERPINA5,SFN,SMTN,STK4,THBS2,TRIM21,WEE1	26	52
INS	other	4.09E-10	8.10E-03	Akt,AKT1,ERK,ERK1/2,GATA2,Gsk3,INS,INSR,Jnk,Mapk,MAPK1,MTOR,PI3K (complex),RPS6KB1,YAP1	B2M,BTRC,CDK5R1,CHKA,CYP17A1,CYP7A1,DTYMK,EPHA3,ESR1,F3,FAS,FN1,HBB,HK2,HMOX1,IFNG,ITGB3,MMP1,MMP2,MSH2,MUC1,NOTCH1,NR1I3,ODC1,PPARG,PTPRE,RARA,RRM1,TJP1,TRIM21,XIAP,ZIC3	15	32
IFNG	cytokine	4.51E-10	3.33E-02	Akt,ERK1/2,IFNG,IRF1,JAK2,MMP2,MTOR,NFkB (complex),P38 MAPK,PRKAA1,RELA,STAT1,STAT3,TP73	C1R,CAMK4,CERT1,CHKA,CSF2RB,CTCF,CYP17A1,CYP7A1,DGKA,DPP4,EPHA3,ESR1,FAS,G6PD,GART,GRK2,HK2,ITGB3,KYNU,MAGEA4,MMP1,MMP2,MUC1,NOTCH1,ODC1,PDK4,PGK1,PPARG,PPP2R1A,PTPRE,RARA,SENP2,SERPINA5,SFN,STK4,TJP1,TLR4,TRIM21,WARS1,XIAP,ZEB2	14	41

PTPRK	phosphatase	4.69E-10	1.00E-03	EGFR,ERK,PTPRK	AR,CDK5R1,ESR1,F3,FAS,HBEGF,HK2,HNRNPA2B1,ITGB3,MMP1,MMP2,MUC1,ODC1,PDK4,PTPRE,XIAP,ZEB2	2	17
G protein alphai	group	4.69E-10	2.94E-02	ADCY,AR,CEBPA,EGFR,ERK,ERK1/2,FOXO1,G protein alphai,GPIIB-IIIA,HIF1A,Jnk,MAP2K1/2,Mapk,MAPK1,MED1,MMP2,MTOR,NFkB (complex),P38 MAPK,PGR,RELA,RHOA,SP1,STAT3,STK4	ALB,AR,B2M,BTRC,CAMK4,CDK5R1,CHKA,CSF2RB,CYP17A1,CYP7A1,DGKA,DTYMK,EPHA3,ERBB4,ESR1,F3,FN1,HBB,HBEGF,HK2,HNRNPA2B1,IFNG,IGF1R,ITGB3,KIF11,KYNU,MATK,MMP1,MMP2,NAE1,NFATC1,NR1I3,ODC1,PDHA1,PGK1,PNMT,PPP2R1A,PTPRE,RARA,RRM1,SENP2,SERPINA5,SMTN,STK4,TLR4,TRIM21,WEE1,XIAP,ZEB2	22	49
EDA	cytokine	5.13E-10	1.00E-03	EDA,EGFR,ERK	AR,CDK5R1,ESR1,F3,FAS,HBEGF,HK2,HNRNPA2B1,ITGB3,MMP1,MMP2,MUC1,ODC1,PDK4,PTPRE,XIAP,ZEB2	2	17
OGT	enzyme	5.18E-10	1.21E-02	Akt,ERK1/2,NFkB (complex),OGT,PRKAA,RELA,SREBF1,YAP1	AR,B2M,CERT1,CYP17A1,CYP7A1,DTYMK,ESR1,F3,FABP3,FABP7,FAS,FN1,HBEGF,HK2,HMOX1,IFNG,KYNU,MMP2,MSH2,MUC1,NOTCH1,PDK4,PPARG,PTPRE,RRM1,SENP2,STK4,TJP1,XIAP	8	29
mir-27	microRNA	5.40E-10	5.00E-03	ERK1/2,mir-27,NFkB (complex)	B2M,CTCF,CYP17A1,CYP7A1,ESR1,FAS,FN1,HBEGF,HK2,HMOX1,IFNG,IGF1R,KYNU,MMP1,MMP2,MSH2,NOTCH1,PSAP,PTPRE,SENP2,XIAP	3	21
PLAUR	transmembrane receptor	5.98E-10	4.40E-03	AKT1,ERK,ERK1/2,MAPK1,MMP2,PLAUR	AR,B2M,CDK5R1,CSF2RB,CYP17A1,CYP7A1,ESR1,FAS,FN1,HBB,HK2,HMOX1,IFNG,IGF1R,ITGB3,MMP1,MMP2,MUC1,ODC1,PDK4,PPP2R1A,PTPRE,TJP1,TRIM21,XIAP	5	25
ANXA6	ion channel	6.12E-10	1.10E-03	ANXA6,EGFR,ERK	AR,CDK5R1,ESR1,F3,FAS,HBEGF,HK2,HNRNPA2B1,ITGB3,MMP1,MMP2,MUC1,ODC1,PDK4,PTPRE,XIAP,ZEB2	2	17
SOS1	other	6.15E-10	5.90E-03	ERK1/2,NFkB (complex),RAC1,RHOA,SOS1	B2M,CTCF,CYP17A1,CYP7A1,ESR1,FAS,FN1,HBEGF,HK2,HMOX1,IFNG,IGF1R,KYNU,MMP1,MMP2,MSH2,NOTCH1,PDHA1,PTPRE,SENP2,XIAP	4	21
RAD51AP1-DYRK4	fusion gene/product	6.20E-10	2.03E-02	Akt,ATR,CEBPA,EGFR,ERBB2,ERK,ERK1/2,ESR1,FOXO1,GSK3B,IKBKB,Jnk,MAP2K1/2,MAPK1,MED1,MMP2,NFkB (complex),RAD51AP1-DYRK4,SREBF2,STAT1,STAT5a/b,YBX1	ALB,AR,B2M,BMP1,BTRC,C1R,CAMK4,CDK5R1,CSF2RB,CYP17A1,CYP7A1,DGKA,DTYMK,EPHA3,ERBB4,ESR1,F3,FABP7,FAP,FN1,GART,GTF2I,HBB,HBEGF,HK2,HNRNPA2B1,IGF1R,KIF11,KYNU,MSH2,MUC1,NOTCH1,ODC1,PDK4,PGK1,PPARG,PPP2R1A,PTPRE,PTPRF,RAC1,RARA,RBM39,SENP2,SMTN,TJP1,TRIM21,WARS1	21	47
BDNF	growth factor	6.63E-10	9.60E-03	Akt,BDNF,ERK1/2,NFkB (complex),RELA,SRC	B2M,CERT1,CYP17A1,CYP7A1,ESR1,F3,FAS,FN1,HBEGF,HK2,HMOX1,IFNG,KYNU,MMP1,MMP2,MSH2,MUC1,NOTCH1,PDK4,PPARG,PTPRE,SENP2,STK4,TJP1,XIAP	5	25
INHBA	growth factor	6.68E-10	1.71E-02	Akt,CEBPA,CEBPB,EGFR,ERK1/2,ERN1,GSK3B,Hsp27,INHBA,Jnk,KAT5,MED1,M	ALB,ANG,APEX1,B2M,BCR,BTRC,CD1A,CYP17A1,CYP7A1,DGKA,DTYMK,EPHA3,ERBB4,ESR1,F3,FABP7,FAP,FAS,GTF2I,HBB,HBEGF,HK2,HMO	24	49

				YC,NR3C1,P38 MAPK,PGR,SMAD2,SMAD3,SREBF2,STAT3,TFEB,TP63,YAP1,YBX1	X1,HNRNPA2B1,IFNG,ITGB3,MATK,MSH2,MUC1,NFATC1,NOTCH1,NR1I3,ODC1,PDK4,PGK1,PNMT,PSAP,PTPRE,RAC1,RARA,RRM1,SERPINA5,SFN,THBS2,TJP1,VAV3,VPS26A,WEE1,XIAP		
PP2A	complex	6.89E-10	4.80E-03	Akt,ERK1/2,MAPK8,NFkB (complex),PP2A,PRKAA,STAT5a/b	AR,B2M,CTCF,CYP17A1,CYP7A1,ESR1,F3,FAS,FN1,HBEGF,HK2,IFNG,IGF1R,KYNU,MSH2,MUC1,NOTCH1,PDK4,PTPRE,RARA,SENP2,TJP1,XIAP	6	23
G protein alphai	group	7.00E-10	8.00E-04	ADCY,EGFR,ERK1/2,G protein alphai	AR,B2M,CYP17A1,CYP7A1,ESR1,F3,FN1,HBEGF,HK2,HNRNPA2B1,IFNG,IGF1R,MMP1,MMP2,NFATC1,PTPRE,XIAP,ZEB2	3	18
ERRFI1	other	7.35E-10	3.90E-03	Akt,EGFR,ERRFI1,FOXO1,GSK3B,MET	AR,CAMK4,ESR1,F3,FN1,HK2,HMOX1,HNRNPA2B1,IFNG,IGF1R,KIF11,MMP1,MUC1,NOTCH1,PDK4,PTPRE,RAC1,TJP1,XIAP,ZEB2	5	20
CXCR4	G-protein coupled receptor	7.68E-10	8.10E-03	Akt,AKT1,CXCR4,ERK1/2,Jnk,MAPK1,P38 MAPK,Ras homolog,RHOA,ZAP70	AR,B2M,BTRC,CYP17A1,CYP7A1,DGKA,ESR1,F3,FAS,HBB,HBEGF,HK2,HMOX1,IFNG,ITGB3,MMP1,MMP2,MSH2,MUC1,NOTCH1,PDHA1,PDK4,PTPRE,RARA,TJP1,TRIM21,XIAP	10	27
POSTN	other	7.76E-10	3.50E-03	Akt,EGFR,ERK,POSTN	AR,CDK5R1,ESR1,FAS,HBEGF,HK2,HNRNPA2B1,IGF1R,ITGB3,MMP1,MMP2,MUC1,NOTCH1,ODC1,PTPRE,TJP1,VEGFB,XIAP,ZEB2	4	19
WWOX	enzyme	8.42E-10	5.50E-03	ERBB4,ERK,GSK3B,NFkB (complex),WWOX	CDK5R1,CTCF,ESR1,HBEGF,HMOX1,IFNG,IGF1R,ITGB3,KYNU,MMP1,MMP2,MSH2,MUC1,NOTCH1,ODC1,PDK4,PGK1,RAC1,SENP2,XIAP	5	20
SERPINC1	enzyme	9.37E-10	6.50E-03	ERK1/2,F10,F2,Hsp27,JAK2,MAPK1,MAPK8,NFkB (complex),P38 MAPK,PRKAA1,PRKCQ,PTK2B,RHOA,SERPINC1,SRC (family),VSIR	AR,B2M,CD1A,CTCF,CYP17A1,CYP7A1,DGKA,EPHA3,ESR1,F3,FN1,HBB,HBEGF,HK2,HMOX1,IFNG,ITGB3,KYNU,MAGEA4,MMP1,MMP2,MSH2,NOTCH1,PDHA1,PTPRE,RARA,SENP2,TJP1,TRIM21,XIAP	15	30
SMAD7	transcription regulator	9.54E-10	2.18E-02	Akt,AKT1,CASP8,CEBPB,ERBB2,ERK,ERK1/2,ERN1,FOXO1,Hsp27,IRF3,Jnk,KAT5,MAP2K3,MAP3K7,MAPK1,MAPK9,Mek,MMP2,MTOR,NR3C1,P38 MAPK,PDPK1,PGR,RELA,RHOA,SMAD2,SMAD7,SREBF1,STAT1,TP63,YAP1	ALB,ANG,APEX1,B2M,BMP1,BTRC,C1R,CAMK4,CD1A,CDK5R1,CERT1,CHKA,CSF2RB,CYP17A1,DGKA,DTYMK,ERBB4,F3,FABP3,FABP7,FAS,GART,HBB,HMOX1,IFNG,ITGB3,KIF11,MATK,MMP1,MSH2,NFATC1,NR1I3,ODC1,PDHA1,PDK4,PGK1,PNMT,PPP2R1A,PTPRF,RAC1,RARA,RRM1,SEM1,SERPINA5,SFN,SMTN,STK4,THBS2,TRIM21,WARS1,WEE1,XIAP	28	52
ADCY	group	9.61E-10	3.40E-03	ADCY,ERK,ERK1/2	B2M,CDK5R1,CYP17A1,CYP7A1,ESR1,FAS,FN1,HK2,IFNG,IGF1R,ITGB3,MMP1,MMP2,MUC1,NFATC1,ODC1,PDK4,PTPRE	3	18
EVL	other	9.63E-10	3.40E-03	Akt,ERK1/2,EVL,Focal adhesion kinase,NANOG,P38 MAPK,PXN	B2M,CYP17A1,CYP7A1,DGKA,ESR1,FAS,FN1,HBEGF,HK2,HMOX1,IFNGR1,IGF1R,ITGB3,MSH2,MUC1,NOTCH1,PDK4,PTPRE,RARA,S100A6,TJP1,XIAP	4	22

GAS6	growth factor	9.64E-10	6.30E-03	Akt,AXL,EGFR,ERK,GAS6,MET,SRC (family)	AR,CDK5R1,ESR1,FAS,FN1,HBEGF,HK2,HMOX1,HNRNPA2B1,IGF1R,ITGB3,MMP1,MMP2,MUC1,NOTCH1,ODC1,PTPRE,TJP1,XIAP,ZEB2	6	20
STAB2	transmembrane receptor	9.64E-10	5.70E-03	ERK1/2,NFkB (complex),STAB2	B2M,CTCF,CYP17A1,CYP7A1,ESR1,FAS,FN1,HBEGF,HK2,HMOX1,IFNG,IGF1R,KYNU,MMP1,MMP2,MSH2,NOTCH1,PTPRE,SENP2,XIAP	2	20
RPSA	translation regulator	9.95E-10	1.10E-03	ERK,MAPK8,MMP2,RHOA,RPSA	CDK5R1,CSF2RB,ESR1,FAS,HBEGF,IFNG,ITGB3,MMP1,MMP2,MUC1,ODC1,PDHA1,PDK4,PPP2R1A,RARA,TJP1	5	16
IL36G	cytokine	1.02E-09	9.60E-03	ERK1/2,IL36G,Jnk,NFkB (complex)	B2M,BTRC,CD1A,CTCF,CYP17A1,CYP7A1,ESR1,FAS,FN1,HBEGF,HK2,HMOX1,IFNG,IGF1R,KYNU,MMP1,MMP2,NOTCH1,PTPRE,RARA,SENP2	4	21
HBEGF	growth factor	1.02E-09	1.56E-02	EGFR,ERBB2,ERBB4,ERK,ERK1/2,HBEGF,Mapk,P38 MAPK	AR,B2M,BMP1,CDK5R1,CYP17A1,CYP7A1,DGKA,ERBB4,ESR1,GART,HK2,HMOX1,HNRNPA2B1,IGF1R,ITGB3,MMP1,MMP2,MSH2,MUC1,NR1I3,ODC1,PDK4,PGK1,PPARG,PTPRE,PTPRF,RARA,RRM1,SMTN,XIAP,ZEB2	8	31
P2RY6	G-protein coupled receptor	1.03E-09	6.00E-03	ERK1/2,NFkB (complex),P2RY6	B2M,CTCF,CYP17A1,CYP7A1,ESR1,FAS,FN1,HBEGF,HK2,HMOX1,IFNG,IGF1R,KYNU,MMP1,MMP2,MSH2,NOTCH1,PTPRE,SENP2,XIAP	2	20
IL36B	cytokine	1.09E-09	9.50E-03	ERK1/2,IL36B,Jnk,NFkB (complex)	B2M,BTRC,CD1A,CTCF,CYP17A1,CYP7A1,ESR1,FAS,FN1,HBEGF,HK2,HMOX1,IFNG,IGF1R,KYNU,MMP1,MMP2,NOTCH1,PTPRE,RARA,SENP2	4	21
FURIN	peptidase	1.15E-09	6.90E-03	AKT1,ERK1/2,FURIN,MMP9,P38 MAPK,RELA,SMAD2,SMAD3	B2M,CERT1,CTCF,CYP17A1,CYP7A1,DGKA,ESR1,F3,FAS,HBEGF,HK2,HMOX1,IGF1R,ITGB3,MMP1,MMP2,MSH2,PPARG,PTPRE,RAC1,RARA,STK4,TJP1,XIAP	7	24
PTK2	kinase	1.22E-09	9.70E-03	Akt,ERK,IGF1R,LCK,MAPK8,MET,PTEN,PTK2,RHOA,YAP1	AR,CDK5R1,DTYMK,F3,FAS,FN1,HBEGF,HMOX1,IFNG,IGF1R,ITGB3,MMP1,MMP2,MUC1,NOTCH1,ODC1,PDHA1,PPARG,RAC1,RARA,RRM1,TJP1	9	22
PIP5K1C	kinase	1.31E-09	1.80E-03	Akt,EGFR,PIP5K1C	AR,ESR1,F3,HK2,HNRNPA2B1,IFNG,IGF1R,MMP1,MMP2,MUC1,NOTCH1,PDK4,PTPRE,TJP1,XIAP,ZEB2	2	16
ITGA3	other	1.31E-09	4.30E-03	ERK,ERK1/2,ITGA3,RHOA,SRC	B2M,CDK5R1,CYP17A1,CYP7A1,ESR1,FAS,FN1,HK2,IFNG,IGF1R,ITGB3,MMP1,MMP2,MUC1,ODC1,PDHA1,PDK4,PTPRE	4	18
NOX5	ion channel	1.34E-09	1.00E-02	Akt,ERK1/2,NOX5,P38 MAPK,RELA,STAT5a/b	B2M,CERT1,CTCF,CYP17A1,CYP7A1,DGKA,ESR1,FAS,HBEGF,HK2,HMOX1,IFNG,ITGB3,MMP1,MMP2,MSH2,MUC1,NOTCH1,PDK4,PPARG,PTPRE,RARA,STK4,TJP1	5	24
S1PR2	G-protein coupled receptor	1.42E-09	8.30E-03	Akt,EGFR,ERK1/2,RELA,S1PR2	AR,B2M,CERT1,CTCF,CYP17A1,CYP7A1,ESR1,FAS,FN1,HBEGF,HK2,HNRNPA2B1,MMP1,MMP2,MUC1,NOTCH1,PDK4,PPARG,PTPRE,STK4,TJP1,XIAP,ZEB2	4	23

VTN	other	1.48E-09	5.60E-03	Akt,ERK1/2,MTOR,VTN	AR,B2M,CHKA,CYP17A1,CYP7A1,ESR1,F3,FN1,HBEGF,HK2,IFNG,MMP1,MMP2,MUC1,NOTCH1,PDK4,PTPRE,SFN,TJP1	4	19
NR5A2	ligand-dependent nuclear receptor	1.50E-09	2.52E-02	ERK1/2,ERN1,FOXO1,NFkB (complex),NR5A2,PRKAA,SMAD2,SMAD3,SREBF1,SREBF2	ANG,AR,B2M,CAMK4,CTCF,CYP17A1,CYP7A1,ESR1,FABP3,FABP7,FAS,FN1,GTF2I,HBEGF,HK2,IFNG,IGF1R,KIF11,KYNU,MMP1,MMP2,MSH2,NOTCH1,PPARG,PTPRE,RAC1,SENP2,XIAP	9	28
PDE4A	enzyme	1.52E-09	3.70E-03	ERK1/2,P38 MAPK,PDE4A	B2M,CYP17A1,CYP7A1,DGKA,ESR1,F3,FAS,FN1,HBEGF,HK2,HMOX1,IGF1R,ITGB3,MMP1,MMP2,MSH2,PTPRE,RARA	3	18
A2M	transporter	1.53E-09	9.30E-03	A2M,Akt,ERK1/2,MAP2K1/2,MMP2,P38 MAPK	B2M,CSF2RB,CYP17A1,CYP7A1,DGKA,ESR1,FAS,HBEGF,HK2,HMOX1,ITGB3,MMP1,MMP2,MSH2,MUC1,NOTCH1,PDK4,PPP2R1A,PTPRE,RARA,RRM1,XIAP	6	22
MAP3K8	kinase	1.68E-09	1.10E-02	CHUK,ERK1/2,MAP2K1,MAP3K8,NFkB (complex)	B2M,CTCF,CYP17A1,CYP7A1,ESR1,F3,FAS,FN1,HBEGF,HK2,HMOX1,IFNG,IGF1R,KYNU,MMP1,MMP2,MSH2,NOTCH1,PTPRE,SENP2,XIAP	4	21
PRTN3	peptidase	1.97E-09	7.20E-03	CXCL8,ERK1/2,IL1B,PRTN3	ACP3,AR,B2M,BTRC,CD74,CYP17A1,CYP7A1,DPP4,ESR1,F3,FAS,FN1,HBEGF,HK2,IFNG,IGF1R,MMP1,MMP2,MUC1,PPARG,PTPRE,RAC1,TLR4	3	23
VDAC1	ion channel	1.98E-09	1.62E-02	CASP8,CASP9,ERK1/2,IL1B,Inflammasome (Nalp3, Asc, Casp1),MAPK8,NFkB (complex),P38 MAPK,VDAC1	ACP3,B2M,BTRC,CTCF,CYP17A1,CYP7A1,DGKA,DPP4,ESR1,F3,FAS,HBEGF,HK2,IFNG,IGF1R,ITGB3,KYNU,MMP1,MMP2,MUC1,NOTCH1,PPARG,PTPRE,RARA,SENP2,TLR4,XIAP	5	27
BAX	transporter	2.07E-09	1.83E-02	BAX,CASP8,CASP9,ERK1/2,IRF3,MAPK8,NFkB (complex),P38 MAPK	B2M,CTCF,CYP17A1,CYP7A1,DGKA,ESR1,F3,FAS,FN1,HBEGF,HK2,IFNG,IGF1R,ITGB3,KYNU,MMP1,MMP2,NOTCH1,PTPRE,RARA,SENP2,STK4,XIAP	5	23
LAIR1	transmembrane receptor	2.09E-09	4.90E-03	ERK,LAIR1,MAPK9,P38 MAPK	CDK5R1,DGKA,ESR1,F3,FN1,HBEGF,HMOX1,IFNG,ITGB3,MMP1,MMP2,MSH2,MUC1,ODC1,PDK4,PPARG,RAC1,RARA,SEM1	4	19
HRAS	enzyme	2.09E-09	8.40E-03	CHUK,ERK,ERK1/2,HRAS,IKBKB	B2M,CDK5R1,CYP17A1,CYP7A1,DGKA,ESR1,FAS,FN1,HK2,HMOX1,IFNG,IGF1R,ITGB3,MMP1,MMP2,NOTCH1,ODC1,PDK4,PTPRE	5	19
SMPD2	enzyme	2.11E-09	9.10E-03	ERK1/2,NFkB (complex),SMPD2	B2M,CTCF,CYP17A1,CYP7A1,ESR1,FAS,FN1,HBEGF,HK2,HMOX1,IFNG,IGF1R,KYNU,MMP1,MMP2,MSH2,NOTCH1,PTPRE,SENP2,XIAP	2	20
KITLG	growth factor	2.16E-09	2.82E-02	Akt,ERK1/2,JUN,KITLG,Mapk,MAPK8,MTOR,P38 MAPK,RELA,RPS6KB1,STAT3,TERT	AR,B2M,CERT1,CHKA,CTCF,CYP17A1,CYP7A1,DGKA,ESR1,FAS,HBEGF,HK2,HMOX1,IFNG,ITGB3,MMP1,MMP2,MSH2,MUC1,NOTCH1,NR1I3,PDK4,PGK1,PPARG,PRKCB,PTPRE,RARA,STK4,TJP1	12	29

Ggtase I	complex	2.16E-09	1.51E-02	Akt,AKT1,AR,ERBB4,ERK1/2,FOXO1,Gg tase I,HTRA2,Jnk,LATS1,PLK1,RELA,STK3,STK4,YAP1	B2M,BTRC,CAMK4,CERT1,CTCF,CYP17A1,CYP7A1,DTYMK,F3,FAS,FN1,HBEGF,HK2,HMOX1,KIF11,MSH2,MUC1,NOTCH1,PDK4,PGK1,PPARG,PTPRE,RARA,RRM1,SFN,SMTN,TJP1,TLR4,WEE1,XIAP	12	30
PDE4D	enzyme	2.21E-09	5.10E-03	ERK1/2,P38 MAPK,PDE4D	B2M,CYP17A1,CYP7A1,DGKA,ESR1,F3,FAS,FN1,HBEGF,HK2,HMOX1,IGF1R,ITGB3,MMP1,MMP2,MSH2,PTPRE,RARA	3	18
GALNT4	enzyme	2.29E-09	2.69E-02	ERK1/2,ERN1,FOXO1,GALNT4,NFkB (complex),PRKAA,SMAD2,SMAD3	ANG,AR,B2M,CAMK4,CTCF,CYP17A1,CYP7A1,ESR1,FAS,FN1,HBEGF,HK2,IFNG,IGF1R,KIF11,KYNU,MMP1,MMP2,MSH2,NOTCH1,PTPRE,RAC1,SENP2,TJP1,XIAP	8	25
Mmp	group	2.37E-09	1.61E-02	EGFR,ERK,ERK1/2,Mmp,TGFB1	ALB,AR,B2M,BMP1,CDK5R1,CYP17A1,CYP7A1,ESR1,F3,FAP,FAS,HK2,HMOX1,HNRNPA2B1,IGF1R,ITGB3,MMP2,MUC1,ODC1,PDK4,PTPRE,SMTN,SMURF2,TJP1,XIAP,ZEB2	5	26
ITSN1	other	2.40E-09	1.14E-02	ERK,ERK1/2,ITSN1,MAP2K1/2,P38 MAPK	B2M,CDK5R1,CYP17A1,CYP7A1,DGKA,ESR1,F3,HK2,HMOX1,IFNG,IGF1R,ITGB3,MMP1,MMP2,MSH2,MUC1,ODC1,PDK4,PTPRE,RARA,RRM1	4	21
CIB1	other	2.44E-09	3.40E-03	Akt,CIB1,ERK,MAP3K5,PAK1	CDK5R1,ESR1,F3,FAS,FN1,HBEGF,IFNG,IGF1R,ITGB3,MMP1,MMP2,MSH2,MUC1,NOTCH1,ODC1,TJP1	4	16
CDKN1A	kinase	2.44E-09	1.15E-02	CCND1,CDKN1A,ERK1/2,Jnk,MAPK9,NFE2L2,NFkB (complex),RB1,RELA	AR,B2M,BTRC,CERT1,CTCF,CYP17A1,CYP7A1,ESR1,FN1,G6PD,HBEGF,HK2,IGF1R,ITGB3,KYNU,MMP1,MSH2,NCOA3,NOTCH1,PPARG,PSAP,PTPRE,RAC1,RRM1,SEM1,SENP2,STK4,XIAP,ZEB2	9	29
SULF2	enzyme	2.53E-09	1.99E-02	Akt,CEBPA,ERK1/2,ERN1,Jnk,MED1,MMP2,NFkB (complex),SMAD2,SMAD3,SREBF2,STAT1,SULF2,YBX1	ALB,ANG,B2M,BTRC,C1R,CSF2RB,CYP17A1,CYP7A1,DTYMK,EPHA3,ESR1,F3,FABP7,FN1,GTF2I,HBB,HBEGF,HK2,IFNG,IGF1R,KYNU,MMP1,MSH2,MUC1,NOTCH1,PDK4,PPP2R1A,PTPRE,RAC1,RARA,SENP2,TJP1,TRIM21,WARS1,XIAP	13	35
SYK/ZAP	group	2.55E-09	4.60E-03	ERK1/2,P38 MAPK,RPS6KB1,SYK/ZAP	B2M,CYP17A1,CYP7A1,DGKA,ESR1,F3,FAS,HBEGF,HK2,HMOX1,IGF1R,ITGB3,MMP1,MMP2,MSH2,MUC1,PTPRE,RARA	3	18
RAP1A	enzyme	2.55E-09	9.80E-03	ERK,ERK1/2,P38 MAPK,RAC1,RAP1A	B2M,CDK5R1,CYP17A1,CYP7A1,DGKA,ESR1,F3,HK2,HMOX1,IFNG,IGF1R,ITGB3,MMP1,MMP2,MSH2,MUC1,ODC1,PDK4,PTPRE,RARA	4	20
G6PD	enzyme	2.59E-09	1.30E-02	ERK1/2,G6PD,NFkB (complex),P38 MAPK,RELA	B2M,CERT1,CYP17A1,CYP7A1,DGKA,ESR1,F3,FAS,HBEGF,HK2,IFNG,IGF1R,ITGB3,KYNU,MMP1,MMP2,NOTCH1,PPARG,PTPRE,RAC1,RARA,SENP2,STK4,XIAP	5	24
ADAM10	peptidase	2.70E-09	6.90E-03	ADAM10,EGFR,ERK1/2,MDM2,P38 MAPK,Ras homolog	AR,B2M,CYP17A1,CYP7A1,DGKA,ESR1,F3,FAS,HBEGF,HK2,HNRNPA2B1,IFNG,ITGB3,MMP1,MSH2,PTPRE,RARA,SFN,TJP1,XIAP,ZEB2	5	21

GAB1	other	2.70E-09	2.06E-02	Akt,ERK,ERK1/2,GAB1,NFkB (complex),SRC	B2M,CDK5R1,CTCF,CYP17A1,CYP7A1,ESR1,F3,FN1,HK2,HMOX1,IFNG,ITGB3,KYNU,MMP1,MMP2,MSH2,MUC1,NOTCH1,ODC1,PTPRE,SENP2,TJP1,XIAP	5	23
AGK	kinase	2.73E-09	3.30E-03	AGK,EGFR,ERK1/2	AR,B2M,CYP17A1,CYP7A1,ESR1,F3,FN1,HBEGF,HK2,HNRNPA2B1,IFNG,IGF1R,MMP1,MMP2,PTPRE,XIAP,ZEB2	2	17
FCHSD2	other	2.74E-09	6.30E-03	Akt,EGFR,ERK1/2,FCHSD2	AR,B2M,CYP17A1,CYP7A1,ESR1,FN1,HBEGF,HK2,HNRNPA2B1,IFNG,MMP1,MMP2,MUC1,NOTCH1,PDK4,PTPRE,TJP1,XIAP,ZEB2	3	19
PACSIN2	transporter	2.74E-09	6.30E-03	Akt,EGFR,ERK1/2,PACSIN2	AR,B2M,CYP17A1,CYP7A1,ESR1,FN1,HBEGF,HK2,HNRNPA2B1,IFNG,MMP1,MMP2,MUC1,NOTCH1,PDK4,PTPRE,TJP1,XIAP,ZEB2	3	19
HMGB1	transcription regulator	2.90E-09	2.67E-02	ERK1/2,HIF1A,HMGB1,IRF3,Jnk,MAPK8,P38 MAPK,RAC1	B2M,BTRC,CYP17A1,CYP7A1,DGKA,ERBB4,ESR1,F3,FAS,HBEGF,HK2,HLA-DRB1,HMOX1,IFNG,IGF1R,ITGB3,MMP1,MMP2,MSH2,MUC1,NAE1,PTPRE,RARA,STK4,XIAP	8	25
EREG	growth factor	2.90E-09	2.40E-03	Akt,EGFR,EREG	AR,ESR1,HBEGF,HK2,HNRNPA2B1,IFNG,IGF1R,MMP1,MMP2,MUC1,NOTCH1,PDK4,PTPRE,TJP1,XIAP,ZEB2	3	16
TLR10	transmembrane receptor	2.95E-09	7.60E-03	ERK,Jnk,P38 MAPK,TLR10	BTRC,CDK5R1,DGKA,ESR1,F3,FN1,HBEGF,HMOX1,IFNG,ITGB3,MMP1,MMP2,MSH2,MUC1,ODC1,PDK4,RARA,XIAP	3	18
MAP3K20	kinase	2.95E-09	7.60E-03	ERK,Jnk,MAP3K20,P38 MAPK	BTRC,CDK5R1,DGKA,ESR1,F3,FN1,HBEGF,HMOX1,IFNG,ITGB3,MMP1,MMP2,MSH2,MUC1,ODC1,PDK4,RARA,XIAP	3	18
ADAM9	peptidase	2.95E-09	3.40E-03	ADAM9,EGFR,ERK1/2	AR,B2M,CYP17A1,CYP7A1,ESR1,F3,FN1,HBEGF,HK2,HNRNPA2B1,IFNG,IGF1R,MMP1,MMP2,PTPRE,XIAP,ZEB2	2	17
SNX1	transporter	2.95E-09	3.30E-03	EGFR,ERK1/2,SNX1	AR,B2M,CYP17A1,CYP7A1,ESR1,F3,FN1,HBEGF,HK2,HNRNPA2B1,IFNG,IGF1R,MMP1,MMP2,PTPRE,XIAP,ZEB2	2	17
GAPDH	enzyme	2.99E-09	4.33E-02	Akt,CEBPB,EGFR,EIF2AK2,GAPDH,Hsp27,IRF3,Jnk,KAT5,MMP2,MTOR,NFkB (complex),NR3C1,P38 MAPK,PGR,PPARGC1A,RPS6KB1,SP1,STAT1,STAT3,TP63,YAP1	ALB,APEX1,BTRC,C1R,CD1A,CHKA,CSF2RB,CTCF,CYP7A1,DGKA,DTYMK,F3,FAS,FN1,HBEGF,HMOX1,HNRNPA2B1,ITGB3,KYNU,MATK,MMP1,MMP2,MSH2,MUC1,NFATC1,NR1I3,PDK4,PGK1,PNMT,PPARG,PPP2R1A,PTPRE,RARA,RRM1,SENP2,SERPINA5,SFN,STK4,THBS2,TRIM21,WARS1,WEE1,ZNF292	22	43
HCG11	other	3.17E-09	7.60E-03	ERK,HCG11,Jnk,P38 MAPK	BTRC,CDK5R1,DGKA,ESR1,F3,FN1,HBEGF,HMOX1,IFNG,ITGB3,MMP1,MMP2,MSH2,MUC1,ODC1,PDK4,RARA,XIAP	3	18
CG	complex	3.18E-09	2.90E-02	ADCY,Akt,CG,EGFR,ERK1/2,MAPK1,MAPK8,Mek,MMP2,NFkB (complex)	ACP3,AR,B2M,C1R,CDK5R1,CSF2RB,CTCF,CYP7A1,FAS,FN1,HBB,HBEGF,HK2,HMOX1,HNRNPA2B1,IFNG,IFNGR1,ITGB3,KYNU,MMP1,MMP2,MS	10	33

					H2,MUC1,NFATC1,PDK4,PPP2R1A,PTPRE,RARA,SENP2,SMURF2,TRIM21,XIAP,ZEB2		
FRY	other	3.18E-09	2.28E-02	ERBB4,ERK1/2,FRY,MYC,P38 MAPK,RELA,STK38,YAP1	B2M,BCR,CERT1,CTCF,CYP17A1,CYP7A1,DGKA,DTYMK,ESR1,F3,FAP,FAS,HBEGF,HK2,HMOX1,IFNG,IGF1R,ITGB3,MMP1,MMP2,MSH2,ODC1,PGK1,PPARG,PTPRE,RARA,RRM1,STK4	6	28
HTATIP2	transcription regulator	3.19E-09	3.70E-03	EGFR,ERK1/2,HTATIP2	AR,B2M,CYP17A1,CYP7A1,ESR1,F3,FN1,HBEGF,HK2,HNRNPA2B1,IFNG,IGF1R,MMP1,MMP2,PTPRE,XIAP,ZEB2	3	17
GRP	growth factor	3.19E-09	3.60E-03	EGFR,ERK1/2,GRP	AR,B2M,CYP17A1,CYP7A1,ESR1,F3,FN1,HBEGF,HK2,HNRNPA2B1,IFNG,IGF1R,MMP1,MMP2,PTPRE,XIAP,ZEB2	2	17
Gpcr	group	3.19E-09	3.70E-03	EGFR,ERK1/2,Gpcr,PRKD1	AR,B2M,CYP17A1,CYP7A1,ESR1,F3,FN1,HBEGF,HK2,HNRNPA2B1,IFNG,IGF1R,MMP1,MMP2,PTPRE,XIAP,ZEB2	3	17
KL	enzyme	3.20E-09	2.02E-02	Akt,CEBPA,ERK1/2,FOXO1,GSK3B,Jnk,KL,MED1,MMP2,NFkB (complex),SREBF2,STAT1,YBX1	ALB,B2M,BTRC,C1R,CAMK4,CSF2RB,CYP17A1,CYP7A1,DTYMK,EPHA3,ESR1,F3,FABP7,FN1,GTF2I,HBB,HBEGF,HK2,IGF1R,KIF11,KYNU,MMP1,MSH2,MUC1,NOTCH1,PDK4,PPP2R1A,PTPRE,RAC1,RARA,SENP2,TJP1,TRIM21,WARS1	12	34
TFF2	other	3.43E-09	4.50E-03	ERK1/2,Jnk,TFF2	B2M,BTRC,CYP17A1,CYP7A1,ESR1,FN1,HBEGF,HK2,IFNG,IGF1R,MMP1,MMP2,MSH2,PTPRE,RARA,XIAP	2	16
IL17RD	other	3.44E-09	5.60E-03	ERK,ERK1/2,IL17RD	B2M,CDK5R1,CYP17A1,CYP7A1,ESR1,FAS,FN1,HK2,IFNG,IGF1R,ITGB3,MMP1,MMP2,MUC1,ODC1,PDK4,PTPRE	2	17
TP63	transcription regulator	3.68E-09	1.72E-02	Akt,ERK,ERK1/2,GSK3B,Jnk,STAT3,TP63,TP73,WWTR1	B2M,BTRC,CDK5R1,CYP17A1,CYP7A1,DTYMK,ESR1,F3,FAS,G6PD,HBEGF,HK2,HMOX1,IGF1R,ITGB3,MSH2,NOTCH1,ODC1,PDK4,PGK1,PTPRE,RAC1,RARA,RRM1,SFN,THBS2,TJP1,WEE1,XIAP,ZEB2	9	30
SAA	group	3.68E-09	2.10E-02	Akt,ERK1/2,NFkB (complex),P38 MAPK,SAA	B2M,CTCF,CYP17A1,CYP7A1,DGKA,ESR1,F3,FAS,HBEGF,HK2,IFNG,ITGB3,KYNU,MMP1,MMP2,MUC1,NOTCH1,PDK4,PTPRE,RARA,SENP2,TJP1,XIAP	5	23
SP4	transcription regulator	3.68E-09	1.09E-02	Akt,ERK,ERK1/2,Mapk,NFkB (complex),PRKAA,RAS,SP4	AR,B2M,CDK5R1,CTCF,CYP17A1,CYP7A1,ESR1,F3,FN1,HK2,HMOX1,IFNG,IGF1R,ITGB3,KYNU,MSH2,MUC1,NR1I3,ODC1,PTPRE,SENP2,TJP1,XIAP	8	23
CXCR2	G-protein coupled receptor	3.73E-09	4.70E-03	CXCR2,ERK1/2,Jnk	B2M,BTRC,CYP17A1,CYP7A1,ESR1,FN1,HBEGF,HK2,IFNG,IGF1R,MMP1,MMP2,MSH2,PTPRE,RARA,XIAP	2	16

KLHL42	other	3.81E-09	6.80E-03	ERK,ERN1,KLHL42,NFkB (complex),PPP2R5E,SMAD2	ANG,CDK5R1,CTCF,ESR1,FAS,FN1,HBEGF,HMOX1,ITGB3,KYNU,MMP2,MSH2,MUC1,NOTCH1,ODC1,PDK4,RAC1,SENP2,XIAP	5	19
PSMD14	peptidase	3.82E-09	2.58E-02	26s Proteasome,EGFR,ERK1/2,HIF1A,Jnk,JUN,MMP2,NR3C1,PSMD14,STAT3	AR,B2M,BTRC,CSF2RB,CYP17A1,CYP7A1,ERBB4,F3,FN1,HBEGF,HK2,HMOX1,HNRNPA2B1,IFNG,ITGB3,MMP1,MMP2,MSH2,MUC1,NAE1,NFATC1,NOTCH1,NR1I3,PGK1,PPP2R1A,PSAP,RARA,TJP1,XIAP,ZEB2	9	30
DDR2	kinase	3.87E-09	1.15E-02	DDR2,ERK1/2,JAK2,MAPT,MMP14,MMP2,SRC,STAT1,STAT3	B2M,C1R,CSF2RB,CYP17A1,CYP7A1,ESR1,FAS,FN1,HBEGF,HK2,HMOX1,IGF1R,MMP1,MMP2,MUC1,NOTCH1,PGK1,PPP2R1A,PTPRE,RARA,TJP1,TRIM21,WARS1	7	23
ADCYAP1	other	3.93E-09	1.24E-02	ADCYAP1,ERK,ERK1/2,P38 MAPK	B2M,CDK5R1,CYP17A1,CYP7A1,DGKA,ESR1,F3,HK2,HMOX1,IFNG,IGF1R,ITGB3,MMP1,MMP2,MSH2,MUC1,ODC1,PDK4,PTPRE,RARA	3	20
Focal adhesion kinase	group	4.06E-09	3.10E-03	ERK1/2,Focal adhesion kinase,NANOG,P38 MAPK	B2M,CYP17A1,CYP7A1,DGKA,ESR1,F3,FAS,FN1,HBEGF,HK2,HMOX1,IFNGR1,IGF1R,ITGB3,MSH2,PTPRE,RARA,S100A6,XIAP	3	19
TP73-AS1	other	4.07E-09	4.81E-02	Akt,CCAR2,ELAVL1,ERK,ERK1/2,FOXO1,HSF1,IRF3,MMP2,MTOR,NFkB (complex),Notch,P38 MAPK,PI3K (family),RELA,RHOA,SIRT1,SREBF1,SREBF2,STAT1,STAT3,TFEB,TP53,TP73-AS1	AR,B2M,C1R,CAMK4,CDK5R1,CERT1,CHKA,CSF2RB,CTCF,CYP17A1,CYP7A1,DGKA,EP300,ESR1,F3,FABP3,FABP7,GART,GTF2I,HBEGF,HMOX1,HSP90AB1,IFNG,ITGB3,KIF11,KYNU,MMP2,MUC1,NDC80,NMRK1,PANK1,PDHA1,PDK4,PGK1,PPP2R1A,PRKCB,PSAP,SENP2,SFN,STK4,TJP1,TRIM21,TTR,VPS26A,WARS1,WEE1,XIAP,YWHAH,ZEB2	23	49
PIK3CA	kinase	4.16E-09	1.30E-02	Akt,AKT1,ERK1/2,FOXO1,GSK3B,MTOR,PI3K (family),PIK3CA	B2M,CAMK4,CHKA,CYP17A1,CYP7A1,ESR1,F3,FN1,HBEGF,HK2,HMOX1,IFNG,IGF1R,KIF11,MMP1,MUC1,NOTCH1,PDK4,PTPRE,RAC1,TJP1,XIAP	7	22
CCR7	G-protein coupled receptor	4.20E-09	5.80E-03	Akt,CCR7,ERK1/2,LCK,MAP2K1/2	B2M,CYP17A1,CYP7A1,ESR1,F3,FN1,HBEGF,HK2,IFNG,MMP1,MMP2,MUC1,NOTCH1,PDK4,PPARG,PTPRE,RRM1,TJP1	4	18
P2RY1	G-protein coupled receptor	4.22E-09	2.70E-02	DGKA,ERK,ERK1/2,NFkB (complex),P2RY1,P38 MAPK,PLC,RAC1,RAP1B,RELA	B2M,CDK5R1,CERT1,CYP17A1,CYP7A1,DGKA,ESR1,F3,HK2,IFNG,IGF1R,ITGB3,KYNU,MMP1,MMP2,MUC1,NOTCH1,ODC1,PDK4,PPARG,PTPRE,RARA,SENP2,STK4,WEE1,XIAP	7	26
GALNT2	enzyme	4.34E-09	8.10E-03	Akt,EGFR,ERK1/2,GALNT2,SRC	AR,B2M,CYP17A1,CYP7A1,ESR1,FN1,HBEGF,HK2,HNRNPA2B1,IFNG,MMP1,MMP2,MUC1,NOTCH1,PDK4,PTPRE,TJP1,XIAP,ZEB2	4	19
CGN	other	4.44E-09	1.22E-02	ARHGEF2,CGN,ERK1/2,NFkB (complex),RHOA,Tgf beta	B2M,CTCF,CYP17A1,CYP7A1,ESR1,FAS,FN1,HBEGF,HK2,HMOX1,IFNG,IGF1R,KYNU,MMP2,MSH2,NOTCH1,PDHA1,PTPRE,SENP2,XIAP	4	20
DUSP6	phosphatase	4.53E-09	1.35E-02	DUSP6,ERK,ERK1/2,PGR	B2M,CDK5R1,CYP17A1,CYP7A1,ESR1,F3,FAS,FN1,HK2,IFNG,IGF1R,ITGB3,MATK,MMP1,MMP2,MUC1,ODC1,PDK4,PNMT,PTPRE,SERPINA5	3	21

ELANE	peptidase	4.63E-09	1.45E-02	ELANE,ERK1/2,NFkB (complex),RELA	B2M,CERT1,CYP17A1,CYP7A1,ESR1,FAS,FN1,HBEGF,HK2,HMOX1,IFNG,IGF1R,KYNU,MMP1,MMP2,MSH2,NOTCH1,PPARG,PTPRE,SENP2,STK4,XIAP	4	22
ZNF148	transcription regulator	4.63E-09	2.18E-02	CASP8,ERK1/2,MAPK8,NFkB (complex),P38 MAPK,ZNF148	B2M,CTCF,CYP17A1,CYP7A1,DGKA,ESR1,F3,FAS,FN1,HBEGF,HK2,IFNG,IGF1R,ITGB3,KYNU,MMP1,MMP2,NOTCH1,PTPRE,RARA,SENP2,XIAP	5	22
VEGFB	growth factor	4.66E-09	8.10E-03	ERK1/2,FLT1,Jnk,SRC (family),VEGFB	B2M,BTRC,CYP17A1,CYP7A1,ESR1,HBEGF,HK2,IFNG,IGF1R,ITGB3,MMP1,MMP2,MSH2,NOTCH1,PTPRE,RARA,XIAP	4	17
HSP90AA1	enzyme	4.66E-09	9.00E-04	c-Src,EGFR,HSP90AA1,LATS1	AR,CYP7A1,ESR1,F3,FN1,HK2,HMOX1,HNRNPA2B1,IFNG,MMP1,MUC1,PTPRE,XIAP,ZEB2	4	14
CD81	other	4.66E-09	2.30E-03	CD81,ERK,MMP2,RAC1	CDK5R1,CSF2RB,ESR1,FAS,HBEGF,IFNG,ITGB3,MMP1,MMP2,MUC1,ODC1,PDK4,PPP2R1A,TJP1	4	14
PELI2	enzyme	4.80E-09	4.50E-03	ERK,Jnk,PELI2	BTRC,CDK5R1,ESR1,FAS,HBEGF,IFNG,ITGB3,MMP1,MMP2,MSH2,MUC1,ODC1,PDK4,RARA,XIAP	2	15
TGFA	growth factor	4.80E-09	2.50E-03	EGFR,MET,RPS6KB1,SRC,TGFA	AR,ESR1,F3,FN1,HK2,HMOX1,HNRNPA2B1,IFNG,IGF1R,MMP1,MMP2,MUC1,PTPRE,XIAP,ZEB2	4	15
SND1-BRAF	fusion gene/product	4.83E-09	8.80E-03	ERK,ERK1/2,MAP2K1/2,SND1-BRAF	B2M,CDK5R1,CYP17A1,CYP7A1,ESR1,FAS,FN1,HK2,IFNG,IGF1R,ITGB3,MMP1,MMP2,MUC1,ODC1,PDK4,PTPRE,RRM1	3	18
DBH-AS1	other	4.83E-09	1.04E-02	DBH-AS1,ERK,Jnk,P38 MAPK	BTRC,CDK5R1,DGKA,ESR1,F3,FN1,HBEGF,HMOX1,IFNG,ITGB3,MMP1,MMP2,MSH2,MUC1,ODC1,PDK4,RARA,XIAP	3	18
CSNK2B	kinase	4.88E-09	1.55E-02	CSNK2B,ERK1/2,NFkB (complex),RELA	B2M,CERT1,CYP17A1,CYP7A1,ESR1,FAS,FN1,HBEGF,HK2,HMOX1,IFNG,IGF1R,KYNU,MMP1,MMP2,MSH2,NOTCH1,PPARG,PTPRE,SENP2,STK4,XIAP	3	22
PTGER2	G-protein coupled receptor	4.88E-09	1.43E-02	AKT1,EGFR,ERK1/2,PTGER2,RELA	AR,B2M,CERT1,CTCF,CYP17A1,CYP7A1,ESR1,F3,FAS,FN1,HBEGF,HK2,HMOX1,HNRNPA2B1,IGF1R,MMP1,MMP2,PPARG,PTPRE,STK4,XIAP,ZEB2	4	22
MAP3K11	kinase	4.88E-09	2.07E-02	ERK,ERK1/2,Jnk,JUN,MAP3K11,P38 MAPK	B2M,BTRC,CDK5R1,CYP17A1,CYP7A1,DGKA,ESR1,F3,HK2,HMOX1,IFNG,IGF1R,ITGB3,MMP1,MMP2,MSH2,MUC1,ODC1,PDK4,PTPRE,RARA,XIAP	5	22
NTRK2	kinase	4.93E-09	1.34E-02	Akt,ERK,ERK1/2,NTRK2,RHOA	B2M,CDK5R1,CYP17A1,CYP7A1,ESR1,F3,FAS,FN1,HK2,IFNG,ITGB3,MMP1,MMP2,MUC1,NOTCH1,ODC1,PDHA1,PTPRE,TJP1	4	19
MAP3K19	kinase	5.41E-09	7.20E-03	ERK1/2,Jnk,MAP2K1/2,MAP3K19	B2M,BTRC,CYP17A1,CYP7A1,ESR1,FN1,HBEGF,HK2,IFNG,IGF1R,MMP1,MMP2,MSH2,PTPRE,RARA,RRM1,XIAP	3	17

NF1	other	5.61E-09	2.10E-02	ADCY,ERK1/2,GNAS,HIF1A,Jnk,JUN,MMP2,NF1	B2M,BTRC,CSF2RB,CYP17A1,CYP7A1,ERBB4,ESR1,FN1,HBEGF,HK2,IFNG,IGF1R,ITGB3,MMP1,MMP2,MSH2,MUC1,NAE1,NFATC1,PPP2R1A,PTPRE,RARA,TJP1,XIAP	6	24
PDGFRB	kinase	5.64E-09	1.19E-02	Akt,AKT1,ERK1/2,JAK1,JAK2,PDGFRB,RAS,SMAD2,TYK2	B2M,CYP17A1,CYP7A1,ESR1,F3,FN1,HBEGF,HK2,HMOX1,IFNG,MMP1,MMP2,MUC1,NOTCH1,PDK4,PTPRE,RAC1,RARA,TJP1,XIAP	9	20
**NRP1**	**transmembrane receptor**	**5.64E-09**	**1.41E-02**	**Akt,EGFR,ERK,KDR,MET,NRP1,SMAD2,SMAD3**	**AR,CDK5R1,ESR1,FAS,FN1,HBEGF,HK2,HMOX1,HNRNPA2B1,IGF1R,ITGB3,MMP1,MMP2,MUC1,NOTCH1,ODC1,PTPRE,RAC1,TJP1,ZEB2**	**7**	**20**
INHBA	growth factor	5.78E-09	1.01E-02	Akt,ERK1/2,GSK3B,INHBA,P38 MAPK,SMAD2,SMAD3	B2M,CYP17A1,CYP7A1,DGKA,ERBB4,ESR1,F3,FAS,HBEGF,HK2,HMOX1,IFNG,ITGB3,MSH2,MUC1,NOTCH1,PDK4,PTPRE,RAC1,RARA,TJP1,VAV3,XIAP	7	23
ANXA2	other	5.88E-09	2.96E-02	Akt,ANXA2,ERK1/2,Jnk,P38 MAPK,SRC,STAT3	ANG,B2M,BTRC,CYP17A1,CYP7A1,DGKA,ESR1,FAS,HBEGF,HK2,HMOX1,IFNG,ITGB3,MMP1,MMP2,MSH2,MUC1,NOTCH1,PDK4,PGK1,PTPRE,RARA,TJP1,XIAP	7	24
KIT	transmembrane receptor	5.93E-09	1.02E-02	AKT1,ERK,ERK1/2,KIT	B2M,CDK5R1,CYP17A1,CYP7A1,ESR1,FAS,FN1,HK2,HMOX1,IFNG,IGF1R,ITGB3,MMP1,MMP2,MUC1,ODC1,PDK4,PTPRE	3	18
IL21	cytokine	6.05E-09	4.35E-02	ERK1/2,IL21,Mapk,NFkB (complex),RELA,STAT1,STAT3	B2M,C1R,CERT1,CYP17A1,CYP7A1,ESR1,FAS,FN1,HBEGF,HK2,IFNG,IGF1R,KYNU,MMP1,MMP2,MSH2,MUC1,NOTCH1,NR1I3,PGK1,PPARG,PTPRE,SENP2,STK4,TLR4,TRIM21,WARS1,XIAP	7	28
PRR7	other	6.10E-09	6.60E-03	ERK1/2,Jnk,PRR7,ZAP70	B2M,BTRC,CYP17A1,CYP7A1,ESR1,FN1,HBEGF,HK2,IFNG,IGF1R,MMP1,MMP2,MSH2,PTPRE,RARA,XIAP	3	16
IL6R	transmembrane receptor	6.34E-09	1.42E-02	ERK1/2,IL6R,MMP2,STAT1,STAT3	B2M,C1R,CSF2RB,CYP17A1,CYP7A1,ESR1,FAS,FN1,HBEGF,HK2,HMOX1,IGF1R,MMP1,MMP2,MUC1,NOTCH1,PGK1,PPP2R1A,PTPRE,TJP1,TRIM21,WARS1	4	22
HTRA1	peptidase	6.60E-09	4.90E-03	Akt,EGFR,HTRA1,Mapk	AR,ESR1,HK2,HNRNPA2B1,IFNG,IGF1R,MMP1,MMP2,MUC1,NOTCH1,NR1I3,PDK4,PTPRE,TJP1,XIAP,ZEB2	4	16
SAA1	transporter	6.70E-09	2.84E-02	Akt,ERK1/2,Jnk,NFkB (complex),P38 MAPK,SAA1	B2M,BTRC,CTCF,CYP17A1,CYP7A1,DGKA,ESR1,FAS,HBEGF,HK2,IFNG,ITGB3,KYNU,MMP1,MMP2,MUC1,NOTCH1,PDK4,PPARG,PTPRE,RARA,SENP2,TJP1	6	23
PRKCB	kinase	6.75E-09	1.30E-02	ERK1/2,Jnk,MAPK8,NCF1,PRKCB,RAS	B2M,BTRC,CYP17A1,CYP7A1,ESR1,F3,FN1,HBEGF,HK2,IFNG,IGF1R,MMP1,MMP2,MSH2,PTPRE,RARA,XIAP	6	17

F2	peptidase	6.93E-09	1.83E-02	ERK1/2,F2,Hsp27,JAK2,MAPK1,MAPK8,NFkB (complex),P38 MAPK,PRKAA1,PRKCQ,PTK2B,RHOA,SRC (family),VSIR	AR,B2M,CD1A,CTCF,CYP17A1,CYP7A1,DGKA,EPHA3,ESR1,F3,FN1,HBB,HBEGF,HK2,HMOX1,IFNG,ITGB3,KYNU,MAGEA4,MMP1,MSH2,NOTCH1,PDHA1,PTPRE,RARA,SENP2,TJP1,TRIM21,XIAP	14	29
ITGA5	transmembrane receptor	7.07E-09	1.90E-02	ERK1/2,ITGA5,NFkB (complex),P38 MAPK	B2M,CTCF,CYP17A1,CYP7A1,DGKA,ESR1,F3,FAS,HBEGF,HK2,IFNG,IGF1R,ITGB3,KYNU,MMP1,MMP2,NOTCH1,PTPRE,RARA,SENP2,XIAP	3	21
TDGF1	growth factor	7.26E-09	6.60E-03	Akt,ERBB4,ERK1/2,GSK3B,TDGF1	B2M,CYP17A1,CYP7A1,ESR1,F3,FN1,HBEGF,HK2,MMP1,MMP2,MUC1,NOTCH1,PDK4,PGK1,PTPRE,RAC1,TJP1	4	17
myosin-light-chain kinase	group	7.26E-09	1.29E-02	ERK1/2,Jnk,MAP2K1,MAP2K1/2,myosin-light-chain kinase	B2M,BTRC,CYP17A1,CYP7A1,ESR1,F3,FN1,HBEGF,HK2,IFNG,IGF1R,MMP1,MMP2,MSH2,PTPRE,RARA,RRM1,XIAP	4	18
STK38	kinase	7.32E-09	2.76E-02	ERK1/2,MYC,P38 MAPK,RELA,STK38,YAP1	B2M,BCR,CERT1,CTCF,CYP17A1,CYP7A1,DGKA,DTYMK,ESR1,F3,FAP,FAS,HBEGF,HK2,HMOX1,IFNG,IGF1R,ITGB3,MMP1,MMP2,MSH2,ODC1,PPARG,PTPRE,RARA,RRM1,STK4	5	27
BCL2L1	other	7.43E-09	3.65E-02	BAX,BCL2L1,CASP1,CASP8,CASP9,caspase,EIF2AK2,ERK1/2,IRF3,MAPK8,NFkB (complex),P38 MAPK	B2M,CTCF,CYP17A1,CYP7A1,DGKA,ESR1,F3,FAS,FN1,HBEGF,HK2,IFNG,IGF1R,ITGB3,KYNU,MMP1,MMP2,NOTCH1,PTPRE,RARA,SENP2,STK4,XIAP,ZNF292	8	24
GHRL	growth factor	7.80E-09	7.00E-03	Akt,c-Src,ERK1/2,GHRL,MMP2	B2M,CSF2RB,CYP17A1,CYP7A1,ESR1,F3,FN1,HBEGF,HK2,HMOX1,MMP1,MMP2,MUC1,NOTCH1,PDK4,PPP2R1A,PTPRE	4	17
F2R	G-protein coupled receptor	8.22E-09	1.65E-02	ERK1/2,F2R,LATS1,NFkB (complex),P38 MAPK,PI3K (complex),RELA,RHOA,YAP1	B2M,CERT1,CTCF,CYP17A1,CYP7A1,DGKA,DTYMK,ESR1,F3,HBEGF,HK2,HMOX1,IGF1R,ITGB3,KYNU,MSH2,MUC1,PDHA1,PPARG,PTPRE,RARA,RRM1,SENP2,STK4,XIAP	8	25
TRIB2	kinase	8.25E-09	4.40E-02	BAX,CASP8,CASP9,CEBPA,ERK1/2,IRF3,MAPK8,MMP14,MMP2,NFkB (complex),P38 MAPK,SRC,STAT3,TRIB2	ALB,B2M,CSF2RB,CTCF,CYP17A1,CYP7A1,DGKA,ESR1,F3,FAS,HBEGF,HK2,HMOX1,IFNG,IGF1R,ITGB3,KYNU,MMP2,NOTCH1,PGK1,PPARG,PPP2R1A,PTPRE,RARA,SENP2,STK4,TJP1,XIAP	9	28
MCF2L	other	8.86E-09	1.29E-02	CDC42,ERK1/2,MCF2L,P38 MAPK,RHOA,Tgf beta	B2M,CYP17A1,CYP7A1,DGKA,ESR1,F3,FAS,HBEGF,HK2,HMOX1,IGF1R,ITGB3,MMP2,MSH2,PDHA1,PTPRE,RARA,XIAP	4	18
SELE	transmembrane receptor	9.00E-09	8.70E-03	ERK,Hsp27,P38 MAPK,SELE	CD1A,CDK5R1,DGKA,ESR1,F3,FN1,HBEGF,HMOX1,IFNG,ITGB3,MMP1,MMP2,MSH2,MUC1,ODC1,PDK4,RARA	3	17
PLCB3	enzyme	9.23E-09	2.80E-03	ERK,KRAS,PLCB3	CDK5R1,ESR1,FAS,HBEGF,IFNG,IGF1R,ITGB3,MMP1,MMP2,MUC1,ODC1,PC,PDK4,YY1	2	14

PIK3C2A	kinase	9.78E-09	4.47E-02	ERK1/2,ERN1,FOXO1,NFkB (complex),PIK3C2A,PRKAA,SMAD2,SMAD3	ANG,AR,B2M,CAMK4,CTCF,CYP17A1,CYP7A1,ESR1,FAS,FN1,HBEGF,HK2,IFNG,IGF1R,KIF11,KYNU,MMP1,MMP2,MSH2,NOTCH1,PTPRE,RAC1,SENP2,XIAP	7	24
SCUBE3	other	9.78E-09	4.47E-02	ERK1/2,ERN1,FOXO1,NFkB (complex),PRKAA,SCUBE3,SMAD2,SMAD3	ANG,AR,B2M,CAMK4,CTCF,CYP17A1,CYP7A1,ESR1,FAS,FN1,HBEGF,HK2,IFNG,IGF1R,KIF11,KYNU,MMP1,MMP2,MSH2,NOTCH1,PTPRE,RAC1,SENP2,XIAP	8	24
MIR100HG	other	9.78E-09	4.47E-02	ERK1/2,ERN1,FOXO1,MIR100HG,NFkB (complex),PRKAA,SMAD2,SMAD3	ANG,AR,B2M,CAMK4,CTCF,CYP17A1,CYP7A1,ESR1,FAS,FN1,HBEGF,HK2,IFNG,IGF1R,KIF11,KYNU,MMP1,MMP2,MSH2,NOTCH1,PTPRE,RAC1,SENP2,XIAP	8	24
ZAP70	kinase	1.01E-08	1.17E-02	ERK1/2,P38 MAPK,ZAP70	B2M,CYP17A1,CYP7A1,DGKA,ESR1,F3,FAS,HBEGF,HK2,HMOX1,IFNG,IGF1R,ITGB3,MMP1,MMP2,MSH2,PTPRE,RARA	3	18
SULF2	enzyme	1.01E-08	8.20E-03	Akt,ERK1/2,SMAD2,SMAD3,SULF2	B2M,CYP17A1,CYP7A1,ESR1,F3,FN1,HBEGF,HK2,IFNG,IGF1R,MMP1,MUC1,NOTCH1,PDK4,PTPRE,RAC1,TJP1,XIAP	4	18
DEFB4A/DEFB4B	other	1.04E-08	9.30E-03	DEFB4A/DEFB4B,ERK1/2,P38 MAPK	B2M,CYP17A1,CYP7A1,DGKA,ESR1,F3,FAS,HBEGF,HK2,HMOX1,IGF1R,ITGB3,MMP1,MMP2,MSH2,PTPRE,RARA	2	17
STK3	kinase	1.05E-08	5.60E-03	AKT1,ERK1/2,Jnk,LATS1,STK3	B2M,BTRC,CYP17A1,CYP7A1,ESR1,FN1,HBEGF,HK2,HMOX1,IFNG,IGF1R,MSH2,MUC1,PTPRE,RARA,XIAP	4	16
HPSE	enzyme	1.06E-08	2.18E-02	Akt,EGFR,ERK,ERK1/2,HPSE	AR,B2M,CDK5R1,CYP17A1,CYP7A1,ESR1,FAS,FN1,HK2,HNRNPA2B1,ITGB3,MMP1,MMP2,MUC1,NOTCH1,ODC1,PTPRE,TJP1,XIAP,ZEB2	4	20
CCAT1	other	1.06E-08	2.41E-02	Akt,CCAT1,ERK1/2,MAP2K1/2,P38 MAPK	B2M,CYP17A1,CYP7A1,DGKA,ESR1,FAS,HBEGF,HK2,HMOX1,ITGB3,MMP1,MMP2,MSH2,MUC1,NOTCH1,PDK4,PTPRE,RARA,RRM1,TJP1	4	20
TMSB4	group	1.08E-08	1.98E-02	ERK,Jnk,P38 MAPK,TMSB4	BTRC,CDK5R1,DGKA,ESR1,F3,FN1,HBEGF,HMOX1,IFNG,ITGB3,MMP1,MMP2,MSH2,MUC1,ODC1,PDK4,RARA,XIAP	3	18
TSC22D1	transcription regulator	1.12E-08	4.77E-02	ERK1/2,ERN1,FOXO1,NFkB (complex),PRKAA,SMAD2,SMAD3,TSC22D1	ANG,AR,B2M,CAMK4,CTCF,CYP17A1,CYP7A1,ESR1,FAS,FN1,HBEGF,HK2,IFNG,IGF1R,KIF11,KYNU,MMP1,MMP2,MSH2,NOTCH1,PTPRE,RAC1,SENP2,XIAP	7	24
ITCH	enzyme	1.13E-08	1.07E-02	CFLAR,ERK1/2,ITCH,Jnk,NFkB (complex),P38 MAPK	B2M,BTRC,CTCF,CYP17A1,CYP7A1,DGKA,ESR1,F3,HBEGF,HK2,HMOX1,IGF1R,ITGB3,KYNU,MSH2,NOTCH1,PTPRE,RARA,SENP2,XIAP	5	20
DEFB103A/DEFB103B	other	1.14E-08	4.80E-03	DEFB103A/DEFB103B,ERK1/2,P38 MAPK	B2M,CYP17A1,CYP7A1,DGKA,ESR1,F3,FAS,FN1,HBEGF,HK2,HMOX1,IGF1R,ITGB3,MSH2,PTPRE,RARA	2	16

IL33	cytokine	1.14E-08	4.35E-02	ERK,ERK1/2,IL33,Jnk,NFkB (complex),P38 MAPK	B2M,BTRC,CDK5R1,CTCF,CYP17A1,CYP7A1,DGKA,ESR1,F3,HK2,IFNG,IGF1R,ITGB3,KYNU,MMP1,MMP2,MUC1,NOTCH1,ODC1,PDK4,PTPRE,RARA,SENP2	6	23
CHCHD2	other	1.17E-08	4.92E-02	CHCHD2,ERK1/2,ERN1,FOXO1,NFkB (complex),PRKAA,SMAD2,SMAD3	ANG,AR,B2M,CAMK4,CTCF,CYP17A1,CYP7A1,ESR1,FAS,FN1,HBEGF,HK2,IFNG,IGF1R,KIF11,KYNU,MMP1,MMP2,MSH2,NOTCH1,PTPRE,RAC1,SENP2,XIAP	7	24
BMP6	growth factor	1.23E-08	5.70E-03	BMP6,ERK1/2,P38 MAPK	B2M,CYP17A1,CYP7A1,DGKA,ESR1,F3,FAS,FN1,HBEGF,HK2,HMOX1,IGF1R,ITGB3,MSH2,PTPRE,RARA	2	16
PTAFR	G-protein coupled receptor	1.25E-08	2.23E-02	Akt,ERK1/2,P38 MAPK,PTAFR	B2M,CYP17A1,CYP7A1,DGKA,ESR1,FAS,HBEGF,HK2,HMOX1,ITGB3,MMP1,MMP2,MSH2,MUC1,NOTCH1,PDK4,PTPRE,RARA,TJP1	4	19
IL32	cytokine	1.28E-08	3.67E-02	CEBPA,EP300,ERK1/2,IL32,NFkB (complex),P38 MAPK	ALB,AR,B2M,CTCF,CYP17A1,CYP7A1,DGKA,ESR1,F3,FAS,HBEGF,HK2,IFNG,IGF1R,ITGB3,KYNU,MMP1,MMP2,NOTCH1,PPARG,PTPRE,RARA,SENP2,XIAP	5	24
CRH	cytokine	1.28E-08	9.70E-03	CRH,ERK1/2,P38 MAPK	B2M,CYP17A1,CYP7A1,DGKA,ESR1,F3,FAS,HBEGF,HK2,HMOX1,IGF1R,ITGB3,MMP1,MMP2,MSH2,PTPRE,RARA	2	17
FLT3	kinase	1.29E-08	2.05E-02	ERK1/2,FLT3,JAK2,MAPT,MMP14,MMP2,SRC,STAT1,STAT3	B2M,C1R,CSF2RB,CYP17A1,CYP7A1,ESR1,FAS,FN1,HBEGF,HK2,HMOX1,IGF1R,MMP2,MUC1,NOTCH1,PGK1,PPP2R1A,PTPRE,RARA,TJP1,TRIM21,WARS1	6	22
VHL	transcription regulator	1.32E-08	1.42E-02	ERK1/2,FGFR1,NFkB (complex),SPRY2,VHL	B2M,CTCF,CYP17A1,CYP7A1,ESR1,FAS,FN1,HBEGF,HMOX1,IFNG,IGF1R,KYNU,MSH2,NOTCH1,PGK1,PTPRE,SENP2,SFN,XIAP	4	19
WNT5A	cytokine	1.33E-08	1.05E-02	Akt,Jnk,MTOR,P38 MAPK,WNT5A	AR,BTRC,CHKA,DGKA,F3,FAS,FN1,HMOX1,IGF1R,ITGB3,MMP1,MSH2,MUC1,NOTCH1,PDK4,RARA,SEMA4D,TJP1,TLR4,XIAP	5	20
PEA15	transporter	1.34E-08	3.10E-03	ERK1/2,Jnk,PEA15	B2M,BTRC,CYP17A1,CYP7A1,ESR1,FN1,HBEGF,HK2,IFNG,IGF1R,MSH2,PTPRE,RARA,XIAP	2	14
CGB3 (includes others)	other	1.47E-08	3.50E-03	CGB3 (includes others),ERK1/2,MMP2	B2M,CSF2RB,CYP17A1,CYP7A1,ESR1,FN1,HBEGF,HK2,IGF1R,MMP1,MMP2,PPP2R1A,PTPRE,TJP1	3	14
FGF18	growth factor	1.47E-08	1.08E-02	ERK1/2,FGF18,P38 MAPK	B2M,CYP17A1,CYP7A1,DGKA,ESR1,F3,FAS,HBEGF,HK2,HMOX1,IGF1R,ITGB3,MMP1,MMP2,MSH2,PTPRE,RARA	2	17
FGFR4	kinase	1.47E-08	1.13E-02	Akt,ERK1/2,FGFR4,MTOR,SRC	AR,B2M,CHKA,CYP17A1,CYP7A1,ESR1,F3,FN1,HBEGF,HK2,IFNG,MMP1,MMP2,NOTCH1,PDK4,PTPRE,TJP1	4	17

RAS	group	1.49E-08	1.84E-02	Akt,ERK,ERK1/2,Mapk,NFkB (complex),PRKAA,RAS	AR,B2M,CDK5R1,CTCF,CYP17A1,CYP7A1,ESR1,F3,FN1,HK2,HMOX1,IFNG,ITGB3,KYNU,MSH2,MUC1,NR1I3,ODC1,PTPRE,SENP2,TJP1,XIAP	7	22
IL17F	cytokine	1.49E-08	2.02E-02	ERK,ERK1/2,IL17F,RELA	B2M,CDK5R1,CERT1,CTCF,CYP17A1,CYP7A1,ESR1,FN1,HK2,IFNG,IGF1R,ITGB3,MMP1,MMP2,MUC1,ODC1,PDK4,PPARG,PTPRE,STK4	3	20
mir-155	microRNA	1.57E-08	1.92E-02	Akt,ERK,Gsk3,mir-155,RELA	CDK5R1,CERT1,CTCF,ESR1,F3,HBEGF,HK2,IFNG,IGF1R,ITGB3,MMP1,MMP2,MUC1,NOTCH1,ODC1,PPARG,STK4,TJP1,WEE1,XIAP	5	20
DNM2	enzyme	1.58E-08	1.06E-02	Akt,DNM2,EGFR,ERK1/2,MMP2	AR,B2M,CSF2RB,CYP17A1,CYP7A1,F3,FN1,HBEGF,HNRNPA2B1,IFNG,MMP2,MUC1,NOTCH1,PDK4,PPP2R1A,TJP1,XIAP,ZEB2	4	18
SH2D1A	other	1.61E-08	5.20E-03	DGKA,ERK1/2,LCK,SH2D1A	B2M,CYP17A1,CYP7A1,ESR1,FN1,HBEGF,HK2,IFNG,IGF1R,MMP1,MMP2,PPARG,PTPRE,WEE1	4	14
PDGFB	growth factor	1.73E-08	6.80E-03	Akt,ERK,PDGFB,PDGFRB	CDK5R1,ESR1,F3,FAS,FN1,HBEGF,IFNG,IGF1R,ITGB3,MMP1,MMP2,MUC1,NOTCH1,ODC1,TJP1	3	15
TNFSF15	cytokine	1.74E-08	3.54E-02	ERK1/2,Jnk,NFkB (complex),P38 MAPK,TNFSF15	B2M,BTRC,CTCF,CYP17A1,CYP7A1,DGKA,ESR1,F3,FAS,HBEGF,HK2,IFNG,IGF1R,ITGB3,KYNU,MMP1,MMP2,NOTCH1,PTPRE,RARA,SENP2	4	21
CASP8	peptidase	1.74E-08	3.09E-02	CASP8,ERK1/2,MAPK8,NFkB (complex),P38 MAPK	B2M,CTCF,CYP17A1,CYP7A1,DGKA,ESR1,F3,FAS,HBEGF,HK2,IFNG,IGF1R,ITGB3,KYNU,MMP1,MMP2,NOTCH1,PTPRE,RARA,SENP2,XIAP	4	21
BTC	growth factor	1.78E-08	8.70E-03	Akt,BTC,ERBB4,ERK1/2	B2M,CYP17A1,CYP7A1,ESR1,F3,FN1,HBEGF,HK2,MMP1,MMP2,MUC1,NOTCH1,PDK4,PGK1,PTPRE,TJP1	3	16
NPY	other	1.78E-08	1.21E-02	ERK,NPY,P38 MAPK	CDK5R1,DGKA,ESR1,F3,FN1,HBEGF,HMOX1,IFNG,ITGB3,MMP1,MMP2,MSH2,MUC1,ODC1,PDK4,RARA	2	16
LAMA3	other	1.78E-08	1.21E-02	ERK,LAMA3,P38 MAPK	CDK5R1,DGKA,ESR1,F3,FN1,HBEGF,HMOX1,IFNG,ITGB3,MMP1,MMP2,MSH2,MUC1,ODC1,PDK4,RARA	3	16
TRAF7	enzyme	1.83E-08	1.82E-02	CHUK,ERK1/2,Jnk,MAP2K1/2,NFkB (complex),P38 MAPK,TRAF7	B2M,BTRC,CTCF,CYP17A1,CYP7A1,ESR1,F3,FAS,FN1,HBEGF,HK2,IFNG,IGF1R,ITGB3,KYNU,NOTCH1,PTPRE,RAC1,RARA,RRM1,SENP2	7	21
IL36A	cytokine	1.83E-08	3.66E-02	ERK1/2,IL36A,Jnk,NFkB (complex),P38 MAPK	B2M,BTRC,CTCF,CYP17A1,CYP7A1,DGKA,ESR1,F3,FAS,HBEGF,HK2,IFNG,IGF1R,ITGB3,KYNU,MMP1,MMP2,NOTCH1,PTPRE,RARA,SENP2	4	21
DGKD	kinase	1.87E-08	6.20E-03	Akt,DGKD,EGFR	AR,ESR1,HK2,HNRNPA2B1,IFNG,IGF1R,MMP1,MMP2,MUC1,NOTCH1,PDK4,PTPRE,TJP1,XIAP,ZEB2	2	15
MAP4K1	kinase	1.88E-08	6.50E-03	ERK,Jnk,LCP2,MAP4K1,Mapk,NFkB (complex),PIK3R1,PLCG1	AR,BTRC,CDK5R1,CTCF,ESR1,FAS,HBEGF,HMOX1,ITGB3,KYNU,MSH2,MUC1,NOTCH1,NR1I3,ODC1,PDK4,RARA,SENP2,XIAP	5	19
PGK1	kinase	1.92E-08	1.08E-02	Akt,ERK,MTOR,PGK1	AR,CDK5R1,CHKA,ESR1,F3,FAS,HBEGF,IFNG,IGF1R,ITGB3,MMP1,MMP2,MUC1,NOTCH1,ODC1,TJP1	3	16

PI3K 纬	group	1.92E-08	1.11E-02	Akt,ERK1/2,PI3K 纬	B2M,CYP17A1,CYP7A1,ESR1,F3,FN1,HBEGF,HK2,IFNG,MMP1,MMP2,MUC1,NOTCH1,PDK4,PTPRE,TJP1	3	16
KLF5	transcription regulator	1.92E-08	5.80E-03	DUSP1,ERK,KLF5,NOTCH1	CDK5R1,CYP17A1,ESR1,FAS,HBEGF,HMOX1,IFNG,IGF1R,ITGB3,MMP2,MUC1,NOTCH1,ODC1,PDK4,PPARG,RARA	4	16
PIK3CG	kinase	2.03E-08	2.06E-02	Akt,ERK,ERK1/2,PIK3CG	B2M,CDK5R1,CYP17A1,CYP7A1,ESR1,F3,FAS,FN1,HK2,IFNG,ITGB3,MMP1,MMP2,MUC1,NOTCH1,ODC1,PTPRE,TJP1	4	18
EGR1	transcription regulator	2.10E-08	3.80E-03	EGR1,ERK	CDK5R1,ESR1,F3,FAS,HBEGF,IFNG,ITGB3,MMP1,MMP2,MUC1,ODC1,PDK4,PPARG	2	13
Hsp90	group	2.12E-08	4.78E-02	ERBB2,ERK,HSF1,Hsp90,Jnk,P38 MAPK,STAT1	AR,BMP1,BTRC,C1R,CDK5R1,DGKA,ERBB4,ESR1,GART,GRK6,HBEGF,HMOX1,HSP90AB1,IFNG,ITGB3,MMP1,MMP2,MSH2,MUC1,ODC1,PDK4,PGK1,PPARG,PTPRF,RARA,RRM1,SMTN,TRIM21,TTR,WARS1,XIAP	7	31
RAP1B	enzyme	2.20E-08	1.42E-02	ERK1/2,P38 MAPK,RAC1,RAP1B	B2M,CYP17A1,CYP7A1,DGKA,ESR1,F3,FAS,HBEGF,HK2,HMOX1,IGF1R,ITGB3,MMP1,MMP2,MSH2,PTPRE,RARA	3	17
KLB	enzyme	2.24E-08	3.59E-02	Akt,CEBPA,ERK,ERK1/2,FOXO1,FRS2,GSK3B,Jnk,KLB,MAPK8,MED1,MMP2,MYC,P38 MAPK,SREBF2,TFEB,YBX1	ALB,B2M,BCR,BTRC,CAMK4,CDK5R1,CSF2RB,CYP17A1,CYP7A1,DGKA,DTYMK,EPHA3,ESR1,F3,FABP7,FAP,FN1,GTF2I,HBB,HBEGF,HK2,IGF1R,KIF11,MMP1,MUC1,NOTCH1,PDK4,PPP2R1A,PSAP,PTPRE,RAC1,RARA,TJP1,VPS26A	15	34
NRAS	enzyme	2.29E-08	2.38E-02	Akt,ERK,ERK1/2,NRAS	B2M,CDK5R1,CYP17A1,CYP7A1,ESR1,F3,FAS,FN1,HK2,IFNG,ITGB3,MMP1,MMP2,MUC1,NOTCH1,ODC1,PTPRE,TJP1	3	18
TIMP3	other	2.30E-08	4.14E-02	Akt,EGFR,ERK1/2,MET,NFkB (complex),RAF1,TIMP3	AR,B2M,CTCF,CYP17A1,CYP7A1,ESR1,FAS,FN1,HBEGF,HK2,HMOX1,HNRNPA2B1,KYNU,MMP1,MSH2,MUC1,NOTCH1,PDK4,PTPRE,SENP2,TJP1,ZEB2	7	22
FLCN	other	2.30E-08	4.53E-02	Akt,ERK1/2,FLCN,FOXO1,Gsk3,HSF1,MTOR,MTORC1,NR3C1,PRKAA,RHOA,RPS6KB1,SREBF1,SREBF2,TP53,YAP1	AR,B2M,CAMK4,CHKA,CYP17A1,CYP7A1,DGKA,DTYMK,ESR1,F3,FABP3,FABP7,FAS,GART,GTF2I,HBEGF,HSP90AB1,IFNG,KIF11,MMP1,MMP2,MUC1,NDC80,NFATC1,NR1I3,PANK1,PDHA1,PDK4,PGK1,PPARG,PTPRE,RRM1,SFN,TJP1,TTR,XIAP,YWHAH	14	37
AGER	transmembrane receptor	2.35E-08	2.94E-02	AGER,EGFR,ERK,ERK1/2,Mapk,P38 MAPK	AR,B2M,CDK5R1,CYP17A1,CYP7A1,DGKA,F3,HMOX1,HNRNPA2B1,IFNG,IGF1R,ITGB3,MMP2,MSH2,MUC1,NR1I3,ODC1,PDK4,RARA,XIAP,ZEB2	6	21
TRAF4	other	2.37E-08	6.20E-03	Akt,EGFR,TRAF4	AR,ESR1,HK2,HNRNPA2B1,IFNG,IGF1R,MMP1,MMP2,MUC1,NOTCH1,PDK4,PTPRE,TJP1,XIAP,ZEB2	2	15
LIMS1	other	2.38E-08	1.17E-02	AKT1,ERK1/2,LIMS1,MTOR	AR,B2M,CHKA,CYP17A1,CYP7A1,ESR1,FN1,HBEGF,HK2,HMOX1,IFNG,IGF1R,MMP1,MMP2,MUC1,PTPRE	3	16

NOD1	other	2.38E-08	8.10E-03	ERK1/2,Jnk,NOD1,P38 MAPK	B2M,BTRC,CYP17A1,CYP7A1,DGKA,ESR1,F3,FAS,HBEGF,HK2,HMOX1,IFNG,IGF1R,ITGB3,PTPRE,XIAP	3	16
S100B	other	2.43E-08	2.02E-02	Akt,GSK3B,NFkB (complex),RHOA,S100B	CTCF,F3,FAS,HMOX1,IFNG,IGF1R,KYNU,MMP1,MMP2,MSH2,MUC1,NOTCH1,PDHA1,PDK4,RAC1,SENP2,TJP1,XIAP	4	18
ADGRE5	G-protein coupled receptor	2.45E-08	3.80E-03	ADGRE5,ERK,Ras homolog	CDK5R1,ESR1,FAS,HBEGF,IFNG,ITGB3,MMP1,MMP2,MUC1,ODC1,PDK4,TJP1	2	12
PEBP1	other	2.50E-08	1.00E-02	ADCY,ERK,ERK1/2,PEBP1,RELA	B2M,CDK5R1,CERT1,CTCF,CYP17A1,CYP7A1,FAS,FN1,HBEGF,HK2,IGF1R,ITGB3,MUC1,NFATC1,ODC1,PDK4,PPARG,PTPRE,STK4	4	19
C3	peptidase	2.50E-08	2.42E-02	Akt,C3,C5AR2,ERK1/2,IRF3,MAPK8,PARP1	B2M,CYP17A1,CYP7A1,ESR1,F3,FAS,FN1,HBEGF,HK2,IFNG,MMP1,MMP2,MUC1,NOTCH1,PDK4,PTPRE,RARA,STK4,TJP1	6	19
P2RY2	G-protein coupled receptor	2.50E-08	4.91E-02	Akt,CEBPA,ERK1/2,FOXO1,GSK3B,Jnk,MAPK8,MED1,MMP2,MYC,P2RY2,Rac,RAC1,RHOA,SREBF2,STAT1,YBX1	ALB,B2M,BCR,BTRC,C1R,CAMK4,CSF2RB,CYP17A1,CYP7A1,DTYMK,EPHA3,ESR1,F3,FABP7,FAP,FN1,GTF2I,HBB,HBEGF,HK2,IGF1R,KIF11,MMP1,MUC1,NOTCH1,ODC1,PDHA1,PDK4,PPP2R1A,PTPRE,RAC1,RARA,TJP1,TRIM21,WARS1	15	35
TSLP	cytokine	2.65E-08	4.84E-02	ERK1/2,Jnk,P38 MAPK,STAT3,STAT5a/b,TSLP	B2M,BTRC,CYP17A1,CYP7A1,DGKA,ESR1,F3,FAS,HBEGF,HK2,HMOX1,IFNG,IGF1R,ITGB3,MMP1,MMP2,MSH2,NOTCH1,PGK1,PTPRE,RARA,XIAP	5	22
Pdgfr	group	2.74E-08	1.27E-02	Akt,ERK,GSK3B,Pdgfr,PDGFRB	CDK5R1,ESR1,F3,FAS,FN1,HBEGF,IFNG,IGF1R,ITGB3,MMP1,MMP2,MUC1,NOTCH1,ODC1,RAC1,TJP1	4	16
NMUR2	G-protein coupled receptor	2.74E-08	3.20E-03	ADCY,ERK,NMUR2	CDK5R1,ESR1,FAS,HBEGF,IFNG,ITGB3,MMP1,MMP2,MUC1,NFATC1,ODC1,PDK4	2	12
IL10	cytokine	2.92E-08	2.66E-02	ERK1/2,IL10,NFkB (complex),STAT1,STAT3,STAT5a/b	B2M,C1R,CD1A,CTCF,CYP17A1,CYP7A1,ESR1,FN1,HBEGF,HK2,HMOX1,IFNG,IGF1R,KYNU,MSH2,MUC1,PGK1,PTPRE,RARA,SENP2,TLR4,TRIM21,WARS1,XIAP	6	24
KLB	enzyme	2.94E-08	1.04E-02	Akt,ERK1/2,GSK3B,KLB	B2M,CYP17A1,CYP7A1,ESR1,F3,FN1,HBEGF,HK2,IGF1R,MMP1,MUC1,NOTCH1,PDK4,PTPRE,RAC1,TJP1	3	16
RASSF1	other	2.94E-08	1.94E-02	ERK1/2,Jnk,RAC1,RASSF1	B2M,BTRC,CYP17A1,CYP7A1,ESR1,FN1,HBEGF,HK2,IFNG,IGF1R,MMP1,MMP2,MSH2,PTPRE,RARA,XIAP	4	16
MADD	other	2.96E-08	8.30E-03	ERK,MADD,MAP2K1/2,RAS	CDK5R1,CYP7A1,ESR1,FAS,HBEGF,HK2,IFNG,ITGB3,MMP1,MMP2,MUC1,ODC1,PDK4,RRM1	3	14

NEU3	enzyme	2.99E-08	9.10E-03	Akt,EGFR,NEU3,Ras homolog	AR,ESR1,HK2,HNRNPA2B1,IFNG,IGF1R,MMP1,MMP2,MUC1,NOTCH1,PDK4,PTPRE,TJP1,XIAP,ZEB2	4	15
NMUR1	G-protein coupled receptor	3.05E-08	3.20E-03	ADCY,ERK,NMUR1	CDK5R1,ESR1,FAS,HBEGF,IFNG,ITGB3,MMP1,MMP2,MUC1,NFATC1,ODC1,PDK4	2	12
AKT3	kinase	3.05E-08	3.60E-03	AKT3,ERK	CDK5R1,ESR1,FAS,HBEGF,IFNG,ITGB3,MMP1,MMP2,MUC1,ODC1,PDK4,RAC1	2	12
Beta Arrestin	group	3.09E-08	5.80E-03	Beta Arrestin,ERK,SMAD2	CDK5R1,ESR1,FAS,FN1,HBEGF,IFNG,ITGB3,MMP1,MUC1,ODC1,PDK4,RAC1,XIAP	2	13
mir-122	microRNA	3.13E-08	1.00E-04	mir-122	CTCF,CYP7A1,G6PD,GALNT10,IGF1R,MAP3K3,MARK1,SEMA4D,VAV3,WARS1,YARS1	1	11
P2RY2	G-protein coupled receptor	3.15E-08	1.34E-02	Akt,ERK1/2,P2RY2,RAC1,RHOA	B2M,CYP17A1,CYP7A1,ESR1,F3,FN1,HBEGF,HK2,IGF1R,MMP1,MUC1,NOTCH1,PDHA1,PDK4,PTPRE,TJP1	4	16
mir-9	microRNA	3.23E-08	1.04E-02	Akt,ERK,mir-9,NTRK3	AR,CDK5R1,ESR1,F3,FAS,HBEGF,IFNG,IGF1R,ITGB3,MMP1,MMP2,MUC1,NOTCH1,ODC1,TJP1	3	15
GRN	growth factor	3.38E-08	1.39E-02	Akt,ERK1/2,GRN,Mapk	B2M,CYP17A1,CYP7A1,ESR1,F3,FN1,HBEGF,HK2,MMP1,MMP2,MUC1,NOTCH1,NR1I3,PDK4,PTPRE,TJP1	3	16
DAB2IP	other	3.48E-08	1.42E-02	Akt,c-Src,CTNNB1,DAB2IP,ERK,Jnk,MAP3K5,RPS6KB1	BTRC,CDK5R1,CYP7A1,ESR1,F3,FAS,FN1,HBEGF,HMOX1,IGF1R,ITGB3,MSH2,MUC1,NOTCH1,ODC1,RARA,TJP1,XIAP	7	18
ITGA2	transmembrane receptor	3.48E-08	1.32E-02	Akt,ERK,ITGA2,RHOA	CDK5R1,ESR1,F3,FAS,HBEGF,IFNG,IGF1R,ITGB3,MMP1,MMP2,MUC1,NOTCH1,ODC1,PDHA1,TJP1	3	15
CDC37	other	3.49E-08	3.52E-02	Akt,CDC37,ERK1/2,GSK3B,MAP2K1/2,RAF1,RB1	B2M,CYP17A1,CYP7A1,ESR1,F3,FAS,FN1,HBEGF,HK2,MMP1,MMP2,MUC1,NOTCH1,PDK4,PTPRE,RAC1,RRM1,TJP1,ZEB2	6	19
SDCBP	enzyme	3.49E-08	3.84E-02	c-Src,ERK1/2,P38 MAPK,SDCBP,SMAD2,SMAD3	B2M,CYP17A1,CYP7A1,DGKA,ESR1,F3,FAS,HBEGF,HK2,HMOX1,IFNG,IGF1R,ITGB3,MMP1,MMP2,MSH2,PTPRE,RAC1,RARA,XIAP	6	20
FGF1	growth factor	3.67E-08	2.63E-02	Akt,ERK1/2,FGF1,FGFR1,GSK3B,Mek,RAF1,RAS,SRC	B2M,CDK5R1,CYP17A1,CYP7A1,ESR1,F3,FAS,FN1,HBEGF,HK2,IFNG,MMP1,MMP2,MUC1,NOTCH1,PDK4,PTPRE,RAC1,SFN,TJP1	8	20
PTPRS	phosphatase	3.68E-08	1.72E-02	EGFR,NFkB (complex),PTPRS	AR,CTCF,ESR1,F3,FAS,HK2,HMOX1,HNRNPA2B1,IFNG,KYNU,MMP2,MSH2,NOTCH1,PTPRE,SENP2,XIAP,ZEB2	2	17

mir-24	microRNA	3.73E-08	5.60E-03	ERK1/2,mir-24	B2M,CYP17A1,CYP7A1,ESR1,FN1,HBEGF,HK2,IFNG,IGF1R,MMP1,MMP2,PSAP,PTPRE	2	13
MGAT1	enzyme	3.73E-08	4.80E-03	EGFR,MGAT1,SMAD2	AR,ESR1,F3,FN1,HK2,HNRNPA2B1,IFNG,ITGB3,MMP1,MMP2,PTPRE,RAC1,ZEB2	3	13
LTB4R	G-protein coupled receptor	3.78E-08	3.40E-03	ADCY,ERK,LTB4R	CDK5R1,ESR1,FAS,HBEGF,IFNG,ITGB3,MMP1,MMP2,MUC1,NFATC1,ODC1,PDK4	2	12
miR-126a-5p (and other miRNAs w/seed AUUAUUA)	mature microRNA	3.88E-08	1.52E-02	Akt,ERK1/2,HBEGF,miR-126a-5p (and other miRNAs w/seed AUUAUUA)	AR,B2M,CYP17A1,CYP7A1,ESR1,F3,FN1,HBEGF,HK2,MMP1,MMP2,MUC1,NOTCH1,PDK4,PTPRE,TJP1	3	16
ENAH	other	3.88E-08	1.51E-02	Akt,ENAH,ERK1/2,Mapk	B2M,CYP17A1,CYP7A1,ESR1,F3,FN1,HBEGF,HK2,MMP1,MMP2,MUC1,NOTCH1,NR1I3,PDK4,PTPRE,TJP1	3	16
G protein beta gamma	complex	3.92E-08	2.24E-02	Akt,ERK1/2,G protein beta gamma,PI3K (family)	B2M,CYP17A1,CYP7A1,ESR1,F3,FN1,HBEGF,HK2,HMOX1,IGF1R,MMP1,MUC1,NOTCH1,PDK4,PTPRE,TJP1,XIAP	3	17
mir-126	microRNA	4.15E-08	1.56E-02	Akt,ERK1/2,HBEGF,mir-126	AR,B2M,CYP17A1,CYP7A1,ESR1,F3,FN1,HBEGF,HK2,MMP1,MMP2,MUC1,NOTCH1,PDK4,PTPRE,TJP1	3	16
Cdk	group	4.15E-08	1.64E-02	Cdk,DAB2,ERK1/2,Jnk	B2M,BTRC,CYP17A1,CYP7A1,ESR1,HASPIN,HBEGF,HK2,IFNG,IGF1R,MMP1,MMP2,MSH2,PTPRE,RARA,XIAP	3	16
DRD2	G-protein coupled receptor	4.28E-08	1.73E-02	ADCY,Akt,DRD2,ERK,MAPK1	AR,CDK5R1,ESR1,F3,FAS,FN1,HBB,HBEGF,IFNG,IGF1R,ITGB3,MMP1,MMP2,MUC1,NFATC1,NOTCH1,ODC1,TJP1,TRIM21,XIAP	4	20
RNF41	enzyme	4.36E-08	1.08E-02	Akt,ERBB3,ERK,RNF41	CDK5R1,ESR1,F3,FAS,FN1,HBEGF,IFNG,IGF1R,ITGB3,MMP1,MMP2,MUC1,NOTCH1,ODC1,TJP1	3	15
CSNK2A1	kinase	4.40E-08	2.36E-02	CSNK2A1,ERK1/2,N-cor,NFkB (complex),P38 MAPK,RELA	B2M,CERT1,CYP7A1,DGKA,ESR1,F3,FN1,HBEGF,HK2,HMOX1,IFNG,IGF1R,ITGB3,KYNU,MSH2,NOTCH1,PPARG,PTPRE,RARA,SENP2,STK4,XIAP	5	22
IL1	group	4.44E-08	4.84E-02	ERK1/2,IL1,Jnk,NFkB (complex),P38 MAPK	B2M,BTRC,CTCF,CYP17A1,CYP7A1,DGKA,ESR1,F3,FAS,HBEGF,HK2,IFNG,IGF1R,ITGB3,KYNU,MMP1,MMP2,NOTCH1,PTPRE,RARA,SENP2	5	21
PTH	other	4.48E-08	7.40E-03	ERK,PTH,SMAD2	CDK5R1,ESR1,FAS,FN1,HBEGF,IFNG,ITGB3,MMP1,MUC1,ODC1,PDK4,RAC1,XIAP	2	13

BDKRB1	G-protein coupled receptor	4.66E-08	5.90E-03	BDKRB1,ERK,NOS2	CDK5R1,ESR1,FAS,HBEGF,IFNG,ITGB3,MMP1,MMP2,MUC1,ODC1,PDK4,TLR4	2	12
ACKR2	G-protein coupled receptor	4.66E-08	2.73E-02	ACKR2,Akt,ERK1/2,FOXO1,GSK3B	B2M,CAMK4,CYP17A1,CYP7A1,ESR1,F3,FN1,HBEGF,HK2,KIF11,MMP1,MMP2,MUC1,NOTCH1,PDK4,PTPRE,RAC1,TJP1	4	18
CCL25	cytokine	4.66E-08	2.73E-02	Akt,CCL25,ERK1/2,FOXO1,GSK3B	B2M,CAMK4,CYP17A1,CYP7A1,ESR1,F3,FN1,HBEGF,HK2,KIF11,MMP1,MMP2,MUC1,NOTCH1,PDK4,PTPRE,RAC1,TJP1	4	18
LINC00963	other	4.76E-08	1.71E-02	Akt,ERK,LINC00963,MTOR	AR,CDK5R1,CHKA,ESR1,F3,FAS,HBEGF,IFNG,IGF1R,ITGB3,MMP1,MMP2,MUC1,NOTCH1,ODC1,TJP1	3	16
S100A7	other	4.83E-08	4.44E-02	Akt,ERK,NFkB (complex),S100A7	CDK5R1,CTCF,ESR1,F3,HBEGF,HMOX1,IFNG,IGF1R,ITGB3,KYNU,MMP1,MMP2,MSH2,MUC1,NOTCH1,ODC1,SENP2,TJP1,XIAP	3	19
GAS5	other	4.83E-08	2.21E-02	Akt,AKT1,GAS5,GSK3B,MTOR,PI3K (family),RELA	AR,CERT1,CHKA,CTCF,ESR1,F3,FAS,HMOX1,IGF1R,MMP1,MMP2,MUC1,NOTCH1,PDK4,PPARG,RAC1,STK4,TJP1,XIAP	6	19
PLC	group	4.89E-08	4.99E-02	DGKA,ERK,NFkB (complex),NFKB1,PLC,RELA	CDK5R1,CERT1,ESR1,FN1,HBEGF,HMOX1,IFNG,ITGB3,KYNU,MMP1,MMP2,MSH2,MUC1,NOTCH1,ODC1,PDK4,PPARG,SENP2,STK4,WEE1,XIAP	5	21
IL27	cytokine	4.89E-08	3.16E-02	ERK1/2,IL27,STAT1,STAT3	B2M,C1R,CYP17A1,CYP7A1,ESR1,FAS,FN1,HBEGF,HK2,HMOX1,IFNG,IGF1R,MMP1,MMP2,MUC1,NFATC1,NOTCH1,PGK1,PTPRE,TRIM21,WARS1	4	21
GIT1	kinase	5.03E-08	2.19E-02	Akt,AKT1,AKT2,ERK1/2,GIT1,MMP2	B2M,CSF2RB,CYP17A1,CYP7A1,F3,FN1,HBEGF,HK2,HMOX1,MMP1,MMP2,MUC1,NOTCH1,PDK4,PPP2R1A,PTPRE,RAC1	6	17
GPR183	G-protein coupled receptor	5.05E-08	1.10E-02	ERK,ERK1/2,GPR183	B2M,CDK5R1,CYP17A1,CYP7A1,FAS,FN1,HBEGF,HK2,IFNG,IGF1R,ITGB3,MUC1,ODC1,PDK4,PTPRE	2	15
DAB2	other	5.05E-08	1.47E-02	DAB2,ERK1/2,Jnk	B2M,BTRC,CYP17A1,CYP7A1,ESR1,HBEGF,HK2,IFNG,IGF1R,MMP1,MMP2,MSH2,PTPRE,RARA,XIAP	2	15
TMEM175	ion channel	5.05E-08	7.10E-03	CTSB,CTSD,ERK,GBA,P38 MAPK,RHOA,TMEM175	CDK5R1,DGKA,ESR1,F3,FAS,FN1,HBEGF,HMOX1,IFNG,MSH2,MUC1,ODC1,PDHA1,PDK4,RARA	3	15
PTPRB	phosphatase	5.09E-08	1.64E-02	Akt,ERK1/2,FGFR1,KDR,PTPRB	B2M,CYP17A1,CYP7A1,ESR1,F3,FN1,HBEGF,HK2,MMP1,MMP2,MUC1,NOTCH1,PDK4,PTPRE,SFN,TJP1	4	16
SNCG	other	5.09E-08	1.82E-02	Akt,ERK1/2,IGF1R,SNCG	B2M,CYP17A1,CYP7A1,F3,FN1,HBEGF,HK2,IGF1R,MMP1,MMP2,MUC1,NOTCH1,PDK4,PTPRE,RAC1,TJP1	4	16

ACVRL1	kinase	5.50E-08	3.48E-02	ACVRL1,ERK1/2,ERN1,MMP14,MMP2,SMAD2,SRC,STAT3	ANG,B2M,CSF2RB,CYP17A1,CYP7A1,ESR1,FN1,HBEGF,HK2,HMOX1,IGF1R,MMP2,NOTCH1,PGK1,PPP2R1A,PTPRE,RAC1,TJP1,TLR4,XIAP	7	20
H6PD	enzyme	5.64E-08	3.53E-02	Akt,CASP4,CHUK,DDIT3,EIF2AK3,ERK1/2,H6PD,HSPA5,IL1B,IRF3,SMAD2	ACADVL,ACP3,ANG,B2M,BTRC,CYP17A1,DPP4,ESR1,FAS,FN1,HBEGF,HK2,IFNG,IGF1R,MUC1,NOTCH1,PDK4,PPARG,PTPRE,RAC1,STK4,TJP1,TLR4,XIAP	9	24
SLC37A4	transporter	5.64E-08	3.53E-02	Akt,CASP4,CHUK,DDIT3,EIF2AK3,ERK1/2,HSPA5,IL1B,IRF3,SLC37A4,SMAD2	ACADVL,ACP3,ANG,B2M,BTRC,CYP17A1,DPP4,ESR1,FAS,FN1,HBEGF,HK2,IFNG,IGF1R,MUC1,NOTCH1,PDK4,PPARG,PTPRE,RAC1,STK4,TJP1,TLR4,XIAP	9	24
GALR1	G-protein coupled receptor	5.85E-08	1.15E-02	ERK,ERK1/2,GALR1	B2M,CDK5R1,CYP17A1,CYP7A1,FAS,FN1,HBEGF,HK2,IFNG,IGF1R,ITGB3,MUC1,ODC1,PDK4,PTPRE	2	15
GPNMB	enzyme	5.86E-08	2.03E-02	c-Src,ERK,GPNMB,HIF1A,KDR	CDK5R1,CYP7A1,ERBB4,ESR1,FAS,FN1,HBEGF,HK2,HMOX1,IFNG,ITGB3,MMP1,MMP2,MUC1,NAE1,NOTCH1,ODC1,PDK4	4	18
ST6GAL1	enzyme	6.08E-08	4.08E-02	Akt,EGFR,RB1,RELA,ST6GAL1	AR,CERT1,CTCF,ESR1,FAS,FN1,HK2,HNRNPA2B1,IGF1R,MMP1,MMP2,MUC1,NOTCH1,PDK4,PPARG,PTPRE,STK4,TJP1,XIAP,ZEB2	5	20
TRIP6	cytokine	6.82E-08	3.36E-02	ERK,NFkB (complex),TRIP6	CDK5R1,CTCF,ESR1,HBEGF,HMOX1,IFNG,ITGB3,KYNU,MMP1,MMP2,MSH2,MUC1,NOTCH1,ODC1,PDK4,SENP2,XIAP	2	17
SENP2	peptidase	7.24E-08	3.17E-02	Akt,NFkB (complex),SENP2	CTCF,F3,FAS,HK2,HMOX1,IFNG,IGF1R,KYNU,MMP1,MMP2,MSH2,MUC1,NOTCH1,PDK4,PGK1,TJP1,XIAP	3	17
CLTC	other	7.24E-08	2.74E-02	Akt,CLTC,NFkB (complex)	CD74,CTCF,F3,FAS,HMOX1,IFNG,IGF1R,KYNU,MMP1,MMP2,MSH2,MUC1,NOTCH1,PDK4,SENP2,TJP1,XIAP	3	17
GADD45B	other	7.24E-08	3.52E-02	ERK,GADD45B,NFkB (complex)	CDK5R1,CTCF,ESR1,HBEGF,HMOX1,IFNG,ITGB3,KYNU,MMP1,MMP2,MSH2,MUC1,NOTCH1,ODC1,PDK4,SENP2,XIAP	2	17
BRAF	kinase	7.39E-08	4.94E-02	BRAF,ERK,ERK1/2,MAP2K1/2,Mek,MMP2,RAF1	B2M,CDK5R1,CSF2RB,CYP17A1,CYP7A1,ESR1,FN1,HK2,IFNG,IGF1R,ITGB3,MMP1,MMP2,MUC1,ODC1,PDK4,PPP2R1A,PTPRE,RRM1,TJP1	6	20
IGFBP6	other	7.54E-08	2.84E-02	ERK,IGFBP6,MAPK8,P38 MAPK	CDK5R1,DGKA,ESR1,F3,FN1,HBEGF,HMOX1,IFNG,ITGB3,MMP1,MMP2,MSH2,MUC1,ODC1,PDK4,RARA	3	16
CCL2	cytokine	7.69E-08	2.94E-02	CCL2,ERK,ERK1/2,MAPK8	B2M,CDK5R1,CYP17A1,CYP7A1,ESR1,FAS,FN1,HK2,IGF1R,ITGB3,MMP1,MMP2,MUC1,ODC1,PDK4,PTPRE,RARA	4	17
ZDHHC6	enzyme	7.85E-08	1.43E-02	Akt,ERK,ZDHHC6	CDK5R1,ESR1,F3,FAS,HBEGF,IFNG,IGF1R,ITGB3,MMP1,MMP2,MUC1,NOTCH1,ODC1,TJP1	2	14

NTRK3	kinase	7.85E-08	1.43E-02	Akt,ERK,NTRK3	CDK5R1,ESR1,F3,FAS,HBEGF,IFNG,IGF1R,ITGB3,MMP1,MMP2,MUC1,NOTCH1,ODC1,TJP1	2	14
TLR8	transmembrane receptor	7.91E-08	4.79E-02	ERK1/2,NFkB (complex),P38 MAPK,RELA,TLR8	B2M,CERT1,CYP17A1,CYP7A1,DGKA,ESR1,F3,FAS,HBEGF,HK2,IFNG,IGF1R,ITGB3,KYNU,MMP1,MMP2,NOTCH1,PPARG,PTPRE,RARA,SENP2,STK4,XIAP	4	23
CDH13	other	8.34E-08	1.90E-02	Akt,CDH13,EGFR,GSK3B	AR,F3,HK2,HNRNPA2B1,IFNG,IGF1R,MMP1,MMP2,MUC1,NOTCH1,PDK4,PTPRE,TJP1,XIAP,ZEB2	3	15
FLT3LG	cytokine	8.48E-08	1.48E-02	Akt,ERK,FLT3LG	CDK5R1,ESR1,F3,FAS,HBEGF,IFNG,IGF1R,ITGB3,MMP1,MMP2,MUC1,NOTCH1,ODC1,TJP1	2	14
FGFR3-TACC3	fusion gene/product	8.48E-08	1.52E-02	Akt,ERK,FGFR3-TACC3	CDK5R1,ESR1,F3,FAS,HBEGF,IFNG,IGF1R,ITGB3,MMP1,MMP2,MUC1,NOTCH1,ODC1,TJP1	2	14
PPP2R5E	phosphatase	8.57E-08	1.44E-02	ERK,NFkB (complex),PPP2R5E	CDK5R1,CTCF,ESR1,FAS,FN1,HBEGF,HMOX1,ITGB3,KYNU,MSH2,MUC1,NOTCH1,ODC1,PDK4,SENP2,XIAP	3	16
TFF3	other	8.59E-08	2.18E-02	Akt,EGFR,ERBB2,TFF3	BMP1,ERBB4,ESR1,FN1,GART,HK2,HNRNPA2B1,IFNG,IGF1R,MMP1,MMP2,MUC1,NOTCH1,PDK4,PGK1,PPARG,PTPRE,PTPRF,RRM1,SMTN,TJP1,XIAP,ZEB2	3	23
IL5	cytokine	8.59E-08	4.85E-02	Akt,ERK1/2,IL5,JAK2,Mapk,MAPK1,RAS,STAT5a/b	AR,B2M,CSF2RB,CYP17A1,CYP7A1,ESR1,F3,FN1,HBB,HBEGF,HK2,IFNG,MMP1,MMP2,MUC1,NOTCH1,NR1I3,PDK4,PTPRE,RARA,TJP1,TRIM21,XIAP	8	23
LGALS8	other	8.65E-08	2.31E-02	ERK1/2,LGALS8,RELA	B2M,CERT1,CTCF,CYP17A1,CYP7A1,ESR1,FAS,FN1,HBEGF,HK2,IFNG,IGF1R,MMP1,MMP2,PPARG,PTPRE,STK4	2	17
CAT	enzyme	8.65E-08	3.99E-02	CAT,ERK,NFkB (complex)	CDK5R1,CTCF,ESR1,HBEGF,HMOX1,IFNG,ITGB3,KYNU,MMP1,MMP2,MSH2,MUC1,NOTCH1,ODC1,PDK4,SENP2,XIAP	2	17
ADTRP	enzyme	8.94E-08	1.47E-02	ADTRP,ERK1/2,F10,MAPK8,MMP2,MMP9,TFPI	B2M,CSF2RB,CYP17A1,CYP7A1,ESR1,FN1,HBEGF,HK2,IGF1R,MMP1,MMP2,PPP2R1A,PTPRE,RARA,TJP1	5	15
KL	enzyme	9.58E-08	1.69E-02	Akt,ERK1/2,KL	B2M,CYP17A1,CYP7A1,ESR1,F3,FN1,HBEGF,HK2,IGF1R,MMP1,MUC1,NOTCH1,PDK4,PTPRE,TJP1	2	15
KDR	kinase	9.73E-08	2.91E-02	Akt,CASP1,ERK1/2,KDR,SRC (family)	B2M,CYP17A1,CYP7A1,ESR1,F3,HBEGF,HK2,IFNG,ITGB3,MMP1,MMP2,MUC1,NOTCH1,PDK4,PTPRE,TJP1	5	16
FDPS	enzyme	9.88E-08	1.61E-02	Akt,ERK,FDPS	CDK5R1,ESR1,F3,FAS,HBEGF,IFNG,IGF1R,ITGB3,MMP1,MMP2,MUC1,NOTCH1,ODC1,TJP1	2	14

SPINK7	other	1.03E-07	8.40E-03	ERK,SPINK7,SRC	CDK5R1,ESR1,FAS,HBEGF,IFNG,IGF1R,ITGB3,MMP1,MMP2,MUC1,ODC1,PDK4	2	12
ANGPT4	growth factor	1.03E-07	1.93E-02	Akt,ANGPT4,ERK1/2	B2M,CYP17A1,CYP7A1,ESR1,F3,FN1,HBEGF,HK2,MMP1,MMP2,MUC1,NOTCH1,PDK4,PTPRE,TJP1	2	15
PIK3R5	kinase	1.03E-07	1.93E-02	Akt,ERK1/2,PIK3R5	B2M,CYP17A1,CYP7A1,ESR1,F3,FN1,HBEGF,HK2,MMP1,MMP2,MUC1,NOTCH1,PDK4,PTPRE,TJP1	2	15
MOSPD2	other	1.03E-07	1.93E-02	Akt,ERK1/2,MOSPD2	B2M,CYP17A1,CYP7A1,ESR1,F3,FN1,HBEGF,HK2,MMP1,MMP2,MUC1,NOTCH1,PDK4,PTPRE,TJP1	2	15
IL2RB	transmembrane receptor	1.03E-07	1.93E-02	Akt,ERK1/2,IL2RB	B2M,CYP17A1,CYP7A1,ESR1,F3,FN1,HBEGF,HK2,MMP1,MMP2,MUC1,NOTCH1,PDK4,PTPRE,TJP1	2	15
LCP2	other	1.03E-07	2.79E-02	ERK,Jnk,LCP2,PIK3R1,RAC1	AR,BTRC,CDK5R1,ESR1,FAS,HBEGF,ITGB3,MMP1,MMP2,MSH2,MUC1,ODC1,PDK4,RARA,XIAP	4	15
PRR14	other	1.07E-07	1.77E-02	Akt,ERK,PRR14	CDK5R1,ESR1,F3,FAS,HBEGF,IFNG,IGF1R,ITGB3,MMP1,MMP2,MUC1,NOTCH1,ODC1,TJP1	2	14
STAM	other	1.10E-07	1.98E-02	Akt,ERK1/2,STAM	B2M,CYP17A1,CYP7A1,ESR1,F3,FN1,HBEGF,HK2,MMP1,MMP2,MUC1,NOTCH1,PDK4,PTPRE,TJP1	2	15
CDH2	other	1.10E-07	1.99E-02	Akt,CDH2,ERK1/2	B2M,CYP17A1,CYP7A1,ESR1,F3,FN1,HBEGF,HK2,MMP1,MMP2,MUC1,NOTCH1,PDK4,PTPRE,TJP1	2	15
FGF7	growth factor	1.10E-07	2.19E-02	Akt,ERK,FGF7,MAPK8	CDK5R1,ESR1,F3,FAS,HBEGF,IFNG,IGF1R,ITGB3,MMP1,MMP2,MUC1,NOTCH1,ODC1,RARA,TJP1	3	15
CLEC4A	transmembrane receptor	1.18E-07	2.08E-02	Akt,CLEC4A,ERK1/2	B2M,CYP17A1,CYP7A1,ESR1,F3,FN1,HBEGF,HK2,MMP1,MMP2,MUC1,NOTCH1,PDK4,PTPRE,TJP1	2	15
CLEC4C	other	1.26E-07	2.19E-02	Akt,CLEC4C,ERK1/2	B2M,CYP17A1,CYP7A1,ESR1,F3,FN1,HBEGF,HK2,MMP1,MMP2,MUC1,NOTCH1,PDK4,PTPRE,TJP1	2	15
LAMTOR1	other	1.29E-07	3.64E-02	AKT1,ERK1/2,Gsk3,LAMTOR1,MTOR,MTORC1,RHOA,RICTOR,RPS6KB1,SREBF1,SREBF2	AR,B2M,CHKA,CYP17A1,CYP7A1,ESR1,FABP3,FABP7,FN1,GTF2I,HBEGF,HK2,HMOX1,IFNG,MMP2,MUC1,PDHA1,PPARG,PTPRE	9	19
IL1RAP	transmembrane receptor	1.33E-07	2.14E-02	Akt,ERK,IL1RAP	CDK5R1,ESR1,F3,FAS,HBEGF,IFNG,IGF1R,ITGB3,MMP1,MMP2,MUC1,NOTCH1,ODC1,TJP1	2	14
CXCL13	cytokine	1.34E-07	2.21E-02	Akt,CXCL13,ERK1/2	B2M,CYP17A1,CYP7A1,ESR1,F3,FN1,HBEGF,HK2,MMP1,MMP2,MUC1,NOTCH1,PDK4,PTPRE,TJP1	2	15

GNA11	enzyme	1.41E-07	3.15E-02	Akt,AKT1,ERK1/2,GNA11,RHOA	B2M,CYP17A1,CYP7A1,ESR1,F3,FN1,HBEGF,HK2,HMOX1,MMP2,MUC1,NOTCH1,PDHA1,PDK4,PTPRE,TJP1	4	16
COP1	enzyme	1.43E-07	2.04E-02	Akt,COP1,ERK,SFN	CDK5R1,DSG2,ESR1,F3,FAS,HBEGF,IFNG,IGF1R,ITGB3,MMP1,MMP2,MUC1,NOTCH1,ODC1	3	14
PRKD3	kinase	1.54E-07	1.94E-02	Akt,ERK,PRKD3,RPS6KB1	CDK5R1,ESR1,F3,FAS,HBEGF,IFNG,IGF1R,ITGB3,MMP1,MMP2,MUC1,NOTCH1,ODC1,TJP1	3	14
LINC00461	other	1.64E-07	2.63E-02	Akt,ERK1/2,LINC00461	B2M,CYP17A1,CYP7A1,ESR1,F3,FN1,HBEGF,HK2,MMP1,MMP2,MUC1,NOTCH1,PDK4,PTPRE,TJP1	2	15
FXN	kinase	1.64E-07	1.17E-02	ERK1/2,FXN,P38 MAPK	B2M,CYP17A1,CYP7A1,DGKA,ESR1,F3,FAS,HBEGF,HK2,HMOX1,IGF1R,ITGB3,MSH2,PTPRE,RARA	2	15
ERK	group	1.72E-07	9.70E-03	ERK	CDK5R1,ESR1,FAS,HBEGF,IFNG,ITGB3,MMP1,MMP2,MUC1,ODC1,PDK4	1	11
CUL5	ion channel	1.76E-07	3.48E-02	Akt,CUL5,EGFR,ERBB2	BMP1,ERBB4,ESR1,FN1,GART,HK2,HNRNPA2B1,IFNG,IGF1R,MMP1,MMP2,MUC1,NOTCH1,PDK4,PGK1,PPARG,PTPRE,PTPRF,RRM1,SMTN,TJP1,XIAP,ZEB2	3	23
LBP	transporter	1.81E-07	4.01E-02	IRF3,Jnk,LBP,P38 MAPK,STAT1,TYK2	BTRC,C1R,DGKA,F3,FAS,HMOX1,IFNG,ITGB3,MMP1,MMP2,MSH2,MUC1,RARA,STK4,TRIM21,WARS1,XIAP	5	17
BTRC	enzyme	1.81E-07	3.07E-02	ATF4,BTRC,MDM2,MTOR,NFkB (complex),NFKB2,PHLPP1	AR,CHKA,CTCF,FAS,FN1,HMOX1,IFNG,IGF1R,KYNU,MMP1,MMP2,MSH2,MUC1,NOTCH1,PRKCB,SENP2,SFN	6	17
CD2	transmembrane receptor	1.91E-07	1.54E-02	CD2,ERK1/2,Mapk,STAT5a/b	B2M,CYP17A1,CYP7A1,ESR1,FN1,HBEGF,HK2,IFNG,IGF1R,MMP1,MMP2,NR1I3,PTPRE,RARA	4	14
HP	peptidase	1.91E-07	4.20E-03	EGFR,HP	AR,ESR1,F3,HK2,HMOX1,HNRNPA2B1,IFNG,MMP1,PTPRE,XIAP,ZEB2	2	11
BCAN	other	1.91E-07	5.00E-03	BCAN,EGFR	AR,ESR1,F3,HK2,HNRNPA2B1,IFNG,ITGB3,MMP1,PTPRE,XIAP,ZEB2	2	11
SLC36A4	transporter	2.02E-07	1.94E-02	Akt,AKT1,EPRS1,MTOR,MTORC1,PAK1,RHOA,RICTOR,RPS6KB1,SLC36A4,SREBF1,SREBF2	AR,CHKA,F3,FABP3,FABP7,FN1,GTF2I,HK2,HMOX1,IFNG,MMP2,MUC1,NOTCH1,PDHA1,PDK4,PPARG,TJP1	10	17
SLC36A1	transporter	2.02E-07	1.94E-02	Akt,AKT1,EPRS1,MTOR,MTORC1,PAK1,RHOA,RICTOR,RPS6KB1,SLC36A1,SREBF1,SREBF2	AR,CHKA,F3,FABP3,FABP7,FN1,GTF2I,HK2,HMOX1,IFNG,MMP2,MUC1,NOTCH1,PDHA1,PDK4,PPARG,TJP1	10	17
ANXA5	transporter	2.05E-07	2.39E-02	ANXA5,ERK1/2,MAP2K1/2,RAF1,RAS	B2M,CYP17A1,CYP7A1,ESR1,FAS,FN1,HBEGF,HK2,IFNG,IGF1R,MMP1,MMP2,PTPRE,RRM1	4	14
RBP4	other	2.15E-07	2.66E-02	ERK1/2,P38 MAPK,RBP4	B2M,CYP17A1,CYP7A1,DGKA,ESR1,F3,FAS,FN1,HBEGF,HK2,HMOX1,IGF1R,ITGB3,MSH2,PTPRE,RARA	3	16

PPP2R2A	phosphatase	2.20E-07	1.95E-02	AP2M1,ERK1/2,MAP2K1/2,PPP2R2A	B2M,CD74,CYP17A1,CYP7A1,ESR1,FAP,FN1,HBEGF,HK2,IGF1R,MMP1,MMP2,PTPRE,RRM1	4	14
LOC102724788/PRODH	enzyme	2.26E-07	2.59E-02	CTNNB1,EGFR,GSK3B,LOC102724788/PRODH	AR,ESR1,F3,FN1,HBEGF,HK2,HNRNPA2B1,IFNG,IGF1R,MMP1,MMP2,PTPRE,RAC1,XIAP,ZEB2	3	15
SMO	G-protein coupled receptor	2.26E-07	4.56E-02	Akt,Mapk,NFKB1,PRKCB,RELA,SMO	CERT1,CTCF,F3,FAS,FN1,HBB,IFNG,IGF1R,MMP1,MMP2,MUC1,NOTCH1,NR1I3,PDK4,PPARG,STK4,TJP1	6	17
ANO1	ion channel	2.34E-07	5.60E-03	ANO1,EGFR	AR,ESR1,F3,HK2,HNRNPA2B1,IFNG,MMP1,NOTCH1,PTPRE,XIAP,ZEB2	2	11
EGLN	group	2.34E-07	5.70E-03	AKT1,EGLN,Gsk3,GSK3B,MTOR,MTORC1,RHOA,RPS6KB1,SREBF1,SREBF2	AR,CHKA,ESR1,FABP3,FABP7,GTF2I,HK2,HMOX1,IFNG,IGF1R,MUC1,PDHA1,PDK3,PPARG,PYGL,SH2B2,XIAP,ZNF292	9	18
EPRS1	enzyme	2.34E-07	1.74E-02	AKT1,EPRS1,Gsk3,GSK3B,MTOR,MTORC1,RHOA,RICTOR,SREBF1,SREBF2,STAT1,STAT6	AR,C1R,CHKA,ESR1,FABP3,FABP7,FAS,FN1,GTF2I,HK2,HMOX1,IFNG,IGF1R,PDHA1,PPARG,TRIM21,WARS1,XIAP	10	18
EDNRB	G-protein coupled receptor	2.41E-07	3.27E-02	Akt,EDNRB,ERK1/2	B2M,CYP17A1,CYP7A1,ESR1,F3,FN1,HBEGF,HK2,MMP1,MMP2,MUC1,NOTCH1,PDK4,PTPRE,TJP1	2	15
TNC	other	2.41E-07	3.19E-02	Akt,ERK1/2,TNC	B2M,CYP17A1,CYP7A1,ESR1,F3,FN1,HBEGF,HK2,MMP1,MMP2,MUC1,NOTCH1,PDK4,PTPRE,TJP1	3	15
mir-181	microRNA	2.57E-07	2.81E-02	mir-181,MMP2,MMP9,NFkB (complex)	AR,CSF2RB,CTCF,FAS,HMOX1,IFNG,KYNU,MMP1,MMP2,MSH2,NOTCH1,PPP2R1A,SENP2,TJP1,XIAP	4	15
DLG1	kinase	2.57E-07	4.03E-02	Akt,DLG1,ERK1/2	B2M,CYP17A1,CYP7A1,ESR1,F3,FN1,HBEGF,HK2,IGF1R,MMP1,MUC1,NOTCH1,PDK4,PTPRE,TJP1	2	15
ITGA4	transmembrane receptor	2.57E-07	3.39E-02	Akt,ERK1/2,ITGA4	B2M,CYP17A1,CYP7A1,ESR1,F3,FN1,HBEGF,HK2,MMP1,MMP2,MUC1,NOTCH1,PDK4,PTPRE,TJP1	2	15
AGT	growth factor	2.58E-07	5.60E-03	AGT,EGFR	AR,ESR1,F3,HK2,HNRNPA2B1,IFNG,MMP1,PPARG,PTPRE,XIAP,ZEB2	2	11
mir-96	microRNA	2.58E-07	3.49E-02	BCR-ABL1,GSK3B,mir-96,NFkB (complex),STAT1,STAT5a/b	C1R,CTCF,FN1,HMOX1,IFNG,IGF1R,KYNU,MMP1,MMP2,MSH2,MUC1,NOTCH1,RAC1,RARA,SENP2,TRIM21,WARS1,XIAP	5	18
RORA	ligand-dependent nuclear receptor	2.71E-07	3.92E-02	CYP19A1,Jnk,P38 MAPK,RORA,Sod	BTRC,DGKA,ESR1,F3,FAS,FN1,HMOX1,IFNG,ITGB3,MMP1,MMP2,MSH2,RARA,XIAP	3	14

PLD2	enzyme	2.74E-07	3.01E-02	Akt,ERK1/2,PLD2	B2M,CYP17A1,CYP7A1,ESR1,F3,FN1,HBEGF,HK2,MMP1,MMP2,MUC1,NOTCH1,PDK4,PTPRE,TJP1	2	15
HSPB1	other	2.79E-07	2.05E-02	ERK1/2,HSF1,HSPB1,Jnk	B2M,BTRC,CYP17A1,CYP7A1,ESR1,FN1,HBEGF,HK2,HSP90AB1,IFNG,IGF1R,MSH2,PGK1,PTPRE,RARA,TTR,XIAP	3	17
F7	peptidase	2.87E-07	4.53E-02	AKT1,EIF4E,ERK1/2,F7,GSK3B,MAPK8,RPS6KB1	AR,B2M,CYP17A1,CYP7A1,F3,FN1,HBEGF,HK2,HMOX1,MMP1,MMP2,MUC1,PTPRE,RAC1,RARA,XIAP	7	16
DICER1	enzyme	3.02E-07	1.27E-02	DICER1,EGFR,MET	AR,ESR1,F3,FN1,HK2,HMOX1,HNRNPA2B1,IFNG,MMP1,MMP2,PTPRE,XIAP,ZEB2	3	13
GRB7	other	3.03E-07	1.39E-02	ERK1/2,GRB7,RAS	B2M,CYP17A1,CYP7A1,ESR1,FN1,HBEGF,HK2,IFNG,IGF1R,MMP1,MMP2,PTPRE	2	12
SHC4	other	3.03E-07	1.39E-02	ERK1/2,RAS,SHC4	B2M,CYP17A1,CYP7A1,ESR1,FN1,HBEGF,HK2,IFNG,IGF1R,MMP1,MMP2,PTPRE	2	12
OPA1	enzyme	3.21E-07	3.46E-02	Akt,ERK1/2,GLB1,LIPE,OPA1,PI3K (complex),Pkc(s),PNPLA2	B2M,CYP17A1,CYP7A1,ESR1,F3,FN1,HBEGF,HK2,MMP1,MUC1,NOTCH1,PC,PDK4,PTPRE,TJP1,XIAP	5	16
ORAI1	ion channel	3.50E-07	4.82E-02	CD3E,ERK1/2,LCK,MAP2K1,ORAI1,PTPN6,RAF1	B2M,CYP17A1,CYP7A1,ESR1,F3,FAS,FN1,HBEGF,HK2,IFNG,IGF1R,MMP1,MMP2,PPARG,PTPRE	6	15
MARK4	kinase	3.50E-07	1.39E-02	AKT1,Gsk3,GSK3B,MARK4,MTOR,MTORC1,RHOA,RICTOR,RPS6KB1,SREBF1,SREBF2,TEAD,YAP/TAZ	AR,CHKA,ESR1,FABP3,FABP7,FN1,GTF2I,HK2,HMOX1,IFNG,IGF1R,MUC1,PDHA1,PPARG,XIAP	10	15
FLT1	kinase	3.51E-07	2.13E-02	ERK1/2,FLT1,Ras homolog,SRC (family)	B2M,CYP17A1,CYP7A1,ESR1,HBEGF,HK2,IGF1R,ITGB3,MMP1,MMP2,NOTCH1,PTPRE,TJP1	4	13
HTR7	G-protein coupled receptor	3.58E-07	1.16E-02	ADCY,ERK1/2,HTR7	B2M,CYP17A1,CYP7A1,ESR1,FN1,HBEGF,HK2,IGF1R,MMP1,MMP2,NFATC1,PTPRE	2	12
PHB	transcription regulator	3.59E-07	4.21E-02	AR,CTNNB1,ERK1/2,MMP2,NFkB (complex),PHB	B2M,CSF2RB,CTCF,CYP17A1,CYP7A1,ESR1,FN1,HBEGF,HK2,HMOX1,IFNG,KYNU,MMP2,MSH2,NOTCH1,PPP2R1A,PTPRE,SENP2,SMTN,TJP1,TLR4,WEE1,XIAP	6	23
HMMR	transmembrane receptor	3.78E-07	2.31E-02	ERK1/2,HMMR,SMAD2	B2M,CYP17A1,CYP7A1,ESR1,FN1,HBEGF,HK2,IGF1R,MMP1,MMP2,PTPRE,RAC1,XIAP	3	13
TRIO	kinase	4.06E-07	5.00E-02	Jnk,P38 MAPK,RAC1,RHOA,TRIO	BTRC,DGKA,F3,FAS,FN1,HMOX1,IFNG,ITGB3,MMP1,MMP2,MSH2,PDHA1,RARA,XIAP	4	14

mir-223	microRNA	4.58E-07	1.05E-02	EGFR,mir-223,SP3	AR,ESR1,F3,HK2,HMGCS1,HNRNPA2B1,IFNG,IGF1R,MMP1,PTPRE,XIAP,ZEB2	3	12
ELF3	transcription regulator	4.58E-07	1.57E-02	ELF3,ERK1/2	B2M,CYP17A1,CYP7A1,ESR1,FN1,HBEGF,HK2,IGF1R,MMP1,MMP2,PPARG,PTPRE	2	12
BCR-ABL1	fusion gene/product	4.93E-07	3.88E-02	BCR-ABL1,GSK3B,NFkB (complex),STAT1,STAT5a/b	C1R,CTCF,HMOX1,IFNG,IGF1R,KYNU,MMP1,MMP2,MSH2,MUC1,NOTCH1,RAC1,RARA,SENP2,TRIM21,WARS1,XIAP	4	17
FPR1	G-protein coupled receptor	5.05E-07	3.46E-02	EGFR,FPR1,Jnk	AR,BTRC,ESR1,F3,HK2,HNRNPA2B1,MMP1,MMP2,MSH2,PTPRE,RARA,XIAP,ZEB2	2	13
PTPN12	phosphatase	5.49E-07	8.50E-03	ABL1,EGFR,PTPN12	AR,ESR1,F3,FAS,HK2,HNRNPA2B1,IFNG,MMP1,PTPRE,XIAP,ZEB2	2	11
HCAR2	G-protein coupled receptor	5.82E-07	1.47E-02	ERK1/2,HCAR2	B2M,CYP17A1,CYP7A1,ESR1,FN1,HBEGF,HK2,IGF1R,MMP1,MMP2,PTPRE,WEE1	2	12
DPP4	peptidase	5.97E-07	3.71E-02	DPP4,ERK1/2,P38 MAPK,PARP1	B2M,CYP17A1,CYP7A1,DGKA,ESR1,F3,FAS,FN1,HBEGF,HK2,HMOX1,IGF1R,MSH2,PTPRE,RARA	3	15
PTPRE	phosphatase	5.98E-07	4.30E-02	Akt,ERK1/2,PTPRE	B2M,CYP17A1,CYP7A1,ESR1,F3,FN1,HBEGF,HK2,MMP1,MMP2,MUC1,NOTCH1,PDK4,TJP1	2	14
mir-146	microRNA	6.01E-07	8.20E-03	EGFR,mir-146	AR,ESR1,F3,HK2,HNRNPA2B1,IFNG,MMP1,PTPRE,TLR4,XIAP,ZEB2	2	11
TRPM2	ion channel	6.24E-07	2.23E-02	AKT1,ERK1/2,PTK2B,TRPM2	B2M,CYP17A1,CYP7A1,ESR1,FN1,HBEGF,HK2,HMOX1,IFNG,IGF1R,MMP1,MMP2,PTPRE	3	13
ADAM15	peptidase	6.57E-07	9.60E-03	ADAM15,EGFR,MMP9	AR,ESR1,F3,HK2,HNRNPA2B1,IFNG,MMP1,PTPRE,TJP1,XIAP,ZEB2	3	11
VEGFD	growth factor	6.81E-07	2.00E-02	ERK1/2,KDR,VEGFD	B2M,CYP17A1,CYP7A1,ESR1,FN1,HBEGF,HK2,IGF1R,MMP1,MMP2,NOTCH1,PTPRE	2	12
TRIM37	enzyme	7.22E-07	3.58E-02	NFkB (complex),TRIM37	CTCF,CUL1,FAS,HMOX1,IFNG,KYNU,MMP1,MMP2,MSH2,NOTCH1,PEX5,SENP2,SFN,XIAP	2	14
RUNX1	transcription regulator	8.16E-07	3.91E-02	NFkB (complex),RUNX1	ALOX12,CTCF,EP300,FAS,HMOX1,IFNG,ITGB3,KYNU,MMP1,MMP2,MSH2,NOTCH1,SENP2,XIAP	2	14
DDIT3	transcription regulator	8.56E-07	2.68E-02	DDIT3,ERK1/2	B2M,CYP17A1,CYP7A1,ESR1,FN1,HBEGF,HK2,IFNG,IGF1R,MMP1,MMP2,PTPRE	2	12
RIPK3	kinase	8.68E-07	3.93E-02	MAPKAPK2,MLKL,MMP2,MMP9,NFkB (complex),RIPK3	CSF2RB,CTCF,FAS,HMOX1,IFNG,KYNU,MMP1,MMP2,MSH2,NOTCH1,PPP2R1A,SENP2,TJP1,XIAP	3	14

PDK4	kinase	8.78E-07	3.33E-02	ERK1/2,KRAS,PDK4	B2M,CYP17A1,CYP7A1,ESR1,FN1,HBEGF,HK2,IGF1R,MMP1,MMP2,PC,PTPRE,YY1	3	13
CDH17	transporter	9.95E-07	3.07E-02	CDH17,ERK1/2,ITGB1	B2M,CYP17A1,CYP7A1,ESR1,FN1,HBEGF,HK2,IGF1R,ITGB3,MMP1,MMP2,PTPRE	2	12
NDFIP1	other	9.95E-07	3.54E-02	Akt,Jnk,NDFIP1	BTRC,F3,IFNG,IGF1R,MMP1,MSH2,MUC1,NOTCH1,PDK4,RARA,TJP1,XIAP	2	12
AHSA1	other	1.00E-06	4.70E-02	AHSA1,ERK1/2,MAP2K1/2,RAF1	B2M,CYP17A1,CYP7A1,ESR1,FAS,FN1,HBEGF,HK2,IGF1R,MMP1,MMP2,PTPRE,RRM1	3	13
HES1	transcription regulator	1.00E-06	4.40E-02	AKT1,ERK1/2,HES1,PRKAA	AR,B2M,CYP17A1,CYP7A1,ESR1,FN1,HBEGF,HK2,HMOX1,IGF1R,MMP1,MMP2,PTPRE	3	13
SNAPIN	other	1.17E-06	3.93E-02	P38 MAPK,SNAPIN,STAT1	C1R,DGKA,F3,FAS,FN1,HMOX1,ITGB3,MMP1,MMP2,MSH2,MUC1,RARA,TRIM21,WARS1	2	14
MTORC1	complex	1.24E-06	1.93E-02	AKT1,Gsk3,GSK3B,MTOR,MTORC1,RHOA,RICTOR,RPS6KB1,SREBF1,SREBF2	AR,CHKA,ESR1,FABP3,FABP7,GTF2I,HK2,HMOX1,IFNG,IGF1R,MUC1,PDHA1,PPARG,XIAP	9	14
CCR2	G-protein coupled receptor	1.39E-06	4.08E-02	CCR2,ERK1/2,SMAD3	B2M,CYP17A1,CYP7A1,ESR1,HBEGF,HK2,IFNG,IGF1R,MMP1,MMP2,PTPRE,RAC1,XIAP	2	13
EGFR	kinase	1.40E-06	1.20E-02	EGFR	AR,ESR1,F3,HK2,HNRNPA2B1,IFNG,MMP1,PTPRE,XIAP,ZEB2	1	10
NTN1	growth factor	1.54E-06	3.94E-02	Creb,ERK1/2,NTN1,RHOA	B2M,CYP17A1,CYP7A1,ESR1,FN1,HBEGF,HK2,IGF1R,MMP1,MMP2,PDHA1,PTPRE	3	12
ERK1/2	group	1.65E-06	2.75E-02	ERK1/2	B2M,CYP17A1,CYP7A1,ESR1,FN1,HBEGF,HK2,IGF1R,MMP1,MMP2,PTPRE	1	11
FGF8	growth factor	2.15E-06	3.38E-02	FGF8,FGFR1,P38 MAPK	DGKA,F3,FAS,FN1,HK2,HMOX1,ITGB3,MMP1,MMP2,MSH2,RARA,SFN,SUB1	3	13
miR-193a-3p (and other miRNAs w/seed ACUGGCC)	mature microRNA	3.04E-06	3.75E-02	Akt,miR-193a-3p (and other miRNAs w/seed ACUGGCC),MTOR	AR,CHKA,ERBB4,F3,IFNG,IGF1R,MMP2,MUC1,NOTCH1,PDK4,TJP1	3	11
mir-193	microRNA	3.52E-06	3.97E-02	Akt,mir-193,MTOR	AR,CHKA,ERBB4,F3,IFNG,IGF1R,MMP2,MUC1,NOTCH1,PDK4,TJP1	3	11
ATF3	transcription regulator	6.57E-06	3.68E-02	Akt,ATF3	DCTD,F3,IFNG,IGF1R,MMP2,MSH2,MUC1,NOTCH1,PDK4,RRM1,TJP1	2	11

PCGEM1	other	2.24E-05	8.00E-04	PCGEM1	CANT1,G6PD,HK2,PDHA1,PDK4,PGK1	1	6
DDX5	enzyme	2.38E-05	1.15E-02	DDX5,let-7	AR,FN1,HK2,IGF1R,ITGB3,MAPK6,MDH1,NDUFAB1,PA2G4,STARD13,TLR4	2	11
FENDRR	other	3.28E-05	2.24E-02	FENDRR,MMP2,MMP9,STAT1	C1R,CSF2RB,FAS,FN1,MUC1,PPP2R1A,TJP1,TRIM21,WARS1	4	9
MCAM	other	3.40E-05	3.20E-03	MCAM,MMP2	CSF2RB,FN1,MMP2,PPP2R1A,TJP1	2	5
let-7	microRNA	3.51E-05	8.60E-03	let-7	AR,FN1,HK2,IGF1R,ITGB3,MAPK6,PA2G4,STARD13,TLR4	1	9
ZFYVE9	peptidase	4.98E-05	2.67E-02	ERN1,SMAD2,ZFYVE9	ANG,FN1,MMP2,RAC1,TJP1,XIAP	3	6
WAC	other	5.41E-05	1.20E-03	WAC	G6PD,PDK4,PPARG	1	3
SP1	transcription regulator	7.55E-05	4.85E-02	SP1	ESR1,FN1,HBEGF,HK2,HMOX1,IGF1R,ITGB3,ZEB2	1	8
ZFYVE9	peptidase	8.01E-05	3.66E-02	SMAD2,ZFYVE9	FN1,MMP2,RAC1,TJP1,XIAP	2	5
ESRRG	ligand-dependent nuclear receptor	9.33E-05	2.40E-03	ESRRG	HK2,KLK1,MDH1,PDHA1,PDK4	1	5
ESRRA	transcription regulator	1.10E-04	1.34E-02	CYP19A1,ESRRA	ESR1,HK2,HMOX1,PNMT,PPARG,RARA	2	6
RNF40	enzyme	1.48E-04	5.00E-03	RNF40	G6PD,PDK4,PPARG	1	3
OLR1	transmembrane receptor	2.20E-04	2.30E-03	OLR1	MMP1,RARA,RARG	1	3
DDR2	kinase	2.39E-04	2.01E-02	DDR2,MMP2	CSF2RB,MMP1,MMP2,PPP2R1A,TJP1	2	5
DEF6	other	3.11E-04	4.66E-02	DEF6	FN1,MMP2,ZEB2	1	3
SFN	other	3.32E-04	2.81E-02	SFN	DSG2,F3,PDK4,TJP1	1	4
PTGS1	enzyme	3.91E-04	1.52E-02	MMP2,MMP9,PTGS1	CSF2RB,MMP2,PPP2R1A,TJP1	3	4
SRSF1	other	3.91E-04	2.31E-02	EIF4E,EIF4EBP1,SRSF1	ESR1,FN1,RAC1,XIAP	2	4
IKBKE	kinase	4.18E-04	3.56E-02	IKBKE	PDHA1,PDK3,PDK4,PGK1,PYGL	1	5
GPI	enzyme	4.23E-04	2.62E-02	GPI	FN1,RAC1,ZEB2	1	3
WT1	transcription regulator	4.26E-04	2.84E-02	WT1	BTK,ESR1,HBEGF,IGF1R,ODC1,TRIM21,WARS1	1	7
mir-17	microRNA	5.30E-04	2.33E-02	mir-17	AR,ESR1,FAS,NCOA3	1	4
NKX2-1	transcription regulator	5.30E-04	2.53E-02	MMP2,NKX2-1	CSF2RB,MMP2,PPP2R1A,TJP1	2	4

SIM2	transcription regulator	5.30E-04	2.13E-02	MMP2,SIM2	CSF2RB,MMP2,PPP2R1A,TJP1	2	4
ESRRA	transcription regulator	5.66E-04	2.51E-02	ESRRA	HK2,HMOX1,PNMT,PPARG,RARA	1	5
miR-615-3p (miRNAs w/seed CCGAGCC)	mature microRNA	5.91E-04	5.20E-03	miR-615-3p (miRNAs w/seed CCGAGCC)	AR,PPARG	1	2
PRKAA1	kinase	8.21E-04	1.88E-02	PRKAA1	EPHA3,FN1,IGF1R,MAGEA4,TJP1	1	5
PAX3-FOXO1	fusion gene/product	8.85E-04	1.48E-02	PAX3-FOXO1	CLCN5,HMOX1,IGF1R,IMPA2,PPP2R1A,XRCC6	1	6
SREBF2	transcription regulator	9.05E-04	8.80E-03	SREBF2	FABP7,GTF2I,PPARG	1	3
ETV5	transcription regulator	9.09E-04	3.82E-02	ETV5,MMP2	CSF2RB,FN1,PPP2R1A,TJP1	2	4
CSF3	cytokine	1.12E-03	2.10E-02	CSF3	PPARG,PRKCB,RARA	1	3
PRKCB	kinase	1.17E-03	3.51E-02	PRKCB	FN1,MMP2	1	2
mir-192	microRNA	1.17E-03	1.26E-02	mir-192	IGF1R,ZEB2	1	2
DYSF	other	1.17E-03	2.63E-02	DYSF	FN1,ITGB3	1	2
mir-503	microRNA	1.17E-03	2.15E-02	mir-503	IGF1R,SMURF2	1	2
MOAP1	other	1.17E-03	9.80E-03	MOAP1	G6PD,HMOX1	1	2
SENP2	peptidase	1.93E-03	1.80E-02	SENP2	HK2,PGK1	1	2
KIF26A	other	1.93E-03	1.06E-02	KIF26A	RAC1,VAV3	1	2
c-Src	group	1.93E-03	1.64E-02	c-Src	CYP7A1,HMOX1	1	2
CDK9	kinase	1.95E-03	3.15E-02	CDK9	G6PD,PDK4,PPARG	1	3
MMP2	peptidase	1.95E-03	1.37E-02	MMP2	CSF2RB,PPP2R1A,TJP1	1	3
EPO	cytokine	2.29E-03	3.28E-02	EPO	MMP2,PRKCB,TJP1	1	3
mir-24	microRNA	2.87E-03	2.68E-02	mir-24	IFNG,PSAP	1	2
Mmp	group	3.99E-03	2.55E-02	Mmp	FAS,TJP1	1	2
NR1D1	ligand-dependent	3.99E-03	2.12E-02	NR1D1	MDH1,TLR4	1	2

	nuclear receptor						
BANCR	other	3.99E-03	2.65E-02	BANCR	MMP1,MMP2	1	2
RUNX1	transcription regulator	5.71E-03	4.75E-02	RUNX1	ALOX12,EP300,ITGB3	1	3
TRIM37	enzyme	5.71E-03	3.99E-02	TRIM37	CUL1,PEX5,SFN	1	3
GATA3	transcription regulator	1.01E-02	3.94E-02	GATA3	ECHS1,TJP1	1	2
EGLN	group	1.12E-02	4.19E-02	EGLN	HK2,PDK3,PYGL,SH2B2,ZNF292	1	5
LY96	transmembrane receptor	1.41E-02	3.06E-02	LY96	TLR4	1	1
HLTF	transcription regulator	1.41E-02	3.47E-02	HLTF	HBB	1	1
PKNOX1	transcription regulator	1.41E-02	3.08E-02	PKNOX1	FABP7	1	1
PROM1	other	1.41E-02	2.70E-02	PROM1	DSG2	1	1
SMG6	enzyme	1.41E-02	3.47E-02	SMG6	HBB	1	1
PSMC3IP	transcription regulator	1.41E-02	2.87E-02	PSMC3IP	CYP17A1	1	1
S100A3	transporter	1.41E-02	2.86E-02	S100A3	RARA	1	1
Dgk	group	1.41E-02	2.87E-02	Dgk	CYP17A1	1	1
ALDH1A3	enzyme	1.41E-02	2.44E-02	ALDH1A3	RRM1	1	1
BLVRA	enzyme	1.41E-02	2.22E-02	BLVRA	CERT1	1	1
CTD_2245E153	other	1.41E-02	2.51E-02	CTD_2245E153	PC	1	1
GFER	enzyme	1.41E-02	2.61E-02	GFER	NR1I3	1	1
PABIR1	other	1.41E-02	2.57E-02	PABIR1	WEE1	1	1
NUP98	transporter	2.81E-02	2.73E-02	NUP98	YWHAH	1	1
COMMD1	transporter	2.81E-02	4.97E-02	COMMD1	ATP7B	1	1
mir-346	microRNA	2.81E-02	4.92E-02	mir-346	BTK	1	1
CDC14A	phosphatase	2.81E-02	2.57E-02	CDC14A	WEE1	1	1
RAMAC	other	2.81E-02	2.14E-02	RAMAC	RUVBL1	1	1

MAP3K5	kinase	4.18E-02	2.39E-02	MAP3K5	MSH2	1	1

Supplementary Table S6: Toxicity analysis by IPA for solasonine. The toxicity analysis by IPA was performed to predict the toxicity of solasonine, by importing the dataset of potential targets of solasonine. The potential toxicity list was obtained and sorted by decreasing of -log(p-value), p-value ≤0.05.

Ingenuity Toxicity Lists	-log(p-value)	Ratio	Molecules
Hepatic Fibrosis	7.80	0.06	ALB,ATP7B,B2M,CYP2E1,CYP7A1,ESR1,F2,FN1,GRHPR,IFNG,IMPDH1,MMP1,MMP2,NMT1,NOTCH1,PAH,PPARG,PTPRE,SERPINA5,THBS2,TLR4
Liver Necrosis/Cell Death	7.00	0.06	AR,ARF6,ATP7B,CYP2E1,CYP7A1,ESR1,F3,FAS,GRK2,HBEGF,HK2,HMOX1,IFNG,IFNGR1,ITGB3,NR1I3,STK4,TLR4,XIAP
Renal Necrosis/Cell Death	7.00	0.04	ALB,AR,ATXN3,ERBB4,F2,F3,FAS,GRK2,HBEGF,HK2,HMOX1,IFNG,IGF1R,NDUFAB1,NF2,PEX5,PPARG,PRKCB,PTK2B,RAC1,SLK,STK4,TLR4,TNFAIP8L2,XIAP,YWHAQ,YY1
Fatty Acid Metabolism	7.00	0.11	ACADSB,ACADVL,ACAT2,ADH1C,ADH7,CYP2E1,ECHS1,ECI2,HADH,HSD17B10,HSD17B4
Cardiac Necrosis/Cell Death	6.48	0.06	AR,DPP4,DSG2,FAS,HK2,HMOX1,IFNG,MMP2,MYBPC3,PRKCB,PTK2B,RAC1,S100A6,STK4,THBS2,TLR4,VEGFB,XIAP
Cell Cycle: G2/M DNA Damage Checkpoint Regulation	6.37	0.16	BTRC,CUL1,EP300,SFN,SKP1,WEE1,YWHAH,YWHAQ
Cardiac Fibrosis	5.67	0.06	AR,DSG2,F3,FN1,HBEGF,HMOX1,IFNG,IGF1R,MYBPC3,PRKCB,STK4,THBS2,TLR4,UTRN,VAV3,VEGFB
Cardiac Hypertrophy	4.80	0.04	EP300,ESR1,FN1,HBB,HBEGF,HCK,ITGB3,MMP1,MMP2,MYBPC3,NOTCH1,PRKCB,RAC1,S100A6,TLR4,TTR,VAV3,VEGFB
Increases Liver Damage	4.38	0.09	CYP2E1,CYP7A1,F2,FAS,IFNG,IFNGR1,IGF1R,TLR4
Liver Proliferation	3.63	0.05	CSF2RB,F2,FAS,HBEGF,IFNG,IFNGR1,IGF1R,ITGB3,NR1I3,ODC1,PPARG,RARA
LPS/IL-1 Mediated Inhibition of RXR Function	3.59	0.05	CYP2E1,CYP2R1,CYP7A1,FABP3,FABP7,GSTT2/GSTT2B,HMGCS1,NR1I3,RARA,SULT1A1,TLR4
Increases Damage of Mitochondria	3.55	0.30	AR,FAS,IFNG
RAR Activation	3.47	0.05	ADH1C,ADH7,CSF2RB,EP300,MMP1,PRKCB,RAC1,RARA,RARG,RBP5
Xenobiotic Metabolism Signaling	3.45	0.04	ADH1C,ADH7,CAMK4,CYP2E1,EP300,GSTT2/GSTT2B,HMOX1,HSP90AB1,MAP3K3,NR1I3,PPP2R1A,PRKCB,SULT1A1
Increases Renal Damage	3.40	0.07	B2M,FCGR2A,GC,HCK,POU2F1,RAC1,TLR4
Increases Cardiac Proliferation	3.10	0.08	ERBB4,HBEGF,HMOX1,NAE1,NOTCH1,RARA
Increases Glomerular Injury	3.10	0.06	B2M,ESR1,FCGR2A,HBEGF,IFNG,IFNGR1,SLK,SMURF2
Aryl Hydrocarbon Receptor Signaling	2.98	0.05	EP300,ESR1,FAS,GSTT2/GSTT2B,HSP90AB1,NCOA3,RARA,RARG
LXR/RXR Activation	2.81	0.06	ALB,CYP7A1,ECHS1,GC,HADH,TLR4,TTR
Increases Liver Hepatitis	2.81	0.07	ESR1,FAS,IFNG,IFNGR1,TLR4,XIAP

Cell Cycle: G1/S Checkpoint Regulation	2.65	0.07	BTRC,CUL1,HDAC7,PA2G4,SKP1
Renal Safety Biomarker Panel (PSTC)	2.57	0.33	ALB,B2M
VDR/RXR Activation	2.39	0.06	EP300,IFNG,NCOA3,PRKCB,YY1
Increases Transmembrane Potential of Mitochondria and Mitochondrial Membrane	2.32	0.08	BCR,FAS,PPARG,RAC1
Increases Renal Proliferation	2.27	0.05	CAMK4,CHKA,CSTF2,ESR1,NDUFAB1,PPP2R1A,TLR4
FXR/RXR Activation	2.12	0.05	ALB,CYP7A1,GC,PPARG,RARA,TTR
Mechanism of Gene Regulation by Peroxisome Proliferators via PPARα	2.03	0.05	EP300,HSD17B4,HSP90AB1,PPARG,PRKCB
Increases Renal Nephritis	2.01	0.07	FCGR2A,HBEGF,IFNG,IFNGR1
Hepatic Stellate Cell Activation	1.92	0.09	CYP2E1,IFNG,TLR4
Cytochrome P450 Panel - Substrate is a Sterol (Human)	1.82	0.14	CYP17A1,CYP7A1
Cytochrome P450 Panel - Substrate is a Sterol (Mouse)	1.82	0.14	CYP17A1,CYP7A1
Cytochrome P450 Panel - Substrate is a Sterol (Rat)	1.82	0.14	CYP17A1,CYP7A1
Increases Cardiac Dysfunction	1.77	0.06	EP300,FAS,PRKCB,STK4
Pro-Apoptosis	1.71	0.07	DAPK2,DFFA,FAS
Cholesterol Biosynthesis	1.71	0.13	ACAT2,HMGCS1
NRF2-mediated Oxidative Stress Response	1.57	0.03	CYP2E1,CYP2R1,EP300,GSTT2/GSTT2B,HMOX1,HSP90AB1,PRKCB
TR/RXR Activation	1.55	0.05	CAMK4,CYP7A1,EP300,NCOA3
Genes associated with Chronic Allograft Nephropathy (Human)	1.48	0.10	FN1,MMP2
Increases Liver Steatosis	1.47	0.04	ATP7B,CYP2E1,HMOX1,NR1I3,PPARG
Increases Liver Hyperplasia/Hyperproliferation	1.43	0.04	FAS,IGF1R,NR1I3,ODC1,S100A6

Decreases Transmembrane Potential of Mitochondria and Mitochondrial Membrane	1.43	0.04	B2M,DFFA,FAS,IFNG,MUC1
Hepatic Cholestasis	1.39	0.03	CYP7A1,ESR1,IFNG,PRKCB,RARA,TLR4
Reversible Glomerulonephritis Biomarker Panel (Rat)	1.37	0.08	HBB,KLK1
Protection from Hypoxia-induced Renal Ischemic Injury (Rat)	1.27	0.25	HMOX1
CAR/RXR Activation	1.23	0.07	NR1I3,SULT1A1
Hypoxia-Inducible Factor Signaling	1.16	0.04	EP300,HSP90AB1,PRKCB
Decreases Depolarization of Mitochondria and Mitochondrial Membrane	1.13	0.06	CD74,FAS
Cytochrome P450 Panel - Substrate is a Vitamin (Human)	1.10	0.17	CYP2R1
Cytochrome P450 Panel - Substrate is a Vitamin (Mouse)	1.04	0.14	CYP2R1
Cytochrome P450 Panel - Substrate is a Vitamin (Rat)	1.04	0.14	CYP2R1
PPARα/RXRα Activation	0.99	0.03	ACVR2B,EP300,HSP90AB1,NCOA3,PRKCB
Negative Acute Phase Response Proteins	0.98	0.13	ALB
Primary Glomerulonephritis Biomarker Panel (Human)	0.85	0.09	HBEGF
Cytochrome P450 Panel - Substrate is a Xenobiotic (Mouse)	0.82	0.08	CYP2E1
TGF-β Signaling	0.81	0.03	ACVR2B,EP300,SMURF2
p53 Signaling	0.81	0.03	EP300,FAS,SFN
Cytochrome P450 Panel - Substrate is a Xenobiotic (Rat)	0.79	0.08	CYP2E1
Decreases Respiration of Mitochondria	0.79	0.08	IFNG
Acute Renal Failure Panel (Rat)	0.76	0.04	HMOX1,SFN

Swelling of Mitochondria	0.76	0.07	FAS
Recovery from Ischemic Acute Renal Failure (Rat)	0.76	0.07	KLK1
Long-term Renal Injury Anti-oxidative Response Panel (Rat)	0.73	0.07	HMOX1
Renal Glomerulus Panel (Human)	0.68	0.06	TJP1
PXR/RXR Activation	0.67	0.03	CYP7A1,NR1I3
Cytochrome P450 Panel - Substrate is a Xenobiotic (Human)	0.66	0.06	CYP2E1
Glutathione Depletion - Phase II Reactions	0.64	0.05	GSTT2/GSTT2B
Increases Bradycardia	0.62	0.05	RAC1
NF-κB Signaling	0.61	0.02	BTRC,EP300,IGF1R,MAP3K3,MAPK6,PRKCB,TLR4
Positive Acute Phase Response Proteins	0.47	0.03	HMOX1
Anti-Apoptosis	0.45	0.03	XIAP
Mitochondrial Dysfunction	0.41	0.02	HSD17B10,NDUFAB1,PDHA1
Oxidative Stress	0.27	0.02	CYP2E1

Supplementary Table S7: Potential therapeutic targets of solasonine for BC. By integrating the potential targets regulated by solasonine, and the genes associated with BC searched in databases of GeneCards, OMIM, TTD, PharmGKB, and DrugBank, a total of 191 genes overlapped were identified, which could be considered as the potential therapeutic targets of solasonine for BC.

**Num**	**Symbol**	**Gene Name**	**Source**				
			GeneCards	OMIM	TTD	PharmGKB	DrugBank
1	CSDE1	Cold shock domain-containing protein E1	√				
2	RGS6	Regulator of G-protein signaling 6	√				
3	POT1	Protection of telomeres protein 1	√				
4	F2	Prothrombin	√				
5	RARG	Retinoic acid receptor gamma	√				
6	NF2	Merlin	√				
7	CUL1	Cullin-1	√				
8	HK2	Hexokinase-2	√				
9	EP300	Histone acetyltransferase p300	√	√			
10	RRM1	Ribonucleoside-diphosphate reductase large subunit	√				√
11	XRCC6	ATP-dependent DNA helicase 2 subunit 1	√				
12	HSD17B4	Peroxisomal multifunctional enzyme type 2	√				
13	MMP2	72 kDa type IV collagenase	√				
14	ACADVL	Very long-chain specific acyl-CoA dehydrogenase, mitochondrial	√				
15	HBEGF	Proheparin-binding EGF-like growth factor	√				
16	HMGCS1	Hydroxymethylglutaryl-CoA synthase, cytoplasmic	√				
17	AR	Androgen receptor	√	√	√		
18	HMOX1	Heme oxygenase 1	√				
19	VAV3	Guanine nucleotide exchange factor VAV3	√				
20	ZEB2	Zinc finger E-box-binding homeobox 2	√				
21	NME3	Nucleoside diphosphate kinase 3	√				
22	BTK	Tyrosine-protein kinase BTK	√				
23	MUC1	Mucin-1	√				
24	MARK1	Serine/threonine-protein kinase MARK1	√				
25	VEGFB	Vascular endothelial growth factor B	√				
26	FABP3	Fatty acid-binding protein, heart	√				
27	KLK1	Kallikrein-1	√				
28	RND1	Rho-related GTP-binding protein Rho6	√				

29	HBB	Hemoglobin subunit beta	√	
30	MAP3K3	Mitogen-activated protein kinase kinase kinase 3	√	
31	DFFA	DNA fragmentation factor subunit alpha	√	
32	RBP5	Retinol-binding protein 5	√	
33	ESR1	Estrogen receptor	√	√
34	RAC1	Ras-related C3 botulinum toxin substrate 1	√	
35	PTPRF	Receptor-type tyrosine-protein phosphatase F	√	
36	HCK	Tyrosine-protein kinase HCK	√	
37	FCGR2A	Low affinity immunoglobulin gamma Fc region receptor II-a	√	
38	EPHA3	Ephrin type-A receptor 3	√	
39	PTK2B	Protein tyrosine kinase 2 beta	√	
40	TRIM21	52 kDa Ro protein	√	
41	DTYMK	Thymidylate kinase	√	
42	PPP2R1A	Serine/threonine-protein phosphatase 2A 65 kDa regulatory subunit A alpha isoform	√	
43	MTAP	S-methyl-5-thioadenosine phosphorylase	√	
44	IGF1R	Insulin-like growth factor 1 receptor	√	
45	FLNB	Filamin-B	√	
46	HDAC7	Histone deacetylase 7	√	
47	ALOX12	Arachidonate 12-lipoxygenase, 12S-type	√	
48	WWP1	NEDD4-like E3 ubiquitin-protein ligase WWP1	√	
49	HSP90AB1	Heat shock protein HSP 90-beta	√	
50	KIF11	Kinesin-like protein KIF11	√	
51	GC	Vitamin D-binding protein	√	
52	MATK	Megakaryocyte-associated tyrosine-protein kinase	√	
53	DCXR	L-xylulose reductase	√	
54	F3	Tissue factor	√	
55	ACVR2B	Activin receptor type-2B	√	
56	CLK1	Dual specificity protein kinase CLK1	√	
57	SENP2	Sentrin-specific protease 2	√	
58	MSN	Moesin	√	
59	SF3B6	Pre-mRNA branch site protein p14	√	
60	RAB26	Ras-related protein Rab-26	√	
61	BMP1	Bone morphogenetic protein 1	√	

62	PAPSS1	Bifunctional 3-phosphoadenosine 5-phosphosulfate synthetase 1	√		
63	PYGL	Glycogen phosphorylase, liver form	√		
64	MSH2	DNA mismatch repair protein Msh2	√	√	√
65	POU2F1	POU domain, class 2, transcription factor 1	√		
66	GTF2I	General transcription factor II-I	√		
67	SKP1	S-phase kinase-associated protein 1	√		
68	DHPS	Deoxyhypusine synthase	√		
69	NCOA3	Nuclear receptor coactivator 3	√	√	
70	TTR	Transthyretin	√		
71	CSNK1G2	Casein kinase I isoform gamma-2	√		
72	PDK2	[Pyruvate dehydrogenase [lipoamide]] kinase isozyme 2, mitochondrial	√		
73	ADH1C	Alcohol dehydrogenase 1C	√		
74	ITPK1	Inositol-tetrakisphosphate 1-kinase	√		
75	BPI	Bactericidal permeability-increasing protein	√		
76	HPD	4-hydroxyphenylpyruvate dioxygenase	√		
77	PAH	Phenylalanine-4-hydroxylase	√		
78	B2M	Beta-2-microglobulin	√		
79	SULT1A1	Sulfotransferase 1A1	√		
80	MAGEA4	Melanoma-associated antigen 4	√		√
81	NSUN5	Putative methyltransferase NSUN5	√		
82	LRBA	Lipopolysaccharide-responsive and beige-like anchor protein	√		
83	SART3	Squamous cell carcinoma antigen recognized by T-cells 3	√		√
84	BCR	Breakpoint cluster region protein	√		
85	PSAP	Proactivator polypeptide	√		
86	YWHAQ	14-3-3 protein theta	√		
87	SMG5	Protein SMG5	√		
88	WEE1	Wee1-like protein kinase	√		
89	TLR4	Toll-like receptor 4	√		
90	CYP7A1	Cytochrome P450 7A1	√		
91	IFNGR1	Interferon-gamma receptor alpha chain	√		
92	FAS	Tumor necrosis factor receptor superfamily member 6	√	√	
93	PEX5	Peroxisomal targeting signal 1 receptor	√		
94	PA2G4	Proliferation-associated protein 2G4	√		

95	EXOSC9	Exosome complex exonuclease RRP45	√		
96	ACACB	Acetyl-CoA carboxylase 2		√	
97	ATXN3	Ataxin-3	√		
98	HNRNPA2B1	Heterogeneous nuclear ribonucleoproteins A2/B1	√		
99	ERBB4	Receptor tyrosine-protein kinase erbB-4	√		
100	ITGB3	Integrin beta-3	√		
101	GAD2	Glutamate decarboxylase 2	√		
102	PCYT2	Ethanolamine-phosphate cytidylyltransferase	√		
103	FAP	Seprase	√		
104	PARN	Poly(A)-specific ribonuclease PARN	√		
105	RPA3	Replication protein A 14 kDa subunit	√		
106	ANXA6	Annexin A6	√		
107	MAGI2	Membrane-associated guanylate kinase, WW and PDZ domain-containing protein 2	√		
108	HLA-DRB1	HLA class II histocompatibility antigen, DRB1-1 beta chain	√		
109	NFATC1	Nuclear factor of activated T-cells, cytoplasmic 1	√		
110	ALK	ALK tyrosine kinase receptor	√		
111	CYP2E1	Cytochrome P450 2E1	√		
112	PPARG	Peroxisome proliferator-activated receptor gamma	√		
113	ATP7B	Copper-transporting ATPase 2	√		√
114	PGK1	Phosphoglycerate kinase 1	√		
115	MMP1	Interstitial collagenase	√		
116	DAPK2	Death-associated protein kinase 2	√		
117	S100A6	Protein S100-A6	√		
118	NOTCH1	Neurogenic locus notch homolog protein 1	√		
119	SERPINA5	Plasma serine protease inhibitor	√		
120	NAE1	NEDD8-activating enzyme E1 regulatory subunit	√		
121	ZIC3	Zinc finger protein ZIC 3	√		
122	ODC1	Ornithine decarboxylase	√		
123	RARA	Retinoic acid receptor alpha	√		
124	PNMT	Phenylethanolamine N-methyltransferase	√		
125	TJP1	Tight junction protein ZO-1	√		
126	AOC3	Membrane primary amine oxidase	√		
127	CSTF2	Cleavage stimulation factor 64 kDa subunit	√		

128	YY1	Transcriptional repressor protein YY1	√			
129	IFNG	Interferon gamma	√			
130	ANG	Angiogenin	√			
131	ACY1	Aminoacylase-1	√			
132	SNX1	Sorting nexin-1	√			
133	CTCF	Transcriptional repressor CTCF	√			
134	TAF13	Transcription initiation factor TFIID subunit 13	√			
135	GGPS1	Geranylgeranyl pyrophosphate synthetase	√			
136	BTRC	F-box/WD repeat-containing protein 1A	√			
137	TNFAIP8L2	Tumor necrosis factor, alpha-induced protein 8-like protein 2	√	√		
138	NBEA	Neurobeachin	√			
139	MYBPC1	Myosin-binding protein C, slow-type	√			
140	STK4	Serine/threonine-protein kinase 4	√			
141	ECI2	Peroxisomal 3,2-trans-enoyl-CoA isomerase	√		√	
142	DPP4	Dipeptidyl peptidase 4	√			
143	ARHGEF1	Rho guanine nucleotide exchange factor 1	√			
144	APEX1	DNA-(apurinic or apyrimidinic site) lyase	√			
145	B3GAT3	Galactosylgalactosylxylosylprotein 3-beta-glucuronosyltransferase 3	√			
146	ARF6	ADP-ribosylation factor 6	√			
147	XIAP	Baculoviral IAP repeat-containing protein 4	√			
148	ECHS1	Enoyl-CoA hydratase, mitochondrial	√			
149	ALB	Serum albumin	√			√
150	PRKCB	Protein kinase C beta type	√			
151	FHIT	Bis(5-adenosyl)-triphosphatase	√			
152	GART	Trifunctional purine biosynthetic protein adenosine-3	√			
153	C1R	Complement C1r subcomponent	√			
154	YWHAH	14-3-3 protein eta	√			
155	OXSR1	Serine/threonine-protein kinase OSR1	√			
156	RTN4IP1	Reticulon-4-interacting protein 1, mitochondrial	√			
157	HMGB3	High mobility group protein B3	√			
158	OAT	Ornithine aminotransferase, mitochondrial	√			
159	SFN	14-3-3 protein sigma	√			
160	STARD13	StAR-related lipid transfer protein 13	√			

161	G6PD	Glucose-6-phosphate 1-dehydrogenase	√		
162	DSG2	Desmoglein-2	√		
163	THBS2	Thrombospondin-2	√		
164	DCTD	Deoxycytidylate deaminase	√		
165	PDK4	[Pyruvate dehydrogenase [lipoamide]] kinase isozyme 4, mitochondrial	√		
166	FN1	Fibronectin	√		
167	CHKA	Choline kinase alpha	√		
168	SMTN	Smoothelin	√		
169	VPS26A	Vacuolar protein sorting-associated protein 26A	√		
170	SEMA4D	Semaphorin-4D	√		
171	CLCN5	Chloride channel protein 5	√		
172	GRHPR	Glyoxylate reductase/hydroxypyruvate reductase	√		
173	NMT1	Glycylpeptide N-tetradecanoyltransferase 1	√		
174	NDC80	Kinetochore protein NDC80 homolog	√		
175	PARP3	Poly [ADP-ribose] polymerase 3	√		
176	RBM39	RNA-binding protein 39	√		
177	CD74	HLA class II histocompatibility antigen gamma chain	√		
178	CYP17A1	Steroid 17-alpha-hydroxylase	√		
179	SLC5A1	Sodium/glucose cotransporter 1	√		
180	DHCR24	Delta(24)-sterol reductase	√		
181	AMY2A	Pancreatic alpha-amylase	√		
182	IL2	Interleukin-2	√		
183	NPC1L1	NPC1-like intracellular cholesterol transporter 1	√		
184	ABCC4	Multidrug resistance-associated protein 4	√		√
185	SERPINA6	Corticosteroid-binding globulin	√		
186	GLI1	Zinc finger protein GLI1	√		
187	POLA1	DNA polymerase alpha catalytic subunit	√		
188	SRD5A2	3-oxo-5-alpha-steroid 4-dehydrogenase 2	√		
189	NR1H3	Oxysterols receptor LXR-alpha	√		
190	SHH	Sonic hedgehog protein	√		
191	SHBG	Sex hormone-binding globulin	√		

Note: BC, Bladder Cancer; OMIM, Online Mendelian Inheritance in Man database; TTD, Therapeutic Target Database; PharmGKB, Pharmacogenomics Knowledgebase Database.

Supplementary Table S8: KEGG pathway and GO terms analyses for genes of solasonine targeting BC. Through KEGG enrichment of identified genes related to solasonine targeting BC, 3 remarkably enriched pathways were obtained (adj.P-value ≤0.05); through GO terms analysis, 692, 16 and 48 remarkably enriched biological process (BP), cellular component (CC) and molecular function (MF) terms were obtained respectively (adj.P-value ≤0.05).

Group	ID	Description	GeneRatio	BgRatio	p-value	p.adjust	q-value	geneID
KEGG	hsa05205	Proteoglycans in cancer	16/155	205/8093	1.71E-06	4.81E-04	4.04E-04	MMP2/HBEGF/VAV3/ESR1/RAC1/IGF1R/FLNB/MSN/TLR4/FAS/ERBB4/ITGB3/ARHGEF1/PRKCB/FN1/SHH
KEGG	hsa04066	HIF-1 signaling pathway	9/155	109/8093	2.29E-04	2.66E-02	2.23E-02	HK2/EP300/HMOX1/IGF1R/TLR4/IFNGR1/PGK1/IFNG/PRKCB
KEGG	hsa05135	Yersinia infection	10/155	137/8093	2.84E-04	2.66E-02	2.23E-02	VAV3/RAC1/FCGR2A/PTK2B/TLR4/NFATC1/ARHGEF1/ARF6/FN1/IL2
BP	GO:0008202	steroid metabolic process	19/189	329/18862	9.16E-10	3.43E-06	2.35E-06	HSD17B4/ACADVL/HMGCS1/ESR1/GC/SULT1A1/CYP7A1/ACACB/CYP2E1/IFNG/GGPS1/YWHAH/G6PD/CYP17A1/DHCR24/NPC1L1/SERPINA6/SRD5A2/SHH
BP	GO:0035265	organ growth	13/189	168/18862	1.28E-08	1.60E-05	1.10E-05	RARG/AR/ESR1/PSAP/ACACB/ERBB4/ANXA6/NOTCH1/YY1/STK4/G6PD/GLI1/SHH
BP	GO:0048608	reproductive structure development	18/189	407/18862	2.72E-08	1.89E-05	1.29E-05	CSDE1/RARG/RRM1/HSD17B4/HMGCS1/AR/ESR1/HSP90AB1/SENP2/MSH2/PSAP/PPARG/NOTCH1/SERPINA5/ANG/STK4/GLI1/SRD5A2/SHH
BP	GO:0061458	reproductive system development	19/189	408/18862	3.06E-08	1.89E-05	1.29E-05	CSDE1/RARG/RRM1/HSD17B4/HMGCS1/AR/ESR1/HSP90AB1/SENP2/MSH2/PSAP/PPARG/NOTCH1/SERPINA5/ANG/STK4/GLI1/SRD5A2/SHH
BP	GO:0018108	peptidyl-tyrosine phosphorylation	18/189	369/18862	3.56E-08	1.89E-05	1.29E-05	NF2/HBEGF/BTK/HCK/EPHA3/PTK2B/PPP2R1A/IGF1R/MATK/CLK1/WEE1/ERBB4/ITGB3/ALK/IFNG/SEMA4D/CD74/IL2
BP	GO:1901617	organic hydroxy compound biosynthetic process	15/189	251/18862	3.71E-08	1.89E-05	1.29E-05	HSD17B4/HMGCS1/ZEB2/PTK2B/ALOX12/PAH/CYP7A1/ACACB/PNMT/IFNG/GGPS1/G6PD/DHCR24/NPC1L1/SRD5A2
BP	GO:0018212	peptidyl-tyrosine modification	18/189	372/18862	4.03E-08	1.89E-05	1.29E-05	NF2/HBEGF/BTK/HCK/EPHA3/PTK2B/PPP2R1A/IGF1R/MATK/CLK1/WEE1/ERBB4/ITGB3/ALK/IFNG/SEMA4D/CD74/IL2
BP	GO:0050866	negative regulation of cell activation	13/189	200/18862	1.17E-07	4.87E-05	3.34E-05	F2/HMOX1/BTK/ALOX12/BPI/HLA-DRB1/TNFAIP8L2/HMGB3/PARP3/CD74/IL2/NR1H3/SHH
BP	GO:0030850	prostate gland development	7/189	41/18862	1.53E-07	5.73E-05	3.93E-05	RARG/AR/ESR1/PSAP/NOTCH1/GLI1/SHH
BP	GO:0060249	anatomical structure homeostasis	19/189	466/18862	2.43E-07	8.28E-05	5.68E-05	POT1/XRCC6/RAC1/PTK2B/HSP90AB1/B2M/SMG5/TLR4/HNRNPA2B1/ITGB3/PARN/RPA3/NOTCH1/TJP1/APEX1/ALB/PDK4/PARP3/POLA1
BP	GO:0060333	interferon-gamma-mediated signaling pathway	9/189	91/18862	3.25E-07	1.01E-04	6.96E-05	HCK/TRIM21/HSP90AB1/B2M/IFNGR1/HLA-DRB1/PPARG/IFNG/NR1H3

BP	GO:0033002	muscle cell proliferation	13/189	222/18862	3.91E-07	1.13E-04	7.72E-05	XRCC6/MMP2/HBEGF/HMOX1/ALOX12/ERBB4/PPARG/NOTCH1/IFNG/ANG/ABCC4/GLI1/SHH
BP	GO:0046777	protein autophosphorylation	13/189	226/18862	4.79E-07	1.28E-04	8.79E-05	BTK/MAP3K3/HCK/PTK2B/IGF1R/CLK1/CSNK1G2/BCR/ERBB4/ALK/DAPK2/STK4/OXSR1
BP	GO:0019217	regulation of fatty acid metabolic process	9/189	98/18862	6.15E-07	1.45E-04	9.94E-05	ACADVL/FABP3/PDK2/CYP7A1/ACACB/PPARG/PDK4/CD74/NR1H3
BP	GO:0019216	regulation of lipid metabolic process	17/189	402/18862	6.53E-07	1.45E-04	9.94E-05	F2/ACADVL/HMGCS1/VAV3/FABP3/PTK2B/PDK2/PSAP/CYP7A1/ACACB/ALK/PPARG/IFNG/GGPS1/PDK4/CD74/NR1H3
BP	GO:0010631	epithelial cell migration	16/189	357/18862	6.58E-07	1.45E-04	9.94E-05	HBEGF/HMOX1/ZEB2/MAP3K3/RAC1/PTK2B/HDAC7/ALOX12/ITGB3/FAP/PPARG/NOTCH1/IFNG/DPP4/ARF6/STARD13
BP	GO:0045088	regulation of innate immune response	15/189	315/18862	6.99E-07	1.45E-04	9.94E-05	CUL1/EP300/XRCC6/MUC1/HCK/TRIM21/HSP90AB1/SKP1/TLR4/IFNGR1/PPARG/IFNG/BTRC/XIAP/NR1H3
BP	GO:0090132	epithelium migration	16/189	360/18862	7.35E-07	1.45E-04	9.94E-05	HBEGF/HMOX1/ZEB2/MAP3K3/RAC1/PTK2B/HDAC7/ALOX12/ITGB3/FAP/PPARG/NOTCH1/IFNG/DPP4/ARF6/STARD13
BP	GO:0090130	tissue migration	16/189	365/18862	8.82E-07	1.65E-04	1.13E-04	HBEGF/HMOX1/ZEB2/MAP3K3/RAC1/PTK2B/HDAC7/ALOX12/ITGB3/FAP/PPARG/NOTCH1/IFNG/DPP4/ARF6/STARD13
BP	GO:0019395	fatty acid oxidation	9/189	105/18862	1.11E-06	1.97E-04	1.35E-04	HSD17B4/ACADVL/FABP3/ALOX12/ACACB/PPARG/ECI2/ECHS1/PDK4
BP	GO:0034440	lipid oxidation	9/189	110/18862	1.64E-06	2.79E-04	1.91E-04	HSD17B4/ACADVL/FABP3/ALOX12/ACACB/PPARG/ECI2/ECHS1/PDK4
BP	GO:0042180	cellular ketone metabolic process	13/189	254/18862	1.78E-06	2.90E-04	1.99E-04	ACADVL/FABP3/PDK2/CYP7A1/ACACB/PPARG/ODC1/PDK4/NMT1/CD74/CYP17A1/SRD5A2/NR1H3
BP	GO:0060736	prostate gland growth	4/189	10/18862	1.96E-06	3.05E-04	2.09E-04	AR/ESR1/PSAP/SHH
BP	GO:0002695	negative regulation of leukocyte activation	11/189	182/18862	2.31E-06	3.46E-04	2.37E-04	HMOX1/BTK/BPI/HLA-DRB1/TNFAIP8L2/HMGB3/PARP3/CD74/IL2/NR1H3/SHH
BP	GO:0002683	negative regulation of immune system process	16/189	403/18862	3.19E-06	4.59E-04	3.15E-04	HMOX1/BTK/TRIM21/BPI/TLR4/HLA-DRB1/PPARG/RARA/TNFAIP8L2/DPP4/HMGB3/PARP3/CD74/IL2/NR1H3/SHH
BP	GO:0006694	steroid biosynthetic process	11/189	190/18862	3.50E-06	4.72E-04	3.24E-04	HSD17B4/HMGCS1/CYP7A1/ACACB/IFNG/GGPS1/G6PD/CYP17A1/DHCR24/NPC1L1/SRD5A2

BP	GO:0042493	response to drug	15/189	359/18862	3.53E-06	4.72E-04	3.24E-04	HMGCS1/HMOX1/VAV3/VEGFB/FABP3/PTK2B/HSP90AB1/B2M/ACACB/GAD2/CYP2E1/PPARG/APEX1/NPC1L1/ABCC4
BP	GO:0060330	regulation of response to interferon-gamma	5/189	25/18862	4.33E-06	5.10E-04	3.50E-04	HSP90AB1/IFNGR1/PPARG/IFNG/NR1H3
BP	GO:0060334	regulation of interferon-gamma-mediated signaling pathway	5/189	25/18862	4.33E-06	5.10E-04	3.50E-04	HSP90AB1/IFNGR1/PPARG/IFNG/NR1H3
BP	GO:0060740	prostate gland epithelium morphogenesis	5/189	25/18862	4.33E-06	5.10E-04	3.50E-04	RARG/AR/ESR1/NOTCH1/SHH
BP	GO:0048732	gland development	16/189	413/18862	4.36E-06	5.10E-04	3.50E-04	RARG/HK2/HMGCS1/AR/HMOX1/ESR1/MSN/PSAP/ERBB4/NOTCH1/SERPINA5/ZIC3/BTRC/ARF6/GLI1/SHH
BP	GO:0034341	response to interferon-gamma	11/189	197/18862	4.96E-06	5.63E-04	3.86E-04	HCK/TRIM21/HSP90AB1/B2M/TLR4/IFNGR1/HLA-DRB1/PPARG/IFNG/CD74/NR1H3
BP	GO:0000723	telomere maintenance	10/189	161/18862	5.27E-06	5.77E-04	3.96E-04	POT1/XRCC6/HSP90AB1/SMG5/HNRNPA2B1/PARN/RPA3/APEX1/PARP3/POLA1
BP	GO:0002831	regulation of response to biotic stimulus	16/189	420/18862	5.39E-06	5.77E-04	3.96E-04	CUL1/EP300/XRCC6/MUC1/HCK/TRIM21/HSP90AB1/SKP1/TLR4/IFNGR1/HLA-DRB1/PPARG/IFNG/BTRC/XIAP/NR1H3
BP	GO:0060512	prostate gland morphogenesis	5/189	27/18862	6.47E-06	6.73E-04	4.61E-04	RARG/AR/ESR1/NOTCH1/SHH
BP	GO:0050673	epithelial cell proliferation	16/189	428/18862	6.84E-06	6.85E-04	4.70E-04	AR/HMOX1/VEGFB/ESR1/F3/B2M/TLR4/ITGB3/FAP/PPARG/NOTCH1/ANG/BTRC/SFN/GLI1/SHH
BP	GO:0043627	response to estrogen	7/189	71/18862	7.02E-06	6.85E-04	4.70E-04	EP300/AR/HMOX1/ESR1/DTYMK/PPARG/RARA
BP	GO:0051972	regulation of telomerase activity	6/189	47/18862	7.13E-06	6.85E-04	4.70E-04	POT1/HSP90AB1/HNRNPA2B1/PARN/PPARG/PARP3
BP	GO:0006695	cholesterol biosynthetic process	7/189	72/18862	7.71E-06	7.05E-04	4.83E-04	HMGCS1/CYP7A1/ACACB/GGPS1/G6PD/DHCR24/NPC1L1
BP	GO:1902653	secondary alcohol biosynthetic process	7/189	72/18862	7.71E-06	7.05E-04	4.83E-04	HMGCS1/CYP7A1/ACACB/GGPS1/G6PD/DHCR24/NPC1L1
BP	GO:0045621	positive regulation of lymphocyte differentiation	8/189	101/18862	8.00E-06	7.13E-04	4.89E-04	XRCC6/BTK/HLA-DRB1/RARA/IFNG/CD74/IL2/SHH
BP	GO:0010632	regulation of epithelial cell migration	13/189	293/18862	8.49E-06	7.39E-04	5.07E-04	HBEGF/HMOX1/MAP3K3/RAC1/PTK2B/HDAC7/ALOX12/ITGB3/PPARG/NOTCH1/IFNG/ARF6/STARD13

BP	GO:1903829	positive regulation of cellular protein localization	13/189	295/18862	9.13E-06	7.77E-04	5.33E-04	F2/EPHA3/HSP90AB1/MSN/YWHAQ/ERBB4/IFNG/ARF6/YWHAH/SFN/NMT1/NDC80/SHH
BP	GO:0007596	blood coagulation	14/189	342/18862	9.51E-06	7.91E-04	5.42E-04	F2/VAV3/HBB/RAC1/ALOX12/F3/ITPK1/TLR4/ITGB3/FAP/SERPINA5/PRKCB/FN1/SHH
BP	GO:0045927	positive regulation of growth	12/189	253/18862	9.75E-06	7.94E-04	5.44E-04	F2/HBEGF/PTK2B/EXOSC9/ACACB/ERBB4/NOTCH1/SFN/FN1/SEMA4D/IL2/GLI1
BP	GO:0032200	telomere organization	10/189	174/18862	1.05E-05	8.22E-04	5.64E-04	POT1/XRCC6/HSP90AB1/SMG5/HNRNPA2B1/PARN/RPA3/APEX1/PARP3/POLA1
BP	GO:0007599	hemostasis	14/189	346/18862	1.08E-05	8.22E-04	5.64E-04	F2/VAV3/HBB/RAC1/ALOX12/F3/ITPK1/TLR4/ITGB3/FAP/SERPINA5/PRKCB/FN1/SHH
BP	GO:0010634	positive regulation of epithelial cell migration	10/189	175/18862	1.10E-05	8.22E-04	5.64E-04	HBEGF/HMOX1/MAP3K3/RAC1/PTK2B/HDAC7/ALOX12/ITGB3/IFNG/ARF6
BP	GO:0008209	androgen metabolic process	5/189	30/18862	1.11E-05	8.22E-04	5.64E-04	HSD17B4/ESR1/CYP17A1/SRD5A2/SHH
BP	GO:0050817	coagulation	14/189	347/18862	1.12E-05	8.22E-04	5.64E-04	F2/VAV3/HBB/RAC1/ALOX12/F3/ITPK1/TLR4/ITGB3/FAP/SERPINA5/PRKCB/FN1/SHH
BP	GO:0071346	cellular response to interferon-gamma	10/189	177/18862	1.22E-05	8.66E-04	5.94E-04	HCK/TRIM21/HSP90AB1/B2M/TLR4/IFNGR1/HLA-DRB1/PPARG/IFNG/NR1H3
BP	GO:2000278	regulation of DNA biosynthetic process	8/189	107/18862	1.23E-05	8.66E-04	5.94E-04	POT1/PTK2B/HSP90AB1/SMG5/HNRNPA2B1/PARN/PPARG/PARP3
BP	GO:0016126	sterol biosynthetic process	7/189	78/18862	1.32E-05	9.12E-04	6.25E-04	HMGCS1/CYP7A1/ACACB/GGPS1/G6PD/DHCR24/NPC1L1
BP	GO:0030098	lymphocyte differentiation	14/189	358/18862	1.59E-05	1.07E-03	7.36E-04	EP300/XRCC6/BTK/PTK2B/MSH2/B2M/HLA-DRB1/RARA/YY1/IFNG/HMGB3/CD74/IL2/SHH
BP	GO:0022612	gland morphogenesis	8/189	111/18862	1.60E-05	1.07E-03	7.36E-04	RARG/AR/ESR1/MSN/NOTCH1/BTRC/GLI1/SHH
BP	GO:0051222	positive regulation of protein transport	13/189	312/18862	1.66E-05	1.08E-03	7.40E-04	RAC1/HSP90AB1/YWHAQ/TLR4/HLA-DRB1/IFNG/ANG/B3GAT3/ARF6/YWHAH/SFN/NMT1/SHH
BP	GO:0072521	purine-containing compound metabolic process	16/189	460/18862	1.67E-05	1.08E-03	7.40E-04	HK2/EP300/HSD17B4/HMGCS1/NME3/MTAP/PAPSS1/TTR/PDK2/SULT1A1/ACACB/PGK1/IFNG/FHIT/GART/PDK4
BP	GO:1903131	mononuclear cell differentiation	15/189	411/18862	1.77E-05	1.11E-03	7.62E-04	EP300/XRCC6/BTK/PTK2B/MSH2/B2M/HLA-DRB1/PPARG/RARA/YY1/IFNG/HMGB3/CD74/IL2/SHH

BP	GO:0010565	regulation of cellular ketone metabolic process	10/189	185/18862	1.79E-05	1.11E-03	7.62E-04	ACADVL/FABP3/PDK2/CYP7A1/ACACB/PPARG/ODC1/PDK4/CD74/NR1H3
BP	GO:0046320	regulation of fatty acid oxidation	5/189	33/18862	1.81E-05	1.11E-03	7.62E-04	ACADVL/FABP3/ACACB/PPARG/PDK4
BP	GO:1903706	regulation of hemopoiesis	15/189	415/18862	1.98E-05	1.20E-03	8.22E-04	RARG/EP300/XRCC6/BTK/PTK2B/B2M/TLR4/HLA-DRB1/RARA/IFNG/PRKCB/HMGB3/CD74/IL2/SHH
BP	GO:0045834	positive regulation of lipid metabolic process	9/189	150/18862	2.07E-05	1.23E-03	8.45E-04	F2/VAV3/FABP3/PTK2B/CYP7A1/PPARG/IFNG/CD74/NR1H3
BP	GO:0046661	male sex differentiation	9/189	153/18862	2.43E-05	1.42E-03	9.73E-04	CSDE1/RRM1/HSD17B4/HMGCS1/AR/ESR1/MSH2/SRD5A2/SHH
BP	GO:0043542	endothelial cell migration	12/189	278/18862	2.49E-05	1.42E-03	9.73E-04	HMOX1/MAP3K3/RAC1/PTK2B/HDAC7/ALOX12/ITGB3/FAP/PPARG/NOTCH1/DPP4/STARD13
BP	GO:0006066	alcohol metabolic process	14/189	373/18862	2.50E-05	1.42E-03	9.73E-04	ACADVL/HMGCS1/PTK2B/TTR/ADH1C/ITPK1/SULT1A1/CYP7A1/ACACB/GGPS1/G6PD/CHKA/DHCR24/NPC1L1
BP	GO:0071897	DNA biosynthetic process	10/189	194/18862	2.69E-05	1.49E-03	1.02E-03	POT1/PTK2B/HSP90AB1/SMG5/HNRNPA2B1/PARN/RPA3/PPARG/PARP3/POLA1
BP	GO:0045428	regulation of nitric oxide biosynthetic process	6/189	59/18862	2.71E-05	1.49E-03	1.02E-03	HBB/RAC1/PTK2B/HSP90AB1/TLR4/IFNG
BP	GO:1904951	positive regulation of establishment of protein localization	13/189	328/18862	2.79E-05	1.52E-03	1.04E-03	RAC1/HSP90AB1/YWHAQ/TLR4/HLA-DRB1/IFNG/ANG/B3GAT3/ARF6/YWHAH/SFN/NMT1/SHH
BP	GO:0080164	regulation of nitric oxide metabolic process	6/189	61/18862	3.28E-05	1.76E-03	1.20E-03	HBB/RAC1/PTK2B/HSP90AB1/TLR4/IFNG
BP	GO:0019693	ribose phosphate metabolic process	15/189	435/18862	3.42E-05	1.80E-03	1.23E-03	HK2/EP300/HSD17B4/HMGCS1/NME3/PAPSS1/PYGL/PDK2/SULT1A1/ACACB/PGK1/IFNG/GART/G6PD/PDK4
BP	GO:0048660	regulation of smooth muscle cell proliferation	9/189	160/18862	3.46E-05	1.80E-03	1.23E-03	XRCC6/MMP2/HBEGF/HMOX1/ALOX12/PPARG/IFNG/ANG/ABCC4
BP	GO:0062012	regulation of small molecule metabolic process	15/189	437/18862	3.60E-05	1.85E-03	1.27E-03	EP300/ACADVL/HMGCS1/FABP3/PTK2B/PDK2/CYP7A1/ACACB/PPARG/ODC1/IFNG/GGPS1/PDK4/CD74/NR1H3
BP	GO:0045429	positive regulation of nitric oxide biosynthetic process	5/189	38/18862	3.68E-05	1.86E-03	1.28E-03	HBB/PTK2B/HSP90AB1/TLR4/IFNG

BP	GO:0048659	smooth muscle cell proliferation	9/189	162/18862	3.81E-05	1.90E-03	1.30E-03	XRCC6/MMP2/HBEGF/HMOX1/ALOX12/PPARG/IFNG/ANG/ABCC4
BP	GO:0046890	regulation of lipid biosynthetic process	10/189	203/18862	3.96E-05	1.95E-03	1.34E-03	ACADVL/HMGCS1/FABP3/CYP7A1/ACACB/IFNG/GGPS1/PDK4/CD74/NR1H3
BP	GO:1904407	positive regulation of nitric oxide metabolic process	5/189	39/18862	4.18E-05	2.03E-03	1.39E-03	HBB/PTK2B/HSP90AB1/TLR4/IFNG
BP	GO:0006631	fatty acid metabolic process	14/189	392/18862	4.30E-05	2.07E-03	1.42E-03	HSD17B4/ACADVL/FABP3/ALOX12/PDK2/CYP7A1/ACACB/CYP2E1/PPARG/ECI2/ECHS1/PDK4/CD74/NR1H3
BP	GO:0045637	regulation of myeloid cell differentiation	11/189	258/18862	6.09E-05	2.89E-03	1.98E-03	RARG/EP300/PTK2B/B2M/TLR4/HLA-DRB1/RARA/IFNG/PRKCB/HMGB3/CD74
BP	GO:0008584	male gonad development	8/189	134/18862	6.23E-05	2.92E-03	2.00E-03	CSDE1/RRM1/HSD17B4/HMGCS1/AR/ESR1/MSH2/SRD5A2
BP	GO:0051251	positive regulation of lymphocyte activation	13/189	356/18862	6.48E-05	2.99E-03	2.05E-03	XRCC6/VAV3/BTK/RAC1/MSH2/TLR4/HLA-DRB1/RARA/IFNG/DPP4/CD74/IL2/SHH
BP	GO:0046546	development of primary male sexual characteristics	8/189	135/18862	6.56E-05	3.00E-03	2.05E-03	CSDE1/RRM1/HSD17B4/HMGCS1/AR/ESR1/MSH2/SRD5A2
BP	GO:0034754	cellular hormone metabolic process	8/189	136/18862	6.91E-05	3.07E-03	2.11E-03	HSD17B4/ESR1/TTR/ADH1C/SULT1A1/CYP17A1/SRD5A2/SHH
BP	GO:0045619	regulation of lymphocyte differentiation	9/189	175/18862	6.95E-05	3.07E-03	2.11E-03	XRCC6/BTK/HLA-DRB1/RARA/IFNG/HMGB3/CD74/IL2/SHH
BP	GO:0007548	sex differentiation	11/189	262/18862	6.99E-05	3.07E-03	2.11E-03	CSDE1/RRM1/HSD17B4/HMGCS1/AR/ESR1/MSH2/ANG/CYP17A1/SRD5A2/SHH
BP	GO:0046425	regulation of JAK-STAT cascade	7/189	101/18862	7.06E-05	3.07E-03	2.11E-03	F2/NF2/PTK2B/PPP2R1A/ERBB4/NOTCH1/IFNG
BP	GO:0002699	positive regulation of immune effector process	10/189	219/18862	7.49E-05	3.20E-03	2.19E-03	HMOX1/BTK/MSH2/B2M/TLR4/HLA-DRB1/RARA/IFNG/CD74/IL2
BP	GO:0046620	regulation of organ growth	7/189	102/18862	7.51E-05	3.20E-03	2.19E-03	ACACB/ERBB4/NOTCH1/YY1/STK4/G6PD/GLI1
BP	GO:0048708	astrocyte differentiation	6/189	72/18862	8.44E-05	3.55E-03	2.43E-03	F2/TLR4/IFNGR1/NOTCH1/IFNG/SHH
BP	GO:0001818	negative regulation of cytokine production	13/189	367/18862	8.80E-05	3.66E-03	2.51E-03	F2/HMOX1/BTK/RAC1/HDAC7/HSP90AB1/BPI/TLR4/HLA-DRB1/RARA/YY1/IFNG/FN1

BP	GO:0050920	regulation of chemotaxis	10/189	224/18862	9.03E-05	3.71E-03	2.54E-03	VEGFB/RAC1/PTK2B/F3/DAPK2/NOTCH1/DPP4/OXSR1/SEMA4D/CD74
BP	GO:0006809	nitric oxide biosynthetic process	6/189	73/18862	9.12E-05	3.71E-03	2.54E-03	HBB/RAC1/PTK2B/HSP90AB1/TLR4/IFNG
BP	GO:1903793	positive regulation of anion transport	15/189	478/18862	9.88E-05	3.98E-03	2.73E-03	FABP3/RAC1/HSP90AB1/YWHAQ/TLR4/HLA-DRB1/IFNG/ANG/B3GAT3/ARF6/YWHAH/SFN/NMT1/NR1H3/SHH
BP	GO:0072332	intrinsic apoptotic signaling pathway by p53 class mediator	6/189	75/18862	1.06E-04	4.19E-03	2.87E-03	EP300/MUC1/MSH2/PDK2/FHIT/CD74
BP	GO:0050678	regulation of epithelial cell proliferation	13/189	374/18862	1.06E-04	4.19E-03	2.87E-03	AR/HMOX1/VEGFB/F3/B2M/TLR4/ITGB3/PPARG/NOTCH1/ANG/SFN/GLI1/SHH
BP	GO:0060742	epithelial cell differentiation involved in prostate gland development	3/189	10/18862	1.13E-04	4.30E-03	2.95E-03	AR/PSAP/NOTCH1
BP	GO:0002700	regulation of production of molecular mediator of immune response	8/189	146/18862	1.14E-04	4.30E-03	2.95E-03	HMOX1/BTK/MSH2/B2M/TLR4/PARP3/CD74/IL2
BP	GO:0040013	negative regulation of locomotion	13/189	377/18862	1.15E-04	4.30E-03	2.95E-03	NF2/HMOX1/RAC1/ERBB4/MAGI2/PPARG/NOTCH1/DPP4/APEX1/STARD13/SEMA4D/CD74/SHH
BP	GO:0042113	B cell activation	12/189	326/18862	1.15E-04	4.30E-03	2.95E-03	EP300/VAV3/BTK/PTK2B/MSH2/TLR4/YY1/PRKCB/HMGB3/PARP3/CD74/IL2
BP	GO:0010594	regulation of endothelial cell migration	10/189	231/18862	1.16E-04	4.30E-03	2.95E-03	HMOX1/MAP3K3/RAC1/PTK2B/HDAC7/ALOX12/ITGB3/PPARG/NOTCH1/STARD13
BP	GO:0044282	small molecule catabolic process	14/189	431/18862	1.18E-04	4.30E-03	2.95E-03	HK2/HSD17B4/ACADVL/DCXR/HPD/PAH/SULT1A1/CYP7A1/ACACB/GAD2/PGK1/ECI2/ECHS1/OAT
BP	GO:0046394	carboxylic acid biosynthetic process	12/189	327/18862	1.19E-04	4.30E-03	2.95E-03	HSD17B4/ACADVL/MTAP/ALOX12/PAH/CYP7A1/ACACB/CYP2E1/OAT/PDK4/CD74/NR1H3
BP	GO:1904892	regulation of STAT cascade	7/189	110/18862	1.21E-04	4.30E-03	2.95E-03	F2/NF2/PTK2B/PPP2R1A/ERBB4/NOTCH1/IFNG
BP	GO:1900739	regulation of protein insertion into mitochondrial membrane involved in apoptotic signaling pathway	4/189	26/18862	1.23E-04	4.30E-03	2.95E-03	YWHAQ/YWHAH/SFN/NMT1

BP	GO:1900740	positive regulation of protein insertion into mitochondrial membrane involved in apoptotic signaling pathway	4/189	26/18862	1.23E-04	4.30E-03	2.95E-03	YWHAQ/YWHAH/SFN/NMT1
BP	GO:0046209	nitric oxide metabolic process	6/189	77/18862	1.23E-04	4.30E-03	2.95E-03	HBB/RAC1/PTK2B/HSP90AB1/TLR4/IFNG
BP	GO:0060420	regulation of heart growth	6/189	77/18862	1.23E-04	4.30E-03	2.95E-03	ACACB/ERBB4/NOTCH1/YY1/G6PD/GLI1
BP	GO:0002218	activation of innate immune response	8/189	148/18862	1.25E-04	4.30E-03	2.95E-03	CUL1/EP300/XRCC6/MUC1/HCK/SKP1/TLR4/BTRC
BP	GO:0002285	lymphocyte activation involved in immune response	9/189	189/18862	1.25E-04	4.30E-03	2.95E-03	PTK2B/MSH2/TLR4/HLA-DRB1/RARA/IFNG/PARP3/CD74/IL2
BP	GO:0030336	negative regulation of cell migration	12/189	330/18862	1.29E-04	4.37E-03	3.00E-03	NF2/HMOX1/RAC1/ERBB4/MAGI2/PPARG/NOTCH1/DPP4/APEX1/STARD13/CD74/SHH
BP	GO:0072593	reactive oxygen species metabolic process	11/189	281/18862	1.30E-04	4.37E-03	3.00E-03	F2/HK2/HBB/RAC1/PTK2B/ALOX12/HSP90AB1/TLR4/IFNG/G6PD/PDK4
BP	GO:0008203	cholesterol metabolic process	8/189	149/18862	1.31E-04	4.37E-03	3.00E-03	ACADVL/HMGCS1/CYP7A1/ACACB/GGPS1/G6PD/DHCR24/NPC1L1
BP	GO:2001057	reactive nitrogen species metabolic process	6/189	78/18862	1.32E-04	4.37E-03	3.00E-03	HBB/RAC1/PTK2B/HSP90AB1/TLR4/IFNG
BP	GO:0007050	cell cycle arrest	10/189	235/18862	1.34E-04	4.40E-03	3.02E-03	EP300/MUC1/HSP90AB1/MSH2/FAP/MAGI2/NOTCH1/IFNG/SFN/DHCR24
BP	GO:2000377	regulation of reactive oxygen species metabolic process	9/189	192/18862	1.41E-04	4.59E-03	3.15E-03	F2/HK2/HBB/RAC1/PTK2B/HSP90AB1/TLR4/IFNG/G6PD
BP	GO:0015949	nucleobase-containing small molecule interconversion	4/189	27/18862	1.43E-04	4.60E-03	3.15E-03	RRM1/NME3/DTYMK/DCTD
BP	GO:0008360	regulation of cell shape	8/189	151/18862	1.44E-04	4.60E-03	3.15E-03	F2/RND1/RAC1/HCK/PTK2B/MSN/FN1/SEMA4D
BP	GO:0016053	organic acid biosynthetic process	12/189	335/18862	1.49E-04	4.65E-03	3.19E-03	HSD17B4/ACADVL/MTAP/ALOX12/PAH/CYP7A1/ACACB/CYP2E1/OAT/PDK4/CD74/NR1H3
BP	GO:0006163	purine nucleotide metabolic process	14/189	441/18862	1.50E-04	4.65E-03	3.19E-03	HK2/EP300/HSD17B4/HMGCS1/NME3/PAPSS1/PDK2/SULT1A1/ACACB/PGK1/IFNG/FHIT/GART/PDK4

BP	GO:0010660	regulation of muscle cell apoptotic process	6/189	80/18862	1.52E-04	4.65E-03	3.19E-03	HMOX1/PTK2B/ALOX12/PPARG/IFNG/STK4
BP	GO:0038093	Fc receptor signaling pathway	10/189	239/18862	1.54E-04	4.65E-03	3.19E-03	CUL1/VAV3/BTK/RAC1/HCK/FCGR2A/HSP90AB1/SKP1/NFATC1/BTRC
BP	GO:0046598	positive regulation of viral entry into host cell	3/189	11/18862	1.54E-04	4.65E-03	3.19E-03	TRIM21/HLA-DRB1/CD74
BP	GO:0051974	negative regulation of telomerase activity	3/189	11/18862	1.54E-04	4.65E-03	3.19E-03	POT1/PPARG/PARP3
BP	GO:0075294	positive regulation by symbiont of entry into host	3/189	11/18862	1.54E-04	4.65E-03	3.19E-03	TRIM21/HLA-DRB1/CD74
BP	GO:0051250	negative regulation of lymphocyte activation	8/189	153/18862	1.57E-04	4.71E-03	3.23E-03	BTK/HLA-DRB1/TNFAIP8L2/HMGB3/PARP3/CD74/IL2/SHH
BP	GO:1902107	positive regulation of leukocyte differentiation	8/189	154/18862	1.64E-04	4.85E-03	3.33E-03	XRCC6/BTK/HLA-DRB1/RARA/IFNG/CD74/IL2/SHH
BP	GO:1903708	positive regulation of hemopoiesis	8/189	154/18862	1.64E-04	4.85E-03	3.33E-03	XRCC6/BTK/HLA-DRB1/RARA/IFNG/CD74/IL2/SHH
BP	GO:0046395	carboxylic acid catabolic process	10/189	243/18862	1.76E-04	5.15E-03	3.53E-03	HSD17B4/ACADVL/DCXR/HPD/PAH/ACACB/GAD2/ECI2/ECHS1/OAT
BP	GO:0046165	alcohol biosynthetic process	8/189	156/18862	1.80E-04	5.22E-03	3.58E-03	HMGCS1/PTK2B/CYP7A1/ACACB/GGPS1/G6PD/DHCR24/NPC1L1
BP	GO:0045806	negative regulation of endocytosis	5/189	53/18862	1.86E-04	5.36E-03	3.68E-03	RAC1/EPHA3/ITGB3/ARF6/NR1H3
BP	GO:0030168	platelet activation	8/189	157/18862	1.88E-04	5.37E-03	3.68E-03	F2/VAV3/HBB/ALOX12/TLR4/ITGB3/PRKCB/FN1
BP	GO:2000146	negative regulation of cell motility	12/189	345/18862	1.95E-04	5.43E-03	3.72E-03	NF2/HMOX1/RAC1/ERBB4/MAGI2/PPARG/NOTCH1/DPP4/APEX1/STARD13/CD74/SHH
BP	GO:1902652	secondary alcohol metabolic process	8/189	158/18862	1.96E-04	5.43E-03	3.72E-03	ACADVL/HMGCS1/CYP7A1/ACACB/GGPS1/G6PD/DHCR24/NPC1L1
BP	GO:0002688	regulation of leukocyte chemotaxis	7/189	119/18862	1.98E-04	5.43E-03	3.72E-03	VEGFB/RAC1/PTK2B/DAPK2/DPP4/OXSR1/CD74
BP	GO:0007162	negative regulation of cell adhesion	11/189	295/18862	1.99E-04	5.43E-03	3.72E-03	NF2/MUC1/RND1/ALOX12/HLA-DRB1/NOTCH1/TNFAIP8L2/SEMA4D/CD74/IL2/SHH

BP	GO:0010657	muscle cell apoptotic process	6/189	84/18862	1.99E-04	5.43E-03	3.72E-03	HMOX1/PTK2B/ALOX12/PPARG/IFNG/STK4
BP	GO:0014031	mesenchymal cell development	6/189	84/18862	1.99E-04	5.43E-03	3.72E-03	ERBB4/ANXA6/NOTCH1/FN1/SEMA4D/SHH
BP	GO:0002381	immunoglobulin production involved in immunoglobulin mediated immune response	5/189	54/18862	2.03E-04	5.48E-03	3.76E-03	BTK/MSH2/HLA-DRB1/PARP3/IL2
BP	GO:0042304	regulation of fatty acid biosynthetic process	5/189	54/18862	2.03E-04	5.48E-03	3.76E-03	ACADVL/CYP7A1/PDK4/CD74/NR1H3
BP	GO:0002696	positive regulation of leukocyte activation	13/189	401/18862	2.11E-04	5.65E-03	3.87E-03	XRCC6/VAV3/BTK/RAC1/MSH2/TLR4/HLA-DRB1/RARA/IFNG/DPP4/CD74/IL2/SHH
BP	GO:0050679	positive regulation of epithelial cell proliferation	9/189	203/18862	2.14E-04	5.69E-03	3.90E-03	AR/HMOX1/VEGFB/F3/TLR4/ITGB3/NOTCH1/ANG/SHH
BP	GO:0001844	protein insertion into mitochondrial membrane involved in apoptotic signaling pathway	4/189	30/18862	2.18E-04	5.76E-03	3.95E-03	YWHAQ/YWHAH/SFN/NMT1
BP	GO:0001755	neural crest cell migration	5/189	55/18862	2.22E-04	5.76E-03	3.95E-03	ERBB4/ANXA6/FN1/SEMA4D/SHH
BP	GO:1903428	positive regulation of reactive oxygen species biosynthetic process	5/189	55/18862	2.22E-04	5.76E-03	3.95E-03	HBB/PTK2B/HSP90AB1/TLR4/IFNG
BP	GO:0030307	positive regulation of cell growth	8/189	161/18862	2.23E-04	5.76E-03	3.95E-03	F2/HBEGF/PTK2B/EXOSC9/SFN/FN1/SEMA4D/IL2
BP	GO:0002685	regulation of leukocyte migration	9/189	205/18862	2.30E-04	5.91E-03	4.05E-03	HMOX1/VEGFB/RAC1/PTK2B/MSN/DAPK2/DPP4/OXSR1/CD74
BP	GO:0051271	negative regulation of cellular component movement	12/189	352/18862	2.34E-04	5.97E-03	4.09E-03	NF2/HMOX1/RAC1/ERBB4/MAGI2/PPARG/NOTCH1/DPP4/APEX1/STARD13/CD74/SHH
BP	GO:0002064	epithelial cell development	9/189	207/18862	2.47E-04	6.26E-03	4.29E-03	RARG/HSD17B4/AR/ESR1/MSN/MAGI2/NOTCH1/TJP1/SFN
BP	GO:0009150	purine ribonucleotide metabolic process	13/189	408/18862	2.50E-04	6.28E-03	4.30E-03	HK2/EP300/HSD17B4/HMGCS1/NME3/PAPSS1/PDK2/SULT1A1/ACACB/PGK1/IFNG/GART/PDK4

BP	GO:0031334	positive regulation of protein complex assembly	10/189	254/18862	2.51E-04	6.28E-03	4.30E-03	ESR1/RAC1/HCK/PTK2B/MSN/TLR4/RPA3/MMP1/IFNG/ARF6
BP	GO:0045582	positive regulation of T cell differentiation	6/189	88/18862	2.56E-04	6.36E-03	4.36E-03	HLA-DRB1/RARA/IFNG/CD74/IL2/SHH
BP	GO:0010745	negative regulation of macrophage derived foam cell differentiation	3/189	13/18862	2.63E-04	6.41E-03	4.39E-03	ITGB3/PPARG/NR1H3
BP	GO:1903054	negative regulation of extracellular matrix organization	3/189	13/18862	2.63E-04	6.41E-03	4.39E-03	FAP/NOTCH1/DPP4
BP	GO:0016125	sterol metabolic process	8/189	165/18862	2.63E-04	6.41E-03	4.39E-03	ACADVL/HMGCS1/CYP7A1/ACACB/GGPS1/G6PD/DHCR24/NPC1L1
BP	GO:0050867	positive regulation of cell activation	13/189	412/18862	2.74E-04	6.59E-03	4.52E-03	XRCC6/VAV3/BTK/RAC1/MSH2/TLR4/HLA-DRB1/RARA/IFNG/DPP4/CD74/IL2/SHH
BP	GO:0007259	JAK-STAT cascade	8/189	166/18862	2.75E-04	6.59E-03	4.52E-03	F2/NF2/PTK2B/PPP2R1A/ERBB4/NOTCH1/IFNG/IL2
BP	GO:0090022	regulation of neutrophil chemotaxis	4/189	32/18862	2.82E-04	6.73E-03	4.61E-03	RAC1/DAPK2/DPP4/CD74
BP	GO:0016054	organic acid catabolic process	10/189	258/18862	2.85E-04	6.75E-03	4.63E-03	HSD17B4/ACADVL/DCXR/HPD/PAH/ACACB/GAD2/ECI2/ECHS1/OAT
BP	GO:0016049	cell growth	14/189	470/18862	2.88E-04	6.78E-03	4.65E-03	F2/RARG/HBEGF/PTK2B/PPP2R1A/HSP90AB1/EXOSC9/PPARG/YY1/SFN/G6PD/FN1/SEMA4D/IL2
BP	GO:1905477	positive regulation of protein localization to membrane	7/189	127/18862	2.95E-04	6.82E-03	4.68E-03	EPHA3/YWHAQ/IFNG/ARF6/YWHAH/SFN/NMT1
BP	GO:0007163	establishment or maintenance of cell polarity	9/189	212/18862	2.95E-04	6.82E-03	4.68E-03	RND1/RAC1/PTK2B/HSP90AB1/MSN/WEE1/ARF6/NDC80/SHH
BP	GO:0008406	gonad development	9/189	212/18862	2.95E-04	6.82E-03	4.68E-03	CSDE1/RRM1/HSD17B4/HMGCS1/AR/ESR1/MSH2/ANG/SRD5A2
BP	GO:0006633	fatty acid biosynthetic process	8/189	168/18862	2.98E-04	6.84E-03	4.69E-03	ACADVL/ALOX12/CYP7A1/ACACB/CYP2E1/PDK4/CD74/NR1H3
BP	GO:0002690	positive regulation of leukocyte chemotaxis	6/189	91/18862	3.08E-04	6.98E-03	4.79E-03	VEGFB/RAC1/PTK2B/DAPK2/OXSR1/CD74

BP	GO:0034502	protein localization to chromosome	6/189	91/18862	3.08E-04	6.98E-03	4.79E-03	POT1/ESR1/MSH2/CTCF/NDC80/PARP3
BP	GO:0050730	regulation of peptidyl-tyrosine phosphorylation	10/189	262/18862	3.22E-04	7.19E-03	4.93E-03	NF2/HBEGF/PTK2B/PPP2R1A/ERBB4/ITGB3/IFNG/SEMA4D/CD74/IL2
BP	GO:0051099	positive regulation of binding	8/189	170/18862	3.22E-04	7.19E-03	4.93E-03	EP300/TRIM21/HSP90AB1/B2M/PPARG/RARA/IFNG/STK4
BP	GO:0030099	myeloid cell differentiation	13/189	419/18862	3.22E-04	7.19E-03	4.93E-03	RARG/EP300/PTK2B/B2M/TLR4/HLA-DRB1/PPARG/RARA/IFNG/PRKCB/HMGB3/G6PD/CD74
BP	GO:0045137	development of primary sexual characteristics	9/189	217/18862	3.50E-04	7.76E-03	5.32E-03	CSDE1/RRM1/HSD17B4/HMGCS1/AR/ESR1/MSH2/ANG/SRD5A2
BP	GO:0030888	regulation of B cell proliferation	5/189	61/18862	3.62E-04	7.90E-03	5.41E-03	VAV3/BTK/TLR4/CD74/IL2
BP	GO:0090181	regulation of cholesterol metabolic process	5/189	61/18862	3.62E-04	7.90E-03	5.41E-03	ACADVL/HMGCS1/CYP7A1/ACACB/GGPS1
BP	GO:0051091	positive regulation of DNA-binding transcription factor activity	10/189	266/18862	3.63E-04	7.90E-03	5.41E-03	EP300/AR/BTK/ESR1/TRIM21/TLR4/ALK/PPARG/PRKCB/SHH
BP	GO:0009259	ribonucleotide metabolic process	13/189	425/18862	3.69E-04	7.95E-03	5.45E-03	HK2/EP300/HSD17B4/HMGCS1/NME3/PAPSS1/PDK2/SULT1A1/ACACB/PGK1/IFNG/GART/PDK4
BP	GO:0045785	positive regulation of cell adhesion	13/189	425/18862	3.69E-04	7.95E-03	5.45E-03	VAV3/RAC1/PTK2B/HLA-DRB1/RARA/TJP1/IFNG/STK4/DPP4/FN1/CD74/IL2/SHH
BP	GO:0010595	positive regulation of endothelial cell migration	7/189	132/18862	3.73E-04	7.97E-03	5.46E-03	HMOX1/MAP3K3/RAC1/PTK2B/HDAC7/ALOX12/ITGB3
BP	GO:0032388	positive regulation of intracellular transport	9/189	219/18862	3.75E-04	7.97E-03	5.46E-03	HSP90AB1/MSN/YWHAQ/IFNG/B3GAT3/YWHAH/SFN/NMT1/SHH
BP	GO:0051235	maintenance of location	11/189	319/18862	3.87E-04	8.19E-03	5.62E-03	F2/HK2/PTK2B/SKP1/ACACB/ITGB3/ANXA6/PPARG/ATP7B/ALB/NR1H3
BP	GO:0043534	blood vessel endothelial cell migration	8/189	175/18862	3.92E-04	8.19E-03	5.62E-03	HMOX1/MAP3K3/PTK2B/HDAC7/ALOX12/PPARG/NOTCH1/STARD13
BP	GO:0097696	STAT cascade	8/189	175/18862	3.92E-04	8.19E-03	5.62E-03	F2/NF2/PTK2B/PPP2R1A/ERBB4/NOTCH1/IFNG/IL2

BP	GO:0010975	regulation of neuron projection development	13/189	428/18862	3.95E-04	8.21E-03	5.63E-03	EP300/MARK1/PTPRF/EPHA3/PTK2B/B2M/MAGI2/ALK/ARF6/YWHAH/RTN4IP1/FN1/SEMA4D
BP	GO:1901030	positive regulation of mitochondrial outer membrane permeabilization involved in apoptotic signaling pathway	4/189	35/18862	4.01E-04	8.30E-03	5.69E-03	YWHAQ/YWHAH/SFN/NMT1
BP	GO:0030258	lipid modification	10/189	270/18862	4.08E-04	8.30E-03	5.69E-03	HSD17B4/ACADVL/FABP3/ALOX12/ACACB/CYP2E1/PPARG/ECI2/ECHS1/PDK4
BP	GO:0045930	negative regulation of mitotic cell cycle	11/189	321/18862	4.08E-04	8.30E-03	5.69E-03	CUL1/EP300/MUC1/MSH2/SKP1/WEE1/FAP/MAGI2/NAE1/SFN/NDC80
BP	GO:0048010	vascular endothelial growth factor receptor signaling pathway	6/189	96/18862	4.11E-04	8.30E-03	5.69E-03	VAV3/VEGFB/RAC1/PTK2B/ITGB3/PRKCB
BP	GO:0009263	deoxyribonucleotide biosynthetic process	3/189	15/18862	4.12E-04	8.30E-03	5.69E-03	RRM1/DTYMK/DCTD
BP	GO:0071391	cellular response to estrogen stimulus	3/189	15/18862	4.12E-04	8.30E-03	5.69E-03	AR/ESR1/RARA
BP	GO:0050773	regulation of dendrite development	6/189	97/18862	4.34E-04	8.69E-03	5.96E-03	MARK1/ALK/ARF6/YWHAH/RTN4IP1/SEMA4D
BP	GO:0072330	monocarboxylic acid biosynthetic process	9/189	224/18862	4.42E-04	8.80E-03	6.03E-03	HSD17B4/ACADVL/ALOX12/CYP7A1/ACACB/CYP2E1/PDK4/CD74/NR1H3
BP	GO:0051148	negative regulation of muscle cell differentiation	5/189	64/18862	4.53E-04	8.97E-03	6.15E-03	NFATC1/NOTCH1/YY1/G6PD/SHH
BP	GO:0090316	positive regulation of intracellular protein transport	8/189	179/18862	4.55E-04	8.97E-03	6.15E-03	HSP90AB1/YWHAQ/IFNG/B3GAT3/YWHAH/SFN/NMT1/SHH
BP	GO:0030522	intracellular receptor signaling pathway	10/189	274/18862	4.58E-04	8.98E-03	6.15E-03	RARG/EP300/AR/ESR1/TLR4/PPARG/RARA/XIAP/YWHAH/NR1H3
BP	GO:0030010	establishment of cell polarity	7/189	137/18862	4.67E-04	9.05E-03	6.21E-03	PTK2B/HSP90AB1/MSN/WEE1/ARF6/NDC80/SHH
BP	GO:0030183	B cell differentiation	7/189	137/18862	4.67E-04	9.05E-03	6.21E-03	EP300/BTK/PTK2B/MSH2/YY1/HMGB3/IL2
BP	GO:0022407	regulation of cell-cell adhesion	13/189	437/18862	4.80E-04	9.27E-03	6.36E-03	NF2/RAC1/ALOX12/HLA-DRB1/NOTCH1/RARA/TJP1/IFNG/TNFAIP8L2/DPP4/CD74/IL2/SHH

BP	GO:1903426	regulation of reactive oxygen species biosynthetic process	6/189	99/18862	4.84E-04	9.30E-03	6.37E-03	HBB/RAC1/PTK2B/HSP90AB1/TLR4/IFNG
BP	GO:0051051	negative regulation of transport	13/189	438/18862	4.91E-04	9.38E-03	6.43E-03	HMOX1/RAC1/EPHA3/PTK2B/YWHAQ/ITGB3/NOTCH1/ARF6/PRKCB/OXSR1/CD74/NR1H3/SHH
BP	GO:0042176	regulation of protein catabolic process	12/189	383/18862	5.01E-04	9.47E-03	6.49E-03	PTK2B/WWP1/HSP90AB1/MSN/RAB26/ATXN3/ODC1/IFNG/SNX1/BTRC/FHIT/SHH
BP	GO:0045591	positive regulation of regulatory T cell differentiation	3/189	16/18862	5.04E-04	9.47E-03	6.49E-03	HLA-DRB1/IFNG/IL2
BP	GO:0050921	positive regulation of chemotaxis	7/189	139/18862	5.09E-04	9.47E-03	6.49E-03	VEGFB/RAC1/PTK2B/F3/DAPK2/OXSR1/CD74
BP	GO:0055076	transition metal ion homeostasis	7/189	139/18862	5.09E-04	9.47E-03	6.49E-03	CUL1/HMOX1/SKP1/B2M/ATP7B/IFNG/XIAP
BP	GO:0008630	intrinsic apoptotic signaling pathway in response to DNA damage	6/189	100/18862	5.11E-04	9.47E-03	6.49E-03	EP300/HMOX1/MUC1/MSH2/SFN/CD74
BP	GO:0060419	heart growth	6/189	100/18862	5.11E-04	9.47E-03	6.49E-03	ACACB/ERBB4/NOTCH1/YY1/G6PD/GLI1
BP	GO:0042593	glucose homeostasis	9/189	229/18862	5.18E-04	9.56E-03	6.55E-03	HK2/RAC1/IGF1R/PYGL/PDK2/CYP7A1/HLA-DRB1/PPARG/PDK4
BP	GO:0070482	response to oxygen levels	12/189	385/18862	5.25E-04	9.56E-03	6.55E-03	HK2/EP300/MMP2/HMOX1/VEGFB/PTK2B/FAS/PPARG/PGK1/NOTCH1/ANG/DPP4
BP	GO:1902105	regulation of leukocyte differentiation	10/189	279/18862	5.27E-04	9.56E-03	6.55E-03	XRCC6/BTK/TLR4/HLA-DRB1/RARA/IFNG/HMGB3/CD74/IL2/SHH
BP	GO:0033865	nucleoside bisphosphate metabolic process	7/189	140/18862	5.31E-04	9.56E-03	6.55E-03	HSD17B4/HMGCS1/PAPSS1/PDK2/SULT1A1/ACACB/PDK4
BP	GO:0033875	ribonucleoside bisphosphate metabolic process	7/189	140/18862	5.31E-04	9.56E-03	6.55E-03	HSD17B4/HMGCS1/PAPSS1/PDK2/SULT1A1/ACACB/PDK4
BP	GO:0034032	purine nucleoside bisphosphate metabolic process	7/189	140/18862	5.31E-04	9.56E-03	6.55E-03	HSD17B4/HMGCS1/PAPSS1/PDK2/SULT1A1/ACACB/PDK4
BP	GO:0033500	carbohydrate homeostasis	9/189	230/18862	5.34E-04	9.56E-03	6.55E-03	HK2/RAC1/IGF1R/PYGL/PDK2/CYP7A1/HLA-DRB1/PPARG/PDK4

BP	GO:1902106	negative regulation of leukocyte differentiation	6/189	101/18862	5.39E-04	9.56E-03	6.55E-03	TLR4/RARA/HMGB3/CD74/IL2/SHH
BP	GO:2000379	positive regulation of reactive oxygen species metabolic process	6/189	101/18862	5.39E-04	9.56E-03	6.55E-03	F2/HBB/PTK2B/HSP90AB1/TLR4/IFNG
BP	GO:0002040	sprouting angiogenesis	8/189	184/18862	5.46E-04	9.62E-03	6.59E-03	HMOX1/VEGFB/MAP3K3/PTK2B/HDAC7/NOTCH1/TJP1/STARD13
BP	GO:0006084	acetyl-CoA metabolic process	4/189	38/18862	5.52E-04	9.62E-03	6.59E-03	HMGCS1/PDK2/ACACB/PDK4
BP	GO:0010742	macrophage derived foam cell differentiation	4/189	38/18862	5.52E-04	9.62E-03	6.59E-03	EP300/ITGB3/PPARG/NR1H3
BP	GO:0090077	foam cell differentiation	4/189	38/18862	5.52E-04	9.62E-03	6.59E-03	EP300/ITGB3/PPARG/NR1H3
BP	GO:0051090	regulation of DNA-binding transcription factor activity	13/189	444/18862	5.57E-04	9.66E-03	6.62E-03	EP300/AR/HMOX1/BTK/ESR1/HCK/TRIM21/TLR4/ALK/PPARG/BTRC/PRKCB/SHH
BP	GO:0046637	regulation of alpha-beta T cell differentiation	5/189	67/18862	5.59E-04	9.66E-03	6.62E-03	HLA-DRB1/RARA/IFNG/IL2/SHH
BP	GO:0042116	macrophage activation	6/189	102/18862	5.68E-04	9.75E-03	6.68E-03	BPI/TLR4/IFNGR1/IFNG/CD74/NR1H3
BP	GO:0007249	I-kappaB kinase/NF-kappaB signaling	10/189	282/18862	5.73E-04	9.79E-03	6.71E-03	HMOX1/BTK/MAP3K3/ESR1/TRIM21/TLR4/HLA-DRB1/BTRC/PRKCB/CD74
BP	GO:0045765	regulation of angiogenesis	11/189	335/18862	5.83E-04	9.92E-03	6.80E-03	HK2/HMOX1/VEGFB/PTK2B/F3/GTF2I/PPARG/PGK1/TJP1/PRKCB/THBS2
BP	GO:0042063	gliogenesis	10/189	283/18862	5.89E-04	9.93E-03	6.81E-03	F2/NF2/PTK2B/NSUN5/TLR4/IFNGR1/PPARG/NOTCH1/IFNG/SHH
BP	GO:0097193	intrinsic apoptotic signaling pathway	10/189	283/18862	5.89E-04	9.93E-03	6.81E-03	CUL1/EP300/HMOX1/MUC1/MSH2/PDK2/DAPK2/FHIT/SFN/CD74
BP	GO:0006576	cellular biogenic amine metabolic process	6/189	103/18862	5.98E-04	1.00E-02	6.87E-03	DHPS/PAH/SULT1A1/ODC1/PNMT/CHKA
BP	GO:0034393	positive regulation of smooth muscle cell apoptotic process	3/189	17/18862	6.07E-04	1.00E-02	6.87E-03	PPARG/IFNG/STK4

BP	GO:0060749	mammary gland alveolus development	3/189	17/18862	6.07E-04	1.00E-02	6.87E-03	AR/ESR1/ERBB4
BP	GO:0061377	mammary gland lobule development	3/189	17/18862	6.07E-04	1.00E-02	6.87E-03	AR/ESR1/ERBB4
BP	GO:0006367	transcription initiation from RNA polymerase II promoter	8/189	187/18862	6.08E-04	1.00E-02	6.87E-03	RARG/AR/ESR1/PPARG/NOTCH1/RARA/TAF13/NR1H3
BP	GO:0002440	production of molecular mediator of immune response	10/189	286/18862	6.39E-04	1.05E-02	7.19E-03	HMOX1/BTK/MSH2/B2M/TLR4/HLA-DRB1/YY1/PARP3/CD74/IL2
BP	GO:0032102	negative regulation of response to external stimulus	12/189	394/18862	6.44E-04	1.05E-02	7.21E-03	F2/TRIM21/ALOX12/FAP/HLA-DRB1/PPARG/NOTCH1/TNFAIP8L2/DPP4/SEMA4D/IL2/NR1H3
BP	GO:0022408	negative regulation of cell-cell adhesion	8/189	189/18862	6.52E-04	1.06E-02	7.27E-03	NF2/ALOX12/HLA-DRB1/NOTCH1/TNFAIP8L2/CD74/IL2/SHH
BP	GO:1903707	negative regulation of hemopoiesis	6/189	105/18862	6.62E-04	1.07E-02	7.36E-03	TLR4/RARA/HMGB3/CD74/IL2/SHH
BP	GO:0030890	positive regulation of B cell proliferation	4/189	40/18862	6.73E-04	1.08E-02	7.44E-03	VAV3/TLR4/CD74/IL2
BP	GO:1901342	regulation of vasculature development	11/189	341/18862	6.75E-04	1.08E-02	7.44E-03	HK2/HMOX1/VEGFB/PTK2B/F3/GTF2I/PPARG/PGK1/TJP1/PRKCB/THBS2
BP	GO:0055021	regulation of cardiac muscle tissue growth	5/189	70/18862	6.84E-04	1.10E-02	7.51E-03	ERBB4/NOTCH1/YY1/G6PD/GLI1
BP	GO:0070997	neuron death	11/189	342/18862	6.91E-04	1.10E-02	7.55E-03	HMOX1/PTK2B/HSP90AB1/MSH2/TLR4/FAS/NAE1/IFNG/XIAP/G6PD/DHCR24
BP	GO:0150115	NA	6/189	106/18862	6.96E-04	1.10E-02	7.57E-03	RAC1/EPHA3/PTK2B/BCR/ARF6/FN1
BP	GO:0070227	lymphocyte apoptotic process	5/189	71/18862	7.30E-04	1.15E-02	7.91E-03	BTK/DFFA/FAS/CD74/IL2
BP	GO:0050731	positive regulation of peptidyl-tyrosine phosphorylation	8/189	193/18862	7.47E-04	1.17E-02	8.03E-03	HBEGF/PTK2B/ERBB4/ITGB3/IFNG/SEMA4D/CD74/IL2
BP	GO:0050864	regulation of B cell activation	8/189	193/18862	7.47E-04	1.17E-02	8.03E-03	VAV3/BTK/MSH2/TLR4/HMGB3/PARP3/CD74/IL2
BP	GO:1905475	regulation of protein localization to membrane	8/189	194/18862	7.73E-04	1.21E-02	8.27E-03	AR/EPHA3/YWHAQ/IFNG/ARF6/YWHAH/SFN/NMT1

BP	GO:0062197	NA	11/189	347/18862	7.79E-04	1.21E-02	8.29E-03	XRCC6/MMP2/HMOX1/BTK/PDK2/TLR4/FAS/PPARG/APEX1/OXSR1/G6PD
BP	GO:0046390	ribose phosphate biosynthetic process	8/189	195/18862	7.99E-04	1.23E-02	8.42E-03	NME3/PAPSS1/PYGL/PDK2/ACACB/GART/G6PD/PDK4
BP	GO:0043535	regulation of blood vessel endothelial cell migration	7/189	150/18862	8.00E-04	1.23E-02	8.42E-03	HMOX1/MAP3K3/HDAC7/ALOX12/PPARG/NOTCH1/STARD13
BP	GO:0050777	negative regulation of immune response	7/189	150/18862	8.00E-04	1.23E-02	8.42E-03	HMOX1/TRIM21/HLA-DRB1/PPARG/PARP3/IL2/NR1H3
BP	GO:0032963	collagen metabolic process	6/189	109/18862	8.06E-04	1.23E-02	8.44E-03	F2/MMP2/CYP7A1/FAP/PPARG/MMP1
BP	GO:0048806	genitalia development	4/189	42/18862	8.12E-04	1.23E-02	8.44E-03	AR/ESR1/SRD5A2/SHH
BP	GO:1902622	regulation of neutrophil migration	4/189	42/18862	8.12E-04	1.23E-02	8.44E-03	RAC1/DAPK2/DPP4/CD74
BP	GO:0043122	regulation of I-kappaB kinase/NF-kappaB signaling	9/189	244/18862	8.15E-04	1.23E-02	8.44E-03	HMOX1/MAP3K3/ESR1/TRIM21/TLR4/HLA-DRB1/BTRC/PRKCB/CD74
BP	GO:0001558	regulation of cell growth	12/189	406/18862	8.36E-04	1.26E-02	8.62E-03	F2/HBEGF/PTK2B/PPP2R1A/EXOSC9/PPARG/YY1/SFN/G6PD/FN1/SEMA4D/IL2
BP	GO:0002902	regulation of B cell apoptotic process	3/189	19/18862	8.52E-04	1.27E-02	8.73E-03	BTK/CD74/IL2
BP	GO:0002697	regulation of immune effector process	13/189	465/18862	8.54E-04	1.27E-02	8.73E-03	F2/HMOX1/BTK/MSH2/B2M/TLR4/HLA-DRB1/RARA/IFNG/C1R/PARP3/CD74/IL2
BP	GO:0044843	cell cycle G1/S phase transition	10/189	298/18862	8.75E-04	1.30E-02	8.91E-03	CUL1/EP300/MUC1/SENP2/WEE1/RPA3/APEX1/SFN/GLI1/POLA1
BP	GO:0051054	positive regulation of DNA metabolic process	8/189	198/18862	8.82E-04	1.31E-02	8.95E-03	POT1/PTK2B/HSP90AB1/MSH2/HNRNPA2B1/PARN/PARP3/IL2
BP	GO:0070266	necroptotic process	4/189	43/18862	8.88E-04	1.31E-02	8.98E-03	PYGL/ITPK1/TLR4/FAS
BP	GO:0051052	regulation of DNA metabolic process	11/189	353/18862	8.95E-04	1.31E-02	9.01E-03	POT1/DFFA/PTK2B/HSP90AB1/MSH2/SMG5/HNRNPA2B1/PARN/PPARG/PARP3/IL2
BP	GO:0006635	fatty acid beta-oxidation	5/189	75/18862	9.37E-04	1.36E-02	9.33E-03	HSD17B4/ACADVL/ACACB/ECI2/ECHS1

BP	GO:0046902	regulation of mitochondrial membrane permeability	5/189	75/18862	9.37E-04	1.36E-02	9.33E-03	HK2/YWHAQ/YWHAH/SFN/NMT1
BP	GO:1902930	regulation of alcohol biosynthetic process	5/189	75/18862	9.37E-04	1.36E-02	9.33E-03	HMGCS1/PTK2B/CYP7A1/ACACB/GGPS1
BP	GO:0042771	intrinsic apoptotic signaling pathway in response to DNA damage by p53 class mediator	4/189	44/18862	9.69E-04	1.39E-02	9.50E-03	EP300/MUC1/MSH2/CD74
BP	GO:0046427	positive regulation of JAK-STAT cascade	4/189	44/18862	9.69E-04	1.39E-02	9.50E-03	F2/PTK2B/ERBB4/NOTCH1
BP	GO:0060421	positive regulation of heart growth	4/189	44/18862	9.69E-04	1.39E-02	9.50E-03	ACACB/ERBB4/NOTCH1/GLI1
BP	GO:1901028	regulation of mitochondrial outer membrane permeabilization involved in apoptotic signaling pathway	4/189	44/18862	9.69E-04	1.39E-02	9.50E-03	YWHAQ/YWHAH/SFN/NMT1
BP	GO:0051098	regulation of binding	11/189	357/18862	9.81E-04	1.40E-02	9.57E-03	EP300/HMOX1/PTPRF/TRIM21/HSP90AB1/SENP2/B2M/PPARG/RARA/IFNG/STK4
BP	GO:0030539	male genitalia development	3/189	20/18862	9.96E-04	1.41E-02	9.64E-03	AR/SRD5A2/SHH
BP	GO:0060444	branching involved in mammary gland duct morphogenesis	3/189	20/18862	9.96E-04	1.41E-02	9.64E-03	AR/ESR1/BTRC
BP	GO:0090049	regulation of cell migration involved in sprouting angiogenesis	5/189	77/18862	1.06E-03	1.49E-02	1.02E-02	HMOX1/MAP3K3/HDAC7/NOTCH1/STARD13
BP	GO:0060326	cell chemotaxis	10/189	306/18862	1.07E-03	1.50E-02	1.03E-02	HBEGF/VAV3/VEGFB/RAC1/PTK2B/DAPK2/NOTCH1/DPP4/OXSR1/CD74
BP	GO:0031349	positive regulation of defense response	11/189	361/18862	1.07E-03	1.50E-02	1.03E-02	CUL1/EP300/XRCC6/BTK/MUC1/HCK/SKP1/TLR4/IFNG/BTRC/IL2
BP	GO:1901987	regulation of cell cycle phase transition	13/189	478/18862	1.10E-03	1.53E-02	1.05E-02	CUL1/EP300/MUC1/PPP2R1A/SENP2/SKP1/WEE1/NAE1/BTRC/APEX1/SFN/NDC80/GLI1
BP	GO:0007093	mitotic cell cycle checkpoint	7/189	159/18862	1.13E-03	1.56E-02	1.07E-02	EP300/MUC1/MSH2/WEE1/NAE1/SFN/NDC80

BP	GO:0030100	regulation of endocytosis	8/189	206/18862	1.14E-03	1.57E-02	1.08E-02	RAC1/EPHA3/B2M/ITGB3/MAGI2/PPARG/ARF6/NR1H3
BP	GO:0031648	protein destabilization	4/189	46/18862	1.15E-03	1.57E-02	1.08E-02	EP300/TRIM21/SENP2/BTRC
BP	GO:0048146	positive regulation of fibroblast proliferation	4/189	46/18862	1.15E-03	1.57E-02	1.08E-02	ESR1/S100A6/FN1/CD74
BP	GO:0060562	epithelial tube morphogenesis	10/189	309/18862	1.15E-03	1.57E-02	1.08E-02	RARG/AR/ESR1/ALOX12/NOTCH1/ZIC3/BTRC/STK4/STARD13/SHH
BP	GO:0002429	immune response-activating cell surface receptor signaling pathway	13/189	481/18862	1.16E-03	1.57E-02	1.08E-02	CUL1/EP300/VAV3/BTK/MUC1/RAC1/HCK/FCGR2A/HSP90AB1/SKP1/HLA-DRB1/BTRC/PRKCB
BP	GO:0002757	immune response-activating signal transduction	13/189	481/18862	1.16E-03	1.57E-02	1.08E-02	CUL1/EP300/VAV3/BTK/MUC1/RAC1/HCK/FCGR2A/HSP90AB1/SKP1/HLA-DRB1/BTRC/PRKCB
BP	GO:0051960	regulation of nervous system development	12/189	422/18862	1.16E-03	1.57E-02	1.08E-02	F2/RARG/NF2/B2M/NSUN5/PPARG/NOTCH1/IFNG/YWHAH/FN1/SEMA4D/SHH
BP	GO:0030111	regulation of Wnt signaling pathway	11/189	365/18862	1.17E-03	1.58E-02	1.08E-02	ESR1/PPP2R1A/SENP2/CSNK1G2/NFATC1/NOTCH1/BTRC/STK4/XIAP/GLI1/SHH
BP	GO:0002312	B cell activation involved in immune response	5/189	79/18862	1.19E-03	1.59E-02	1.09E-02	PTK2B/MSH2/TLR4/PARP3/IL2
BP	GO:0048145	regulation of fibroblast proliferation	5/189	79/18862	1.19E-03	1.59E-02	1.09E-02	ESR1/PPARG/S100A6/FN1/CD74
BP	GO:0007159	leukocyte cell-cell adhesion	11/189	366/18862	1.20E-03	1.59E-02	1.09E-02	RAC1/MSN/HLA-DRB1/RARA/IFNG/TNFAIP8L2/DPP4/SEMA4D/CD74/IL2/SHH
BP	GO:0050727	regulation of inflammatory response	11/189	366/18862	1.20E-03	1.59E-02	1.09E-02	BTK/ESR1/HCK/TLR4/HLA-DRB1/PPARG/IFNG/TNFAIP8L2/XIAP/IL2/NR1H3
BP	GO:0002833	positive regulation of response to biotic stimulus	9/189	258/18862	1.20E-03	1.59E-02	1.09E-02	CUL1/EP300/XRCC6/MUC1/HCK/SKP1/TLR4/HLA-DRB1/BTRC
BP	GO:0008637	apoptotic mitochondrial changes	6/189	118/18862	1.22E-03	1.61E-02	1.10E-02	HK2/YWHAQ/ERBB4/YWHAH/SFN/NMT1
BP	GO:0010712	regulation of collagen metabolic process	4/189	47/18862	1.24E-03	1.61E-02	1.11E-02	F2/CYP7A1/FAP/PPARG

BP	GO:0035722	interleukin-12-mediated signaling pathway	4/189	47/18862	1.24E-03	1.61E-02	1.11E-02	MTAP/MSN/HNRNPA2B1/IFNG
BP	GO:0120163	negative regulation of cold-induced thermogenesis	4/189	47/18862	1.24E-03	1.61E-02	1.11E-02	ACVR2B/TLR4/NOTCH1/NR1H3
BP	GO:0002703	regulation of leukocyte mediated immunity	8/189	209/18862	1.25E-03	1.61E-02	1.11E-02	HMOX1/BTK/MSH2/B2M/TLR4/HLA-DRB1/PARP3/IL2
BP	GO:0010833	telomere maintenance via telomere lengthening	5/189	80/18862	1.25E-03	1.61E-02	1.11E-02	POT1/HSP90AB1/SMG5/HNRNPA2B1/PARN
BP	GO:0014032	neural crest cell development	5/189	80/18862	1.25E-03	1.61E-02	1.11E-02	ERBB4/ANXA6/FN1/SEMA4D/SHH
BP	GO:0048144	fibroblast proliferation	5/189	80/18862	1.25E-03	1.61E-02	1.11E-02	ESR1/PPARG/S100A6/FN1/CD74
BP	GO:0001894	tissue homeostasis	9/189	260/18862	1.27E-03	1.63E-02	1.11E-02	RAC1/PTK2B/B2M/TLR4/ITGB3/NOTCH1/TJP1/ALB/PDK4
BP	GO:0002758	innate immune response-activating signal transduction	6/189	119/18862	1.27E-03	1.63E-02	1.11E-02	CUL1/EP300/MUC1/HCK/SKP1/BTRC
BP	GO:0006006	glucose metabolic process	8/189	210/18862	1.29E-03	1.63E-02	1.12E-02	HK2/EP300/DCXR/PDK2/ACACB/PGK1/G6PD/PDK4
BP	GO:0010001	glial cell differentiation	8/189	210/18862	1.29E-03	1.63E-02	1.12E-02	F2/NSUN5/TLR4/IFNGR1/PPARG/NOTCH1/IFNG/SHH
BP	GO:0009308	amine metabolic process	7/189	163/18862	1.30E-03	1.64E-02	1.13E-02	DHPS/PAH/SULT1A1/ODC1/PNMT/AOC3/CHKA
BP	GO:0010888	negative regulation of lipid storage	3/189	22/18862	1.33E-03	1.67E-02	1.14E-02	ITGB3/PPARG/NR1H3
BP	GO:0022617	extracellular matrix disassembly	5/189	81/18862	1.33E-03	1.67E-02	1.14E-02	MMP2/BMP1/FAP/MMP1/DPP4
BP	GO:0045540	regulation of cholesterol biosynthetic process	4/189	48/18862	1.35E-03	1.67E-02	1.14E-02	HMGCS1/CYP7A1/ACACB/GGPS1
BP	GO:0051204	protein insertion into mitochondrial membrane	4/189	48/18862	1.35E-03	1.67E-02	1.14E-02	YWHAQ/YWHAH/SFN/NMT1
BP	GO:0106118	regulation of sterol biosynthetic process	4/189	48/18862	1.35E-03	1.67E-02	1.14E-02	HMGCS1/CYP7A1/ACACB/GGPS1

BP	GO:1904894	positive regulation of STAT cascade	4/189	48/18862	1.35E-03	1.67E-02	1.14E-02	F2/PTK2B/ERBB4/NOTCH1
BP	GO:0050870	positive regulation of T cell activation	8/189	212/18862	1.37E-03	1.69E-02	1.16E-02	RAC1/HLA-DRB1/RARA/IFNG/DPP4/CD74/IL2/SHH
BP	GO:0072331	signal transduction by p53 class mediator	9/189	263/18862	1.38E-03	1.70E-02	1.16E-02	EP300/MUC1/MSH2/PDK2/RPA3/TAF13/FHIT/SFN/CD74
BP	GO:0009165	nucleotide biosynthetic process	9/189	264/18862	1.41E-03	1.73E-02	1.19E-02	RRM1/NME3/DTYMK/PAPSS1/PDK2/ACACB/GART/DCTD/PDK4
BP	GO:0019218	regulation of steroid metabolic process	6/189	122/18862	1.45E-03	1.76E-02	1.21E-02	ACADVL/HMGCS1/CYP7A1/ACACB/IFNG/GGPS1
BP	GO:0046638	positive regulation of alpha-beta T cell differentiation	4/189	49/18862	1.45E-03	1.76E-02	1.21E-02	HLA-DRB1/RARA/IFNG/SHH
BP	GO:0071349	cellular response to interleukin-12	4/189	49/18862	1.45E-03	1.76E-02	1.21E-02	MTAP/MSN/HNRNPA2B1/IFNG
BP	GO:0071622	regulation of granulocyte chemotaxis	4/189	49/18862	1.45E-03	1.76E-02	1.21E-02	RAC1/DAPK2/DPP4/CD74
BP	GO:0048638	regulation of developmental growth	10/189	319/18862	1.46E-03	1.76E-02	1.21E-02	AR/ACACB/ERBB4/NOTCH1/YY1/STK4/G6PD/FN1/SEMA4D/GLI1
BP	GO:0032092	positive regulation of protein binding	5/189	83/18862	1.48E-03	1.78E-02	1.22E-02	EP300/TRIM21/HSP90AB1/B2M/STK4
BP	GO:0048864	stem cell development	5/189	83/18862	1.48E-03	1.78E-02	1.22E-02	ERBB4/ANXA6/FN1/SEMA4D/SHH
BP	GO:1903409	reactive oxygen species biosynthetic process	6/189	123/18862	1.51E-03	1.80E-02	1.24E-02	HBB/RAC1/PTK2B/HSP90AB1/TLR4/IFNG
BP	GO:1901293	nucleoside phosphate biosynthetic process	9/189	267/18862	1.53E-03	1.82E-02	1.25E-02	RRM1/NME3/DTYMK/PAPSS1/PDK2/ACACB/GART/DCTD/PDK4
BP	GO:0002718	regulation of cytokine production involved in immune response	5/189	84/18862	1.56E-03	1.85E-02	1.27E-02	HMOX1/BTK/B2M/TLR4/CD74
BP	GO:0045668	negative regulation of osteoblast differentiation	4/189	50/18862	1.57E-03	1.85E-02	1.27E-02	HDAC7/PPARG/NOTCH1/SEMA4D
BP	GO:0070671	response to interleukin-12	4/189	50/18862	1.57E-03	1.85E-02	1.27E-02	MTAP/MSN/HNRNPA2B1/IFNG

BP	GO:0038095	Fc-epsilon receptor signaling pathway	7/189	169/18862	1.60E-03	1.88E-02	1.29E-02	CUL1/VAV3/BTK/RAC1/SKP1/NFATC1/BTRC
BP	GO:1901990	regulation of mitotic cell cycle phase transition	12/189	439/18862	1.63E-03	1.89E-02	1.30E-02	CUL1/EP300/MUC1/PPP2R1A/SENP2/SKP1/WEE1/NAE1/BTRC/APEX1/SFN/NDC80
BP	GO:0010810	regulation of cell-substrate adhesion	8/189	218/18862	1.63E-03	1.89E-02	1.30E-02	NF2/RAC1/EPHA3/PTK2B/ATXN3/NOTCH1/STK4/FN1
BP	GO:0042445	hormone metabolic process	8/189	218/18862	1.63E-03	1.89E-02	1.30E-02	HSD17B4/ESR1/TTR/ADH1C/SULT1A1/CYP17A1/SRD5A2/SHH
BP	GO:0071695	anatomical structure maturation	8/189	218/18862	1.63E-03	1.89E-02	1.30E-02	MMP2/BTK/RND1/PTK2B/PPARG/ANG/G6PD/SEMA4D
BP	GO:0034109	homotypic cell-cell adhesion	5/189	85/18862	1.64E-03	1.89E-02	1.30E-02	HBB/ALOX12/ITGB3/DSG2/FN1
BP	GO:0042509	regulation of tyrosine phosphorylation of STAT protein	5/189	85/18862	1.64E-03	1.89E-02	1.30E-02	NF2/PPP2R1A/ERBB4/IFNG/IL2
BP	GO:0046889	positive regulation of lipid biosynthetic process	5/189	85/18862	1.64E-03	1.89E-02	1.30E-02	FABP3/CYP7A1/IFNG/CD74/NR1H3
BP	GO:0006909	phagocytosis	11/189	381/18862	1.65E-03	1.89E-02	1.30E-02	VAV3/RAC1/HCK/FCGR2A/HSP90AB1/TLR4/ITGB3/PPARG/RARA/IFNG/NR1H3
BP	GO:0043277	apoptotic cell clearance	4/189	51/18862	1.69E-03	1.92E-02	1.32E-02	RAC1/ITGB3/RARA/NR1H3
BP	GO:0043370	regulation of CD4-positive, alpha-beta T cell differentiation	4/189	51/18862	1.69E-03	1.92E-02	1.32E-02	HLA-DRB1/RARA/IFNG/IL2
BP	GO:0097300	programmed necrotic cell death	4/189	51/18862	1.69E-03	1.92E-02	1.32E-02	PYGL/ITPK1/TLR4/FAS
BP	GO:0003002	regionalization	10/189	326/18862	1.71E-03	1.94E-02	1.33E-02	RARG/EP300/AR/ACVR2B/SENP2/NOTCH1/ZIC3/YY1/GLI1/SHH
BP	GO:0032925	regulation of activin receptor signaling pathway	3/189	24/18862	1.72E-03	1.94E-02	1.33E-02	ACVR2B/MAGI2/SHH
BP	GO:0090023	positive regulation of neutrophil chemotaxis	3/189	24/18862	1.72E-03	1.94E-02	1.33E-02	RAC1/DAPK2/CD74
BP	GO:0055072	iron ion homeostasis	5/189	86/18862	1.73E-03	1.95E-02	1.34E-02	CUL1/HMOX1/SKP1/B2M/IFNG

BP	GO:0006979	response to oxidative stress	12/189	444/18862	1.79E-03	2.00E-02	1.37E-02	MMP2/HMOX1/BTK/HBB/PTK2B/PDK2/TLR4/CYP2E1/APEX1/OXSR1/G6PD/DHCR24
BP	GO:0008361	regulation of cell size	7/189	173/18862	1.83E-03	2.04E-02	1.40E-02	RARG/VAV3/RAC1/HSP90AB1/MSN/FN1/SEMA4D
BP	GO:0002576	platelet degranulation	6/189	128/18862	1.85E-03	2.05E-02	1.40E-02	VEGFB/PSAP/ITGB3/ALB/FN1/ABCC4
BP	GO:0032479	regulation of type I interferon production	6/189	128/18862	1.85E-03	2.05E-02	1.40E-02	EP300/XRCC6/TRIM21/TLR4/YY1/POLA1
BP	GO:0045667	regulation of osteoblast differentiation	6/189	128/18862	1.85E-03	2.05E-02	1.40E-02	HDAC7/ACVR2B/PPARG/NOTCH1/SEMA4D/GLI1
BP	GO:0043254	regulation of protein complex assembly	12/189	446/18862	1.85E-03	2.05E-02	1.40E-02	EP300/ESR1/RAC1/HCK/PTK2B/MSN/TLR4/PEX5/RPA3/MMP1/IFNG/ARF6
BP	GO:0000082	G1/S transition of mitotic cell cycle	9/189	275/18862	1.87E-03	2.05E-02	1.40E-02	CUL1/EP300/MUC1/SENP2/WEE1/RPA3/APEX1/SFN/POLA1
BP	GO:0071214	cellular response to abiotic stimulus	10/189	330/18862	1.87E-03	2.05E-02	1.40E-02	EP300/XRCC6/MMP2/RAC1/TLR4/FAS/MMP1/YY1/OXSR1/NMT1
BP	GO:0104004	cellular response to environmental stimulus	10/189	330/18862	1.87E-03	2.05E-02	1.40E-02	EP300/XRCC6/MMP2/RAC1/TLR4/FAS/MMP1/YY1/OXSR1/NMT1
BP	GO:0001649	osteoblast differentiation	8/189	223/18862	1.88E-03	2.05E-02	1.40E-02	HSD17B4/HDAC7/ACVR2B/PPARG/NOTCH1/SEMA4D/GLI1/SHH
BP	GO:0045089	positive regulation of innate immune response	8/189	223/18862	1.88E-03	2.05E-02	1.40E-02	CUL1/EP300/XRCC6/MUC1/HCK/SKP1/TLR4/BTRC
BP	GO:0022409	positive regulation of cell-cell adhesion	9/189	276/18862	1.92E-03	2.05E-02	1.41E-02	RAC1/HLA-DRB1/RARA/TJP1/IFNG/DPP4/CD74/IL2/SHH
BP	GO:0031098	stress-activated protein kinase signaling cascade	9/189	276/18862	1.92E-03	2.05E-02	1.41E-02	CUL1/MAP3K3/PTK2B/IGF1R/SKP1/TLR4/FAS/BTRC/STK4
BP	GO:0007260	tyrosine phosphorylation of STAT protein	5/189	88/18862	1.92E-03	2.05E-02	1.41E-02	NF2/PPP2R1A/ERBB4/IFNG/IL2
BP	GO:0032606	type I interferon production	6/189	129/18862	1.92E-03	2.05E-02	1.41E-02	EP300/XRCC6/TRIM21/TLR4/YY1/POLA1
BP	GO:0000302	response to reactive oxygen species	8/189	224/18862	1.93E-03	2.05E-02	1.41E-02	MMP2/HMOX1/BTK/HBB/PTK2B/PDK2/CYP2E1/APEX1

BP	GO:0001783	B cell apoptotic process	3/189	25/18862	1.94E-03	2.05E-02	1.41E-02	BTK/CD74/IL2
BP	GO:0008211	glucocorticoid metabolic process	3/189	25/18862	1.94E-03	2.05E-02	1.41E-02	YWHAH/CYP17A1/SERPINA6
BP	GO:0090151	establishment of protein localization to mitochondrial membrane	4/189	53/18862	1.95E-03	2.05E-02	1.41E-02	YWHAQ/YWHAH/SFN/NMT1
BP	GO:0045766	positive regulation of angiogenesis	7/189	175/18862	1.95E-03	2.05E-02	1.41E-02	HK2/HMOX1/VEGFB/PTK2B/F3/TJP1/PRKCB
BP	GO:0048771	tissue remodeling	7/189	175/18862	1.95E-03	2.05E-02	1.41E-02	MMP2/RAC1/PTK2B/ACVR2B/ITGB3/PDK4/IL2
BP	GO:0051897	positive regulation of protein kinase B signaling	7/189	175/18862	1.95E-03	2.05E-02	1.41E-02	HBEGF/ESR1/RAC1/IGF1R/HSP90AB1/F3/ERBB4
BP	GO:1904018	positive regulation of vasculature development	7/189	175/18862	1.95E-03	2.05E-02	1.41E-02	HK2/HMOX1/VEGFB/PTK2B/F3/TJP1/PRKCB
BP	GO:0060560	developmental growth involved in morphogenesis	8/189	225/18862	1.99E-03	2.09E-02	1.43E-02	RARG/ESR1/HSP90AB1/MAGI2/NOTCH1/FN1/SEMA4D/SHH
BP	GO:0030595	leukocyte chemotaxis	8/189	226/18862	2.04E-03	2.14E-02	1.47E-02	VAV3/VEGFB/RAC1/PTK2B/DAPK2/DPP4/OXSR1/CD74
BP	GO:0072329	monocarboxylic acid catabolic process	6/189	131/18862	2.08E-03	2.16E-02	1.48E-02	HSD17B4/ACADVL/DCXR/ACACB/ECI2/ECHS1
BP	GO:0002712	regulation of B cell mediated immunity	4/189	54/18862	2.09E-03	2.16E-02	1.48E-02	BTK/MSH2/PARP3/IL2
BP	GO:0002889	regulation of immunoglobulin mediated immune response	4/189	54/18862	2.09E-03	2.16E-02	1.48E-02	BTK/MSH2/PARP3/IL2
BP	GO:0016447	somatic recombination of immunoglobulin gene segments	4/189	54/18862	2.09E-03	2.16E-02	1.48E-02	MSH2/YY1/PARP3/IL2
BP	GO:0046622	positive regulation of organ growth	4/189	54/18862	2.09E-03	2.16E-02	1.48E-02	ACACB/ERBB4/NOTCH1/GLI1
BP	GO:0014033	neural crest cell differentiation	5/189	90/18862	2.12E-03	2.17E-02	1.48E-02	ERBB4/ANXA6/FN1/SEMA4D/SHH

BP	GO:0014068	positive regulation of phosphatidylinositol 3-kinase signaling	5/189	90/18862	2.12E-03	2.17E-02	1.48E-02	F2/IGF1R/ERBB4/FN1/SEMA4D
BP	GO:0090559	regulation of membrane permeability	5/189	90/18862	2.12E-03	2.17E-02	1.48E-02	HK2/YWHAQ/YWHAH/SFN/NMT1
BP	GO:0006165	nucleoside diphosphate phosphorylation	6/189	132/18862	2.16E-03	2.20E-02	1.51E-02	HK2/EP300/NME3/DTYMK/PGK1/IFNG
BP	GO:0019883	antigen processing and presentation of endogenous antigen	3/189	26/18862	2.17E-03	2.20E-02	1.51E-02	B2M/HLA-DRB1/CD74
BP	GO:0061082	myeloid leukocyte cytokine production	3/189	26/18862	2.17E-03	2.20E-02	1.51E-02	HMOX1/TLR4/CD74
BP	GO:0050810	regulation of steroid biosynthetic process	5/189	91/18862	2.22E-03	2.24E-02	1.54E-02	HMGCS1/CYP7A1/ACACB/IFNG/GGPS1
BP	GO:0045620	negative regulation of lymphocyte differentiation	4/189	55/18862	2.24E-03	2.24E-02	1.54E-02	HMGB3/CD74/IL2/SHH
BP	GO:0097345	mitochondrial outer membrane permeabilization	4/189	55/18862	2.24E-03	2.24E-02	1.54E-02	YWHAQ/YWHAH/SFN/NMT1
BP	GO:0002687	positive regulation of leukocyte migration	6/189	133/18862	2.24E-03	2.24E-02	1.54E-02	VEGFB/RAC1/PTK2B/DAPK2/OXSR1/CD74
BP	GO:0046939	nucleotide phosphorylation	6/189	133/18862	2.24E-03	2.24E-02	1.54E-02	HK2/EP300/NME3/DTYMK/PGK1/IFNG
BP	GO:0051402	neuron apoptotic process	8/189	230/18862	2.28E-03	2.28E-02	1.56E-02	HMOX1/PTK2B/HSP90AB1/MSH2/FAS/NAE1/XIAP/G6PD
BP	GO:0055017	cardiac muscle tissue growth	5/189	92/18862	2.33E-03	2.32E-02	1.59E-02	ERBB4/NOTCH1/YY1/G6PD/GLI1
BP	GO:0010883	regulation of lipid storage	4/189	56/18862	2.39E-03	2.37E-02	1.62E-02	ACACB/ITGB3/PPARG/NR1H3
BP	GO:0003401	axis elongation	3/189	27/18862	2.43E-03	2.37E-02	1.62E-02	ESR1/MAGI2/SHH
BP	GO:0035116	embryonic hindlimb morphogenesis	3/189	27/18862	2.43E-03	2.37E-02	1.62E-02	RARG/NOTCH1/SHH

BP	GO:0045606	positive regulation of epidermal cell differentiation	3/189	27/18862	2.43E-03	2.37E-02	1.62E-02	NCOA3/NOTCH1/SFN
BP	GO:0071624	positive regulation of granulocyte chemotaxis	3/189	27/18862	2.43E-03	2.37E-02	1.62E-02	RAC1/DAPK2/CD74
BP	GO:0043123	positive regulation of I-kappaB kinase/NF-kappaB signaling	7/189	182/18862	2.43E-03	2.37E-02	1.62E-02	HMOX1/MAP3K3/TRIM21/TLR4/HLA-DRB1/PRKCB/CD74
BP	GO:0006869	lipid transport	12/189	461/18862	2.44E-03	2.37E-02	1.62E-02	FABP3/PSAP/CYP7A1/ACACB/ITGB3/PPARG/SERPINA5/CHKA/NPC1L1/ABCC4/NR1H3/SHH
BP	GO:0002042	cell migration involved in sprouting angiogenesis	5/189	93/18862	2.44E-03	2.37E-02	1.62E-02	HMOX1/MAP3K3/HDAC7/NOTCH1/STARD13
BP	GO:0002367	cytokine production involved in immune response	5/189	93/18862	2.44E-03	2.37E-02	1.62E-02	HMOX1/BTK/B2M/TLR4/CD74
BP	GO:0014013	regulation of gliogenesis	5/189	93/18862	2.44E-03	2.37E-02	1.62E-02	F2/NF2/PPARG/NOTCH1/SHH
BP	GO:0001503	ossification	11/189	401/18862	2.46E-03	2.38E-02	1.63E-02	HSD17B4/MMP2/PTK2B/HDAC7/ACVR2B/BMP1/PPARG/NOTCH1/SEMA4D/GLI1/SHH
BP	GO:1903039	positive regulation of leukocyte cell-cell adhesion	8/189	234/18862	2.54E-03	2.45E-02	1.68E-02	RAC1/HLA-DRB1/RARA/IFNG/DPP4/CD74/IL2/SHH
BP	GO:0003158	endothelium development	6/189	137/18862	2.60E-03	2.50E-02	1.72E-02	ALOX12/ACVR2B/MSN/NOTCH1/TJP1/STARD13
BP	GO:0045861	negative regulation of proteolysis	10/189	346/18862	2.64E-03	2.53E-02	1.74E-02	F2/TRIM21/HSP90AB1/SERPINA5/XIAP/FHIT/SFN/DHCR24/SERPINA6/SHH
BP	GO:0038061	NIK/NF-kappaB signaling	7/189	185/18862	2.67E-03	2.55E-02	1.75E-02	CUL1/EP300/HDAC7/SKP1/TLR4/ALK/BTRC
BP	GO:0001889	liver development	6/189	138/18862	2.69E-03	2.55E-02	1.75E-02	HMGCS1/HMOX1/NOTCH1/ZIC3/ARF6/GLI1
BP	GO:0010039	response to iron ion	3/189	28/18862	2.70E-03	2.55E-02	1.75E-02	HMOX1/B2M/G6PD
BP	GO:0060603	mammary gland duct morphogenesis	3/189	28/18862	2.70E-03	2.55E-02	1.75E-02	AR/ESR1/BTRC
BP	GO:0072376	protein activation cascade	3/189	28/18862	2.70E-03	2.55E-02	1.75E-02	F2/F3/FN1

BP	GO:0072378	blood coagulation, fibrin clot formation	3/189	28/18862	2.70E-03	2.55E-02	1.75E-02	F2/F3/FN1
BP	GO:1903749	positive regulation of establishment of protein localization to mitochondrion	4/189	58/18862	2.72E-03	2.56E-02	1.76E-02	YWHAQ/YWHAH/SFN/NMT1
BP	GO:0001666	response to hypoxia	10/189	348/18862	2.75E-03	2.58E-02	1.77E-02	HK2/EP300/MMP2/HMOX1/VEGFB/PTK2B/PGK1/NOTCH1/ANG/DPP4
BP	GO:0050767	regulation of neurogenesis	10/189	348/18862	2.75E-03	2.58E-02	1.77E-02	F2/NF2/B2M/PPARG/NOTCH1/IFNG/YWHAH/FN1/SEMA4D/SHH
BP	GO:0042100	B cell proliferation	5/189	96/18862	2.80E-03	2.62E-02	1.80E-02	VAV3/BTK/TLR4/CD74/IL2
BP	GO:0048661	positive regulation of smooth muscle cell proliferation	5/189	96/18862	2.80E-03	2.62E-02	1.80E-02	MMP2/HBEGF/HMOX1/ALOX12/ABCC4
BP	GO:0043030	regulation of macrophage activation	4/189	59/18862	2.89E-03	2.68E-02	1.84E-02	BPI/TLR4/CD74/NR1H3
BP	GO:0046718	viral entry into host cell	6/189	140/18862	2.89E-03	2.68E-02	1.84E-02	TRIM21/WWP1/ITGB3/HLA-DRB1/DPP4/CD74
BP	GO:0061008	hepaticobiliary system development	6/189	140/18862	2.89E-03	2.68E-02	1.84E-02	HMGCS1/HMOX1/NOTCH1/ZIC3/ARF6/GLI1
BP	GO:0050690	regulation of defense response to virus by virus	3/189	29/18862	2.99E-03	2.75E-02	1.89E-02	RAC1/HCK/B2M
BP	GO:1902624	positive regulation of neutrophil migration	3/189	29/18862	2.99E-03	2.75E-02	1.89E-02	RAC1/DAPK2/CD74
BP	GO:0060759	regulation of response to cytokine stimulus	7/189	189/18862	3.00E-03	2.76E-02	1.89E-02	HSP90AB1/TLR4/IFNGR1/PPARG/IFNG/CD74/NR1H3
BP	GO:0032722	positive regulation of chemokine production	4/189	60/18862	3.07E-03	2.81E-02	1.93E-02	HMOX1/TLR4/IFNG/CD74
BP	GO:1902110	positive regulation of mitochondrial membrane permeability involved in apoptotic process	4/189	60/18862	3.07E-03	2.81E-02	1.93E-02	YWHAQ/YWHAH/SFN/NMT1

BP	GO:1904356	regulation of telomere maintenance via telomere lengthening	4/189	60/18862	3.07E-03	2.81E-02	1.93E-02	POT1/SMG5/HNRNPA2B1/PARN
BP	GO:1901605	alpha-amino acid metabolic process	7/189	191/18862	3.18E-03	2.90E-02	1.99E-02	MTAP/HPD/PAH/GAD2/ODC1/GART/OAT
BP	GO:0002702	positive regulation of production of molecular mediator of immune response	5/189	99/18862	3.20E-03	2.91E-02	1.99E-02	MSH2/B2M/TLR4/CD74/IL2
BP	GO:0002824	positive regulation of adaptive immune response based on somatic recombination of immune receptors built from immunoglobulin superfamily domains	5/189	99/18862	3.20E-03	2.91E-02	1.99E-02	BTK/MSH2/B2M/HLA-DRB1/IL2
BP	GO:0048754	branching morphogenesis of an epithelial tube	6/189	143/18862	3.21E-03	2.91E-02	1.99E-02	AR/ESR1/NOTCH1/BTRC/STK4/SHH
BP	GO:0046824	positive regulation of nucleocytoplasmic transport	4/189	61/18862	3.27E-03	2.94E-02	2.02E-02	HSP90AB1/IFNG/SFN/SHH
BP	GO:1905330	regulation of morphogenesis of an epithelium	4/189	61/18862	3.27E-03	2.94E-02	2.02E-02	AR/ESR1/ALOX12/SHH
BP	GO:0006221	pyrimidine nucleotide biosynthetic process	3/189	30/18862	3.29E-03	2.94E-02	2.02E-02	NME3/DTYMK/DCTD
BP	GO:0045577	regulation of B cell differentiation	3/189	30/18862	3.29E-03	2.94E-02	2.02E-02	BTK/HMGB3/IL2
BP	GO:0060045	positive regulation of cardiac muscle cell proliferation	3/189	30/18862	3.29E-03	2.94E-02	2.02E-02	ERBB4/NOTCH1/GLI1
BP	GO:0030900	forebrain development	10/189	357/18862	3.30E-03	2.94E-02	2.02E-02	NF2/RRM1/B2M/NSUN5/ERBB4/ALK/NOTCH1/GART/GLI1/SHH
BP	GO:0007044	cell-substrate junction assembly	5/189	100/18862	3.35E-03	2.98E-02	2.04E-02	RAC1/EPHA3/PTK2B/BCR/FN1
BP	GO:0009755	hormone-mediated signaling pathway	7/189	193/18862	3.37E-03	2.99E-02	2.05E-02	RARG/EP300/AR/ESR1/PPARG/RARA/YWHAH

BP	GO:0031589	cell-substrate adhesion	10/189	359/18862	3.43E-03	3.04E-02	2.08E-02	NF2/RAC1/EPHA3/PTK2B/BCR/ATXN3/ITGB3/NOTCH1/STK4/FN1
BP	GO:0045453	bone resorption	4/189	62/18862	3.46E-03	3.04E-02	2.09E-02	RAC1/PTK2B/ITGB3/PDK4
BP	GO:0070527	platelet aggregation	4/189	62/18862	3.46E-03	3.04E-02	2.09E-02	HBB/ALOX12/ITGB3/FN1
BP	GO:1902686	mitochondrial outer membrane permeabilization involved in programmed cell death	4/189	62/18862	3.46E-03	3.04E-02	2.09E-02	YWHAQ/YWHAH/SFN/NMT1
BP	GO:0046634	regulation of alpha-beta T cell activation	5/189	101/18862	3.49E-03	3.06E-02	2.09E-02	HLA-DRB1/RARA/IFNG/IL2/SHH
BP	GO:1901214	regulation of neuron death	9/189	302/18862	3.49E-03	3.06E-02	2.09E-02	HMOX1/PTK2B/HSP90AB1/MSH2/TLR4/NAE1/IFNG/G6PD/DHCR24
BP	GO:0036293	response to decreased oxygen levels	10/189	360/18862	3.50E-03	3.06E-02	2.09E-02	HK2/EP300/MMP2/HMOX1/VEGFB/PTK2B/PGK1/NOTCH1/ANG/DPP4
BP	GO:0051896	regulation of protein kinase B signaling	8/189	247/18862	3.53E-03	3.07E-02	2.10E-02	HBEGF/ESR1/RAC1/IGF1R/HSP90AB1/F3/ERBB4/MAGI2
BP	GO:0045580	regulation of T cell differentiation	6/189	146/18862	3.56E-03	3.09E-02	2.12E-02	HLA-DRB1/RARA/IFNG/CD74/IL2/SHH
BP	GO:1902806	regulation of cell cycle G1/S phase transition	7/189	195/18862	3.57E-03	3.09E-02	2.12E-02	EP300/MUC1/SENP2/WEE1/APEX1/SFN/GLI1
BP	GO:0033157	regulation of intracellular protein transport	8/189	248/18862	3.61E-03	3.12E-02	2.14E-02	HSP90AB1/YWHAQ/IFNG/B3GAT3/YWHAH/SFN/NMT1/SHH
BP	GO:0034390	smooth muscle cell apoptotic process	3/189	31/18862	3.62E-03	3.12E-02	2.14E-02	PPARG/IFNG/STK4
BP	GO:0034391	regulation of smooth muscle cell apoptotic process	3/189	31/18862	3.62E-03	3.12E-02	2.14E-02	PPARG/IFNG/STK4
BP	GO:0071887	leukocyte apoptotic process	5/189	102/18862	3.64E-03	3.13E-02	2.15E-02	BTK/DFFA/FAS/CD74/IL2
BP	GO:0048662	negative regulation of smooth muscle cell proliferation	4/189	63/18862	3.67E-03	3.13E-02	2.15E-02	HMOX1/PPARG/IFNG/ANG

BP	GO:0061180	mammary gland epithelium development	4/189	63/18862	3.67E-03	3.13E-02	2.15E-02	AR/ESR1/ERBB4/BTRC
BP	GO:0043393	regulation of protein binding	7/189	196/18862	3.67E-03	3.13E-02	2.15E-02	EP300/PTPRF/TRIM21/HSP90AB1/SENP2/B2M/STK4
BP	GO:0006352	DNA-templated transcription, initiation	8/189	249/18862	3.70E-03	3.15E-02	2.16E-02	RARG/AR/ESR1/PPARG/NOTCH1/RARA/TAF13/NR1H3
BP	GO:0019318	hexose metabolic process	8/189	250/18862	3.79E-03	3.20E-02	2.20E-02	HK2/EP300/DCXR/PDK2/ACACB/PGK1/G6PD/PDK4
BP	GO:1901991	negative regulation of mitotic cell cycle phase transition	8/189	250/18862	3.79E-03	3.20E-02	2.20E-02	CUL1/EP300/MUC1/SKP1/WEE1/NAE1/SFN/NDC80
BP	GO:0030593	neutrophil chemotaxis	5/189	103/18862	3.80E-03	3.20E-02	2.20E-02	VAV3/RAC1/DAPK2/DPP4/CD74
BP	GO:0032526	response to retinoic acid	5/189	103/18862	3.80E-03	3.20E-02	2.20E-02	RARG/EPHA3/PTK2B/PPARG/RARA
BP	GO:0090092	regulation of transmembrane receptor protein serine/threonine kinase signaling pathway	8/189	251/18862	3.88E-03	3.23E-02	2.21E-02	EP300/HSP90AB1/ACVR2B/MAGI2/PPARG/NOTCH1/XIAP/SHH
BP	GO:0002562	somatic diversification of immune receptors via germline recombination within a single locus	4/189	64/18862	3.88E-03	3.23E-02	2.21E-02	MSH2/YY1/PARP3/IL2
BP	GO:0016444	somatic cell DNA recombination	4/189	64/18862	3.88E-03	3.23E-02	2.21E-02	MSH2/YY1/PARP3/IL2
BP	GO:0016445	somatic diversification of immunoglobulins	4/189	64/18862	3.88E-03	3.23E-02	2.21E-02	MSH2/YY1/PARP3/IL2
BP	GO:0035794	positive regulation of mitochondrial membrane permeability	4/189	64/18862	3.88E-03	3.23E-02	2.21E-02	YWHAQ/YWHAH/SFN/NMT1
BP	GO:0071300	cellular response to retinoic acid	4/189	64/18862	3.88E-03	3.23E-02	2.21E-02	RARG/EPHA3/PTK2B/PPARG
BP	GO:0007389	pattern specification process	11/189	426/18862	3.89E-03	3.23E-02	2.21E-02	RARG/EP300/AR/ACVR2B/SENP2/ERBB4/NOTCH1/ZIC3/YY1/GLI1/SHH

BP	GO:0002821	positive regulation of adaptive immune response	5/189	104/18862	3.96E-03	3.23E-02	2.22E-02	BTK/MSH2/B2M/HLA-DRB1/IL2
BP	GO:0010661	positive regulation of muscle cell apoptotic process	3/189	32/18862	3.97E-03	3.23E-02	2.22E-02	PPARG/IFNG/STK4
BP	GO:0010743	regulation of macrophage derived foam cell differentiation	3/189	32/18862	3.97E-03	3.23E-02	2.22E-02	ITGB3/PPARG/NR1H3
BP	GO:0038128	ERBB2 signaling pathway	3/189	32/18862	3.97E-03	3.23E-02	2.22E-02	HBEGF/MATK/ERBB4
BP	GO:0043372	positive regulation of CD4-positive, alpha-beta T cell differentiation	3/189	32/18862	3.97E-03	3.23E-02	2.22E-02	HLA-DRB1/RARA/IFNG
BP	GO:0045684	positive regulation of epidermis development	3/189	32/18862	3.97E-03	3.23E-02	2.22E-02	NCOA3/NOTCH1/SFN
BP	GO:0048384	retinoic acid receptor signaling pathway	3/189	32/18862	3.97E-03	3.23E-02	2.22E-02	RARG/PPARG/RARA
BP	GO:1903902	positive regulation of viral life cycle	3/189	32/18862	3.97E-03	3.23E-02	2.22E-02	TRIM21/HLA-DRB1/CD74
BP	GO:0002791	regulation of peptide secretion	9/189	308/18862	3.97E-03	3.23E-02	2.22E-02	RAC1/TLR4/HLA-DRB1/IFNG/ANG/DPP4/ARF6/CD74/NR1H3
BP	GO:0045862	positive regulation of proteolysis	10/189	367/18862	4.01E-03	3.26E-02	2.23E-02	PTK2B/ALOX12/F3/SART3/FAS/ATXN3/PPARG/IFNG/BTRC/FN1
BP	GO:0006090	pyruvate metabolic process	6/189	150/18862	4.06E-03	3.29E-02	2.25E-02	HK2/EP300/PDK2/PGK1/IFNG/PDK4
BP	GO:0050871	positive regulation of B cell activation	6/189	150/18862	4.06E-03	3.29E-02	2.25E-02	VAV3/BTK/MSH2/TLR4/CD74/IL2
BP	GO:0070265	necrotic cell death	4/189	65/18862	4.11E-03	3.31E-02	2.27E-02	PYGL/ITPK1/TLR4/FAS
BP	GO:2000573	positive regulation of DNA biosynthetic process	4/189	65/18862	4.11E-03	3.31E-02	2.27E-02	POT1/PTK2B/HSP90AB1/PARN
BP	GO:0006637	acyl-CoA metabolic process	5/189	105/18862	4.12E-03	3.31E-02	2.27E-02	HSD17B4/HMGCS1/PDK2/ACACB/PDK4

BP	GO:0035383	thioester metabolic process	5/189	105/18862	4.12E-03	3.31E-02	2.27E-02	HSD17B4/HMGCS1/PDK2/ACACB/PDK4
BP	GO:0032535	regulation of cellular component size	10/189	370/18862	4.24E-03	3.34E-02	2.29E-02	RARG/VAV3/RAC1/HCK/PTK2B/HSP90AB1/MSN/ARF6/FN1/SEMA4D
BP	GO:0010887	negative regulation of cholesterol storage	2/189	10/18862	4.26E-03	3.34E-02	2.29E-02	PPARG/NR1H3
BP	GO:0060513	prostatic bud formation	2/189	10/18862	4.26E-03	3.34E-02	2.29E-02	AR/SHH
BP	GO:0060525	prostate glandular acinus development	2/189	10/18862	4.26E-03	3.34E-02	2.29E-02	ESR1/NOTCH1
BP	GO:1905461	positive regulation of vascular associated smooth muscle cell apoptotic process	2/189	10/18862	4.26E-03	3.34E-02	2.29E-02	PPARG/STK4
BP	GO:2000343	positive regulation of chemokine (C-X-C motif) ligand 2 production	2/189	10/18862	4.26E-03	3.34E-02	2.29E-02	TLR4/CD74
BP	GO:0007229	integrin-mediated signaling pathway	5/189	106/18862	4.29E-03	3.34E-02	2.29E-02	VAV3/HCK/PTK2B/ITGB3/FN1
BP	GO:0009062	fatty acid catabolic process	5/189	106/18862	4.29E-03	3.34E-02	2.29E-02	HSD17B4/ACADVL/ACACB/ECI2/ECHS1
BP	GO:0018958	phenol-containing compound metabolic process	5/189	106/18862	4.29E-03	3.34E-02	2.29E-02	ZEB2/PAH/SULT1A1/CYP2E1/PNMT
BP	GO:0044774	mitotic DNA integrity checkpoint	5/189	106/18862	4.29E-03	3.34E-02	2.29E-02	EP300/MUC1/MSH2/NAE1/SFN
BP	GO:0048259	regulation of receptor-mediated endocytosis	5/189	106/18862	4.29E-03	3.34E-02	2.29E-02	RAC1/B2M/ITGB3/MAGI2/ARF6
BP	GO:0010922	positive regulation of phosphatase activity	3/189	33/18862	4.33E-03	3.34E-02	2.29E-02	HSP90AB1/MAGI2/IFNG
BP	GO:0045589	regulation of regulatory T cell differentiation	3/189	33/18862	4.33E-03	3.34E-02	2.29E-02	HLA-DRB1/IFNG/IL2
BP	GO:0050869	negative regulation of B cell activation	3/189	33/18862	4.33E-03	3.34E-02	2.29E-02	BTK/HMGB3/PARP3

BP	GO:0051973	positive regulation of telomerase activity	3/189	33/18862	4.33E-03	3.34E-02	2.29E-02	POT1/HSP90AB1/PARN
BP	GO:0055094	response to lipoprotein particle	3/189	33/18862	4.33E-03	3.34E-02	2.29E-02	HMGCS1/TLR4/PPARG
BP	GO:0002294	CD4-positive, alpha-beta T cell differentiation involved in immune response	4/189	66/18862	4.34E-03	3.34E-02	2.29E-02	HLA-DRB1/RARA/IFNG/IL2
BP	GO:0030193	regulation of blood coagulation	4/189	66/18862	4.34E-03	3.34E-02	2.29E-02	F2/ALOX12/F3/FAP
BP	GO:0046635	positive regulation of alpha-beta T cell activation	4/189	66/18862	4.34E-03	3.34E-02	2.29E-02	HLA-DRB1/RARA/IFNG/SHH
BP	GO:1902108	regulation of mitochondrial membrane permeability involved in apoptotic process	4/189	66/18862	4.34E-03	3.34E-02	2.29E-02	YWHAQ/YWHAH/SFN/NMT1
BP	GO:0009952	anterior/posterior pattern specification	7/189	203/18862	4.44E-03	3.41E-02	2.34E-02	RARG/EP300/ACVR2B/NOTCH1/ZIC3/YY1/SHH
BP	GO:0002526	acute inflammatory response	5/189	107/18862	4.47E-03	3.41E-02	2.34E-02	F2/BTK/F3/PPARG/FN1
BP	GO:0046632	alpha-beta T cell differentiation	5/189	107/18862	4.47E-03	3.41E-02	2.34E-02	HLA-DRB1/RARA/IFNG/IL2/SHH
BP	GO:0044409	entry into host	6/189	153/18862	4.48E-03	3.41E-02	2.34E-02	TRIM21/WWP1/ITGB3/HLA-DRB1/DPP4/CD74
BP	GO:0002287	alpha-beta T cell activation involved in immune response	4/189	67/18862	4.58E-03	3.44E-02	2.36E-02	HLA-DRB1/RARA/IFNG/IL2
BP	GO:0002293	alpha-beta T cell differentiation involved in immune response	4/189	67/18862	4.58E-03	3.44E-02	2.36E-02	HLA-DRB1/RARA/IFNG/IL2
BP	GO:0033866	nucleoside bisphosphate biosynthetic process	4/189	67/18862	4.58E-03	3.44E-02	2.36E-02	PAPSS1/PDK2/ACACB/PDK4
BP	GO:0034030	ribonucleoside bisphosphate biosynthetic process	4/189	67/18862	4.58E-03	3.44E-02	2.36E-02	PAPSS1/PDK2/ACACB/PDK4
BP	GO:0034033	purine nucleoside bisphosphate biosynthetic process	4/189	67/18862	4.58E-03	3.44E-02	2.36E-02	PAPSS1/PDK2/ACACB/PDK4

BP	GO:1900046	regulation of hemostasis	4/189	67/18862	4.58E-03	3.44E-02	2.36E-02	F2/ALOX12/F3/FAP
BP	GO:1905953	negative regulation of lipid localization	4/189	67/18862	4.58E-03	3.44E-02	2.36E-02	ITGB3/PPARG/NR1H3/SHH
BP	GO:2000514	regulation of CD4-positive, alpha-beta T cell activation	4/189	67/18862	4.58E-03	3.44E-02	2.36E-02	HLA-DRB1/RARA/IFNG/IL2
BP	GO:0002446	neutrophil mediated immunity	12/189	499/18862	4.59E-03	3.44E-02	2.36E-02	F2/XRCC6/HBB/RAC1/FCGR2A/HSP90AB1/PYGL/TTR/BPI/B2M/PSAP/PA2G4
BP	GO:0001938	positive regulation of endothelial cell proliferation	5/189	108/18862	4.65E-03	3.44E-02	2.36E-02	HMOX1/VEGFB/F3/ITGB3/ANG
BP	GO:0002708	positive regulation of lymphocyte mediated immunity	5/189	108/18862	4.65E-03	3.44E-02	2.36E-02	BTK/MSH2/B2M/HLA-DRB1/IL2
BP	GO:0071156	regulation of cell cycle arrest	5/189	108/18862	4.65E-03	3.44E-02	2.36E-02	EP300/MUC1/HSP90AB1/FAP/SFN
BP	GO:0043281	regulation of cysteine-type endopeptidase activity involved in apoptotic process	7/189	205/18862	4.69E-03	3.44E-02	2.36E-02	ALOX12/F3/FAS/PPARG/XIAP/SFN/DHCR24
BP	GO:0001819	positive regulation of cytokine production	11/189	437/18862	4.70E-03	3.44E-02	2.36E-02	EP300/XRCC6/HMOX1/F3/B2M/TLR4/IFNGR1/RARA/IFNG/CD74/IL2
BP	GO:0009112	nucleobase metabolic process	3/189	34/18862	4.72E-03	3.44E-02	2.36E-02	RRM1/TTR/GART
BP	GO:0010543	regulation of platelet activation	3/189	34/18862	4.72E-03	3.44E-02	2.36E-02	F2/ALOX12/TLR4
BP	GO:0010614	negative regulation of cardiac muscle hypertrophy	3/189	34/18862	4.72E-03	3.44E-02	2.36E-02	NOTCH1/YY1/G6PD
BP	GO:0035137	hindlimb morphogenesis	3/189	34/18862	4.72E-03	3.44E-02	2.36E-02	RARG/NOTCH1/SHH
BP	GO:0043276	anoikis	3/189	34/18862	4.72E-03	3.44E-02	2.36E-02	DAPK2/NOTCH1/PDK4
BP	GO:0045922	negative regulation of fatty acid metabolic process	3/189	34/18862	4.72E-03	3.44E-02	2.36E-02	ACADVL/CYP7A1/ACACB

BP	GO:0048261	negative regulation of receptor-mediated endocytosis	3/189	34/18862	4.72E-03	3.44E-02	2.36E-02	RAC1/ITGB3/ARF6
BP	GO:0071402	cellular response to lipoprotein particle stimulus	3/189	34/18862	4.72E-03	3.44E-02	2.36E-02	HMGCS1/TLR4/PPARG
BP	GO:0090050	positive regulation of cell migration involved in sprouting angiogenesis	3/189	34/18862	4.72E-03	3.44E-02	2.36E-02	HMOX1/MAP3K3/HDAC7
BP	GO:0002822	regulation of adaptive immune response based on somatic recombination of immune receptors built from immunoglobulin superfamily domains	6/189	155/18862	4.77E-03	3.47E-02	2.38E-02	BTK/MSH2/B2M/HLA-DRB1/PARP3/IL2
BP	GO:0007004	telomere maintenance via telomerase	4/189	68/18862	4.83E-03	3.50E-02	2.40E-02	POT1/HSP90AB1/SMG5/PARN
BP	GO:0045685	regulation of glial cell differentiation	4/189	68/18862	4.83E-03	3.50E-02	2.40E-02	F2/PPARG/NOTCH1/SHH
BP	GO:0051403	stress-activated MAPK cascade	8/189	261/18862	4.90E-03	3.54E-02	2.43E-02	CUL1/MAP3K3/PTK2B/IGF1R/SKP1/TLR4/FAS/BTRC
BP	GO:0009132	nucleoside diphosphate metabolic process	6/189	156/18862	4.92E-03	3.55E-02	2.43E-02	HK2/EP300/NME3/DTYMK/PGK1/IFNG
BP	GO:0072522	purine-containing compound biosynthetic process	7/189	208/18862	5.07E-03	3.61E-02	2.47E-02	NME3/MTAP/PAPSS1/PDK2/ACACB/GART/PDK4
BP	GO:0002706	regulation of lymphocyte mediated immunity	6/189	157/18862	5.07E-03	3.61E-02	2.47E-02	BTK/MSH2/B2M/HLA-DRB1/PARP3/IL2
BP	GO:1905710	positive regulation of membrane permeability	4/189	69/18862	5.08E-03	3.61E-02	2.47E-02	YWHAQ/YWHAH/SFN/NMT1
BP	GO:0045191	regulation of isotype switching	3/189	35/18862	5.12E-03	3.61E-02	2.47E-02	MSH2/PARP3/IL2
BP	GO:0003157	endocardium development	2/189	11/18862	5.18E-03	3.61E-02	2.47E-02	NOTCH1/STK4

BP	GO:0006558	L-phenylalanine metabolic process	2/189	11/18862	5.18E-03	3.61E-02	2.47E-02	HPD/PAH
BP	GO:0006559	L-phenylalanine catabolic process	2/189	11/18862	5.18E-03	3.61E-02	2.47E-02	HPD/PAH
BP	GO:0006702	androgen biosynthetic process	2/189	11/18862	5.18E-03	3.61E-02	2.47E-02	CYP17A1/SRD5A2
BP	GO:0021936	regulation of cerebellar granule cell precursor proliferation	2/189	11/18862	5.18E-03	3.61E-02	2.47E-02	GLI1/SHH
BP	GO:0032957	inositol trisphosphate metabolic process	2/189	11/18862	5.18E-03	3.61E-02	2.47E-02	PTK2B/ITPK1
BP	GO:0033197	response to vitamin E	2/189	11/18862	5.18E-03	3.61E-02	2.47E-02	HMGCS1/PPARG
BP	GO:0045060	negative thymic T cell selection	2/189	11/18862	5.18E-03	3.61E-02	2.47E-02	CD74/SHH
BP	GO:0045348	positive regulation of MHC class II biosynthetic process	2/189	11/18862	5.18E-03	3.61E-02	2.47E-02	TLR4/IFNG
BP	GO:0045657	positive regulation of monocyte differentiation	2/189	11/18862	5.18E-03	3.61E-02	2.47E-02	HLA-DRB1/CD74
BP	GO:0071492	cellular response to UV-A	2/189	11/18862	5.18E-03	3.61E-02	2.47E-02	MMP2/MMP1
BP	GO:1900222	negative regulation of amyloid-beta clearance	2/189	11/18862	5.18E-03	3.61E-02	2.47E-02	IFNGR1/IFNG
BP	GO:1902221	erythrose 4-phosphate/phosphoenolpyruvate family amino acid metabolic process	2/189	11/18862	5.18E-03	3.61E-02	2.47E-02	HPD/PAH
BP	GO:1902222	erythrose 4-phosphate/phosphoenolpyruvate family amino acid catabolic process	2/189	11/18862	5.18E-03	3.61E-02	2.47E-02	HPD/PAH
BP	GO:0000075	cell cycle checkpoint	7/189	209/18862	5.20E-03	3.61E-02	2.48E-02	EP300/MUC1/MSH2/WEE1/NAE1/SFN/NDC80

BP	GO:0051651	maintenance of location in cell	7/189	209/18862	5.20E-03	3.61E-02	2.48E-02	F2/HK2/PTK2B/SKP1/ANXA6/ATP7B/ALB
BP	GO:0002286	T cell activation involved in immune response	5/189	111/18862	5.22E-03	3.62E-02	2.48E-02	HLA-DRB1/RARA/IFNG/CD74/IL2
BP	GO:0044106	cellular amine metabolic process	6/189	158/18862	5.23E-03	3.62E-02	2.48E-02	DHPS/PAH/SULT1A1/ODC1/PNMT/CHKA
BP	GO:0035303	regulation of dephosphorylation	7/189	210/18862	5.34E-03	3.69E-02	2.53E-02	PPP2R1A/HSP90AB1/SMG5/MAGI2/IFNG/B3GAT3/SEMA4D
BP	GO:0006879	cellular iron ion homeostasis	4/189	70/18862	5.35E-03	3.69E-02	2.53E-02	CUL1/HMOX1/SKP1/IFNG
BP	GO:0051092	positive regulation of NF-kappaB transcription factor activity	6/189	159/18862	5.39E-03	3.71E-02	2.54E-02	AR/BTK/TRIM21/TLR4/ALK/PRKCB
BP	GO:0021782	glial cell development	5/189	112/18862	5.42E-03	3.72E-02	2.55E-02	NSUN5/TLR4/IFNGR1/IFNG/SHH
BP	GO:0014741	negative regulation of muscle hypertrophy	3/189	36/18862	5.54E-03	3.78E-02	2.59E-02	NOTCH1/YY1/G6PD
BP	GO:0030224	monocyte differentiation	3/189	36/18862	5.54E-03	3.78E-02	2.59E-02	HLA-DRB1/PPARG/CD74
BP	GO:0032435	negative regulation of proteasomal ubiquitin-dependent protein catabolic process	3/189	36/18862	5.54E-03	3.78E-02	2.59E-02	HSP90AB1/FHIT/SHH
BP	GO:0045066	regulatory T cell differentiation	3/189	36/18862	5.54E-03	3.78E-02	2.59E-02	HLA-DRB1/IFNG/IL2
BP	GO:1904358	positive regulation of telomere maintenance via telomere lengthening	3/189	36/18862	5.54E-03	3.78E-02	2.59E-02	POT1/HNRNPA2B1/PARN
BP	GO:0050818	regulation of coagulation	4/189	71/18862	5.63E-03	3.82E-02	2.62E-02	F2/ALOX12/F3/FAP
BP	GO:0021700	developmental maturation	8/189	268/18862	5.73E-03	3.88E-02	2.66E-02	MMP2/BTK/RND1/PTK2B/PPARG/ANG/G6PD/SEMA4D

BP	GO:1901988	negative regulation of cell cycle phase transition	8/189	268/18862	5.73E-03	3.88E-02	2.66E-02	CUL1/EP300/MUC1/SKP1/WEE1/NAE1/SFN/NDC80
BP	GO:0002223	stimulatory C-type lectin receptor signaling pathway	5/189	114/18862	5.83E-03	3.94E-02	2.70E-02	CUL1/EP300/MUC1/SKP1/BTRC
BP	GO:0050863	regulation of T cell activation	9/189	327/18862	5.84E-03	3.94E-02	2.70E-02	RAC1/HLA-DRB1/RARA/IFNG/TNFAIP8L2/DPP4/CD74/IL2/SHH
BP	GO:0014855	striated muscle cell proliferation	4/189	72/18862	5.91E-03	3.98E-02	2.73E-02	ERBB4/NOTCH1/GLI1/SHH
BP	GO:1903747	regulation of establishment of protein localization to mitochondrion	4/189	72/18862	5.91E-03	3.98E-02	2.73E-02	YWHAQ/YWHAH/SFN/NMT1
BP	GO:0046621	negative regulation of organ growth	3/189	37/18862	5.99E-03	4.01E-02	2.75E-02	YY1/STK4/G6PD
BP	GO:0048713	regulation of oligodendrocyte differentiation	3/189	37/18862	5.99E-03	4.01E-02	2.75E-02	PPARG/NOTCH1/SHH
BP	GO:0090218	positive regulation of lipid kinase activity	3/189	37/18862	5.99E-03	4.01E-02	2.75E-02	F2/VAV3/PTK2B
BP	GO:0002903	negative regulation of B cell apoptotic process	2/189	12/18862	6.17E-03	4.02E-02	2.76E-02	CD74/IL2
BP	GO:0006570	tyrosine metabolic process	2/189	12/18862	6.17E-03	4.02E-02	2.76E-02	HPD/PAH
BP	GO:0009265	2'-deoxyribonucleotide biosynthetic process	2/189	12/18862	6.17E-03	4.02E-02	2.76E-02	DTYMK/DCTD
BP	GO:0038110	interleukin-2-mediated signaling pathway	2/189	12/18862	6.17E-03	4.02E-02	2.76E-02	PTK2B/IL2
BP	GO:0043383	negative T cell selection	2/189	12/18862	6.17E-03	4.02E-02	2.76E-02	CD74/SHH
BP	GO:0046322	negative regulation of fatty acid oxidation	2/189	12/18862	6.17E-03	4.02E-02	2.76E-02	ACADVL/ACACB
BP	GO:0046385	deoxyribose phosphate biosynthetic process	2/189	12/18862	6.17E-03	4.02E-02	2.76E-02	DTYMK/DCTD

BP	GO:0051095	regulation of helicase activity	2/189	12/18862	6.17E-03	4.02E-02	2.76E-02	POT1/MSH2
BP	GO:0051549	positive regulation of keratinocyte migration	2/189	12/18862	6.17E-03	4.02E-02	2.76E-02	HBEGF/ARF6
BP	GO:0060442	branching involved in prostate gland morphogenesis	2/189	12/18862	6.17E-03	4.02E-02	2.76E-02	ESR1/SHH
BP	GO:0060601	lateral sprouting from an epithelium	2/189	12/18862	6.17E-03	4.02E-02	2.76E-02	AR/SHH
BP	GO:0061052	negative regulation of cell growth involved in cardiac muscle cell development	2/189	12/18862	6.17E-03	4.02E-02	2.76E-02	YY1/G6PD
BP	GO:0061430	bone trabecula morphogenesis	2/189	12/18862	6.17E-03	4.02E-02	2.76E-02	MMP2/SEMA4D
BP	GO:0071801	regulation of podosome assembly	2/189	12/18862	6.17E-03	4.02E-02	2.76E-02	HCK/MSN
BP	GO:0090085	regulation of protein deubiquitination	2/189	12/18862	6.17E-03	4.02E-02	2.76E-02	TRIM21/SART3
BP	GO:1903037	regulation of leukocyte cell-cell adhesion	9/189	330/18862	6.19E-03	4.02E-02	2.76E-02	RAC1/HLA-DRB1/RARA/IFNG/TNFAIP8L2/DPP4/CD74/IL2/SHH
BP	GO:0002292	T cell differentiation involved in immune response	4/189	73/18862	6.21E-03	4.02E-02	2.76E-02	HLA-DRB1/RARA/IFNG/IL2
BP	GO:0031100	animal organ regeneration	4/189	73/18862	6.21E-03	4.02E-02	2.76E-02	HMOX1/PPARG/NOTCH1/GLI1
BP	GO:0005996	monosaccharide metabolic process	8/189	272/18862	6.25E-03	4.03E-02	2.77E-02	HK2/EP300/DCXR/PDK2/ACACB/PGK1/G6PD/PDK4
BP	GO:1903034	regulation of response to wounding	6/189	164/18862	6.25E-03	4.03E-02	2.77E-02	F2/HBEGF/PTPRF/ALOX12/F3/FAP
BP	GO:0033044	regulation of chromosome organization	8/189	273/18862	6.38E-03	4.08E-02	2.80E-02	POT1/MUC1/SART3/SMG5/HNRNPA2B1/PARN/CTCF/NDC80
BP	GO:0043491	protein kinase B signaling	8/189	273/18862	6.38E-03	4.08E-02	2.80E-02	HBEGF/ESR1/RAC1/IGF1R/HSP90AB1/F3/ERBB4/MAGI2

BP	GO:0002714	positive regulation of B cell mediated immunity	3/189	38/18862	6.46E-03	4.08E-02	2.80E-02	BTK/MSH2/IL2
BP	GO:0002891	positive regulation of immunoglobulin mediated immune response	3/189	38/18862	6.46E-03	4.08E-02	2.80E-02	BTK/MSH2/IL2
BP	GO:0009163	nucleoside biosynthetic process	3/189	38/18862	6.46E-03	4.08E-02	2.80E-02	NME3/DTYMK/MTAP
BP	GO:0009595	detection of biotic stimulus	3/189	38/18862	6.46E-03	4.08E-02	2.80E-02	TLR4/FAP/HLA-DRB1
BP	GO:0042307	positive regulation of protein import into nucleus	3/189	38/18862	6.46E-03	4.08E-02	2.80E-02	HSP90AB1/IFNG/SHH
BP	GO:0042401	cellular biogenic amine biosynthetic process	3/189	38/18862	6.46E-03	4.08E-02	2.80E-02	PAH/ODC1/PNMT
BP	GO:0045923	positive regulation of fatty acid metabolic process	3/189	38/18862	6.46E-03	4.08E-02	2.80E-02	PPARG/CD74/NR1H3
BP	GO:0051154	negative regulation of striated muscle cell differentiation	3/189	38/18862	6.46E-03	4.08E-02	2.80E-02	NOTCH1/YY1/G6PD
BP	GO:0097242	amyloid-beta clearance	3/189	38/18862	6.46E-03	4.08E-02	2.80E-02	IGF1R/IFNGR1/IFNG
BP	GO:2000249	regulation of actin cytoskeleton reorganization	3/189	38/18862	6.46E-03	4.08E-02	2.80E-02	HCK/PTK2B/TJP1
BP	GO:0097529	myeloid leukocyte migration	7/189	218/18862	6.51E-03	4.11E-02	2.82E-02	VAV3/VEGFB/RAC1/PTK2B/DAPK2/DPP4/CD74
BP	GO:0048469	cell maturation	6/189	166/18862	6.62E-03	4.17E-02	2.86E-02	BTK/RND1/PTK2B/PPARG/ANG/G6PD
BP	GO:0045787	positive regulation of cell cycle	10/189	395/18862	6.64E-03	4.18E-02	2.87E-02	EP300/MUC1/TRIM21/HSP90AB1/FAP/RARA/APEX1/SFN/NDC80/GLI1
BP	GO:0002220	innate immune response activating cell surface receptor signaling pathway	5/189	118/18862	6.73E-03	4.22E-02	2.89E-02	CUL1/EP300/MUC1/SKP1/BTRC
BP	GO:0002761	regulation of myeloid leukocyte differentiation	5/189	118/18862	6.73E-03	4.22E-02	2.89E-02	TLR4/HLA-DRB1/RARA/IFNG/CD74

BP	GO:0046916	cellular transition metal ion homeostasis	5/189	118/18862	6.73E-03	4.22E-02	2.89E-02	CUL1/HMOX1/SKP1/ATP7B/IFNG
BP	GO:0002200	somatic diversification of immune receptors	4/189	75/18862	6.83E-03	4.25E-02	2.92E-02	MSH2/YY1/PARP3/IL2
BP	GO:0006278	RNA-dependent DNA biosynthetic process	4/189	75/18862	6.83E-03	4.25E-02	2.92E-02	POT1/HSP90AB1/SMG5/PARN
BP	GO:0043536	positive regulation of blood vessel endothelial cell migration	4/189	75/18862	6.83E-03	4.25E-02	2.92E-02	HMOX1/MAP3K3/HDAC7/ALOX12
BP	GO:0009309	amine biosynthetic process	3/189	39/18862	6.94E-03	4.29E-02	2.94E-02	PAH/ODC1/PNMT
BP	GO:0032660	regulation of interleukin-17 production	3/189	39/18862	6.94E-03	4.29E-02	2.94E-02	TLR4/IFNG/IL2
BP	GO:0045622	regulation of T-helper cell differentiation	3/189	39/18862	6.94E-03	4.29E-02	2.94E-02	HLA-DRB1/RARA/IL2
BP	GO:2000279	negative regulation of DNA biosynthetic process	3/189	39/18862	6.94E-03	4.29E-02	2.94E-02	POT1/PPARG/PARP3
BP	GO:2000516	positive regulation of CD4-positive, alpha-beta T cell activation	3/189	39/18862	6.94E-03	4.29E-02	2.94E-02	HLA-DRB1/RARA/IFNG
BP	GO:0050868	negative regulation of T cell activation	5/189	119/18862	6.97E-03	4.30E-02	2.95E-02	HLA-DRB1/TNFAIP8L2/CD74/IL2/SHH
BP	GO:0120254	NA	5/189	119/18862	6.97E-03	4.30E-02	2.95E-02	HSD17B4/ALOX12/CYP2E1/CYP17A1/SRD5A2
BP	GO:0050670	regulation of lymphocyte proliferation	7/189	221/18862	7.00E-03	4.30E-02	2.95E-02	VAV3/BTK/TLR4/HLA-DRB1/CD74/IL2/SHH
BP	GO:0006805	xenobiotic metabolic process	5/189	120/18862	7.22E-03	4.34E-02	2.98E-02	HSP90AB1/SULT1A1/CYP2E1/AOC3/ACY1
BP	GO:0034605	cellular response to heat	5/189	120/18862	7.22E-03	4.34E-02	2.98E-02	EP300/HMOX1/HSP90AB1/ATXN3/RPA3
BP	GO:0050708	regulation of protein secretion	8/189	279/18862	7.24E-03	4.34E-02	2.98E-02	RAC1/TLR4/HLA-DRB1/IFNG/ANG/DPP4/ARF6/NR1H3

BP	GO:0002551	mast cell chemotaxis	2/189	13/18862	7.24E-03	4.34E-02	2.98E-02	VEGFB/RAC1
BP	GO:0003161	cardiac conduction system development	2/189	13/18862	7.24E-03	4.34E-02	2.98E-02	NOTCH1/DSG2
BP	GO:0032354	response to follicle-stimulating hormone	2/189	13/18862	7.24E-03	4.34E-02	2.98E-02	HMGCS1/NOTCH1
BP	GO:0038183	bile acid signaling pathway	2/189	13/18862	7.24E-03	4.34E-02	2.98E-02	CYP7A1/ABCC4
BP	GO:0042762	regulation of sulfur metabolic process	2/189	13/18862	7.24E-03	4.34E-02	2.98E-02	PDK2/PDK4
BP	GO:0048302	regulation of isotype switching to IgG isotypes	2/189	13/18862	7.24E-03	4.34E-02	2.98E-02	MSH2/IL2
BP	GO:0060841	venous blood vessel development	2/189	13/18862	7.24E-03	4.34E-02	2.98E-02	ACVR2B/NOTCH1
BP	GO:0070424	regulation of nucleotide-binding oligomerization domain containing signaling pathway	2/189	13/18862	7.24E-03	4.34E-02	2.98E-02	TLR4/XIAP
BP	GO:0070486	leukocyte aggregation	2/189	13/18862	7.24E-03	4.34E-02	2.98E-02	MSN/SEMA4D
BP	GO:0071352	cellular response to interleukin-2	2/189	13/18862	7.24E-03	4.34E-02	2.98E-02	PTK2B/IL2
BP	GO:0071635	negative regulation of transforming growth factor beta production	2/189	13/18862	7.24E-03	4.34E-02	2.98E-02	HSP90AB1/FN1
BP	GO:1902947	regulation of tau-protein kinase activity	2/189	13/18862	7.24E-03	4.34E-02	2.98E-02	HSP90AB1/IFNG
BP	GO:1904668	positive regulation of ubiquitin protein ligase activity	2/189	13/18862	7.24E-03	4.34E-02	2.98E-02	SKP1/BTRC
BP	GO:0016064	immunoglobulin mediated immune response	7/189	223/18862	7.34E-03	4.38E-02	3.00E-02	BTK/MSH2/HLA-DRB1/C1R/PARP3/CD74/IL2
BP	GO:0032944	regulation of mononuclear cell proliferation	7/189	223/18862	7.34E-03	4.38E-02	3.00E-02	VAV3/BTK/TLR4/HLA-DRB1/CD74/IL2/SHH

BP	GO:0002819	regulation of adaptive immune response	6/189	170/18862	7.41E-03	4.39E-02	3.01E-02	BTK/MSH2/B2M/HLA-DRB1/PARP3/IL2
BP	GO:0048639	positive regulation of developmental growth	6/189	170/18862	7.41E-03	4.39E-02	3.01E-02	ACACB/ERBB4/NOTCH1/FN1/SEMA4D/GLI1
BP	GO:0009262	deoxyribonucleotide metabolic process	3/189	40/18862	7.45E-03	4.39E-02	3.01E-02	RRM1/DTYMK/DCTD
BP	GO:0055023	positive regulation of cardiac muscle tissue growth	3/189	40/18862	7.45E-03	4.39E-02	3.01E-02	ERBB4/NOTCH1/GLI1
BP	GO:0060443	mammary gland morphogenesis	3/189	40/18862	7.45E-03	4.39E-02	3.01E-02	AR/ESR1/BTRC
BP	GO:0072528	pyrimidine-containing compound biosynthetic process	3/189	40/18862	7.45E-03	4.39E-02	3.01E-02	NME3/DTYMK/DCTD
BP	GO:1904591	positive regulation of protein import	3/189	40/18862	7.45E-03	4.39E-02	3.01E-02	HSP90AB1/IFNG/SHH
BP	GO:2000008	regulation of protein localization to cell surface	3/189	40/18862	7.45E-03	4.39E-02	3.01E-02	HSP90AB1/ERBB4/ARF6
BP	GO:0016042	lipid catabolic process	9/189	340/18862	7.46E-03	4.39E-02	3.01E-02	HSD17B4/ACADVL/FABP3/CYP7A1/ACACB/ALK/ECI2/ECHS1/YWHAH
BP	GO:0051205	protein insertion into membrane	4/189	77/18862	7.48E-03	4.40E-02	3.02E-02	YWHAQ/YWHAH/SFN/NMT1
BP	GO:0001659	temperature homeostasis	6/189	171/18862	7.61E-03	4.47E-02	3.06E-02	ACADVL/IGF1R/ACVR2B/TLR4/NOTCH1/NR1H3
BP	GO:0046651	lymphocyte proliferation	8/189	282/18862	7.70E-03	4.51E-02	3.09E-02	VAV3/BTK/MSN/TLR4/HLA-DRB1/CD74/IL2/SHH
BP	GO:0019724	B cell mediated immunity	7/189	226/18862	7.87E-03	4.61E-02	3.16E-02	BTK/MSH2/HLA-DRB1/C1R/PARP3/CD74/IL2
BP	GO:0032689	negative regulation of interferon-gamma production	3/189	41/18862	7.98E-03	4.64E-02	3.18E-02	TLR4/HLA-DRB1/RARA
BP	GO:0033574	response to testosterone	3/189	41/18862	7.98E-03	4.64E-02	3.18E-02	AR/MTAP/MSN
BP	GO:0046596	regulation of viral entry into host cell	3/189	41/18862	7.98E-03	4.64E-02	3.18E-02	TRIM21/HLA-DRB1/CD74

BP	GO:0007498	mesoderm development	5/189	123/18862	7.99E-03	4.64E-02	3.18E-02	NF2/BTK/HCK/ITGB3/SHH
BP	GO:1990266	neutrophil migration	5/189	123/18862	7.99E-03	4.64E-02	3.18E-02	VAV3/RAC1/DAPK2/DPP4/CD74
BP	GO:0061138	morphogenesis of a branching epithelium	6/189	173/18862	8.04E-03	4.66E-02	3.20E-02	AR/ESR1/NOTCH1/BTRC/STK4/SHH
BP	GO:0032943	mononuclear cell proliferation	8/189	285/18862	8.18E-03	4.73E-02	3.24E-02	VAV3/BTK/MSN/TLR4/HLA-DRB1/CD74/IL2/SHH
BP	GO:0032204	regulation of telomere maintenance	4/189	79/18862	8.18E-03	4.73E-02	3.24E-02	POT1/SMG5/HNRNPA2B1/PARN
BP	GO:0009266	response to temperature stimulus	7/189	228/18862	8.24E-03	4.73E-02	3.24E-02	EP300/ACADVL/HMOX1/HSP90AB1/ATXN3/RPA3/PPARG
BP	GO:2000045	regulation of G1/S transition of mitotic cell cycle	6/189	174/18862	8.26E-03	4.73E-02	3.24E-02	EP300/MUC1/SENP2/WEE1/APEX1/SFN
BP	GO:0006721	terpenoid metabolic process	5/189	124/18862	8.26E-03	4.73E-02	3.24E-02	HMGCS1/TTR/ADH1C/CYP2E1/GGPS1
BP	GO:0051053	negative regulation of DNA metabolic process	5/189	124/18862	8.26E-03	4.73E-02	3.24E-02	POT1/DFFA/MSH2/PPARG/PARP3
BP	GO:0071621	granulocyte chemotaxis	5/189	124/18862	8.26E-03	4.73E-02	3.24E-02	VAV3/RAC1/DAPK2/DPP4/CD74
BP	GO:0003413	chondrocyte differentiation involved in endochondral bone morphogenesis	2/189	14/18862	8.40E-03	4.73E-02	3.24E-02	RARG/ANXA6
BP	GO:0010935	regulation of macrophage cytokine production	2/189	14/18862	8.40E-03	4.73E-02	3.24E-02	TLR4/CD74
BP	GO:0032966	negative regulation of collagen biosynthetic process	2/189	14/18862	8.40E-03	4.73E-02	3.24E-02	CYP7A1/PPARG
BP	GO:0036295	cellular response to increased oxygen levels	2/189	14/18862	8.40E-03	4.73E-02	3.24E-02	FAS/PPARG
BP	GO:0045059	positive thymic T cell selection	2/189	14/18862	8.40E-03	4.73E-02	3.24E-02	CD74/SHH

BP	GO:0048291	isotype switching to IgG isotypes	2/189	14/18862	8.40E-03	4.73E-02	3.24E-02	MSH2/IL2
BP	GO:0051547	regulation of keratinocyte migration	2/189	14/18862	8.40E-03	4.73E-02	3.24E-02	HBEGF/ARF6
BP	GO:0051917	regulation of fibrinolysis	2/189	14/18862	8.40E-03	4.73E-02	3.24E-02	F2/FAP
BP	GO:0070141	response to UV-A	2/189	14/18862	8.40E-03	4.73E-02	3.24E-02	MMP2/MMP1
BP	GO:0070669	response to interleukin-2	2/189	14/18862	8.40E-03	4.73E-02	3.24E-02	PTK2B/IL2
BP	GO:0097284	hepatocyte apoptotic process	2/189	14/18862	8.40E-03	4.73E-02	3.24E-02	STK4/ARF6
BP	GO:0097531	mast cell migration	2/189	14/18862	8.40E-03	4.73E-02	3.24E-02	VEGFB/RAC1
BP	GO:0042110	T cell activation	11/189	474/18862	8.45E-03	4.74E-02	3.25E-02	RAC1/MSN/B2M/HLA-DRB1/RARA/IFNG/TNFAIP8L2/DPP4/CD74/IL2/SHH
BP	GO:0009152	purine ribonucleotide biosynthetic process	6/189	175/18862	8.48E-03	4.74E-02	3.25E-02	NME3/PAPSS1/PDK2/ACACB/GART/PDK4
BP	GO:0052126	movement in host environment	6/189	175/18862	8.48E-03	4.74E-02	3.25E-02	TRIM21/WWP1/ITGB3/HLA-DRB1/DPP4/CD74
BP	GO:0014002	astrocyte development	3/189	42/18862	8.53E-03	4.74E-02	3.25E-02	TLR4/IFNGR1/IFNG
BP	GO:0031670	cellular response to nutrient	3/189	42/18862	8.53E-03	4.74E-02	3.25E-02	HMOX1/PDK2/PPARG
BP	GO:0032965	regulation of collagen biosynthetic process	3/189	42/18862	8.53E-03	4.74E-02	3.25E-02	F2/CYP7A1/PPARG
BP	GO:0045687	positive regulation of glial cell differentiation	3/189	42/18862	8.53E-03	4.74E-02	3.25E-02	PPARG/NOTCH1/SHH
BP	GO:1901659	glycosyl compound biosynthetic process	3/189	42/18862	8.53E-03	4.74E-02	3.25E-02	NME3/DTYMK/MTAP
BP	GO:0002705	positive regulation of leukocyte mediated immunity	5/189	125/18862	8.54E-03	4.74E-02	3.25E-02	BTK/MSH2/B2M/HLA-DRB1/IL2

BP	GO:0071466	cellular response to xenobiotic stimulus	5/189	125/18862	8.54E-03	4.74E-02	3.25E-02	HSP90AB1/SULT1A1/CYP2E1/AOC3/ACY1
BP	GO:0007160	cell-matrix adhesion	7/189	230/18862	8.62E-03	4.77E-02	3.27E-02	NF2/RAC1/EPHA3/PTK2B/BCR/ITGB3/FN1
BP	GO:2000116	regulation of cysteine-type endopeptidase activity	7/189	230/18862	8.62E-03	4.77E-02	3.27E-02	ALOX12/F3/FAS/PPARG/XIAP/SFN/DHCR24
BP	GO:0044262	cellular carbohydrate metabolic process	8/189	288/18862	8.68E-03	4.80E-02	3.29E-02	HK2/EP300/PTK2B/PYGL/PDK2/ITPK1/ACACB/PDK4
BP	GO:0010921	regulation of phosphatase activity	6/189	176/18862	8.71E-03	4.80E-02	3.29E-02	PPP2R1A/HSP90AB1/MAGI2/IFNG/B3GAT3/SEMA4D
BP	GO:0048762	mesenchymal cell differentiation	7/189	231/18862	8.82E-03	4.85E-02	3.33E-02	EPHA3/ERBB4/ANXA6/NOTCH1/FN1/SEMA4D/SHH
BP	GO:0045471	response to ethanol	5/189	126/18862	8.82E-03	4.85E-02	3.33E-02	PTK2B/CYP7A1/CYP2E1/G6PD/IL2
BP	GO:0043367	CD4-positive, alpha-beta T cell differentiation	4/189	81/18862	8.92E-03	4.88E-02	3.35E-02	HLA-DRB1/RARA/IFNG/IL2
BP	GO:0050672	negative regulation of lymphocyte proliferation	4/189	81/18862	8.92E-03	4.88E-02	3.35E-02	BTK/HLA-DRB1/IL2/SHH
BP	GO:0032386	regulation of intracellular transport	9/189	350/18862	8.93E-03	4.88E-02	3.35E-02	HSP90AB1/MSN/YWHAQ/IFNG/B3GAT3/YWHAH/SFN/NMT1/SHH
BP	GO:0001936	regulation of endothelial cell proliferation	6/189	177/18862	8.94E-03	4.88E-02	3.35E-02	HMOX1/VEGFB/F3/ITGB3/PPARG/ANG
BP	GO:0001959	regulation of cytokine-mediated signaling pathway	6/189	177/18862	8.94E-03	4.88E-02	3.35E-02	HSP90AB1/IFNGR1/PPARG/IFNG/CD74/NR1H3
BP	GO:0021762	substantia nigra development	3/189	43/18862	9.11E-03	4.93E-02	3.38E-02	YWHAQ/YWHAH/G6PD
BP	GO:0032620	interleukin-17 production	3/189	43/18862	9.11E-03	4.93E-02	3.38E-02	TLR4/IFNG/IL2
BP	GO:0046189	phenol-containing compound biosynthetic process	3/189	43/18862	9.11E-03	4.93E-02	3.38E-02	ZEB2/PAH/PNMT
BP	GO:0061383	trabecula morphogenesis	3/189	43/18862	9.11E-03	4.93E-02	3.38E-02	MMP2/NOTCH1/SEMA4D

BP	GO:2000273	positive regulation of signaling receptor activity	3/189	43/18862	9.11E-03	4.93E-02	3.38E-02	HBEGF/NCOA3/IFNG
BP	GO:0014066	regulation of phosphatidylinositol 3-kinase signaling	5/189	127/18862	9.11E-03	4.93E-02	3.38E-02	F2/IGF1R/ERBB4/FN1/SEMA4D
BP	GO:0097305	response to alcohol	7/189	233/18862	9.22E-03	4.98E-02	3.42E-02	HMGCS1/PTK2B/CYP7A1/CYP2E1/PPARG/G6PD/IL2
CC	GO:0045177	apical part of cell	14/191	414/19520	1.47E-05	4.89E-03	4.01E-03	NF2/MUC1/HSP90AB1/MSN/ITPK1/FAP/NOTCH1/TJP1/DPP4/DSG2/FN1/CLCN5/SLC5A1/NPC1L1
CC	GO:0005667	transcription factor complex	12/191	365/19520	2.41E-05	4.89E-03	4.01E-03	APEX1/EP300/XRCC6/HSP90AB1/NFATC1/POU2F1/PPARG/RARA/RARG/TAF13/YY1/NR1H3
CC	GO:0031091	platelet alpha granule	6/191	91/19520	2.71E-04	4.07E-02	3.34E-02	VEGFB/ITGB3/SERPINA5/ALB/THBS2/FN1
CC	GO:0072562	blood microparticle	7/191	146/19520	5.94E-04	4.07E-02	3.34E-02	F2/HBB/GC/MSN/ALB/C1R/FN1
CC	GO:0031527	filopodium membrane	3/191	18/19520	6.75E-04	4.07E-02	3.34E-02	NF2/ITGB3/ARF6
CC	GO:0016324	apical plasma membrane	11/191	351/19520	7.06E-04	4.07E-02	3.34E-02	MUC1/HSP90AB1/MSN/ITPK1/NOTCH1/DPP4/DSG2/FN1/SLC5A1/NPC1L1/ABCC4
CC	GO:0005925	focal adhesion	12/191	416/19520	8.43E-04	4.07E-02	3.34E-02	RAC1/HCK/PTK2B/FLNB/MSN/B2M/YWHAQ/ITGB3/FAP/ANXA6/DPP4/ARF6
CC	GO:0030055	cell-substrate junction	12/191	423/19520	9.73E-04	4.07E-02	3.34E-02	RAC1/HCK/PTK2B/FLNB/MSN/B2M/YWHAQ/ITGB3/FAP/ANXA6/DPP4/ARF6
CC	GO:0031258	lamellipodium membrane	3/191	22/19520	1.24E-03	4.07E-02	3.34E-02	ITGB3/FAP/DPP4
CC	GO:0034774	secretory granule lumen	10/191	322/19520	1.31E-03	4.07E-02	3.34E-02	XRCC6/VEGFB/HSP90AB1/PYGL/TTR/BPI/B2M/PA2G4/ALB/FN1
CC	GO:0060205	cytoplasmic vesicle lumen	10/191	326/19520	1.44E-03	4.07E-02	3.34E-02	XRCC6/VEGFB/HSP90AB1/PYGL/TTR/BPI/B2M/PA2G4/ALB/FN1
CC	GO:0031983	vesicle lumen	10/191	328/19520	1.51E-03	4.07E-02	3.34E-02	XRCC6/VEGFB/HSP90AB1/PYGL/TTR/BPI/B2M/PA2G4/ALB/FN1

CC	GO:0005775	vacuolar lumen	7/191	173/19520	1.60E-03	4.07E-02	3.34E-02	GC/TTR/BPI/PSAP/PA2G4/DAPK2/CD74
CC	GO:0001726	ruffle	7/191	175/19520	1.71E-03	4.07E-02	3.34E-02	NF2/RAC1/TLR4/ITGB3/FAP/S100A6/ARF6
CC	GO:0042611	MHC protein complex	3/191	25/19520	1.81E-03	4.07E-02	3.34E-02	B2M/HLA-DRB1/CD74
CC	GO:0031253	cell projection membrane	10/191	337/19520	1.84E-03	4.07E-02	3.34E-02	NF2/RAC1/HSP90AB1/MSN/ITGB3/FAP/DPP4/ARF6/SLC5A1/NPC1L1
MF	GO:0033293	monocarboxylic acid binding	8/191	71/18337	7.27E-07	4.13E-04	3.35E-04	FABP3/PYGL/PSAP/ACACB/PPARG/SERPINA5/RARA/ALB
MF	GO:0004713	protein tyrosine kinase activity	10/191	135/18337	1.52E-06	4.31E-04	3.49E-04	BTK/HCK/EPHA3/PTK2B/IGF1R/MATK/CLK1/WEE1/ERBB4/ALK
MF	GO:0043177	organic acid binding	8/191	114/18337	2.57E-05	1.83E-03	1.48E-03	HMGCS1/FABP3/HBB/ACACB/GAD2/PPARG/SERPINA5/RARA
MF	GO:0042162	telomeric DNA binding	5/191	36/18337	3.37E-05	2.13E-03	1.73E-03	POT1/XRCC6/SMG5/HNRNPA2B1/APEX1
MF	GO:0033218	amide binding	14/191	391/18337	6.34E-05	3.04E-03	2.46E-03	ACADVL/IGF1R/HSP90AB1/PSAP/TLR4/PEX5/ACACB/HLA-DRB1/NFATC1/PPARG/ANG/ECI2/CD74/DHCR24
MF	GO:0003684	damaged DNA binding	6/191	66/18337	6.38E-05	3.04E-03	2.46E-03	POT1/EP300/XRCC6/MSH2/RPA3/APEX1
MF	GO:0016922	nuclear receptor binding	7/191	97/18337	6.94E-05	3.04E-03	2.46E-03	RARG/EP300/ESR1/NCOA3/PPARG/PRKCB/YWHAH
MF	GO:0002020	protease binding	8/191	131/18337	6.94E-05	3.04E-03	2.46E-03	F3/SART3/PSAP/ITGB3/FAP/SERPINA5/DPP4/FN1
MF	GO:0005496	steroid binding	7/191	100/18337	8.43E-05	3.43E-03	2.78E-03	AR/ESR1/GC/ANXA6/SERPINA6/NR1H3/SHBG
MF	GO:0005536	glucose binding	3/191	10/18337	1.26E-04	4.79E-03	3.88E-03	HK2/PYGL/G6PD
MF	GO:0031406	carboxylic acid binding	9/191	184/18337	1.36E-04	4.79E-03	3.88E-03	FABP3/PYGL/PSAP/ACACB/GAD2/PPARG/SERPINA5/RARA/ALB
MF	GO:0005539	glycosaminoglycan binding	10/191	228/18337	1.43E-04	4.79E-03	3.88E-03	F2/HBEGF/VEGFB/PTPRF/ANXA6/SERPINA5/ANG/THBS2/FN1/SHH

MF	GO:0016616	oxidoreductase activity, acting on the CH-OH group of donors, NAD or NADP as acceptor	7/191	120/18337	2.63E-04	7.88E-03	6.38E-03	HSD17B4/DCXR/ADH1C/G6PD/GRHPR/ABCC4/SRD5A2
MF	GO:0008201	heparin binding	8/191	164/18337	3.27E-04	9.32E-03	7.55E-03	F2/HBEGF/VEGFB/PTPRF/SERPINA5/ANG/THBS2/FN1
MF	GO:0005154	epidermal growth factor receptor binding	4/191	33/18337	3.68E-04	9.96E-03	8.07E-03	HBEGF/VAV3/ERBB4/SNX1
MF	GO:0004252	serine-type endopeptidase activity	8/191	168/18337	3.85E-04	9.96E-03	8.07E-03	F2/MMP2/KLK1/F3/FAP/MMP1/DPP4/C1R
MF	GO:0097718	disordered domain specific binding	4/191	34/18337	4.14E-04	1.02E-02	8.23E-03	RRM1/HSP90AB1/NCOA3/FN1
MF	GO:0016614	oxidoreductase activity, acting on CH-OH group of donors	7/191	130/18337	4.28E-04	1.02E-02	8.23E-03	HSD17B4/DCXR/ADH1C/G6PD/GRHPR/ABCC4/SRD5A2
MF	GO:0000217	DNA secondary structure binding	4/191	36/18337	5.18E-04	1.18E-02	9.55E-03	POT1/MSH2/YY1/HMGB3
MF	GO:0061629	RNA polymerase II sequence-specific DNA-binding transcription factor binding	10/191	271/18337	5.67E-04	1.24E-02	1.01E-02	RARG/EP300/ESR1/GTF2I/NCOA3/EXOSC9/PPARG/APEX1/PRKCB/YWHAH
MF	GO:0035257	nuclear hormone receptor binding	7/191	140/18337	6.67E-04	1.29E-02	1.05E-02	RARG/EP300/ESR1/NCOA3/PPARG/PRKCB/YWHAH
MF	GO:0000400	four-way junction DNA binding	3/191	17/18337	6.79E-04	1.29E-02	1.05E-02	MSH2/YY1/HMGB3
MF	GO:0023026	MHC class II protein complex binding	3/191	17/18337	6.79E-04	1.29E-02	1.05E-02	HSP90AB1/HLA-DRB1/CD74
MF	GO:0070851	growth factor receptor binding	7/191	141/18337	6.96E-04	1.29E-02	1.05E-02	HBEGF/VAV3/VEGFB/ERBB4/ITGB3/SNX1/IL2
MF	GO:0019825	oxygen binding	4/191	39/18337	7.05E-04	1.29E-02	1.05E-02	HBB/CYP2E1/ALB/CYP17A1
MF	GO:0008022	protein C-terminus binding	8/191	185/18337	7.28E-04	1.29E-02	1.05E-02	EP300/XRCC6/PTK2B/MSH2/YWHAQ/PPARG/TAF13/FN1
MF	GO:0008236	serine-type peptidase activity	8/191	186/18337	7.54E-04	1.30E-02	1.05E-02	F2/MMP2/KLK1/F3/FAP/MMP1/DPP4/C1R

MF	GO:0017171	serine hydrolase activity	8/191	188/18337	8.09E-04	1.34E-02	1.08E-02	F2/MMP2/KLK1/F3/FAP/MMP1/DPP4/C1R
MF	GO:0019842	vitamin binding	7/191	145/18337	8.21E-04	1.34E-02	1.08E-02	RBP5/GC/PYGL/ACACB/GAD2/ALB/OAT
MF	GO:0035258	steroid hormone receptor binding	5/191	77/18337	1.25E-03	1.98E-02	1.61E-02	EP300/ESR1/PPARG/PRKCB/YWHAH
MF	GO:0033613	activating transcription factor binding	5/191	78/18337	1.33E-03	2.04E-02	1.65E-02	EP300/HDAC7/GTF2I/EXOSC9/PPARG
MF	GO:0019199	transmembrane receptor protein kinase activity	5/191	80/18337	1.49E-03	2.23E-02	1.80E-02	EPHA3/IGF1R/ACVR2B/ERBB4/ALK
MF	GO:0001224	RNA polymerase II transcription cofactor binding	3/191	24/18337	1.92E-03	2.77E-02	2.24E-02	AR/ESR1/NFATC1
MF	GO:0008013	beta-catenin binding	5/191	85/18337	1.95E-03	2.77E-02	2.24E-02	EP300/AR/ESR1/SKP1/BTRC
MF	GO:0051427	hormone receptor binding	7/191	173/18337	2.27E-03	3.15E-02	2.55E-02	RARG/EP300/ESR1/NCOA3/PPARG/PRKCB/YWHAH
MF	GO:0001223	transcription coactivator binding	3/191	26/18337	2.42E-03	3.21E-02	2.60E-02	AR/ESR1/NFATC1
MF	GO:0023023	MHC protein complex binding	3/191	26/18337	2.42E-03	3.21E-02	2.60E-02	HSP90AB1/HLA-DRB1/CD74
MF	GO:0030170	pyridoxal phosphate binding	4/191	55/18337	2.57E-03	3.25E-02	2.63E-02	PYGL/GAD2/ALB/OAT
MF	GO:0070279	vitamin B6 binding	4/191	55/18337	2.57E-03	3.25E-02	2.63E-02	PYGL/GAD2/ALB/OAT
MF	GO:0005507	copper ion binding	4/191	60/18337	3.53E-03	4.37E-02	3.54E-02	ATP7B/AOC3/ANG/ALB
MF	GO:0004714	transmembrane receptor protein tyrosine kinase activity	4/191	61/18337	3.75E-03	4.54E-02	3.68E-02	EPHA3/IGF1R/ERBB4/ALK
MF	GO:0001530	lipopolysaccharide binding	3/191	32/18337	4.42E-03	4.84E-02	3.92E-02	F2/BPI/TLR4
MF	GO:0031490	chromatin DNA binding	5/191	103/18337	4.47E-03	4.84E-02	3.92E-02	EP300/NOTCH1/RARA/CTCF/APEX1

MF	GO:0004300	enoyl-CoA hydratase activity	2/191	10/18337	4.60E-03	4.84E-02	3.92E-02	HSD17B4/ECHS1
MF	GO:0008239	dipeptidyl-peptidase activity	2/191	10/18337	4.60E-03	4.84E-02	3.92E-02	FAP/DPP4
MF	GO:0032356	oxidized DNA binding	2/191	10/18337	4.60E-03	4.84E-02	3.92E-02	POT1/MSH2
MF	GO:0043208	glycosphingolipid binding	2/191	10/18337	4.60E-03	4.84E-02	3.92E-02	PSAP/IL2
MF	GO:0098505	G-rich strand telomeric DNA binding	2/191	10/18337	4.60E-03	4.84E-02	3.92E-02	POT1/HNRNPA2B1

Note： KEGG, Kyoto Encyclopedia of Genes and Genomes; GO,Gene Ontology; BC, Bladder Carcinoma; BP, biological process; CC, cellular component; MF, molecular function

Supplementary Table S9: Binding free energy during 200ns molecular docking simulation of NRP1 docking with solasonine.

Binding free energy during 200ns MDS of NRP1 docking with solasonine.					
#Frame	Residue number	MMPBSA (kcal/mol)	MM (kcal/mol)	MM_VDW (kcal/mol)	MM_COU (kcal/mol)
P~1PHE	273	0.226	0.226	0	0.227
P~2LYS	274	0.197	0.197	0	0.197
P~3CYS	275	-0.013	-0.013	0	-0.013
P~4MET	276	0.004	0.004	0	0.005
P~5GLU	277	-0.204	-0.204	0	-0.203
P~6ALA	278	0.016	0.016	0	0.017
P~7LEU	279	0.017	0.017	-0.001	0.018
P~8GLY	280	0.016	0.016	0	0.017
P~9MET	281	0.039	0.039	-0.003	0.043
P~10GLU	282	-0.244	-0.244	-0.002	-0.242
P~11SER	283	0.005	0.005	-0.001	0.006
P~12GLY	284	0.018	0.018	-0.001	0.018
P~13GLU	285	-0.155	-0.155	-0.001	-0.154
P~14ILE	286	0.003	0.003	-0.002	0.005
P~15HIS	287	-0.051	-0.051	-0.002	-0.05
P~16SER	288	-0.06	-0.06	-0.002	-0.057
P~17ASP	289	-0.02	-0.02	-0.002	-0.019
P~18GLN	290	0.002	0.002	-0.002	0.004
P~19ILE	291	0.034	0.034	-0.006	0.039
P~20THR	292	-0.038	-0.038	-0.006	-0.031
P~21ALA	293	0.103	0.103	-0.015	0.118
P~22SER	294	0.044	0.044	-0.028	0.072
P~23SER	295	-0.498	-0.498	-0.117	-0.381
P~24GLN	296	-0.018	-0.018	-0.157	0.138
P~25TYR	297	-8.053	-8.053	-10.726	2.673
P~26SER	298	-0.982	-0.982	-0.274	-0.708
P~27THR	299	-0.303	-0.303	-0.054	-0.25

P~28ASN	300	-0.173	-0.173	-0.209	0.036
P~29TRP	301	-8.749	-8.749	-4.633	-4.116
P~30SER	302	-0.18	-0.18	-0.047	-0.134
P~31ALA	303	-0.069	-0.069	-0.017	-0.052
P~32GLU	304	0.023	0.023	-0.012	0.035
P~33ARG	305	-0.353	-0.353	-0.054	-0.299
P~34SER	306	0.02	0.02	-0.012	0.033
P~35ARG	307	0.188	0.188	-0.008	0.196
P~36LEU	308	0.031	0.031	-0.004	0.035
P~37ASN	309	0.01	0.01	-0.004	0.014
P~38TYR	310	-0.06	-0.06	-0.016	-0.044
P~39PRO	311	-0.028	-0.028	-0.012	-0.017
P~40GLU	312	0.448	0.448	-0.041	0.489
P~41ASN	313	-0.347	-0.347	-0.23	-0.117
P~42GLY	314	0.016	0.016	-0.066	0.083
P~43TRP	315	-0.636	-0.636	-0.248	-0.389
P~44THR	316	-3.656	-3.656	-3.354	-0.301
P~45PRO	317	-7.554	-7.554	1.018	-8.572
P~46GLY	318	-2.875	-2.875	-2.484	-0.391
P~47GLU	319	-6.382	-6.382	-3.703	-2.679
P~48ASP	320	-25.796	-25.796	-5.801	-19.995
P~49SER	321	-0.567	-0.567	-0.366	-0.201
P~50TYR	322	0.156	0.156	-0.077	0.233
P~51ARG	323	1.052	1.052	-0.098	1.149
P~52GLU	324	-1.804	-1.804	-0.18	-1.623
P~53TRP	325	-0.084	-0.084	-0.036	-0.049
P~54ILE	326	-0.047	-0.047	-0.023	-0.024
P~55GLN	327	-0.043	-0.043	-0.006	-0.037
P~56VAL	328	-0.019	-0.019	-0.003	-0.016
P~57ASP	329	-0.255	-0.255	-0.001	-0.253
P~58LEU	330	0.009	0.009	-0.001	0.01
P~59GLY	331	0.007	0.007	0	0.007
P~60LEU	332	-0.012	-0.012	0	-0.012

P~61LEU	333	0.006	0.006	0	0.006
P~62ARG	334	0.194	0.194	0	0.195
P~63PHE	335	0	0	0	0
P~64VAL	336	0.011	0.011	0	0.012
P~65THR	337	-0.011	-0.011	0	-0.011
P~66ALA	338	0.01	0.01	-0.001	0.011
P~67VAL	339	-0.002	-0.002	-0.002	0
P~68GLY	340	-0.01	-0.01	-0.001	-0.008
P~69THR	341	0.078	0.078	-0.005	0.084
P~70GLN	342	-0.079	-0.079	-0.013	-0.066
P~71GLY	343	0.093	0.093	-0.016	0.109
P~72ALA	344	-0.122	-0.122	-0.076	-0.046
P~73ILE	345	-0.174	-0.174	-0.18	0.005
P~74SER	346	-1.49	-1.49	-0.923	-0.567
P~75LYS	347	-1.244	-1.244	-0.311	-0.934
P~76GLU	348	-0.669	-0.669	-3.171	2.502
P~77THR	349	-15.043	-15.043	-14.17	-0.873
P~78LYS	350	-2.967	-2.967	-2.719	-0.248
P~79LYS	351	-3.863	-3.863	-5.278	1.415
P~80LYS	352	0.149	0.149	-0.293	0.442
P~81TYR	353	-3.109	-3.109	-4.329	1.219
P~82TYR	354	-0.115	-0.115	-0.063	-0.052
P~83VAL	355	0.021	0.021	-0.038	0.058
P~84LYS	356	1.13	1.13	-0.022	1.152
P~85THR	357	-0.085	-0.085	-0.009	-0.076
P~86TYR	358	0.035	0.035	-0.01	0.045
P~87LYS	359	0.716	0.716	-0.006	0.722
P~88ILE	360	-0.002	-0.002	-0.003	0.001
P~89ASP	361	-0.72	-0.72	-0.002	-0.718
P~90VAL	362	-0.006	-0.006	-0.001	-0.006
P~91SER	363	0.003	0.003	-0.001	0.004
P~92SER	364	0.011	0.011	0	0.011
P~93ASN	365	-0.008	-0.008	0	-0.007

P~94GLY	366	-0.006	-0.006	0	-0.005
P~95GLU	367	-0.227	-0.227	-0.001	-0.226
P~96ASP	368	-0.26	-0.26	0	-0.26
P~97TRP	369	-0.009	-0.009	-0.002	-0.006
P~98ILE	370	-0.013	-0.013	-0.001	-0.012
P~99THR	371	0.03	0.03	-0.001	0.031
P~100ILE	372	-0.016	-0.016	-0.001	-0.015
P~101LYS	373	0.403	0.403	-0.001	0.404
P~102GLU	374	-0.448	-0.448	-0.001	-0.448
P~103GLY	375	-0.002	-0.002	0	-0.002
P~104ASN	376	-0.001	-0.001	0	-0.001
P~105LYS	377	0.347	0.347	0	0.347
P~106PRO	378	0.02	0.02	-0.001	0.02
P~107VAL	379	-0.014	-0.014	-0.001	-0.012
P~108LEU	380	0.036	0.036	-0.004	0.04
P~109PHE	381	-0.051	-0.051	-0.003	-0.048
P~110GLN	382	0.021	0.021	-0.003	0.024
P~111GLY	383	0.072	0.072	-0.003	0.074
P~112ASN	384	-0.067	-0.067	-0.008	-0.059
P~113THR	385	0.017	0.017	-0.006	0.023
P~114ASN	386	-0.056	-0.056	-0.011	-0.045
P~115PRO	387	-0.032	-0.032	-0.027	-0.005
P~116THR	388	-0.03	-0.03	-0.01	-0.021
P~117ASP	389	-0.514	-0.514	-0.004	-0.51
P~118VAL	390	0.015	0.015	-0.003	0.018
P~119VAL	391	-0.02	-0.02	-0.003	-0.017
P~120VAL	392	0.009	0.009	-0.001	0.011
P~121ALA	393	0.006	0.006	-0.001	0.007
P~122VAL	394	-0.023	-0.023	-0.001	-0.022
P~123PHE	395	0.011	0.011	-0.001	0.012
P~124PRO	396	0.014	0.014	0	0.015
P~125LYS	397	0.257	0.257	0	0.257
P~126PRO	398	0.002	0.002	0	0.002

P~127LEU	399	-0.001	-0.001	0	-0.001
P~128ILE	400	-0.007	-0.007	0	-0.006
P~129THR	401	0	0	0	0
P~130ARG	402	0.215	0.215	-0.001	0.216
P~131PHE	403	0.001	0.001	-0.001	0.002
P~132VAL	404	0.004	0.004	-0.002	0.006
P~133ARG	405	0.66	0.66	-0.006	0.666
P~134ILE	406	-0.049	-0.049	-0.011	-0.038
P~135LYS	407	1.257	1.257	-0.022	1.279
P~136PRO	408	-0.094	-0.094	-0.052	-0.042
P~137ALA	409	-0.084	-0.084	-0.018	-0.066
P~138THR	410	0.04	0.04	-0.029	0.069
P~139TRP	411	-0.144	-0.144	-0.437	0.293
P~140GLU	412	-1.756	-1.756	-0.126	-1.63
P~141THR	413	-0.789	-0.789	-0.905	0.116
P~142GLY	414	-0.174	-0.174	-0.772	0.598
P~143ILE	415	-1.382	-1.382	-1.058	-0.324
P~144SER	416	-0.102	-0.102	-0.259	0.157
P~145MET	417	-0.165	-0.165	-0.05	-0.115
P~146ARG	418	0.654	0.654	-0.026	0.68
P~147PHE	419	0	0	-0.008	0.009
P~148GLU	420	-0.409	-0.409	-0.002	-0.407
P~149VAL	421	0.006	0.006	-0.001	0.007
P~150TYR	422	-0.02	-0.02	-0.001	-0.019
P~151GLY	423	-0.001	-0.001	0	-0.001
P~152CYS	424	-0.006	-0.006	0	-0.006
P~153LYS	425	0.161	0.161	0	0.162
P~154ILE	426	0.003	0.003	0	0.004
P~155THR	427	-0.127	-0.127	0	-0.127
Total		-97.744	-97.744	-67.662	-30.082

Note: P~, Protein; MMPBSA, molecular mechanics poisson boltzmann surface area; MM, molecular mechanics; VDW, van der Waals energies; COU, coulombic electrostatic interaction energy.
